# Deletion of 9p drives B-ALL through heterozygous inactivation of *Pax5* and *Cd72* in preleukemic cells

**DOI:** 10.1172/jci.insight.199464

**Published:** 2026-02-17

**Authors:** Belén Ruiz-Corzo, Ana Casado-García, Ninad Oak, Paula Somoza-Cotillas, Andrea López-Álvarez de Neyra, Jorge Martínez-Cano, Alba Pérez-Pons, Elena G. Sánchez, Oscar Blanco, Diego Alonso-López, Javier De Las Rivas, Susana Riesco, Pablo Prieto-Matos, Francisco Javier García Criado, María Begoña García Cenador, Alberto Orfao, Manuel Ramírez-Orellana, César Cobaleda, Carolina Vicente-Dueñas, Kim E. Nichols, Isidro Sánchez-García

**Affiliations:** 1 Experimental Therapeutics and Translational Oncology Program, Instituto de Biología Molecular y Celular del Cáncer, CSIC-USAL, Campus M. de Unamuno s/n, Salamanca, Spain.; 2 Institute of Biomedical Research of Salamanca (IBSAL), Universidad de Salamanca, Campus Miguel de Unamuno s/n, Salamanca, Spain.; 3 Department of Oncology, St. Jude Children’s Research Hospital, Memphis, Tennessee, USA.; 4 Immune system development and function Unit, Centro de Biología Molecular Severo Ochoa (Consejo Superior de Investigaciones Científicas -Universidad Autónoma de Madrid), Madrid, Spain.; 5 Cancer Research Center (IBMCC, USAL-CSIC), Department of Medicine and Cytometry Service (NUCLEUS), University of Salamanca, Salamanca, Spain, and Biomedical Research Institute of Salamanca (IBSAL), Salamanca, Spain.; 6 Biomedical Research Networking Center Consortium (CIBERONC; CB16/12/00400), Madrid, Spain.; 7 Oncohematology Laboratory, Advance Terapy Unit, Fundación para la Investigación Biomédica del Hospital Universitario Niño Jesús (FIB HUNJ), Madrid, Spain.; 8 Departamento de Anatomía Patológica, Universidad de Salamanca, Salamanca, Spain.; 9 Bioinformatics Unit, and; 10 Bioinformatics and Functional Genomics Research Group, Cancer Research Center (CSIC-USAL), Salamanca, Spain.; 11 Department of Pediatrics, Hospital Universitario de Salamanca, Salamanca, Spain.; 12 Departamento de Cirugía, Universidad de Salamanca, Salamanca, Spain.; 13 Department of Pediatric Hematology and Oncology, Hospital Infantil Universitario Niño Jesús and Institute of Biomedical Research Hospital Universitario La Princesa, Madrid, Spain.

**Keywords:** Hematology, Oncology, Genetic risk factors, Leukemias, Mouse models

## Abstract

The contribution of 9p deletion to B cell acute lymphoblastic leukemia (B-ALL) has remained elusive since its discovery more than 40 years ago. Here we show that loss of *CD72* is recurrent in B-ALL cases containing *PAX5* deletions, and that *Cd72* haploinsufficiency drives B-ALL development in *Pax5^+/–^* mice. Mechanistically, *Cd72^+/–^*;*Pax5^+/–^* precursor B cells exhibited an inflammatory transcriptional profile characterized by a decrease in *Myd88* expression, a finding that aligns with our previous studies of B-ALL development in *Pax5^+/–^* mice following exposure to immune stressors. These combined genomic analyses and functional models provide compelling evidence that co-deletion of 2 contiguous genes, *Pax5* and *Cd72*, drives B cell leukemogenesis.

## Abstract 2

The contribution of 9p deletion in B-ALL has remained elusive since its discovery more than 40 years ago. We combined genomic analyses and functional models to show that the co-deletion of two contiguous genes, *Pax5* and *Cd72*, drives oncogenesis.

## Graphical abstract

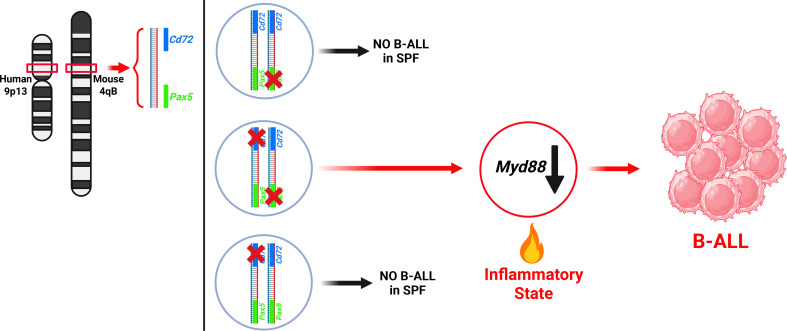

## Introduction

Deletion of the short arm of chromosome 9 (del9p) is a frequent karyotypic abnormality in B cell acute lymphoblastic leukemia (B-ALL). This deletion occurs in 10% of children with B-ALL where it has been associated with an unfavorable prognosis (1–7). Although first reported as a recurrent structural abnormality in B-ALL more than 40 years ago, the underlying molecular targets of this chromosomal event remain elusive (6, 8).

Chromosome 9p contains several neoplasia-associated genes, among them *PAX5* at 9p13.2, but also *CDKN2A* and *CDKN2B* at 9p21.3. Accordingly, leukemic cells harboring *PAX5* deletion almost always exhibit concurrent *CDKN2A* deletion (9). Hence, the current paradigm builds on the notion that, by eliminating the genes encoding the cell cycle inhibitors p16 (INK4a) and p14 (ARF), 9p deletions contribute to B-ALL by circumventing a CDKN2A-dependent proliferative block in precursor B cells. Nevertheless, several observations indicate that this might not be the case. Although these genes are considered to be tumor suppressor genes, mutation of the remaining *CDKN2A* allele is infrequent in B-ALL blasts (10, 11). Furthermore, in mice, which share synteny with humans in this chromosomal region, *Pax5* heterozygosity does not cooperate with concurrent *Cdkn2a* deletion to induce B-ALL development (12, 13). This latter observation suggests that the human locus may also contain as yet unidentified genes responsible for B-ALL development. In this study, we identified how *Cd72* loss cooperates with the deletion of *Pax5* to drive B-ALL development.

## Results

### Loss of CD72 is recurrent in B-ALL containing deletions of PAX5.

**Figure 1:**
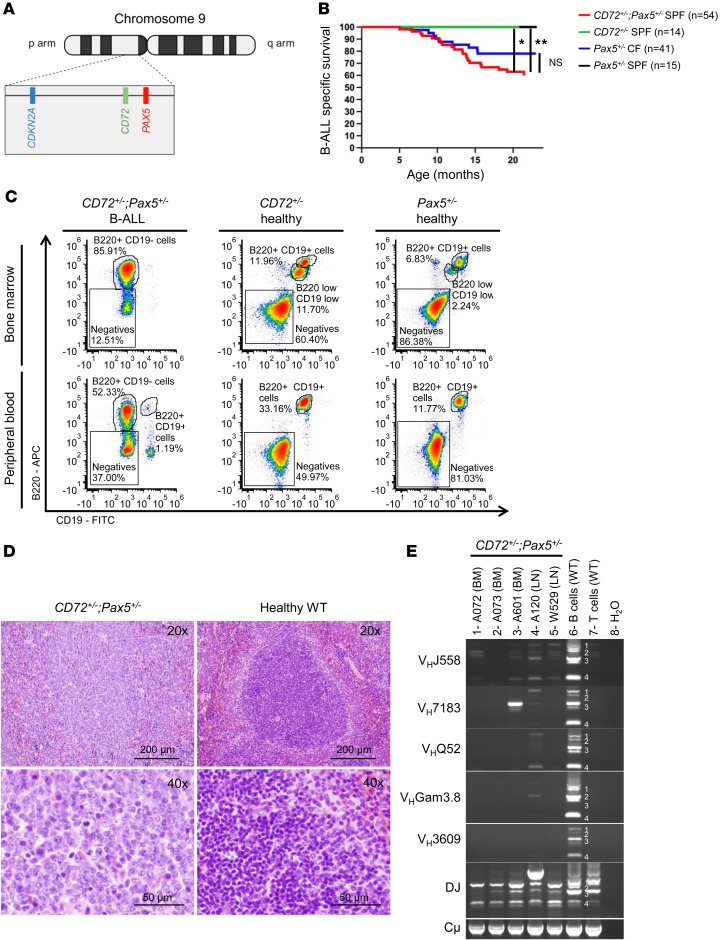
*Pax5* and *Cd72* heterozygosity cooperate in driving B-ALL. (**A**) Localization of *PAX5*, *CD72*, and *CDKN2A* on human chromosome 9. *PAX5* and *CDKN2A* are frequently deleted in human B-ALL. Created with BioRender. (**B**) B-ALL–specific survival curve of *Cd72^+/–^;Pax5^+/–^* mice maintained in a specific pathogen–free (SPF) facility (red, *n* = 54), compared with *Pax5^+/–^* maintained in a conventional facility (CF) (blue, *n* = 41; *P* = 0.1137), *Pax5^+/–^* mice maintained in an SPF facility (black, *n* = 15; **P = 0.0083), and *Cd72^+/–^* mice maintained in an SPF facility (green, *n* = 14; *P = 0.0120) mice. Differences in Kaplan-Meier survival plots of transgenic and WT mice were analyzed using the log-rank (Mantel-Cox) test. (**C**) Flow cytometric analysis of the bone marrow and peripheral blood of a *Cd72^+/–^;Pax5^+/–^* (B834) mouse with leukemia. Representative plots of cell subsets are shown, alongside comparisons with healthy *Cd72^+/–^* (B924) and *Pax5^+/–^* (S907) mice. (**D**) Splenic H&E staining of *Cd72^+/–^;Pax5^+/–^* (A120) leukemic mouse shows infiltration of blast cells in spleen. Loss of normal architecture due to infiltrating leukemic cells can be seen. Tissues from a control littermate WT mouse are shown for reference. Scale bars: 100 μm (top) and 50 μm (bottom). (**E**) PCR analysis of immunoglobulin heavy-chain gene rearrangements in leukemia-infiltrated bone marrow (BM) and lymph nodes (LNs) of *Cd72^+/–^;Pax5^+/–^* mice with the disease (*n* = 5). Thymocytes (T cells) were included as a negative control, and sorted CD19^+^ B cells (B cells) from the spleens of healthy mice were included as a positive control for polyclonal rearrangements within the mature B cell population (indicated by numbers 1–8).

Consistent with the notion that the 9p locus might contain unidentified genes responsible for B-ALL development, molecular studies of B-ALL in mouse models in which the disease arises after exposure to environmental triggers (12–14) have identified deletions affecting *Pax5* without concurrent involvement of *Cdkn2a* (Supplemental Figure 1A; supplemental material available online with this article; https://doi.org/10.1172/jci.insight.199464DS1). Of note, the *CD72* gene is contiguous with *Pax5* and juxtaposed between *CDKN2A* and *PAX5* in humans (Figure 1A and Supplemental Figure 1). Therefore, *CD72* is always deleted in the leukemic cells from patients with B-ALL that harbor *CDKN2A* and *PAX5* deletions. CD72 is a co-receptor of the B cell receptor (BCR), and it is expressed during all stages of B cell development (except on plasma cells), where it plays both positive and negative roles in regulating B cell function (15). These observations suggest that its loss might cooperate with deletions of *PAX5* to drive B-ALL development. However, whether the concomitant loss of *PAX5* and *CD72* can be responsible for B-ALL development remains unexplored.

### Cd72 haploinsufficiency drives B-ALL development in Pax5^+/–^ mice.

**Figure 2:**
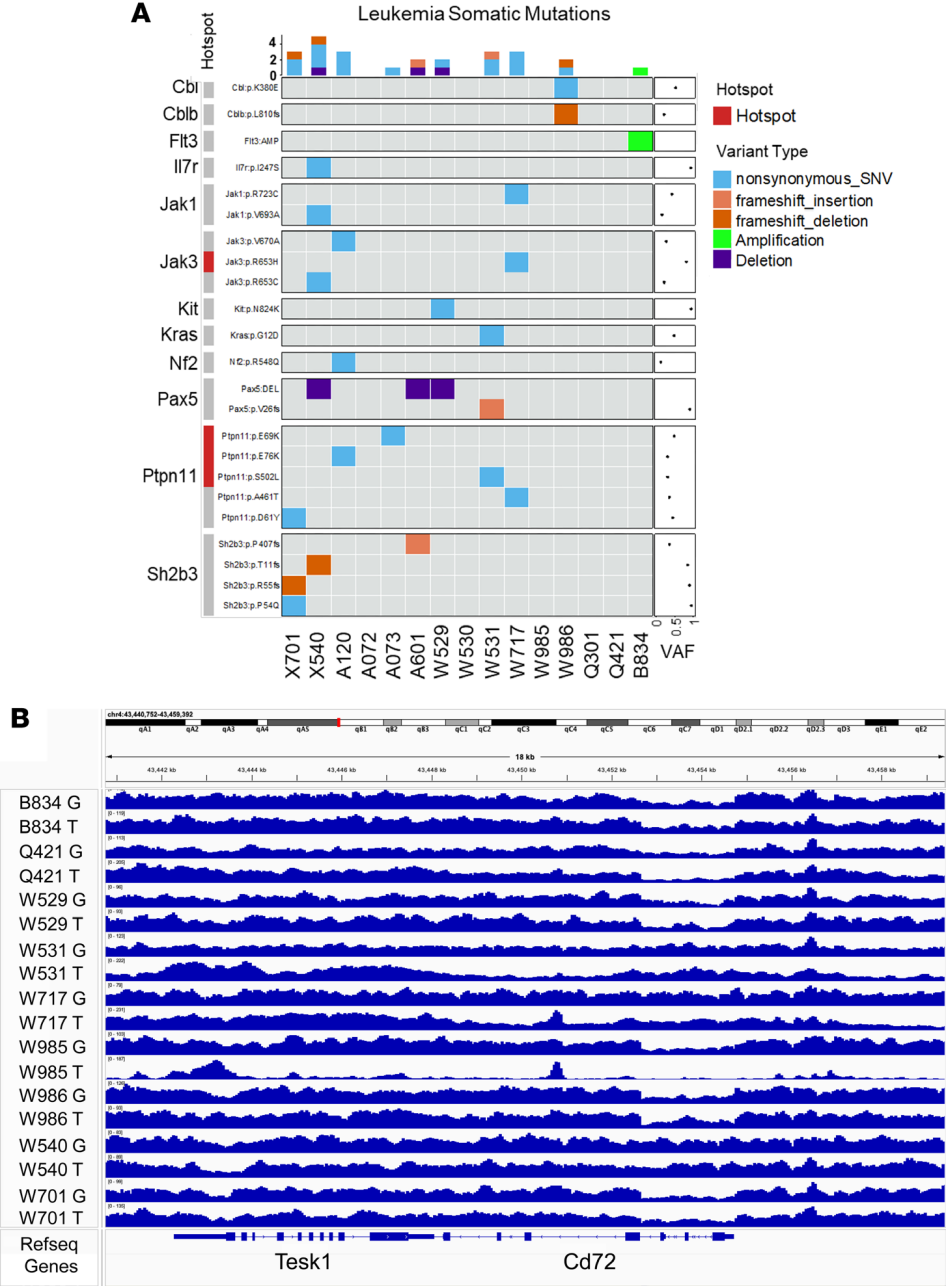
Genetic changes in B-ALL blasts from *Cd72^+/–^;Pax5^+/–^* mice. (**A**) Whole-genome sequencing (WGS) of leukemic blasts from *Cd72^+/–^;Pax5^+/–^* mice. Oncoprint summarizing somatic single nucleotide variants (SNVs) and copy number alterations across 15 leukemia samples from *Cd72^+/–^*;*Pax5^+/–^* mice. Alterations are grouped by gene. Tumor DNA was extracted from whole leukemic bone marrow, with matched tail DNA serving as the germline reference. Known hotspot mutations previously reported in human or mouse leukemia are highlighted in red. The mean variant allele frequency (VAF) for each SNV is displayed in the dot plot to the right. (**B**) Visualization of somatic alterations in leukemic *Pax5^+/–^*;*Cd72^+/–^* mice. No somatic *Cd72* alterations were found in all the *Pax5^+/–^*;*Cd72^+/–^* mice analyzed by WGS, except the germline deletion for first 4 exons as expected. G, germline DNA; T, tumor DNA.

To functionally investigate the effects of single or combined *Pax5* and *Cd72* haploinsufficiency, we generated cohorts of *Cd72^+/–^* mice, *Pax5^+/–^* mice, and *Cd72^+/–^*;*Pax5^+/–^* double heterozygous mice by interbreeding, and examined whether *Cd72^+/–^*;*Pax5^+/–^* animals were prone to B-ALL development. Individual *Pax5^+/–^* or *Cd72^+/–^* mice, housed in specific pathogen–free (SPF) conditions, did not spontaneously develop B-ALL after 2 years, corroborating previous results (12, 13). However, B-ALL development was observed in 38.89% (21 of 54) of *Cd72^+/–^*;*Pax5^+/–^* animals maintained in SPF conditions (Supplemental Figure 2). The appearance of leukemia in *Cd72^+/–^*;*Pax5^+/–^* mice occurred between 5 and 20 months of age (Figure 1B). B-ALL manifested with splenomegaly, disruption of splenic architecture due to blast infiltration, and appearance of blast cells in the peripheral blood (PB) (Figure 1, C and D). FACS analysis revealed a CD19^–^B220^+^IgM^–^CKIT^+/–^CD25^+/–^ phenotype of tumor cells, which extended through the bone marrow (BM), PB, spleen, and lymph nodes (LNs) (Figure 1C and Supplemental Figure 3) and also infiltrated nonlymphoid tissues, including the uterus, liver, kidney, lung, pancreas, and small intestine (Supplemental Figure 4). All B-ALL cells displayed clonal immature BCR rearrangement (Figure 1E). Similar to B-ALL cells generated in *Pax5^+/–^* mice following exposure to inflammatory stimuli (12, 13, 16–19), B-ALL blasts developing in *Cd72^+/–^*;*Pax5^+/–^* mice were also able to grow in the absence of IL-7 and to propagate the disease in secondary recipients. These leukemic *Cd72^+/–^*;*Pax5^+/–^* B-ALL cells were isolated from the BM of diseased mice, as B220^+^, by magnetic-activated cell sorting (MACS), and then cultured in medium containing IL-7 and, later, propagated in IL-7–independent culture conditions (9, 16). These leukemic *Cd72^+/–^*;*Pax5^+/–^* B-ALL cells were injected into syngenic mice (*n* = 10), and all animals developed B-ALL by day 14 when the disease was confirmed by the presence of blast cells in the PB. To identify the somatically acquired second hits leading to B-ALL development in *Cd72^+/–^*;*Pax5^+/–^* mice, we performed whole-genome sequencing of paired blood and germline tail DNA samples from 15 *Cd72^+/–^*;*Pax5^+/–^* animals. The percentage of leukemic cells in the sequenced blood samples ranged from 55% to 96%. We identified several somatically acquired recurrent mutations and copy number variations (CNVs) involving B cell transcription factors and key surface receptors in the blasts from diseased *Cd72^+/–^*;*Pax5^+/–^* mice (Figure 2 and Supplemental Figure 5), and mutations affecting the JAK/STAT and RAS signaling pathways were detected (Figure 2A). These patterns were similar to those previously observed in the leukemic cells from *Pax5^+/–^* mice following exposure to inflammatory stimuli or antibiotic treatment and in human B-ALL samples (12, 13, 16–19). However, there were no somatic genetic changes (either CNVs or single nucleotide variants [SNVs]) affecting the *Cdkn2a* genes and wild-type (WT) *Cd72* allele (Figure 2B). Altogether, these results strongly suggest that haploinsufficiency of *Pax5* and *Cd72* is the true driver of B-ALL in cells harboring del9p.

### Cd72^+/–^;Pax5^+/–^ precursor B cells exhibit an inflammatory transcriptional profile.

**Figure 3:**
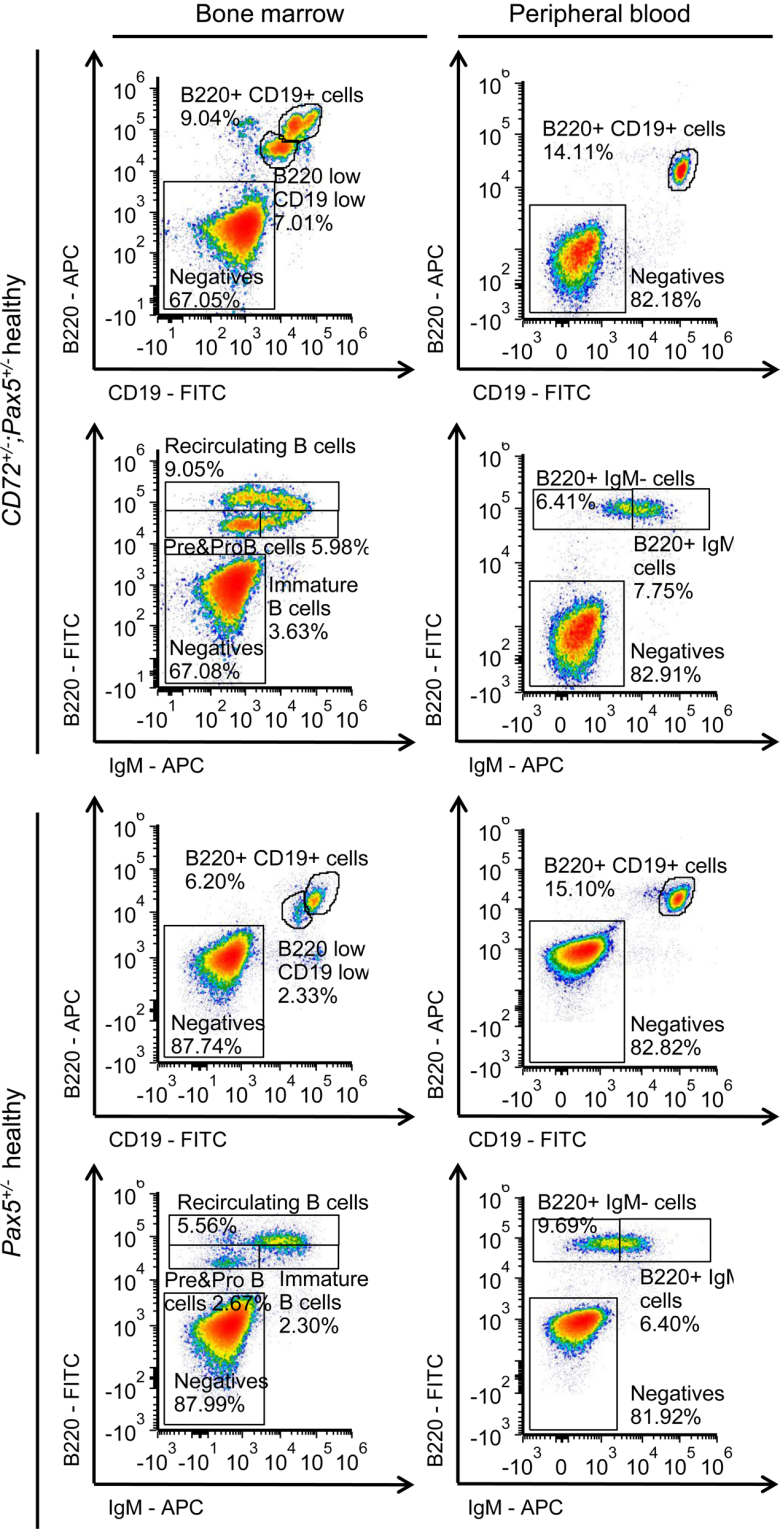
B cell differentiation of precursor B cells from *Cd72^+/–^*;*Pax5^+/–^* mice compared to *Pax5^+/–^* mice. Flow cytometric analysis of hematopoietic subsets in healthy *CD72^+/–^;Pax5^+/–^* mice compared to *Pax5^+/–^* mice. Representative plots of cell subsets from the bone marrow (BM) and peripheral blood (PB) are shown.

**Figure 4:**
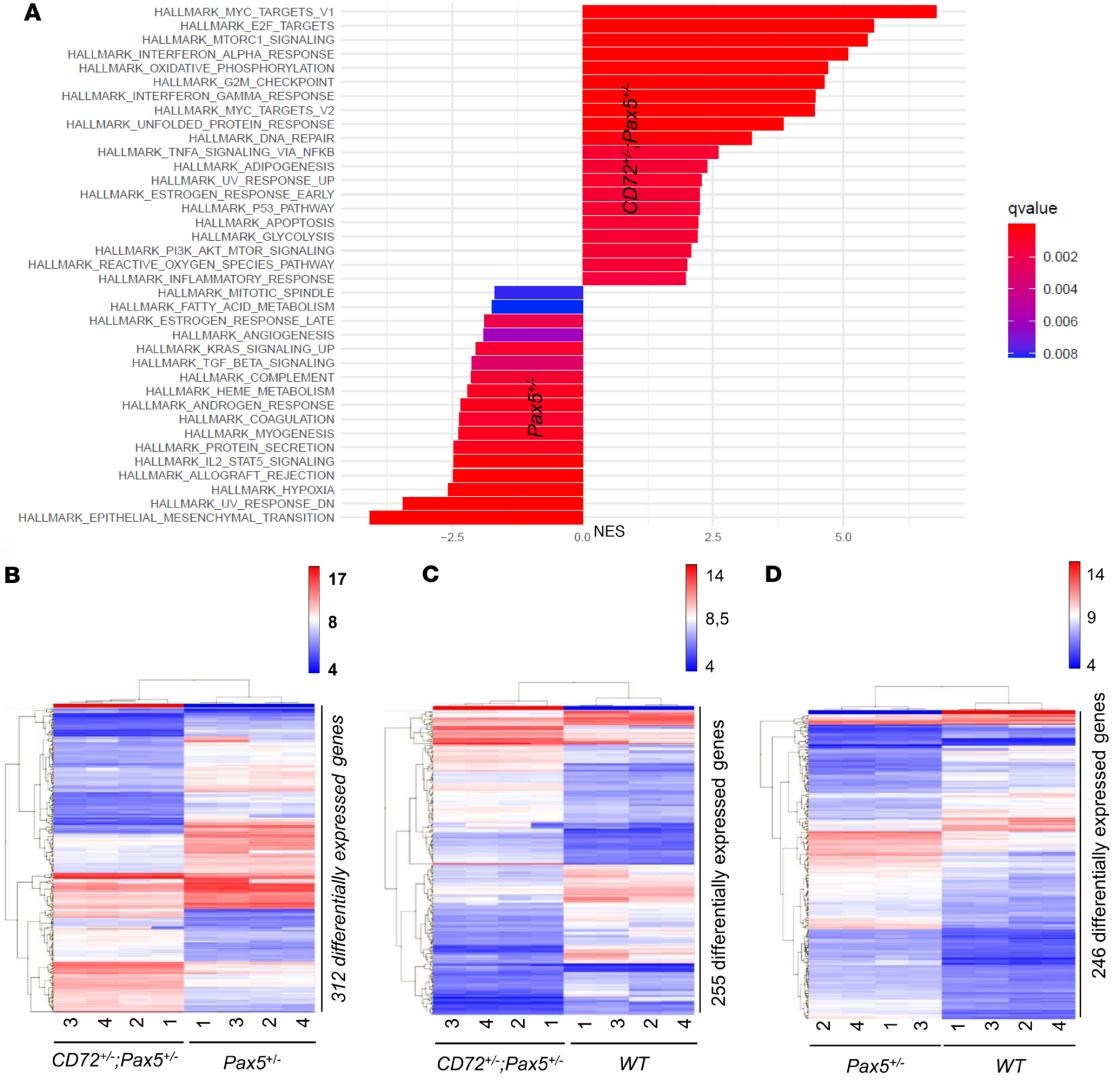
*CD72^+/–^*;*Pax5^+/–^* preleukemic cells are distinct from *Pax5^+/–^* or WT pro-B cells. (**A**) GSEA comparing *Cd72^+/–^;Pax5^+/–^* and *Pax5^+/–^* preleukemic pro-B cells in order to identify potential signatures of gene expression associated with different biological processes. The bar plot displays the normalized enrichment scores (NES) for Hallmark gene sets significantly enriched between the 2 cell populations. Positive NES values (right side) indicate gene sets enriched in *Cd72^+/–^;Pax5^+/–^* cells, while negative NES values (left side) indicate gene sets enriched in *Pax5^+/–^* cells. Bars are colored by the false discovery rate (FDR) *q* value, with the most statistically significant gene sets shown in red and less significant in blue, as indicated by the gradient scale. *Cd72^+/–^;Pax5^+/–^* cells exhibit strong enrichment for proliferative and metabolic pathways such as MYC, E2F, p53, and MTORC1. In contrast, *Pax5^+/–^* cells are enriched for pathways associated with immune signaling, differentiation, and stress responses, including complement system, IL-2/STAT5 signaling, epithelial-mesenchymal transition, and hypoxia hallmarks. Only gene sets with *q* values of less than 0.01 are displayed. These findings suggest distinct transcriptional programs underpin the biological differences between *Cd72^+/–^;Pax5^+/–^* and *Pax5^+/–^* populations. (**B**–**D**) Transcriptomic analysis of the indicated genotypes shows genes that were significantly induced or repressed within preleukemic B cells (bone marrow B220^+^ cells) of *CD72^+/–^*; *Pax5^+/–^* (**B** and **C**) and *Pax5^+/–^* (**D**) mice compared with *Pax5^+/–^* or WT cells. This was determined using significance analysis of microarrays with an |FC| of greater than 2 and *P* value of less than 0.05. Each row represents a separate gene, and each column denotes a separate mRNA sample. The level of expression of each gene in each sample is represented using a red–blue color scale (upregulated genes are displayed in red and downregulated genes in blue).

**Figure 5:**
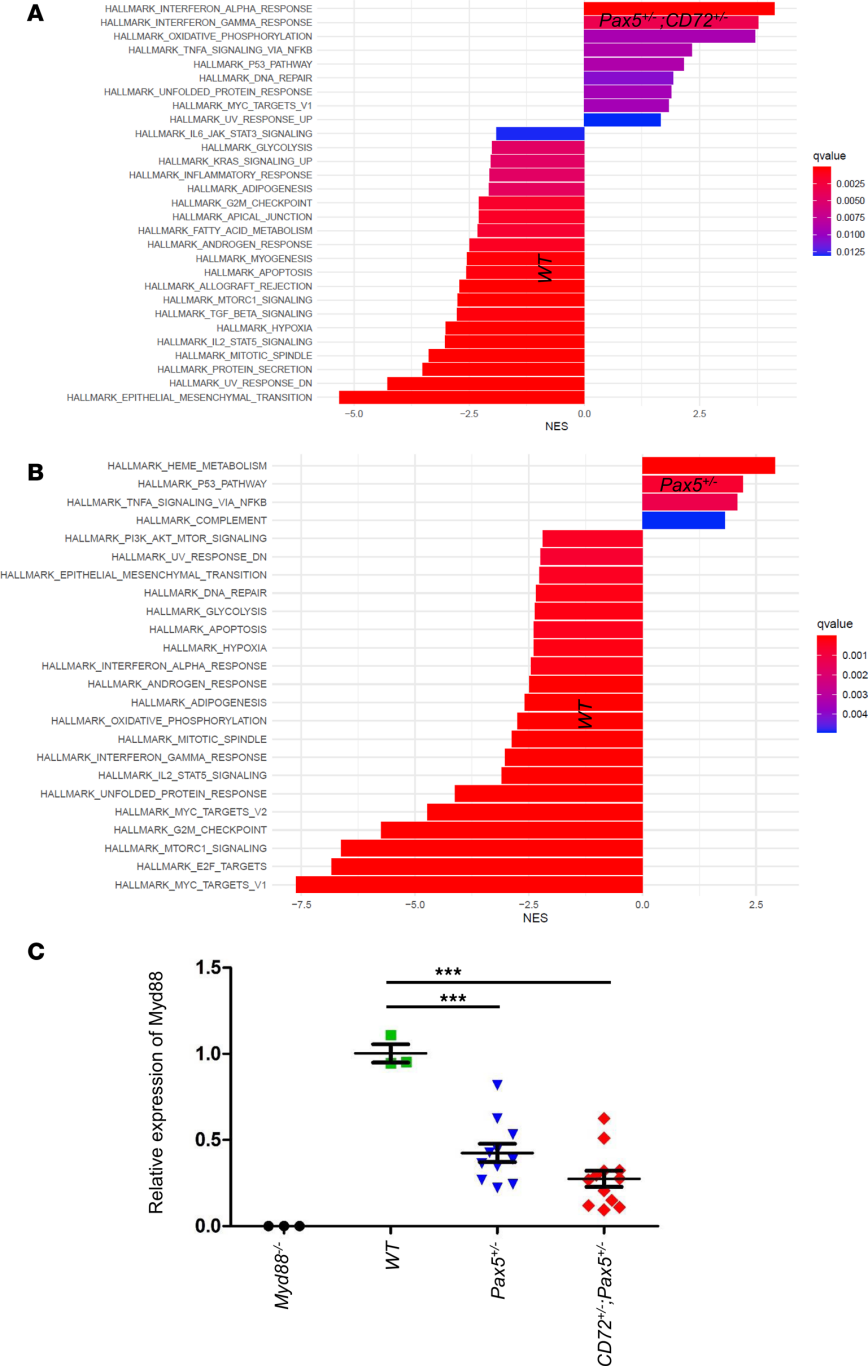
Pathways transcriptionally enriched in preleukemic *CD72^+/–^;Pax5^+/–^* and *Pax5^+/–^* cells. GSEA of Hallmark pathways comparing *CD72^+/–^;Pax5^+/–^* and WT pro-B cells (**A**) or *Pax5^+/–^* and WT pro-B cells (**B**). Bar plot displays the normalized enrichment scores (NES) for significantly enriched Hallmark gene sets, comparing the 2 experimental conditions indicated in each case. Gene sets with positive NES values (right side) are enriched in the *CD72^+/–^;Pax5^+/–^* or the *Pax5^+/–^* group, while those with negative NES values (left side) are enriched in the WT group. The bars are color coded according to the false discovery rate (FDR) *q* values, with more significant gene sets shown in red and less significant ones in blue, as indicated by the color gradient legend. Only pathways with FDR *q* values below the significance threshold are shown (*q* < 0.05). (**C**) *CD72* heterozygosity promotes *Myd88* downregulation in *CD72^+/–^;Pax5^+/–^* pro-B cells. *Myd88* mRNA expression was quantified by qPCR in cultured *CD72^+/–^;Pax5^+/–^* (*n* = 4) and *Pax5^+/–^* (*n* = 4) pro-B cells. Total bone marrow from *Myd88^−/−^* mice was included as a negative control, and bone marrow pro-B cells from WT mice (*n* = 3) were used as a reference. Each dot represents an individual sample, and bars indicate the mean ± SEM. Data were analyzed by 1-way ANOVA (***P < 0.0001) followed by Dunnett’s multiple-comparison test versus the control group (WT pro-B cells), and multiplicity-adjusted *P* values are reported.

To elucidate how the combined loss of these 2 genes might contribute to B-leukemogenesis, we next examined the effects of *Cd72* downregulation in preleukemic B cells by examining B cell differentiation and transcriptional profiles of precursor B cells from *Cd72^+/–^*;*Pax5^+/–^* mice compared to *Pax5^+/–^* mice. We observed no differences in B cell developmental compartments in the BM and PB (Figure 3). Transcriptional profiling of *Cd72^+/–^*;*Pax5^+/–^* preleukemic precursor B cells revealed significant enrichment of inflammatory pathways and, as expected, downregulation of *Cd72* (Figure 4 and Supplemental Tables 1–3). These results are in agreement with prior in vitro studies showing that proinflammatory cytokines like IL-6, IL-1β, and TNF-α (20), or TGF-β–dependent signaling (21, 22), can promote the malignant transformation of preleukemic B cells. *Cd72^+/–^;Pax5^+/–^* cells exhibited strong enrichment for transcriptomic signatures characteristic of proliferative and metabolic pathways such as MYC, E2F, p53, and MTORC1. In contrast, *Pax5^+/–^* cells were enriched for pathways associated with immune signaling, differentiation, and stress responses, including complement system, IL-2/STAT5 signaling, epithelial-mesenchymal transition, and hypoxia hallmarks. This scenario agrees with the identification of reactive oxygen species (ROS) signatures associated with the *Pax5alt* B-ALL subtype in human patients (23, 24). In addition, the expression of genes involved in the IFN-α response, TNF-α signaling via NF-κB, and IFN-γ response was augmented in *Cd72^+/–^*;*Pax5^+/–^* preleukemic precursor B cells (Figure 4A and Figure 5, A and B), a finding that is compatible with the *Myd88* downregulation observed in *Cd72^+/–^*;*Pax5^+/–^* preleukemic cells (Figure 5C). This finding also conforms with previous results showing that *Myd88* deficiency results in tissue-specific changes in cytokine induction and inflammation in infected mice (25).

### Longitudinal profiling of proinflammatory cytokines uncovers patterns of dysregulation and associations with B-ALL development in Cd72^+/–^;Pax5^+/–^ mice.

To determine whether the transformation of preleukemic B cells to full-blown B-ALL in *Cd72**^+/–^***;*Pax5**^+/–^*** mice is in part due to dysregulated expression of inflammatory cytokines in *Cd72**^+/–^***;*Pax5**^+/–^*** mice, we measured concentrations of 7 key inflammatory cytokines (IL-2, IL-4, IL-6, IL-10, IL-17a, TNF, and IFN-γ) in the serum of *Cd72**^+/–^***;*Pax5**^+/–^*** mice that developed B-ALL, of *Cd72**^+/–^***;*Pax5**^+/–^*** mice without B-ALL, and of age-matched healthy *Pax5**^+/–^*** mice. We observed that IL-6 and IL-10 cytokine dysregulation was detectable in serum samples taken at routine intervals at a healthy (non-leukemic) stage in *Cd72**^+/–^***;*Pax5**^+/–^*** mice that would later develop B-ALL, prior to the first phenotypic signs of illness (Supplemental Figure 6). Thus, induction of a leukemogenic state is associated with an increase in IL-6 cytokine secretion in *Cd72**^+/–^***;*Pax5**^+/–^*** mice. These results are in agreement with in vitro studies showing that proinflammatory cytokines like IL-6, IL-1β, and TNF-α (20) or TGF-β–dependent signaling (21, 22) promote malignant transformation of preleukemic B cells.

### Reduced expression of Myd88 in preleukemic Cd72^+/–^;Pax5^+/–^ precursor B cells.

Transcriptional analyses suggested a dysregulation of MyD88-dependent inflammatory pathways in *Cd72^+/–^*;*Pax5^+/–^* preleukemic B cells. To this end, we have previously shown that reduced Myd88 expression in *Pax5^+/–^* precursor B cells is a driver of clonal evolution to B-ALL (18). This effect of reduced Myd88 expression on leukemia progression is independent of infection, as *Myd88^+/–^;Pax5^+/–^* mice develop leukemia even when the mice are kept in an SPF facility. To elucidate whether reduction of Myd88 expression is involved in the transition of premalignant *Cd72^+/–^*;*Pax5^+/–^* B cells to B-ALL, we next performed a real-time PCR (qPCR) quantification using pro-B cells from *Cd72^+/–^*;*Pax5^+/–^* and *Pax5^+/–^* mice. Compared with pro-B cells from *Pax5^+/–^* mice, *Myd88* expression was significantly decreased in *Cd72^+/–^*;*Pax5^+/–^* cells (Figure 5C). Altogether, these findings reveal that progression to B-ALL in the context of *Cd72* and *Pax5* haploinsufficiency is leads to an inflammatory state characterized by a decrease in *Myd88* expression, a pattern resembling that observed in the B-ALL triggered by infection exposure or antibiotic treatment of *Pax5^+/–^* mice (12, 13, 16–19).

## Discussion

Our study clarifies the long-debated contribution of del9p to B-ALL pathogenesis by identifying combined *Pax5* and *Cd72* haploinsufficiency as a functional driver of leukemogenesis (1–5). Integrated genomic analyses of focal deletions in mouse B-ALLs identified 2 contiguous downregulated genes in 9p, *Cd72* and *Pax5*. Consistent with the absence of recurrent point mutations affecting *PAX5* or *CD72*, our findings support a model in which del9p promotes leukemogenesis primarily through combined haploinsufficiency rather than through sequential mutational inactivation. Here, we demonstrate that the co-deletion is truly the driver of B-ALL development. Importantly, the B-ALL development was seen only when both genes were co-deleted under SPF conditions. B-ALL development is never seen in single *Pax5^+/–^* mice when they are kept under an SPF environment (12, 13, 16–19). However, B-ALL appears in single *Pax5^+/–^* mice when they are exposed to infections by keeping *Pax5^+/–^* mice in conventional cages (12, 13, 16–19). These results demonstrate the tumor suppressor nature of the global *CD72-PAX5* region, but not of either gene alone. However, these data do not rule out putative roles for other genes, including *CDKN2A*. Mechanistically, *Cd72^+/–^*;*Pax5^+/–^* preleukemic cells show an inflammatory transcriptional profile characterized by a decrease in *Myd88* expression, a finding that aligns with our previous studies in *Pax5^+/–^* mice where B-ALL development only occurs following exposure to immune stressors (12, 13, 16–19), in which we have shown that genetic reduction of *Myd88* expression alone is sufficient to trigger B-ALL development in *Pax5^+/–^* mice kept under SPF conditions, a scenario where single *Pax5^+/–^* mice never develop B-ALL (18). These results identify therapeutic targets against del9p B-ALL progression, providing opportunities for the treatment and possibly also the prevention of del9p B-ALL. In conclusion, our results clarify the role played by 9p deletion in B-ALL oncogenesis and identify *Pax5* and *Cd72* as tumor suppressors in the context of this deletion. We believe that our findings provide critical insights into the pathogenesis of 9p deletion in leukemia and the future development of better therapies.

## Methods

### Sex as a biological variable.

Sex was not considered as a biological variable.

### Mice.

*Pax5^+/–^* (26), *Cd72^+/–^* (MMRRC:030139-MU [B6.129P2-Cd72tm1Jrp/Mmmh]) (27), and control WT mice of a mixed C57BL/6 × CBA background were born and kept at the SPF facility. *Pax5^+/–^* mice were crossed with *Cd72^+/–^* mice to generate animals of genotype *Cd72^+/–^*;*Pax5^+/–^* mice. The genetic background of the mice was tested using the miniMUGA array (28) performed through Transnetyx, confirming the presence of a mixed C57BL/6 × CBA/J hybrid background with a percentage of C57BL/6 ranging from 40.4% to 75.5%. There is a very small amount of 129 sequence in the immediate proximity of the mutated sequences of *Pax5* and *Cd72* genes in chromosome 4, a result of the fact that the knockouts were originally generated in 129 ES cells, and the selection for the mutated allele has kept this very small portion of 129 sequence in this region, but it is negligible in the total of the genome (0.2%–0.4%) and only in this region (see Supplemental Table 4 and Supplemental Data Set). Only a cohort of *Pax5^+/–^* mice was exposed to conventional pathogens present in non-SPF animal facilities (conventional facility, CF), as previously described (12). Housing environmental conditions included a temperature of 21°C ± 2°C, humidity of 55% ± 10%, and a 12-hour/12-hour light/dark cycle. Mice had access to food and water ad libitum. During housing, animals were monitored daily for health status. No data were excluded from the analyses. The inclusion criteria were based on the genotype of the mouse: transgenic versus control littermate. The experiments were not randomized and the investigators were not blinded to group allocation during experiments and outcome assessment. Upon signs of disease, mice were sacrificed and subjected to standard necropsy procedures. All major organs were examined under the dissecting microscope. Tissue samples were taken from homogeneous portions of the resected organ and fixed immediately after excision. The differences in the Kaplan-Meier survival plots of all cohorts of mice under study were analyzed using the log-rank (Mantel-Cox) test.

### Flow cytometry.

Nucleated cells were obtained from total mouse BM (flushing from the long bones), PB, thymus, LNs, or spleen. To prepare cells for flow cytometry, contaminating red blood cells were lysed with RCLB lysis buffer RCLB lysis buffer (0.155M NH_4_CL, 10mM KHCO_3_, 10mM EDTA; ph 7.4 [with 2M HCL]) and the remaining cells were then washed in PBS with 1% FCS. Nonspecific antibody binding was suppressed by preincubation of cells with CD16/CD32 Fc-block solution (BD Biosciences) at a dilution of 1:100 for 5 minutes at room temperature. For each analysis, a total of at least 50.000 viable (PI^–^) cells were assessed. Later, samples were stained with the mix of antibodies detailed below for 20 minutes at 4°C in the dark. Prior to acquisition in the flow cytometer, samples were washed with PBS with 1% FCS to eliminate the excess of antibody and then resuspended in PBS with 1% FCS containing 10 μg/mL PI to exclude dead cells from further analysis. The samples and the data were acquired in a Cytek Northern Lights 2000 spectral cytometer (2 lasers, red and blue) and analyzed with FCS Express software. Specific fluorescence of the fluorophores, as well as known forward and orthogonal light scattering properties of mouse cells, were used to establish gates. The gating strategy used in FACS analysis is shown in Supplemental Figure 7.

The following antibodies and concentrations were used for flow cytometry: anti-CD8a (BioLegend 100708; 1:250, clone 53-6.7), anti-CD4 (BioLegend 100516; 1:250, clone RM4-5), anti-CD25 (BD 553866; 1:500, clone PC61), anti-CD11b (BioLegend 101208; 1:200, clone M1/70), anti-CD19 (BioLegend 152404; 1:100, clone 1D3/CD19), anti-B220 (BioLegend 103206; 1:100, clone RA3-6B2; BioLegend 103212, 1:100, clone RA3-6B2), anti-IgM (BioLegend 406509; 1:100, clone RMM-1), anti-Ly6G/Ly6C (BioLegend 108412; 1:100, clone RB6-8C5), anti-CD117 (BioLegend 105805; 1:200, clone 2B8), anti-CD45 (BD 559864; 1:100, clone 30-F1 1), and anti-Ter119 (BioLegend 116208; 1:100, clone TER-199).

### Histology.

Animals were sacrificed by cervical dislocation; tissue samples were formalin-fixed and included in paraffin. Pathology assessment was performed on H&E-stained sections under the supervision of Oscar Blanco, an expert pathologist at the Salamanca University Hospital (Spain).

### V(D)J recombination.

Immunoglobulin rearrangements were amplified by PCR using the primers indicated in Table 1. Cycling conditions consisted of an initial heat activation at 95°C followed by 31–37 cycles of denaturation for 1 minute at 95°C, annealing for 1 minute at 65°C, and elongation for 1 minute, 45 seconds at 72°C. This was followed by a final elongation for 10 minutes at 72°C.

### Microarray data analysis.

The total RNA was first isolated using TRIzol (Life Technologies), and then it was subjected to purification with the RNeasy Mini Kit (Qiagen) using also the On-Column DNase treatment option. Quality and quantification of RNA samples were determined by electrophoresis.

Determination of the expression of the different genes in the RNA samples was performed using Affymetrix Mouse Gene 1.0 ST arrays. All bioinformatic analyses of the array data were performed using Applied Biosystems Transcriptome Analysis Console (TAC) software (v4.0.3). This software was utilized to identify differentially expressed genes by comparing the expression levels between different conditions. To determine statistical significance, a threshold of *P* value less than 0.05 and |fold change| (|FC|) greater than 2 was applied.

### Enrichment analysis.

In order to identify potential signatures of gene expression associated with different biological processes, gene set enrichment analysis (GSEA) was performed using the clusterProfiler suite of R packages (29–31). Gene sets were obtained from the Molecular Signatures Database (MSigDB) (32).

### Whole-genome sequencing.

Tumor DNA was derived from the BM where the percentage of blasts cells were between 55% and 96% in each B-ALL and germline control DNA was obtained from the tail when the mice were 4 weeks old. Genomic DNA libraries were prepared from sheared DNA with the HyperPrep Library Preparation Kit (Roche, 07962363001). Paired-end 150-cycle sequencing was performed on a NovaSeq 6000 (Illumina). Illumina paired-end reads were preprocessed and were mapped to the mouse reference genome (mm10) with BWA (33). We used an ensemble approach to call somatic mutations (SNV/indels) with multiple published tools, including Mutect2 (34), SomaticSniper (35), VarScan2 (36), MuSE (37), and Strelka2 (38). The consensus calls by at least 2 callers were considered as confident mutations. The consensus call sets were further manually reviewed for the read depth, mapping quality, and strand bias to remove additional artifacts. Somatic CNVs were determined by CNVkit (39). For somatic structural variants (SV), 4 SV callers were implemented in the workflow for SV calling, including Delly (40), Lumpy (41), Manta (42), and Gridss (43). The SV calls passing the default quality filters of each caller were merged using SURVIVOR (44) and genotyped by SVtyper (45). The intersected call sets were manually reviewed for the supporting soft-clipped and discordant read counts at both ends of a putative SV site using IGV (https://igv.org/).

### Pro-B cell culture.

Pro-B cell culture was carried out as previously described (12, 13, 16–19). Pro-B cells were purified from BM using MACS, selecting with anti-B220 beads (Milteny Biotec). Pro-B cells were maintained and expanded by culturing them in Iscove’s modified Dulbecco’s medium (IMDM) supplemented with 50 μM β-mercaptoethanol, 1 mM L-Gln, 2% heat-inactivated FCS, 1 mM penicillin-streptomycin (BioWhittaker), 0.03% (w/v) primatone RL (Sigma-Aldrich), and 5 ng/mL mrIL-7 (R&D Systems), in the presence of mitomycin C–treated ST2 feeder cells. Tumor pro-B cells that could grow independently of IL-7 were grown in the same medium without this cytokine.

### Transplantation.

Transplantation was carried out as previously described (12, 13, 16–19). IL-7–independent leukemic pro-B cells were intravenously injected into 12-week-old male syngenic (C57BL/6 × CBA) mice that had previously been sublethally irradiated (4 Gy). Leukemia development in the injected mice was confirmed by the presence of blast cells in PB.

### Quantification of cytokine levels in serum.

Quantification of cytokine levels in serum was carried out as previously described (41). Serum cytokine levels were analyzed using the Cytometric Bead Array immunoassay system (CBA) (BD Biosciences), which simultaneously assesses IL-2, IL-4, IL-6, IL-10, IL-17A, TNF-α, and IFN-γ in serum from the mice (Mouse Th1 Th2 Th17 Cytokine Kit; BD Biosciences, 560485). Data acquisition was performed on a FACSCanto II flow cytometer (BD Biosciences) using the FACSDiva software program (BD Biosciences). For the evaluation of cytokine serum levels or cytokine secretion into the culture supernatants, 50 μL of serum was collected. Briefly, 50 μL of the serum was incubated at room temperature for 2 hours at room temperature with 50 μL of anti-cytokine mAb–coated beads and with 50 μL of the appropriate phycoerythrin-conjugated (PE-conjugated) anticytokine antibody detector. After this incubation period, samples were washed once (5 minutes at 200*g*) in order to remove the excess of detector antibodies. Immediately afterwards, data acquisition was performed on a FACSCanto II flow cytometer (BD Biosciences) using the FACSDiva software program (BD Biosciences). During acquisition, information was stored for 3,000 events corresponding to each bead population analyzed per sample (total number of beads >9,000). For data analysis, the FCAP Array software v3.0 program (BD Biosciences) was used. The levels of cytokines are listed in Supplemental Table 5.

### qPCR of Myd88.

cDNA for use in qPCR studies was synthesized using reverse transcriptase (SuperScript VILO cDNA Synthesis Kit; Invitrogen). qPCR reactions were performed in an Eppendorf MasterCycler Realplex machine. Assays used for qPCR were from Integrated DNA Technologies: Myd88 (assay ID: Mm.PT.58.8716051.gs) and GAPDH (assay ID: Mm.PT.39a.1). In addition, the probes were designed so that genomic DNA would not be detected during the PCR. Measurement of GAPDH gene product expression was used as an endogenous control. Total bone marrow from *Myd88^–/–^* mice (B6.129P2(SJL)-Myd88^tm1.1Defr^/J) were used as negative control. All samples were run in triplicate. The comparative Ct method (ΔΔCt) was used to calculate relative expression of the transcript of interest and a positive control. The change in threshold cycle (ΔCt) of each sample was calculated as the Ct value of the tested gene (target) minus the Ct value of GAPDH (endogenous control). The ΔΔCt of each sample was obtained by subtracting the ΔCt value of the reference from the ΔCt value of the sample. The ΔCt reference value used was the ΔCt obtained from sorted BM pro-B cells of WT mice housed in the CF. The fold change in each group, calculated as 2^–ΔΔCt^, was compared. Differences among the 3 groups were assessed using 1-way ANOVA. Post hoc multiple comparisons were performed using Dunnett’s test to compare each experimental group with the control group (WT pro-B cells), with multiplicity-adjusted *P* values reported.

### Statistics.

Sample sizes were determined based on the literature describing mouse modeling of natural infection–driven leukemia (12–14, 46) and were justified by power calculations estimating 90% power to detect differential leukemia incidence. Statistical analyses were performed using Prism v8.2.1 (GraphPad Software). Differences in Kaplan-Meier survival plots of transgenic and WT mice were analyzed using the log-rank (Mantel-Cox) test. The level of significance was set at a *P* value of less than 0.05. In the figures, each dot represents an individual sample and the bars indicate the mean ± SEM, as indicated in the figure legend.

### Study approval.

All animal work has been conducted according to relevant national and international guidelines and it has been approved by the Bioethics Committee of University of Salamanca and by the Bioethics Subcommittee of Consejo Superior de Investigaciones Científicas (CSIC).

### Data availability.

Authors confirm that all relevant data are included in the paper and/or its supplemental material files. All data reported in this article are deposited in the NCBI’s Sequence Read Archive (SRA) and Gene Expression Omnibus (GEO) (47), under the umbrella BioProject accession number PRJNA1291066. All values underlying the figures, including individual data points and values underlying reported means, are provided in the Supporting Data Values file with separate, clearly labeled sheets for each figure panel, as well as in Supplemental Tables 1–5.

## Author contributions

Initial conception of the project was by BRC, ACG, NO, CVD, KEN, and ISG. Development of methodology was performed by BRC, ACG, NO, PSC, ALAN, JMC, APP, EGS, OB, DAL, JR, SR, PPM, FJGC, MBGC, AO, MRO, CC, CVD, KEN, and ISG. OB, MBGC, FJGC, and CVD performed pathology review. BRC, ACG, NO, PSC, ALAN, JMC, APP, EGS, OB, DAL, JR, SR, PPM, FJGC, MBGC, AO, MRO, CC, CVD, KEN, and ISG were responsible for analysis and interpretation of the data (e.g., statistical analysis, biostatistics, computational analysis). Manuscript preparation was performed by BRC, ACG, NO, PSC, ALAN, JMC, APP, EGS, OB, DAL, JR, SR, PPM, FJGC, MBGC, AO, MRO, CC, CVD, KEN, and ISG. Administrative, technical, or material support (i.e., reporting or organizing the data, constructing databases) was compiled by BRC, ACG, NO, PSC, ALAN, DAL, CVD, KEN, and ISG. The study was supervised by CVD, KEN, and ISG. Conceptualization and execution of this study were distributed equally among 3 lead investigators whose distinct expertise was essential for its completion. BRC designed and spearheaded the bulk of the experimental laboratory work. ACG was responsible for the core theoretical design and framework, and NO performed the whole-genome sequencing, primary statistical and bioinformatic analyses. Given that the study’s success relied entirely on the integration of these 3 fundamental pillars, we consider that these authors contributed equally to this work and should be recognized as co–first authors.

## Funding support

This work is funded partially by the NIH, and is subject to the NIH Public Access Policy. Through acceptance of this federal funding, the NIH has been given a right to make the work publicly available in PubMed Central.

- MICIU/AEI/ 10.13039/501100011033 and ERDF/EU grants PID2021-122185OB-I00 and PID2024-155590OB-I00 (to ISG).
- Junta de Castilla y León grants UIC-017, CSI144P20, and CSI016P23 (to ISG).
- Fundacion Unoentrecienmil CUNINA2 project (to ISG).
- Fundación Científica de la Asociación Española contra el Cáncer (TRNSC247893SÁNC) (to ISG).
- Instituto de Salud Carlos III (ISCIII) (AC24/00021) (to ISG).
- Fundación Científica de la Asociación Española contra el Cáncer grant PRYCO211305SANC (to CC, MR, and ISG).
- “La Caixa” Foundation under the project code LCF/PR/HR25/52450048 (to CC, MR, and ISG).
- TRANSCAN2023-1858-066 – REACTION Project (to MR and ISG). Research at C. Cobaleda’s laboratory was partially supported by Grant PID2021-122787OB-I00 funded by
- MICIU/AEI/ 10.13039/501100011033 and ERDF/EU grant PID2021-122787OB-I00 (to CC).
- “Fundación Síndrome de Wolf-Hirschhorn o 4p-” research contract (to CC).
- “Fundación Ramón Areces” and “Banco de Santander” institutional grants to the Centro de Biología Molecular.
- Instituto de Salud Carlos III (ISCIII) project PI22/00379 (to CVD).
- American Lebanese Syrian Associated Charities (ALSAC) (to KEN).
- National Cancer Institute/NIH grant R01CA241452 (to KEN).
- National Institute of Allergy and Infectious Diseases Histiocytosis Association Cures within Reach grant R21AI113490 (to KEN).
- Asociación Pablo Ugarte (to MR).
- FSE-Conserjería de Educación de la Junta de Castilla y León 2019 (ESF, European Social Fund) fellowship CSI067-18 (to ACG).
- Juan de la Cierva 2022 grant JDC2022-049078-I (to ACG).
- FSE-Conserjería de Educación de la Junta de Castilla y León 2022 (ESF, European Social Fund) fellowship CSI002-22 (to BRC).
- Ayuda para Contratos predoctorales para la formación de doctores (PRE2022-103628) (to ALAN).
- Universidad Autónoma de Madrid predoctoral fellowship FPI-UAM 2019 (to JMC).

## Supplementary Material

Supplemental data

Supplemental data set 1

Unedited blot and gel images

Supplemental table 1

Supplemental table 2

Supplemental table 3

Supplemental table 4

Supplemental table 5

Supporting data values

## Figures and Tables

**Table 1:**
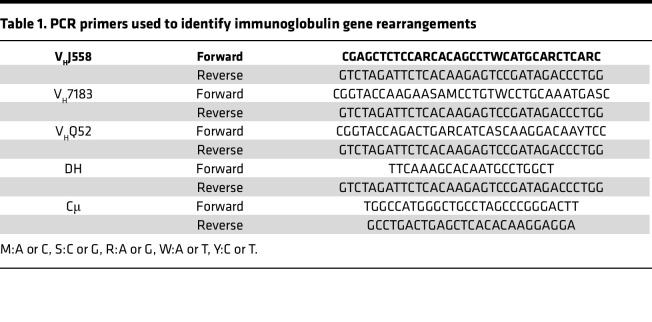
PCR primers used to identify immunoglobulin gene rearrangements

## Version 1 02/17/2026

In-Press Preview

## Version 2 04/08/2026

Electronic publication
