# Supplementary material for: Deletion of 9p drives B-ALL through heterozygous inactivation of Pax5 and Cd72 in preleukemic cells
Source: JCI Insight. 2026 Feb 17;11(7):e199464. doi: 10.1172/jci.insight.199464 (PMC13134721; doi:10.1172/jci.insight.199464)
Supplement: Supplemental data [file jciinsight-11-199464-s203.pdf]

Supplemental material

Deletion 9p drives B-ALL through the heterozygous inactivation of Pax5 and Cd72 in pre-leukemic cells

Belén Ruiz-Corzo[1,2*], Ana Casado-García[1,2*], Ninad Oak[3*], Paula Somoza-Cotillas[1,2], Andrea López-Álvarez de Neyra[1,2], Jorge Martínez-Cano[4], Alba Pérez-Pons[5,6], Elena G. Sánchez[7], Oscar Blanco[2,8], Diego Alonso-López[9], Javier De Las Rivas [2,10], Susana Riesco [2,11], Pablo Prieto-Matos[2,11], Francisco Javier García Criado[2,12], María Begoña García Cenador[2,12], Alberto Orfao[5,6], Manuel Ramírez-Orellana[13], César Cobaleda[4], Carolina Vicente-Dueñas[2,11#], Kim E Nichols[3#], and Isidro Sánchez-García[1,2#]

(, #) Should be considered equal first authors and senior authors, respectively.*

[1] Experimental Therapeutics and Translational Oncology Program, Instituto de Biología Molecular y Celular del Cáncer, CSIC-USAL, Campus M. de Unamuno s/n, Salamanca, Spain;
[2]Institute of Biomedical Research of Salamanca (IBSAL), Universidad de Salamanca, Campus Miguel de Unamuno s/n, 37007 Salamanca, Spain.
[3] Department of Oncology, St. Jude Children's Research Hospital, Memphis, TN, United States.
[4] Immune system development and function Unit, Centro de Biología Molecular Severo Ochoa (Consejo Superior de Investigaciones Científicas -Universidad Autónoma de Madrid), Madrid, Spain.
[5] Cancer Research Center (IBMCC, USAL-CSIC), Department of Medicine and Cytometry Service (NUCLEUS), University of Salamanca (https://ror.org/02f40zc51), Salamanca, Spain; and Biomedical Research Institute of Salamanca (IBSAL), Salamanca, Spain.
[6] Biomedical Research Networking Center Consortium (CIBERONC; CB16/12/00400), Madrid, Spain.
[7]Oncohematology Laboratory. Advance Terapy Unit. Fundación para la Investigación Biomédica del Hospital Universitario Niño Jesús (FIB HUNJ), Madrid, Spain.
[8] Departamento de Anatomía Patológica, Universidad de Salamanca, Salamanca, Spain.
[9]Bioinformatics Unit, Cancer Research Center (CSIC-USAL), Salamanca, Spain.
[10] Bioinformatics and Functional Genomics Research Group, Cancer Research Center (CSIC-USAL), Salamanca, Spain.
[11] Department of Pediatrics, Hospital Universitario de Salamanca, Paseo de San Vicente, 58-182, Salamanca 37007, Spain.
[12] Departamento de Cirugía, Universidad de Salamanca, Salamanca, Spain.
[13]Department of Pediatric Hematology and Oncology, Hospital Infantil Universitario Niño Jesús Jesús & Institute of Biomedical Research Hospital Universitario La Princesa, Madrid, Spain.

Supplemental data

Supplemental Tables

Supplemental Table 1: Differentially expressed genes in preleukemic *CD72*$^{+/-}$*; Pax5*$^{+/-}$ versus preleukemic *Pax5*$^{+/-}$ proB cells. 312 genes-probesets.

Supplemental Table 2: Differentially expressed genes in preleukemic *CD72*$^{+/-}$*; Pax5*$^{+/-}$ versus WT proB cells. 255 genes-probesets.

Supplemental Table 3: Differentially expressed genes in preleukemic *Pax5*$^{+/-}$ versus WT proB cells. 246 genes-probesets.

Supplemental Table 4. Genetic Background of the mice using the miniMUGA array

Supplemental Table 5. Cytokine serum levels of IL-6, IL-2, IL-4, IL-10, IL-17A, TNFα and IFNγ in *Cd72*$^{+/-}$;*Pax5*$^{+/-}$ *and Cd72*$^{+/-}$ mice over time (3, 5, 7, 9, 11 and 13 months of age).

Supplemental zip file.

Supplemental Strain_Genotyping.zip. Details of the strain genotyping results diagnosed by SNPs, using the MiniMUGA Background Analysis v2.3.1, for the mice listed in Supplemental Table 4.

Supplemental Figures

Supplemental Figure 1

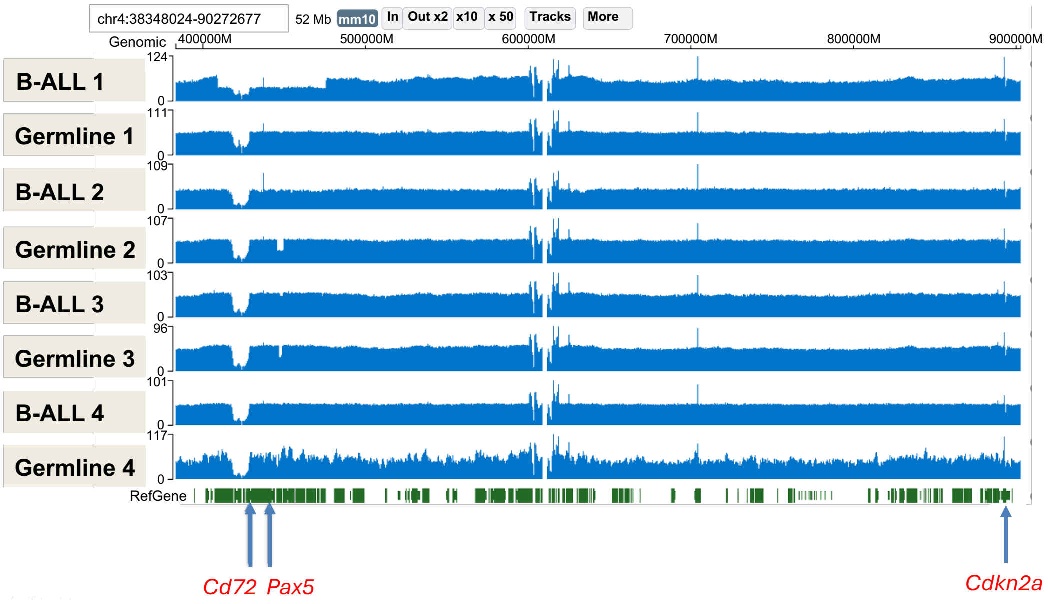

Supplemental Figure 1: Representative chromosomal 4 alterations affecting the *Pax5* gene in murine B-ALL showing DNA deletion encompassing the *Pax5* and *Cd72* loci. Genome-wide copy number data are shown for B-cell acute lymphoblastic leukemia (B-ALL) samples (top track) compared to matched germline DNA (bottom track), visualized using the UCSC Genome Browser (mm10 assembly). The genomic coordinates span chr4: 38348024-90272677 (52 Mb). The blue read-depth signal represents normalized copy number, with relative gains and losses detectable by comparison between tracks. Focal deletion is observed in the B-ALL sample around the *Pax5* locus. Additional deletions affecting *Cd72* and other genes are also annotated. These somatic copy number losses suggest a pathogenic role in leukemogenesis.

Supplemental Figure 2

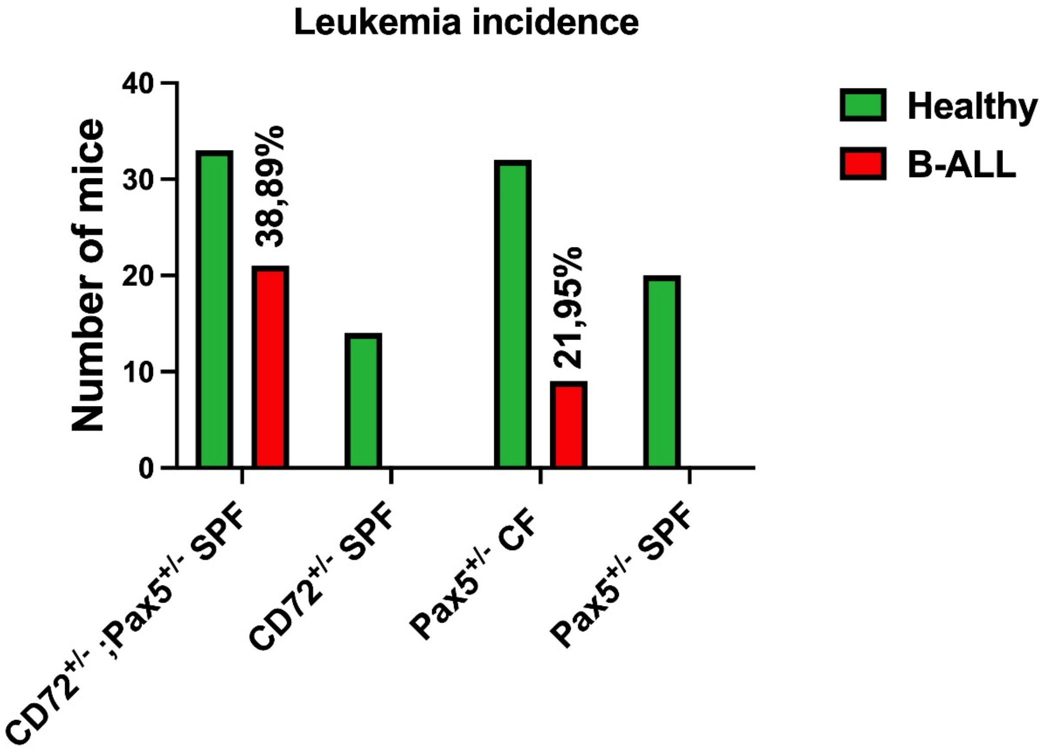

Supplemental Figure 2: *CD72*$^{+/-}$*; Pax5*$^{+/-}$ mice housed in SPF conditions developed leukemia. *CD72*$^{+/-}$*; Pax5*$^{+/-}$ mice (n= 54) that were not exposed to infections, develop B-ALL with an incidence of 38,89%, which is slightly higher than that of *Pax5*$^{+/-}$ mice (n= 41) when exposed to infection in a conventional facility (CF) (21,95%). None of the *CD72*$^{+/-}$ (n= 14) or *Pax5*$^{+/-}$ (n= 15) mice housed in SPF conditions developed the disease. (SPF: specific pathogen free; CF: conventional facility).

Supplemental Figure 3

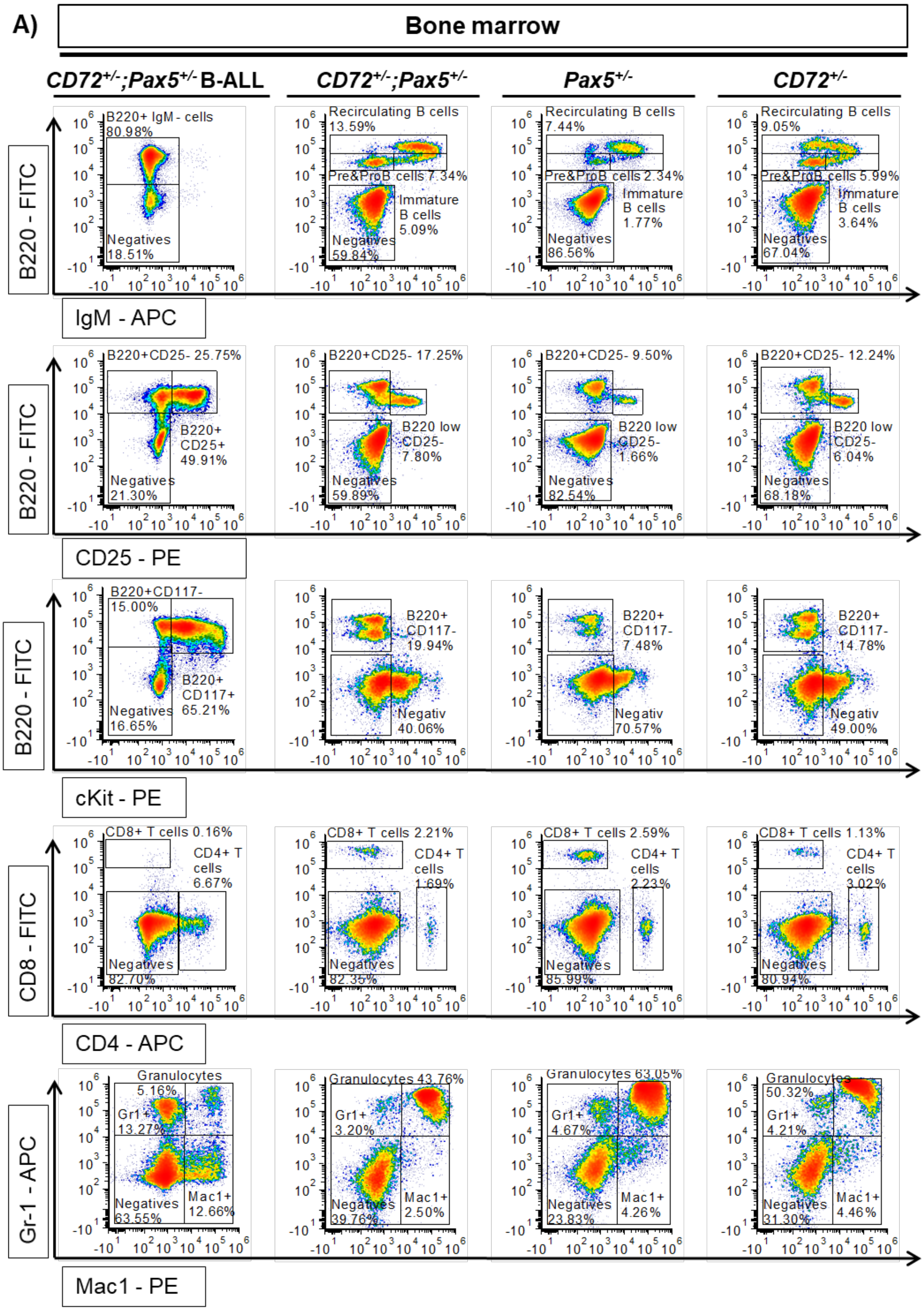

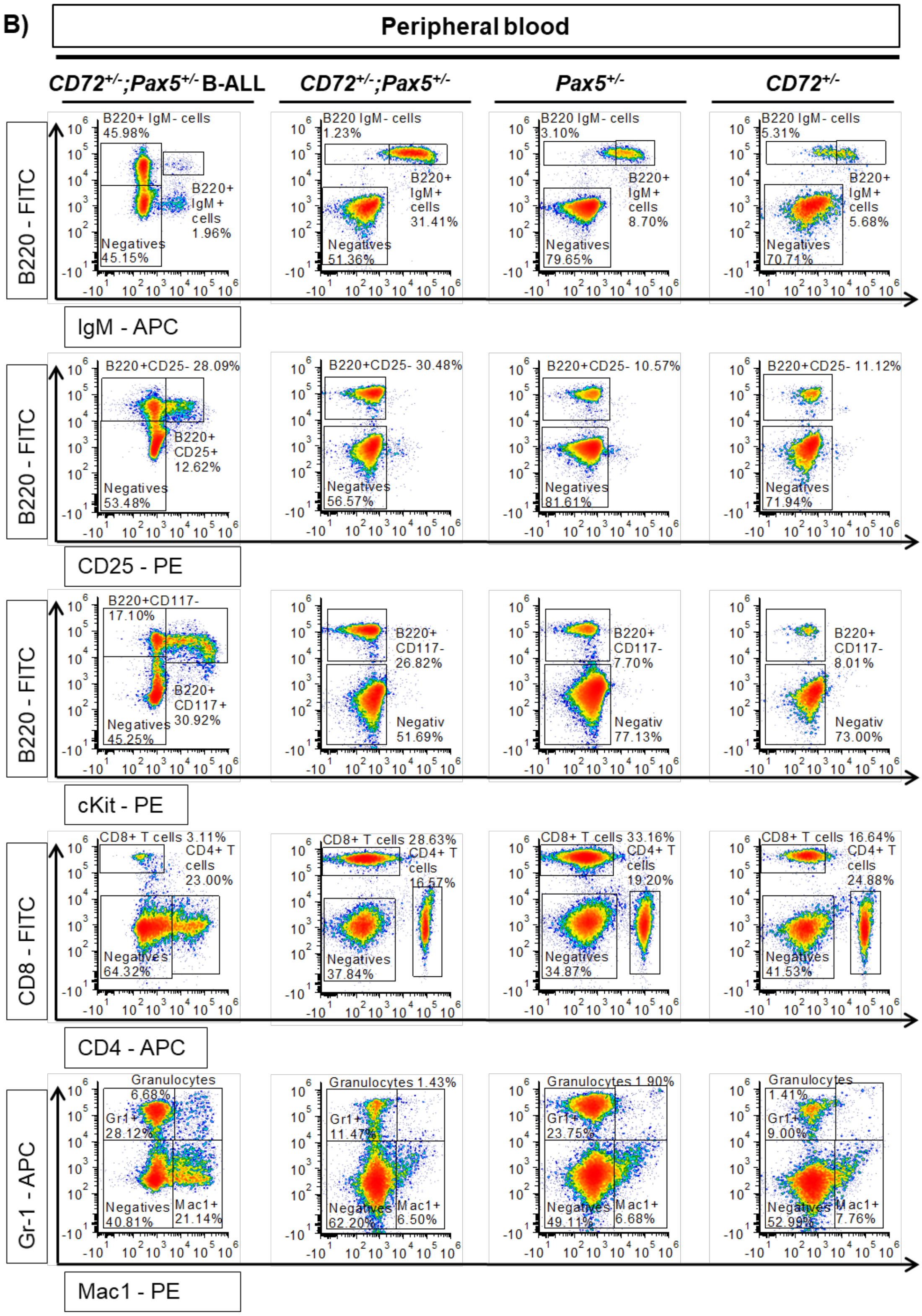
B)
Peripheral blood
CD72+/-;Pax5+/- B-ALL
CD72+/-;Pax5+/-
Pax5+/-
CD72+/-
B220 - FITC
IgM - APC
B220+ IgM- cells 45.98%
B220+ IgM+ cells 1.96%
Negatives 45.15%
B220 IgM- cells 1.23%
B220+ IgM+ cells 31.41%
Negatives 51.36%
B220 IgM- cells 3.10%
B220+ IgM+ cells 8.70%
Negatives 79.65%
B220 IgM- cells 5.31%
B220+ IgM+ cells 5.68%
Negatives 70.71%
CD25 - PE
B220+CD25- 28.09%
B220+ CD25+ 12.62%
Negatives 53.48%
B220+CD25- 30.48%
Negatives 56.57%
B220+CD25- 10.57%
Negatives 81.61%
B220+CD25- 11.12%
Negatives 71.94%
cKit - PE
B220+CD117- 17.10%
B220+ CD117+ 30.92%
Negatives 45.25%
B220+ CD117- 26.82%
Negativ 51.69%
B220+ CD117- 7.70%
Negativ 77.13%
B220+ CD117- 8.01%
Negativ 73.00%
CD8 - FITC
CD4 - APC
CD8+ T cells 3.11%
CD4+ T cells 23.00%
Negatives 64.32%
CD8+ T cells 28.63%
CD4+ T cells 16.57%
Negatives 37.84%
CD8+ T cells 33.16%
CD4+ T cells 19.20%
Negatives 34.87%
CD8+ T cells 16.64%
CD4+ T cells 24.88%
Negatives 41.53%
Gr-1 - APC
Mac1 - PE
Granulocytes 6.68%
Gr1+ 28.12%
Negatives 40.81%
Mac1+ 21.14%
Granulocytes 1.43%
Gr1+ 11.47%
Negatives 62.20%
Mac1+ 6.50%
Granulocytes 1.90%
Gr1+ 23.75%
Negatives 49.11%
Mac1+ 6.68%
Granulocytes 1.41%
Gr1+ 9.00%
Negatives 52.99%
Mac1+ 7.76%

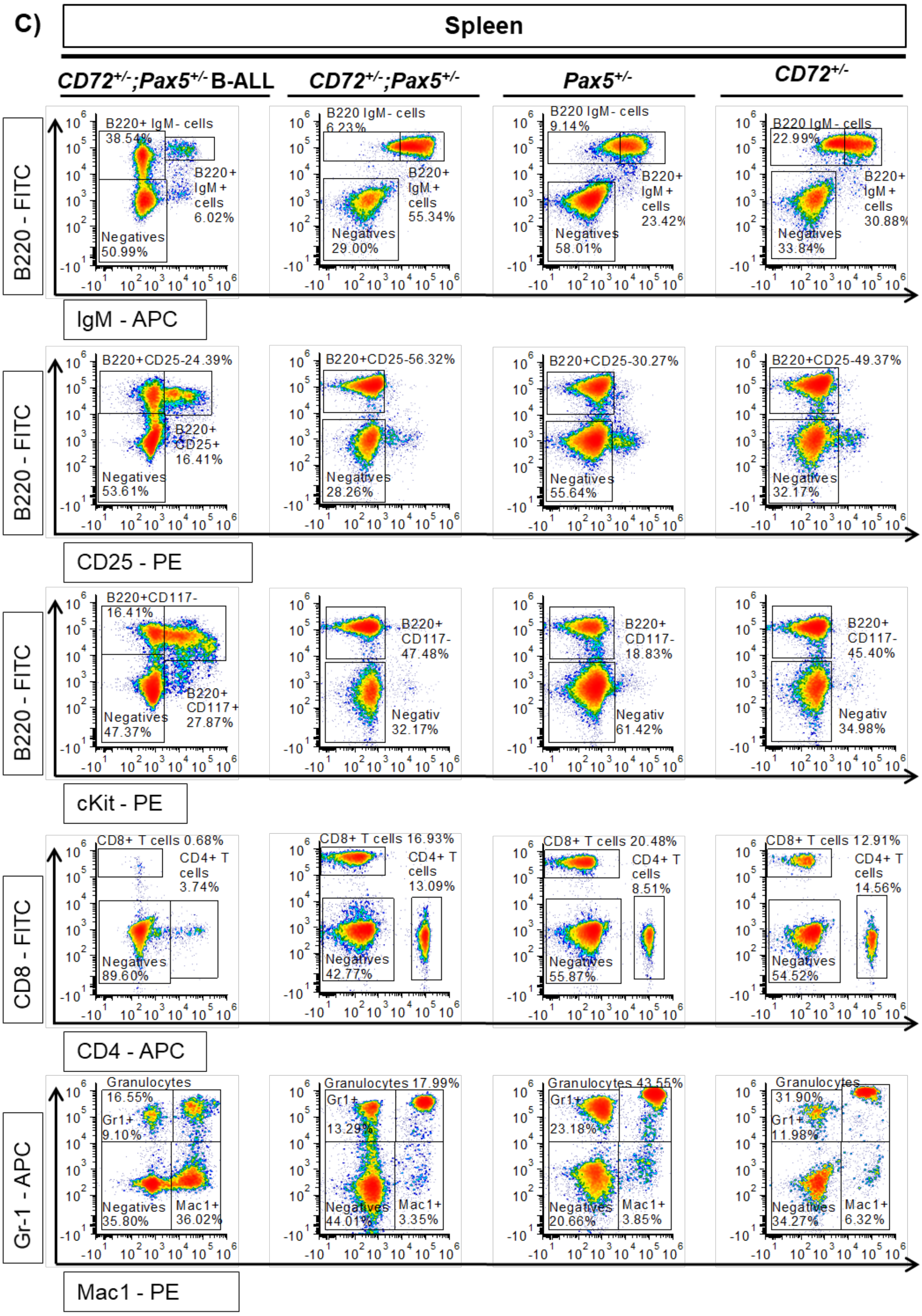
C)
Spleen
CD72+/-;Pax5+/- B-ALL
CD72+/-;Pax5+/-
Pax5+/-
CD72+/-
B220 - FITC
IgM - APC
B220+ IgM- cells 38.54%
B220+ IgM+ cells 6.02%
Negatives 50.99%
B220 IgM- cells 6.23%
B220+ IgM+ cells 55.34%
Negatives 29.00%
B220 IgM- cells 9.14%
B220+ IgM+ cells 23.42%
Negatives 58.01%
B220 IgM- cells 22.99%
B220+ IgM+ cells 30.88%
Negatives 33.84%
CD25 - PE
B220+CD25-24.39%
B220+ CD25+ 16.41%
Negatives 53.61%
B220+CD25-56.32%
Negatives 28.26%
B220+CD25-30.27%
Negatives 55.64%
B220+CD25-49.37%
Negatives 32.17%
cKit - PE
B220+CD117- 16.41%
B220+ CD117+ 27.87%
Negatives 47.37%
B220+ CD117- 47.48%
Negativ 32.17%
B220+ CD117- 18.83%
Negativ 61.42%
B220+ CD117- 45.40%
Negativ 34.98%
CD8 - FITC
CD4 - APC
CD8+ T cells 0.68%
CD4+ T cells 3.74%
Negatives 89.60%
CD8+ T cells 16.93%
CD4+ T cells 13.09%
Negatives 42.77%
CD8+ T cells 20.48%
CD4+ T cells 8.51%
Negatives 55.87%
CD8+ T cells 12.91%
CD4+ T cells 14.56%
Negatives 54.52%
Gr-1 - APC
Mac1 - PE
Granulocytes 16.55%
Gr1+ 9.10%
Negatives 35.80%
Mac1+ 36.02%
Granulocytes 17.99%
Gr1+ 13.29%
Negatives 44.01%
Mac1+ 3.35%
Granulocytes 43.55%
Gr1+ 23.18%
Negatives 20.66%
Mac1+ 3.85%
Granulocytes 31.90%
Gr1+ 11.98%
Negatives 34.27%
Mac1+ 6.32%

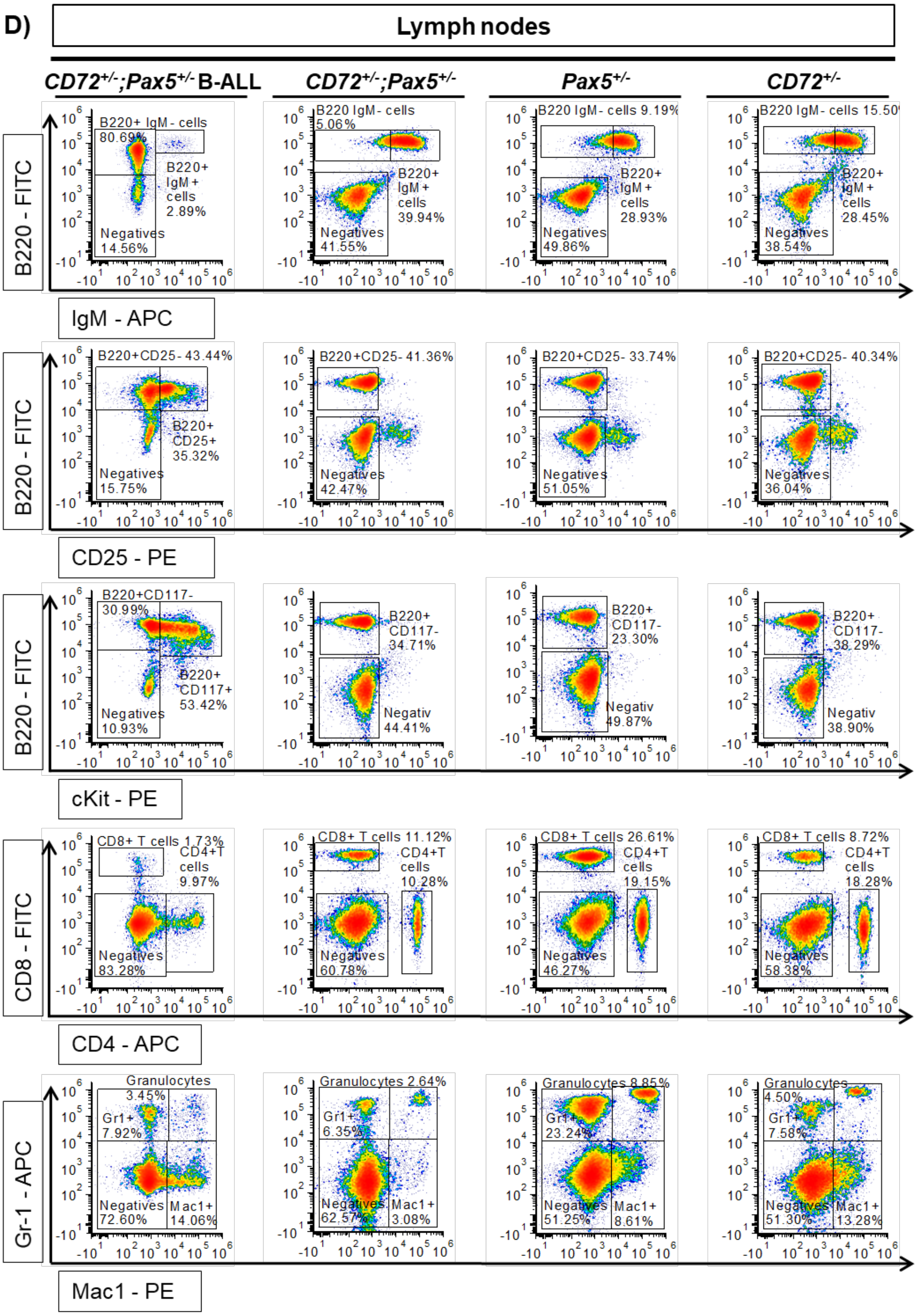

Supplemental Figure 3: B-ALL in *CD72*$^{+/-}$*; Pax5*$^{+/-}$ mice. Flow cytometric analysis of hematopoietic subsets in diseased *CD72*$^{+/-}$*; Pax5*$^{+/-}$ mice. Representative plots of cell subsets from the bone marrow (**A**), peripheral blood (**B**), spleen (**C**), and lymph nodes (**D**) show accumulation of blast B cells in a leukemic *CD72*$^{+/-}$*; Pax5*$^{+/-}$ mouse (B834) compared to age-matched healthy littermates *CD72*$^{+/-}$*; Pax5*$^{+/-}$ (B855), *Pax5*$^{+/-}$ (S907) and *CD72*$^{+/-}$ (B924) mice.

Supplemental Figure 4

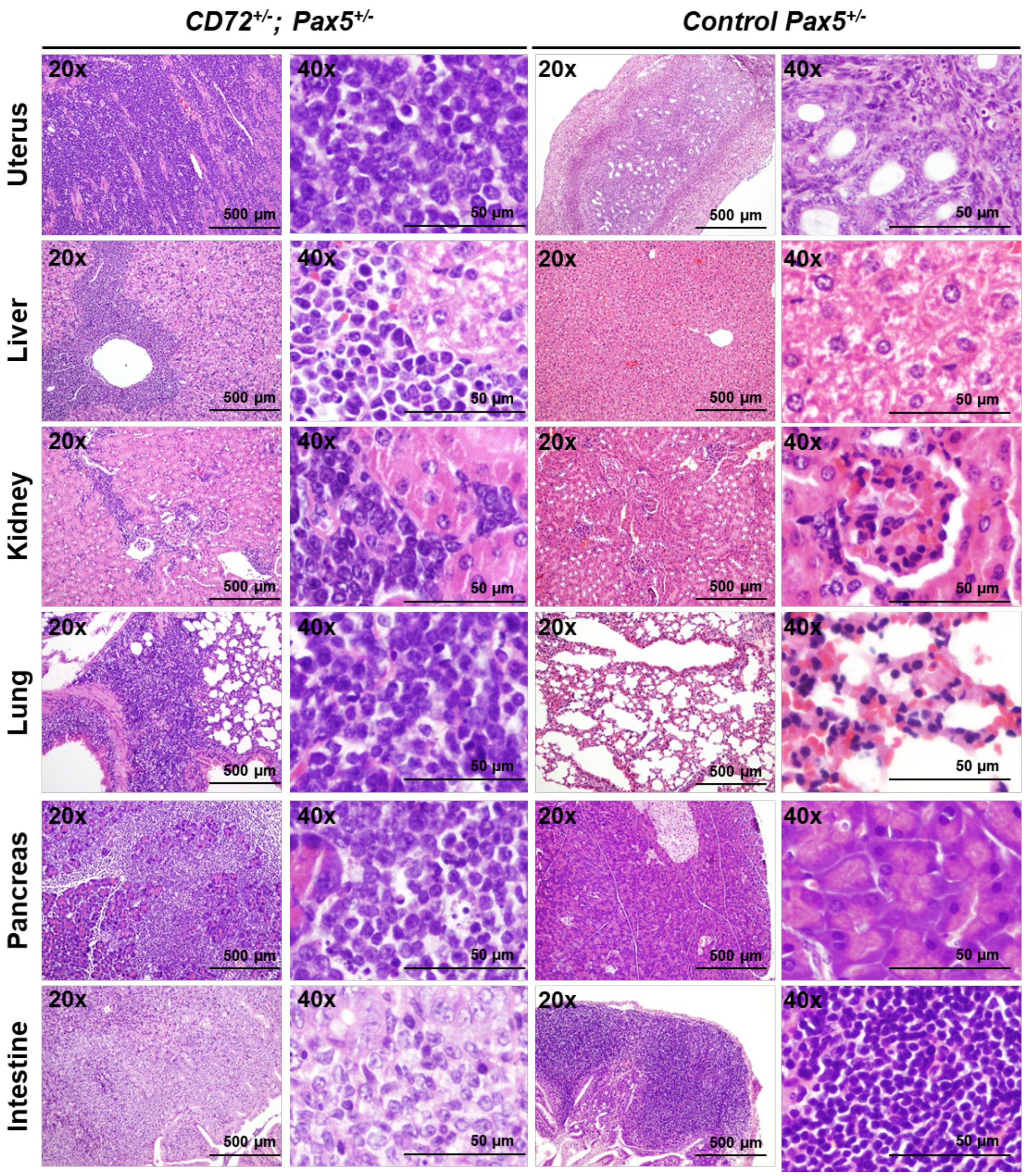

Supplemental Figure 4: B-ALL in a *CD72*$^{+/-}$*;Pax5*$^{+/-}$ mouse. Haematoxylin and eosin staining of tumour-bearing *CD72*$^{+/-}$*; Pax5*$^{+/-}$ mice (A072) housed in a specific pathogen-free facility (SPF), reveals infiltrating blast cells in the uterus, liver, kidney, lung, pancreas and intestine. Loss of normal tissue architecture is observed with cells morphologically resembling lymphoblasts (n=1). Tissues from a control *Pax5*$^{+/-}$ littermate mouse are included for reference. Magnification and corresponding scale bars are indicated in each case.

Supplemental Figure 5

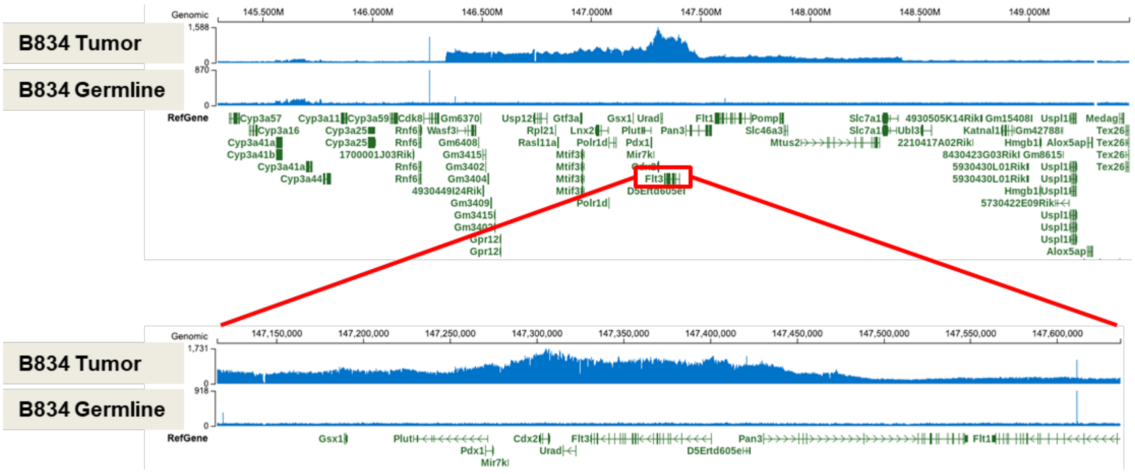

Supplemental Figure 5: Focal somatic amplification of the *Flt3* locus in a murine B-ALL tumor from a *Cd72*$^{+/-}$;*Pax5*$^{+/-}$ mouse. Copy number profiles of a B-ALL tumor (B834) and matched germline DNA (tail) are shown across chr5:145,000,000–149,000,000 (mm10 assembly), visualized using the UCSC Genome Browser. Read-depth signals (blue) reveal a focal amplification at the *Flt3* locus (highlighted in red), absent in the germline sample. *Flt3* encodes a receptor tyrosine kinase critical for hematopoietic stem and progenitor cell survival and proliferation.

Supplemental Figure 6

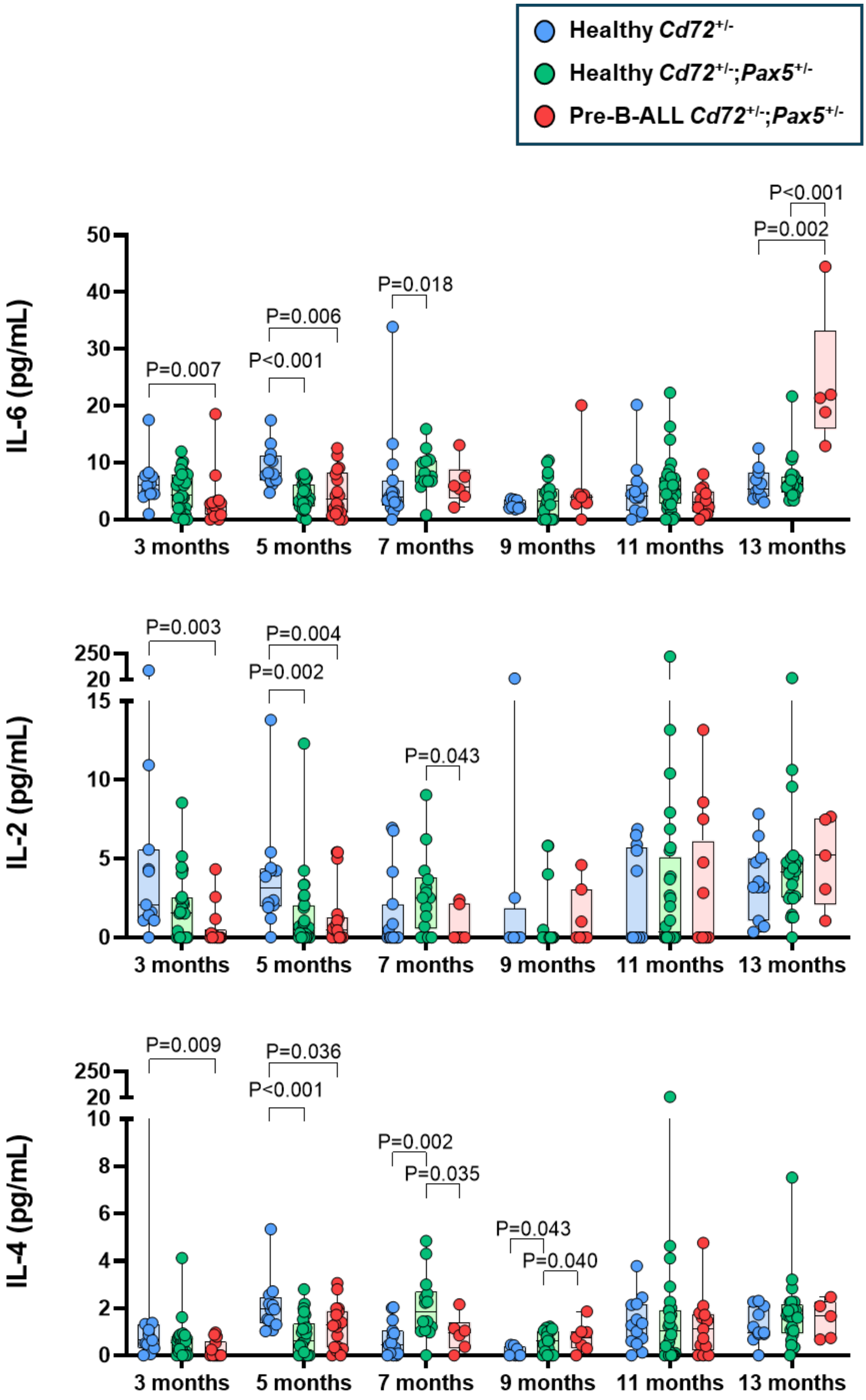

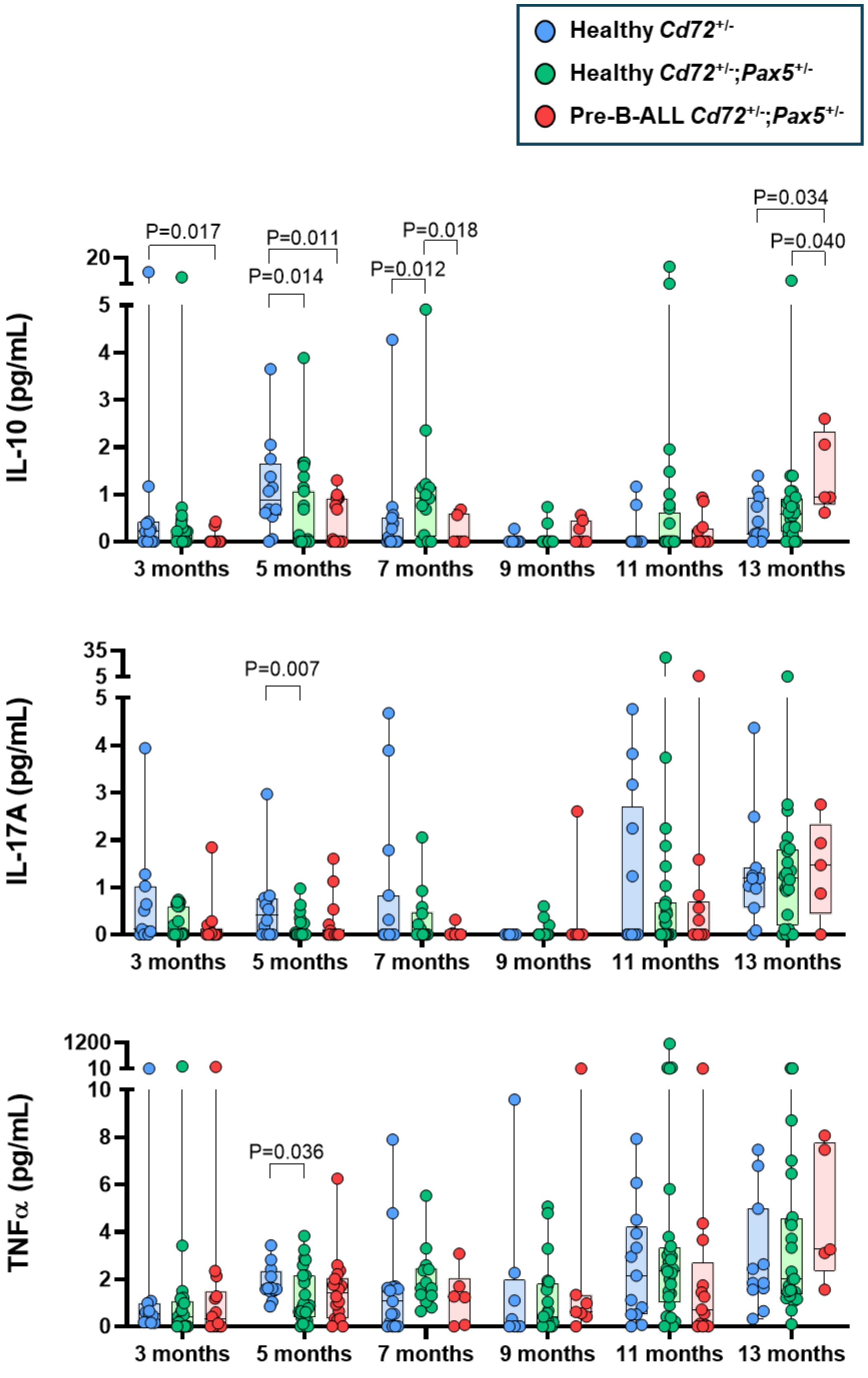
Healthy Cd72+/-
Healthy Cd72+/-;Pax5+/-
Pre-B-ALL Cd72+/-;Pax5+/-
IL-10 (pg/mL)
P=0.017
P=0.011
P=0.014
P=0.018
P=0.012
P=0.034
P=0.040
3 months
5 months
7 months
9 months
11 months
13 months
IL-17A (pg/mL)
P=0.007
3 months
5 months
7 months
9 months
11 months
13 months
TNFα (pg/mL)
P=0.036
3 months
5 months
7 months
9 months
11 months
13 months

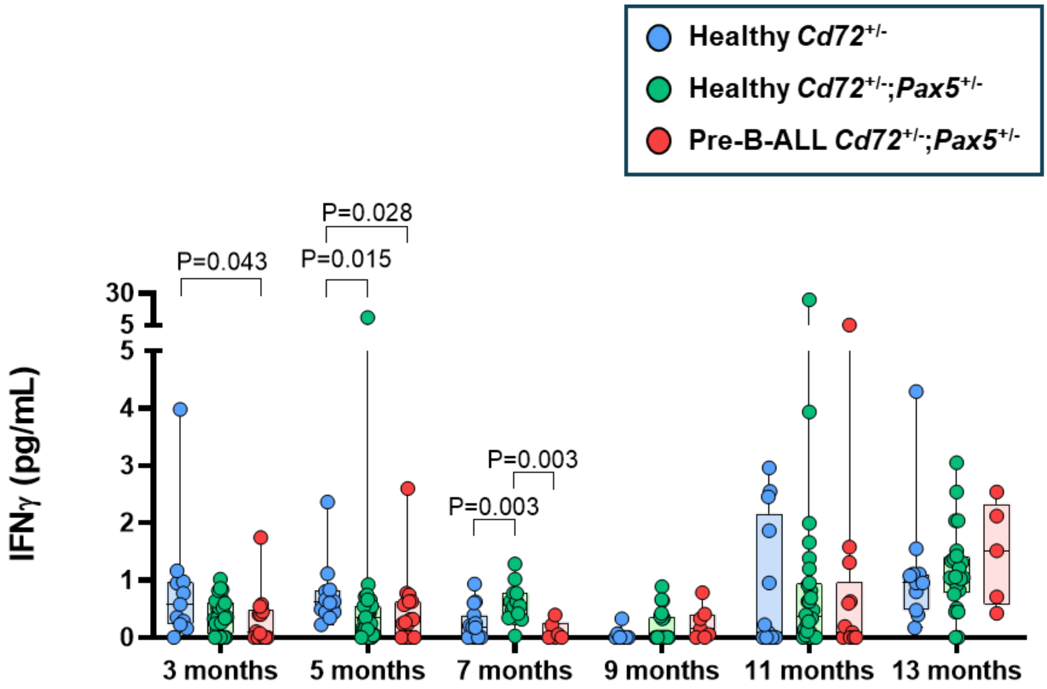

Supplemental Figure 6: Cytokine serum levels in *Cd72*$^{+/-}$;*Pax5*$^{+/-}$mice over time. Serum concentrations of IL-6, IL-2, IL-4, IL-10, IL-17A, TNFα and IFNγ were quantified in *Cd72*$^{+/-}$;*Pax5*$^{+/-}$ mice that developed B-ALL (red) and compared with healthy *Cd72*$^{+/-}$;*Pax5*$^{+/-}$ (green) and healthy *Cd72*$^{+/-}$ (blue) littermates at 3, 5, 7, 9, 11 and 13 months of age using a Cytometric Bead Array immunoassay (CBA, BD Biosciences). All animals were housed under specific-pathogen-free (SPF) conditions, as detailed in the Methods section. Each dot represents an individual mouse and box-and-whisker plots indicate median, interquartile range and range. P values above brackets indicate statistically significant differences between the indicated groups at each time point (U de Mann-Whitney test for each time point).

Supplemental Figure 7

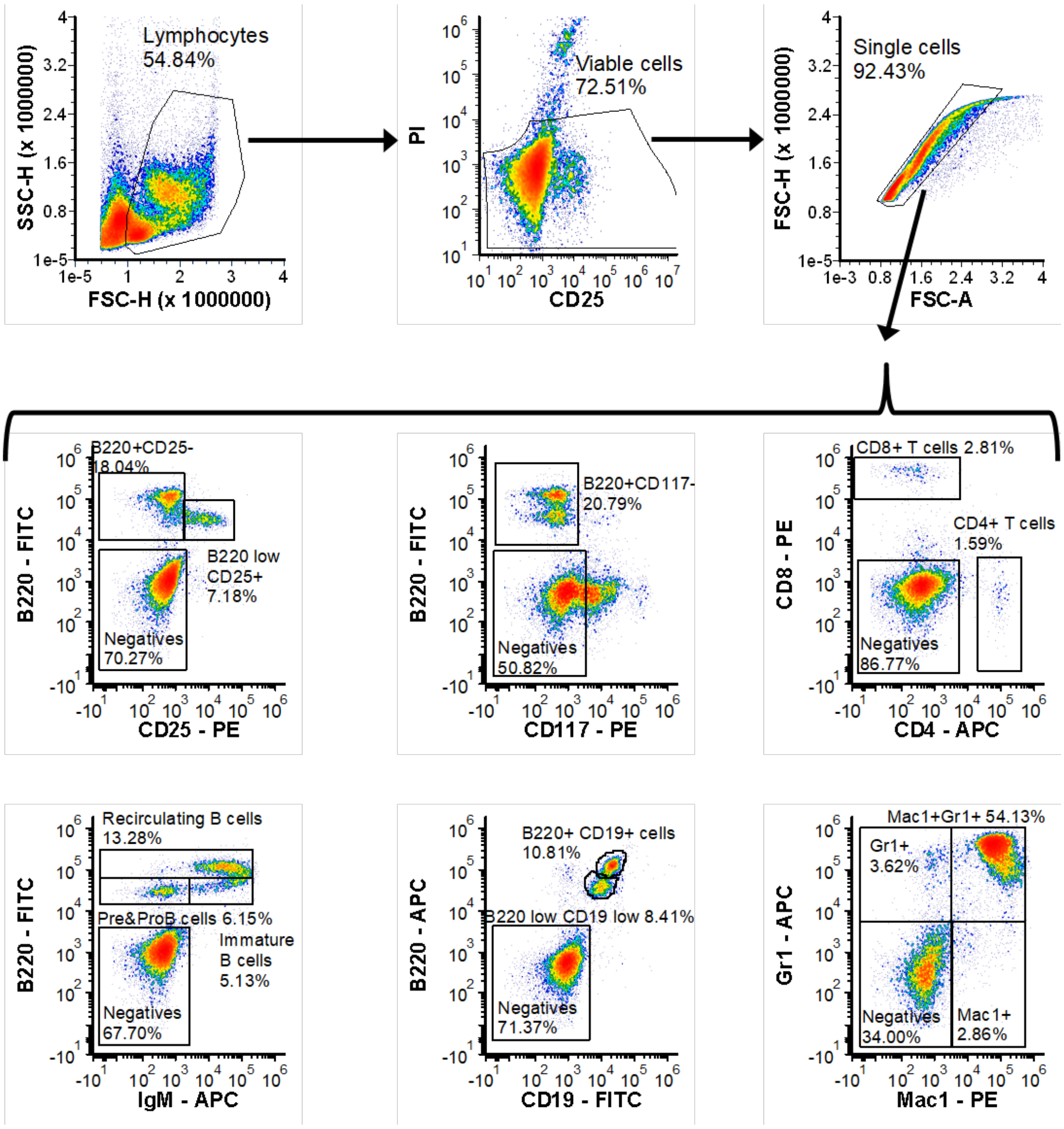

Supplemental Figure 7: Gating strategy used in FACs analysis. This figure exemplifies the gating strategy used in all cytometric analyses. For each analysis, a total of at least 100.000 viable cells (PI-; propidium iodide negative cells) were assessed. Singlets were selected prior gating strategy that is specific for each population. Bone marrow cells stained with the indicated antibodies are shown as an example. The same gating strategy was used for all FACs analysis presented in Figure 1C and Supplemental Figure 3.
