# Supplementary material for: Deletion of 9p drives B-ALL through heterozygous inactivation of Pax5 and Cd72 in preleukemic cells
Source: JCI Insight. 2026 Feb 17;11(7):e199464. doi: 10.1172/jci.insight.199464 (PMC13134721; doi:10.1172/jci.insight.199464)
Supplement: Supplemental data set 1 [file jciinsight-11-199464-s204.zip › Strain_Genotyping/A072-results-report.pdf]

MiniMUGA Background Analysis v2.3.1

**Sample ID**	A072
**Neogen ID**	AAAU-4505
**Summary**	The genotype of this sample is of **excellent** quality. It is **female** and **outbred**, and likely a mix of **C57BL/6J and C57BL/6NRj** and **CBA/J**. Clustering of unexplained markers is evidence of an additional background strain.<br><br>Diagnostic SNPs are likely explained by the presence of the background strains<br>• Solution 1: 129S5/SvEvBrd and C57BL/6J and C57BL/6NRj<br>◦ C57BL/6J: 70 / 163 (42.9%)<br>◦ C57BL/6NRj: 28 / 40 (70.0%)<br>◦ 129S5/SvEvBrd: 1 / 5 (20.0%)<br>• Solution 2: 129S5/SvEvBrd and C57BL/6JRj and C57BL/6NRj<br>◦ C57BL/6JRj: 70 / 163 (42.9%)<br>◦ C57BL/6NRj: 28 / 40 (70.0%)<br>◦ 129S5/SvEvBrd: 1 / 5 (20.0%)<br><br>NOTE: There is a discrepancy between the diagnostic backgrounds detected and the primary and secondary background analysis (C57BL/6NRj, CBA/J, C57BL/6J). This is uncommon and should be investigated further.<br><br>No genetic constructs were detected in this sample.<br><br>WARNING:<br>• There is a discrepancy between the diagnostic backgrounds detected ((129S5/SvEvBrd and C57BL/6J and C57BL/6NRj) or (129S5/SvEvBrd and C57BL/6JRj and C57BL/6NRj)) and the primary background (C57BL/6J and C57BL/6NRj) and secondary background (CBA/J). This is uncommon and should be investigated further.<br>• The presence of a single diagnostic heterozygous call for a single inbred strain should be treated with caution.<br>• This sample likely has more than 2 genetic backgrounds (unexplained regions and/or fractured ideogram). The strain selected for secondary background may be incorrect. The estimation of the contribution of primary and secondary background are likely incorrect. This can potentially be addressed with input from the user.
**Genotyping Quality**	**Excellent (17 N calls)**<br>All reported results are dependent on genotyping quality.
**Chromosomal Sex**	XX
**Inbreeding Estimate**	39.9% Inbred<br>(Percentage of the genome (autosomal and X chromosomes) that is homozygous or hemizygous for primary, secondary, and unknown backgrounds. See Genome Analysis)

Constructs Detected

BlastR	bpA	Cas9	chlor	cHS4	Cre	DTA	Flp	g_FP	hCMV_a	hCMV_b	hTK_pr	iCre	IRES	Luc	r_FP	rtTA	SV40	tTA
-	-	-	-	-	-	-	-	-	-	-	-	-	-	-	-	-	-	-

MiniMUGA Background Analysis v2.3.1

Refined Ideogram

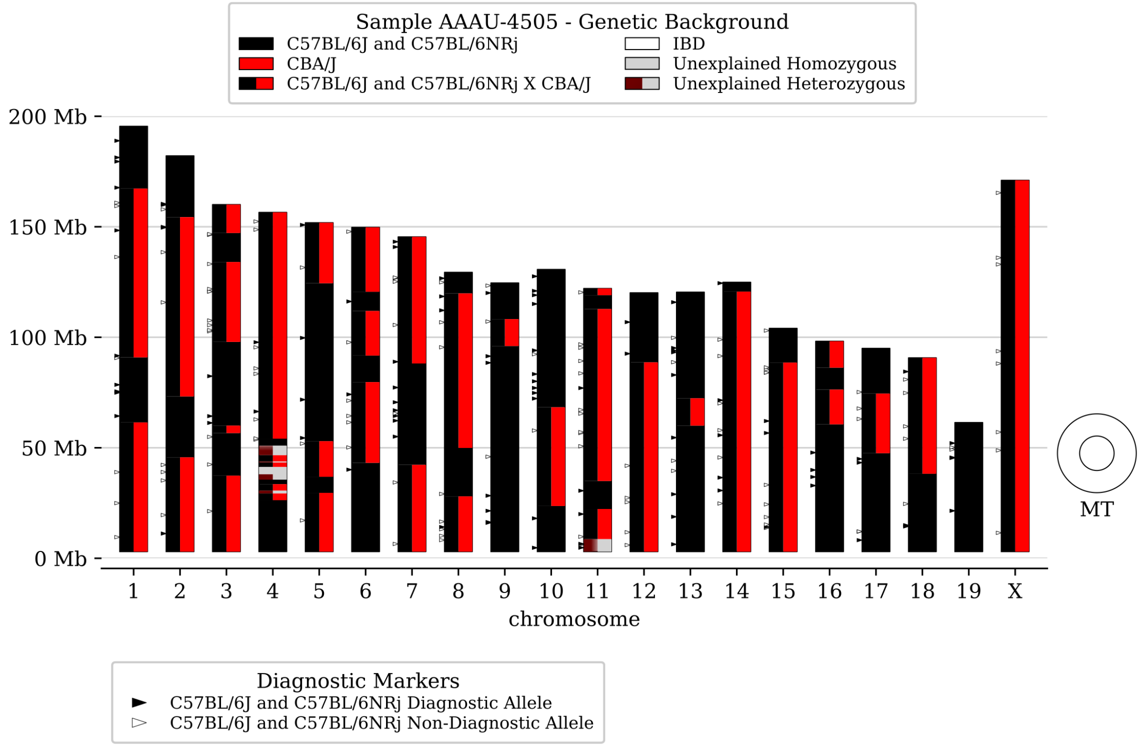

Genome Analysis

Background	Zygosity	Informative Markers	Informative Markers %	Genome %
C57BL/6J and C57BL/6NRj	N/A	1181	44.6%	39.7%
CBA/J	Homozygous	1	0.0%	0.0%
C57BL/6J and C57BL/6NRj X CBA/J	Heterozygous	1440	54.4%	59.6%
Unexplained	Homozygous	5	0.2%	0.1%
Unexplained	Heterozygous	21	0.8%	0.5%
	**Total**	2648	100.0%	99.9%

Y Chromosome

Not Applicable

MT Genome

MT Haplogroup 6 - 100.0% Consistent
Includes C57BL/6J, C57BL/6NRj, CBA/J and 165 other strains

MiniMUGA Background Analysis v2.3.1

Backgrounds Detected (Diagnostic Alleles)

Diagnostic Class	Diagnostic Alleles Observed: Homozygous	Heterozygous	Potential	% Observed
C57BL/6J, C57BL/6JJicTac, C57BL/6JRj	0	45	102	44.1%
C57BL/6NRj, C57BL/6NTac	0	11	15	73.3%
C57BL/6J, C57BL/6JRj	0	10	31	32.3%
C57BL/6NJ, C57BL/6NRj, C57BL/6NTac	0	9	10	90.0%
C57BL/6J, C57BL/6JEiJ, C57BL/6JJicTac, C57BL/6JRj	0	8	21	38.1%
B6N-Tyr<c-Brd>/BrdCrCrl, C57BL/6J, C57BL/6JJicTac, C57BL/6JRj	0	4	5	80.0%
C57BL/6NRj	0	3	10	30.0%
B6N-Tyr<c-Brd>/BrdCrCrl, C57BL/6NCrl, C57BL/6NHsd, C57BL/6NJ, C57BL/6NRj, C57BL/6NTac	0	2	2	100.0%
C57BL/6NCrl, C57BL/6NHsd, C57BL/6NJ, C57BL/6NRj, C57BL/6NTac	0	2	2	100.0%
129S5/SvEvBrd	0	1	5	20.0%
B6N-Tyr<c-Brd>/BrdCrCrl, C57BL/6J, C57BL/6JEiJ, C57BL/6JJicTac, C57BL/6JRj	0	1	1	100.0%
C57BL/6J, C57BL/6JBomTac, C57BL/6JEiJ, C57BL/6JJicTac, C57BL/6JOlaHsd, C57BL/6JRj	0	1	2	50.0%
C57BL/6J, C57BL/6JEiJ, C57BL/6JJicTac, C57BL/6JOlaHsd, C57BL/6JRj	0	1	1	100.0%
C57BL/6NHsd, C57BL/6NJ, C57BL/6NRj, C57BL/6NTac	0	1	1	100.0%

Minimal Strain Sets Explaining All Diagnostic Classes (Number of Markers Explained):

- Solution 1: 129S5/SvEvBrd and C57BL/6J and C57BL/6NRj
  - C57BL/6J: 70 / 163 (42.9%)
  - C57BL/6NRj: 28 / 40 (70.0%)
  - 129S5/SvEvBrd: 1 / 5 (20.0%)
- Solution 2: 129S5/SvEvBrd and C57BL/6JRj and C57BL/6NRj
  - C57BL/6JRj: 70 / 163 (42.9%)
  - C57BL/6NRj: 28 / 40 (70.0%)
  - 129S5/SvEvBrd: 1 / 5 (20.0%)

Chromosome	Start (Mb)	Stop (Mb)	Background	Zygosity
1	3000000	61451021	C57BL/6J and C57BL/6NRj and CBA/J	Heterozygous
1	61451021	90903197	C57BL/6J and C57BL/6NRj	N/A
1	90903197	167271106	C57BL/6J and C57BL/6NRj and CBA/J	Heterozygous
1	167271106	195471971	C57BL/6J and C57BL/6NRj	N/A
2	3000000	45666278	C57BL/6J and C57BL/6NRj and CBA/J	Heterozygous
2	45666278	73223831	C57BL/6J and C57BL/6NRj	N/A
2	73223831	154349372	C57BL/6J and C57BL/6NRj and CBA/J	Heterozygous
2	154349372	182113224	C57BL/6J and C57BL/6NRj	N/A
3	3000000	37371933	C57BL/6J and C57BL/6NRj and CBA/J	Heterozygous

MiniMUGA Background Analysis v2.3.1

**Diplotype Intervals**	3	37371933	56655047	C57BL/6J and C57BL/6NRj	N/A
	3	56655047	60027525	C57BL/6J and C57BL/6NRj and CBA/J	Heterozygous
	3	60027525	97902156	C57BL/6J and C57BL/6NRj	N/A
	3	97902156	134049530	C57BL/6J and C57BL/6NRj and CBA/J	Heterozygous
	3	134049530	147169673	C57BL/6J and C57BL/6NRj	N/A
	3	147169673	160039680	C57BL/6J and C57BL/6NRj and CBA/J	Heterozygous
	4	3000000	26280383	C57BL/6J and C57BL/6NRj	N/A
	4	26280383	29346519	C57BL/6J and C57BL/6NRj and CBA/J	Heterozygous
	4	29346519	30650814	Unexplained	Heterozygous
	4	30650814	33527174	C57BL/6J and C57BL/6NRj and CBA/J	Heterozygous
	4	33527174	35563307	C57BL/6J and C57BL/6NRj	N/A
	4	35563307	37995481	Unexplained	Heterozygous
	4	37995481	41348396	Unexplained	Homozygous
	4	41348396	43372387	C57BL/6J and C57BL/6NRj and CBA/J	Heterozygous
	4	43372387	43819249	Unexplained	Heterozygous
	4	43819249	46665692	C57BL/6J and C57BL/6NRj and CBA/J	Heterozygous
	4	46665692	50929602	Unexplained	Heterozygous
	4	50929602	54114833	C57BL/6J and C57BL/6NRj	N/A
	4	54114833	156508116	C57BL/6J and C57BL/6NRj and CBA/J	Heterozygous
	5	3000000	29588943	C57BL/6J and C57BL/6NRj and CBA/J	Heterozygous
	5	29588943	36875036	C57BL/6J and C57BL/6NRj	N/A
	5	36875036	52975754	C57BL/6J and C57BL/6NRj and CBA/J	Heterozygous
	5	52975754	124446826	C57BL/6J and C57BL/6NRj	N/A
	5	124446826	151834684	C57BL/6J and C57BL/6NRj and CBA/J	Heterozygous
	6	3000000	43184432	C57BL/6J and C57BL/6NRj	N/A
	6	43184432	79701235	C57BL/6J and C57BL/6NRj and CBA/J	Heterozygous
	6	79701235	91705499	C57BL/6J and C57BL/6NRj	N/A
	6	91705499	111891908	C57BL/6J and C57BL/6NRj and CBA/J	Heterozygous
	6	111891908	120584622	C57BL/6J and C57BL/6NRj	N/A
	6	120584622	149736546	C57BL/6J and C57BL/6NRj and CBA/J	Heterozygous
	7	3000000	42273938	C57BL/6J and C57BL/6NRj and CBA/J	Heterozygous

MiniMUGA Background Analysis v2.3.1

7	42273938	88139775	C57BL/6J and C57BL/6NRj	N/A
7	88139775	145441459	C57BL/6J and C57BL/6NRj and CBA/J	Heterozygous
8	3000000	28015811	C57BL/6J and C57BL/6NRj and CBA/J	Heterozygous
8	28015811	49918752	C57BL/6J and C57BL/6NRj	N/A
8	49918752	119835722	C57BL/6J and C57BL/6NRj and CBA/J	Heterozygous
8	119835722	129401213	C57BL/6J and C57BL/6NRj	N/A
9	3000000	96036921	C57BL/6J and C57BL/6NRj	N/A
9	96036921	108206968	C57BL/6J and C57BL/6NRj and CBA/J	Heterozygous
9	108206968	124595110	C57BL/6J and C57BL/6NRj	N/A
10	3000000	23654421	C57BL/6J and C57BL/6NRj	N/A
10	23654421	68332199	C57BL/6J and C57BL/6NRj and CBA/J	Heterozygous
10	68332199	130694993	C57BL/6J and C57BL/6NRj	N/A
11	3000000	8694811	Unexplained	Heterozygous
11	8694811	22302070	C57BL/6J and C57BL/6NRj and CBA/J	Heterozygous
11	22302070	34971453	C57BL/6J and C57BL/6NRj	N/A
11	34971453	112771442	C57BL/6J and C57BL/6NRj and CBA/J	Heterozygous
11	112771442	119038285	C57BL/6J and C57BL/6NRj	N/A
11	119038285	122082543	C57BL/6J and C57BL/6NRj and CBA/J	Heterozygous
12	3000000	88650858	C57BL/6J and C57BL/6NRj and CBA/J	Heterozygous
12	88650858	120129022	C57BL/6J and C57BL/6NRj	N/A
13	3000000	60016573	C57BL/6J and C57BL/6NRj	N/A
13	60016573	72382747	C57BL/6J and C57BL/6NRj and CBA/J	Heterozygous
13	72382747	120421639	C57BL/6J and C57BL/6NRj	N/A
14	3000000	120643228	C57BL/6J and C57BL/6NRj and CBA/J	Heterozygous
14	120643228	124902244	C57BL/6J and C57BL/6NRj	N/A
15	3000000	88538882	C57BL/6J and C57BL/6NRj and CBA/J	Heterozygous
15	88538882	104043685	C57BL/6J and C57BL/6NRj	N/A
16	3000000	60597221	C57BL/6J and C57BL/6NRj	N/A
16	60597221	76315797	C57BL/6J and C57BL/6NRj and CBA/J	Heterozygous
16	76315797	86241389	C57BL/6J and C57BL/6NRj	N/A

MiniMUGA Background Analysis v2.3.1

	16	86241389	98207768	C57BL/6J and C57BL/6NRj and CBA/J	Heterozygous
	17	3000000	47545390	C57BL/6J and C57BL/6NRj	N/A
	17	47545390	74502727	C57BL/6J and C57BL/6NRj and CBA/J	Heterozygous
	17	74502727	94987271	C57BL/6J and C57BL/6NRj	N/A
	18	3000000	38237964	C57BL/6J and C57BL/6NRj	N/A
	18	38237964	90702639	C57BL/6J and C57BL/6NRj and CBA/J	Heterozygous
	19	3000000	61431566	C57BL/6J and C57BL/6NRj	N/A
	X	3000000	171031299	C57BL/6J and C57BL/6NRj and CBA/J	Heterozygous
	MT	0	0	IBD	Hemizygous
