# Supplementary material for: Deletion of 9p drives B-ALL through heterozygous inactivation of Pax5 and Cd72 in preleukemic cells
Source: JCI Insight. 2026 Feb 17;11(7):e199464. doi: 10.1172/jci.insight.199464 (PMC13134721; doi:10.1172/jci.insight.199464)
Supplement: Supplemental data set 1 [file jciinsight-11-199464-s204.zip › Strain_Genotyping/A073-results-report.pdf]

MiniMUGA Background Analysis v2.3.1

<table>
<tr><th>Sample ID</th><td>A073</td></tr>
<tr><th>Neogen ID</th><td>AAAU-4507</td></tr>
<tr><th>Summary</th><td>The genotype of this sample is of excellent quality. It is female and outbred, and likely a mix of C57BL/6J and C57BL/6NTac and CBA/J. Clustering of unexplained markers is evidence of an additional background strain.

Diagnostic SNPs are likely explained by the presence of the background strains
Solution 1: 129S5/SvEvBrd and C57BL/6J and C57BL/6NRj
C57BL/6J: 76 / 163 (46.6%)
C57BL/6NRj: 24 / 40 (60.0%)
129S5/SvEvBrd: 1 / 5 (20.0%)
Solution 2: 129S5/SvEvBrd and C57BL/6JRj and C57BL/6NRj
C57BL/6JRj: 76 / 163 (46.6%)
C57BL/6NRj: 24 / 40 (60.0%)
129S5/SvEvBrd: 1 / 5 (20.0%)
NOTE: There is a discrepancy between the diagnostic backgrounds detected and the primary and secondary background analysis (CBA/J, C57BL/6J, C57BL/6NTac). This is uncommon and should be investigated further.

No genetic constructs were detected in this sample.

WARNING:
There is a discrepancy between the diagnostic backgrounds detected ((129S5/SvEvBrd and C57BL/6J and C57BL/6NRj) or (129S5/SvEvBrd and C57BL/6JRj and C57BL/6NRj)) and the primary background (C57BL/6J and C57BL/6NTac) and secondary background (CBA/J). This is uncommon and should be investigated further.
The presence of a single diagnostic heterozygous call for a single inbred strain should be treated with caution.
This sample likely has more than 2 genetic backgrounds (unexplained regions and/or fractured ideogram). The strain selected for secondary background may be incorrect. The estimation of the contribution of primary and secondary background are likely incorrect. This can potentially be addressed with input from the user.</td></tr>
<tr><th>Genotyping Quality</th><td>Excellent (13 N calls)
All reported results are dependent on genotyping quality.</td></tr>
<tr><th>Chromosomal Sex</th><td>XX</td></tr>
<tr><th>Inbreeding Estimate</th><td>42.7% Inbred
(Percentage of the genome (autosomal and X chromosomes) that is homozygous or hemizygous for primary, secondary, and unknown backgrounds. See Genome Analysis)</td></tr>
<tr><th>Constructs Detected</th><td>

BlastR	bpA	Cas9	chlor	cHS4	Cre	DTA	Flp	g_FP	hCMV_a	hCMV_b	hTK_pr	iCre	IRES	Luc	r_FP	rtTA	SV40	tTA
-	-	-	-	-	-	-	-	-	-	-	-	-	-	-	-	-	-	-

</td></tr>
</table>

MiniMUGA Background Analysis v2.3.1

Refined Ideogram

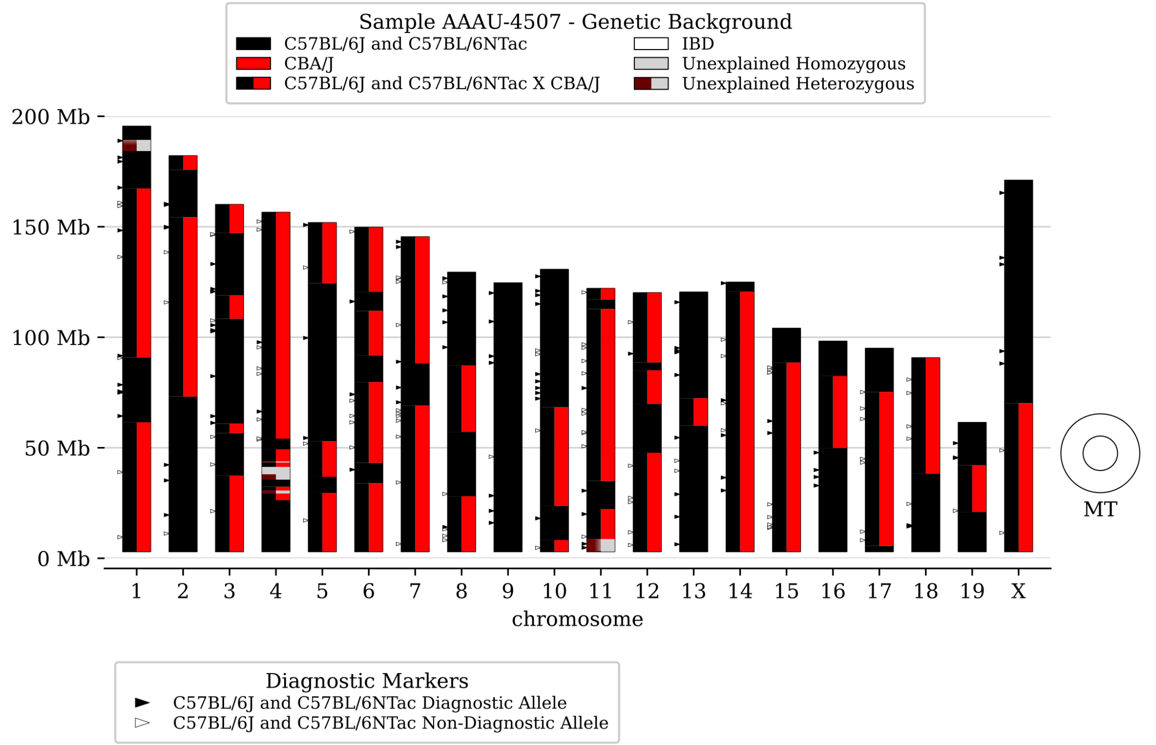

Genome Analysis

Background	Zygosity	Informative Markers	Informative Markers %	Genome %
C57BL/6J and C57BL/6NTac	N/A	1261	47.5%	42.5%
CBA/J	Homozygous	1	0.0%	0.0%
C57BL/6J and C57BL/6NTac X CBA/J	Heterozygous	1361	51.3%	56.7%
Unexplained	Homozygous	5	0.2%	0.1%
Unexplained	Heterozygous	27	1.0%	0.6%
	**Total**	2655	100.0%	99.9%

**Y Chromosome**	Not Applicable
**MT Genome**	MT Haplogroup 6 - 100.0% Consistent<br>Includes C57BL/6J, C57BL/6NTac, CBA/J and 165 other strains

MiniMUGA Background Analysis v2.3.1

Backgrounds Detected (Diagnostic Alleles)

Diagnostic Class	Diagnostic Alleles Observed: Homozygous	Heterozygous	Potential	% Observed
C57BL/6J, C57BL/6JJicTac, C57BL/6JRj	0	51	102	50.0%
C57BL/6J, C57BL/6JEiJ, C57BL/6JJicTac, C57BL/6JRj	0	10	21	47.6%
C57BL/6NRj, C57BL/6NTac	0	10	15	66.7%
C57BL/6J, C57BL/6JRj	0	8	31	25.8%
C57BL/6NJ, C57BL/6NRj, C57BL/6NTac	0	8	10	80.0%
B6N-Tyr<c-Brd>/BrdCrCrl, C57BL/6J, C57BL/6JJicTac, C57BL/6JRj	0	4	5	80.0%
B6N-Tyr<c-Brd>/BrdCrCrl, C57BL/6NCrl, C57BL/6NHsd, C57BL/6NJ, C57BL/6NRj, C57BL/6NTac	0	2	2	100.0%
C57BL/6NCrl, C57BL/6NHsd, C57BL/6NJ, C57BL/6NRj, C57BL/6NTac	0	2	2	100.0%
129S5/SvEvBrd	0	1	5	20.0%
B6N-Tyr<c-Brd>/BrdCrCrl, C57BL/6J, C57BL/6JEiJ, C57BL/6JJicTac, C57BL/6JRj	0	1	1	100.0%
C57BL/6J, C57BL/6JBomTac, C57BL/6JEiJ, C57BL/6JJicTac, C57BL/6JOlaHsd, C57BL/6JRj	0	1	2	50.0%
C57BL/6J, C57BL/6JEiJ, C57BL/6JJicTac, C57BL/6JOlaHsd, C57BL/6JRj	0	1	1	100.0%
C57BL/6NHsd, C57BL/6NJ, C57BL/6NRj, C57BL/6NTac	0	1	1	100.0%
C57BL/6NRj	0	1	10	10.0%

Minimal Strain Sets Explaining All Diagnostic Classes (Number of Markers Explained):

- Solution 1: 129S5/SvEvBrd and C57BL/6J and C57BL/6NRj
    - C57BL/6J: 76 / 163 (46.6%)
    - C57BL/6NRj: 24 / 40 (60.0%)
    - 129S5/SvEvBrd: 1 / 5 (20.0%)
- Solution 2: 129S5/SvEvBrd and C57BL/6JRj and C57BL/6NRj
    - C57BL/6JRj: 76 / 163 (46.6%)
    - C57BL/6NRj: 24 / 40 (60.0%)
    - 129S5/SvEvBrd: 1 / 5 (20.0%)

Chromosome	Start (Mb)	Stop (Mb)	Background	Zygosity
1	3000000	61451021	C57BL/6J and C57BL/6NTac and CBA/J	Heterozygous
1	61451021	90903197	C57BL/6J and C57BL/6NTac	N/A
1	90903197	167271106	C57BL/6J and C57BL/6NTac and CBA/J	Heterozygous
1	167271106	184243385	C57BL/6J and C57BL/6NTac	N/A
1	184243385	189310430	Unexplained	Heterozygous
1	189310430	195471971	C57BL/6J and C57BL/6NTac	N/A
2	3000000	73223831	C57BL/6J and C57BL/6NTac	N/A
2	73223831	154349372	C57BL/6J and C57BL/6NTac and CBA/J	Heterozygous
2	154349372	175780822	C57BL/6J and C57BL/6NTac	N/A

MiniMUGA Background Analysis v2.3.1

**Diplotype Intervals**	2	175780822	182113224	C57BL/6J and C57BL/6NTac and CBA/J	Heterozygous
	3	3000000	37371933	C57BL/6J and C57BL/6NTac and CBA/J	Heterozygous
	3	37371933	56655047	C57BL/6J and C57BL/6NTac	N/A
	3	56655047	60850190	C57BL/6J and C57BL/6NTac and CBA/J	Heterozygous
	3	60850190	108381941	C57BL/6J and C57BL/6NTac	N/A
	3	108381941	118919242	C57BL/6J and C57BL/6NTac and CBA/J	Heterozygous
	3	118919242	147169673	C57BL/6J and C57BL/6NTac	N/A
	3	147169673	160039680	C57BL/6J and C57BL/6NTac and CBA/J	Heterozygous
	4	3000000	26280383	C57BL/6J and C57BL/6NTac	N/A
	4	26280383	29346519	C57BL/6J and C57BL/6NTac and CBA/J	Heterozygous
	4	29346519	30650814	Unexplained	Heterozygous
	4	30650814	32327128	C57BL/6J and C57BL/6NTac and CBA/J	Heterozygous
	4	32327128	35563307	C57BL/6J and C57BL/6NTac	N/A
	4	35563307	37995481	Unexplained	Heterozygous
	4	37995481	41348396	Unexplained	Homozygous
	4	41348396	43372387	C57BL/6J and C57BL/6NTac and CBA/J	Heterozygous
	4	43372387	43819249	Unexplained	Heterozygous
	4	43819249	49280860	C57BL/6J and C57BL/6NTac and CBA/J	Heterozygous
	4	49280860	54114833	C57BL/6J and C57BL/6NTac	N/A
	4	54114833	156508116	C57BL/6J and C57BL/6NTac and CBA/J	Heterozygous
	5	3000000	29588943	C57BL/6J and C57BL/6NTac and CBA/J	Heterozygous
	5	29588943	36875036	C57BL/6J and C57BL/6NTac	N/A
	5	36875036	52975754	C57BL/6J and C57BL/6NTac and CBA/J	Heterozygous
	5	52975754	124446826	C57BL/6J and C57BL/6NTac	N/A
	5	124446826	151834684	C57BL/6J and C57BL/6NTac and CBA/J	Heterozygous
	6	3000000	33933268	C57BL/6J and C57BL/6NTac and CBA/J	Heterozygous
	6	33933268	43184432	C57BL/6J and C57BL/6NTac	N/A
	6	43184432	79701235	C57BL/6J and C57BL/6NTac and CBA/J	Heterozygous
	6	79701235	91705499	C57BL/6J and C57BL/6NTac	N/A
	6	91705499	111891908	C57BL/6J and C57BL/6NTac and CBA/J	Heterozygous

MiniMUGA Background Analysis v2.3.1

6	111891908	120584622	C57BL/6J and C57BL/6NTac	N/A
6	120584622	149736546	C57BL/6J and C57BL/6NTac and CBA/J	Heterozygous
7	3000000	69096424	C57BL/6J and C57BL/6NTac and CBA/J	Heterozygous
7	69096424	88139775	C57BL/6J and C57BL/6NTac	N/A
7	88139775	145441459	C57BL/6J and C57BL/6NTac and CBA/J	Heterozygous
8	3000000	28015811	C57BL/6J and C57BL/6NTac and CBA/J	Heterozygous
8	28015811	57187999	C57BL/6J and C57BL/6NTac	N/A
8	57187999	87222027	C57BL/6J and C57BL/6NTac and CBA/J	Heterozygous
8	87222027	129401213	C57BL/6J and C57BL/6NTac	N/A
9	3000000	124595110	C57BL/6J and C57BL/6NTac	N/A
10	3000000	8205640	C57BL/6J and C57BL/6NTac and CBA/J	Heterozygous
10	8205640	23654421	C57BL/6J and C57BL/6NTac	N/A
10	23654421	68332199	C57BL/6J and C57BL/6NTac and CBA/J	Heterozygous
10	68332199	130694993	C57BL/6J and C57BL/6NTac	N/A
11	3000000	8694811	Unexplained	Heterozygous
11	8694811	22302070	C57BL/6J and C57BL/6NTac and CBA/J	Heterozygous
11	22302070	34971453	C57BL/6J and C57BL/6NTac	N/A
11	34971453	112771442	C57BL/6J and C57BL/6NTac and CBA/J	Heterozygous
11	112771442	117025607	C57BL/6J and C57BL/6NTac	N/A
11	117025607	122082543	C57BL/6J and C57BL/6NTac and CBA/J	Heterozygous
12	3000000	47723179	C57BL/6J and C57BL/6NTac and CBA/J	Heterozygous
12	47723179	69789714	C57BL/6J and C57BL/6NTac	N/A
12	69789714	85015902	C57BL/6J and C57BL/6NTac and CBA/J	Heterozygous
12	85015902	88650858	C57BL/6J and C57BL/6NTac	N/A
12	88650858	120129022	C57BL/6J and C57BL/6NTac and CBA/J	Heterozygous
13	3000000	60016573	C57BL/6J and C57BL/6NTac	N/A
13	60016573	72382747	C57BL/6J and C57BL/6NTac and CBA/J	Heterozygous
13	72382747	120421639	C57BL/6J and C57BL/6NTac	N/A
14	3000000	120643228	C57BL/6J and C57BL/6NTac and CBA/J	Heterozygous
14	120643228	124902244	C57BL/6J and C57BL/6NTac	N/A

MiniMUGA Background Analysis v2.3.1

	15	3000000	88538882	C57BL/6J and C57BL/6NTac and CBA/J	Heterozygous
	15	88538882	104043685	C57BL/6J and C57BL/6NTac	N/A
	16	3000000	49897727	C57BL/6J and C57BL/6NTac	N/A
	16	49897727	82429429	C57BL/6J and C57BL/6NTac and CBA/J	Heterozygous
	16	82429429	98207768	C57BL/6J and C57BL/6NTac	N/A
	17	3000000	5603932	C57BL/6J and C57BL/6NTac	N/A
	17	5603932	75218527	C57BL/6J and C57BL/6NTac and CBA/J	Heterozygous
	17	75218527	94987271	C57BL/6J and C57BL/6NTac	N/A
	18	3000000	38237964	C57BL/6J and C57BL/6NTac	N/A
	18	38237964	90702639	C57BL/6J and C57BL/6NTac and CBA/J	Heterozygous
	19	3000000	20955280	C57BL/6J and C57BL/6NTac	N/A
	19	20955280	42043276	C57BL/6J and C57BL/6NTac and CBA/J	Heterozygous
	19	42043276	61431566	C57BL/6J and C57BL/6NTac	N/A
	X	3000000	70193631	C57BL/6J and C57BL/6NTac and CBA/J	Heterozygous
	X	70193631	171031299	C57BL/6J and C57BL/6NTac	N/A
	MT	0	0	IBD	Hemizygous
