# Supplementary material for: Deletion of 9p drives B-ALL through heterozygous inactivation of Pax5 and Cd72 in preleukemic cells
Source: JCI Insight. 2026 Feb 17;11(7):e199464. doi: 10.1172/jci.insight.199464 (PMC13134721; doi:10.1172/jci.insight.199464)
Supplement: Supplemental data set 1 [file jciinsight-11-199464-s204.zip › Strain_Genotyping/A120-results-report.pdf]

MiniMUGA Background Analysis v2.3.1

**Sample ID**	A120
**Neogen ID**	AAAU-4503
**Summary**	The genotype of this sample is of **excellent** quality. It is **female** and **outbred**, and likely a mix of **C57BL/6J and C57BL/6NTac** and **CBA/J**. Clustering of unexplained markers is evidence of an additional background strain.<br><br>Diagnostic SNPs are likely explained by the presence of the background strains<br>• Solution 1: 129S5/SvEvBrd and C57BL/6J and C57BL/6NRj<br>◦ C57BL/6J: 69 / 162 (42.6%)<br>◦ C57BL/6NRj: 15 / 37 (40.5%)<br>◦ 129S5/SvEvBrd: 1 / 5 (20.0%)<br>• Solution 2: 129S5/SvEvBrd and C57BL/6JRj and C57BL/6NRj<br>◦ C57BL/6JRj: 69 / 162 (42.6%)<br>◦ C57BL/6NRj: 15 / 37 (40.5%)<br>◦ 129S5/SvEvBrd: 1 / 5 (20.0%)<br><br>NOTE: There is a discrepancy between the diagnostic backgrounds detected and the primary and secondary background analysis (CBA/J, C57BL/6J, C57BL/6NTac). This is uncommon and should be investigated further.<br><br>No genetic constructs were detected in this sample.<br><br>WARNING:<br>• There is a discrepancy between the diagnostic backgrounds detected ((129S5/SvEvBrd and C57BL/6J and C57BL/6NRj) or (129S5/SvEvBrd and C57BL/6JRj and C57BL/6NRj)) and the primary background (C57BL/6J and C57BL/6NTac) and secondary background (CBA/J). This is uncommon and should be investigated further.<br>• The presence of a single diagnostic heterozygous call for a single inbred strain should be treated with caution.<br>• This sample likely has more than 2 genetic backgrounds (unexplained regions and/or fractured ideogram). The strain selected for secondary background may be incorrect. The estimation of the contribution of primary and secondary background are likely incorrect. This can potentially be addressed with input from the user.
**Genotyping Quality**	**Excellent (11 N calls)**<br>All reported results are dependent on genotyping quality.
**Chromosomal Sex**	XX
**Inbreeding Estimate**	57.8% Inbred<br>(Percentage of the genome (autosomal and X chromosomes) that is homozygous or hemizygous for primary, secondary, and unknown backgrounds. See Genome Analysis)
**Constructs Detected**	(see below)

Constructs Detected

BlastR	bpA	Cas9	chlor	cHS4	Cre	DTA	Flp	g_FP	hCMV_a	hCMV_b	hTK_pr	iCre	IRES	Luc	r_FP	rtTA	SV40	tTA
-	-	-	-	-	-	-	-	-	-	-	-	-	-	-	-	-	-	-

MiniMUGA Background Analysis v2.3.1

**Refined Ideogram**	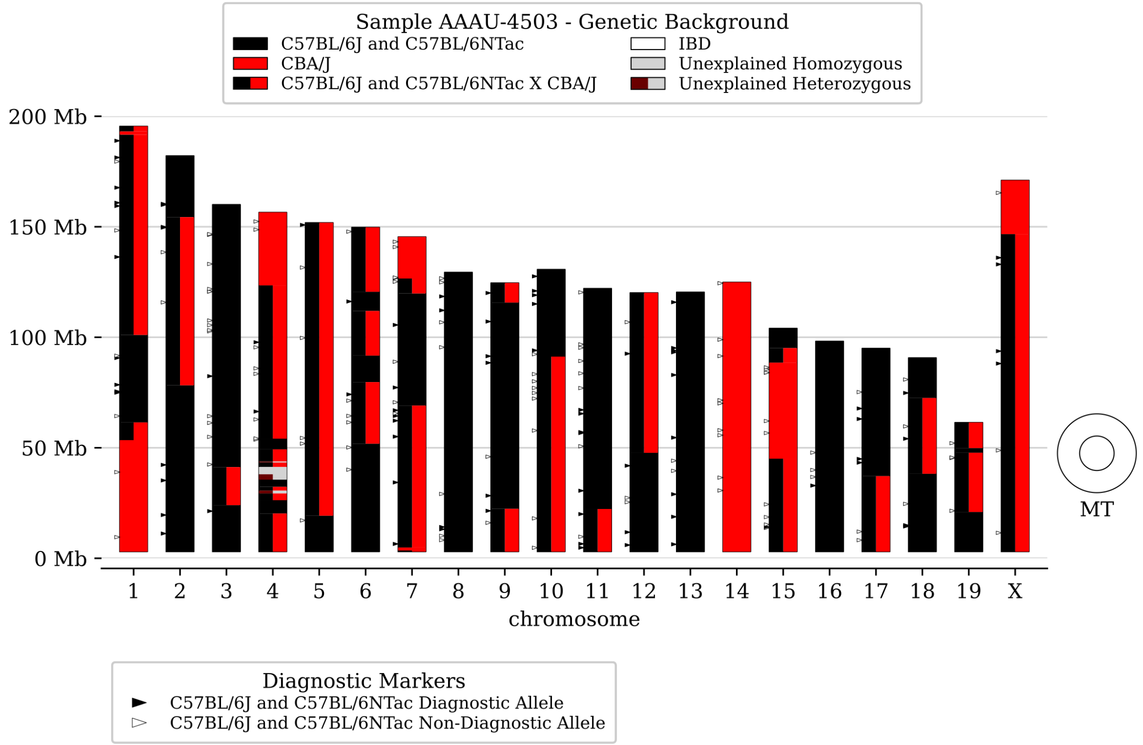

Genome Analysis

Background	Zygosity	Informative Markers	Informative Markers %	Genome %
C57BL/6J and C57BL/6NTac	N/A	1190	44.8%	46.2%
CBA/J	Homozygous	287	10.8%	11.5%
C57BL/6J and C57BL/6NTac X CBA/J	Heterozygous	1165	43.8%	42.0%
Unexplained	Homozygous	5	0.2%	0.1%
Unexplained	Heterozygous	11	0.4%	0.2%
	**Total**	2658	100.0%	100.0%

**Y Chromosome**	Not Applicable
**MT Genome**	MT Haplogroup 6 - 100.0% Consistent<br>Includes C57BL/6J, C57BL/6NTac, CBA/J and 165 other strains

MiniMUGA Background Analysis v2.3.1

Backgrounds Detected (Diagnostic Alleles)

	Diagnostic Alleles Observed			
**Diagnostic Class**	**Homozygous**	**Heterozygous**	**Potential**	**% Observed**
C57BL/6J, C57BL/6JJicTac, C57BL/6JRj	6	43	102	48.0%
C57BL/6NRj, C57BL/6NTac	4	3	15	46.7%
C57BL/6J, C57BL/6JEiJ, C57BL/6JJicTac, C57BL/6JRj	0	9	21	42.9%
C57BL/6J, C57BL/6JRj	1	7	31	25.8%
C57BL/6NJ, C57BL/6NRj, C57BL/6NTac	1	5	10	60.0%
129S5/SvEvBrd	0	1	5	20.0%
B6N-Tyr<c-Brd>/BrdCrCrl, C57BL/6J, C57BL/6JEiJ, C57BL/6JJicTac, C57BL/6JRj	0	1	1	100.0%
B6N-Tyr<c-Brd>/BrdCrCrl, C57BL/6J, C57BL/6JJicTac, C57BL/6JRj	0	1	5	20.0%
B6N-Tyr<c-Brd>/BrdCrCrl, C57BL/6NCrl, C57BL/6NHsd, C57BL/6NJ, C57BL/6NRj, C57BL/6NTac	0	1	2	50.0%
C57BL/6J, C57BL/6JBomTac, C57BL/6JEiJ, C57BL/6JJicTac, C57BL/6JOlaHsd, C57BL/6JRj	0	1	2	50.0%
C57BL/6NRj	0	1	10	10.0%

Minimal Strain Sets Explaining All Diagnostic Classes (Number of Markers Explained):

- Solution 1: 129S5/SvEvBrd and C57BL/6J and C57BL/6NRj
  - C57BL/6J: 69 / 162 (42.6%)
  - C57BL/6NRj: 15 / 37 (40.5%)
  - 129S5/SvEvBrd: 1 / 5 (20.0%)
- Solution 2: 129S5/SvEvBrd and C57BL/6JRj and C57BL/6NRj
  - C57BL/6JRj: 69 / 162 (42.6%)
  - C57BL/6NRj: 15 / 37 (40.5%)
  - 129S5/SvEvBrd: 1 / 5 (20.0%)

Chromosome	Start (Mb)	Stop (Mb)	Background	Zygosity
1	3000000	53457225	CBA/J	Homozygous
1	53457225	61451021	C57BL/6J and C57BL/6NTac and CBA/J	Heterozygous
1	61451021	101065154	C57BL/6J and C57BL/6NTac	N/A
1	101065154	191629867	C57BL/6J and C57BL/6NTac and CBA/J	Heterozygous
1	191629867	193164602	CBA/J	Homozygous
1	193164602	195471971	C57BL/6J and C57BL/6NTac and CBA/J	Heterozygous
2	3000000	78267191	C57BL/6J and C57BL/6NTac	N/A
2	78267191	154349372	C57BL/6J and C57BL/6NTac and CBA/J	Heterozygous
2	154349372	182113224	C57BL/6J and C57BL/6NTac	N/A
3	3000000	24042899	C57BL/6J and C57BL/6NTac	N/A
3	24042899	41244072	C57BL/6J and C57BL/6NTac and CBA/J	Heterozygous
3	41244072	160039680	C57BL/6J and C57BL/6NTac	N/A

MiniMUGA Background Analysis v2.3.1

**Diplotype Intervals**	4	3000000	20258658	C57BL/6J and C57BL/6NTac and CBA/J	Heterozygous
	4	20258658	26280383	C57BL/6J and C57BL/6NTac	N/A
	4	26280383	29346519	C57BL/6J and C57BL/6NTac and CBA/J	Heterozygous
	4	29346519	30650814	Unexplained	Heterozygous
	4	30650814	32327128	C57BL/6J and C57BL/6NTac and CBA/J	Heterozygous
	4	32327128	35563307	C57BL/6J and C57BL/6NTac	N/A
	4	35563307	37995481	Unexplained	Heterozygous
	4	37995481	41348396	Unexplained	Homozygous
	4	41348396	43372387	C57BL/6J and C57BL/6NTac and CBA/J	Heterozygous
	4	43372387	43819249	Unexplained	Heterozygous
	4	43819249	49280860	C57BL/6J and C57BL/6NTac and CBA/J	Heterozygous
	4	49280860	54114833	C57BL/6J and C57BL/6NTac	N/A
	4	54114833	123466633	C57BL/6J and C57BL/6NTac and CBA/J	Heterozygous
	4	123466633	156508116	CBA/J	Homozygous
	5	3000000	19267794	C57BL/6J and C57BL/6NTac	N/A
	5	19267794	151834684	C57BL/6J and C57BL/6NTac and CBA/J	Heterozygous
	6	3000000	51852686	C57BL/6J and C57BL/6NTac	N/A
	6	51852686	79701235	C57BL/6J and C57BL/6NTac and CBA/J	Heterozygous
	6	79701235	91705499	C57BL/6J and C57BL/6NTac	N/A
	6	91705499	111891908	C57BL/6J and C57BL/6NTac and CBA/J	Heterozygous
	6	111891908	120584622	C57BL/6J and C57BL/6NTac	N/A
	6	120584622	149736546	C57BL/6J and C57BL/6NTac and CBA/J	Heterozygous
	7	3000000	3525314	C57BL/6J and C57BL/6NTac and CBA/J	Heterozygous
	7	3525314	4876138	CBA/J	Homozygous
	7	4876138	69096424	C57BL/6J and C57BL/6NTac and CBA/J	Heterozygous
	7	69096424	119823617	C57BL/6J and C57BL/6NTac	N/A
	7	119823617	126580094	C57BL/6J and C57BL/6NTac and CBA/J	Heterozygous
	7	126580094	145441459	CBA/J	Homozygous
	8	3000000	129401213	C57BL/6J and C57BL/6NTac	N/A
	9	3000000	22379457	C57BL/6J and C57BL/6NTac and CBA/J	Heterozygous
	9	22379457	115715944	C57BL/6J and C57BL/6NTac	N/A

MiniMUGA Background Analysis v2.3.1

	9	115715944	124595110	C57BL/6J and C57BL/6NTac and CBA/J	Heterozygous
	10	3000000	91235291	C57BL/6J and C57BL/6NTac and CBA/J	Heterozygous
	10	91235291	130694993	C57BL/6J and C57BL/6NTac	N/A
	11	3000000	22302070	C57BL/6J and C57BL/6NTac and CBA/J	Heterozygous
	11	22302070	122082543	C57BL/6J and C57BL/6NTac	N/A
	12	3000000	47723179	C57BL/6J and C57BL/6NTac	N/A
	12	47723179	120129022	C57BL/6J and C57BL/6NTac and CBA/J	Heterozygous
	13	3000000	120421639	C57BL/6J and C57BL/6NTac	N/A
	14	3000000	124902244	CBA/J	Homozygous
	15	3000000	45058066	C57BL/6J and C57BL/6NTac and CBA/J	Heterozygous
	15	45058066	88538882	CBA/J	Homozygous
	15	88538882	95109176	C57BL/6J and C57BL/6NTac and CBA/J	Heterozygous
	15	95109176	104043685	C57BL/6J and C57BL/6NTac	N/A
	16	3000000	98207768	C57BL/6J and C57BL/6NTac	N/A
	17	3000000	37167871	C57BL/6J and C57BL/6NTac and CBA/J	Heterozygous
	17	37167871	94987271	C57BL/6J and C57BL/6NTac	N/A
	18	3000000	38237964	C57BL/6J and C57BL/6NTac	N/A
	18	38237964	72510867	C57BL/6J and C57BL/6NTac and CBA/J	Heterozygous
	18	72510867	90702639	C57BL/6J and C57BL/6NTac	N/A
	19	3000000	20955280	C57BL/6J and C57BL/6NTac	N/A
	19	20955280	47746251	C57BL/6J and C57BL/6NTac and CBA/J	Heterozygous
	19	47746251	49870985	C57BL/6J and C57BL/6NTac	N/A
	19	49870985	61431566	C57BL/6J and C57BL/6NTac and CBA/J	Heterozygous
	X	3000000	146651558	C57BL/6J and C57BL/6NTac and CBA/J	Heterozygous
	X	146651558	171031299	CBA/J	Homozygous
	MT	0	0	IBD	Hemizygous
