# Supplementary material for: Deletion of 9p drives B-ALL through heterozygous inactivation of Pax5 and Cd72 in preleukemic cells
Source: JCI Insight. 2026 Feb 17;11(7):e199464. doi: 10.1172/jci.insight.199464 (PMC13134721; doi:10.1172/jci.insight.199464)
Supplement: Supplemental data set 1 [file jciinsight-11-199464-s204.zip › Strain_Genotyping/A121-results-report.pdf]

MiniMUGA Background Analysis v2.3.1

**Sample ID**	A121
**Neogen ID**	AAAU-4506

Summary

The genotype of this sample is of **excellent** quality. It is **female** and **outbred**, and likely a mix of **C57BL/6J and C57BL/6NRj** and **CBA/J**. Clustering of unexplained markers is evidence of an additional background strain.

Diagnostic SNPs are likely explained by the presence of the background strains

- Solution 1: 129S5/SvEvBrd and C57BL/6J and C57BL/6NRj
    - C57BL/6J: 52 / 155 (33.5%)
    - C57BL/6NRj: 21 / 40 (52.5%)
    - 129S5/SvEvBrd: 1 / 5 (20.0%)
- Solution 2: 129S5/SvEvBrd and C57BL/6JRj and C57BL/6NRj
    - C57BL/6JRj: 52 / 155 (33.5%)
    - C57BL/6NRj: 21 / 40 (52.5%)
    - 129S5/SvEvBrd: 1 / 5 (20.0%)

NOTE: There is a discrepancy between the diagnostic backgrounds detected and the primary and secondary background analysis (C57BL/6NRj, CBA/J, C57BL/6J). This is uncommon and should be investigated further.

No genetic constructs were detected in this sample.

WARNING:

- There is a discrepancy between the diagnostic backgrounds detected ((129S5/SvEvBrd and C57BL/6J and C57BL/6NRj) or (129S5/SvEvBrd and C57BL/6JRj and C57BL/6NRj)) and the primary background (C57BL/6J and C57BL/6NRj) and secondary background (CBA/J). This is uncommon and should be investigated further.
- The presence of a single diagnostic heterozygous call for a single inbred strain should be treated with caution.
- This sample likely has more than 2 genetic backgrounds (unexplained regions and/or fractured ideogram). The strain selected for secondary background may be incorrect. The estimation of the contribution of primary and secondary background are likely incorrect. This can potentially be addressed with input from the user.

**Genotyping Quality**	**Excellent (19 N calls)**<br>All reported results are dependent on genotyping quality.
**Chromosomal Sex**	XX
**Inbreeding Estimate**	49.2% Inbred<br>(Percentage of the genome (autosomal and X chromosomes) that is homozygous or hemizygous for primary, secondary, and unknown backgrounds. See Genome Analysis)

Constructs Detected

BlastR	bpA	Cas9	chlor	cHS4	Cre	DTA	Flp	g_FP	hCMV_a	hCMV_b	hTK_pr	iCre	IRES	Luc	r_FP	rtTA	SV40	tTA
-	-	-	-	-	-	-	-	-	-	-	-	-	-	-	-	-	-	-

MiniMUGA Background Analysis v2.3.1

Refined Ideogram

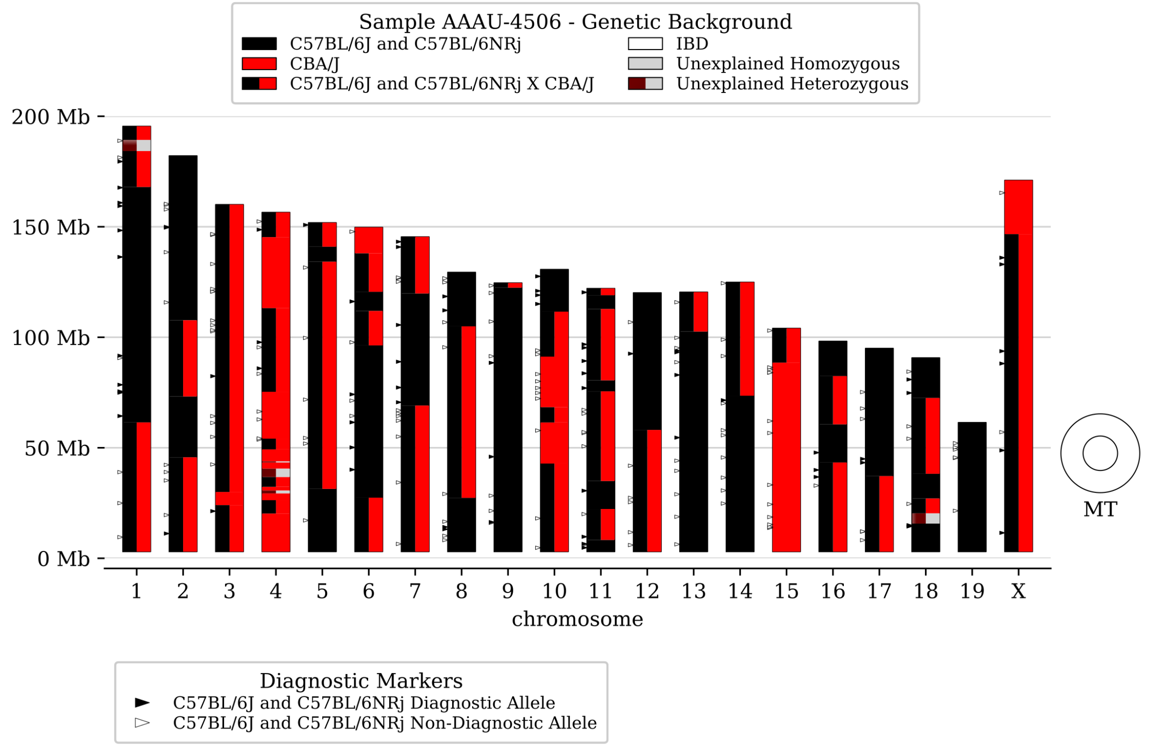

Genome Analysis

Background	Zygosity	Informative Markers	Informative Markers %	Genome %
C57BL/6J and C57BL/6NRj	N/A	1047	39.4%	39.3%
CBA/J	Homozygous	211	7.9%	9.8%
C57BL/6J and C57BL/6NRj X CBA/J	Heterozygous	1381	51.9%	50.3%
Unexplained	Heterozygous	21	0.8%	0.6%
	**Total**	2660	100.0%	100.0%

Y Chromosome

Not Applicable

MT Genome

MT Haplogroup 6 - 100.0% Consistent
Includes C57BL/6J, C57BL/6NRj, CBA/J and 165 other strains

MiniMUGA Background Analysis v2.3.1

Backgrounds Detected (Diagnostic Alleles)

	Diagnostic Alleles Observed			
**Diagnostic Class**	**Homozygous**	**Heterozygous**	**Potential**	**% Observed**
C57BL/6J, C57BL/6JJicTac, C57BL/6JRj	5	29	102	33.3%
C57BL/6J, C57BL/6JEiJ, C57BL/6JJicTac, C57BL/6JRj	2	8	21	47.6%
C57BL/6NJ, C57BL/6NRj, C57BL/6NTac	4	4	10	80.0%
C57BL/6NRj, C57BL/6NTac	3	4	15	46.7%
C57BL/6J, C57BL/6JRj	0	7	31	22.6%
B6N-Tyr<c-Brd>/BrdCrCrl, C57BL/6NCrl, C57BL/6NHsd, C57BL/6NJ, C57BL/6NRj, C57BL/6NTac	1	1	2	100.0%
C57BL/6J, C57BL/6JEiJ, C57BL/6JJicTac, C57BL/6JOlaHsd, C57BL/6JRj	1	0	1	100.0%
C57BL/6NCrl, C57BL/6NHsd, C57BL/6NJ, C57BL/6NRj, C57BL/6NTac	0	2	2	100.0%
129S5/SvEvBrd	0	1	5	20.0%
C57BL/6NHsd, C57BL/6NJ, C57BL/6NRj, C57BL/6NTac	0	1	1	100.0%
C57BL/6NRj	0	1	10	10.0%

Minimal Strain Sets Explaining All Diagnostic Classes (Number of Markers Explained):

- Solution 1: 129S5/SvEvBrd and C57BL/6J and C57BL/6NRj
  - C57BL/6J: 52 / 155 (33.5%)
  - C57BL/6NRj: 21 / 40 (52.5%)
  - 129S5/SvEvBrd: 1 / 5 (20.0%)
- Solution 2: 129S5/SvEvBrd and C57BL/6JRj and C57BL/6NRj
  - C57BL/6JRj: 52 / 155 (33.5%)
  - C57BL/6NRj: 21 / 40 (52.5%)
  - 129S5/SvEvBrd: 1 / 5 (20.0%)

Chromosome	Start (Mb)	Stop (Mb)	Background	Zygosity
1	3000000	61451021	C57BL/6J and C57BL/6NRj and CBA/J	Heterozygous
1	61451021	168019536	C57BL/6J and C57BL/6NRj	N/A
1	168019536	184243385	C57BL/6J and C57BL/6NRj and CBA/J	Heterozygous
1	184243385	189310430	Unexplained	Heterozygous
1	189310430	195471971	C57BL/6J and C57BL/6NRj and CBA/J	Heterozygous
2	3000000	45666278	C57BL/6J and C57BL/6NRj and CBA/J	Heterozygous
2	45666278	73223831	C57BL/6J and C57BL/6NRj	N/A
2	73223831	107690757	C57BL/6J and C57BL/6NRj and CBA/J	Heterozygous
2	107690757	182113224	C57BL/6J and C57BL/6NRj	N/A
3	3000000	24042899	C57BL/6J and C57BL/6NRj and CBA/J	Heterozygous
3	24042899	30013882	CBA/J	Homozygous
3	30013882	160039680	C57BL/6J and C57BL/6NRj and CBA/J	Heterozygous
4	3000000	20258658	CBA/J	Homozygous

MiniMUGA Background Analysis v2.3.1

**Diplotype Intervals**	4	20258658	26280383	C57BL/6J and C57BL/6NRj and CBA/J	Heterozygous
	4	26280383	29346519	CBA/J	Homozygous
	4	29346519	30650814	Unexplained	Heterozygous
	4	30650814	32327128	CBA/J	Homozygous
	4	32327128	36784495	C57BL/6J and C57BL/6NRj and CBA/J	Heterozygous
	4	36784495	40531709	Unexplained	Heterozygous
	4	40531709	43372387	CBA/J	Homozygous
	4	43372387	43819249	Unexplained	Heterozygous
	4	43819249	49280860	CBA/J	Homozygous
	4	49280860	54114833	C57BL/6J and C57BL/6NRj and CBA/J	Heterozygous
	4	54114833	75318594	CBA/J	Homozygous
	4	75318594	113155495	C57BL/6J and C57BL/6NRj and CBA/J	Heterozygous
	4	113155495	145315418	CBA/J	Homozygous
	4	145315418	156508116	C57BL/6J and C57BL/6NRj and CBA/J	Heterozygous
	5	3000000	31408123	C57BL/6J and C57BL/6NRj	N/A
	5	31408123	134172373	C57BL/6J and C57BL/6NRj and CBA/J	Heterozygous
	5	134172373	140985717	C57BL/6J and C57BL/6NRj	N/A
	5	140985717	151834684	C57BL/6J and C57BL/6NRj and CBA/J	Heterozygous
	6	3000000	27400239	C57BL/6J and C57BL/6NRj and CBA/J	Heterozygous
	6	27400239	96327282	C57BL/6J and C57BL/6NRj	N/A
	6	96327282	111891908	C57BL/6J and C57BL/6NRj and CBA/J	Heterozygous
	6	111891908	120584622	C57BL/6J and C57BL/6NRj	N/A
	6	120584622	137951677	C57BL/6J and C57BL/6NRj and CBA/J	Heterozygous
	6	137951677	149736546	CBA/J	Homozygous
	7	3000000	69096424	C57BL/6J and C57BL/6NRj and CBA/J	Heterozygous
	7	69096424	119823617	C57BL/6J and C57BL/6NRj	N/A
	7	119823617	145441459	C57BL/6J and C57BL/6NRj and CBA/J	Heterozygous
	8	3000000	27348459	C57BL/6J and C57BL/6NRj	N/A
	8	27348459	104937322	C57BL/6J and C57BL/6NRj and CBA/J	Heterozygous
	8	104937322	129401213	C57BL/6J and C57BL/6NRj	N/A
	9	3000000	122442159	C57BL/6J and C57BL/6NRj	N/A
	9	122442159	124595110	C57BL/6J and C57BL/6NRj and CBA/J	Heterozygous

MiniMUGA Background Analysis v2.3.1

	10	3000000	42858234	C57BL/6J and C57BL/6NRj and CBA/J	Heterozygous
	10	42858234	61450853	CBA/J	Homozygous
	10	61450853	68332199	C57BL/6J and C57BL/6NRj and CBA/J	Heterozygous
	10	68332199	91235291	CBA/J	Homozygous
	10	91235291	111566142	C57BL/6J and C57BL/6NRj and CBA/J	Heterozygous
	10	111566142	130694993	C57BL/6J and C57BL/6NRj	N/A
	11	3000000	8304515	C57BL/6J and C57BL/6NRj	N/A
	11	8304515	22302070	C57BL/6J and C57BL/6NRj and CBA/J	Heterozygous
	11	22302070	34971453	C57BL/6J and C57BL/6NRj	N/A
	11	34971453	75520821	C57BL/6J and C57BL/6NRj and CBA/J	Heterozygous
	11	75520821	80516310	C57BL/6J and C57BL/6NRj	N/A
	11	80516310	112771442	C57BL/6J and C57BL/6NRj and CBA/J	Heterozygous
	11	112771442	119038285	C57BL/6J and C57BL/6NRj	N/A
	11	119038285	122082543	C57BL/6J and C57BL/6NRj and CBA/J	Heterozygous
	12	3000000	58069123	C57BL/6J and C57BL/6NRj and CBA/J	Heterozygous
	12	58069123	120129022	C57BL/6J and C57BL/6NRj	N/A
	13	3000000	102595519	C57BL/6J and C57BL/6NRj	N/A
	13	102595519	120421639	C57BL/6J and C57BL/6NRj and CBA/J	Heterozygous
	14	3000000	73554565	C57BL/6J and C57BL/6NRj	N/A
	14	73554565	124902244	C57BL/6J and C57BL/6NRj and CBA/J	Heterozygous
	15	3000000	88538882	CBA/J	Homozygous
	15	88538882	104043685	C57BL/6J and C57BL/6NRj and CBA/J	Heterozygous
	16	3000000	43324710	C57BL/6J and C57BL/6NRj and CBA/J	Heterozygous
	16	43324710	60597221	C57BL/6J and C57BL/6NRj	N/A
	16	60597221	82429429	C57BL/6J and C57BL/6NRj and CBA/J	Heterozygous
	16	82429429	98207768	C57BL/6J and C57BL/6NRj	N/A
	17	3000000	37167871	C57BL/6J and C57BL/6NRj and CBA/J	Heterozygous
	17	37167871	94987271	C57BL/6J and C57BL/6NRj	N/A
	18	3000000	15685654	C57BL/6J and C57BL/6NRj	N/A
	18	15685654	20363699	Unexplained	Heterozygous

MiniMUGA Background Analysis v2.3.1

	18	20363699	27036500	C57BL/6J and C57BL/6NRj and CBA/J	Heterozygous
	18	27036500	38237964	C57BL/6J and C57BL/6NRj	N/A
	18	38237964	72510867	C57BL/6J and C57BL/6NRj and CBA/J	Heterozygous
	18	72510867	90702639	C57BL/6J and C57BL/6NRj	N/A
	19	3000000	61431566	C57BL/6J and C57BL/6NRj	N/A
	X	3000000	146651558	C57BL/6J and C57BL/6NRj and CBA/J	Heterozygous
	X	146651558	171031299	CBA/J	Homozygous
	MT	0	0	IBD	Hemizygous
