# Supplementary material for: Deletion of 9p drives B-ALL through heterozygous inactivation of Pax5 and Cd72 in preleukemic cells
Source: JCI Insight. 2026 Feb 17;11(7):e199464. doi: 10.1172/jci.insight.199464 (PMC13134721; doi:10.1172/jci.insight.199464)
Supplement: Supplemental data set 1 [file jciinsight-11-199464-s204.zip › Strain_Genotyping/B147-results-report.pdf]

MiniMUGA Background Analysis v2.3.1

Sample ID

B147

Neogen ID

AAAU-4547

Summary

The genotype of this sample is of **excellent** quality. It is **female** and **outbred**, and likely a mix of **C57BL/6J and C57BL/6NTac** and **CBA/J**. Clustering of unexplained markers is evidence of an additional background strain.

Diagnostic SNPs are likely explained by the presence of the background strains

- Solution 1: 129S5/SvEvBrd and C57BL/6J and C57BL/6NTac
  - C57BL/6J: 78 / 156 (50.0%)
  - C57BL/6NTac: 8 / 25 (32.0%)
  - 129S5/SvEvBrd: 1 / 5 (20.0%)
- Solution 2: 129S5/SvEvBrd and C57BL/6J and C57BL/6NRj
  - C57BL/6J: 78 / 156 (50.0%)
  - C57BL/6NRj: 8 / 25 (32.0%)
  - 129S5/SvEvBrd: 1 / 5 (20.0%)
- Solution 3: 129S5/SvEvBrd and C57BL/6JRj and C57BL/6NTac
  - C57BL/6JRj: 78 / 156 (50.0%)
  - C57BL/6NTac: 8 / 25 (32.0%)
  - 129S5/SvEvBrd: 1 / 5 (20.0%)
- Solution 4: 129S5/SvEvBrd and C57BL/6JRj and C57BL/6NRj
  - C57BL/6JRj: 78 / 156 (50.0%)
  - C57BL/6NRj: 8 / 25 (32.0%)
  - 129S5/SvEvBrd: 1 / 5 (20.0%)

NOTE: There is a discrepancy between the diagnostic backgrounds detected and the primary and secondary background analysis (CBA/J, C57BL/6J, C57BL/6NTac). This is uncommon and should be investigated further.

No genetic constructs were detected in this sample.

WARNING:

- There is a discrepancy between the diagnostic backgrounds detected ((129S5/SvEvBrd and C57BL/6J and C57BL/6NTac) or (129S5/SvEvBrd and C57BL/6J and C57BL/6NRj) or (129S5/SvEvBrd and C57BL/6JRj and C57BL/6NTac) or (129S5/SvEvBrd and C57BL/6JRj and C57BL/6NRj)) and the primary background (C57BL/6J and C57BL/6NTac) and secondary background (CBA/J). This is uncommon and should be investigated further.
- The presence of a single diagnostic heterozygous call for a single inbred strain should be treated with caution.
- This sample likely has more than 2 genetic backgrounds (unexplained regions and/or fractured ideogram). The strain selected for secondary background may be incorrect. The estimation of the contribution of primary and secondary background are likely incorrect. This can potentially be addressed with input from the user.

Genotyping Quality

Excellent (4 N calls)
All reported results are dependent on genotyping quality.

Chromosomal Sex

XX

Inbreeding Estimate

35.9% Inbred
(Percentage of the genome (autosomal and X chromosomes) that is homozygous or hemizygous for primary, secondary, and unknown backgrounds. See Genome Analysis)

Constructs Detected

BlastR	bpA	Cas9	chlor	cHS4	Cre	DTA	Flp	g_FP	hCMV_a	hCMV_b	hTK_pr	iCre	IRES	Luc	r_FP	rtTA	SV40	tTA
-	-	-	-	-	-	-	-	-	-	-	-	-	-	-	-	-	-	-

MiniMUGA Background Analysis v2.3.1

Refined Ideogram

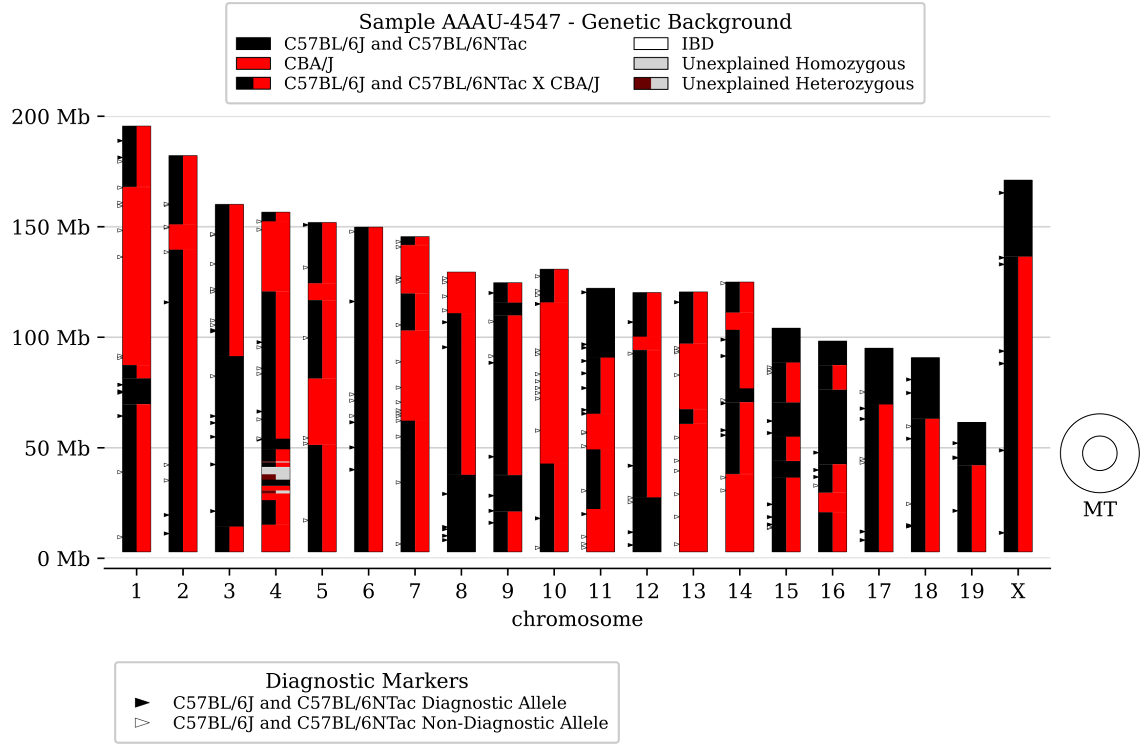

Genome Analysis

Background	Zygosity	Informative Markers	Informative Markers %	Genome %
C57BL/6J and C57BL/6NTac	N/A	505	19.0%	15.8%
CBA/J	Homozygous	485	18.3%	20.0%
C57BL/6J and C57BL/6NTac X CBA/J	Heterozygous	1652	62.2%	64.0%
Unexplained	Homozygous	5	0.2%	0.1%
Unexplained	Heterozygous	9	0.3%	0.2%
	**Total**	2656	100.0%	100.1%

**Y Chromosome**	Not Applicable
**MT Genome**	MT Haplogroup 6 - 100.0% Consistent<br>Includes C57BL/6J, C57BL/6NTac, CBA/J and 165 other strains

MiniMUGA Background Analysis v2.3.1

Backgrounds Detected (Diagnostic Alleles)

	Diagnostic Alleles Observed			
**Diagnostic Class**	**Homozygous**	**Heterozygous**	**Potential**	**% Observed**
C57BL/6J, C57BL/6JJicTac, C57BL/6JRj	12	37	102	48.0%
C57BL/6J, C57BL/6JRj	1	14	31	48.4%
C57BL/6J, C57BL/6JEiJ, C57BL/6JJicTac, C57BL/6JRj	2	10	21	57.1%
C57BL/6NRj, C57BL/6NTac	0	5	15	33.3%
C57BL/6NJ, C57BL/6NRj, C57BL/6NTac	0	3	10	30.0%
129S5/SvEvBrd	0	1	5	20.0%
B6N-Tyr<c-Brd>/BrdCrCrl, C57BL/6J, C57BL/6JEiJ, C57BL/6JJicTac, C57BL/6JRj	0	1	1	100.0%
C57BL/6J, C57BL/6JEiJ, C57BL/6JJicTac, C57BL/6JOlaHsd, C57BL/6JRj	0	1	1	100.0%

Minimal Strain Sets Explaining All Diagnostic Classes (Number of Markers Explained):

- Solution 1: 129S5/SvEvBrd and C57BL/6J and C57BL/6NTac
  - C57BL/6J: 78 / 156 (50.0%)
  - C57BL/6NTac: 8 / 25 (32.0%)
  - 129S5/SvEvBrd: 1 / 5 (20.0%)
- Solution 2: 129S5/SvEvBrd and C57BL/6J and C57BL/6NRj
  - C57BL/6J: 78 / 156 (50.0%)
  - C57BL/6NRj: 8 / 25 (32.0%)
  - 129S5/SvEvBrd: 1 / 5 (20.0%)
- Solution 3: 129S5/SvEvBrd and C57BL/6JRj and C57BL/6NTac
  - C57BL/6JRj: 78 / 156 (50.0%)
  - C57BL/6NTac: 8 / 25 (32.0%)
  - 129S5/SvEvBrd: 1 / 5 (20.0%)
- Solution 4: 129S5/SvEvBrd and C57BL/6JRj and C57BL/6NRj
  - C57BL/6JRj: 78 / 156 (50.0%)
  - C57BL/6NRj: 8 / 25 (32.0%)
  - 129S5/SvEvBrd: 1 / 5 (20.0%)

Chromosome	Start (Mb)	Stop (Mb)	Background	Zygosity
1	3000000	69700765	C57BL/6J and C57BL/6NTac and CBA/J	Heterozygous
1	69700765	81439894	C57BL/6J and C57BL/6NTac	N/A
1	81439894	87433360	C57BL/6J and C57BL/6NTac and CBA/J	Heterozygous
1	87433360	168019536	CBA/J	Homozygous
1	168019536	195471971	C57BL/6J and C57BL/6NTac and CBA/J	Heterozygous
2	3000000	139631657	C57BL/6J and C57BL/6NTac and CBA/J	Heterozygous
2	139631657	151062687	CBA/J	Homozygous
2	151062687	182113224	C57BL/6J and C57BL/6NTac and CBA/J	Heterozygous
3	3000000	14328941	C57BL/6J and C57BL/6NTac and CBA/J	Heterozygous
3	14328941	91461564	C57BL/6J and C57BL/6NTac	N/A
3	91461564	160039680	C57BL/6J and C57BL/6NTac and CBA/J	Heterozygous
4	3000000	15188739	CBA/J	Homozygous

MiniMUGA Background Analysis v2.3.1

**Diplotype Intervals**	4	15188739	26280383	C57BL/6J and C57BL/6NTac and CBA/J	Heterozygous
	4	26280383	29346519	CBA/J	Homozygous
	4	29346519	30650814	Unexplained	Heterozygous
	4	30650814	32777492	CBA/J	Homozygous
	4	32777492	35563307	C57BL/6J and C57BL/6NTac	N/A
	4	35563307	37995481	Unexplained	Heterozygous
	4	37995481	41348396	Unexplained	Homozygous
	4	41348396	43372387	C57BL/6J and C57BL/6NTac and CBA/J	Heterozygous
	4	43372387	43819249	Unexplained	Heterozygous
	4	43819249	49280860	C57BL/6J and C57BL/6NTac and CBA/J	Heterozygous
	4	49280860	54114833	C57BL/6J and C57BL/6NTac	N/A
	4	54114833	120738488	C57BL/6J and C57BL/6NTac and CBA/J	Heterozygous
	4	120738488	152440879	CBA/J	Homozygous
	4	152440879	156508116	C57BL/6J and C57BL/6NTac and CBA/J	Heterozygous
	5	3000000	51299144	C57BL/6J and C57BL/6NTac and CBA/J	Heterozygous
	5	51299144	81363817	CBA/J	Homozygous
	5	81363817	116795433	C57BL/6J and C57BL/6NTac and CBA/J	Heterozygous
	5	116795433	124446826	CBA/J	Homozygous
	5	124446826	151834684	C57BL/6J and C57BL/6NTac and CBA/J	Heterozygous
	6	3000000	149736546	C57BL/6J and C57BL/6NTac and CBA/J	Heterozygous
	7	3000000	62277367	C57BL/6J and C57BL/6NTac and CBA/J	Heterozygous
	7	62277367	103084424	CBA/J	Homozygous
	7	103084424	119823617	C57BL/6J and C57BL/6NTac and CBA/J	Heterozygous
	7	119823617	141750158	CBA/J	Homozygous
	7	141750158	145441459	C57BL/6J and C57BL/6NTac and CBA/J	Heterozygous
	8	3000000	37790271	C57BL/6J and C57BL/6NTac	N/A
	8	37790271	110881875	C57BL/6J and C57BL/6NTac and CBA/J	Heterozygous
	8	110881875	129401213	CBA/J	Homozygous
	9	3000000	21110559	C57BL/6J and C57BL/6NTac and CBA/J	Heterozygous
	9	21110559	37691490	C57BL/6J and C57BL/6NTac	N/A
	9	37691490	109855467	C57BL/6J and C57BL/6NTac and CBA/J	Heterozygous
	9	109855467	115715944	C57BL/6J and C57BL/6NTac	N/A
	9	115715944	124595110	C57BL/6J and C57BL/6NTac and CBA/J	Heterozygous

MiniMUGA Background Analysis v2.3.1

	10	3000000	42858234	C57BL/6J and C57BL/6NTac and CBA/J	Heterozygous
	10	42858234	115781736	CBA/J	Homozygous
	10	115781736	130694993	C57BL/6J and C57BL/6NTac and CBA/J	Heterozygous
	11	3000000	22302070	CBA/J	Homozygous
	11	22302070	49269299	C57BL/6J and C57BL/6NTac and CBA/J	Heterozygous
	11	49269299	65400332	CBA/J	Homozygous
	11	65400332	90803561	C57BL/6J and C57BL/6NTac and CBA/J	Heterozygous
	11	90803561	122082543	C57BL/6J and C57BL/6NTac	N/A
	12	3000000	27585493	C57BL/6J and C57BL/6NTac	N/A
	12	27585493	94246475	C57BL/6J and C57BL/6NTac and CBA/J	Heterozygous
	12	94246475	100284662	CBA/J	Homozygous
	12	100284662	120129022	C57BL/6J and C57BL/6NTac and CBA/J	Heterozygous
	13	3000000	60796031	CBA/J	Homozygous
	13	60796031	67442927	C57BL/6J and C57BL/6NTac and CBA/J	Heterozygous
	13	67442927	97130661	CBA/J	Homozygous
	13	97130661	120421639	C57BL/6J and C57BL/6NTac and CBA/J	Heterozygous
	14	3000000	38092288	CBA/J	Homozygous
	14	38092288	70580779	C57BL/6J and C57BL/6NTac and CBA/J	Heterozygous
	14	70580779	76871639	C57BL/6J and C57BL/6NTac	N/A
	14	76871639	103377147	C57BL/6J and C57BL/6NTac and CBA/J	Heterozygous
	14	103377147	111185375	CBA/J	Homozygous
	14	111185375	124902244	C57BL/6J and C57BL/6NTac and CBA/J	Heterozygous
	15	3000000	36473640	C57BL/6J and C57BL/6NTac and CBA/J	Heterozygous
	15	36473640	44010563	C57BL/6J and C57BL/6NTac	N/A
	15	44010563	55016741	C57BL/6J and C57BL/6NTac and CBA/J	Heterozygous
	15	55016741	70554147	C57BL/6J and C57BL/6NTac	N/A
	15	70554147	88538882	C57BL/6J and C57BL/6NTac and CBA/J	Heterozygous
	15	88538882	104043685	C57BL/6J and C57BL/6NTac	N/A
	16	3000000	20813513	C57BL/6J and C57BL/6NTac and CBA/J	Heterozygous
	16	20813513	29701002	CBA/J	Homozygous
	16	29701002	42525074	C57BL/6J and C57BL/6NTac and CBA/J	Heterozygous
	16	42525074	76315797	C57BL/6J and C57BL/6NTac	N/A

MiniMUGA Background Analysis v2.3.1

	16	76315797	87403166	C57BL/6J and C57BL/6NTac and CBA/J	Heterozygous
	16	87403166	98207768	C57BL/6J and C57BL/6NTac	N/A
	17	3000000	69590784	C57BL/6J and C57BL/6NTac and CBA/J	Heterozygous
	17	69590784	94987271	C57BL/6J and C57BL/6NTac	N/A
	18	3000000	63069205	C57BL/6J and C57BL/6NTac and CBA/J	Heterozygous
	18	63069205	90702639	C57BL/6J and C57BL/6NTac	N/A
	19	3000000	42043276	C57BL/6J and C57BL/6NTac and CBA/J	Heterozygous
	19	42043276	61431566	C57BL/6J and C57BL/6NTac	N/A
	X	3000000	136441962	C57BL/6J and C57BL/6NTac and CBA/J	Heterozygous
	X	136441962	171031299	C57BL/6J and C57BL/6NTac	N/A
	MT	0	0	IBD	Hemizygous
