# Supplementary material for: Deletion of 9p drives B-ALL through heterozygous inactivation of Pax5 and Cd72 in preleukemic cells
Source: JCI Insight. 2026 Feb 17;11(7):e199464. doi: 10.1172/jci.insight.199464 (PMC13134721; doi:10.1172/jci.insight.199464)
Supplement: Supplemental data set 1 [file jciinsight-11-199464-s204.zip › Strain_Genotyping/B273-results-report.pdf]

MiniMUGA Background Analysis v2.3.1

<table>
<tr><th>Sample ID</th><td>B273</td></tr>
<tr><th>Neogen ID</th><td>AAAU-4540</td></tr>
<tr><th>Summary</th><td>The genotype of this sample is of excellent quality. It is female and outbred, and likely a mix of C57BL/6J and C57BL/6NTac and CBA/J. Clustering of unexplained markers is evidence of an additional background strain.

Diagnostic SNPs are likely explained by the presence of the background strains

Solution 1: 129S5/SvEvBrd and C57BL/6J and C57BL/6NRj
C57BL/6J: 97 / 158 (61.4%)
C57BL/6NRj: 10 / 37 (27.0%)
129S5/SvEvBrd: 1 / 5 (20.0%)
Solution 2: 129S5/SvEvBrd and C57BL/6JRj and C57BL/6NRj
C57BL/6JRj: 97 / 158 (61.4%)
C57BL/6NRj: 10 / 37 (27.0%)
129S5/SvEvBrd: 1 / 5 (20.0%)

NOTE: There is a discrepancy between the diagnostic backgrounds detected and the primary and secondary background analysis (CBA/J, C57BL/6J, C57BL/6NTac). This is uncommon and should be investigated further.

No genetic constructs were detected in this sample.

WARNING:

There is a discrepancy between the diagnostic backgrounds detected ((129S5/SvEvBrd and C57BL/6J and C57BL/6NRj) or (129S5/SvEvBrd and C57BL/6JRj and C57BL/6NRj)) and the primary background (C57BL/6J and C57BL/6NTac) and secondary background (CBA/J). This is uncommon and should be investigated further.
The presence of a single diagnostic heterozygous call for a single inbred strain should be treated with caution.
This sample likely has more than 2 genetic backgrounds (unexplained regions and/or fractured ideogram). The strain selected for secondary background may be incorrect. The estimation of the contribution of primary and secondary background are likely incorrect. This can potentially be addressed with input from the user.
</td></tr>
<tr><th>Genotyping Quality</th><td>Excellent (6 N calls)
All reported results are dependent on genotyping quality.</td></tr>
<tr><th>Chromosomal Sex</th><td>XX</td></tr>
<tr><th>Inbreeding Estimate</th><td>41.4% Inbred
(Percentage of the genome (autosomal and X chromosomes) that is homozygous or hemizygous for primary, secondary, and unknown backgrounds. See Genome Analysis)</td></tr>
<tr><th>Constructs Detected</th><td>
<table>
<tr><th>BlastR</th><th>bpA</th><th>Cas9</th><th>chlor</th><th>cHS4</th><th>Cre</th><th>DTA</th><th>Flp</th><th>g_FP</th><th>hCMV_a</th><th>hCMV_b</th><th>hTK_pr</th><th>iCre</th><th>IRES</th><th>Luc</th><th>r_FP</th><th>rtTA</th><th>SV40</th><th>tTA</th></tr>
<tr><td>-</td><td>-</td><td>-</td><td>-</td><td>-</td><td>-</td><td>-</td><td>-</td><td>-</td><td>-</td><td>-</td><td>-</td><td>-</td><td>-</td><td>-</td><td>-</td><td>-</td><td>-</td><td>-</td></tr>
</table></td></tr>
</table>

MiniMUGA Background Analysis v2.3.1

Refined Ideogram

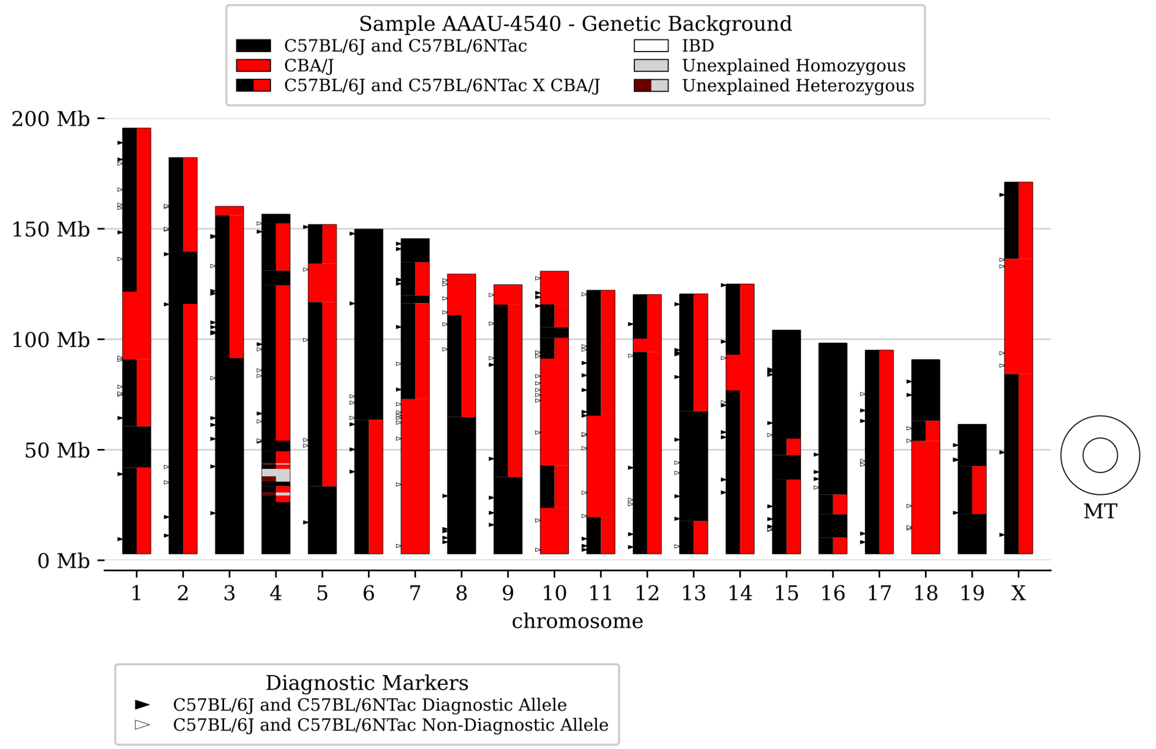

Genome Analysis

Background	Zygosity	Informative Markers	Informative Markers %	Genome %
C57BL/6J and C57BL/6NTac	N/A	681	25.6%	25.5%
CBA/J	Homozygous	455	17.1%	15.7%
C57BL/6J and C57BL/6NTac X CBA/J	Heterozygous	1508	56.7%	58.5%
Unexplained	Homozygous	5	0.2%	0.1%
Unexplained	Heterozygous	10	0.4%	0.2%
	**Total**	2659	100.0%	100.0%

Y Chromosome

Not Applicable

MT Genome

MT Haplogroup 6 - 100.0% Consistent
Includes C57BL/6J, C57BL/6NTac, CBA/J and 165 other strains

MiniMUGA Background Analysis v2.3.1

Backgrounds Detected (Diagnostic Alleles)

Diagnostic Class	Diagnostic Alleles Observed: Homozygous	Heterozygous	Potential	% Observed
C57BL/6J, C57BL/6JJicTac, C57BL/6JRj	11	57	102	66.7%
C57BL/6J, C57BL/6JEiJ, C57BL/6JJicTac, C57BL/6JRj	4	10	21	66.7%
C57BL/6J, C57BL/6JRj	2	10	31	38.7%
C57BL/6NJ, C57BL/6NRj, C57BL/6NTac	0	4	10	40.0%
C57BL/6NRj, C57BL/6NTac	0	4	15	26.7%
129S5/SvEvBrd	0	1	5	20.0%
B6N-Tyr<c-Brd>/BrdCrCrl, C57BL/6J, C57BL/6JBomTac, C57BL/6JEiJ, C57BL/6JJicTac, C57BL/6JOlaHsd, C57BL/6JRj	0	1	2	50.0%
B6N-Tyr<c-Brd>/BrdCrCrl, C57BL/6J, C57BL/6JEiJ, C57BL/6JJicTac, C57BL/6JRj	0	1	1	100.0%
B6N-Tyr<c-Brd>/BrdCrCrl, C57BL/6NCrl, C57BL/6NHsd, C57BL/6NJ, C57BL/6NRj, C57BL/6NTac	0	1	2	50.0%
C57BL/6J, C57BL/6JEiJ, C57BL/6JJicTac, C57BL/6JOlaHsd, C57BL/6JRj	0	1	1	100.0%
C57BL/6NRj	0	1	10	10.0%

Minimal Strain Sets Explaining All Diagnostic Classes (Number of Markers Explained):

- Solution 1: 129S5/SvEvBrd and C57BL/6J and C57BL/6NRj
    - C57BL/6J: 97 / 158 (61.4%)
    - C57BL/6NRj: 10 / 37 (27.0%)
    - 129S5/SvEvBrd: 1 / 5 (20.0%)
- Solution 2: 129S5/SvEvBrd and C57BL/6JRj and C57BL/6NRj
    - C57BL/6JRj: 97 / 158 (61.4%)
    - C57BL/6NRj: 10 / 37 (27.0%)
    - 129S5/SvEvBrd: 1 / 5 (20.0%)

Chromosome	Start (Mb)	Stop (Mb)	Background	Zygosity
1	3000000	41869819	C57BL/6J and C57BL/6NTac and CBA/J	Heterozygous
1	41869819	60621237	C57BL/6J and C57BL/6NTac	N/A
1	60621237	90903197	C57BL/6J and C57BL/6NTac and CBA/J	Heterozygous
1	90903197	121519847	CBA/J	Homozygous
1	121519847	195471971	C57BL/6J and C57BL/6NTac and CBA/J	Heterozygous
2	3000000	115970567	C57BL/6J and C57BL/6NTac and CBA/J	Heterozygous
2	115970567	139631657	C57BL/6J and C57BL/6NTac	N/A
2	139631657	182113224	C57BL/6J and C57BL/6NTac and CBA/J	Heterozygous
3	3000000	91461564	C57BL/6J and C57BL/6NTac	N/A
3	91461564	156090101	C57BL/6J and C57BL/6NTac and CBA/J	Heterozygous
3	156090101	160039680	CBA/J	Homozygous
4	3000000	26280383	C57BL/6J and C57BL/6NTac	N/A

MiniMUGA Background Analysis v2.3.1

**Diplotype Intervals**	4	26280383	29346519	C57BL/6J and C57BL/6NTac and CBA/J	Heterozygous
	4	29346519	30650814	Unexplained	Heterozygous
	4	30650814	33527174	C57BL/6J and C57BL/6NTac and CBA/J	Heterozygous
	4	33527174	35563307	C57BL/6J and C57BL/6NTac	N/A
	4	35563307	37995481	Unexplained	Heterozygous
	4	37995481	41348396	Unexplained	Homozygous
	4	41348396	43372387	C57BL/6J and C57BL/6NTac and CBA/J	Heterozygous
	4	43372387	43819249	Unexplained	Heterozygous
	4	43819249	49280860	C57BL/6J and C57BL/6NTac and CBA/J	Heterozygous
	4	49280860	54114833	C57BL/6J and C57BL/6NTac	N/A
	4	54114833	124400069	C57BL/6J and C57BL/6NTac and CBA/J	Heterozygous
	4	124400069	131104093	C57BL/6J and C57BL/6NTac	N/A
	4	131104093	152440879	C57BL/6J and C57BL/6NTac and CBA/J	Heterozygous
	4	152440879	156508116	C57BL/6J and C57BL/6NTac	N/A
	5	3000000	33482235	C57BL/6J and C57BL/6NTac	N/A
	5	33482235	116795433	C57BL/6J and C57BL/6NTac and CBA/J	Heterozygous
	5	116795433	134172373	CBA/J	Homozygous
	5	134172373	151834684	C57BL/6J and C57BL/6NTac and CBA/J	Heterozygous
	6	3000000	63548966	C57BL/6J and C57BL/6NTac and CBA/J	Heterozygous
	6	63548966	149736546	C57BL/6J and C57BL/6NTac	N/A
	7	3000000	72944748	CBA/J	Homozygous
	7	72944748	116328796	C57BL/6J and C57BL/6NTac and CBA/J	Heterozygous
	7	116328796	119823617	C57BL/6J and C57BL/6NTac	N/A
	7	119823617	134805535	C57BL/6J and C57BL/6NTac and CBA/J	Heterozygous
	7	134805535	145441459	C57BL/6J and C57BL/6NTac	N/A
	8	3000000	64818329	C57BL/6J and C57BL/6NTac	N/A
	8	64818329	110881875	C57BL/6J and C57BL/6NTac and CBA/J	Heterozygous
	8	110881875	129401213	CBA/J	Homozygous
	9	3000000	37691490	C57BL/6J and C57BL/6NTac	N/A
	9	37691490	115715944	C57BL/6J and C57BL/6NTac and CBA/J	Heterozygous
	9	115715944	124595110	CBA/J	Homozygous
	10	3000000	23654421	CBA/J	Homozygous

MiniMUGA Background Analysis v2.3.1

10	23654421	42917049	C57BL/6J and C57BL/6NTac and CBA/J	Heterozygous
10	42917049	91235291	CBA/J	Homozygous
10	91235291	100561092	C57BL/6J and C57BL/6NTac and CBA/J	Heterozygous
10	100561092	105450288	C57BL/6J and C57BL/6NTac	N/A
10	105450288	115781736	C57BL/6J and C57BL/6NTac and CBA/J	Heterozygous
10	115781736	130694993	CBA/J	Homozygous
11	3000000	19463075	C57BL/6J and C57BL/6NTac and CBA/J	Heterozygous
11	19463075	65400332	CBA/J	Homozygous
11	65400332	122082543	C57BL/6J and C57BL/6NTac and CBA/J	Heterozygous
12	3000000	94246475	C57BL/6J and C57BL/6NTac and CBA/J	Heterozygous
12	94246475	100284662	CBA/J	Homozygous
12	100284662	120129022	C57BL/6J and C57BL/6NTac and CBA/J	Heterozygous
13	3000000	17741397	C57BL/6J and C57BL/6NTac and CBA/J	Heterozygous
13	17741397	67442927	C57BL/6J and C57BL/6NTac	N/A
13	67442927	120421639	C57BL/6J and C57BL/6NTac and CBA/J	Heterozygous
14	3000000	76871639	C57BL/6J and C57BL/6NTac and CBA/J	Heterozygous
14	76871639	93002544	CBA/J	Homozygous
14	93002544	124902244	C57BL/6J and C57BL/6NTac and CBA/J	Heterozygous
15	3000000	36473640	C57BL/6J and C57BL/6NTac and CBA/J	Heterozygous
15	36473640	47626553	C57BL/6J and C57BL/6NTac	N/A
15	47626553	55016741	C57BL/6J and C57BL/6NTac and CBA/J	Heterozygous
15	55016741	104043685	C57BL/6J and C57BL/6NTac	N/A
16	3000000	10284757	C57BL/6J and C57BL/6NTac and CBA/J	Heterozygous
16	10284757	20813513	C57BL/6J and C57BL/6NTac	N/A
16	20813513	29701002	C57BL/6J and C57BL/6NTac and CBA/J	Heterozygous
16	29701002	98207768	C57BL/6J and C57BL/6NTac	N/A
17	3000000	94987271	C57BL/6J and C57BL/6NTac and CBA/J	Heterozygous
18	3000000	54023745	CBA/J	Homozygous
18	54023745	63069205	C57BL/6J and C57BL/6NTac and CBA/J	Heterozygous
18	63069205	90702639	C57BL/6J and C57BL/6NTac	N/A
19	3000000	20955280	C57BL/6J and C57BL/6NTac	N/A

MiniMUGA Background Analysis v2.3.1

	19	20955280	42582533	C57BL/6J and C57BL/6NTac and CBA/J	Heterozygous
	19	42582533	61431566	C57BL/6J and C57BL/6NTac	N/A
	X	3000000	84237192	C57BL/6J and C57BL/6NTac and CBA/J	Heterozygous
	X	84237192	136441962	CBA/J	Homozygous
	X	136441962	171031299	C57BL/6J and C57BL/6NTac and CBA/J	Heterozygous
	MT	0	0	IBD	Hemizygous
