# Supplementary material for: Deletion of 9p drives B-ALL through heterozygous inactivation of Pax5 and Cd72 in preleukemic cells
Source: JCI Insight. 2026 Feb 17;11(7):e199464. doi: 10.1172/jci.insight.199464 (PMC13134721; doi:10.1172/jci.insight.199464)
Supplement: Supplemental data set 1 [file jciinsight-11-199464-s204.zip › Strain_Genotyping/B397-results-report.pdf]

MiniMUGA Background Analysis v2.3.1

**Sample ID**	B397
**Neogen ID**	AAAU-4536
**Summary**	The genotype of this sample is of **excellent** quality. It is **female** and **outbred**, and likely a mix of **C57BL/6J and C57BL/6NTac** and **CBA/J**. Clustering of unexplained markers is evidence of an additional background strain.<br><br>Diagnostic SNPs are likely explained by the presence of the background strains<br>• Solution 1: 129S5/SvEvBrd and C57BL/6J and C57BL/6NRj<br>◦ C57BL/6J: 83 / 160 (51.9%)<br>◦ C57BL/6NRj: 9 / 35 (25.7%)<br>◦ 129S5/SvEvBrd: 1 / 5 (20.0%)<br>• Solution 2: 129S5/SvEvBrd and C57BL/6JRj and C57BL/6NRj<br>◦ C57BL/6JRj: 83 / 160 (51.9%)<br>◦ C57BL/6NRj: 9 / 35 (25.7%)<br>◦ 129S5/SvEvBrd: 1 / 5 (20.0%)<br><br>NOTE: There is a discrepancy between the diagnostic backgrounds detected and the primary and secondary background analysis (CBA/J, C57BL/6J, C57BL/6NTac). This is uncommon and should be investigated further.<br><br>No genetic constructs were detected in this sample.<br><br>WARNING:<br>• There is a discrepancy between the diagnostic backgrounds detected ((129S5/SvEvBrd and C57BL/6J and C57BL/6NRj) or (129S5/SvEvBrd and C57BL/6JRj and C57BL/6NRj)) and the primary background (C57BL/6J and C57BL/6NTac) and secondary background (CBA/J). This is uncommon and should be investigated further.<br>• The presence of a single diagnostic heterozygous call for a single inbred strain should be treated with caution.<br>• This sample likely has more than 2 genetic backgrounds (unexplained regions and/or fractured ideogram). The strain selected for secondary background may be incorrect. The estimation of the contribution of primary and secondary background are likely incorrect. This can potentially be addressed with input from the user.
**Genotyping Quality**	**Excellent (15 N calls)**<br>All reported results are dependent on genotyping quality.
**Chromosomal Sex**	XX
**Inbreeding Estimate**	40.9% Inbred<br>(Percentage of the genome (autosomal and X chromosomes) that is homozygous or hemizygous for primary, secondary, and unknown backgrounds. See Genome Analysis)

Constructs Detected

BlastR	bpA	Cas9	chlor	cHS4	Cre	DTA	Flp	g_FP	hCMV_a	hCMV_b	hTK_pr	iCre	IRES	Luc	r_FP	rtTA	SV40	tTA
-	-	-	-	-	-	-	-	-	-	-	-	-	-	-	-	-	-	-

MiniMUGA Background Analysis v2.3.1

Refined Ideogram

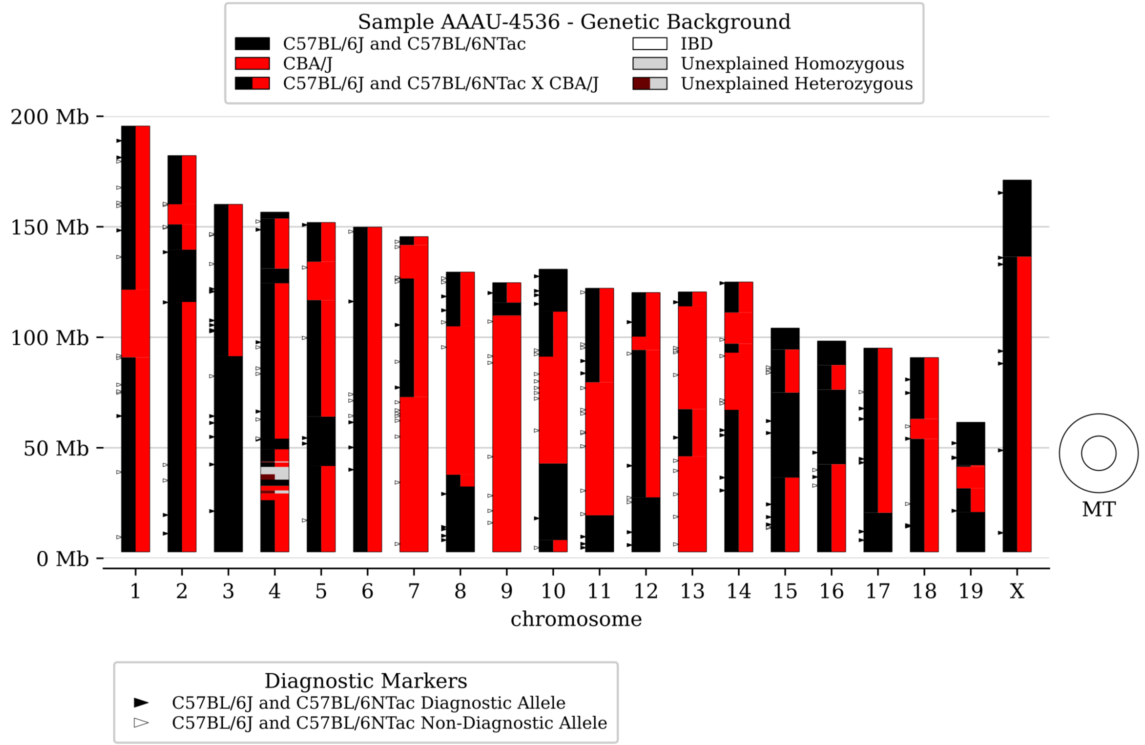

Genome Analysis

Background	Zygosity	Informative Markers	Informative Markers %	Genome %
C57BL/6J and C57BL/6NTac	N/A	523	19.7%	18.0%
CBA/J	Homozygous	551	20.8%	22.7%
C57BL/6J and C57BL/6NTac X CBA/J	Heterozygous	1566	59.0%	59.0%
Unexplained	Homozygous	5	0.2%	0.1%
Unexplained	Heterozygous	10	0.4%	0.2%
	**Total**	2655	100.1%	100.0%

Y Chromosome

Not Applicable

MT Genome

MT Haplogroup 6 - 100.0% Consistent
Includes C57BL/6J, C57BL/6NTac, CBA/J and 165 other strains

MiniMUGA Background Analysis v2.3.1

Backgrounds Detected (Diagnostic Alleles)

	Diagnostic Alleles Observed			
**Diagnostic Class**	**Homozygous**	**Heterozygous**	**Potential**	**% Observed**
C57BL/6J, C57BL/6JJicTac, C57BL/6JRj	11	45	102	54.9%
C57BL/6J, C57BL/6JRj	3	10	31	41.9%
C57BL/6J, C57BL/6JEiJ, C57BL/6JJicTac, C57BL/6JRj	3	9	21	57.1%
C57BL/6NJ, C57BL/6NRj, C57BL/6NTac	0	4	10	40.0%
C57BL/6NRj, C57BL/6NTac	0	4	15	26.7%
129S5/SvEvBrd	0	1	5	20.0%
B6N-Tyr<c-Brd>/BrdCrCrl, C57BL/6J, C57BL/6JEiJ, C57BL/6JJicTac, C57BL/6JRj	0	1	1	100.0%
B6N-Tyr<c-Brd>/BrdCrCrl, C57BL/6J, C57BL/6JJicTac, C57BL/6JRj	0	1	5	20.0%
C57BL/6NRj	0	1	10	10.0%

Minimal Strain Sets Explaining All Diagnostic Classes (Number of Markers Explained):

- Solution 1: 129S5/SvEvBrd and C57BL/6J and C57BL/6NRj
  - C57BL/6J: 83 / 160 (51.9%)
  - C57BL/6NRj: 9 / 35 (25.7%)
  - 129S5/SvEvBrd: 1 / 5 (20.0%)
- Solution 2: 129S5/SvEvBrd and C57BL/6JRj and C57BL/6NRj
  - C57BL/6JRj: 83 / 160 (51.9%)
  - C57BL/6NRj: 9 / 35 (25.7%)
  - 129S5/SvEvBrd: 1 / 5 (20.0%)

Diplotype Intervals

Chromosome	Start (Mb)	Stop (Mb)	Background	Zygosity
1	3000000	90903197	C57BL/6J and C57BL/6NTac and CBA/J	Heterozygous
1	90903197	121519847	CBA/J	Homozygous
1	121519847	195471971	C57BL/6J and C57BL/6NTac and CBA/J	Heterozygous
2	3000000	115970567	C57BL/6J and C57BL/6NTac and CBA/J	Heterozygous
2	115970567	139631657	C57BL/6J and C57BL/6NTac	N/A
2	139631657	151062687	C57BL/6J and C57BL/6NTac and CBA/J	Heterozygous
2	151062687	160174252	CBA/J	Homozygous
2	160174252	182113224	C57BL/6J and C57BL/6NTac and CBA/J	Heterozygous
3	3000000	91461564	C57BL/6J and C57BL/6NTac	N/A
3	91461564	160039680	C57BL/6J and C57BL/6NTac and CBA/J	Heterozygous
4	3000000	26280383	C57BL/6J and C57BL/6NTac and CBA/J	Heterozygous
4	26280383	29346519	CBA/J	Homozygous
4	29346519	30650814	Unexplained	Heterozygous
4	30650814	32777492	CBA/J	Homozygous
4	32777492	35563307	C57BL/6J and C57BL/6NTac	N/A
4	35563307	37995481	Unexplained	Heterozygous

MiniMUGA Background Analysis v2.3.1

4	37995481	41348396	Unexplained	Homozygous
4	41348396	43372387	C57BL/6J and C57BL/6NTac and CBA/J	Heterozygous
4	43372387	43819249	Unexplained	Heterozygous
4	43819249	49280860	C57BL/6J and C57BL/6NTac and CBA/J	Heterozygous
4	49280860	54114833	C57BL/6J and C57BL/6NTac	N/A
4	54114833	124400069	C57BL/6J and C57BL/6NTac and CBA/J	Heterozygous
4	124400069	131104093	C57BL/6J and C57BL/6NTac	N/A
4	131104093	153688585	C57BL/6J and C57BL/6NTac and CBA/J	Heterozygous
4	153688585	156508116	C57BL/6J and C57BL/6NTac	N/A
5	3000000	41755530	C57BL/6J and C57BL/6NTac and CBA/J	Heterozygous
5	41755530	64102638	C57BL/6J and C57BL/6NTac	N/A
5	64102638	116795433	C57BL/6J and C57BL/6NTac and CBA/J	Heterozygous
5	116795433	134172373	CBA/J	Homozygous
5	134172373	151834684	C57BL/6J and C57BL/6NTac and CBA/J	Heterozygous
6	3000000	149736546	C57BL/6J and C57BL/6NTac and CBA/J	Heterozygous
7	3000000	72944748	CBA/J	Homozygous
7	72944748	126580094	C57BL/6J and C57BL/6NTac and CBA/J	Heterozygous
7	126580094	141750158	CBA/J	Homozygous
7	141750158	145441459	C57BL/6J and C57BL/6NTac and CBA/J	Heterozygous
8	3000000	32467133	C57BL/6J and C57BL/6NTac	N/A
8	32467133	37790271	C57BL/6J and C57BL/6NTac and CBA/J	Heterozygous
8	37790271	104937322	CBA/J	Homozygous
8	104937322	129401213	C57BL/6J and C57BL/6NTac and CBA/J	Heterozygous
9	3000000	109855467	CBA/J	Homozygous
9	109855467	115715944	C57BL/6J and C57BL/6NTac	N/A
9	115715944	124595110	C57BL/6J and C57BL/6NTac and CBA/J	Heterozygous
10	3000000	8205640	C57BL/6J and C57BL/6NTac and CBA/J	Heterozygous
10	8205640	42917049	C57BL/6J and C57BL/6NTac	N/A
10	42917049	91235291	CBA/J	Homozygous
10	91235291	111566142	C57BL/6J and C57BL/6NTac and CBA/J	Heterozygous
10	111566142	130694993	C57BL/6J and C57BL/6NTac	N/A
11	3000000	19463075	C57BL/6J and C57BL/6NTac	N/A

MiniMUGA Background Analysis v2.3.1

11	19463075	79617327	CBA/J	Homozygous
11	79617327	122082543	C57BL/6J and C57BL/6NTac and CBA/J	Heterozygous
12	3000000	27585493	C57BL/6J and C57BL/6NTac	N/A
12	27585493	94246475	C57BL/6J and C57BL/6NTac and CBA/J	Heterozygous
12	94246475	100284662	CBA/J	Homozygous
12	100284662	120129022	C57BL/6J and C57BL/6NTac and CBA/J	Heterozygous
13	3000000	46136691	CBA/J	Homozygous
13	46136691	67442927	C57BL/6J and C57BL/6NTac and CBA/J	Heterozygous
13	67442927	113994455	CBA/J	Homozygous
13	113994455	120421639	C57BL/6J and C57BL/6NTac and CBA/J	Heterozygous
14	3000000	67152629	C57BL/6J and C57BL/6NTac and CBA/J	Heterozygous
14	67152629	93002544	CBA/J	Homozygous
14	93002544	97106405	C57BL/6J and C57BL/6NTac and CBA/J	Heterozygous
14	97106405	111185375	CBA/J	Homozygous
14	111185375	124902244	C57BL/6J and C57BL/6NTac and CBA/J	Heterozygous
15	3000000	36473640	C57BL/6J and C57BL/6NTac and CBA/J	Heterozygous
15	36473640	74996398	C57BL/6J and C57BL/6NTac	N/A
15	74996398	94412127	C57BL/6J and C57BL/6NTac and CBA/J	Heterozygous
15	94412127	104043685	C57BL/6J and C57BL/6NTac	N/A
16	3000000	42525074	C57BL/6J and C57BL/6NTac and CBA/J	Heterozygous
16	42525074	76315797	C57BL/6J and C57BL/6NTac	N/A
16	76315797	87403166	C57BL/6J and C57BL/6NTac and CBA/J	Heterozygous
16	87403166	98207768	C57BL/6J and C57BL/6NTac	N/A
17	3000000	20616647	C57BL/6J and C57BL/6NTac	N/A
17	20616647	94987271	C57BL/6J and C57BL/6NTac and CBA/J	Heterozygous
18	3000000	54023745	C57BL/6J and C57BL/6NTac and CBA/J	Heterozygous
18	54023745	63069205	CBA/J	Homozygous
18	63069205	90702639	C57BL/6J and C57BL/6NTac and CBA/J	Heterozygous
19	3000000	20955280	C57BL/6J and C57BL/6NTac	N/A
19	20955280	31636352	C57BL/6J and C57BL/6NTac and CBA/J	Heterozygous
19	31636352	41403951	CBA/J	Homozygous
19	41403951	42043276	C57BL/6J and C57BL/6NTac and CBA/J	Heterozygous

MiniMUGA Background Analysis v2.3.1

	19	42043276	61431566	C57BL/6J and C57BL/6NTac	N/A
	X	3000000	136441962	C57BL/6J and C57BL/6NTac and CBA/J	Heterozygous
	X	136441962	171031299	C57BL/6J and C57BL/6NTac	N/A
	MT	0	0	IBD	Hemizygous
