# Supplementary material for: Deletion of 9p drives B-ALL through heterozygous inactivation of Pax5 and Cd72 in preleukemic cells
Source: JCI Insight. 2026 Feb 17;11(7):e199464. doi: 10.1172/jci.insight.199464 (PMC13134721; doi:10.1172/jci.insight.199464)
Supplement: Supplemental data set 1 [file jciinsight-11-199464-s204.zip › Strain_Genotyping/B398-results-report.pdf]

# MiniMUGA Background Analysis v2.3.1

<table>
<tr><th>Sample ID</th><td>B398</td></tr>
<tr><th>Neogen ID</th><td>AAAU-4537</td></tr>
<tr><th>Summary</th><td>The genotype of this sample is of excellent quality. It is female and outbred, and likely a mix of C57BL/6J and C57BL/6NTac and CBA/J. Clustering of unexplained markers is evidence of an additional background strain.<br><br>Diagnostic SNPs are likely explained by the presence of the background strains<ul><li>Solution 1: 129S5/SvEvBrd and C57BL/6J and C57BL/6NRj<ul><li>C57BL/6J: 94 / 163 (57.7%)</li><li>C57BL/6NRj: 7 / 35 (20.0%)</li><li>129S5/SvEvBrd: 1 / 5 (20.0%)</li></ul></li><li>Solution 2: 129S5/SvEvBrd and C57BL/6JRj and C57BL/6NRj<ul><li>C57BL/6JRj: 94 / 163 (57.7%)</li><li>C57BL/6NRj: 7 / 35 (20.0%)</li><li>129S5/SvEvBrd: 1 / 5 (20.0%)</li></ul></li></ul><br>NOTE: There is a discrepancy between the diagnostic backgrounds detected and the primary and secondary background analysis (CBA/J, C57BL/6J, C57BL/6NTac). This is uncommon and should be investigated further.<br><br>The sample may contain the following genetic constructs: bpA<br><br>WARNING:<ul><li>There is a discrepancy between the diagnostic backgrounds detected ((129S5/SvEvBrd and C57BL/6J and C57BL/6NRj) or (129S5/SvEvBrd and C57BL/6JRj and C57BL/6NRj)) and the primary background (C57BL/6J and C57BL/6NTac) and secondary background (CBA/J). This is uncommon and should be investigated further.</li><li>The presence of a single diagnostic heterozygous call for a single inbred strain should be treated with caution.</li><li>This sample likely has more than 2 genetic backgrounds (unexplained regions and/or fractured ideogram). The strain selected for secondary background may be incorrect. The estimation of the contribution of primary and secondary background are likely incorrect. This can potentially be addressed with input from the user.</li></ul></td></tr>
<tr><th>Genotyping Quality</th><td>Excellent (60 N calls)<br>All reported results are dependent on genotyping quality.</td></tr>
<tr><th>Chromosomal Sex</th><td>XX</td></tr>
<tr><th>Inbreeding Estimate</th><td>41.4% Inbred<br>(Percentage of the genome (autosomal and X chromosomes) that is homozygous or hemizygous for primary, secondary, and unknown backgrounds. See Genome Analysis)</td></tr>
<tr><th>Constructs Detected</th><td>

| BlastR | bpA | Cas9 | chlor | cHS4 | Cre | DTA | Flp | g_FP | hCMV_a | hCMV_b | hTK_pr | iCre | IRES | Luc | r_FP | rtTA | SV40 | tTA |
|---|---|---|---|---|---|---|---|---|---|---|---|---|---|---|---|---|---|---|
| - | ?* | - | - | - | - | - | - | - | - | - | - | - | - | - | - | - | - | - |

* This sample has abnormal raw intensity values. Therefore, positive or questionable returns for constructs should be treated with caution.</td></tr>
</table>

# MiniMUGA Background Analysis v2.3.1

| | |
|---|---|
| **Refined Ideogram** | 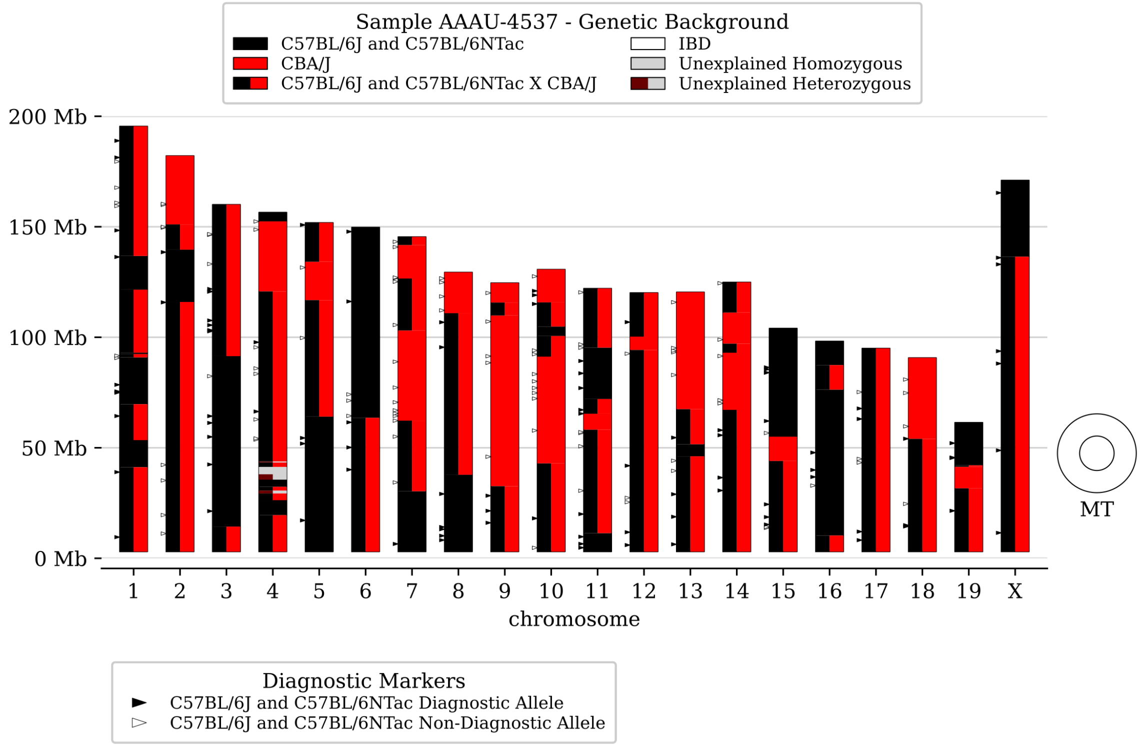 |



**Genome Analysis**

| Background | Zygosity | Informative Markers | Informative Markers % | Genome % |
|---|---|---|---|---|
| C57BL/6J and C57BL/6NTac | N/A | 656 | 24.9% | 23.1% |
| CBA/J | Homozygous | 476 | 18.1% | 18.2% |
| C57BL/6J and C57BL/6NTac X CBA/J | Heterozygous | 1481 | 56.3% | 58.4% |
| Unexplained | Homozygous | 5 | 0.2% | 0.1% |
| Unexplained | Heterozygous | 13 | 0.5% | 0.2% |
| | **Total** | 2631 | 100.0% | 100.0% |

| | |
|---|---|
| **Y Chromosome** | Not Applicable |
| **MT Genome** | MT Haplogroup 6 - 100.0% Consistent<br>Includes C57BL/6J, C57BL/6NTac, CBA/J and 165 other strains |

# MiniMUGA Background Analysis v2.3.1

**Backgrounds Detected (Diagnostic Alleles)**

| Diagnostic Class | Diagnostic Alleles Observed: Homozygous | Heterozygous | Potential | % Observed |
|---|---|---|---|---|
| C57BL/6J, C57BL/6JJicTac, C57BL/6JRj | 8 | 55 | 102 | 61.8% |
| C57BL/6J, C57BL/6JEiJ, C57BL/6JJicTac, C57BL/6JRj | 4 | 10 | 21 | 66.7% |
| C57BL/6J, C57BL/6JRj | 1 | 12 | 31 | 41.9% |
| C57BL/6NJ, C57BL/6NRj, C57BL/6NTac | 0 | 3 | 10 | 30.0% |
| C57BL/6NRj, C57BL/6NTac | 0 | 3 | 15 | 20.0% |
| 129S5/SvEvBrd | 0 | 1 | 5 | 20.0% |
| B6N-Tyr<c-Brd>/BrdCrCrl, C57BL/6J, C57BL/6JEiJ, C57BL/6JJicTac, C57BL/6JRj | 0 | 1 | 1 | 100.0% |
| B6N-Tyr<c-Brd>/BrdCrCrl, C57BL/6J, C57BL/6JJicTac, C57BL/6JRj | 0 | 1 | 5 | 20.0% |
| C57BL/6J, C57BL/6JBomTac, C57BL/6JEiJ, C57BL/6JJicTac, C57BL/6JOlaHsd, C57BL/6JRj | 0 | 1 | 2 | 50.0% |
| C57BL/6J, C57BL/6JEiJ, C57BL/6JJicTac, C57BL/6JOlaHsd, C57BL/6JRj | 0 | 1 | 1 | 100.0% |
| C57BL/6NRj | 0 | 1 | 10 | 10.0% |

**Minimal Strain Sets Explaining All Diagnostic Classes (Number of Markers Explained):**

- Solution 1: 129S5/SvEvBrd and C57BL/6J and C57BL/6NRj
    - C57BL/6J: 94 / 163 (57.7%)
    - C57BL/6NRj: 7 / 35 (20.0%)
    - 129S5/SvEvBrd: 1 / 5 (20.0%)
- Solution 2: 129S5/SvEvBrd and C57BL/6JRj and C57BL/6NRj
    - C57BL/6JRj: 94 / 163 (57.7%)
    - C57BL/6NRj: 7 / 35 (20.0%)
    - 129S5/SvEvBrd: 1 / 5 (20.0%)

| Chromosome | Start (Mb) | Stop (Mb) | Background | Zygosity |
|---|---|---|---|---|
| 1 | 3000000 | 41199760 | C57BL/6J and C57BL/6NTac and CBA/J | Heterozygous |
| 1 | 41199760 | 53457225 | C57BL/6J and C57BL/6NTac | N/A |
| 1 | 53457225 | 69700765 | C57BL/6J and C57BL/6NTac and CBA/J | Heterozygous |
| 1 | 69700765 | 90903197 | C57BL/6J and C57BL/6NTac | N/A |
| 1 | 90903197 | 92392684 | C57BL/6J and C57BL/6NTac and CBA/J | Heterozygous |
| 1 | 92392684 | 93042662 | C57BL/6J and C57BL/6NTac | N/A |
| 1 | 93042662 | 121519847 | C57BL/6J and C57BL/6NTac and CBA/J | Heterozygous |
| 1 | 121519847 | 136798402 | C57BL/6J and C57BL/6NTac | N/A |
| 1 | 136798402 | 195471971 | C57BL/6J and C57BL/6NTac and CBA/J | Heterozygous |
| 2 | 3000000 | 115970567 | C57BL/6J and C57BL/6NTac and CBA/J | Heterozygous |
| 2 | 115970567 | 139631657 | C57BL/6J and C57BL/6NTac | N/A |
| 2 | 139631657 | 151062687 | C57BL/6J and C57BL/6NTac and CBA/J | Heterozygous |

# MiniMUGA Background Analysis v2.3.1

**Diplotype Intervals**

| | | | | |
|---|---|---|---|---|
| 2 | 151062687 | 182113224 | CBA/J | Homozygous |
| 3 | 3000000 | 14328941 | C57BL/6J and C57BL/6NTac and CBA/J | Heterozygous |
| 3 | 14328941 | 91461564 | C57BL/6J and C57BL/6NTac | N/A |
| 3 | 91461564 | 160039680 | C57BL/6J and C57BL/6NTac and CBA/J | Heterozygous |
| 4 | 3000000 | 19515600 | C57BL/6J and C57BL/6NTac and CBA/J | Heterozygous |
| 4 | 19515600 | 26280383 | C57BL/6J and C57BL/6NTac | N/A |
| 4 | 26280383 | 29346519 | C57BL/6J and C57BL/6NTac and CBA/J | Heterozygous |
| 4 | 29346519 | 30650814 | Unexplained | Heterozygous |
| 4 | 30650814 | 32327128 | C57BL/6J and C57BL/6NTac and CBA/J | Heterozygous |
| 4 | 32327128 | 35563307 | C57BL/6J and C57BL/6NTac | N/A |
| 4 | 35563307 | 37995481 | Unexplained | Heterozygous |
| 4 | 37995481 | 41348396 | Unexplained | Homozygous |
| 4 | 41348396 | 43372387 | C57BL/6J and C57BL/6NTac and CBA/J | Heterozygous |
| 4 | 43372387 | 43819249 | Unexplained | Heterozygous |
| 4 | 43819249 | 120738488 | C57BL/6J and C57BL/6NTac and CBA/J | Heterozygous |
| 4 | 120738488 | 152440879 | CBA/J | Homozygous |
| 4 | 152440879 | 156508116 | C57BL/6J and C57BL/6NTac | N/A |
| 5 | 3000000 | 64102638 | C57BL/6J and C57BL/6NTac | N/A |
| 5 | 64102638 | 116795433 | C57BL/6J and C57BL/6NTac and CBA/J | Heterozygous |
| 5 | 116795433 | 134172373 | CBA/J | Homozygous |
| 5 | 134172373 | 151834684 | C57BL/6J and C57BL/6NTac and CBA/J | Heterozygous |
| 6 | 3000000 | 63548966 | C57BL/6J and C57BL/6NTac and CBA/J | Heterozygous |
| 6 | 63548966 | 149736546 | C57BL/6J and C57BL/6NTac | N/A |
| 7 | 3000000 | 30335112 | C57BL/6J and C57BL/6NTac | N/A |
| 7 | 30335112 | 62277367 | C57BL/6J and C57BL/6NTac and CBA/J | Heterozygous |
| 7 | 62277367 | 103084424 | CBA/J | Homozygous |
| 7 | 103084424 | 126580094 | C57BL/6J and C57BL/6NTac and CBA/J | Heterozygous |
| 7 | 126580094 | 141750158 | CBA/J | Homozygous |
| 7 | 141750158 | 145441459 | C57BL/6J and C57BL/6NTac and CBA/J | Heterozygous |
| 8 | 3000000 | 37790271 | C57BL/6J and C57BL/6NTac | N/A |
| 8 | 37790271 | 110881875 | C57BL/6J and C57BL/6NTac and CBA/J | Heterozygous |
| 8 | 110881875 | 129401213 | CBA/J | Homozygous |

# MiniMUGA Background Analysis v2.3.1

| | | | | |
|---|---|---|---|---|
| 9 | 3000000 | 32605981 | C57BL/6J and C57BL/6NTac and CBA/J | Heterozygous |
| 9 | 32605981 | 109855467 | CBA/J | Homozygous |
| 9 | 109855467 | 115715944 | C57BL/6J and C57BL/6NTac and CBA/J | Heterozygous |
| 9 | 115715944 | 124595110 | CBA/J | Homozygous |
| 10 | 3000000 | 42917049 | C57BL/6J and C57BL/6NTac and CBA/J | Heterozygous |
| 10 | 42917049 | 91235291 | CBA/J | Homozygous |
| 10 | 91235291 | 100561092 | C57BL/6J and C57BL/6NTac and CBA/J | Heterozygous |
| 10 | 100561092 | 104861956 | C57BL/6J and C57BL/6NTac | N/A |
| 10 | 104861956 | 115781736 | C57BL/6J and C57BL/6NTac and CBA/J | Heterozygous |
| 10 | 115781736 | 130694993 | CBA/J | Homozygous |
| 11 | 3000000 | 11383915 | C57BL/6J and C57BL/6NTac | N/A |
| 11 | 11383915 | 58168384 | C57BL/6J and C57BL/6NTac and CBA/J | Heterozygous |
| 11 | 58168384 | 65400332 | CBA/J | Homozygous |
| 11 | 65400332 | 72044583 | C57BL/6J and C57BL/6NTac and CBA/J | Heterozygous |
| 11 | 72044583 | 95314593 | C57BL/6J and C57BL/6NTac | N/A |
| 11 | 95314593 | 122082543 | C57BL/6J and C57BL/6NTac and CBA/J | Heterozygous |
| 12 | 3000000 | 94246475 | C57BL/6J and C57BL/6NTac and CBA/J | Heterozygous |
| 12 | 94246475 | 100284662 | CBA/J | Homozygous |
| 12 | 100284662 | 120129022 | C57BL/6J and C57BL/6NTac and CBA/J | Heterozygous |
| 13 | 3000000 | 46136691 | C57BL/6J and C57BL/6NTac and CBA/J | Heterozygous |
| 13 | 46136691 | 51605798 | C57BL/6J and C57BL/6NTac | N/A |
| 13 | 51605798 | 67442927 | C57BL/6J and C57BL/6NTac and CBA/J | Heterozygous |
| 13 | 67442927 | 120421639 | CBA/J | Homozygous |
| 14 | 3000000 | 67152629 | C57BL/6J and C57BL/6NTac and CBA/J | Heterozygous |
| 14 | 67152629 | 93002544 | CBA/J | Homozygous |
| 14 | 93002544 | 97106405 | C57BL/6J and C57BL/6NTac and CBA/J | Heterozygous |
| 14 | 97106405 | 111185375 | CBA/J | Homozygous |
| 14 | 111185375 | 124902244 | C57BL/6J and C57BL/6NTac and CBA/J | Heterozygous |
| 15 | 3000000 | 44010563 | C57BL/6J and C57BL/6NTac and CBA/J | Heterozygous |
| 15 | 44010563 | 55016741 | CBA/J | Homozygous |
| 15 | 55016741 | 104043685 | C57BL/6J and C57BL/6NTac | N/A |
| 16 | 3000000 | 10284757 | C57BL/6J and C57BL/6NTac and CBA/J | Heterozygous |

# MiniMUGA Background Analysis v2.3.1

| | | | | | |
|---|---|---|---|---|---|
| | 16 | 10284757 | 76315797 | C57BL/6J and C57BL/6NTac | N/A |
| | 16 | 76315797 | 87403166 | C57BL/6J and C57BL/6NTac and CBA/J | Heterozygous |
| | 16 | 87403166 | 98207768 | C57BL/6J and C57BL/6NTac | N/A |
| | 17 | 3000000 | 94987271 | C57BL/6J and C57BL/6NTac and CBA/J | Heterozygous |
| | 18 | 3000000 | 54023745 | C57BL/6J and C57BL/6NTac and CBA/J | Heterozygous |
| | 18 | 54023745 | 90702639 | CBA/J | Homozygous |
| | 19 | 3000000 | 31636352 | C57BL/6J and C57BL/6NTac and CBA/J | Heterozygous |
| | 19 | 31636352 | 41403951 | CBA/J | Homozygous |
| | 19 | 41403951 | 42043276 | C57BL/6J and C57BL/6NTac and CBA/J | Heterozygous |
| | 19 | 42043276 | 61431566 | C57BL/6J and C57BL/6NTac | N/A |
| | X | 3000000 | 136441962 | C57BL/6J and C57BL/6NTac and CBA/J | Heterozygous |
| | X | 136441962 | 171031299 | C57BL/6J and C57BL/6NTac | N/A |
| | MT | 0 | 0 | IBD | Hemizygous |
