# Supplementary material for: Deletion of 9p drives B-ALL through heterozygous inactivation of Pax5 and Cd72 in preleukemic cells
Source: JCI Insight. 2026 Feb 17;11(7):e199464. doi: 10.1172/jci.insight.199464 (PMC13134721; doi:10.1172/jci.insight.199464)
Supplement: Supplemental data set 1 [file jciinsight-11-199464-s204.zip › Strain_Genotyping/B531-results-report.pdf]

MiniMUGA Background Analysis v2.3.1

<table>
<tr><th>Sample ID</th><td>B531</td></tr>
<tr><th>Neogen ID</th><td>AAAU-4499</td></tr>
<tr><th>Summary</th><td>The genotype of this sample is of excellent quality. It is XO and outbred, and likely a mix of C57BL/6J and C57BL/6NTac and CBA/J. Clustering of unexplained markers is evidence of an additional background strain.

Diagnostic SNPs are likely explained by the presence of the background strains
Solution 1: C57BL/6J and C57BL/6NTac
C57BL/6J: 62 / 161 (38.5%)
C57BL/6NTac: 20 / 30 (66.7%)
Solution 2: C57BL/6J and C57BL/6NRj
C57BL/6J: 62 / 161 (38.5%)
C57BL/6NRj: 20 / 30 (66.7%)
Solution 3: C57BL/6JRj and C57BL/6NTac
C57BL/6JRj: 62 / 161 (38.5%)
C57BL/6NTac: 20 / 30 (66.7%)
Solution 4: C57BL/6JRj and C57BL/6NRj
C57BL/6JRj: 62 / 161 (38.5%)
C57BL/6NRj: 20 / 30 (66.7%)
No genetic constructs were detected in this sample.

WARNING:
This sample likely has more than 2 genetic backgrounds (unexplained regions and/or fractured ideogram). The strain selected for secondary background may be incorrect. The estimation of the contribution of primary and secondary background are likely incorrect. This can potentially be addressed with input from the user.</td></tr>
<tr><th>Genotyping Quality</th><td>Excellent (18 N calls)
All reported results are dependent on genotyping quality.</td></tr>
<tr><th>Chromosomal Sex</th><td>XO</td></tr>
<tr><th>Inbreeding Estimate</th><td>70.8% Inbred
(Percentage of the genome (autosomal and X chromosomes) that is homozygous or hemizygous for primary, secondary, and unknown backgrounds. See Genome Analysis)</td></tr>
<tr><th>Constructs Detected</th><td>

BlastR	bpA	Cas9	chlor	cHS4	Cre	DTA	Flp	g_FP	hCMV_a	hCMV_b	hTK_pr	iCre	IRES	Luc	r_FP	rtTA	SV40	tTA
-	-	-	-	-	-	-	-	-	-	-	-	-	-	-	-	-	-	-

</td></tr>
</table>

MiniMUGA Background Analysis v2.3.1

**Refined Ideogram**	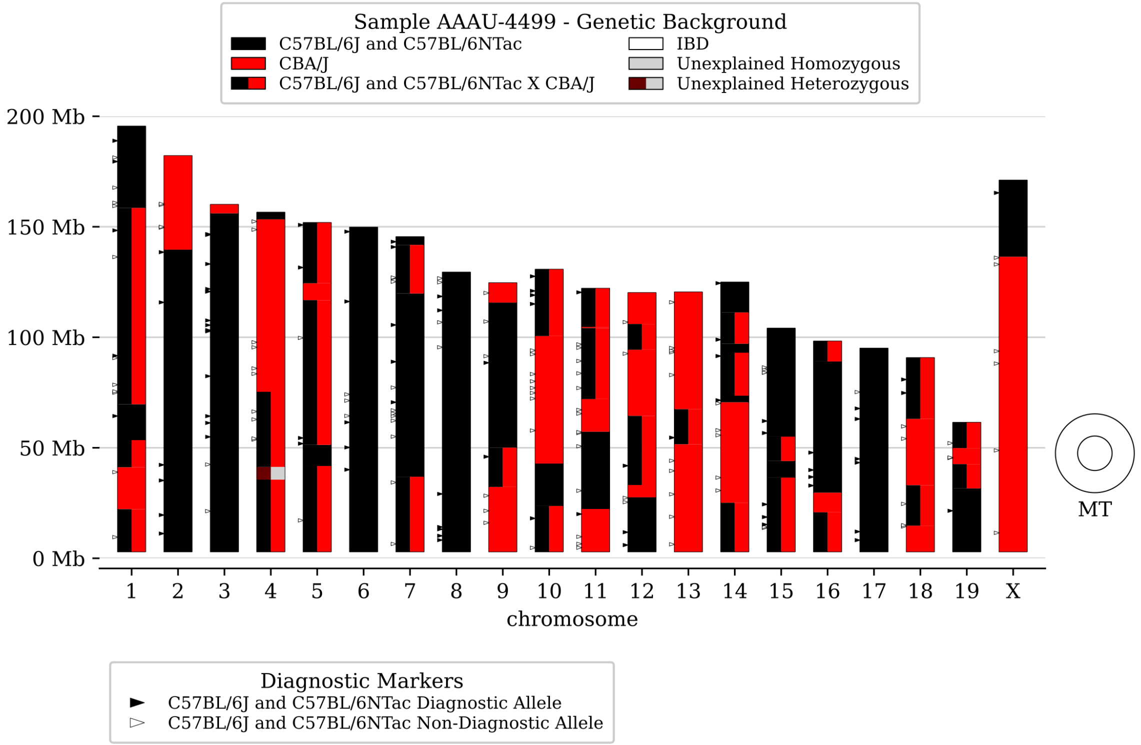

Genome Analysis

Background	Zygosity	Informative Markers	Informative Markers %	Genome %
C57BL/6J and C57BL/6NTac	N/A	1188	44.7%	44.8%
CBA/J	Homozygous	689	25.9%	26.0%
C57BL/6J and C57BL/6NTac X CBA/J	Heterozygous	773	29.1%	29.0%
Unexplained	Heterozygous	10	0.4%	0.2%
	**Total**	2660	100.1%	100.0%

**Y Chromosome**	Not Applicable
**MT Genome**	MT Haplogroup 6 - 100.0% Consistent<br>Includes C57BL/6J, C57BL/6NTac, CBA/J and 165 other strains

MiniMUGA Background Analysis v2.3.1

Backgrounds Detected (Diagnostic Alleles)

	Diagnostic Alleles Observed			
Diagnostic Class	Homozygous	Heterozygous	Potential	% Observed
C57BL/6J, C57BL/6JJicTac, C57BL/6JRj	11	31	102	41.2%
C57BL/6NRj, C57BL/6NTac	3	7	15	66.7%
C57BL/6J, C57BL/6JEiJ, C57BL/6JJicTac, C57BL/6JRj	2	8	21	47.6%
C57BL/6J, C57BL/6JRj	4	4	31	25.8%
C57BL/6NJ, C57BL/6NRj, C57BL/6NTac	2	4	10	60.0%
C57BL/6NCrl, C57BL/6NHsd, C57BL/6NJ, C57BL/6NRj, C57BL/6NTac	2	0	2	100.0%
C57BL/6NHsd, C57BL/6NJ, C57BL/6NRj, C57BL/6NTac	1	0	1	100.0%
B6N-Tyr<c-Brd>/BrdCrCrl, C57BL/6J, C57BL/6JBomTac, C57BL/6JEiJ, C57BL/6JJicTac, C57BL/6JOlaHsd, C57BL/6JRj	0	1	2	50.0%
B6N-Tyr<c-Brd>/BrdCrCrl, C57BL/6J, C57BL/6JJicTac, C57BL/6JRj	0	1	5	20.0%
B6N-Tyr<c-Brd>/BrdCrCrl, C57BL/6NCrl, C57BL/6NHsd, C57BL/6NJ, C57BL/6NRj, C57BL/6NTac	0	1	2	50.0%

Minimal Strain Sets Explaining All Diagnostic Classes (Number of Markers Explained):

- Solution 1: C57BL/6J and C57BL/6NTac
  - C57BL/6J: 62 / 161 (38.5%)
  - C57BL/6NTac: 20 / 30 (66.7%)
- Solution 2: C57BL/6J and C57BL/6NRj
  - C57BL/6J: 62 / 161 (38.5%)
  - C57BL/6NRj: 20 / 30 (66.7%)
- Solution 3: C57BL/6JRj and C57BL/6NTac
  - C57BL/6JRj: 62 / 161 (38.5%)
  - C57BL/6NTac: 20 / 30 (66.7%)
- Solution 4: C57BL/6JRj and C57BL/6NRj
  - C57BL/6JRj: 62 / 161 (38.5%)
  - C57BL/6NRj: 20 / 30 (66.7%)

Chromosome	Start (Mb)	Stop (Mb)	Background	Zygosity
1	3000000	22212275	C57BL/6J and C57BL/6NTac and CBA/J	Heterozygous
1	22212275	41199760	CBA/J	Homozygous
1	41199760	53457225	C57BL/6J and C57BL/6NTac and CBA/J	Heterozygous
1	53457225	69700765	C57BL/6J and C57BL/6NTac	N/A
1	69700765	158479371	C57BL/6J and C57BL/6NTac and CBA/J	Heterozygous
1	158479371	195471971	C57BL/6J and C57BL/6NTac	N/A
2	3000000	139631657	C57BL/6J and C57BL/6NTac	N/A
2	139631657	182113224	CBA/J	Homozygous
3	3000000	156090101	C57BL/6J and C57BL/6NTac	N/A
3	156090101	160039680	CBA/J	Homozygous
4	3000000	35563307	C57BL/6J and C57BL/6NTac and CBA/J	Heterozygous

MiniMUGA Background Analysis v2.3.1

**Diplotype Intervals**	4	35563307	41348396	Unexplained	Heterozygous
	4	41348396	75318594	C57BL/6J and C57BL/6NTac and CBA/J	Heterozygous
	4	75318594	153356388	CBA/J	Homozygous
	4	153356388	156508116	C57BL/6J and C57BL/6NTac	N/A
	5	3000000	41755530	C57BL/6J and C57BL/6NTac and CBA/J	Heterozygous
	5	41755530	51299144	C57BL/6J and C57BL/6NTac	N/A
	5	51299144	116795433	C57BL/6J and C57BL/6NTac and CBA/J	Heterozygous
	5	116795433	124446826	CBA/J	Homozygous
	5	124446826	151834684	C57BL/6J and C57BL/6NTac and CBA/J	Heterozygous
	6	3000000	149736546	C57BL/6J and C57BL/6NTac	N/A
	7	3000000	36856023	C57BL/6J and C57BL/6NTac and CBA/J	Heterozygous
	7	36856023	119823617	C57BL/6J and C57BL/6NTac	N/A
	7	119823617	141750158	C57BL/6J and C57BL/6NTac and CBA/J	Heterozygous
	7	141750158	145441459	C57BL/6J and C57BL/6NTac	N/A
	8	3000000	129401213	C57BL/6J and C57BL/6NTac	N/A
	9	3000000	32287190	CBA/J	Homozygous
	9	32287190	50015698	C57BL/6J and C57BL/6NTac and CBA/J	Heterozygous
	9	50015698	115715944	C57BL/6J and C57BL/6NTac	N/A
	9	115715944	124595110	CBA/J	Homozygous
	10	3000000	23654421	C57BL/6J and C57BL/6NTac and CBA/J	Heterozygous
	10	23654421	42858234	C57BL/6J and C57BL/6NTac	N/A
	10	42858234	100561092	CBA/J	Homozygous
	10	100561092	130694993	C57BL/6J and C57BL/6NTac and CBA/J	Heterozygous
	11	3000000	22302070	CBA/J	Homozygous
	11	22302070	57276575	C57BL/6J and C57BL/6NTac	N/A
	11	57276575	72044583	CBA/J	Homozygous
	11	72044583	104154012	C57BL/6J and C57BL/6NTac and CBA/J	Heterozygous
	11	104154012	104675339	CBA/J	Homozygous
	11	104675339	122082543	C57BL/6J and C57BL/6NTac and CBA/J	Heterozygous
	12	3000000	27585493	C57BL/6J and C57BL/6NTac	N/A
	12	27585493	33130555	CBA/J	Homozygous
	12	33130555	64411355	C57BL/6J and C57BL/6NTac and CBA/J	Heterozygous

MiniMUGA Background Analysis v2.3.1

12	64411355	94371165	CBA/J	Homozygous
12	94371165	105994851	C57BL/6J and C57BL/6NTac and CBA/J	Heterozygous
12	105994851	120129022	CBA/J	Homozygous
13	3000000	51605798	CBA/J	Homozygous
13	51605798	67442927	C57BL/6J and C57BL/6NTac and CBA/J	Heterozygous
13	67442927	120421639	CBA/J	Homozygous
14	3000000	25112834	C57BL/6J and C57BL/6NTac and CBA/J	Heterozygous
14	25112834	70580779	CBA/J	Homozygous
14	70580779	73554565	C57BL/6J and C57BL/6NTac	N/A
14	73554565	93002544	C57BL/6J and C57BL/6NTac and CBA/J	Heterozygous
14	93002544	97106405	C57BL/6J and C57BL/6NTac	N/A
14	97106405	111185375	C57BL/6J and C57BL/6NTac and CBA/J	Heterozygous
14	111185375	124902244	C57BL/6J and C57BL/6NTac	N/A
15	3000000	36473640	C57BL/6J and C57BL/6NTac and CBA/J	Heterozygous
15	36473640	44010563	C57BL/6J and C57BL/6NTac	N/A
15	44010563	55016741	C57BL/6J and C57BL/6NTac and CBA/J	Heterozygous
15	55016741	104043685	C57BL/6J and C57BL/6NTac	N/A
16	3000000	20813513	C57BL/6J and C57BL/6NTac and CBA/J	Heterozygous
16	20813513	29701002	CBA/J	Homozygous
16	29701002	89037512	C57BL/6J and C57BL/6NTac	N/A
16	89037512	98207768	C57BL/6J and C57BL/6NTac and CBA/J	Heterozygous
17	3000000	94987271	C57BL/6J and C57BL/6NTac	N/A
18	3000000	14753212	CBA/J	Homozygous
18	14753212	33050504	C57BL/6J and C57BL/6NTac and CBA/J	Heterozygous
18	33050504	63069205	CBA/J	Homozygous
18	63069205	90702639	C57BL/6J and C57BL/6NTac and CBA/J	Heterozygous
19	3000000	31636352	C57BL/6J and C57BL/6NTac	N/A
19	31636352	42582533	C57BL/6J and C57BL/6NTac and CBA/J	Heterozygous
19	42582533	49870985	CBA/J	Homozygous
19	49870985	61431566	C57BL/6J and C57BL/6NTac and CBA/J	Heterozygous
X	3000000	136441962	CBA/J	Hemizygous
X	136441962	171031299	C57BL/6J and C57BL/6NTac	Hemizygous

MiniMUGA Background Analysis v2.3.1

	MT	0	0	IBD	Hemizygous
