# Supplementary material for: Deletion of 9p drives B-ALL through heterozygous inactivation of Pax5 and Cd72 in preleukemic cells
Source: JCI Insight. 2026 Feb 17;11(7):e199464. doi: 10.1172/jci.insight.199464 (PMC13134721; doi:10.1172/jci.insight.199464)
Supplement: Supplemental data set 1 [file jciinsight-11-199464-s204.zip › Strain_Genotyping/B532-results-report.pdf]

MiniMUGA Background Analysis v2.3.1

**Sample ID**	B532
**Neogen ID**	AAAU-4498
**Summary**	The genotype of this sample is of **excellent** quality. It is **female** and **outbred**, and likely a mix of **C57BL/6J and C57BL/6NTac** and **CBA/J**. Clustering of unexplained markers is evidence of an additional background strain.<br><br>Diagnostic SNPs are likely explained by the presence of the background strains<br>• Solution 1: C57BL/6J and C57BL/6NTac<br>◦ C57BL/6J: 62 / 160 (38.8%)<br>◦ C57BL/6NTac: 15 / 30 (50.0%)<br>• Solution 2: C57BL/6J and C57BL/6NRj<br>◦ C57BL/6J: 62 / 160 (38.8%)<br>◦ C57BL/6NRj: 15 / 30 (50.0%)<br>• Solution 3: C57BL/6JRj and C57BL/6NTac<br>◦ C57BL/6JRj: 62 / 160 (38.8%)<br>◦ C57BL/6NTac: 15 / 30 (50.0%)<br>• Solution 4: C57BL/6JRj and C57BL/6NRj<br>◦ C57BL/6JRj: 62 / 160 (38.8%)<br>◦ C57BL/6NRj: 15 / 30 (50.0%)<br><br>No genetic constructs were detected in this sample.<br><br>WARNING:<br>• This sample likely has more than 2 genetic backgrounds (unexplained regions and/or fractured ideogram). The strain selected for secondary background may be incorrect. The estimation of the contribution of primary and secondary background are likely incorrect. This can potentially be addressed with input from the user.
**Genotyping Quality**	**Excellent (10 N calls)**<br>All reported results are dependent on genotyping quality.
**Chromosomal Sex**	XX
**Inbreeding Estimate**	74.0% Inbred<br>(Percentage of the genome (autosomal and X chromosomes) that is homozygous or hemizygous for primary, secondary, and unknown backgrounds. See Genome Analysis)
**Constructs Detected**	(see table below)

Constructs Detected

BlastR	bpA	Cas9	chlor	cHS4	Cre	DTA	Flp	g_FP	hCMV_a	hCMV_b	hTK_pr	iCre	IRES	Luc	r_FP	rtTA	SV40	tTA
-	-	-	-	-	-	-	-	-	-	-	-	-	-	-	-	-	-	-

MiniMUGA Background Analysis v2.3.1

Refined Ideogram

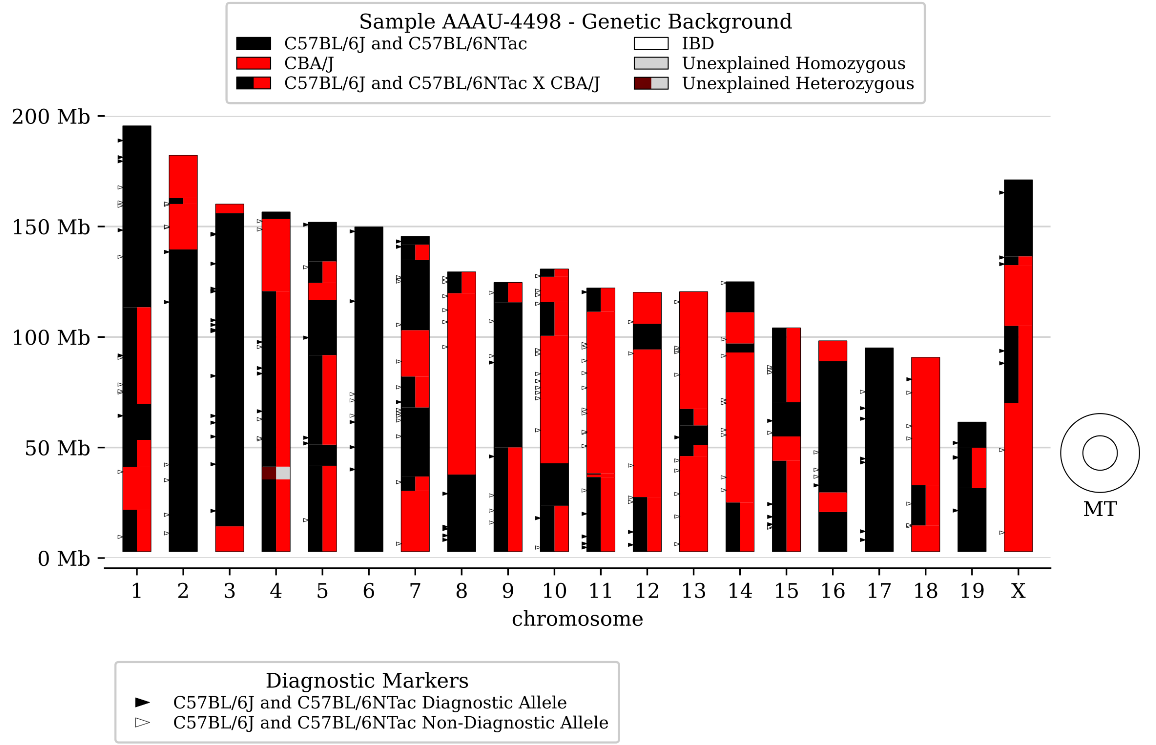

Genome Analysis

Background	Zygosity	Informative Markers	Informative Markers %	Genome %
C57BL/6J and C57BL/6NTac	N/A	1053	39.6%	41.3%
CBA/J	Homozygous	915	34.4%	32.7%
C57BL/6J and C57BL/6NTac X CBA/J	Heterozygous	680	25.6%	25.8%
Unexplained	Heterozygous	10	0.4%	0.2%
	**Total**	2658	100.0%	100.0%

Y Chromosome

Not Applicable

MT Genome

MT Haplogroup 6 - 100.0% Consistent
Includes C57BL/6J, C57BL/6NTac, CBA/J and 165 other strains

MiniMUGA Background Analysis v2.3.1

Backgrounds Detected (Diagnostic Alleles)

Diagnostic Class	Diagnostic Alleles Observed: Homozygous	Heterozygous	Potential	% Observed
C57BL/6J, C57BL/6JJicTac, C57BL/6JRj	10	31	102	40.2%
C57BL/6J, C57BL/6JEiJ, C57BL/6JJicTac, C57BL/6JRj	3	8	21	52.4%
C57BL/6J, C57BL/6JRj	3	5	31	25.8%
C57BL/6NRj, C57BL/6NTac	1	7	15	53.3%
C57BL/6NJ, C57BL/6NRj, C57BL/6NTac	2	2	10	40.0%
B6N-Tyr<c-Brd>/BrdCrCrl, C57BL/6J, C57BL/6JEiJ, C57BL/6JJicTac, C57BL/6JRj	0	1	1	100.0%
B6N-Tyr<c-Brd>/BrdCrCrl, C57BL/6J, C57BL/6JJicTac, C57BL/6JRj	0	1	5	20.0%
B6N-Tyr<c-Brd>/BrdCrCrl, C57BL/6NCrl, C57BL/6NHsd, C57BL/6NJ, C57BL/6NRj, C57BL/6NTac	0	1	2	50.0%
C57BL/6NCrl, C57BL/6NHsd, C57BL/6NJ, C57BL/6NRj, C57BL/6NTac	0	1	2	50.0%
C57BL/6NHsd, C57BL/6NJ, C57BL/6NRj, C57BL/6NTac	0	1	1	100.0%

Minimal Strain Sets Explaining All Diagnostic Classes (Number of Markers Explained):

- Solution 1: C57BL/6J and C57BL/6NTac
  - C57BL/6J: 62 / 160 (38.8%)
  - C57BL/6NTac: 15 / 30 (50.0%)
- Solution 2: C57BL/6J and C57BL/6NRj
  - C57BL/6J: 62 / 160 (38.8%)
  - C57BL/6NRj: 15 / 30 (50.0%)
- Solution 3: C57BL/6JRj and C57BL/6NTac
  - C57BL/6JRj: 62 / 160 (38.8%)
  - C57BL/6NTac: 15 / 30 (50.0%)
- Solution 4: C57BL/6JRj and C57BL/6NRj
  - C57BL/6JRj: 62 / 160 (38.8%)
  - C57BL/6NRj: 15 / 30 (50.0%)

Chromosome	Start (Mb)	Stop (Mb)	Background	Zygosity
1	3000000	21843183	C57BL/6J and C57BL/6NTac and CBA/J	Heterozygous
1	21843183	41199760	CBA/J	Homozygous
1	41199760	53457225	C57BL/6J and C57BL/6NTac and CBA/J	Heterozygous
1	53457225	69700765	C57BL/6J and C57BL/6NTac	N/A
1	69700765	113437009	C57BL/6J and C57BL/6NTac and CBA/J	Heterozygous
1	113437009	195471971	C57BL/6J and C57BL/6NTac	N/A
2	3000000	139631657	C57BL/6J and C57BL/6NTac	N/A
2	139631657	160174252	CBA/J	Homozygous
2	160174252	162852192	C57BL/6J and C57BL/6NTac and CBA/J	Heterozygous
2	162852192	182113224	CBA/J	Homozygous
3	3000000	14328941	CBA/J	Homozygous

MiniMUGA Background Analysis v2.3.1

**Diplotype Intervals**	3	14328941	156090101	C57BL/6J and C57BL/6NTac	N/A
	3	156090101	160039680	CBA/J	Homozygous
	4	3000000	35563307	C57BL/6J and C57BL/6NTac and CBA/J	Heterozygous
	4	35563307	41348396	Unexplained	Heterozygous
	4	41348396	120738488	C57BL/6J and C57BL/6NTac and CBA/J	Heterozygous
	4	120738488	153356388	CBA/J	Homozygous
	4	153356388	156508116	C57BL/6J and C57BL/6NTac	N/A
	5	3000000	41755530	C57BL/6J and C57BL/6NTac and CBA/J	Heterozygous
	5	41755530	51299144	C57BL/6J and C57BL/6NTac	N/A
	5	51299144	91772803	C57BL/6J and C57BL/6NTac and CBA/J	Heterozygous
	5	91772803	116795433	C57BL/6J and C57BL/6NTac	N/A
	5	116795433	124446826	CBA/J	Homozygous
	5	124446826	134172373	C57BL/6J and C57BL/6NTac and CBA/J	Heterozygous
	5	134172373	151834684	C57BL/6J and C57BL/6NTac	N/A
	6	3000000	149736546	C57BL/6J and C57BL/6NTac	N/A
	7	3000000	30335112	CBA/J	Homozygous
	7	30335112	36856023	C57BL/6J and C57BL/6NTac and CBA/J	Heterozygous
	7	36856023	68153750	C57BL/6J and C57BL/6NTac	N/A
	7	68153750	82097045	C57BL/6J and C57BL/6NTac and CBA/J	Heterozygous
	7	82097045	103084424	CBA/J	Homozygous
	7	103084424	134805535	C57BL/6J and C57BL/6NTac	N/A
	7	134805535	141750158	C57BL/6J and C57BL/6NTac and CBA/J	Heterozygous
	7	141750158	145441459	C57BL/6J and C57BL/6NTac	N/A
	8	3000000	37790271	C57BL/6J and C57BL/6NTac	N/A
	8	37790271	119835722	CBA/J	Homozygous
	8	119835722	129401213	C57BL/6J and C57BL/6NTac and CBA/J	Heterozygous
	9	3000000	50015698	C57BL/6J and C57BL/6NTac and CBA/J	Heterozygous
	9	50015698	115715944	C57BL/6J and C57BL/6NTac	N/A
	9	115715944	124595110	C57BL/6J and C57BL/6NTac and CBA/J	Heterozygous
	10	3000000	23654421	C57BL/6J and C57BL/6NTac and CBA/J	Heterozygous
	10	23654421	42858234	C57BL/6J and C57BL/6NTac	N/A
	10	42858234	100561092	CBA/J	Homozygous

MiniMUGA Background Analysis v2.3.1

10	100561092	115781736	C57BL/6J and C57BL/6NTac and CBA/J	Heterozygous
10	115781736	127271560	CBA/J	Homozygous
10	127271560	130694993	C57BL/6J and C57BL/6NTac and CBA/J	Heterozygous
11	3000000	36618681	C57BL/6J and C57BL/6NTac and CBA/J	Heterozygous
11	36618681	37766977	CBA/J	Homozygous
11	37766977	38300130	C57BL/6J and C57BL/6NTac and CBA/J	Heterozygous
11	38300130	111437394	CBA/J	Homozygous
11	111437394	122082543	C57BL/6J and C57BL/6NTac and CBA/J	Heterozygous
12	3000000	27585493	C57BL/6J and C57BL/6NTac and CBA/J	Heterozygous
12	27585493	94371165	CBA/J	Homozygous
12	94371165	105994851	C57BL/6J and C57BL/6NTac	N/A
12	105994851	120129022	CBA/J	Homozygous
13	3000000	46136691	CBA/J	Homozygous
13	46136691	51136860	C57BL/6J and C57BL/6NTac and CBA/J	Heterozygous
13	51136860	60016573	C57BL/6J and C57BL/6NTac	N/A
13	60016573	67442927	C57BL/6J and C57BL/6NTac and CBA/J	Heterozygous
13	67442927	120421639	CBA/J	Homozygous
14	3000000	25112834	C57BL/6J and C57BL/6NTac and CBA/J	Heterozygous
14	25112834	93002544	CBA/J	Homozygous
14	93002544	97106405	C57BL/6J and C57BL/6NTac	N/A
14	97106405	111185375	CBA/J	Homozygous
14	111185375	124902244	C57BL/6J and C57BL/6NTac	N/A
15	3000000	44010563	C57BL/6J and C57BL/6NTac and CBA/J	Heterozygous
15	44010563	55016741	CBA/J	Homozygous
15	55016741	70554147	C57BL/6J and C57BL/6NTac	N/A
15	70554147	104043685	C57BL/6J and C57BL/6NTac and CBA/J	Heterozygous
16	3000000	20813513	C57BL/6J and C57BL/6NTac	N/A
16	20813513	29701002	CBA/J	Homozygous
16	29701002	89037512	C57BL/6J and C57BL/6NTac	N/A
16	89037512	98207768	CBA/J	Homozygous
17	3000000	94987271	C57BL/6J and C57BL/6NTac	N/A
18	3000000	14753212	CBA/J	Homozygous
18	14753212	33050504	C57BL/6J and C57BL/6NTac and CBA/J	Heterozygous

MiniMUGA Background Analysis v2.3.1

	18	33050504	90702639	CBA/J	Homozygous
	19	3000000	31636352	C57BL/6J and C57BL/6NTac	N/A
	19	31636352	49870985	C57BL/6J and C57BL/6NTac and CBA/J	Heterozygous
	19	49870985	61431566	C57BL/6J and C57BL/6NTac	N/A
	X	3000000	70193631	CBA/J	Homozygous
	X	70193631	105020820	C57BL/6J and C57BL/6NTac and CBA/J	Heterozygous
	X	105020820	132528229	CBA/J	Homozygous
	X	132528229	136441962	C57BL/6J and C57BL/6NTac and CBA/J	Heterozygous
	X	136441962	171031299	C57BL/6J and C57BL/6NTac	N/A
	MT	0	0	IBD	Hemizygous
