# Supplementary material for: Deletion of 9p drives B-ALL through heterozygous inactivation of Pax5 and Cd72 in preleukemic cells
Source: JCI Insight. 2026 Feb 17;11(7):e199464. doi: 10.1172/jci.insight.199464 (PMC13134721; doi:10.1172/jci.insight.199464)
Supplement: Supplemental data set 1 [file jciinsight-11-199464-s204.zip › Strain_Genotyping/B834-results-report.pdf]

MiniMUGA Background Analysis v2.3.1

**Sample ID**	B834
**Neogen ID**	AAAU-4543
**Summary**	The genotype of this sample is of **excellent** quality. It is **female** and **outbred**, and likely a mix of **CBA/J** and **C57BL/6JRj**. Clustering of unexplained markers is evidence of an additional background strain.<br><br>Diagnostic SNPs are likely explained by the presence of the background strains<br>• Solution 1: 129S5/SvEvBrd and C57BL/6J and C57BL/6NTac<br>◦ C57BL/6J: 81 / 158 (51.3%)<br>◦ C57BL/6NTac: 10 / 25 (40.0%)<br>◦ 129S5/SvEvBrd: 1 / 5 (20.0%)<br>• Solution 2: 129S5/SvEvBrd and C57BL/6J and C57BL/6NRj<br>◦ C57BL/6J: 81 / 158 (51.3%)<br>◦ C57BL/6NRj: 10 / 25 (40.0%)<br>◦ 129S5/SvEvBrd: 1 / 5 (20.0%)<br>• Solution 3: 129S5/SvEvBrd and C57BL/6JRj and C57BL/6NTac<br>◦ C57BL/6JRj: 81 / 158 (51.3%)<br>◦ C57BL/6NTac: 10 / 25 (40.0%)<br>◦ 129S5/SvEvBrd: 1 / 5 (20.0%)<br>• Solution 4: 129S5/SvEvBrd and C57BL/6JRj and C57BL/6NRj<br>◦ C57BL/6JRj: 81 / 158 (51.3%)<br>◦ C57BL/6NRj: 10 / 25 (40.0%)<br>◦ 129S5/SvEvBrd: 1 / 5 (20.0%)<br><br>NOTE: There is a discrepancy between the diagnostic backgrounds detected and the primary and secondary background analysis (CBA/J, C57BL/6JRj). This is uncommon and should be investigated further.<br><br>No genetic constructs were detected in this sample.<br><br>WARNING:<br>• There is a discrepancy between the diagnostic backgrounds detected ((129S5/SvEvBrd and C57BL/6J and C57BL/6NTac) or (129S5/SvEvBrd and C57BL/6J and C57BL/6NRj) or (129S5/SvEvBrd and C57BL/6JRj and C57BL/6NTac) or (129S5/SvEvBrd and C57BL/6JRj and C57BL/6NRj)) and the primary background (CBA/J) and secondary background (C57BL/6JRj). This is uncommon and should be investigated further.<br>• The presence of a single diagnostic heterozygous call for a single inbred strain should be treated with caution.<br>• This sample likely has more than 2 genetic backgrounds (unexplained regions and/or fractured ideogram). The strain selected for secondary background may be incorrect. The estimation of the contribution of primary and secondary background are likely incorrect. This can potentially be addressed with input from the user.
**Genotyping Quality**	**Excellent (10 N calls)**<br>All reported results are dependent on genotyping quality.
**Chromosomal Sex**	XX
**Inbreeding Estimate**	49.8% Inbred<br>(Percentage of the genome (autosomal and X chromosomes) that is homozygous or hemizygous for primary, secondary, and unknown backgrounds. See Genome Analysis)
**Constructs Detected**	see below

Constructs Detected

BlastR	bpA	Cas9	chlor	cHS4	Cre	DTA	Flp	g_FP	hCMV_a	hCMV_b	hTK_pr	iCre	IRES	Luc	r_FP	rtTA	SV40	tTA
-	-	-	-	-	-	-	-	-	-	-	-	-	-	-	-	-	-	-

MiniMUGA Background Analysis v2.3.1

Refined Ideogram

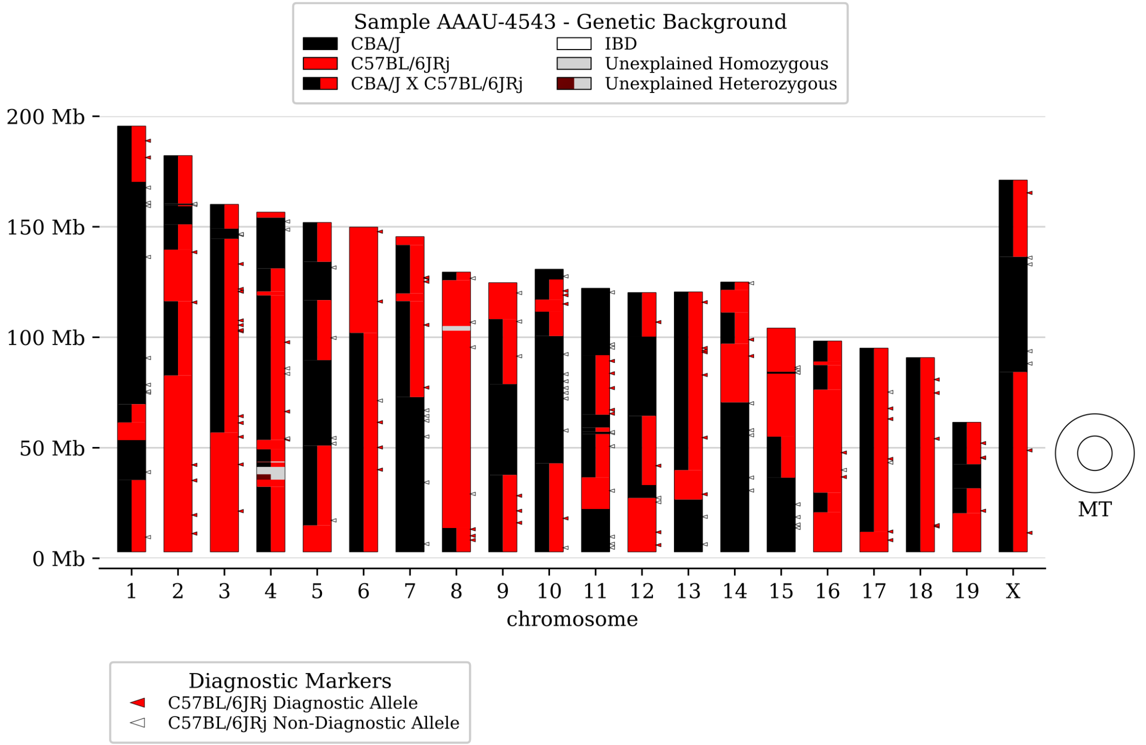

Genome Analysis

Background	Zygosity	Informative Markers	Informative Markers %	Genome %
CBA/J	Homozygous	829	29.5%	26.1%
C57BL/6JRj	Homozygous	617	21.9%	23.5%
CBA/J X C57BL/6JRj	Heterozygous	1344	47.8%	50.1%
Unexplained	Homozygous	11	0.4%	0.2%
Unexplained	Heterozygous	13	0.5%	0.1%
	**Total**	2814	100.1%	100.0%

Y Chromosome

Not Applicable

MT Genome

MT Haplogroup 6 - 100.0% Consistent
Includes C57BL/6JRj, CBA/J and 166 other strains

MiniMUGA Background Analysis v2.3.1

Backgrounds Detected (Diagnostic Alleles)

	Diagnostic Alleles Observed			
**Diagnostic Class**	**Homozygous**	**Heterozygous**	**Potential**	**% Observed**
C57BL/6J, C57BL/6JJicTac, C57BL/6JRj	7	50	102	55.9%
C57BL/6J, C57BL/6JEiJ, C57BL/6JJicTac, C57BL/6JRj	2	9	21	52.4%
C57BL/6J, C57BL/6JRj	1	9	31	32.3%
C57BL/6NRj, C57BL/6NTac	3	3	15	40.0%
C57BL/6NJ, C57BL/6NRj, C57BL/6NTac	1	3	10	40.0%
129S5/SvEvBrd	0	1	5	20.0%
B6N-Tyr<c-Brd>/BrdCrCrl, C57BL/6J, C57BL/6JBomTac, C57BL/6JEiJ, C57BL/6JJicTac, C57BL/6JOlaHsd, C57BL/6JRj	0	1	2	50.0%
B6N-Tyr<c-Brd>/BrdCrCrl, C57BL/6J, C57BL/6JEiJ, C57BL/6JJicTac, C57BL/6JRj	0	1	1	100.0%
C57BL/6J, C57BL/6JEiJ, C57BL/6JJicTac, C57BL/6JOlaHsd, C57BL/6JRj	0	1	1	100.0%

Minimal Strain Sets Explaining All Diagnostic Classes (Number of Markers Explained):

- Solution 1: 129S5/SvEvBrd and C57BL/6J and C57BL/6NTac
  - C57BL/6J: 81 / 158 (51.3%)
  - C57BL/6NTac: 10 / 25 (40.0%)
  - 129S5/SvEvBrd: 1 / 5 (20.0%)
- Solution 2: 129S5/SvEvBrd and C57BL/6J and C57BL/6NRj
  - C57BL/6J: 81 / 158 (51.3%)
  - C57BL/6NRj: 10 / 25 (40.0%)
  - 129S5/SvEvBrd: 1 / 5 (20.0%)
- Solution 3: 129S5/SvEvBrd and C57BL/6JRj and C57BL/6NTac
  - C57BL/6JRj: 81 / 158 (51.3%)
  - C57BL/6NTac: 10 / 25 (40.0%)
  - 129S5/SvEvBrd: 1 / 5 (20.0%)
- Solution 4: 129S5/SvEvBrd and C57BL/6JRj and C57BL/6NRj
  - C57BL/6JRj: 81 / 158 (51.3%)
  - C57BL/6NRj: 10 / 25 (40.0%)
  - 129S5/SvEvBrd: 1 / 5 (20.0%)

Chromosome	Start (Mb)	Stop (Mb)	Background	Zygosity
1	3000000	35382930	CBA/J and C57BL/6JRj	Heterozygous
1	35382930	53457225	CBA/J	Homozygous
1	53457225	61451021	C57BL/6JRj	Homozygous
1	61451021	69700765	CBA/J and C57BL/6JRj	Heterozygous
1	69700765	170316822	CBA/J	Homozygous
1	170316822	195471971	CBA/J and C57BL/6JRj	Heterozygous
2	3000000	82717338	C57BL/6JRj	Homozygous
2	82717338	116294153	CBA/J and C57BL/6JRj	Heterozygous
2	116294153	139631657	C57BL/6JRj	Homozygous
2	139631657	151062687	CBA/J and C57BL/6JRj	Heterozygous
2	151062687	159275367	CBA/J	Homozygous
2	159275367	159754795	CBA/J and C57BL/6JRj	Heterozygous
2	159754795	160529876	CBA/J	Homozygous

MiniMUGA Background Analysis v2.3.1

**Diplotype Intervals**	2	160529876	182113224	CBA/J and C57BL/6JRj	Heterozygous
	3	3000000	56913628	C57BL/6JRj	Homozygous
	3	56913628	144518770	CBA/J and C57BL/6JRj	Heterozygous
	3	144518770	149139970	CBA/J	Homozygous
	3	149139970	160039680	CBA/J and C57BL/6JRj	Heterozygous
	4	3000000	32327128	CBA/J and C57BL/6JRj	Heterozygous
	4	32327128	35563307	C57BL/6JRj	Homozygous
	4	35563307	37995481	Unexplained	Heterozygous
	4	37995481	41348396	Unexplained	Homozygous
	4	41348396	43372387	CBA/J and C57BL/6JRj	Heterozygous
	4	43372387	43819249	Unexplained	Heterozygous
	4	43819249	49280860	CBA/J and C57BL/6JRj	Heterozygous
	4	49280860	53565639	C57BL/6JRj	Homozygous
	4	53565639	118913935	CBA/J and C57BL/6JRj	Heterozygous
	4	118913935	120738488	C57BL/6JRj	Homozygous
	4	120738488	131104093	CBA/J and C57BL/6JRj	Heterozygous
	4	131104093	154141341	CBA/J	Homozygous
	4	154141341	156508116	C57BL/6JRj	Homozygous
	5	3000000	14885741	C57BL/6JRj	Homozygous
	5	14885741	50962467	CBA/J and C57BL/6JRj	Heterozygous
	5	50962467	89660496	CBA/J	Homozygous
	5	89660496	116795433	CBA/J and C57BL/6JRj	Heterozygous
	5	116795433	134172373	CBA/J	Homozygous
	5	134172373	151834684	CBA/J and C57BL/6JRj	Heterozygous
	6	3000000	101966063	CBA/J and C57BL/6JRj	Heterozygous
	6	101966063	149736546	C57BL/6JRj	Homozygous
	7	3000000	72944748	CBA/J	Homozygous
	7	72944748	116328796	CBA/J and C57BL/6JRj	Heterozygous
	7	116328796	119823617	C57BL/6JRj	Homozygous
	7	119823617	141750158	CBA/J and C57BL/6JRj	Heterozygous
	7	141750158	145441459	C57BL/6JRj	Homozygous
	8	3000000	13696571	CBA/J and C57BL/6JRj	Heterozygous
	8	13696571	103034593	C57BL/6JRj	Homozygous
	8	103034593	105092038	Unexplained	Homozygous
	8	105092038	125832225	C57BL/6JRj	Homozygous
	8	125832225	129401213	CBA/J and C57BL/6JRj	Heterozygous
	9	3000000	37691490	CBA/J and C57BL/6JRj	Heterozygous
	9	37691490	78825300	CBA/J	Homozygous
	9	78825300	108206968	CBA/J and C57BL/6JRj	Heterozygous
	9	108206968	124595110	C57BL/6JRj	Homozygous

MiniMUGA Background Analysis v2.3.1

10	3000000	42917049	CBA/J and C57BL/6JRj	Heterozygous
10	42917049	100561092	CBA/J	Homozygous
10	100561092	111566142	CBA/J and C57BL/6JRj	Heterozygous
10	111566142	117048365	C57BL/6JRj	Homozygous
10	117048365	126139155	CBA/J and C57BL/6JRj	Heterozygous
10	126139155	130694993	CBA/J	Homozygous
11	3000000	22302070	CBA/J	Homozygous
11	22302070	36618681	C57BL/6JRj	Homozygous
11	36618681	56158551	CBA/J and C57BL/6JRj	Heterozygous
11	56158551	57257590	CBA/J	Homozygous
11	57257590	59127711	CBA/J and C57BL/6JRj	Heterozygous
11	59127711	64987182	CBA/J	Homozygous
11	64987182	91888658	CBA/J and C57BL/6JRj	Heterozygous
11	91888658	122082543	CBA/J	Homozygous
12	3000000	27287329	C57BL/6JRj	Homozygous
12	27287329	33130555	CBA/J	Homozygous
12	33130555	64411355	CBA/J and C57BL/6JRj	Heterozygous
12	64411355	100284662	CBA/J	Homozygous
12	100284662	120129022	CBA/J and C57BL/6JRj	Heterozygous
13	3000000	26607981	CBA/J	Homozygous
13	26607981	39905651	C57BL/6JRj	Homozygous
13	39905651	120421639	CBA/J and C57BL/6JRj	Heterozygous
14	3000000	70580779	CBA/J	Homozygous
14	70580779	97106405	C57BL/6JRj	Homozygous
14	97106405	111185375	CBA/J and C57BL/6JRj	Heterozygous
14	111185375	121458497	C57BL/6JRj	Homozygous
14	121458497	124902244	CBA/J and C57BL/6JRj	Heterozygous
15	3000000	36473640	CBA/J	Homozygous
15	36473640	55016741	CBA/J and C57BL/6JRj	Heterozygous
15	55016741	83510499	C57BL/6JRj	Homozygous
15	83510499	84406413	CBA/J	Homozygous
15	84406413	104043685	C57BL/6JRj	Homozygous
16	3000000	20813513	C57BL/6JRj	Homozygous
16	20813513	29701002	CBA/J and C57BL/6JRj	Heterozygous
16	29701002	76315797	C57BL/6JRj	Homozygous
16	76315797	87403166	CBA/J and C57BL/6JRj	Heterozygous
16	87403166	89037512	C57BL/6JRj	Homozygous
16	89037512	98207768	CBA/J and C57BL/6JRj	Heterozygous
17	3000000	11948365	C57BL/6JRj	Homozygous
17	11948365	94987271	CBA/J and C57BL/6JRj	Heterozygous

MiniMUGA Background Analysis v2.3.1

	18	3000000	90702639	CBA/J and C57BL/6JRj	Heterozygous
	19	3000000	20394588	C57BL/6JRj	Homozygous
	19	20394588	31636352	CBA/J and C57BL/6JRj	Heterozygous
	19	31636352	42582533	CBA/J	Homozygous
	19	42582533	61431566	CBA/J and C57BL/6JRj	Heterozygous
	X	3000000	84237192	CBA/J and C57BL/6JRj	Heterozygous
	X	84237192	136441962	CBA/J	Homozygous
	X	136441962	171031299	CBA/J and C57BL/6JRj	Heterozygous
	MT	0	0	IBD	Hemizygous
