# Supplementary material for: Deletion of 9p drives B-ALL through heterozygous inactivation of Pax5 and Cd72 in preleukemic cells
Source: JCI Insight. 2026 Feb 17;11(7):e199464. doi: 10.1172/jci.insight.199464 (PMC13134721; doi:10.1172/jci.insight.199464)
Supplement: Supplemental data set 1 [file jciinsight-11-199464-s204.zip › Strain_Genotyping/B835-results-report.pdf]

MiniMUGA Background Analysis v2.3.1

**Sample ID**	B835
**Neogen ID**	AAAU-4542
**Summary**	The genotype of this sample is of **good** quality. Errors in sample background determination are more frequent with this level of genotyping quality, particularly in samples from standard commercial inbred strains. Please consult the Report Guide and Neogen if reported results are unexpected.<br><br>It is **female** and **outbred**, and likely a mix of **C57BL/6J and C57BL/6NTac** and **CBA/J**. Clustering of unexplained markers is evidence of an additional background strain.<br><br>Diagnostic SNPs are likely explained by the presence of the background strains<br>• Solution 1: 129S5/SvEvBrd and C57BL/6J and C57BL/6NRj<br>◦ C57BL/6J: 70 / 155 (45.2%)<br>◦ C57BL/6NRj: 14 / 35 (40.0%)<br>◦ 129S5/SvEvBrd: 1 / 5 (20.0%)<br>• Solution 2: 129S5/SvEvBrd and C57BL/6JRj and C57BL/6NRj<br>◦ C57BL/6JRj: 70 / 155 (45.2%)<br>◦ C57BL/6NRj: 14 / 35 (40.0%)<br>◦ 129S5/SvEvBrd: 1 / 5 (20.0%)<br><br>NOTE: There is a discrepancy between the diagnostic backgrounds detected and the primary and secondary background analysis (CBA/J, C57BL/6J, C57BL/6NTac). This is uncommon and should be investigated further.<br><br>No genetic constructs were detected in this sample.<br><br>WARNING:<br>• There is a discrepancy between the diagnostic backgrounds detected ((129S5/SvEvBrd and C57BL/6J and C57BL/6NRj) or (129S5/SvEvBrd and C57BL/6JRj and C57BL/6NRj)) and the primary background (C57BL/6J and C57BL/6NTac) and secondary background (CBA/J). This is uncommon and should be investigated further.<br>• The presence of a single diagnostic heterozygous call for a single inbred strain should be treated with caution.<br>• This sample likely has more than 2 genetic backgrounds (unexplained regions and/or fractured ideogram). The strain selected for secondary background may be incorrect. The estimation of the contribution of primary and secondary background are likely incorrect. This can potentially be addressed with input from the user.
**Genotyping Quality**	**Good (117 N calls)**<br>All reported results are dependent on genotyping quality.
**Chromosomal Sex**	XX
**Inbreeding Estimate**	54.0% Inbred<br>(Percentage of the genome (autosomal and X chromosomes) that is homozygous or hemizygous for primary, secondary, and unknown backgrounds. See Genome Analysis)

Constructs Detected

BlastR	bpA	Cas9	chlor	cHS4	Cre	DTA	Flp	g_FP	hCMV_a	hCMV_b	hTK_pr	iCre	IRES	Luc	r_FP	rtTA	SV40	tTA
-	-	-	-	-	-	-	-	-	-	-	-	-	-	-	-	-	-	-

MiniMUGA Background Analysis v2.3.1

Refined Ideogram

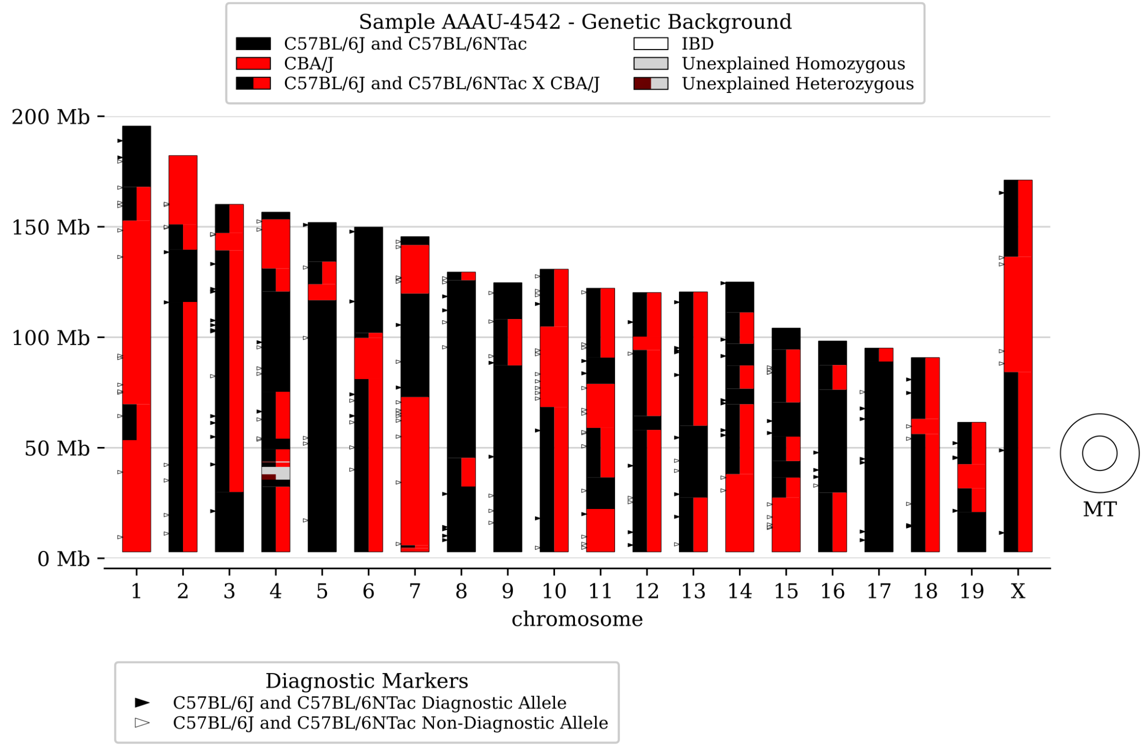

Genome Analysis

Background	Zygosity	Informative Markers	Informative Markers %	Genome %
C57BL/6J and C57BL/6NTac	N/A	925	36.2%	33.6%
CBA/J	Homozygous	489	19.1%	20.3%
C57BL/6J and C57BL/6NTac X CBA/J	Heterozygous	1127	44.1%	45.8%
Unexplained	Homozygous	6	0.2%	0.1%
Unexplained	Heterozygous	8	0.3%	0.1%
	**Total**	2555	99.9%	99.9%

**Y Chromosome**	Not Applicable
**MT Genome**	MT Haplogroup 6 - 100.0% Consistent<br>Includes C57BL/6J, C57BL/6NTac, CBA/J and 165 other strains

MiniMUGA Background Analysis v2.3.1

Backgrounds Detected (Diagnostic Alleles)

Diagnostic Class	Diagnostic Alleles Observed: Homozygous	Heterozygous	Potential	% Observed
C57BL/6J, C57BL/6JJicTac, C57BL/6JRj	10	35	102	44.1%
C57BL/6J, C57BL/6JEiJ, C57BL/6JJicTac, C57BL/6JRj	2	12	21	66.7%
C57BL/6J, C57BL/6JRj	2	8	31	32.3%
C57BL/6NRj, C57BL/6NTac	3	4	15	46.7%
C57BL/6NJ, C57BL/6NRj, C57BL/6NTac	1	4	10	50.0%
C57BL/6NRj	1	1	10	20.0%
129S5/SvEvBrd	0	1	5	20.0%
B6N-Tyr<c-Brd>/BrdCrCrl, C57BL/6J, C57BL/6JEiJ, C57BL/6JJicTac, C57BL/6JRj	0	1	1	100.0%

Minimal Strain Sets Explaining All Diagnostic Classes (Number of Markers Explained):

- Solution 1: 129S5/SvEvBrd and C57BL/6J and C57BL/6NRj
  - C57BL/6J: 70 / 155 (45.2%)
  - C57BL/6NRj: 14 / 35 (40.0%)
  - 129S5/SvEvBrd: 1 / 5 (20.0%)
- Solution 2: 129S5/SvEvBrd and C57BL/6JRj and C57BL/6NRj
  - C57BL/6JRj: 70 / 155 (45.2%)
  - C57BL/6NRj: 14 / 35 (40.0%)
  - 129S5/SvEvBrd: 1 / 5 (20.0%)

Diplotype Intervals

Chromosome	Start (Mb)	Stop (Mb)	Background	Zygosity
1	3000000	53457225	CBA/J	Homozygous
1	53457225	69700765	C57BL/6J and C57BL/6NTac and CBA/J	Heterozygous
1	69700765	152801739	CBA/J	Homozygous
1	152801739	168019536	C57BL/6J and C57BL/6NTac and CBA/J	Heterozygous
1	168019536	195471971	C57BL/6J and C57BL/6NTac	N/A
2	3000000	115970567	C57BL/6J and C57BL/6NTac and CBA/J	Heterozygous
2	115970567	139631657	C57BL/6J and C57BL/6NTac	N/A
2	139631657	151062687	C57BL/6J and C57BL/6NTac and CBA/J	Heterozygous
2	151062687	182113224	CBA/J	Homozygous
3	3000000	30013882	C57BL/6J and C57BL/6NTac	N/A
3	30013882	139297311	C57BL/6J and C57BL/6NTac and CBA/J	Heterozygous
3	139297311	147169673	CBA/J	Homozygous
3	147169673	160039680	C57BL/6J and C57BL/6NTac and CBA/J	Heterozygous
4	3000000	32327128	C57BL/6J and C57BL/6NTac and CBA/J	Heterozygous
4	32327128	35563307	C57BL/6J and C57BL/6NTac	N/A
4	35563307	37995481	Unexplained	Heterozygous
4	37995481	41348396	Unexplained	Homozygous

	4	41348396	43372387	C57BL/6J and C57BL/6NTac and CBA/J	Heterozygous
	4	43372387	43819249	Unexplained	Heterozygous
	4	43819249	49280860	C57BL/6J and C57BL/6NTac and CBA/J	Heterozygous
	4	49280860	54114833	C57BL/6J and C57BL/6NTac	N/A
	4	54114833	75318594	C57BL/6J and C57BL/6NTac and CBA/J	Heterozygous
	4	75318594	120738488	C57BL/6J and C57BL/6NTac	N/A
	4	120738488	131104093	C57BL/6J and C57BL/6NTac and CBA/J	Heterozygous
	4	131104093	153356388	CBA/J	Homozygous
	4	153356388	156508116	C57BL/6J and C57BL/6NTac	N/A
	5	3000000	116795433	C57BL/6J and C57BL/6NTac	N/A
	5	116795433	124011933	CBA/J	Homozygous
	5	124011933	134172373	C57BL/6J and C57BL/6NTac and CBA/J	Heterozygous
	5	134172373	151834684	C57BL/6J and C57BL/6NTac	N/A
	6	3000000	81074930	C57BL/6J and C57BL/6NTac and CBA/J	Heterozygous
	6	81074930	99727922	CBA/J	Homozygous
	6	99727922	101966063	C57BL/6J and C57BL/6NTac and CBA/J	Heterozygous
	6	101966063	149736546	C57BL/6J and C57BL/6NTac	N/A
	7	3000000	3194086	CBA/J	Homozygous
	7	3194086	3211215	C57BL/6J and C57BL/6NTac and CBA/J	Heterozygous
	7	3211215	4418092	CBA/J	Homozygous
	7	4418092	4504539	C57BL/6J and C57BL/6NTac and CBA/J	Heterozygous
	7	4504539	4847141	CBA/J	Homozygous
	7	4847141	5883380	C57BL/6J and C57BL/6NTac and CBA/J	Heterozygous
	7	5883380	72944748	CBA/J	Homozygous
	7	72944748	119823617	C57BL/6J and C57BL/6NTac	N/A
	7	119823617	141749783	CBA/J	Homozygous
	7	141749783	145441459	C57BL/6J and C57BL/6NTac	N/A
	8	3000000	32467133	C57BL/6J and C57BL/6NTac	N/A
	8	32467133	45403996	C57BL/6J and C57BL/6NTac and CBA/J	Heterozygous
	8	45403996	125832225	C57BL/6J and C57BL/6NTac	N/A
	8	125832225	129401213	C57BL/6J and C57BL/6NTac and CBA/J	Heterozygous
	9	3000000	87286420	C57BL/6J and C57BL/6NTac	N/A

MiniMUGA Background Analysis v2.3.1

	9	87286420	108206968	C57BL/6J and C57BL/6NTac and CBA/J	Heterozygous
	9	108206968	124595110	C57BL/6J and C57BL/6NTac	N/A
	10	3000000	68444097	C57BL/6J and C57BL/6NTac and CBA/J	Heterozygous
	10	68444097	104861956	CBA/J	Homozygous
	10	104861956	130694993	C57BL/6J and C57BL/6NTac and CBA/J	Heterozygous
	11	3000000	22302070	CBA/J	Homozygous
	11	22302070	36618681	C57BL/6J and C57BL/6NTac	N/A
	11	36618681	58994880	C57BL/6J and C57BL/6NTac and CBA/J	Heterozygous
	11	58994880	78913943	CBA/J	Homozygous
	11	78913943	90803561	C57BL/6J and C57BL/6NTac	N/A
	11	90803561	122082543	C57BL/6J and C57BL/6NTac and CBA/J	Heterozygous
	12	3000000	58069123	C57BL/6J and C57BL/6NTac and CBA/J	Heterozygous
	12	58069123	64411355	C57BL/6J and C57BL/6NTac	N/A
	12	64411355	94246475	C57BL/6J and C57BL/6NTac and CBA/J	Heterozygous
	12	94246475	100284662	CBA/J	Homozygous
	12	100284662	120129022	C57BL/6J and C57BL/6NTac and CBA/J	Heterozygous
	13	3000000	27430346	C57BL/6J and C57BL/6NTac and CBA/J	Heterozygous
	13	27430346	60016573	C57BL/6J and C57BL/6NTac	N/A
	13	60016573	120421639	C57BL/6J and C57BL/6NTac and CBA/J	Heterozygous
	14	3000000	38092288	CBA/J	Homozygous
	14	38092288	69660428	C57BL/6J and C57BL/6NTac and CBA/J	Heterozygous
	14	69660428	76773671	C57BL/6J and C57BL/6NTac	N/A
	14	76773671	87213383	C57BL/6J and C57BL/6NTac and CBA/J	Heterozygous
	14	87213383	97106405	C57BL/6J and C57BL/6NTac	N/A
	14	97106405	111185375	C57BL/6J and C57BL/6NTac and CBA/J	Heterozygous
	14	111185375	124902244	C57BL/6J and C57BL/6NTac	N/A
	15	3000000	27443955	CBA/J	Homozygous
	15	27443955	36473640	C57BL/6J and C57BL/6NTac and CBA/J	Heterozygous
	15	36473640	44010563	C57BL/6J and C57BL/6NTac	N/A
	15	44010563	55016741	C57BL/6J and C57BL/6NTac and CBA/J	Heterozygous
	15	55016741	70554147	C57BL/6J and C57BL/6NTac	N/A

MiniMUGA Background Analysis v2.3.1

	15	70554147	94412127	C57BL/6J and C57BL/6NTac and CBA/J	Heterozygous
	15	94412127	104043685	C57BL/6J and C57BL/6NTac	N/A
	16	3000000	29701002	C57BL/6J and C57BL/6NTac and CBA/J	Heterozygous
	16	29701002	76315797	C57BL/6J and C57BL/6NTac	N/A
	16	76315797	87403166	C57BL/6J and C57BL/6NTac and CBA/J	Heterozygous
	16	87403166	98207768	C57BL/6J and C57BL/6NTac	N/A
	17	3000000	89028083	C57BL/6J and C57BL/6NTac	N/A
	17	89028083	94987271	C57BL/6J and C57BL/6NTac and CBA/J	Heterozygous
	18	3000000	56215244	C57BL/6J and C57BL/6NTac and CBA/J	Heterozygous
	18	56215244	63069205	CBA/J	Homozygous
	18	63069205	90702639	C57BL/6J and C57BL/6NTac and CBA/J	Heterozygous
	19	3000000	20955280	C57BL/6J and C57BL/6NTac	N/A
	19	20955280	31636352	C57BL/6J and C57BL/6NTac and CBA/J	Heterozygous
	19	31636352	42582533	CBA/J	Homozygous
	19	42582533	61431566	C57BL/6J and C57BL/6NTac and CBA/J	Heterozygous
	X	3000000	84237192	C57BL/6J and C57BL/6NTac and CBA/J	Heterozygous
	X	84237192	136441962	CBA/J	Homozygous
	X	136441962	171031299	C57BL/6J and C57BL/6NTac and CBA/J	Heterozygous
	MT	0	0	IBD	Hemizygous
