# Supplementary material for: Deletion of 9p drives B-ALL through heterozygous inactivation of Pax5 and Cd72 in preleukemic cells
Source: JCI Insight. 2026 Feb 17;11(7):e199464. doi: 10.1172/jci.insight.199464 (PMC13134721; doi:10.1172/jci.insight.199464)
Supplement: Supplemental data set 1 [file jciinsight-11-199464-s204.zip › Strain_Genotyping/B854-results-report.pdf]

MiniMUGA Background Analysis v2.3.1

**Sample ID**	B854
**Neogen ID**	AAAU-4508
**Summary**	The genotype of this sample is of **excellent** quality. It is **XO** and **outbred**, and likely a mix of **C57BL/6J and C57BL/6NTac** and **CBA/J**. Clustering of unexplained markers is evidence of an additional background strain.<br><br>Diagnostic SNPs are likely explained by the presence of the background strains<br>• Solution 1: C57BL/6J and C57BL/6NTac<br>◦ C57BL/6J: 59 / 160 (36.9%)<br>◦ C57BL/6NTac: 14 / 27 (51.9%)<br>• Solution 2: C57BL/6J and C57BL/6NRj<br>◦ C57BL/6J: 59 / 160 (36.9%)<br>◦ C57BL/6NRj: 14 / 27 (51.9%)<br>• Solution 3: C57BL/6JRj and C57BL/6NTac<br>◦ C57BL/6JRj: 59 / 160 (36.9%)<br>◦ C57BL/6NTac: 14 / 27 (51.9%)<br>• Solution 4: C57BL/6JRj and C57BL/6NRj<br>◦ C57BL/6JRj: 59 / 160 (36.9%)<br>◦ C57BL/6NRj: 14 / 27 (51.9%)<br><br>No genetic constructs were detected in this sample.
**Genotyping Quality**	**Excellent (33 N calls)**<br>All reported results are dependent on genotyping quality.
**Chromosomal Sex**	XO
**Inbreeding Estimate**	68.2% Inbred<br>(Percentage of the genome (autosomal and X chromosomes) that is homozygous or hemizygous for primary, secondary, and unknown backgrounds. See Genome Analysis)

Constructs Detected

BlastR	bpA	Cas9	chlor	cHS4	Cre	DTA	Flp	g_FP	hCMV_a	hCMV_b	hTK_pr	iCre	IRES	Luc	r_FP	rtTA	SV40	tTA
-	-	-	-	-	-	-	-	-	-	-	-	-	-	-	-	-	-	-

Refined Ideogram

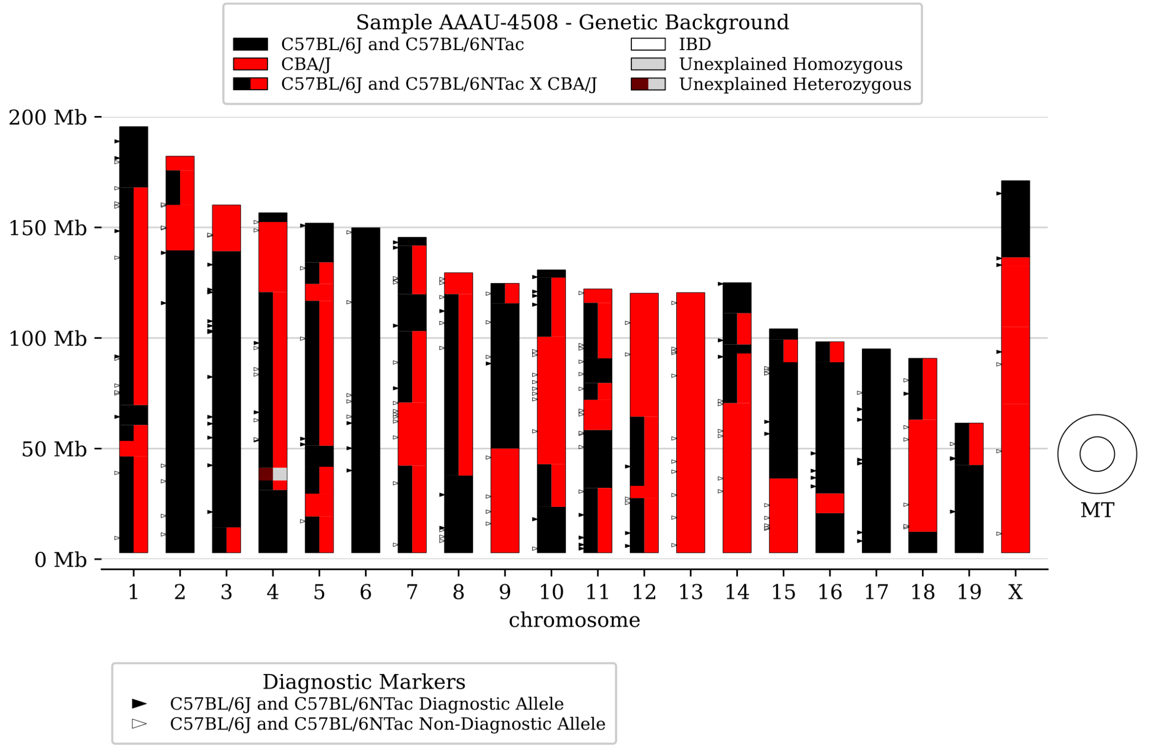

MiniMUGA Background Analysis v2.3.1

Genome Analysis

Background	Zygosity	Informative Markers	Informative Markers %	Genome %
C57BL/6J and C57BL/6NTac	N/A	1035	39.0%	39.4%
CBA/J	Homozygous	671	25.3%	27.2%
C57BL/6J and C57BL/6NTac X CBA/J	Heterozygous	935	35.2%	33.1%
Unexplained	Heterozygous	13	0.5%	0.2%
	**Total**	2654	100.0%	99.9%

Y Chromosome

Not Applicable

MT Genome

MT Haplogroup 6 - 100.0% Consistent
Includes C57BL/6J, C57BL/6NTac, CBA/J and 165 other strains

Backgrounds Detected (Diagnostic Alleles)

	Diagnostic Alleles Observed			
**Diagnostic Class**	**Homozygous**	**Heterozygous**	**Potential**	**% Observed**
C57BL/6J, C57BL/6JJicTac, C57BL/6JRj	12	27	102	38.2%
C57BL/6J, C57BL/6JEiJ, C57BL/6JJicTac, C57BL/6JRj	4	5	21	42.9%
C57BL/6J, C57BL/6JRj	4	5	31	29.0%
C57BL/6NRj, C57BL/6NTac	3	5	15	53.3%
C57BL/6NJ, C57BL/6NRj, C57BL/6NTac	2	3	10	50.0%
B6N-Tyr<c-Brd>/BrdCrCrl, C57BL/6J, C57BL/6JEiJ, C57BL/6JJicTac, C57BL/6JRj	0	1	1	100.0%
B6N-Tyr<c-Brd>/BrdCrCrl, C57BL/6J, C57BL/6JJicTac, C57BL/6JRj	0	1	5	20.0%
B6N-Tyr<c-Brd>/BrdCrCrl, C57BL/6NCrl, C57BL/6NHsd, C57BL/6NJ, C57BL/6NRj, C57BL/6NTac	0	1	2	50.0%

Minimal Strain Sets Explaining All Diagnostic Classes (Number of Markers Explained):

- Solution 1: C57BL/6J and C57BL/6NTac
  - C57BL/6J: 59 / 160 (36.9%)
  - C57BL/6NTac: 14 / 27 (51.9%)
- Solution 2: C57BL/6J and C57BL/6NRj
  - C57BL/6J: 59 / 160 (36.9%)
  - C57BL/6NRj: 14 / 27 (51.9%)
- Solution 3: C57BL/6JRj and C57BL/6NTac
  - C57BL/6JRj: 59 / 160 (36.9%)
  - C57BL/6NTac: 14 / 27 (51.9%)
- Solution 4: C57BL/6JRj and C57BL/6NRj
  - C57BL/6JRj: 59 / 160 (36.9%)
  - C57BL/6NRj: 14 / 27 (51.9%)

Chromosome	Start (Mb)	Stop (Mb)	Background	Zygosity
1	3000000	46434628	C57BL/6J and C57BL/6NTac and CBA/J	Heterozygous
1	46434628	53457225	CBA/J	Homozygous
1	53457225	60621237	C57BL/6J and C57BL/6NTac and CBA/J	Heterozygous
1	60621237	69700765	C57BL/6J and C57BL/6NTac	N/A
1	69700765	168019536	C57BL/6J and C57BL/6NTac and CBA/J	Heterozygous

MiniMUGA Background Analysis v2.3.1

**Diplotype Intervals**	1	168019536	195471971	C57BL/6J and C57BL/6NTac	N/A
	2	3000000	139631657	C57BL/6J and C57BL/6NTac	N/A
	2	139631657	160174252	CBA/J	Homozygous
	2	160174252	175780822	C57BL/6J and C57BL/6NTac and CBA/J	Heterozygous
	2	175780822	182113224	CBA/J	Homozygous
	3	3000000	14328941	C57BL/6J and C57BL/6NTac and CBA/J	Heterozygous
	3	14328941	139297311	C57BL/6J and C57BL/6NTac	N/A
	3	139297311	160039680	CBA/J	Homozygous
	4	3000000	31293075	C57BL/6J and C57BL/6NTac	N/A
	4	31293075	35563307	C57BL/6J and C57BL/6NTac and CBA/J	Heterozygous
	4	35563307	41348396	Unexplained	Heterozygous
	4	41348396	120738488	C57BL/6J and C57BL/6NTac and CBA/J	Heterozygous
	4	120738488	152440879	CBA/J	Homozygous
	4	152440879	156508116	C57BL/6J and C57BL/6NTac	N/A
	5	3000000	19267794	C57BL/6J and C57BL/6NTac and CBA/J	Heterozygous
	5	19267794	29588943	CBA/J	Homozygous
	5	29588943	41755530	C57BL/6J and C57BL/6NTac and CBA/J	Heterozygous
	5	41755530	51299144	C57BL/6J and C57BL/6NTac	N/A
	5	51299144	116795433	C57BL/6J and C57BL/6NTac and CBA/J	Heterozygous
	5	116795433	124446826	CBA/J	Homozygous
	5	124446826	134172373	C57BL/6J and C57BL/6NTac and CBA/J	Heterozygous
	5	134172373	151834684	C57BL/6J and C57BL/6NTac	N/A
	6	3000000	149736546	C57BL/6J and C57BL/6NTac	N/A
	7	3000000	42273938	C57BL/6J and C57BL/6NTac and CBA/J	Heterozygous
	7	42273938	70828686	CBA/J	Homozygous
	7	70828686	103084424	C57BL/6J and C57BL/6NTac and CBA/J	Heterozygous
	7	103084424	119823617	C57BL/6J and C57BL/6NTac	N/A
	7	119823617	141750158	C57BL/6J and C57BL/6NTac and CBA/J	Heterozygous
	7	141750158	145441459	C57BL/6J and C57BL/6NTac	N/A
	8	3000000	37790271	C57BL/6J and C57BL/6NTac	N/A
	8	37790271	119835722	C57BL/6J and C57BL/6NTac and CBA/J	Heterozygous
	8	119835722	129401213	CBA/J	Homozygous

MiniMUGA Background Analysis v2.3.1

9	3000000	50015698	CBA/J	Homozygous
9	50015698	115715944	C57BL/6J and C57BL/6NTac	N/A
9	115715944	124595110	C57BL/6J and C57BL/6NTac and CBA/J	Heterozygous
10	3000000	23654421	C57BL/6J and C57BL/6NTac	N/A
10	23654421	42917049	C57BL/6J and C57BL/6NTac and CBA/J	Heterozygous
10	42917049	100561092	CBA/J	Homozygous
10	100561092	127271560	C57BL/6J and C57BL/6NTac and CBA/J	Heterozygous
10	127271560	130694993	C57BL/6J and C57BL/6NTac	N/A
11	3000000	32168654	C57BL/6J and C57BL/6NTac and CBA/J	Heterozygous
11	32168654	58406228	C57BL/6J and C57BL/6NTac	N/A
11	58406228	72044583	CBA/J	Homozygous
11	72044583	79617327	C57BL/6J and C57BL/6NTac and CBA/J	Heterozygous
11	79617327	90803561	C57BL/6J and C57BL/6NTac	N/A
11	90803561	115896459	C57BL/6J and C57BL/6NTac and CBA/J	Heterozygous
11	115896459	122082543	CBA/J	Homozygous
12	3000000	27585493	C57BL/6J and C57BL/6NTac and CBA/J	Heterozygous
12	27585493	33130555	CBA/J	Homozygous
12	33130555	64411355	C57BL/6J and C57BL/6NTac and CBA/J	Heterozygous
12	64411355	120129022	CBA/J	Homozygous
13	3000000	120421639	CBA/J	Homozygous
14	3000000	70580779	CBA/J	Homozygous
14	70580779	93002544	C57BL/6J and C57BL/6NTac and CBA/J	Heterozygous
14	93002544	97106405	C57BL/6J and C57BL/6NTac	N/A
14	97106405	111185375	C57BL/6J and C57BL/6NTac and CBA/J	Heterozygous
14	111185375	124902244	C57BL/6J and C57BL/6NTac	N/A
15	3000000	36473640	CBA/J	Homozygous
15	36473640	89025824	C57BL/6J and C57BL/6NTac	N/A
15	89025824	99220995	C57BL/6J and C57BL/6NTac and CBA/J	Heterozygous
15	99220995	104043685	C57BL/6J and C57BL/6NTac	N/A
16	3000000	20813513	C57BL/6J and C57BL/6NTac	N/A
16	20813513	29701002	CBA/J	Homozygous
16	29701002	89037512	C57BL/6J and C57BL/6NTac	N/A

MiniMUGA Background Analysis v2.3.1

	16	89037512	98207768	C57BL/6J and C57BL/6NTac and CBA/J	Heterozygous
	17	3000000	94987271	C57BL/6J and C57BL/6NTac	N/A
	18	3000000	12406382	C57BL/6J and C57BL/6NTac	N/A
	18	12406382	63069205	CBA/J	Homozygous
	18	63069205	90702639	C57BL/6J and C57BL/6NTac and CBA/J	Heterozygous
	19	3000000	42582533	C57BL/6J and C57BL/6NTac	N/A
	19	42582533	61431566	C57BL/6J and C57BL/6NTac and CBA/J	Heterozygous
	X	3000000	70193631	CBA/J	Hemizygous
	X	70193631	105020820	C57BL/6J and C57BL/6NTac and CBA/J	Hemizygous
	X	105020820	132528229	CBA/J	Hemizygous
	X	132528229	136441962	C57BL/6J and C57BL/6NTac and CBA/J	Hemizygous
	X	136441962	171031299	C57BL/6J and C57BL/6NTac	Hemizygous
	MT	0	0	IBD	Hemizygous
