# Supplementary material for: Deletion of 9p drives B-ALL through heterozygous inactivation of Pax5 and Cd72 in preleukemic cells
Source: JCI Insight. 2026 Feb 17;11(7):e199464. doi: 10.1172/jci.insight.199464 (PMC13134721; doi:10.1172/jci.insight.199464)
Supplement: Supplemental data set 1 [file jciinsight-11-199464-s204.zip › Strain_Genotyping/B857-results-report.pdf]

MiniMUGA Background Analysis v2.3.1

<table>
<tr><th>Sample ID</th><td>B857</td></tr>
<tr><th>Neogen ID</th><td>AAAU-4501</td></tr>
<tr><th>Summary</th><td>
The genotype of this sample is of good quality. Errors in sample background determination are more frequent with this level of genotyping quality, particularly in samples from standard commercial inbred strains. Please consult the Report Guide and Neogen if reported results are unexpected.

It is XO and outbred, and likely a mix of C57BL/6J and C57BL/6NTac and CBA/J. Clustering of unexplained markers is evidence of an additional background strain.

Diagnostic SNPs are likely explained by the presence of the background strains

Solution 1: C57BL/6J and C57BL/6NTac and DBA/2J
C57BL/6J: 64 / 162 (39.5%)
C57BL/6NTac: 15 / 29 (51.7%)
DBA/2J: 1 / 117 (0.9%)
Solution 2: C57BL/6J and C57BL/6NRj and DBA/2J
C57BL/6J: 64 / 162 (39.5%)
C57BL/6NRj: 15 / 29 (51.7%)
DBA/2J: 1 / 117 (0.9%)
Solution 3: C57BL/6JRj and C57BL/6NTac and DBA/2J
C57BL/6JRj: 64 / 162 (39.5%)
C57BL/6NTac: 15 / 29 (51.7%)
DBA/2J: 1 / 117 (0.9%)
Solution 4: C57BL/6JRj and C57BL/6NRj and DBA/2J
C57BL/6JRj: 64 / 162 (39.5%)
C57BL/6NRj: 15 / 29 (51.7%)
DBA/2J: 1 / 117 (0.9%)

NOTE: There is a discrepancy between the diagnostic backgrounds detected and the primary and secondary background analysis (CBA/J, C57BL/6J, C57BL/6NTac). This is uncommon and should be investigated further.

No genetic constructs were detected in this sample.

WARNING:

There is a discrepancy between the diagnostic backgrounds detected ((C57BL/6J and C57BL/6NTac and DBA/2J) or (C57BL/6J and C57BL/6NRj and DBA/2J) or (C57BL/6JRj and C57BL/6NTac and DBA/2J) or (C57BL/6JRj and C57BL/6NRj and DBA/2J)) and the primary background (C57BL/6J and C57BL/6NTac) and secondary background (CBA/J). This is uncommon and should be investigated further.
The presence of a single diagnostic heterozygous call for a single inbred strain should be treated with caution.
This sample likely has more than 2 genetic backgrounds (unexplained regions and/or fractured ideogram). The strain selected for secondary background may be incorrect. The estimation of the contribution of primary and secondary background are likely incorrect. This can potentially be addressed with input from the user.

</td></tr>
<tr><th>Genotyping Quality</th><td>Good (160 N calls)
All reported results are dependent on genotyping quality.</td></tr>
<tr><th>Chromosomal Sex</th><td>XO</td></tr>
<tr><th>Inbreeding Estimate</th><td>70.7% Inbred
(Percentage of the genome (autosomal and X chromosomes) that is homozygous or hemizygous for primary, secondary, and unknown backgrounds. See Genome Analysis)</td></tr>
</table>

MiniMUGA Background Analysis v2.3.1

Constructs Detected

BlastR	bpA	Cas9	chlor	cHS4	Cre	DTA	Flp	g_FP	hCMV_a	hCMV_b	hTK_pr	iCre	IRES	Luc	r_FP	rtTA	SV40	tTA
-	-	-	-	-	-	-	-	-	-	-	-	-	-	-	-	-	-	-

Refined Ideogram

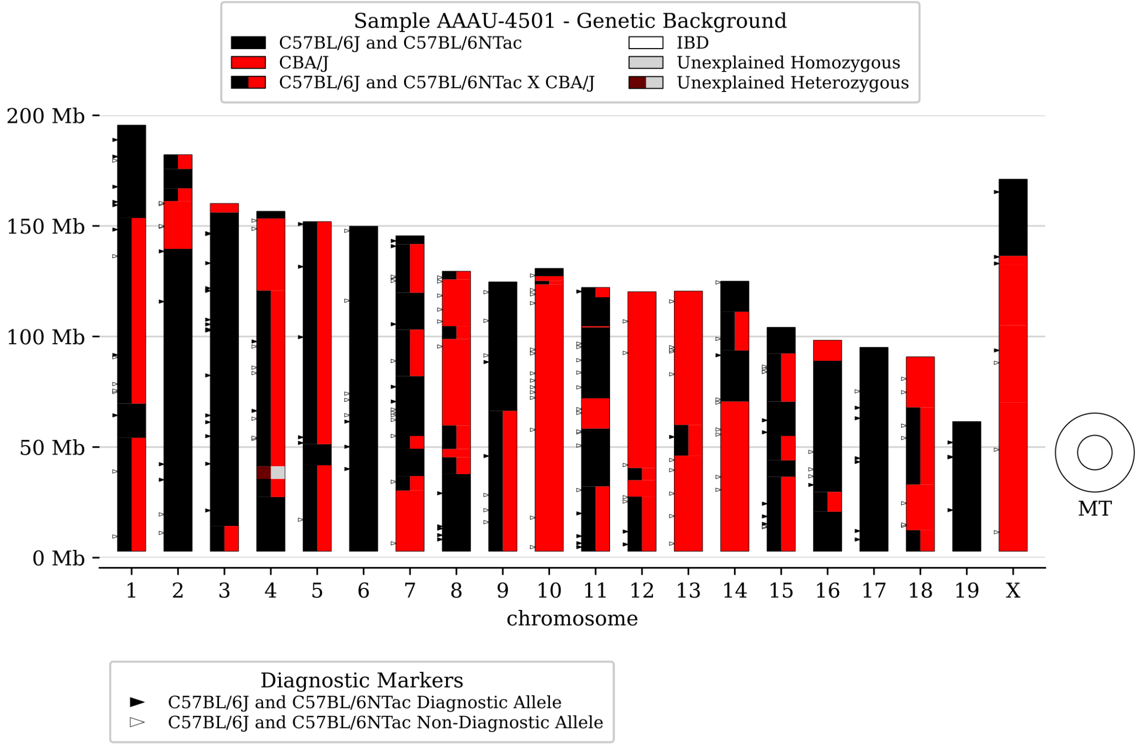

Genome Analysis

Background	Zygosity	Informative Markers	Informative Markers %	Genome %
C57BL/6J and C57BL/6NTac	N/A	1151	45.6%	42.4%
CBA/J	Homozygous	656	26.0%	26.9%
C57BL/6J and C57BL/6NTac X CBA/J	Heterozygous	704	27.9%	30.5%
Unexplained	Heterozygous	15	0.6%	0.2%
	**Total**	2526	100.1%	100.0%

Y Chromosome

Not Applicable

MT Genome

MT Haplogroup 6 - 100.0% Consistent
Includes C57BL/6J, C57BL/6NTac, CBA/J and 165 other strains

MiniMUGA Background Analysis v2.3.1

Backgrounds Detected (Diagnostic Alleles)

Diagnostic Class	Diagnostic Alleles Observed			
	Homozygous	Heterozygous	Potential	% Observed
C57BL/6J, C57BL/6JJicTac, C57BL/6JRj	11	28	102	38.2%
C57BL/6J, C57BL/6JRj	4	6	31	32.3%
C57BL/6J, C57BL/6JEiJ, C57BL/6JJicTac, C57BL/6JRj	1	11	21	57.1%
C57BL/6NRj, C57BL/6NTac	0	9	15	60.0%
C57BL/6NJ, C57BL/6NRj, C57BL/6NTac	1	3	10	40.0%
C57BL/6NCrl, C57BL/6NHsd, C57BL/6NJ, C57BL/6NRj, C57BL/6NTac	1	0	2	50.0%
B6N-Tyr<c-Brd>/BrdCrCrl, C57BL/6J, C57BL/6JBomTac, C57BL/6JEiJ, C57BL/6JJicTac, C57BL/6JOlaHsd, C57BL/6JRj	0	1	2	50.0%
B6N-Tyr<c-Brd>/BrdCrCrl, C57BL/6J, C57BL/6JEiJ, C57BL/6JJicTac, C57BL/6JRj	0	1	1	100.0%
B6N-Tyr<c-Brd>/BrdCrCrl, C57BL/6J, C57BL/6JJicTac, C57BL/6JRj	0	1	5	20.0%
B6N-Tyr<c-Brd>/BrdCrCrl, C57BL/6NCrl, C57BL/6NHsd, C57BL/6NJ, C57BL/6NRj, C57BL/6NTac	0	1	2	50.0%
DBA/2J	0	1	117	0.9%

Minimal Strain Sets Explaining All Diagnostic Classes (Number of Markers Explained):

- Solution 1: C57BL/6J and C57BL/6NTac and DBA/2J
  - C57BL/6J: 64 / 162 (39.5%)
  - C57BL/6NTac: 15 / 29 (51.7%)
  - DBA/2J: 1 / 117 (0.9%)
- Solution 2: C57BL/6J and C57BL/6NRj and DBA/2J
  - C57BL/6J: 64 / 162 (39.5%)
  - C57BL/6NRj: 15 / 29 (51.7%)
  - DBA/2J: 1 / 117 (0.9%)
- Solution 3: C57BL/6JRj and C57BL/6NTac and DBA/2J
  - C57BL/6JRj: 64 / 162 (39.5%)
  - C57BL/6NTac: 15 / 29 (51.7%)
  - DBA/2J: 1 / 117 (0.9%)
- Solution 4: C57BL/6JRj and C57BL/6NRj and DBA/2J
  - C57BL/6JRj: 64 / 162 (39.5%)
  - C57BL/6NRj: 15 / 29 (51.7%)
  - DBA/2J: 1 / 117 (0.9%)

Chromosome	Start (Mb)	Stop (Mb)	Background	Zygosity
1	3000000	54159014	C57BL/6J and C57BL/6NTac and CBA/J	Heterozygous
1	54159014	69700765	C57BL/6J and C57BL/6NTac	N/A
1	69700765	153548642	C57BL/6J and C57BL/6NTac and CBA/J	Heterozygous
1	153548642	195471971	C57BL/6J and C57BL/6NTac	N/A
2	3000000	139631657	C57BL/6J and C57BL/6NTac	N/A
2	139631657	161221795	CBA/J	Homozygous
2	161221795	166963888	C57BL/6J and C57BL/6NTac and CBA/J	Heterozygous

MiniMUGA Background Analysis v2.3.1

**Diplotype Intervals**	2	166963888	175780822	C57BL/6J and C57BL/6NTac	N/A
	2	175780822	182113224	C57BL/6J and C57BL/6NTac and CBA/J	Heterozygous
	3	3000000	14328941	C57BL/6J and C57BL/6NTac and CBA/J	Heterozygous
	3	14328941	156090101	C57BL/6J and C57BL/6NTac	N/A
	3	156090101	160039680	CBA/J	Homozygous
	4	3000000	27432072	C57BL/6J and C57BL/6NTac	N/A
	4	27432072	35563307	C57BL/6J and C57BL/6NTac and CBA/J	Heterozygous
	4	35563307	41348396	Unexplained	Heterozygous
	4	41348396	120738488	C57BL/6J and C57BL/6NTac and CBA/J	Heterozygous
	4	120738488	153356388	CBA/J	Homozygous
	4	153356388	156508116	C57BL/6J and C57BL/6NTac	N/A
	5	3000000	41755530	C57BL/6J and C57BL/6NTac and CBA/J	Heterozygous
	5	41755530	51299144	C57BL/6J and C57BL/6NTac	N/A
	5	51299144	151834684	C57BL/6J and C57BL/6NTac and CBA/J	Heterozygous
	6	3000000	149736546	C57BL/6J and C57BL/6NTac	N/A
	7	3000000	30335112	CBA/J	Homozygous
	7	30335112	36856023	C57BL/6J and C57BL/6NTac and CBA/J	Heterozygous
	7	36856023	49270765	C57BL/6J and C57BL/6NTac	N/A
	7	49270765	54993432	C57BL/6J and C57BL/6NTac and CBA/J	Heterozygous
	7	54993432	82097045	C57BL/6J and C57BL/6NTac	N/A
	7	82097045	103084424	C57BL/6J and C57BL/6NTac and CBA/J	Heterozygous
	7	103084424	119823617	C57BL/6J and C57BL/6NTac	N/A
	7	119823617	141750158	C57BL/6J and C57BL/6NTac and CBA/J	Heterozygous
	7	141750158	145441459	C57BL/6J and C57BL/6NTac	N/A
	8	3000000	37790271	C57BL/6J and C57BL/6NTac	N/A
	8	37790271	45375506	C57BL/6J and C57BL/6NTac and CBA/J	Heterozygous
	8	45375506	49154575	CBA/J	Homozygous
	8	49154575	59780851	C57BL/6J and C57BL/6NTac and CBA/J	Heterozygous
	8	59780851	98889795	CBA/J	Homozygous
	8	98889795	104681042	C57BL/6J and C57BL/6NTac and CBA/J	Heterozygous
	8	104681042	125832225	CBA/J	Homozygous

MiniMUGA Background Analysis v2.3.1

8	125832225	129401213	C57BL/6J and C57BL/6NTac and CBA/J	Heterozygous
9	3000000	66341356	C57BL/6J and C57BL/6NTac and CBA/J	Heterozygous
9	66341356	124595110	C57BL/6J and C57BL/6NTac	N/A
10	3000000	123578116	CBA/J	Homozygous
10	123578116	125116966	C57BL/6J and C57BL/6NTac and CBA/J	Heterozygous
10	125116966	127271560	CBA/J	Homozygous
10	127271560	130694993	C57BL/6J and C57BL/6NTac	N/A
11	3000000	32168654	C57BL/6J and C57BL/6NTac and CBA/J	Heterozygous
11	32168654	58406228	C57BL/6J and C57BL/6NTac	N/A
11	58406228	72044583	CBA/J	Homozygous
11	72044583	104154012	C57BL/6J and C57BL/6NTac	N/A
11	104154012	104675339	CBA/J	Homozygous
11	104675339	117737311	C57BL/6J and C57BL/6NTac	N/A
11	117737311	122082543	C57BL/6J and C57BL/6NTac and CBA/J	Heterozygous
12	3000000	27585493	C57BL/6J and C57BL/6NTac and CBA/J	Heterozygous
12	27585493	35044597	CBA/J	Homozygous
12	35044597	40524158	C57BL/6J and C57BL/6NTac and CBA/J	Heterozygous
12	40524158	120129022	CBA/J	Homozygous
13	3000000	46136691	CBA/J	Homozygous
13	46136691	60016573	C57BL/6J and C57BL/6NTac and CBA/J	Heterozygous
13	60016573	120421639	CBA/J	Homozygous
14	3000000	70580779	CBA/J	Homozygous
14	70580779	93706418	C57BL/6J and C57BL/6NTac	N/A
14	93706418	111152686	C57BL/6J and C57BL/6NTac and CBA/J	Heterozygous
14	111152686	124902244	C57BL/6J and C57BL/6NTac	N/A
15	3000000	36532074	C57BL/6J and C57BL/6NTac and CBA/J	Heterozygous
15	36532074	44010563	C57BL/6J and C57BL/6NTac	N/A
15	44010563	55016741	C57BL/6J and C57BL/6NTac and CBA/J	Heterozygous
15	55016741	70554147	C57BL/6J and C57BL/6NTac	N/A
15	70554147	92274837	C57BL/6J and C57BL/6NTac and CBA/J	Heterozygous
15	92274837	104043685	C57BL/6J and C57BL/6NTac	N/A
16	3000000	20813513	C57BL/6J and C57BL/6NTac	N/A

MiniMUGA Background Analysis v2.3.1

	16	20813513	29701002	C57BL/6J and C57BL/6NTac and CBA/J	Heterozygous
	16	29701002	89037512	C57BL/6J and C57BL/6NTac	N/A
	16	89037512	98207768	CBA/J	Homozygous
	17	3000000	94987271	C57BL/6J and C57BL/6NTac	N/A
	18	3000000	12406382	C57BL/6J and C57BL/6NTac and CBA/J	Heterozygous
	18	12406382	33050504	CBA/J	Homozygous
	18	33050504	67937187	C57BL/6J and C57BL/6NTac and CBA/J	Heterozygous
	18	67937187	90702639	CBA/J	Homozygous
	19	3000000	61431566	C57BL/6J and C57BL/6NTac	N/A
	X	3000000	70193631	CBA/J	Hemizygous
	X	70193631	105020820	C57BL/6J and C57BL/6NTac and CBA/J	Hemizygous
	X	105020820	132528229	CBA/J	Hemizygous
	X	132528229	136441962	C57BL/6J and C57BL/6NTac and CBA/J	Hemizygous
	X	136441962	171031299	C57BL/6J and C57BL/6NTac	Hemizygous
	MT	0	0	IBD	Hemizygous
