# Supplementary material for: Deletion of 9p drives B-ALL through heterozygous inactivation of Pax5 and Cd72 in preleukemic cells
Source: JCI Insight. 2026 Feb 17;11(7):e199464. doi: 10.1172/jci.insight.199464 (PMC13134721; doi:10.1172/jci.insight.199464)
Supplement: Supplemental data set 1 [file jciinsight-11-199464-s204.zip › Strain_Genotyping/B924-results-report.pdf]

MiniMUGA Background Analysis v2.3.1

**Sample ID**	B924
**Neogen ID**	AAAU-5380
**Summary**	The genotype of this sample is of **excellent** quality. It is **female** and **outbred**, and likely a mix of **C57BL/6J and C57BL/6NTac** and **CBA/J**. Clustering of unexplained markers is evidence of an additional background strain.<br><br>Diagnostic SNPs are likely explained by the presence of the background strains<br>• Solution 1: 129S5/SvEvBrd and C57BL/6J and C57BL/6NRj<br>◦ C57BL/6J: 70 / 155 (45.2%)<br>◦ C57BL/6NRj: 15 / 37 (40.5%)<br>◦ 129S5/SvEvBrd: 1 / 5 (20.0%)<br>• Solution 2: 129S5/SvEvBrd and C57BL/6JRj and C57BL/6NRj<br>◦ C57BL/6JRj: 70 / 155 (45.2%)<br>◦ C57BL/6NRj: 15 / 37 (40.5%)<br>◦ 129S5/SvEvBrd: 1 / 5 (20.0%)<br><br>NOTE: There is a discrepancy between the diagnostic backgrounds detected and the primary and secondary background analysis (C57BL/6J, CBA/J, C57BL/6NTac). This is uncommon and should be investigated further.<br><br>No genetic constructs were detected in this sample.<br><br>WARNING:<br>• There is a discrepancy between the diagnostic backgrounds detected ((129S5/SvEvBrd and C57BL/6J and C57BL/6NRj) or (129S5/SvEvBrd and C57BL/6JRj and C57BL/6NRj)) and the primary background (C57BL/6J and C57BL/6NTac) and secondary background (CBA/J). This is uncommon and should be investigated further.<br>• The presence of a single diagnostic heterozygous call for a single inbred strain should be treated with caution.<br>• This sample likely has more than 2 genetic backgrounds (unexplained regions and/or fractured ideogram). The strain selected for secondary background may be incorrect. The estimation of the contribution of primary and secondary background are likely incorrect. This can potentially be addressed with input from the user.
**Genotyping Quality**	**Excellent (5 N calls)**<br>All reported results are dependent on genotyping quality.
**Chromosomal Sex**	XX
**Inbreeding Estimate**	56.6% Inbred<br>(Percentage of the genome (autosomal and X chromosomes) that is homozygous or hemizygous for primary, secondary, and unknown backgrounds. See Genome Analysis)

Constructs Detected

BlastR	bpA	Cas9	chlor	cHS4	Cre	DTA	Flp	g_FP	hCMV_a	hCMV_b	hTK_pr	iCre	IRES	Luc	r_FP	rtTA	SV40	tTA
-	-	-	-	-	-	-	-	-	-	-	-	-	-	-	-	-	-	-

MiniMUGA Background Analysis v2.3.1

Refined Ideogram

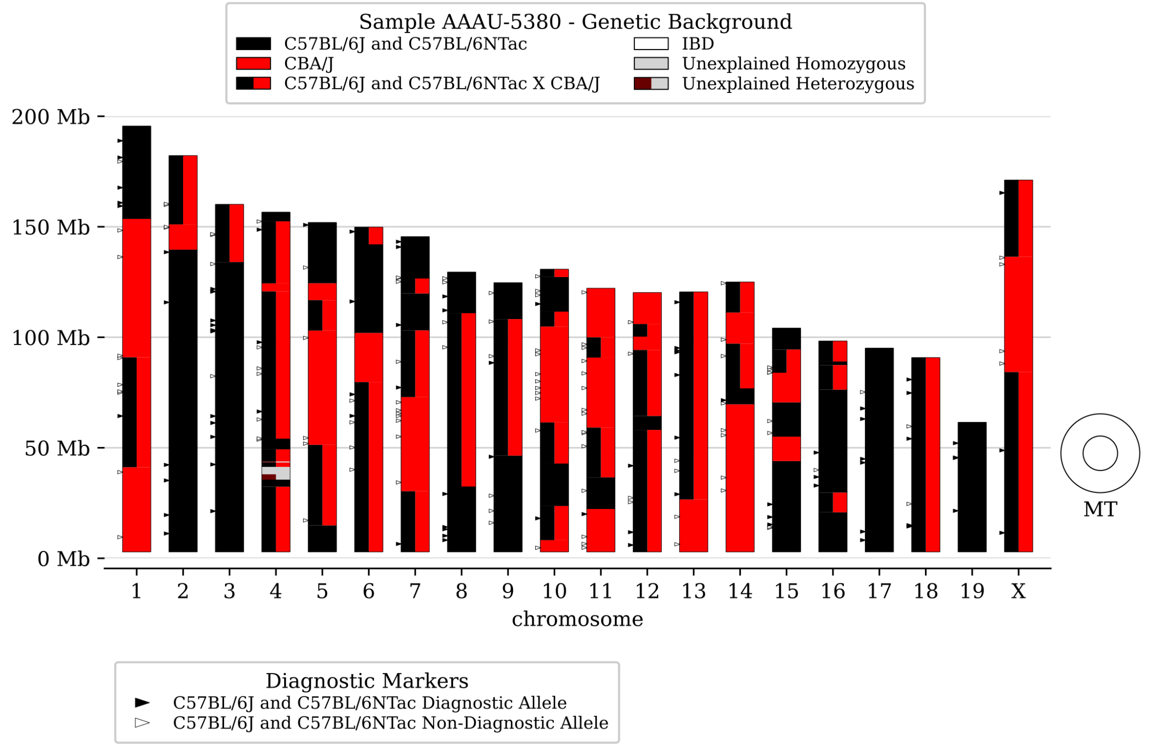

Genome Analysis

Background	Zygosity	Informative Markers	Informative Markers %	Genome %
C57BL/6J and C57BL/6NTac	N/A	1032	38.6%	34.6%
CBA/J	Homozygous	588	22.0%	21.9%
C57BL/6J and C57BL/6NTac X CBA/J	Heterozygous	1038	38.9%	43.3%
Unexplained	Homozygous	5	0.2%	0.1%
Unexplained	Heterozygous	8	0.3%	0.1%
	**Total**	2671	100.0%	100.0%

Y Chromosome

Not Applicable

MT Genome

MT Haplogroup 6 - 100.0% Consistent
Includes C57BL/6J, C57BL/6NTac, CBA/J and 165 other strains

MiniMUGA Background Analysis v2.3.1

Backgrounds Detected (Diagnostic Alleles)

	Diagnostic Alleles Observed			
**Diagnostic Class**	**Homozygous**	**Heterozygous**	**Potential**	**% Observed**
C57BL/6J, C57BL/6JJicTac, C57BL/6JRj	20	29	102	48.0%
C57BL/6J, C57BL/6JRj	6	4	31	32.3%
C57BL/6J, C57BL/6JEiJ, C57BL/6JJicTac, C57BL/6JRj	4	6	21	47.6%
C57BL/6NRj, C57BL/6NTac	2	5	15	46.7%
C57BL/6NJ, C57BL/6NRj, C57BL/6NTac	0	6	10	60.0%
129S5/SvEvBrd	0	1	5	20.0%
B6N-Tyr<c-Brd>/BrdCrCrl, C57BL/6J, C57BL/6JEiJ, C57BL/6JJicTac, C57BL/6JRj	0	1	1	100.0%
B6N-Tyr<c-Brd>/BrdCrCrl, C57BL/6NCrl, C57BL/6NHsd, C57BL/6NJ, C57BL/6NRj, C57BL/6NTac	0	1	2	50.0%
C57BL/6NRj	0	1	10	10.0%

Minimal Strain Sets Explaining All Diagnostic Classes (Number of Markers Explained):

- Solution 1: 129S5/SvEvBrd and C57BL/6J and C57BL/6NRj
    - C57BL/6J: 70 / 155 (45.2%)
    - C57BL/6NRj: 15 / 37 (40.5%)
    - 129S5/SvEvBrd: 1 / 5 (20.0%)
- Solution 2: 129S5/SvEvBrd and C57BL/6JRj and C57BL/6NRj
    - C57BL/6JRj: 70 / 155 (45.2%)
    - C57BL/6NRj: 15 / 37 (40.5%)
    - 129S5/SvEvBrd: 1 / 5 (20.0%)

Chromosome	Start (Mb)	Stop (Mb)	Background	Zygosity
1	3000000	41199760	CBA/J	Homozygous
1	41199760	90903197	C57BL/6J and C57BL/6NTac and CBA/J	Heterozygous
1	90903197	153548642	CBA/J	Homozygous
1	153548642	195471971	C57BL/6J and C57BL/6NTac	N/A
2	3000000	139631657	C57BL/6J and C57BL/6NTac	N/A
2	139631657	151062687	CBA/J	Homozygous
2	151062687	182113224	C57BL/6J and C57BL/6NTac and CBA/J	Heterozygous
3	3000000	134049530	C57BL/6J and C57BL/6NTac	N/A
3	134049530	160039680	C57BL/6J and C57BL/6NTac and CBA/J	Heterozygous
4	3000000	32327128	C57BL/6J and C57BL/6NTac and CBA/J	Heterozygous
4	32327128	35563307	C57BL/6J and C57BL/6NTac	N/A
4	35563307	37995481	Unexplained	Heterozygous
4	37995481	41348396	Unexplained	Homozygous
4	41348396	43372387	C57BL/6J and C57BL/6NTac and CBA/J	Heterozygous
4	43372387	43819249	Unexplained	Heterozygous

MiniMUGA Background Analysis v2.3.1

Diplotype Intervals

4	43819249	49280860	C57BL/6J and C57BL/6NTac and CBA/J	Heterozygous
4	49280860	54114833	C57BL/6J and C57BL/6NTac	N/A
4	54114833	120738488	C57BL/6J and C57BL/6NTac and CBA/J	Heterozygous
4	120738488	124400069	CBA/J	Homozygous
4	124400069	152440879	C57BL/6J and C57BL/6NTac and CBA/J	Heterozygous
4	152440879	156508116	C57BL/6J and C57BL/6NTac	N/A
5	3000000	14885741	C57BL/6J and C57BL/6NTac	N/A
5	14885741	51299144	C57BL/6J and C57BL/6NTac and CBA/J	Heterozygous
5	51299144	103011554	CBA/J	Homozygous
5	103011554	116795433	C57BL/6J and C57BL/6NTac and CBA/J	Heterozygous
5	116795433	124446826	CBA/J	Homozygous
5	124446826	151834684	C57BL/6J and C57BL/6NTac	N/A
6	3000000	79701235	C57BL/6J and C57BL/6NTac and CBA/J	Heterozygous
6	79701235	101966063	CBA/J	Homozygous
6	101966063	142043514	C57BL/6J and C57BL/6NTac	N/A
6	142043514	149736546	C57BL/6J and C57BL/6NTac and CBA/J	Heterozygous
7	3000000	30335112	C57BL/6J and C57BL/6NTac and CBA/J	Heterozygous
7	30335112	72944748	CBA/J	Homozygous
7	72944748	103084424	C57BL/6J and C57BL/6NTac and CBA/J	Heterozygous
7	103084424	119823617	C57BL/6J and C57BL/6NTac	N/A
7	119823617	126580094	C57BL/6J and C57BL/6NTac and CBA/J	Heterozygous
7	126580094	145441459	C57BL/6J and C57BL/6NTac	N/A
8	3000000	32467133	C57BL/6J and C57BL/6NTac	N/A
8	32467133	110881875	C57BL/6J and C57BL/6NTac and CBA/J	Heterozygous
8	110881875	129401213	C57BL/6J and C57BL/6NTac	N/A
9	3000000	46452041	C57BL/6J and C57BL/6NTac	N/A
9	46452041	108206968	C57BL/6J and C57BL/6NTac and CBA/J	Heterozygous
9	108206968	124595110	C57BL/6J and C57BL/6NTac	N/A
10	3000000	8205640	CBA/J	Homozygous
10	8205640	23654421	C57BL/6J and C57BL/6NTac and CBA/J	Heterozygous
10	23654421	42858234	C57BL/6J and C57BL/6NTac	N/A

MiniMUGA Background Analysis v2.3.1

	10	42858234	61450853	C57BL/6J and C57BL/6NTac and CBA/J	Heterozygous
	10	61450853	104861956	CBA/J	Homozygous
	10	104861956	111566142	C57BL/6J and C57BL/6NTac and CBA/J	Heterozygous
	10	111566142	127271560	C57BL/6J and C57BL/6NTac	N/A
	10	127271560	130694993	C57BL/6J and C57BL/6NTac and CBA/J	Heterozygous
	11	3000000	22302070	CBA/J	Homozygous
	11	22302070	36618681	C57BL/6J and C57BL/6NTac	N/A
	11	36618681	59127711	C57BL/6J and C57BL/6NTac and CBA/J	Heterozygous
	11	59127711	90803561	CBA/J	Homozygous
	11	90803561	99943553	C57BL/6J and C57BL/6NTac and CBA/J	Heterozygous
	11	99943553	122082543	CBA/J	Homozygous
	12	3000000	58069123	C57BL/6J and C57BL/6NTac and CBA/J	Heterozygous
	12	58069123	64411355	C57BL/6J and C57BL/6NTac	N/A
	12	64411355	94246475	C57BL/6J and C57BL/6NTac and CBA/J	Heterozygous
	12	94246475	100284662	CBA/J	Homozygous
	12	100284662	105994851	C57BL/6J and C57BL/6NTac and CBA/J	Heterozygous
	12	105994851	120129022	CBA/J	Homozygous
	13	3000000	26607981	CBA/J	Homozygous
	13	26607981	120421639	C57BL/6J and C57BL/6NTac and CBA/J	Heterozygous
	14	3000000	69660428	CBA/J	Homozygous
	14	69660428	76871639	C57BL/6J and C57BL/6NTac	N/A
	14	76871639	97106405	C57BL/6J and C57BL/6NTac and CBA/J	Heterozygous
	14	97106405	111185375	CBA/J	Homozygous
	14	111185375	124902244	C57BL/6J and C57BL/6NTac and CBA/J	Heterozygous
	15	3000000	44010563	C57BL/6J and C57BL/6NTac	N/A
	15	44010563	55016741	CBA/J	Homozygous
	15	55016741	70554147	C57BL/6J and C57BL/6NTac	N/A
	15	70554147	83940554	CBA/J	Homozygous
	15	83940554	94412127	C57BL/6J and C57BL/6NTac and CBA/J	Heterozygous
	15	94412127	104043685	C57BL/6J and C57BL/6NTac	N/A
	16	3000000	20813513	C57BL/6J and C57BL/6NTac	N/A
	16	20813513	29701002	C57BL/6J and C57BL/6NTac and CBA/J	Heterozygous

MiniMUGA Background Analysis v2.3.1

	16	29701002	76315797	C57BL/6J and C57BL/6NTac	N/A
	16	76315797	87403166	C57BL/6J and C57BL/6NTac and CBA/J	Heterozygous
	16	87403166	89037512	C57BL/6J and C57BL/6NTac	N/A
	16	89037512	98207768	C57BL/6J and C57BL/6NTac and CBA/J	Heterozygous
	17	3000000	94987271	C57BL/6J and C57BL/6NTac	N/A
	18	3000000	90702639	C57BL/6J and C57BL/6NTac and CBA/J	Heterozygous
	19	3000000	61431566	C57BL/6J and C57BL/6NTac	N/A
	X	3000000	84237192	C57BL/6J and C57BL/6NTac and CBA/J	Heterozygous
	X	84237192	136441962	CBA/J	Homozygous
	X	136441962	171031299	C57BL/6J and C57BL/6NTac and CBA/J	Heterozygous
	MT	0	0	IBD	Hemizygous
