# Supplementary material for: Deletion of 9p drives B-ALL through heterozygous inactivation of Pax5 and Cd72 in preleukemic cells
Source: JCI Insight. 2026 Feb 17;11(7):e199464. doi: 10.1172/jci.insight.199464 (PMC13134721; doi:10.1172/jci.insight.199464)
Supplement: Supplemental data set 1 [file jciinsight-11-199464-s204.zip › Strain_Genotyping/G063-results-report.pdf]

MiniMUGA Background Analysis v2.3.1

**Sample ID**	G063
**Neogen ID**	AAAU-5386
**Summary**	The genotype of this sample is of **excellent** quality. It is **female** and **outbred**, and likely a mix of **C57BL/6J and C57BL/6NTac** and **CBA/J**. Clustering of unexplained markers is evidence of an additional background strain.<br><br>Diagnostic SNPs are likely explained by the presence of the background strains<br>• Solution 1: 129S5/SvEvBrd and C57BL/6J and C57BL/6NRj<br>◦ C57BL/6J: 80 / 158 (50.6%)<br>◦ C57BL/6NRj: 13 / 35 (37.1%)<br>◦ 129S5/SvEvBrd: 1 / 5 (20.0%)<br>• Solution 2: 129S5/SvEvBrd and C57BL/6JRj and C57BL/6NRj<br>◦ C57BL/6JRj: 80 / 158 (50.6%)<br>◦ C57BL/6NRj: 13 / 35 (37.1%)<br>◦ 129S5/SvEvBrd: 1 / 5 (20.0%)<br><br>NOTE: There is a discrepancy between the diagnostic backgrounds detected and the primary and secondary background analysis (C57BL/6J, CBA/J, C57BL/6NTac). This is uncommon and should be investigated further.<br><br>No genetic constructs were detected in this sample.<br><br>WARNING:<br>• There is a discrepancy between the diagnostic backgrounds detected ((129S5/SvEvBrd and C57BL/6J and C57BL/6NRj) or (129S5/SvEvBrd and C57BL/6JRj and C57BL/6NRj)) and the primary background (C57BL/6J and C57BL/6NTac) and secondary background (CBA/J). This is uncommon and should be investigated further.<br>• The presence of a single diagnostic heterozygous call for a single inbred strain should be treated with caution.<br>• This sample likely has more than 2 genetic backgrounds (unexplained regions and/or fractured ideogram). The strain selected for secondary background may be incorrect. The estimation of the contribution of primary and secondary background are likely incorrect. This can potentially be addressed with input from the user.
**Genotyping Quality**	**Excellent (7 N calls)**<br>All reported results are dependent on genotyping quality.
**Chromosomal Sex**	XX
**Inbreeding Estimate**	53.4% Inbred<br>(Percentage of the genome (autosomal and X chromosomes) that is homozygous or hemizygous for primary, secondary, and unknown backgrounds. See Genome Analysis)

Constructs Detected

BlastR	bpA	Cas9	chlor	cHS4	Cre	DTA	Flp	g_FP	hCMV_a	hCMV_b	hTK_pr	iCre	IRES	Luc	r_FP	rtTA	SV40	tTA
-	-	-	-	-	-	-	-	-	-	-	-	-	-	-	-	-	-	-

MiniMUGA Background Analysis v2.3.1

Refined Ideogram

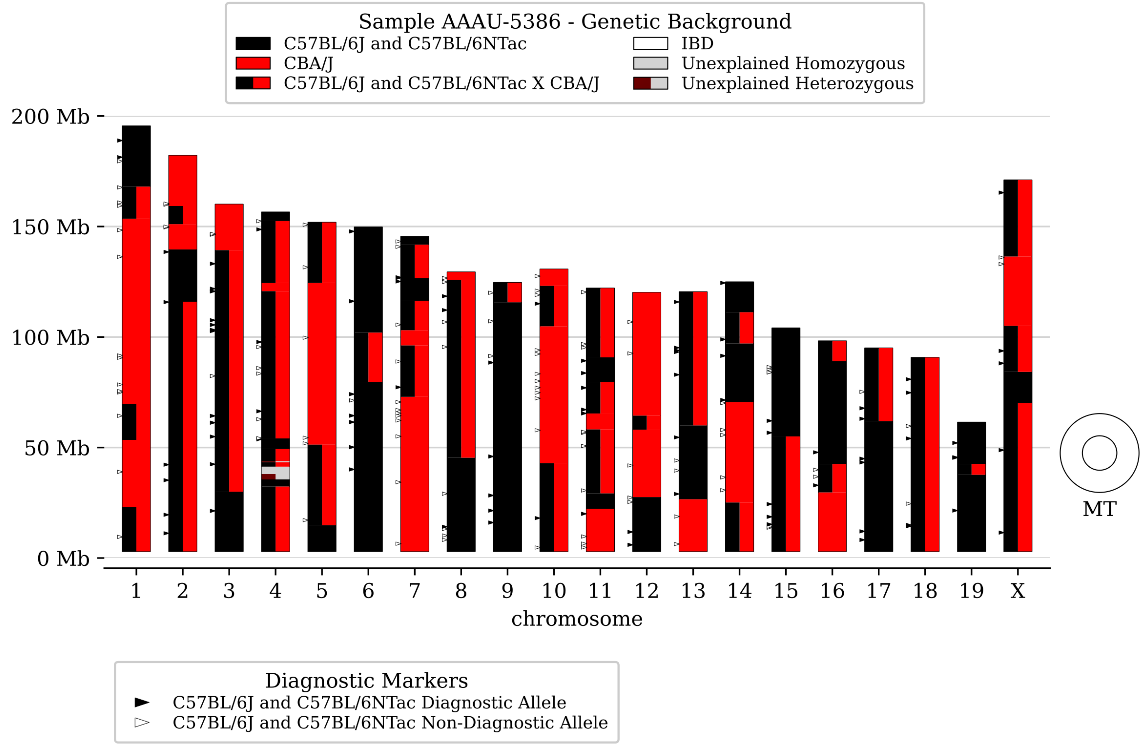

Genome Analysis

Background	Zygosity	Informative Markers	Informative Markers %	Genome %
C57BL/6J and C57BL/6NTac	N/A	777	29.3%	28.5%
CBA/J	Homozygous	661	24.9%	24.7%
C57BL/6J and C57BL/6NTac X CBA/J	Heterozygous	1205	45.4%	46.5%
Unexplained	Homozygous	5	0.2%	0.1%
Unexplained	Heterozygous	8	0.3%	0.1%
	**Total**	2656	100.1%	99.9%

Y Chromosome

Not Applicable

MT Genome

MT Haplogroup 6 - 100.0% Consistent
Includes C57BL/6J, C57BL/6NTac, CBA/J and 165 other strains

MiniMUGA Background Analysis v2.3.1

Backgrounds Detected (Diagnostic Alleles)

Diagnostic Class	Diagnostic Alleles Observed: Homozygous	Heterozygous	Potential	% Observed
C57BL/6J, C57BL/6JJicTac, C57BL/6JRj	10	44	102	52.9%
C57BL/6J, C57BL/6JRj	3	8	31	35.5%
C57BL/6J, C57BL/6JEiJ, C57BL/6JJicTac, C57BL/6JRj	1	11	21	57.1%
C57BL/6NRj, C57BL/6NTac	3	4	15	46.7%
C57BL/6NJ, C57BL/6NRj, C57BL/6NTac	2	3	10	50.0%
129S5/SvEvBrd	0	1	5	20.0%
B6N-Tyr<c-Brd>/BrdCrCrl, C57BL/6J, C57BL/6JBomTac, C57BL/6JEiJ, C57BL/6JJicTac, C57BL/6JOlaHsd, C57BL/6JRj	0	1	2	50.0%
B6N-Tyr<c-Brd>/BrdCrCrl, C57BL/6J, C57BL/6JEiJ, C57BL/6JJicTac, C57BL/6JRj	0	1	1	100.0%
C57BL/6J, C57BL/6JEiJ, C57BL/6JJicTac, C57BL/6JOlaHsd, C57BL/6JRj	0	1	1	100.0%
C57BL/6NRj	0	1	10	10.0%

Minimal Strain Sets Explaining All Diagnostic Classes (Number of Markers Explained):

- Solution 1: 129S5/SvEvBrd and C57BL/6J and C57BL/6NRj
  - C57BL/6J: 80 / 158 (50.6%)
  - C57BL/6NRj: 13 / 35 (37.1%)
  - 129S5/SvEvBrd: 1 / 5 (20.0%)
- Solution 2: 129S5/SvEvBrd and C57BL/6JRj and C57BL/6NRj
  - C57BL/6JRj: 80 / 158 (50.6%)
  - C57BL/6NRj: 13 / 35 (37.1%)
  - 129S5/SvEvBrd: 1 / 5 (20.0%)

Chromosome	Start (Mb)	Stop (Mb)	Background	Zygosity
1	3000000	23043069	C57BL/6J and C57BL/6NTac and CBA/J	Heterozygous
1	23043069	53457225	CBA/J	Homozygous
1	53457225	69700765	C57BL/6J and C57BL/6NTac and CBA/J	Heterozygous
1	69700765	153548642	CBA/J	Homozygous
1	153548642	168019536	C57BL/6J and C57BL/6NTac and CBA/J	Heterozygous
1	168019536	195471971	C57BL/6J and C57BL/6NTac	N/A
2	3000000	115970567	C57BL/6J and C57BL/6NTac and CBA/J	Heterozygous
2	115970567	139631657	C57BL/6J and C57BL/6NTac	N/A
2	139631657	151062687	CBA/J	Homozygous
2	151062687	159275367	C57BL/6J and C57BL/6NTac and CBA/J	Heterozygous
2	159275367	182113224	CBA/J	Homozygous
3	3000000	30013882	C57BL/6J and C57BL/6NTac	N/A
3	30013882	139297311	C57BL/6J and C57BL/6NTac and CBA/J	Heterozygous
3	139297311	160039680	CBA/J	Homozygous

MiniMUGA Background Analysis v2.3.1

Diplotype Intervals

4	3000000	32327128	C57BL/6J and C57BL/6NTac and CBA/J	Heterozygous
4	32327128	35563307	C57BL/6J and C57BL/6NTac	N/A
4	35563307	37995481	Unexplained	Heterozygous
4	37995481	41348396	Unexplained	Homozygous
4	41348396	43372387	C57BL/6J and C57BL/6NTac and CBA/J	Heterozygous
4	43372387	43819249	Unexplained	Heterozygous
4	43819249	49280860	C57BL/6J and C57BL/6NTac and CBA/J	Heterozygous
4	49280860	54114833	C57BL/6J and C57BL/6NTac	N/A
4	54114833	120738488	C57BL/6J and C57BL/6NTac and CBA/J	Heterozygous
4	120738488	124400069	CBA/J	Homozygous
4	124400069	152440879	C57BL/6J and C57BL/6NTac and CBA/J	Heterozygous
4	152440879	156508116	C57BL/6J and C57BL/6NTac	N/A
5	3000000	14885741	C57BL/6J and C57BL/6NTac	N/A
5	14885741	51299144	C57BL/6J and C57BL/6NTac and CBA/J	Heterozygous
5	51299144	124446826	CBA/J	Homozygous
5	124446826	151834684	C57BL/6J and C57BL/6NTac and CBA/J	Heterozygous
6	3000000	79701235	C57BL/6J and C57BL/6NTac	N/A
6	79701235	101966063	C57BL/6J and C57BL/6NTac and CBA/J	Heterozygous
6	101966063	149736546	C57BL/6J and C57BL/6NTac	N/A
7	3000000	72944748	CBA/J	Homozygous
7	72944748	96169686	C57BL/6J and C57BL/6NTac and CBA/J	Heterozygous
7	96169686	103084424	CBA/J	Homozygous
7	103084424	116328796	C57BL/6J and C57BL/6NTac and CBA/J	Heterozygous
7	116328796	126580094	C57BL/6J and C57BL/6NTac	N/A
7	126580094	141750158	C57BL/6J and C57BL/6NTac and CBA/J	Heterozygous
7	141750158	145441459	C57BL/6J and C57BL/6NTac	N/A
8	3000000	45403996	C57BL/6J and C57BL/6NTac	N/A
8	45403996	125832225	C57BL/6J and C57BL/6NTac and CBA/J	Heterozygous
8	125832225	129401213	CBA/J	Homozygous
9	3000000	115715944	C57BL/6J and C57BL/6NTac	N/A
9	115715944	124595110	C57BL/6J and C57BL/6NTac and CBA/J	Heterozygous
10	3000000	42917049	C57BL/6J and C57BL/6NTac and CBA/J	Heterozygous

MiniMUGA Background Analysis v2.3.1

10	42917049	104861956	CBA/J	Homozygous
10	104861956	123114195	C57BL/6J and C57BL/6NTac and CBA/J	Heterozygous
10	123114195	130694993	CBA/J	Homozygous
11	3000000	22302070	CBA/J	Homozygous
11	22302070	29286327	C57BL/6J and C57BL/6NTac	N/A
11	29286327	58168384	C57BL/6J and C57BL/6NTac and CBA/J	Heterozygous
11	58168384	65400332	CBA/J	Homozygous
11	65400332	79617327	C57BL/6J and C57BL/6NTac and CBA/J	Heterozygous
11	79617327	90803561	C57BL/6J and C57BL/6NTac	N/A
11	90803561	122082543	C57BL/6J and C57BL/6NTac and CBA/J	Heterozygous
12	3000000	27585493	C57BL/6J and C57BL/6NTac	N/A
12	27585493	58069123	CBA/J	Homozygous
12	58069123	64411355	C57BL/6J and C57BL/6NTac and CBA/J	Heterozygous
12	64411355	120129022	CBA/J	Homozygous
13	3000000	26607981	CBA/J	Homozygous
13	26607981	60016573	C57BL/6J and C57BL/6NTac	N/A
13	60016573	120421639	C57BL/6J and C57BL/6NTac and CBA/J	Heterozygous
14	3000000	25112834	C57BL/6J and C57BL/6NTac and CBA/J	Heterozygous
14	25112834	70580779	CBA/J	Homozygous
14	70580779	97106405	C57BL/6J and C57BL/6NTac	N/A
14	97106405	111185375	C57BL/6J and C57BL/6NTac and CBA/J	Heterozygous
14	111185375	124902244	C57BL/6J and C57BL/6NTac	N/A
15	3000000	55016741	C57BL/6J and C57BL/6NTac and CBA/J	Heterozygous
15	55016741	104043685	C57BL/6J and C57BL/6NTac	N/A
16	3000000	29701002	CBA/J	Homozygous
16	29701002	42525074	C57BL/6J and C57BL/6NTac and CBA/J	Heterozygous
16	42525074	89037512	C57BL/6J and C57BL/6NTac	N/A
16	89037512	98207768	C57BL/6J and C57BL/6NTac and CBA/J	Heterozygous
17	3000000	61991666	C57BL/6J and C57BL/6NTac	N/A
17	61991666	94987271	C57BL/6J and C57BL/6NTac and CBA/J	Heterozygous
18	3000000	90702639	C57BL/6J and C57BL/6NTac and CBA/J	Heterozygous
19	3000000	37681034	C57BL/6J and C57BL/6NTac	N/A

MiniMUGA Background Analysis v2.3.1

	19	37681034	42582533	C57BL/6J and C57BL/6NTac and CBA/J	Heterozygous
	19	42582533	61431566	C57BL/6J and C57BL/6NTac	N/A
	X	3000000	70193631	C57BL/6J and C57BL/6NTac and CBA/J	Heterozygous
	X	70193631	84237192	C57BL/6J and C57BL/6NTac	N/A
	X	84237192	105020820	C57BL/6J and C57BL/6NTac and CBA/J	Heterozygous
	X	105020820	136441962	CBA/J	Homozygous
	X	136441962	171031299	C57BL/6J and C57BL/6NTac and CBA/J	Heterozygous
	MT	0	0	IBD	Hemizygous
