# Supplementary material for: Deletion of 9p drives B-ALL through heterozygous inactivation of Pax5 and Cd72 in preleukemic cells
Source: JCI Insight. 2026 Feb 17;11(7):e199464. doi: 10.1172/jci.insight.199464 (PMC13134721; doi:10.1172/jci.insight.199464)
Supplement: Supplemental data set 1 [file jciinsight-11-199464-s204.zip › Strain_Genotyping/G524-results-report.pdf]

MiniMUGA Background Analysis v2.3.1

Sample ID: G524

Neogen ID: AAAU-5383

Summary:

The genotype of this sample is of **excellent** quality. It is **female** and **outbred**, and likely a mix of **C57BL/6J and C57BL/6NTac** and **CBA/J**. Clustering of unexplained markers is evidence of an additional background strain.

Diagnostic SNPs are likely explained by the presence of the background strains

- Solution 1: 129S5/SvEvBrd and C57BL/6J and C57BL/6NTac
  - C57BL/6J: 58 / 155 (37.4%)
  - C57BL/6NTac: 13 / 27 (48.1%)
  - 129S5/SvEvBrd: 1 / 5 (20.0%)
- Solution 2: 129S5/SvEvBrd and C57BL/6J and C57BL/6NRj
  - C57BL/6J: 58 / 155 (37.4%)
  - C57BL/6NRj: 13 / 27 (48.1%)
  - 129S5/SvEvBrd: 1 / 5 (20.0%)
- Solution 3: 129S5/SvEvBrd and C57BL/6JRj and C57BL/6NTac
  - C57BL/6JRj: 58 / 155 (37.4%)
  - C57BL/6NTac: 13 / 27 (48.1%)
  - 129S5/SvEvBrd: 1 / 5 (20.0%)
- Solution 4: 129S5/SvEvBrd and C57BL/6JRj and C57BL/6NRj
  - C57BL/6JRj: 58 / 155 (37.4%)
  - C57BL/6NRj: 13 / 27 (48.1%)
  - 129S5/SvEvBrd: 1 / 5 (20.0%)

NOTE: There is a discrepancy between the diagnostic backgrounds detected and the primary and secondary background analysis (C57BL/6J, CBA/J, C57BL/6NTac). This is uncommon and should be investigated further.

No genetic constructs were detected in this sample.

WARNING:

- There is a discrepancy between the diagnostic backgrounds detected ((129S5/SvEvBrd and C57BL/6J and C57BL/6NTac) or (129S5/SvEvBrd and C57BL/6J and C57BL/6NRj) or (129S5/SvEvBrd and C57BL/6JRj and C57BL/6NTac) or (129S5/SvEvBrd and C57BL/6JRj and C57BL/6NRj)) and the primary background (C57BL/6J and C57BL/6NTac) and secondary background (CBA/J). This is uncommon and should be investigated further.
- The presence of a single diagnostic heterozygous call for a single inbred strain should be treated with caution.
- This sample likely has more than 2 genetic backgrounds (unexplained regions and/or fractured ideogram). The strain selected for secondary background may be incorrect. The estimation of the contribution of primary and secondary background are likely incorrect. This can potentially be addressed with input from the user.

Genotyping Quality:

Excellent (3 N calls)
All reported results are dependent on genotyping quality.

Chromosomal Sex: XX

Inbreeding Estimate:

72.9% Inbred
(Percentage of the genome (autosomal and X chromosomes) that is homozygous or hemizygous for primary, secondary, and unknown backgrounds. See Genome Analysis)

Constructs Detected:

BlastR	bpA	Cas9	chlor	cHS4	Cre	DTA	Flp	g_FP	hCMV_a	hCMV_b	hTK_pr	iCre	IRES	Luc	r_FP	rtTA	SV40	tTA
-	-	-	-	-	-	-	-	-	-	-	-	-	-	-	-	-	-	-

MiniMUGA Background Analysis v2.3.1

Refined Ideogram

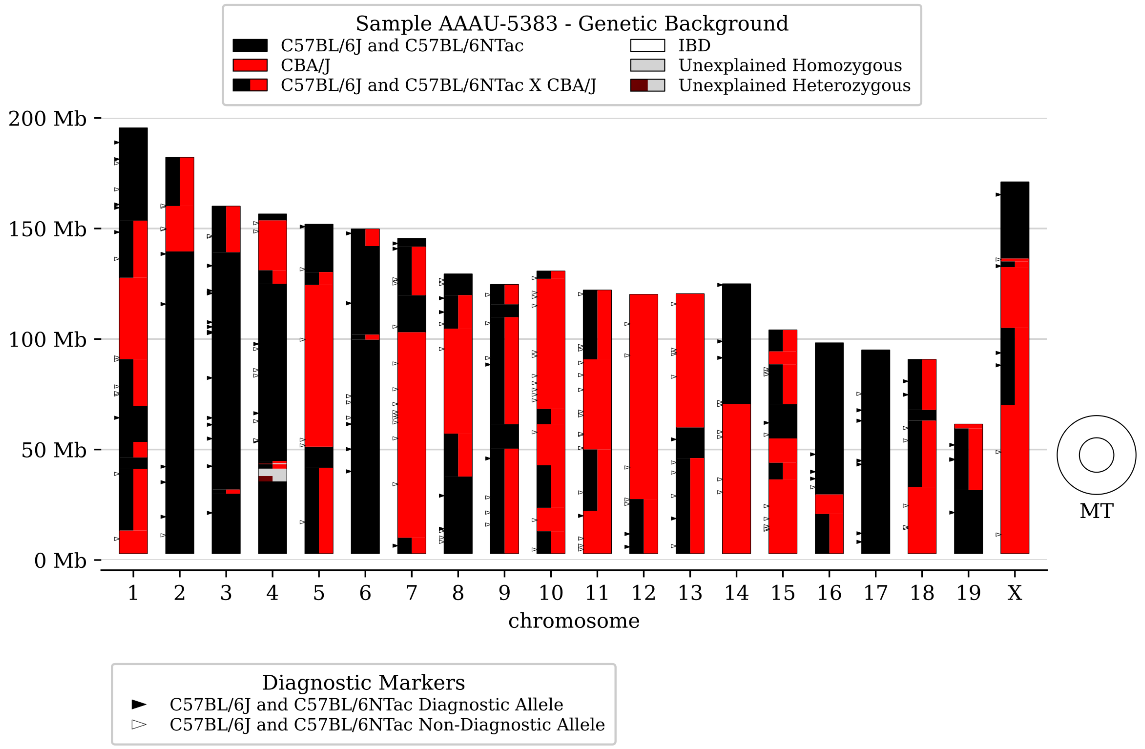

Genome Analysis

Background	Zygosity	Informative Markers	Informative Markers %	Genome %
C57BL/6J and C57BL/6NTac	N/A	974	36.4%	39.3%
CBA/J	Homozygous	956	35.7%	33.4%
C57BL/6J and C57BL/6NTac X CBA/J	Heterozygous	736	27.5%	27.0%
Unexplained	Homozygous	5	0.2%	0.1%
Unexplained	Heterozygous	5	0.2%	0.1%
	**Total**	2676	100.0%	99.9%

Y Chromosome

Not Applicable

MT Genome

MT Haplogroup 6 - 100.0% Consistent
Includes C57BL/6J, C57BL/6NTac, CBA/J and 165 other strains

MiniMUGA Background Analysis v2.3.1

Backgrounds Detected (Diagnostic Alleles)

Diagnostic Class	Diagnostic Alleles Observed: Homozygous	Heterozygous	Potential	% Observed
C57BL/6J, C57BL/6JJicTac, C57BL/6JRj	19	16	102	34.3%
C57BL/6J, C57BL/6JEiJ, C57BL/6JJicTac, C57BL/6JRj	9	3	21	57.1%
C57BL/6J, C57BL/6JRj	5	5	31	32.3%
C57BL/6NRj, C57BL/6NTac	0	7	15	46.7%
C57BL/6NJ, C57BL/6NRj, C57BL/6NTac	1	4	10	50.0%
B6N-Tyr<c-Brd>/BrdCrCrl, C57BL/6J, C57BL/6JEiJ, C57BL/6JJicTac, C57BL/6JRj	1	0	1	100.0%
129S5/SvEvBrd	0	1	5	20.0%
B6N-Tyr<c-Brd>/BrdCrCrl, C57BL/6NCrl, C57BL/6NHsd, C57BL/6NJ, C57BL/6NRj, C57BL/6NTac	0	1	2	50.0%

Minimal Strain Sets Explaining All Diagnostic Classes (Number of Markers Explained):

- Solution 1: 129S5/SvEvBrd and C57BL/6J and C57BL/6NTac
  - C57BL/6J: 58 / 155 (37.4%)
  - C57BL/6NTac: 13 / 27 (48.1%)
  - 129S5/SvEvBrd: 1 / 5 (20.0%)
- Solution 2: 129S5/SvEvBrd and C57BL/6J and C57BL/6NRj
  - C57BL/6J: 58 / 155 (37.4%)
  - C57BL/6NRj: 13 / 27 (48.1%)
  - 129S5/SvEvBrd: 1 / 5 (20.0%)
- Solution 3: 129S5/SvEvBrd and C57BL/6JRj and C57BL/6NTac
  - C57BL/6JRj: 58 / 155 (37.4%)
  - C57BL/6NTac: 13 / 27 (48.1%)
  - 129S5/SvEvBrd: 1 / 5 (20.0%)
- Solution 4: 129S5/SvEvBrd and C57BL/6JRj and C57BL/6NRj
  - C57BL/6JRj: 58 / 155 (37.4%)
  - C57BL/6NRj: 13 / 27 (48.1%)
  - 129S5/SvEvBrd: 1 / 5 (20.0%)

Chromosome	Start (Mb)	Stop (Mb)	Background	Zygosity
1	3000000	13365974	CBA/J	Homozygous
1	13365974	41199760	C57BL/6J and C57BL/6NTac and CBA/J	Heterozygous
1	41199760	46434628	C57BL/6J and C57BL/6NTac	N/A
1	46434628	53457225	C57BL/6J and C57BL/6NTac and CBA/J	Heterozygous
1	53457225	69700765	C57BL/6J and C57BL/6NTac	N/A
1	69700765	90903197	C57BL/6J and C57BL/6NTac and CBA/J	Heterozygous
1	90903197	127744193	CBA/J	Homozygous
1	127744193	153548642	C57BL/6J and C57BL/6NTac and CBA/J	Heterozygous
1	153548642	195471971	C57BL/6J and C57BL/6NTac	N/A
2	3000000	139631657	C57BL/6J and C57BL/6NTac	N/A
2	139631657	160174252	CBA/J	Homozygous

MiniMUGA Background Analysis v2.3.1

**Diplotype Intervals**	2	160174252	182113224	C57BL/6J and C57BL/6NTac and CBA/J	Heterozygous
	3	3000000	30013882	C57BL/6J and C57BL/6NTac	N/A
	3	30013882	31868563	C57BL/6J and C57BL/6NTac and CBA/J	Heterozygous
	3	31868563	139297311	C57BL/6J and C57BL/6NTac	N/A
	3	139297311	160039680	C57BL/6J and C57BL/6NTac and CBA/J	Heterozygous
	4	3000000	35563307	C57BL/6J and C57BL/6NTac	N/A
	4	35563307	37995481	Unexplained	Heterozygous
	4	37995481	41348396	Unexplained	Homozygous
	4	41348396	43372387	C57BL/6J and C57BL/6NTac and CBA/J	Heterozygous
	4	43372387	43819249	Unexplained	Heterozygous
	4	43819249	44820333	C57BL/6J and C57BL/6NTac and CBA/J	Heterozygous
	4	44820333	124990087	C57BL/6J and C57BL/6NTac	N/A
	4	124990087	131104093	C57BL/6J and C57BL/6NTac and CBA/J	Heterozygous
	4	131104093	153688585	CBA/J	Homozygous
	4	153688585	156508116	C57BL/6J and C57BL/6NTac	N/A
	5	3000000	41755530	C57BL/6J and C57BL/6NTac and CBA/J	Heterozygous
	5	41755530	51299144	C57BL/6J and C57BL/6NTac	N/A
	5	51299144	124446826	CBA/J	Homozygous
	5	124446826	130280923	C57BL/6J and C57BL/6NTac and CBA/J	Heterozygous
	5	130280923	151834684	C57BL/6J and C57BL/6NTac	N/A
	6	3000000	99727922	C57BL/6J and C57BL/6NTac	N/A
	6	99727922	101966063	C57BL/6J and C57BL/6NTac and CBA/J	Heterozygous
	6	101966063	142043514	C57BL/6J and C57BL/6NTac	N/A
	6	142043514	149736546	C57BL/6J and C57BL/6NTac and CBA/J	Heterozygous
	7	3000000	10069735	C57BL/6J and C57BL/6NTac and CBA/J	Heterozygous
	7	10069735	103084424	CBA/J	Homozygous
	7	103084424	119823617	C57BL/6J and C57BL/6NTac	N/A
	7	119823617	141750158	C57BL/6J and C57BL/6NTac and CBA/J	Heterozygous
	7	141750158	145441459	C57BL/6J and C57BL/6NTac	N/A
	8	3000000	37790271	C57BL/6J and C57BL/6NTac	N/A
	8	37790271	57187999	C57BL/6J and C57BL/6NTac and CBA/J	Heterozygous

MiniMUGA Background Analysis v2.3.1

8	57187999	104681042	CBA/J	Homozygous
8	104681042	119835722	C57BL/6J and C57BL/6NTac and CBA/J	Heterozygous
8	119835722	129401213	C57BL/6J and C57BL/6NTac	N/A
9	3000000	50381732	C57BL/6J and C57BL/6NTac and CBA/J	Heterozygous
9	50381732	61484305	C57BL/6J and C57BL/6NTac	N/A
9	61484305	109855467	C57BL/6J and C57BL/6NTac and CBA/J	Heterozygous
9	109855467	115715944	C57BL/6J and C57BL/6NTac	N/A
9	115715944	124595110	C57BL/6J and C57BL/6NTac and CBA/J	Heterozygous
10	3000000	12999693	C57BL/6J and C57BL/6NTac and CBA/J	Heterozygous
10	12999693	23654421	CBA/J	Homozygous
10	23654421	42858234	C57BL/6J and C57BL/6NTac and CBA/J	Heterozygous
10	42858234	61450853	CBA/J	Homozygous
10	61450853	68332199	C57BL/6J and C57BL/6NTac and CBA/J	Heterozygous
10	68332199	127271560	CBA/J	Homozygous
10	127271560	130694993	C57BL/6J and C57BL/6NTac and CBA/J	Heterozygous
11	3000000	22302070	CBA/J	Homozygous
11	22302070	50018951	C57BL/6J and C57BL/6NTac and CBA/J	Heterozygous
11	50018951	90803561	CBA/J	Homozygous
11	90803561	122082543	C57BL/6J and C57BL/6NTac and CBA/J	Heterozygous
12	3000000	27585493	C57BL/6J and C57BL/6NTac and CBA/J	Heterozygous
12	27585493	120129022	CBA/J	Homozygous
13	3000000	46136691	C57BL/6J and C57BL/6NTac and CBA/J	Heterozygous
13	46136691	60016573	C57BL/6J and C57BL/6NTac	N/A
13	60016573	120421639	CBA/J	Homozygous
14	3000000	70580779	CBA/J	Homozygous
14	70580779	124902244	C57BL/6J and C57BL/6NTac	N/A
15	3000000	36473640	CBA/J	Homozygous
15	36473640	44010563	C57BL/6J and C57BL/6NTac and CBA/J	Heterozygous
15	44010563	55016741	CBA/J	Homozygous
15	55016741	70554147	C57BL/6J and C57BL/6NTac	N/A
15	70554147	88538882	C57BL/6J and C57BL/6NTac and CBA/J	Heterozygous
15	88538882	94412127	CBA/J	Homozygous
15	94412127	104043685	C57BL/6J and C57BL/6NTac and CBA/J	Heterozygous

MiniMUGA Background Analysis v2.3.1

	16	3000000	20813513	C57BL/6J and C57BL/6NTac and CBA/J	Heterozygous
	16	20813513	29701002	CBA/J	Homozygous
	16	29701002	98207768	C57BL/6J and C57BL/6NTac	N/A
	17	3000000	94987271	C57BL/6J and C57BL/6NTac	N/A
	18	3000000	33050504	CBA/J	Homozygous
	18	33050504	63069205	C57BL/6J and C57BL/6NTac and CBA/J	Heterozygous
	18	63069205	67937187	C57BL/6J and C57BL/6NTac	N/A
	18	67937187	90702639	C57BL/6J and C57BL/6NTac and CBA/J	Heterozygous
	19	3000000	31636352	C57BL/6J and C57BL/6NTac	N/A
	19	31636352	59526280	C57BL/6J and C57BL/6NTac and CBA/J	Heterozygous
	19	59526280	61431566	CBA/J	Homozygous
	X	3000000	70193631	CBA/J	Homozygous
	X	70193631	105020820	C57BL/6J and C57BL/6NTac and CBA/J	Heterozygous
	X	105020820	132528229	CBA/J	Homozygous
	X	132528229	135099309	C57BL/6J and C57BL/6NTac and CBA/J	Heterozygous
	X	135099309	136441962	CBA/J	Homozygous
	X	136441962	171031299	C57BL/6J and C57BL/6NTac	N/A
	MT	0	0	IBD	Hemizygous
