# Supplementary material for: Deletion of 9p drives B-ALL through heterozygous inactivation of Pax5 and Cd72 in preleukemic cells
Source: JCI Insight. 2026 Feb 17;11(7):e199464. doi: 10.1172/jci.insight.199464 (PMC13134721; doi:10.1172/jci.insight.199464)
Supplement: Supplemental data set 1 [file jciinsight-11-199464-s204.zip › Strain_Genotyping/G531-results-report.pdf]

MiniMUGA Background Analysis v2.3.1

**Sample ID**	G531
**Neogen ID**	AAAU-5385
**Summary**	The genotype of this sample is of **excellent** quality. It is **female** and **outbred**, and likely a mix of **C57BL/6J and C57BL/6NTac** and **CBA/J**. Clustering of unexplained markers is evidence of an additional background strain.<br><br>Diagnostic SNPs are likely explained by the presence of the background strains<br>• Solution 1: 129S5/SvEvBrd and C57BL/6J and C57BL/6NTac<br>◦ C57BL/6J: 59 / 155 (38.1%)<br>◦ C57BL/6NTac: 13 / 29 (44.8%)<br>◦ 129S5/SvEvBrd: 1 / 5 (20.0%)<br>• Solution 2: 129S5/SvEvBrd and C57BL/6J and C57BL/6NRj<br>◦ C57BL/6J: 59 / 155 (38.1%)<br>◦ C57BL/6NRj: 13 / 29 (44.8%)<br>◦ 129S5/SvEvBrd: 1 / 5 (20.0%)<br>• Solution 3: 129S5/SvEvBrd and C57BL/6JRj and C57BL/6NTac<br>◦ C57BL/6JRj: 59 / 155 (38.1%)<br>◦ C57BL/6NTac: 13 / 29 (44.8%)<br>◦ 129S5/SvEvBrd: 1 / 5 (20.0%)<br>• Solution 4: 129S5/SvEvBrd and C57BL/6JRj and C57BL/6NRj<br>◦ C57BL/6JRj: 59 / 155 (38.1%)<br>◦ C57BL/6NRj: 13 / 29 (44.8%)<br>◦ 129S5/SvEvBrd: 1 / 5 (20.0%)<br><br>NOTE: There is a discrepancy between the diagnostic backgrounds detected and the primary and secondary background analysis (C57BL/6J, CBA/J, C57BL/6NTac). This is uncommon and should be investigated further.<br><br>No genetic constructs were detected in this sample.<br><br>WARNING:<br>• There is a discrepancy between the diagnostic backgrounds detected ((129S5/SvEvBrd and C57BL/6J and C57BL/6NTac) or (129S5/SvEvBrd and C57BL/6J and C57BL/6NRj) or (129S5/SvEvBrd and C57BL/6JRj and C57BL/6NTac) or (129S5/SvEvBrd and C57BL/6JRj and C57BL/6NRj)) and the primary background (C57BL/6J and C57BL/6NTac) and secondary background (CBA/J). This is uncommon and should be investigated further.<br>• The presence of a single diagnostic heterozygous call for a single inbred strain should be treated with caution.<br>• This sample likely has more than 2 genetic backgrounds (unexplained regions and/or fractured ideogram). The strain selected for secondary background may be incorrect. The estimation of the contribution of primary and secondary background are likely incorrect. This can potentially be addressed with input from the user.
**Genotyping Quality**	**Excellent (1 N calls)**<br>All reported results are dependent on genotyping quality.
**Chromosomal Sex**	XX
**Inbreeding Estimate**	69.7% Inbred<br>(Percentage of the genome (autosomal and X chromosomes) that is homozygous or hemizygous for primary, secondary, and unknown backgrounds. See Genome Analysis)
**Constructs Detected**	see below

Constructs Detected

BlastR	bpA	Cas9	chlor	cHS4	Cre	DTA	Flp	g_FP	hCMV_a	hCMV_b	hTK_pr	iCre	IRES	Luc	r_FP	rtTA	SV40	tTA
-	-	-	-	-	-	-	-	-	-	-	-	-	-	-	-	-	-	-

MiniMUGA Background Analysis v2.3.1

Refined Ideogram

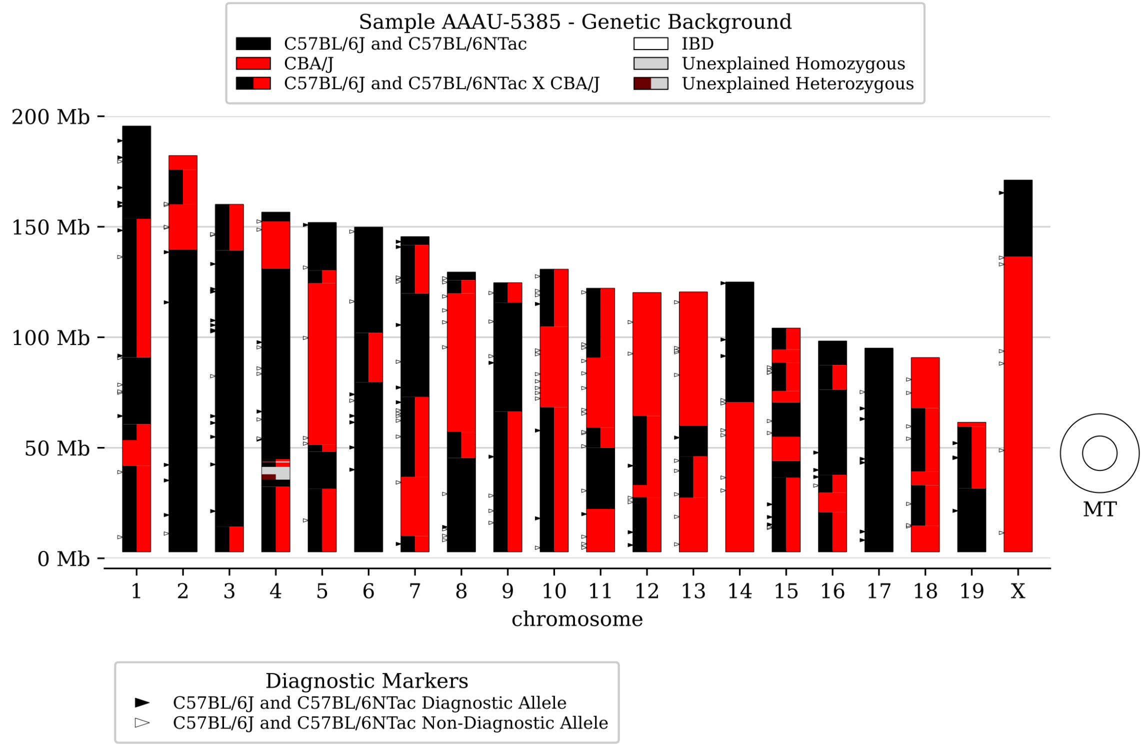

Genome Analysis

Background	Zygosity	Informative Markers	Informative Markers %	Genome %
C57BL/6J and C57BL/6NTac	N/A	1039	38.8%	41.2%
CBA/J	Homozygous	848	31.7%	28.4%
C57BL/6J and C57BL/6NTac X CBA/J	Heterozygous	779	29.1%	30.2%
Unexplained	Homozygous	5	0.2%	0.1%
Unexplained	Heterozygous	6	0.2%	0.1%
	**Total**	2677	100.0%	100.0%

**Y Chromosome**	Not Applicable
**MT Genome**	MT Haplogroup 6 - 100.0% Consistent<br>Includes C57BL/6J, C57BL/6NTac, CBA/J and 165 other strains

MiniMUGA Background Analysis v2.3.1

Backgrounds Detected (Diagnostic Alleles)

Diagnostic Class	Diagnostic Alleles Observed: Homozygous	Heterozygous	Potential	% Observed
C57BL/6J, C57BL/6JJicTac, C57BL/6JRj	20	18	102	37.3%
C57BL/6J, C57BL/6JEiJ, C57BL/6JJicTac, C57BL/6JRj	7	3	21	47.6%
C57BL/6J, C57BL/6JRj	4	6	31	32.3%
C57BL/6NJ, C57BL/6NRj, C57BL/6NTac	3	2	10	50.0%
C57BL/6NRj, C57BL/6NTac	0	6	15	40.0%
B6N-Tyr<c-Brd>/BrdCrCrl, C57BL/6J, C57BL/6JEiJ, C57BL/6JJicTac, C57BL/6JRj	1	0	1	100.0%
129S5/SvEvBrd	0	1	5	20.0%
B6N-Tyr<c-Brd>/BrdCrCrl, C57BL/6NCrl, C57BL/6NHsd, C57BL/6NJ, C57BL/6NRj, C57BL/6NTac	0	1	2	50.0%
C57BL/6NCrl, C57BL/6NHsd, C57BL/6NJ, C57BL/6NRj, C57BL/6NTac	0	1	2	50.0%

Minimal Strain Sets Explaining All Diagnostic Classes (Number of Markers Explained):

- Solution 1: 129S5/SvEvBrd and C57BL/6J and C57BL/6NTac
  - C57BL/6J: 59 / 155 (38.1%)
  - C57BL/6NTac: 13 / 29 (44.8%)
  - 129S5/SvEvBrd: 1 / 5 (20.0%)
- Solution 2: 129S5/SvEvBrd and C57BL/6J and C57BL/6NRj
  - C57BL/6J: 59 / 155 (38.1%)
  - C57BL/6NRj: 13 / 29 (44.8%)
  - 129S5/SvEvBrd: 1 / 5 (20.0%)
- Solution 3: 129S5/SvEvBrd and C57BL/6JRj and C57BL/6NTac
  - C57BL/6JRj: 59 / 155 (38.1%)
  - C57BL/6NTac: 13 / 29 (44.8%)
  - 129S5/SvEvBrd: 1 / 5 (20.0%)
- Solution 4: 129S5/SvEvBrd and C57BL/6JRj and C57BL/6NRj
  - C57BL/6JRj: 59 / 155 (38.1%)
  - C57BL/6NRj: 13 / 29 (44.8%)
  - 129S5/SvEvBrd: 1 / 5 (20.0%)

Chromosome	Start (Mb)	Stop (Mb)	Background	Zygosity
1	3000000	41869819	C57BL/6J and C57BL/6NTac and CBA/J	Heterozygous
1	41869819	53457225	CBA/J	Homozygous
1	53457225	60621237	C57BL/6J and C57BL/6NTac and CBA/J	Heterozygous
1	60621237	90903197	C57BL/6J and C57BL/6NTac	N/A
1	90903197	153548642	C57BL/6J and C57BL/6NTac and CBA/J	Heterozygous
1	153548642	195471971	C57BL/6J and C57BL/6NTac	N/A
2	3000000	139631657	C57BL/6J and C57BL/6NTac	N/A
2	139631657	160174252	CBA/J	Homozygous
2	160174252	175780822	C57BL/6J and C57BL/6NTac and CBA/J	Heterozygous
2	175780822	182113224	CBA/J	Homozygous

**Diplotype Intervals**	3	3000000	14328941	C57BL/6J and C57BL/6NTac and CBA/J	Heterozygous
	3	14328941	139297311	C57BL/6J and C57BL/6NTac	N/A
	3	139297311	160039680	C57BL/6J and C57BL/6NTac and CBA/J	Heterozygous
	4	3000000	32327128	C57BL/6J and C57BL/6NTac and CBA/J	Heterozygous
	4	32327128	35563307	C57BL/6J and C57BL/6NTac	N/A
	4	35563307	37995481	Unexplained	Heterozygous
	4	37995481	41348396	Unexplained	Homozygous
	4	41348396	43372387	C57BL/6J and C57BL/6NTac and CBA/J	Heterozygous
	4	43372387	43819249	Unexplained	Heterozygous
	4	43819249	44820333	C57BL/6J and C57BL/6NTac and CBA/J	Heterozygous
	4	44820333	131104093	C57BL/6J and C57BL/6NTac	N/A
	4	131104093	152440879	CBA/J	Homozygous
	4	152440879	156508116	C57BL/6J and C57BL/6NTac	N/A
	5	3000000	31408123	C57BL/6J and C57BL/6NTac and CBA/J	Heterozygous
	5	31408123	48265431	C57BL/6J and C57BL/6NTac	N/A
	5	48265431	51299144	C57BL/6J and C57BL/6NTac and CBA/J	Heterozygous
	5	51299144	124446826	CBA/J	Homozygous
	5	124446826	130280923	C57BL/6J and C57BL/6NTac and CBA/J	Heterozygous
	5	130280923	151834684	C57BL/6J and C57BL/6NTac	N/A
	6	3000000	79701235	C57BL/6J and C57BL/6NTac	N/A
	6	79701235	101966063	C57BL/6J and C57BL/6NTac and CBA/J	Heterozygous
	6	101966063	149736546	C57BL/6J and C57BL/6NTac	N/A
	7	3000000	10069735	C57BL/6J and C57BL/6NTac and CBA/J	Heterozygous
	7	10069735	36856023	CBA/J	Homozygous
	7	36856023	72944748	C57BL/6J and C57BL/6NTac and CBA/J	Heterozygous
	7	72944748	119823617	C57BL/6J and C57BL/6NTac	N/A
	7	119823617	141750158	C57BL/6J and C57BL/6NTac and CBA/J	Heterozygous
	7	141750158	145441459	C57BL/6J and C57BL/6NTac	N/A
	8	3000000	45403996	C57BL/6J and C57BL/6NTac	N/A
	8	45403996	57187999	C57BL/6J and C57BL/6NTac and CBA/J	Heterozygous
	8	57187999	119835722	CBA/J	Homozygous

MiniMUGA Background Analysis v2.3.1

	8	119835722	125832225	C57BL/6J and C57BL/6NTac and CBA/J	Heterozygous
	8	125832225	129401213	C57BL/6J and C57BL/6NTac	N/A
	9	3000000	66341356	C57BL/6J and C57BL/6NTac and CBA/J	Heterozygous
	9	66341356	115715944	C57BL/6J and C57BL/6NTac	N/A
	9	115715944	124595110	C57BL/6J and C57BL/6NTac and CBA/J	Heterozygous
	10	3000000	68332199	C57BL/6J and C57BL/6NTac and CBA/J	Heterozygous
	10	68332199	104861956	CBA/J	Homozygous
	10	104861956	130694993	C57BL/6J and C57BL/6NTac and CBA/J	Heterozygous
	11	3000000	22302070	CBA/J	Homozygous
	11	22302070	50018951	C57BL/6J and C57BL/6NTac	N/A
	11	50018951	59127711	C57BL/6J and C57BL/6NTac and CBA/J	Heterozygous
	11	59127711	90803561	CBA/J	Homozygous
	11	90803561	122082543	C57BL/6J and C57BL/6NTac and CBA/J	Heterozygous
	12	3000000	27585493	C57BL/6J and C57BL/6NTac and CBA/J	Heterozygous
	12	27585493	33130555	CBA/J	Homozygous
	12	33130555	64411355	C57BL/6J and C57BL/6NTac and CBA/J	Heterozygous
	12	64411355	120129022	CBA/J	Homozygous
	13	3000000	27430346	CBA/J	Homozygous
	13	27430346	46136691	C57BL/6J and C57BL/6NTac and CBA/J	Heterozygous
	13	46136691	60016573	C57BL/6J and C57BL/6NTac	N/A
	13	60016573	120421639	CBA/J	Homozygous
	14	3000000	70580779	CBA/J	Homozygous
	14	70580779	124902244	C57BL/6J and C57BL/6NTac	N/A
	15	3000000	36473640	C57BL/6J and C57BL/6NTac and CBA/J	Heterozygous
	15	36473640	44010563	C57BL/6J and C57BL/6NTac	N/A
	15	44010563	55016741	CBA/J	Homozygous
	15	55016741	70554147	C57BL/6J and C57BL/6NTac	N/A
	15	70554147	75654766	CBA/J	Homozygous
	15	75654766	88538882	C57BL/6J and C57BL/6NTac and CBA/J	Heterozygous
	15	88538882	94412127	CBA/J	Homozygous
	15	94412127	104043685	C57BL/6J and C57BL/6NTac and CBA/J	Heterozygous
	16	3000000	20813513	C57BL/6J and C57BL/6NTac and CBA/J	Heterozygous
	16	20813513	29701002	CBA/J	Homozygous

MiniMUGA Background Analysis v2.3.1

	16	29701002	37740023	C57BL/6J and C57BL/6NTac and CBA/J	Heterozygous
	16	37740023	76315797	C57BL/6J and C57BL/6NTac	N/A
	16	76315797	87403166	C57BL/6J and C57BL/6NTac and CBA/J	Heterozygous
	16	87403166	98207768	C57BL/6J and C57BL/6NTac	N/A
	17	3000000	94987271	C57BL/6J and C57BL/6NTac	N/A
	18	3000000	14753212	CBA/J	Homozygous
	18	14753212	33050504	C57BL/6J and C57BL/6NTac and CBA/J	Heterozygous
	18	33050504	39285932	CBA/J	Homozygous
	18	39285932	67937187	C57BL/6J and C57BL/6NTac and CBA/J	Heterozygous
	18	67937187	90702639	CBA/J	Homozygous
	19	3000000	31636352	C57BL/6J and C57BL/6NTac	N/A
	19	31636352	59526280	C57BL/6J and C57BL/6NTac and CBA/J	Heterozygous
	19	59526280	61431566	CBA/J	Homozygous
	X	3000000	136441962	CBA/J	Homozygous
	X	136441962	171031299	C57BL/6J and C57BL/6NTac	N/A
	MT	0	0	IBD	Hemizygous
