# Supplementary material for: Deletion of 9p drives B-ALL through heterozygous inactivation of Pax5 and Cd72 in preleukemic cells
Source: JCI Insight. 2026 Feb 17;11(7):e199464. doi: 10.1172/jci.insight.199464 (PMC13134721; doi:10.1172/jci.insight.199464)
Supplement: Supplemental data set 1 [file jciinsight-11-199464-s204.zip › Strain_Genotyping/M229-results-report.pdf]

MiniMUGA Background Analysis v2.3.1

**Sample ID**	M229
**Neogen ID**	AAAU-4519
**Summary**	The genotype of this sample is of **excellent** quality. It is **female** and **outbred**, and likely a mix of **C57BL/6J and C57BL/6NTac** and **CBA/J**. Clustering of unexplained markers is evidence of an additional background strain.<br><br>Diagnostic SNPs are likely explained by the presence of the background strains<br>• Solution 1: 129S5/SvEvBrd and C57BL/6J and C57BL/6NRj<br>◦ C57BL/6J: 49 / 157 (31.2%)<br>◦ C57BL/6NRj: 22 / 40 (55.0%)<br>◦ 129S5/SvEvBrd: 1 / 5 (20.0%)<br>• Solution 2: 129S5/SvEvBrd and C57BL/6JRj and C57BL/6NRj<br>◦ C57BL/6JRj: 49 / 157 (31.2%)<br>◦ C57BL/6NRj: 22 / 40 (55.0%)<br>◦ 129S5/SvEvBrd: 1 / 5 (20.0%)<br><br>NOTE: There is a discrepancy between the diagnostic backgrounds detected and the primary and secondary background analysis (CBA/J, C57BL/6J, C57BL/6NTac). This is uncommon and should be investigated further.<br><br>No genetic constructs were detected in this sample.<br><br>WARNING:<br>• There is a discrepancy between the diagnostic backgrounds detected ((129S5/SvEvBrd and C57BL/6J and C57BL/6NRj) or (129S5/SvEvBrd and C57BL/6JRj and C57BL/6NRj)) and the primary background (C57BL/6J and C57BL/6NTac) and secondary background (CBA/J). This is uncommon and should be investigated further.<br>• The presence of a single diagnostic heterozygous call for a single inbred strain should be treated with caution.<br>• This sample likely has more than 2 genetic backgrounds (unexplained regions and/or fractured ideogram). The strain selected for secondary background may be incorrect. The estimation of the contribution of primary and secondary background are likely incorrect. This can potentially be addressed with input from the user.
**Genotyping Quality**	**Excellent (13 N calls)**<br>All reported results are dependent on genotyping quality.
**Chromosomal Sex**	XX
**Inbreeding Estimate**	57.7% Inbred<br>(Percentage of the genome (autosomal and X chromosomes) that is homozygous or hemizygous for primary, secondary, and unknown backgrounds. See Genome Analysis)

Constructs Detected

BlastR	bpA	Cas9	chlor	cHS4	Cre	DTA	Flp	g_FP	hCMV_a	hCMV_b	hTK_pr	iCre	IRES	Luc	r_FP	rtTA	SV40	tTA
-	-	-	-	-	-	-	-	-	-	-	-	-	-	-	-	-	-	-

MiniMUGA Background Analysis v2.3.1

Refined Ideogram

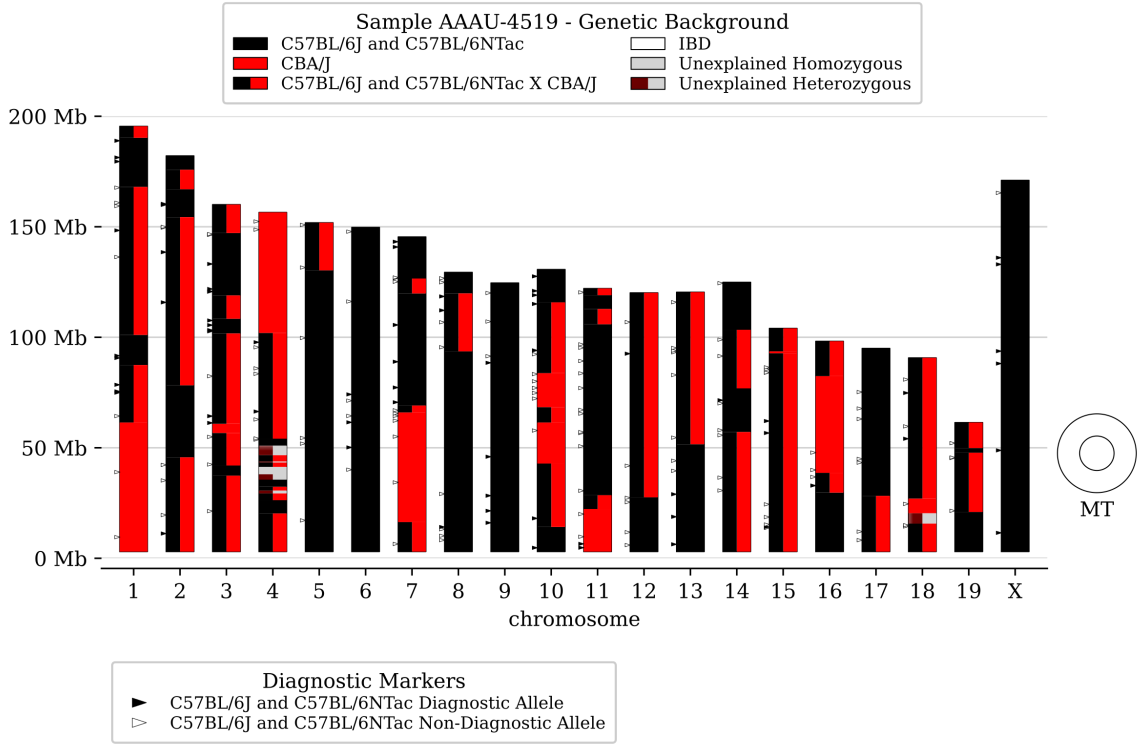

Genome Analysis

Background	Zygosity	Informative Markers	Informative Markers %	Genome %
C57BL/6J and C57BL/6NTac	N/A	1223	45.9%	47.0%
CBA/J	Homozygous	231	8.7%	10.6%
C57BL/6J and C57BL/6NTac X CBA/J	Heterozygous	1189	44.6%	41.8%
Unexplained	Homozygous	5	0.2%	0.1%
Unexplained	Heterozygous	19	0.7%	0.5%
	**Total**	2667	100.1%	100.0%

Y Chromosome

Not Applicable

MT Genome

MT Haplogroup 6 - 100.0% Consistent
Includes C57BL/6J, C57BL/6NTac, CBA/J and 165 other strains

MiniMUGA Background Analysis v2.3.1

Backgrounds Detected (Diagnostic Alleles)

	Diagnostic Alleles Observed			
**Diagnostic Class**	**Homozygous**	**Heterozygous**	**Potential**	**% Observed**
C57BL/6J, C57BL/6JJicTac, C57BL/6JRj	7	27	102	33.3%
C57BL/6J, C57BL/6JEiJ, C57BL/6JJicTac, C57BL/6JRj	4	4	21	38.1%
C57BL/6NJ, C57BL/6NRj, C57BL/6NTac	3	4	10	70.0%
C57BL/6NRj, C57BL/6NTac	1	8	15	60.0%
C57BL/6J, C57BL/6JRj	1	4	31	16.1%
B6N-Tyr<c-Brd>/BrdCrCrl, C57BL/6NCrl, C57BL/6NHsd, C57BL/6NJ, C57BL/6NRj, C57BL/6NTac	1	0	2	50.0%
C57BL/6NCrl, C57BL/6NHsd, C57BL/6NJ, C57BL/6NRj, C57BL/6NTac	0	2	2	100.0%
C57BL/6NRj	0	2	10	20.0%
129S5/SvEvBrd	0	1	5	20.0%
B6N-Tyr<c-Brd>/BrdCrCrl, C57BL/6J, C57BL/6JEiJ, C57BL/6JJicTac, C57BL/6JRj	0	1	1	100.0%
C57BL/6J, C57BL/6JBomTac, C57BL/6JEiJ, C57BL/6JJicTac, C57BL/6JOlaHsd, C57BL/6JRj	0	1	2	50.0%
C57BL/6NHsd, C57BL/6NJ, C57BL/6NRj, C57BL/6NTac	0	1	1	100.0%

Minimal Strain Sets Explaining All Diagnostic Classes (Number of Markers Explained):

- Solution 1: 129S5/SvEvBrd and C57BL/6J and C57BL/6NRj
  - C57BL/6J: 49 / 157 (31.2%)
  - C57BL/6NRj: 22 / 40 (55.0%)
  - 129S5/SvEvBrd: 1 / 5 (20.0%)
- Solution 2: 129S5/SvEvBrd and C57BL/6JRj and C57BL/6NRj
  - C57BL/6JRj: 49 / 157 (31.2%)
  - C57BL/6NRj: 22 / 40 (55.0%)
  - 129S5/SvEvBrd: 1 / 5 (20.0%)

Chromosome	Start (Mb)	Stop (Mb)	Background	Zygosity
1	3000000	61451021	CBA/J	Homozygous
1	61451021	87433360	C57BL/6J and C57BL/6NTac and CBA/J	Heterozygous
1	87433360	101065154	C57BL/6J and C57BL/6NTac	N/A
1	101065154	168019536	C57BL/6J and C57BL/6NTac and CBA/J	Heterozygous
1	168019536	190259445	C57BL/6J and C57BL/6NTac	N/A
1	190259445	195471971	C57BL/6J and C57BL/6NTac and CBA/J	Heterozygous
2	3000000	45666278	C57BL/6J and C57BL/6NTac and CBA/J	Heterozygous
2	45666278	78267191	C57BL/6J and C57BL/6NTac	N/A
2	78267191	154349372	C57BL/6J and C57BL/6NTac and CBA/J	Heterozygous
2	154349372	166963888	C57BL/6J and C57BL/6NTac	N/A
2	166963888	175780822	C57BL/6J and C57BL/6NTac and CBA/J	Heterozygous

MiniMUGA Background Analysis v2.3.1

**Diplotype Intervals**	2	175780822	182113224	C57BL/6J and C57BL/6NTac	N/A
	3	3000000	37371933	C57BL/6J and C57BL/6NTac and CBA/J	Heterozygous
	3	37371933	41975127	C57BL/6J and C57BL/6NTac	N/A
	3	41975127	56655047	C57BL/6J and C57BL/6NTac and CBA/J	Heterozygous
	3	56655047	60850190	CBA/J	Homozygous
	3	60850190	101716043	C57BL/6J and C57BL/6NTac and CBA/J	Heterozygous
	3	101716043	108381941	C57BL/6J and C57BL/6NTac	N/A
	3	108381941	118919242	C57BL/6J and C57BL/6NTac and CBA/J	Heterozygous
	3	118919242	147169673	C57BL/6J and C57BL/6NTac	N/A
	3	147169673	160039680	C57BL/6J and C57BL/6NTac and CBA/J	Heterozygous
	4	3000000	20258658	C57BL/6J and C57BL/6NTac and CBA/J	Heterozygous
	4	20258658	26280383	C57BL/6J and C57BL/6NTac	N/A
	4	26280383	29346519	C57BL/6J and C57BL/6NTac and CBA/J	Heterozygous
	4	29346519	30650814	Unexplained	Heterozygous
	4	30650814	32327128	C57BL/6J and C57BL/6NTac and CBA/J	Heterozygous
	4	32327128	35563307	C57BL/6J and C57BL/6NTac	N/A
	4	35563307	37995481	Unexplained	Heterozygous
	4	37995481	41348396	Unexplained	Homozygous
	4	41348396	43372387	C57BL/6J and C57BL/6NTac and CBA/J	Heterozygous
	4	43372387	43819249	Unexplained	Heterozygous
	4	43819249	46665692	C57BL/6J and C57BL/6NTac and CBA/J	Heterozygous
	4	46665692	50929602	Unexplained	Heterozygous
	4	50929602	54114833	C57BL/6J and C57BL/6NTac	N/A
	4	54114833	101914190	C57BL/6J and C57BL/6NTac and CBA/J	Heterozygous
	4	101914190	156508116	CBA/J	Homozygous
	5	3000000	130280923	C57BL/6J and C57BL/6NTac	N/A
	5	130280923	151834684	C57BL/6J and C57BL/6NTac and CBA/J	Heterozygous
	6	3000000	149736546	C57BL/6J and C57BL/6NTac	N/A
	7	3000000	16360273	C57BL/6J and C57BL/6NTac and CBA/J	Heterozygous
	7	16360273	65997088	CBA/J	Homozygous
	7	65997088	69096424	C57BL/6J and C57BL/6NTac and CBA/J	Heterozygous
	7	69096424	119823617	C57BL/6J and C57BL/6NTac	N/A

MiniMUGA Background Analysis v2.3.1

7	119823617	126580094	C57BL/6J and C57BL/6NTac and CBA/J	Heterozygous
7	126580094	145441459	C57BL/6J and C57BL/6NTac	N/A
8	3000000	93626178	C57BL/6J and C57BL/6NTac	N/A
8	93626178	119835722	C57BL/6J and C57BL/6NTac and CBA/J	Heterozygous
8	119835722	129401213	C57BL/6J and C57BL/6NTac	N/A
9	3000000	124595110	C57BL/6J and C57BL/6NTac	N/A
10	3000000	14185354	C57BL/6J and C57BL/6NTac	N/A
10	14185354	42858234	C57BL/6J and C57BL/6NTac and CBA/J	Heterozygous
10	42858234	61450853	CBA/J	Homozygous
10	61450853	68332199	C57BL/6J and C57BL/6NTac and CBA/J	Heterozygous
10	68332199	83779430	CBA/J	Homozygous
10	83779430	115781736	C57BL/6J and C57BL/6NTac and CBA/J	Heterozygous
10	115781736	130694993	C57BL/6J and C57BL/6NTac	N/A
11	3000000	22302070	CBA/J	Homozygous
11	22302070	28525615	C57BL/6J and C57BL/6NTac and CBA/J	Heterozygous
11	28525615	105886229	C57BL/6J and C57BL/6NTac	N/A
11	105886229	112771442	C57BL/6J and C57BL/6NTac and CBA/J	Heterozygous
11	112771442	119038285	C57BL/6J and C57BL/6NTac	N/A
11	119038285	122082543	C57BL/6J and C57BL/6NTac and CBA/J	Heterozygous
12	3000000	27585493	C57BL/6J and C57BL/6NTac	N/A
12	27585493	120129022	C57BL/6J and C57BL/6NTac and CBA/J	Heterozygous
13	3000000	51605798	C57BL/6J and C57BL/6NTac	N/A
13	51605798	120421639	C57BL/6J and C57BL/6NTac and CBA/J	Heterozygous
14	3000000	57122837	C57BL/6J and C57BL/6NTac and CBA/J	Heterozygous
14	57122837	76871639	C57BL/6J and C57BL/6NTac	N/A
14	76871639	103377147	C57BL/6J and C57BL/6NTac and CBA/J	Heterozygous
14	103377147	124902244	C57BL/6J and C57BL/6NTac	N/A
15	3000000	92737752	C57BL/6J and C57BL/6NTac and CBA/J	Heterozygous
15	92737752	93634603	CBA/J	Homozygous
15	93634603	104043685	C57BL/6J and C57BL/6NTac and CBA/J	Heterozygous

MiniMUGA Background Analysis v2.3.1

	16	3000000	29701002	C57BL/6J and C57BL/6NTac	N/A
	16	29701002	38637269	C57BL/6J and C57BL/6NTac and CBA/J	Heterozygous
	16	38637269	82429429	CBA/J	Homozygous
	16	82429429	98207768	C57BL/6J and C57BL/6NTac and CBA/J	Heterozygous
	17	3000000	28225412	C57BL/6J and C57BL/6NTac and CBA/J	Heterozygous
	17	28225412	94987271	C57BL/6J and C57BL/6NTac	N/A
	18	3000000	15685654	C57BL/6J and C57BL/6NTac and CBA/J	Heterozygous
	18	15685654	20363699	Unexplained	Heterozygous
	18	20363699	27036500	CBA/J	Homozygous
	18	27036500	90702639	C57BL/6J and C57BL/6NTac and CBA/J	Heterozygous
	19	3000000	20955280	C57BL/6J and C57BL/6NTac	N/A
	19	20955280	47746251	C57BL/6J and C57BL/6NTac and CBA/J	Heterozygous
	19	47746251	49870985	C57BL/6J and C57BL/6NTac	N/A
	19	49870985	61431566	C57BL/6J and C57BL/6NTac and CBA/J	Heterozygous
	X	3000000	171031299	C57BL/6J and C57BL/6NTac	N/A
	MT	0	0	IBD	Hemizygous
