# Supplementary material for: Deletion of 9p drives B-ALL through heterozygous inactivation of Pax5 and Cd72 in preleukemic cells
Source: JCI Insight. 2026 Feb 17;11(7):e199464. doi: 10.1172/jci.insight.199464 (PMC13134721; doi:10.1172/jci.insight.199464)
Supplement: Supplemental data set 1 [file jciinsight-11-199464-s204.zip › Strain_Genotyping/Q098-results-report.pdf]

MiniMUGA Background Analysis v2.3.1

<table>
<tr><th>Sample ID</th><td>Q098</td></tr>
<tr><th>Neogen ID</th><td>AAAU-4529</td></tr>
<tr><th>Summary</th><td>The genotype of this sample is of excellent quality. It is XO and outbred, and likely a mix of C57BL/6J and C57BL/6NTac and CBA/J. Clustering of unexplained markers is evidence of an additional background strain.

Diagnostic SNPs are likely explained by the presence of the background strains
Solution 1: 129S5/SvEvBrd and C57BL/6J and C57BL/6NRj
C57BL/6J: 108 / 165 (65.5%)
C57BL/6NRj: 13 / 39 (33.3%)
129S5/SvEvBrd: 1 / 5 (20.0%)
Solution 2: 129S5/SvEvBrd and C57BL/6JRj and C57BL/6NRj
C57BL/6JRj: 108 / 165 (65.5%)
C57BL/6NRj: 13 / 39 (33.3%)
129S5/SvEvBrd: 1 / 5 (20.0%)
NOTE: There is a discrepancy between the diagnostic backgrounds detected and the primary and secondary background analysis (CBA/J, C57BL/6J, C57BL/6NTac). This is uncommon and should be investigated further.

No genetic constructs were detected in this sample.

WARNING:
There is a discrepancy between the diagnostic backgrounds detected ((129S5/SvEvBrd and C57BL/6J and C57BL/6NRj) or (129S5/SvEvBrd and C57BL/6JRj and C57BL/6NRj)) and the primary background (C57BL/6J and C57BL/6NTac) and secondary background (CBA/J). This is uncommon and should be investigated further.
The presence of a single diagnostic heterozygous call for a single inbred strain should be treated with caution.
This sample likely has more than 2 genetic backgrounds (unexplained regions and/or fractured ideogram). The strain selected for secondary background may be incorrect. The estimation of the contribution of primary and secondary background are likely incorrect. This can potentially be addressed with input from the user.</td></tr>
<tr><th>Genotyping Quality</th><td>Excellent (15 N calls)
All reported results are dependent on genotyping quality.</td></tr>
<tr><th>Chromosomal Sex</th><td>XO</td></tr>
<tr><th>Inbreeding Estimate</th><td>54.6% Inbred
(Percentage of the genome (autosomal and X chromosomes) that is homozygous or hemizygous for primary, secondary, and unknown backgrounds. See Genome Analysis)</td></tr>
<tr><th>Constructs Detected</th><td>
<table>
<tr><th>BlastR</th><th>bpA</th><th>Cas9</th><th>chlor</th><th>cHS4</th><th>Cre</th><th>DTA</th><th>Flp</th><th>g_FP</th><th>hCMV_a</th><th>hCMV_b</th><th>hTK_pr</th><th>iCre</th><th>IRES</th><th>Luc</th><th>r_FP</th><th>rtTA</th><th>SV40</th><th>tTA</th></tr>
<tr><td>-</td><td>-</td><td>-</td><td>-</td><td>-</td><td>-</td><td>-</td><td>-</td><td>-</td><td>-</td><td>-</td><td>-</td><td>-</td><td>-</td><td>-</td><td>-</td><td>-</td><td>-</td><td>-</td></tr>
</table>
</td></tr>
</table>

MiniMUGA Background Analysis v2.3.1

Refined Ideogram

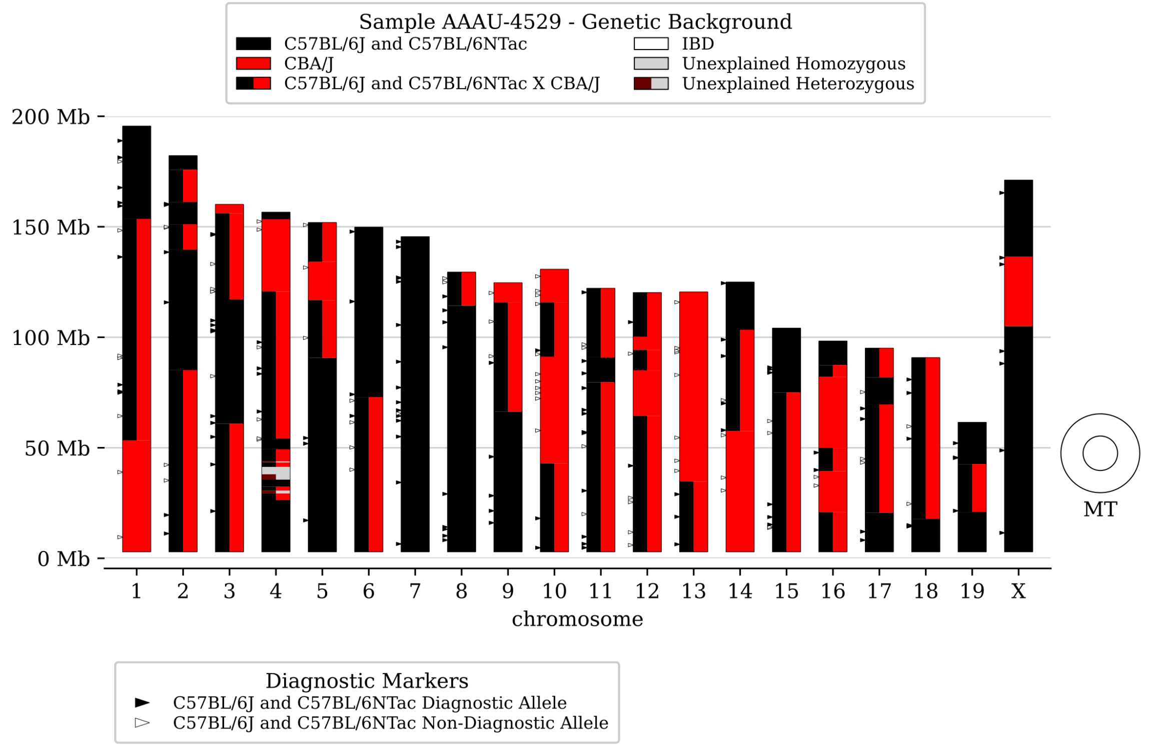

Genome Analysis

Background	Zygosity	Informative Markers	Informative Markers %	Genome %
C57BL/6J and C57BL/6NTac	N/A	1131	42.6%	37.9%
CBA/J	Homozygous	410	15.4%	15.3%
C57BL/6J and C57BL/6NTac X CBA/J	Heterozygous	1101	41.4%	46.5%
Unexplained	Homozygous	5	0.2%	0.1%
Unexplained	Heterozygous	11	0.4%	0.2%
	**Total**	2658	100.0%	100.0%

**Y Chromosome**	Not Applicable
**MT Genome**	MT Haplogroup 6 - 100.0% Consistent<br>Includes C57BL/6J, C57BL/6NTac, CBA/J and 165 other strains

MiniMUGA Background Analysis v2.3.1

Backgrounds Detected (Diagnostic Alleles)

	Diagnostic Alleles Observed			
**Diagnostic Class**	**Homozygous**	**Heterozygous**	**Potential**	**% Observed**
C57BL/6J, C57BL/6JJicTac, C57BL/6JRj	13	61	102	72.5%
C57BL/6J, C57BL/6JRj	4	12	31	51.6%
C57BL/6J, C57BL/6JEiJ, C57BL/6JJicTac, C57BL/6JRj	1	11	21	57.1%
C57BL/6NRj, C57BL/6NTac	0	5	15	33.3%
C57BL/6NJ, C57BL/6NRj, C57BL/6NTac	0	4	10	40.0%
B6N-Tyr<c-Brd>/BrdCrCrl, C57BL/6J, C57BL/6JJicTac, C57BL/6JRj	0	2	5	40.0%
C57BL/6NCrl, C57BL/6NHsd, C57BL/6NJ, C57BL/6NRj, C57BL/6NTac	0	2	2	100.0%
129S5/SvEvBrd	0	1	5	20.0%
B6N-Tyr<c-Brd>/BrdCrCrl, C57BL/6J, C57BL/6JBomTac, C57BL/6JEiJ, C57BL/6JJicTac, C57BL/6JOlaHsd, C57BL/6JRj	0	1	2	50.0%
B6N-Tyr<c-Brd>/BrdCrCrl, C57BL/6J, C57BL/6JEiJ, C57BL/6JJicTac, C57BL/6JRj	0	1	1	100.0%
B6N-Tyr<c-Brd>/BrdCrCrl, C57BL/6NCrl, C57BL/6NHsd, C57BL/6NJ, C57BL/6NRj, C57BL/6NTac	0	1	2	50.0%
C57BL/6J, C57BL/6JBomTac, C57BL/6JEiJ, C57BL/6JJicTac, C57BL/6JOlaHsd, C57BL/6JRj	0	1	2	50.0%
C57BL/6J, C57BL/6JEiJ, C57BL/6JJicTac, C57BL/6JOlaHsd, C57BL/6JRj	0	1	1	100.0%
C57BL/6NRj	0	1	10	10.0%

Minimal Strain Sets Explaining All Diagnostic Classes (Number of Markers Explained):

- Solution 1: 129S5/SvEvBrd and C57BL/6J and C57BL/6NRj
  - C57BL/6J: 108 / 165 (65.5%)
  - C57BL/6NRj: 13 / 39 (33.3%)
  - 129S5/SvEvBrd: 1 / 5 (20.0%)
- Solution 2: 129S5/SvEvBrd and C57BL/6JRj and C57BL/6NRj
  - C57BL/6JRj: 108 / 165 (65.5%)
  - C57BL/6NRj: 13 / 39 (33.3%)
  - 129S5/SvEvBrd: 1 / 5 (20.0%)

Chromosome	Start (Mb)	Stop (Mb)	Background	Zygosity
1	3000000	53295370	CBA/J	Homozygous
1	53295370	153548642	C57BL/6J and C57BL/6NTac and CBA/J	Heterozygous
1	153548642	195471971	C57BL/6J and C57BL/6NTac	N/A
2	3000000	85084816	C57BL/6J and C57BL/6NTac and CBA/J	Heterozygous
2	85084816	139631657	C57BL/6J and C57BL/6NTac	N/A
2	139631657	151062687	C57BL/6J and C57BL/6NTac and CBA/J	Heterozygous
2	151062687	161221795	C57BL/6J and C57BL/6NTac	N/A
2	161221795	175780822	C57BL/6J and C57BL/6NTac and CBA/J	Heterozygous
2	175780822	182113224	C57BL/6J and C57BL/6NTac	N/A

MiniMUGA Background Analysis v2.3.1

Diplotype Intervals	3	3000000	60850190	C57BL/6J and C57BL/6NTac and CBA/J	Heterozygous
	3	60850190	117076578	C57BL/6J and C57BL/6NTac	N/A
	3	117076578	156090101	C57BL/6J and C57BL/6NTac and CBA/J	Heterozygous
	3	156090101	160039680	CBA/J	Homozygous
	4	3000000	26280383	C57BL/6J and C57BL/6NTac	N/A
	4	26280383	29346519	C57BL/6J and C57BL/6NTac and CBA/J	Heterozygous
	4	29346519	30650814	Unexplained	Heterozygous
	4	30650814	32327128	C57BL/6J and C57BL/6NTac and CBA/J	Heterozygous
	4	32327128	35563307	C57BL/6J and C57BL/6NTac	N/A
	4	35563307	37995481	Unexplained	Heterozygous
	4	37995481	41348396	Unexplained	Homozygous
	4	41348396	43372387	C57BL/6J and C57BL/6NTac and CBA/J	Heterozygous
	4	43372387	43819249	Unexplained	Heterozygous
	4	43819249	49280860	C57BL/6J and C57BL/6NTac and CBA/J	Heterozygous
	4	49280860	54114833	C57BL/6J and C57BL/6NTac	N/A
	4	54114833	120738488	C57BL/6J and C57BL/6NTac and CBA/J	Heterozygous
	4	120738488	153356388	CBA/J	Homozygous
	4	153356388	156508116	C57BL/6J and C57BL/6NTac	N/A
	5	3000000	90681879	C57BL/6J and C57BL/6NTac	N/A
	5	90681879	116795433	C57BL/6J and C57BL/6NTac and CBA/J	Heterozygous
	5	116795433	134172373	CBA/J	Homozygous
	5	134172373	151834684	C57BL/6J and C57BL/6NTac and CBA/J	Heterozygous
	6	3000000	72868996	C57BL/6J and C57BL/6NTac and CBA/J	Heterozygous
	6	72868996	149736546	C57BL/6J and C57BL/6NTac	N/A
	7	3000000	145441459	C57BL/6J and C57BL/6NTac	N/A
	8	3000000	114311388	C57BL/6J and C57BL/6NTac	N/A
	8	114311388	129401213	C57BL/6J and C57BL/6NTac and CBA/J	Heterozygous
	9	3000000	66341356	C57BL/6J and C57BL/6NTac	N/A
	9	66341356	115715944	C57BL/6J and C57BL/6NTac and CBA/J	Heterozygous
	9	115715944	124595110	CBA/J	Homozygous
	10	3000000	42917049	C57BL/6J and C57BL/6NTac and CBA/J	Heterozygous
	10	42917049	91235291	CBA/J	Homozygous

10	91235291	115781736	C57BL/6J and C57BL/6NTac and CBA/J	Heterozygous
10	115781736	130694993	CBA/J	Homozygous
11	3000000	79617327	C57BL/6J and C57BL/6NTac and CBA/J	Heterozygous
11	79617327	90803561	C57BL/6J and C57BL/6NTac	N/A
11	90803561	122082543	C57BL/6J and C57BL/6NTac and CBA/J	Heterozygous
12	3000000	64411355	C57BL/6J and C57BL/6NTac and CBA/J	Heterozygous
12	64411355	85015902	CBA/J	Homozygous
12	85015902	94246475	C57BL/6J and C57BL/6NTac and CBA/J	Heterozygous
12	94246475	100284662	CBA/J	Homozygous
12	100284662	120129022	C57BL/6J and C57BL/6NTac and CBA/J	Heterozygous
13	3000000	34755755	C57BL/6J and C57BL/6NTac and CBA/J	Heterozygous
13	34755755	120421639	CBA/J	Homozygous
14	3000000	57544602	CBA/J	Homozygous
14	57544602	103377147	C57BL/6J and C57BL/6NTac and CBA/J	Heterozygous
14	103377147	124902244	C57BL/6J and C57BL/6NTac	N/A
15	3000000	74996398	C57BL/6J and C57BL/6NTac and CBA/J	Heterozygous
15	74996398	104043685	C57BL/6J and C57BL/6NTac	N/A
16	3000000	20813513	C57BL/6J and C57BL/6NTac and CBA/J	Heterozygous
16	20813513	39422954	CBA/J	Homozygous
16	39422954	49897727	C57BL/6J and C57BL/6NTac and CBA/J	Heterozygous
16	49897727	82107593	CBA/J	Homozygous
16	82107593	87403166	C57BL/6J and C57BL/6NTac and CBA/J	Heterozygous
16	87403166	98207768	C57BL/6J and C57BL/6NTac	N/A
17	3000000	20616647	C57BL/6J and C57BL/6NTac	N/A
17	20616647	69590784	C57BL/6J and C57BL/6NTac and CBA/J	Heterozygous
17	69590784	81881415	C57BL/6J and C57BL/6NTac	N/A
17	81881415	94987271	C57BL/6J and C57BL/6NTac and CBA/J	Heterozygous
18	3000000	17841108	C57BL/6J and C57BL/6NTac	N/A
18	17841108	90702639	C57BL/6J and C57BL/6NTac and CBA/J	Heterozygous
19	3000000	20955280	C57BL/6J and C57BL/6NTac	N/A
19	20955280	42582533	C57BL/6J and C57BL/6NTac and CBA/J	Heterozygous

MiniMUGA Background Analysis v2.3.1

	19	42582533	61431566	C57BL/6J and C57BL/6NTac	N/A
	X	3000000	105020820	C57BL/6J and C57BL/6NTac	Hemizygous
	X	105020820	136441962	C57BL/6J and C57BL/6NTac and CBA/J	Hemizygous
	X	136441962	171031299	C57BL/6J and C57BL/6NTac	Hemizygous
	MT	0	0	IBD	Hemizygous
