# Supplementary material for: Deletion of 9p drives B-ALL through heterozygous inactivation of Pax5 and Cd72 in preleukemic cells
Source: JCI Insight. 2026 Feb 17;11(7):e199464. doi: 10.1172/jci.insight.199464 (PMC13134721; doi:10.1172/jci.insight.199464)
Supplement: Supplemental data set 1 [file jciinsight-11-199464-s204.zip › Strain_Genotyping/Q099-results-report.pdf]

MiniMUGA Background Analysis v2.3.1

**Sample ID**	Q099
**Neogen ID**	AAAU-4488
**Summary**	The genotype of this sample is of **excellent** quality. It is **female** and **outbred**, and likely a mix of **C57BL/6J and C57BL/6NTac** and **CBA/J**. Clustering of unexplained markers is evidence of an additional background strain.<br><br>Diagnostic SNPs are likely explained by the presence of the background strains<br>• Solution 1: C57BL/6J and C57BL/6NTac<br>◦ C57BL/6J: 87 / 162 (53.7%)<br>◦ C57BL/6NTac: 17 / 30 (56.7%)<br>• Solution 2: C57BL/6J and C57BL/6NRj<br>◦ C57BL/6J: 87 / 162 (53.7%)<br>◦ C57BL/6NRj: 17 / 30 (56.7%)<br>• Solution 3: C57BL/6JRj and C57BL/6NTac<br>◦ C57BL/6JRj: 87 / 162 (53.7%)<br>◦ C57BL/6NTac: 17 / 30 (56.7%)<br>• Solution 4: C57BL/6JRj and C57BL/6NRj<br>◦ C57BL/6JRj: 87 / 162 (53.7%)<br>◦ C57BL/6NRj: 17 / 30 (56.7%)<br><br>No genetic constructs were detected in this sample.
**Genotyping Quality**	**Excellent (21 N calls)**<br>All reported results are dependent on genotyping quality.
**Chromosomal Sex**	XX
**Inbreeding Estimate**	53.6% Inbred<br>(Percentage of the genome (autosomal and X chromosomes) that is homozygous or hemizygous for primary, secondary, and unknown backgrounds. See Genome Analysis)

Constructs Detected

BlastR	bpA	Cas9	chlor	cHS4	Cre	DTA	Flp	g_FP	hCMV_a	hCMV_b	hTK_pr	iCre	IRES	Luc	r_FP	rtTA	SV40	tTA
-	-	-	-	-	-	-	-	-	-	-	-	-	-	-	-	-	-	-

Refined Ideogram

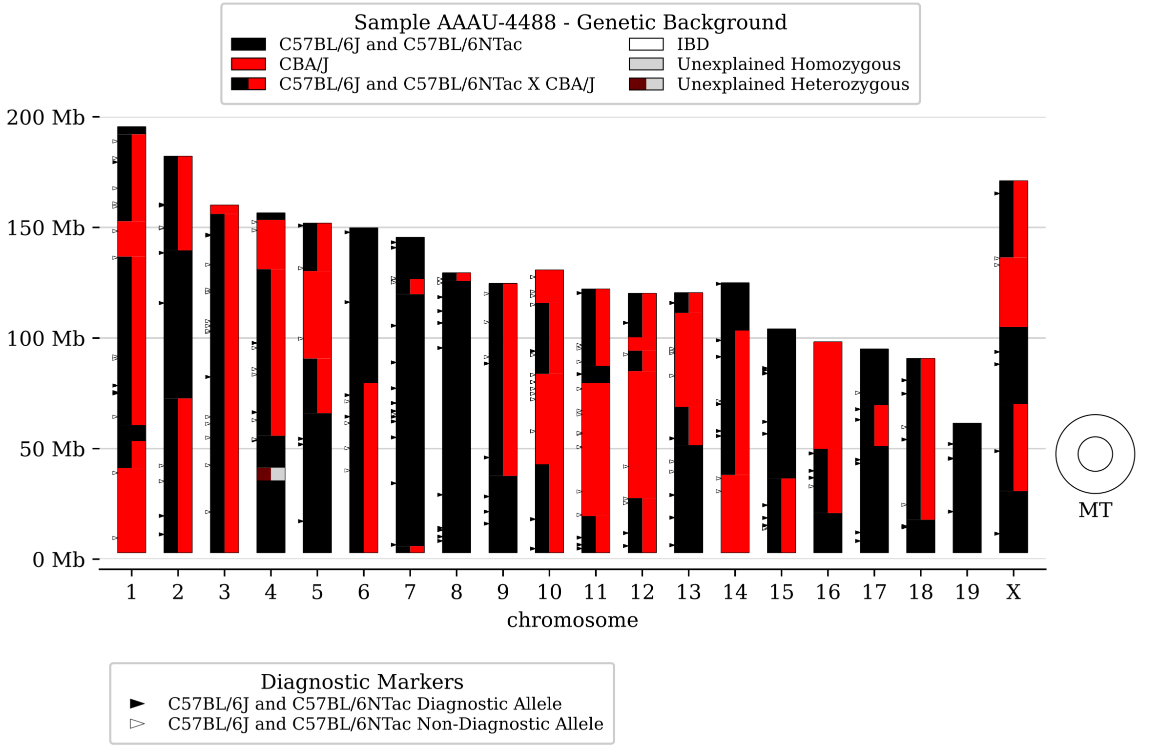

MiniMUGA Background Analysis v2.3.1

Genome Analysis

Background	Zygosity	Informative Markers	Informative Markers %	Genome %
C57BL/6J and C57BL/6NTac	N/A	1084	41.0%	35.9%
CBA/J	Homozygous	490	18.5%	17.7%
C57BL/6J and C57BL/6NTac X CBA/J	Heterozygous	1061	40.1%	46.1%
Unexplained	Heterozygous	10	0.4%	0.2%
	**Total**	2645	100.0%	99.9%

Y Chromosome

Not Applicable

MT Genome

MT Haplogroup 6 - 100.0% Consistent
Includes C57BL/6J, C57BL/6NTac, CBA/J and 165 other strains

Backgrounds Detected (Diagnostic Alleles)

	Diagnostic Alleles Observed			
**Diagnostic Class**	**Homozygous**	**Heterozygous**	**Potential**	**% Observed**
C57BL/6J, C57BL/6JJicTac, C57BL/6JRj	5	53	102	56.9%
C57BL/6J, C57BL/6JRj	6	7	31	41.9%
C57BL/6J, C57BL/6JEiJ, C57BL/6JJicTac, C57BL/6JRj	2	11	21	61.9%
C57BL/6NRj, C57BL/6NTac	0	9	15	60.0%
C57BL/6NJ, C57BL/6NRj, C57BL/6NTac	0	4	10	40.0%
C57BL/6NCrl, C57BL/6NHsd, C57BL/6NJ, C57BL/6NRj, C57BL/6NTac	0	2	2	100.0%
B6N-Tyr<c-Brd>/BrdCrCrl, C57BL/6J, C57BL/6JEiJ, C57BL/6JJicTac, C57BL/6JRj	0	1	1	100.0%
B6N-Tyr<c-Brd>/BrdCrCrl, C57BL/6J, C57BL/6JJicTac, C57BL/6JRj	0	1	5	20.0%
B6N-Tyr<c-Brd>/BrdCrCrl, C57BL/6NCrl, C57BL/6NHsd, C57BL/6NJ, C57BL/6NRj, C57BL/6NTac	0	1	2	50.0%
C57BL/6J, C57BL/6JBomTac, C57BL/6JEiJ, C57BL/6JJicTac, C57BL/6JOlaHsd, C57BL/6JRj	0	1	2	50.0%
C57BL/6NHsd, C57BL/6NJ, C57BL/6NRj, C57BL/6NTac	0	1	1	100.0%

Minimal Strain Sets Explaining All Diagnostic Classes (Number of Markers Explained):

- Solution 1: C57BL/6J and C57BL/6NTac
  - C57BL/6J: 87 / 162 (53.7%)
  - C57BL/6NTac: 17 / 30 (56.7%)
- Solution 2: C57BL/6J and C57BL/6NRj
  - C57BL/6J: 87 / 162 (53.7%)
  - C57BL/6NRj: 17 / 30 (56.7%)
- Solution 3: C57BL/6JRj and C57BL/6NTac
  - C57BL/6JRj: 87 / 162 (53.7%)
  - C57BL/6NTac: 17 / 30 (56.7%)
- Solution 4: C57BL/6JRj and C57BL/6NRj
  - C57BL/6JRj: 87 / 162 (53.7%)
  - C57BL/6NRj: 17 / 30 (56.7%)

Chromosome	Start (Mb)	Stop (Mb)	Background	Zygosity
1	3000000	41199760	CBA/J	Homozygous
1	41199760	53457225	C57BL/6J and C57BL/6NTac and CBA/J	Heterozygous

MiniMUGA Background Analysis v2.3.1

**Diplotype Intervals**	1	53457225	60621237	C57BL/6J and C57BL/6NTac	N/A
	1	60621237	136798402	C57BL/6J and C57BL/6NTac and CBA/J	Heterozygous
	1	136798402	152801739	CBA/J	Homozygous
	1	152801739	192078159	C57BL/6J and C57BL/6NTac and CBA/J	Heterozygous
	1	192078159	195471971	C57BL/6J and C57BL/6NTac	N/A
	2	3000000	72629186	C57BL/6J and C57BL/6NTac and CBA/J	Heterozygous
	2	72629186	139631657	C57BL/6J and C57BL/6NTac	N/A
	2	139631657	182113224	C57BL/6J and C57BL/6NTac and CBA/J	Heterozygous
	3	3000000	156090101	C57BL/6J and C57BL/6NTac and CBA/J	Heterozygous
	3	156090101	160039680	CBA/J	Homozygous
	4	3000000	35563307	C57BL/6J and C57BL/6NTac	N/A
	4	35563307	41348396	Unexplained	Heterozygous
	4	41348396	55826643	C57BL/6J and C57BL/6NTac	N/A
	4	55826643	131104093	C57BL/6J and C57BL/6NTac and CBA/J	Heterozygous
	4	131104093	153356388	CBA/J	Homozygous
	4	153356388	156508116	C57BL/6J and C57BL/6NTac	N/A
	5	3000000	66015308	C57BL/6J and C57BL/6NTac	N/A
	5	66015308	90681879	C57BL/6J and C57BL/6NTac and CBA/J	Heterozygous
	5	90681879	130280923	CBA/J	Homozygous
	5	130280923	151834684	C57BL/6J and C57BL/6NTac and CBA/J	Heterozygous
	6	3000000	79701235	C57BL/6J and C57BL/6NTac and CBA/J	Heterozygous
	6	79701235	149736546	C57BL/6J and C57BL/6NTac	N/A
	7	3000000	5883380	C57BL/6J and C57BL/6NTac and CBA/J	Heterozygous
	7	5883380	119823617	C57BL/6J and C57BL/6NTac	N/A
	7	119823617	126580094	C57BL/6J and C57BL/6NTac and CBA/J	Heterozygous
	7	126580094	145441459	C57BL/6J and C57BL/6NTac	N/A
	8	3000000	125832225	C57BL/6J and C57BL/6NTac	N/A
	8	125832225	129401213	C57BL/6J and C57BL/6NTac and CBA/J	Heterozygous
	9	3000000	37691490	C57BL/6J and C57BL/6NTac	N/A
	9	37691490	124595110	C57BL/6J and C57BL/6NTac and CBA/J	Heterozygous
	10	3000000	42858234	C57BL/6J and C57BL/6NTac and CBA/J	Heterozygous

MiniMUGA Background Analysis v2.3.1

10	42858234	83779430	CBA/J	Homozygous
10	83779430	115781736	C57BL/6J and C57BL/6NTac and CBA/J	Heterozygous
10	115781736	130694993	CBA/J	Homozygous
11	3000000	19463075	C57BL/6J and C57BL/6NTac and CBA/J	Heterozygous
11	19463075	79617327	CBA/J	Homozygous
11	79617327	87432699	C57BL/6J and C57BL/6NTac	N/A
11	87432699	122082543	C57BL/6J and C57BL/6NTac and CBA/J	Heterozygous
12	3000000	27585493	C57BL/6J and C57BL/6NTac and CBA/J	Heterozygous
12	27585493	85015902	CBA/J	Homozygous
12	85015902	94246475	C57BL/6J and C57BL/6NTac and CBA/J	Heterozygous
12	94246475	100284662	CBA/J	Homozygous
12	100284662	120129022	C57BL/6J and C57BL/6NTac and CBA/J	Heterozygous
13	3000000	51605798	C57BL/6J and C57BL/6NTac	N/A
13	51605798	68886272	C57BL/6J and C57BL/6NTac and CBA/J	Heterozygous
13	68886272	111361745	CBA/J	Homozygous
13	111361745	120421639	C57BL/6J and C57BL/6NTac and CBA/J	Heterozygous
14	3000000	38092288	CBA/J	Homozygous
14	38092288	103377147	C57BL/6J and C57BL/6NTac and CBA/J	Heterozygous
14	103377147	124902244	C57BL/6J and C57BL/6NTac	N/A
15	3000000	36473640	C57BL/6J and C57BL/6NTac and CBA/J	Heterozygous
15	36473640	104043685	C57BL/6J and C57BL/6NTac	N/A
16	3000000	20813513	C57BL/6J and C57BL/6NTac	N/A
16	20813513	49897727	C57BL/6J and C57BL/6NTac and CBA/J	Heterozygous
16	49897727	98207768	CBA/J	Homozygous
17	3000000	51237708	C57BL/6J and C57BL/6NTac	N/A
17	51237708	69590784	C57BL/6J and C57BL/6NTac and CBA/J	Heterozygous
17	69590784	94987271	C57BL/6J and C57BL/6NTac	N/A
18	3000000	17841108	C57BL/6J and C57BL/6NTac	N/A
18	17841108	90702639	C57BL/6J and C57BL/6NTac and CBA/J	Heterozygous
19	3000000	61431566	C57BL/6J and C57BL/6NTac	N/A
X	3000000	30815921	C57BL/6J and C57BL/6NTac	N/A
X	30815921	70193631	C57BL/6J and C57BL/6NTac and CBA/J	Heterozygous

MiniMUGA Background Analysis v2.3.1

	X	70193631	105020820	C57BL/6J and C57BL/6NTac	N/A
	X	105020820	136441962	CBA/J	Homozygous
	X	136441962	171031299	C57BL/6J and C57BL/6NTac and CBA/J	Heterozygous
	MT	0	0	IBD	Hemizygous
