# Supplementary material for: Deletion of 9p drives B-ALL through heterozygous inactivation of Pax5 and Cd72 in preleukemic cells
Source: JCI Insight. 2026 Feb 17;11(7):e199464. doi: 10.1172/jci.insight.199464 (PMC13134721; doi:10.1172/jci.insight.199464)
Supplement: Supplemental data set 1 [file jciinsight-11-199464-s204.zip › Strain_Genotyping/Q113-results-report.pdf]

MiniMUGA Background Analysis v2.3.1

<table>
<tr><th>Sample ID</th><td>Q113</td></tr>
<tr><th>Neogen ID</th><td>AAAU-4489</td></tr>
<tr><th>Summary</th><td>The genotype of this sample is of excellent quality. It is female and outbred, and likely a mix of C57BL/6J and C57BL/6NTac and CBA/J. Clustering of unexplained markers is evidence of an additional background strain.

Diagnostic SNPs are likely explained by the presence of the background strains
Solution 1: C57BL/6J and C57BL/6NRj
C57BL/6J: 68 / 162 (42.0%)
C57BL/6NRj: 15 / 39 (38.5%)
Solution 2: C57BL/6JRj and C57BL/6NRj
C57BL/6JRj: 68 / 162 (42.0%)
C57BL/6NRj: 15 / 39 (38.5%)
NOTE: There is a discrepancy between the diagnostic backgrounds detected and the primary and secondary background analysis (CBA/J, C57BL/6J, C57BL/6NTac). This is uncommon and should be investigated further.

No genetic constructs were detected in this sample.

WARNING:
There is a discrepancy between the diagnostic backgrounds detected ((C57BL/6J and C57BL/6NRj) or (C57BL/6JRj and C57BL/6NRj)) and the primary background (C57BL/6J and C57BL/6NTac) and secondary background (CBA/J). This is uncommon and should be investigated further.</td></tr>
<tr><th>Genotyping Quality</th><td>Excellent (10 N calls)
All reported results are dependent on genotyping quality.</td></tr>
<tr><th>Chromosomal Sex</th><td>XX</td></tr>
<tr><th>Inbreeding Estimate</th><td>53.6% Inbred
(Percentage of the genome (autosomal and X chromosomes) that is homozygous or hemizygous for primary, secondary, and unknown backgrounds. See Genome Analysis)</td></tr>
<tr><th>Constructs Detected</th><td>

BlastR	bpA	Cas9	chlor	cHS4	Cre	DTA	Flp	g_FP	hCMV_a	hCMV_b	hTK_pr	iCre	IRES	Luc	r_FP	rtTA	SV40	tTA
-	-	-	-	-	-	-	-	-	-	-	-	-	-	-	-	-	-	-

</td></tr>
</table>

MiniMUGA Background Analysis v2.3.1

Refined Ideogram

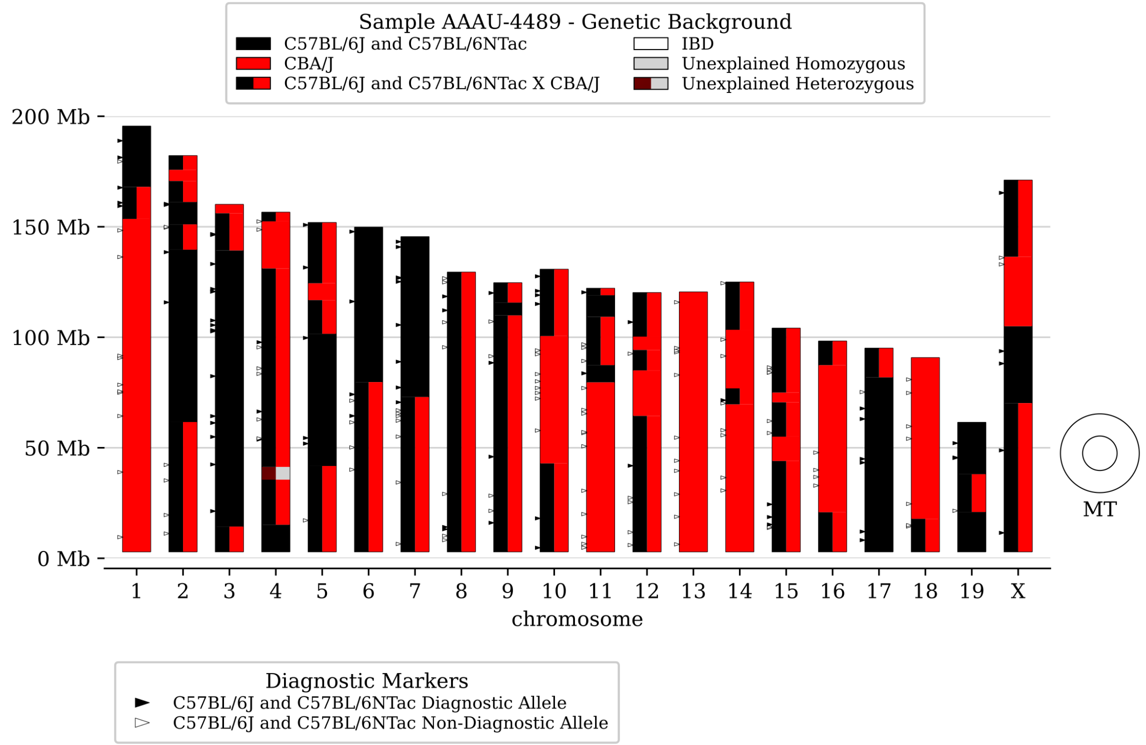

Genome Analysis

Background	Zygosity	Informative Markers	Informative Markers %	Genome %
C57BL/6J and C57BL/6NTac	N/A	851	32.2%	24.6%
CBA/J	Homozygous	715	27.0%	29.0%
C57BL/6J and C57BL/6NTac X CBA/J	Heterozygous	1070	40.4%	46.2%
Unexplained	Heterozygous	10	0.4%	0.2%
	**Total**	2646	100.0%	100.0%

Y Chromosome

Not Applicable

MT Genome

MT Haplogroup 6 - 100.0% Consistent
Includes C57BL/6J, C57BL/6NTac, CBA/J and 165 other strains

MiniMUGA Background Analysis v2.3.1

Backgrounds Detected (Diagnostic Alleles)

Diagnostic Class	Diagnostic Alleles Observed: Homozygous	Diagnostic Alleles Observed: Heterozygous	Potential	% Observed
C57BL/6J, C57BL/6JJicTac, C57BL/6JRj	8	38	102	45.1%
C57BL/6J, C57BL/6JRj	3	7	31	32.3%
C57BL/6J, C57BL/6JEiJ, C57BL/6JJicTac, C57BL/6JRj	1	7	21	38.1%
C57BL/6NRj, C57BL/6NTac	0	6	15	40.0%
C57BL/6NJ, C57BL/6NRj, C57BL/6NTac	0	5	10	50.0%
B6N-Tyr<c-Brd>/BrdCrCrl, C57BL/6J, C57BL/6JBomTac, C57BL/6JEiJ, C57BL/6JJicTac, C57BL/6JOlaHsd, C57BL/6JRj	0	2	2	100.0%
C57BL/6NCrl, C57BL/6NHsd, C57BL/6NJ, C57BL/6NRj, C57BL/6NTac	0	2	2	100.0%
B6N-Tyr<c-Brd>/BrdCrCrl, C57BL/6J, C57BL/6JEiJ, C57BL/6JJicTac, C57BL/6JRj	0	1	1	100.0%
B6N-Tyr<c-Brd>/BrdCrCrl, C57BL/6J, C57BL/6JJicTac, C57BL/6JRj	0	1	5	20.0%
B6N-Tyr<c-Brd>/BrdCrCrl, C57BL/6NCrl, C57BL/6NHsd, C57BL/6NJ, C57BL/6NRj, C57BL/6NTac	0	1	2	50.0%
C57BL/6NRj	0	1	10	10.0%

Minimal Strain Sets Explaining All Diagnostic Classes (Number of Markers Explained):

- Solution 1: C57BL/6J and C57BL/6NRj
  - C57BL/6J: 68 / 162 (42.0%)
  - C57BL/6NRj: 15 / 39 (38.5%)
- Solution 2: C57BL/6JRj and C57BL/6NRj
  - C57BL/6JRj: 68 / 162 (42.0%)
  - C57BL/6NRj: 15 / 39 (38.5%)

Chromosome	Start (Mb)	Stop (Mb)	Background	Zygosity
1	3000000	153548642	CBA/J	Homozygous
1	153548642	168019536	C57BL/6J and C57BL/6NTac and CBA/J	Heterozygous
1	168019536	195471971	C57BL/6J and C57BL/6NTac	N/A
2	3000000	61636788	C57BL/6J and C57BL/6NTac and CBA/J	Heterozygous
2	61636788	139631657	C57BL/6J and C57BL/6NTac	N/A
2	139631657	151062687	C57BL/6J and C57BL/6NTac and CBA/J	Heterozygous
2	151062687	161221795	C57BL/6J and C57BL/6NTac	N/A
2	161221795	170694096	C57BL/6J and C57BL/6NTac and CBA/J	Heterozygous
2	170694096	175780822	CBA/J	Homozygous
2	175780822	182113224	C57BL/6J and C57BL/6NTac and CBA/J	Heterozygous
3	3000000	14328941	C57BL/6J and C57BL/6NTac and CBA/J	Heterozygous
3	14328941	139297311	C57BL/6J and C57BL/6NTac	N/A
3	139297311	156090101	C57BL/6J and C57BL/6NTac and CBA/J	Heterozygous

	3	156090101	160039680	CBA/J	Homozygous
**Diplotype Intervals**	4	3000000	15188739	C57BL/6J and C57BL/6NTac	N/A
	4	15188739	35563307	C57BL/6J and C57BL/6NTac and CBA/J	Heterozygous
	4	35563307	41348396	Unexplained	Heterozygous
	4	41348396	131104093	C57BL/6J and C57BL/6NTac and CBA/J	Heterozygous
	4	131104093	152440879	CBA/J	Homozygous
	4	152440879	156508116	C57BL/6J and C57BL/6NTac and CBA/J	Heterozygous
	5	3000000	41755530	C57BL/6J and C57BL/6NTac and CBA/J	Heterozygous
	5	41755530	101581477	C57BL/6J and C57BL/6NTac	N/A
	5	101581477	116795433	C57BL/6J and C57BL/6NTac and CBA/J	Heterozygous
	5	116795433	124446826	CBA/J	Homozygous
	5	124446826	151834684	C57BL/6J and C57BL/6NTac and CBA/J	Heterozygous
	6	3000000	79701235	C57BL/6J and C57BL/6NTac and CBA/J	Heterozygous
	6	79701235	149736546	C57BL/6J and C57BL/6NTac	N/A
	7	3000000	72944748	C57BL/6J and C57BL/6NTac and CBA/J	Heterozygous
	7	72944748	145441459	C57BL/6J and C57BL/6NTac	N/A
	8	3000000	129401213	C57BL/6J and C57BL/6NTac and CBA/J	Heterozygous
	9	3000000	109855467	C57BL/6J and C57BL/6NTac and CBA/J	Heterozygous
	9	109855467	115715944	C57BL/6J and C57BL/6NTac	N/A
	9	115715944	124595110	C57BL/6J and C57BL/6NTac and CBA/J	Heterozygous
	10	3000000	42917049	C57BL/6J and C57BL/6NTac and CBA/J	Heterozygous
	10	42917049	100561092	CBA/J	Homozygous
	10	100561092	130694993	C57BL/6J and C57BL/6NTac and CBA/J	Heterozygous
	11	3000000	79617327	CBA/J	Homozygous
	11	79617327	87432699	C57BL/6J and C57BL/6NTac	N/A
	11	87432699	109213602	C57BL/6J and C57BL/6NTac and CBA/J	Heterozygous
	11	109213602	119038285	C57BL/6J and C57BL/6NTac	N/A
	11	119038285	122082543	C57BL/6J and C57BL/6NTac and CBA/J	Heterozygous
	12	3000000	64411355	C57BL/6J and C57BL/6NTac and CBA/J	Heterozygous
	12	64411355	85015902	CBA/J	Homozygous
	12	85015902	94246475	C57BL/6J and C57BL/6NTac and CBA/J	Heterozygous
	12	94246475	100284662	CBA/J	Homozygous

MiniMUGA Background Analysis v2.3.1

	12	100284662	120129022	C57BL/6J and C57BL/6NTac and CBA/J	Heterozygous
	13	3000000	120421639	CBA/J	Homozygous
	14	3000000	69660428	CBA/J	Homozygous
	14	69660428	76871639	C57BL/6J and C57BL/6NTac and CBA/J	Heterozygous
	14	76871639	103377147	CBA/J	Homozygous
	14	103377147	124902244	C57BL/6J and C57BL/6NTac and CBA/J	Heterozygous
	15	3000000	44010563	C57BL/6J and C57BL/6NTac and CBA/J	Heterozygous
	15	44010563	55016741	CBA/J	Homozygous
	15	55016741	70554147	C57BL/6J and C57BL/6NTac and CBA/J	Heterozygous
	15	70554147	74996398	CBA/J	Homozygous
	15	74996398	104043685	C57BL/6J and C57BL/6NTac and CBA/J	Heterozygous
	16	3000000	20813513	C57BL/6J and C57BL/6NTac and CBA/J	Heterozygous
	16	20813513	87403166	CBA/J	Homozygous
	16	87403166	98207768	C57BL/6J and C57BL/6NTac and CBA/J	Heterozygous
	17	3000000	81881415	C57BL/6J and C57BL/6NTac	N/A
	17	81881415	94987271	C57BL/6J and C57BL/6NTac and CBA/J	Heterozygous
	18	3000000	17841108	C57BL/6J and C57BL/6NTac and CBA/J	Heterozygous
	18	17841108	90702639	CBA/J	Homozygous
	19	3000000	20955280	C57BL/6J and C57BL/6NTac	N/A
	19	20955280	38025239	C57BL/6J and C57BL/6NTac and CBA/J	Heterozygous
	19	38025239	61431566	C57BL/6J and C57BL/6NTac	N/A
	X	3000000	70193631	C57BL/6J and C57BL/6NTac and CBA/J	Heterozygous
	X	70193631	105020820	C57BL/6J and C57BL/6NTac	N/A
	X	105020820	136441962	CBA/J	Homozygous
	X	136441962	171031299	C57BL/6J and C57BL/6NTac and CBA/J	Heterozygous
	MT	0	0	IBD	Hemizygous
