# Supplementary material for: Deletion of 9p drives B-ALL through heterozygous inactivation of Pax5 and Cd72 in preleukemic cells
Source: JCI Insight. 2026 Feb 17;11(7):e199464. doi: 10.1172/jci.insight.199464 (PMC13134721; doi:10.1172/jci.insight.199464)
Supplement: Supplemental data set 1 [file jciinsight-11-199464-s204.zip › Strain_Genotyping/Q301-results-report.pdf]

MiniMUGA Background Analysis v2.3.1

**Sample ID**	Q301
**Neogen ID**	AAAU-4521
**Summary**	The genotype of this sample is of **excellent** quality. It is **female** and **outbred**, and likely a mix of **C57BL/6J and C57BL/6NTac** and **CBA/J**. Clustering of unexplained markers is evidence of an additional background strain.<br><br>Diagnostic SNPs are likely explained by the presence of the background strains<br>• Solution 1: 129S5/SvEvBrd and C57BL/6J and C57BL/6NRj<br>◦ C57BL/6J: 56 / 160 (35.0%)<br>◦ C57BL/6NRj: 18 / 40 (45.0%)<br>◦ 129S5/SvEvBrd: 1 / 5 (20.0%)<br>• Solution 2: 129S5/SvEvBrd and C57BL/6JRj and C57BL/6NRj<br>◦ C57BL/6JRj: 56 / 160 (35.0%)<br>◦ C57BL/6NRj: 18 / 40 (45.0%)<br>◦ 129S5/SvEvBrd: 1 / 5 (20.0%)<br><br>NOTE: There is a discrepancy between the diagnostic backgrounds detected and the primary and secondary background analysis (CBA/J, C57BL/6J, C57BL/6NTac). This is uncommon and should be investigated further.<br><br>No genetic constructs were detected in this sample.<br><br>WARNING:<br>• There is a discrepancy between the diagnostic backgrounds detected ((129S5/SvEvBrd and C57BL/6J and C57BL/6NRj) or (129S5/SvEvBrd and C57BL/6JRj and C57BL/6NRj)) and the primary background (C57BL/6J and C57BL/6NTac) and secondary background (CBA/J). This is uncommon and should be investigated further.<br>• The presence of a single diagnostic heterozygous call for a single inbred strain should be treated with caution.<br>• This sample likely has more than 2 genetic backgrounds (unexplained regions and/or fractured ideogram). The strain selected for secondary background may be incorrect. The estimation of the contribution of primary and secondary background are likely incorrect. This can potentially be addressed with input from the user.
**Genotyping Quality**	**Excellent (23 N calls)**<br>All reported results are dependent on genotyping quality.
**Chromosomal Sex**	XX
**Inbreeding Estimate**	44.3% Inbred<br>(Percentage of the genome (autosomal and X chromosomes) that is homozygous or hemizygous for primary, secondary, and unknown backgrounds. See Genome Analysis)

Constructs Detected

BlastR	bpA	Cas9	chlor	cHS4	Cre	DTA	Flp	g_FP	hCMV_a	hCMV_b	hTK_pr	iCre	IRES	Luc	r_FP	rtTA	SV40	tTA
-	-	-	-	-	-	-	-	-	-	-	-	-	-	-	-	-	-	-

MiniMUGA Background Analysis v2.3.1

Refined Ideogram

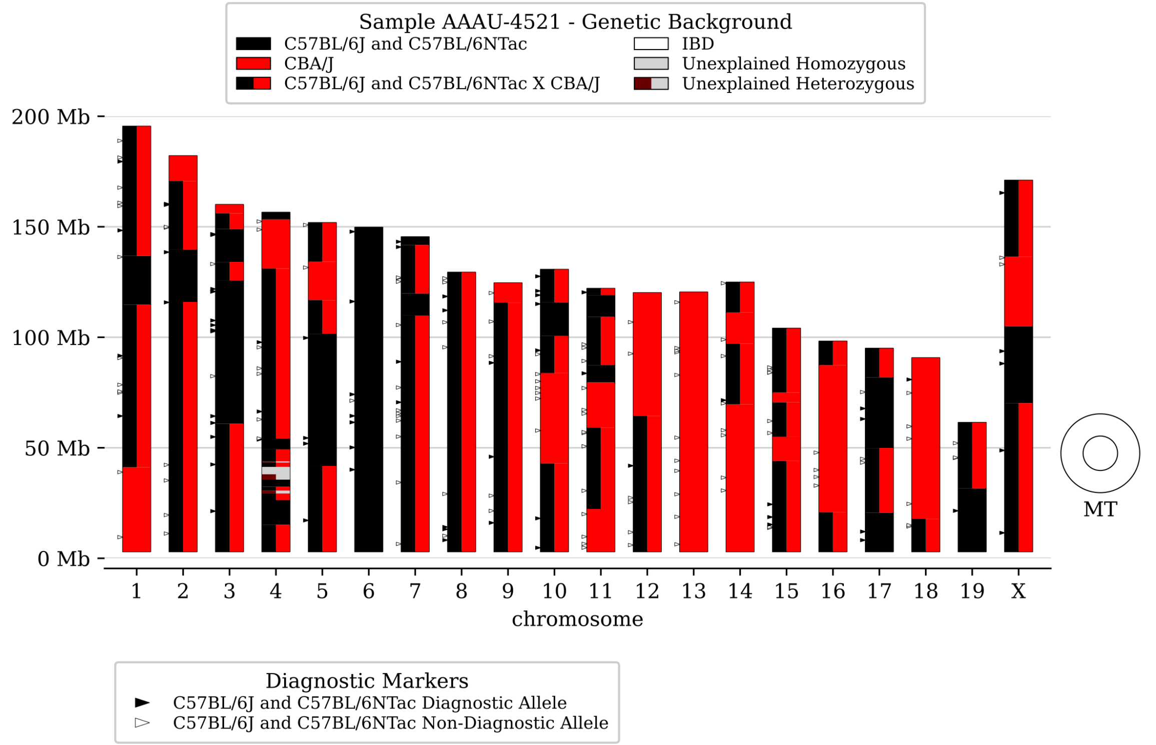

Genome Analysis

Background	Zygosity	Informative Markers	Informative Markers %	Genome %
C57BL/6J and C57BL/6NTac	N/A	592	22.4%	20.0%
CBA/J	Homozygous	609	23.0%	24.2%
C57BL/6J and C57BL/6NTac X CBA/J	Heterozygous	1428	54.0%	55.5%
Unexplained	Homozygous	5	0.2%	0.1%
Unexplained	Heterozygous	10	0.4%	0.2%
	**Total**	2644	100.0%	100.0%

**Y Chromosome**	Not Applicable
**MT Genome**	MT Haplogroup 6 - 100.0% Consistent<br>Includes C57BL/6J, C57BL/6NTac, CBA/J and 165 other strains

MiniMUGA Background Analysis v2.3.1

Backgrounds Detected (Diagnostic Alleles)

Diagnostic Class	Diagnostic Alleles Observed: Homozygous	Heterozygous	Potential	% Observed
C57BL/6J, C57BL/6JJicTac, C57BL/6JRj	11	26	102	36.3%
C57BL/6J, C57BL/6JRj	1	8	31	29.0%
C57BL/6J, C57BL/6JEiJ, C57BL/6JJicTac, C57BL/6JRj	0	8	21	38.1%
C57BL/6NJ, C57BL/6NRj, C57BL/6NTac	0	7	10	70.0%
C57BL/6NRj, C57BL/6NTac	0	6	15	40.0%
C57BL/6NCrl, C57BL/6NHsd, C57BL/6NJ, C57BL/6NRj, C57BL/6NTac	0	2	2	100.0%
129S5/SvEvBrd	0	1	5	20.0%
B6N-Tyr<c-Brd>/BrdCrCrl, C57BL/6J, C57BL/6JEiJ, C57BL/6JJicTac, C57BL/6JRj	0	1	1	100.0%
B6N-Tyr<c-Brd>/BrdCrCrl, C57BL/6J, C57BL/6JJicTac, C57BL/6JRj	0	1	5	20.0%
B6N-Tyr<c-Brd>/BrdCrCrl, C57BL/6NCrl, C57BL/6NHsd, C57BL/6NJ, C57BL/6NRj, C57BL/6NTac	0	1	2	50.0%
C57BL/6NHsd, C57BL/6NJ, C57BL/6NRj, C57BL/6NTac	0	1	1	100.0%
C57BL/6NRj	0	1	10	10.0%

Minimal Strain Sets Explaining All Diagnostic Classes (Number of Markers Explained):

- Solution 1: 129S5/SvEvBrd and C57BL/6J and C57BL/6NRj
  - C57BL/6J: 56 / 160 (35.0%)
  - C57BL/6NRj: 18 / 40 (45.0%)
  - 129S5/SvEvBrd: 1 / 5 (20.0%)
- Solution 2: 129S5/SvEvBrd and C57BL/6JRj and C57BL/6NRj
  - C57BL/6JRj: 56 / 160 (35.0%)
  - C57BL/6NRj: 18 / 40 (45.0%)
  - 129S5/SvEvBrd: 1 / 5 (20.0%)

Chromosome	Start (Mb)	Stop (Mb)	Background	Zygosity
1	3000000	41199760	CBA/J	Homozygous
1	41199760	114727849	C57BL/6J and C57BL/6NTac and CBA/J	Heterozygous
1	114727849	136798402	C57BL/6J and C57BL/6NTac	N/A
1	136798402	195471971	C57BL/6J and C57BL/6NTac and CBA/J	Heterozygous
2	3000000	115970567	C57BL/6J and C57BL/6NTac and CBA/J	Heterozygous
2	115970567	139631657	C57BL/6J and C57BL/6NTac	N/A
2	139631657	170694096	C57BL/6J and C57BL/6NTac and CBA/J	Heterozygous
2	170694096	182113224	CBA/J	Homozygous
3	3000000	60850190	C57BL/6J and C57BL/6NTac and CBA/J	Heterozygous
3	60850190	125708355	C57BL/6J and C57BL/6NTac	N/A
3	125708355	134049530	C57BL/6J and C57BL/6NTac and CBA/J	Heterozygous

MiniMUGA Background Analysis v2.3.1

Diplotype Intervals	3	134049530	148967944	C57BL/6J and C57BL/6NTac	N/A
	3	148967944	156090101	C57BL/6J and C57BL/6NTac and CBA/J	Heterozygous
	3	156090101	160039680	CBA/J	Homozygous
	4	3000000	15188739	C57BL/6J and C57BL/6NTac and CBA/J	Heterozygous
	4	15188739	26280383	C57BL/6J and C57BL/6NTac	N/A
	4	26280383	29346519	C57BL/6J and C57BL/6NTac and CBA/J	Heterozygous
	4	29346519	30650814	Unexplained	Heterozygous
	4	30650814	32327128	C57BL/6J and C57BL/6NTac and CBA/J	Heterozygous
	4	32327128	35563307	C57BL/6J and C57BL/6NTac	N/A
	4	35563307	37995481	Unexplained	Heterozygous
	4	37995481	41348396	Unexplained	Homozygous
	4	41348396	43372387	C57BL/6J and C57BL/6NTac and CBA/J	Heterozygous
	4	43372387	43819249	Unexplained	Heterozygous
	4	43819249	49280860	C57BL/6J and C57BL/6NTac and CBA/J	Heterozygous
	4	49280860	54114833	C57BL/6J and C57BL/6NTac	N/A
	4	54114833	131104093	C57BL/6J and C57BL/6NTac and CBA/J	Heterozygous
	4	131104093	153356388	CBA/J	Homozygous
	4	153356388	156508116	C57BL/6J and C57BL/6NTac	N/A
	5	3000000	41755530	C57BL/6J and C57BL/6NTac and CBA/J	Heterozygous
	5	41755530	101581477	C57BL/6J and C57BL/6NTac	N/A
	5	101581477	116795433	C57BL/6J and C57BL/6NTac and CBA/J	Heterozygous
	5	116795433	134172373	CBA/J	Homozygous
	5	134172373	151834684	C57BL/6J and C57BL/6NTac and CBA/J	Heterozygous
	6	3000000	149736546	C57BL/6J and C57BL/6NTac	N/A
	7	3000000	109805667	C57BL/6J and C57BL/6NTac and CBA/J	Heterozygous
	7	109805667	119823617	C57BL/6J and C57BL/6NTac	N/A
	7	119823617	141750158	C57BL/6J and C57BL/6NTac and CBA/J	Heterozygous
	7	141750158	145441459	C57BL/6J and C57BL/6NTac	N/A
	8	3000000	129401213	C57BL/6J and C57BL/6NTac and CBA/J	Heterozygous
	9	3000000	115715944	C57BL/6J and C57BL/6NTac and CBA/J	Heterozygous
	9	115715944	124595110	CBA/J	Homozygous
	10	3000000	42917049	C57BL/6J and C57BL/6NTac and CBA/J	Heterozygous

MiniMUGA Background Analysis v2.3.1

10	42917049	83779430	CBA/J	Homozygous
10	83779430	100561092	C57BL/6J and C57BL/6NTac and CBA/J	Heterozygous
10	100561092	115781736	C57BL/6J and C57BL/6NTac	N/A
10	115781736	130694993	C57BL/6J and C57BL/6NTac and CBA/J	Heterozygous
11	3000000	22302070	CBA/J	Homozygous
11	22302070	59127711	C57BL/6J and C57BL/6NTac and CBA/J	Heterozygous
11	59127711	79617327	CBA/J	Homozygous
11	79617327	87432699	C57BL/6J and C57BL/6NTac	N/A
11	87432699	109213602	C57BL/6J and C57BL/6NTac and CBA/J	Heterozygous
11	109213602	119038285	C57BL/6J and C57BL/6NTac	N/A
11	119038285	122082543	C57BL/6J and C57BL/6NTac and CBA/J	Heterozygous
12	3000000	64411355	C57BL/6J and C57BL/6NTac and CBA/J	Heterozygous
12	64411355	120129022	CBA/J	Homozygous
13	3000000	120421639	CBA/J	Homozygous
14	3000000	69660428	CBA/J	Homozygous
14	69660428	97106405	C57BL/6J and C57BL/6NTac and CBA/J	Heterozygous
14	97106405	111185375	CBA/J	Homozygous
14	111185375	124902244	C57BL/6J and C57BL/6NTac and CBA/J	Heterozygous
15	3000000	44010563	C57BL/6J and C57BL/6NTac and CBA/J	Heterozygous
15	44010563	55016741	CBA/J	Homozygous
15	55016741	70554147	C57BL/6J and C57BL/6NTac and CBA/J	Heterozygous
15	70554147	74996398	CBA/J	Homozygous
15	74996398	104043685	C57BL/6J and C57BL/6NTac and CBA/J	Heterozygous
16	3000000	20813513	C57BL/6J and C57BL/6NTac and CBA/J	Heterozygous
16	20813513	87403166	CBA/J	Homozygous
16	87403166	98207768	C57BL/6J and C57BL/6NTac and CBA/J	Heterozygous
17	3000000	20616647	C57BL/6J and C57BL/6NTac	N/A
17	20616647	49885651	C57BL/6J and C57BL/6NTac and CBA/J	Heterozygous
17	49885651	81881415	C57BL/6J and C57BL/6NTac	N/A
17	81881415	94987271	C57BL/6J and C57BL/6NTac and CBA/J	Heterozygous
18	3000000	17841108	C57BL/6J and C57BL/6NTac and CBA/J	Heterozygous
18	17841108	90702639	CBA/J	Homozygous

MiniMUGA Background Analysis v2.3.1

	19	3000000	31636352	C57BL/6J and C57BL/6NTac	N/A
	19	31636352	61431566	C57BL/6J and C57BL/6NTac and CBA/J	Heterozygous
	X	3000000	70193631	C57BL/6J and C57BL/6NTac and CBA/J	Heterozygous
	X	70193631	105020820	C57BL/6J and C57BL/6NTac	N/A
	X	105020820	136441962	CBA/J	Homozygous
	X	136441962	171031299	C57BL/6J and C57BL/6NTac and CBA/J	Heterozygous
	MT	0	0	IBD	Hemizygous
