# Supplementary material for: Deletion of 9p drives B-ALL through heterozygous inactivation of Pax5 and Cd72 in preleukemic cells
Source: JCI Insight. 2026 Feb 17;11(7):e199464. doi: 10.1172/jci.insight.199464 (PMC13134721; doi:10.1172/jci.insight.199464)
Supplement: Supplemental data set 1 [file jciinsight-11-199464-s204.zip › Strain_Genotyping/Q304-results-report.pdf]

MiniMUGA Background Analysis v2.3.1

<table>
<tr><td>Sample ID</td><td>Q304</td></tr>
<tr><td>Neogen ID</td><td>AAAU-4525</td></tr>
<tr><td>Summary</td><td>The genotype of this sample is of excellent quality. It is XO and outbred, and likely a mix of C57BL/6J and C57BL/6NTac and CBA/J. Clustering of unexplained markers is evidence of an additional background strain.

Diagnostic SNPs are likely explained by the presence of the background strains
• Solution 1: 129S5/SvEvBrd and C57BL/6J and C57BL/6NTac
◦ C57BL/6J: 68 / 162 (42.0%)
◦ C57BL/6NTac: 9 / 27 (33.3%)
◦ 129S5/SvEvBrd: 1 / 5 (20.0%)
• Solution 2: 129S5/SvEvBrd and C57BL/6J and C57BL/6NRj
◦ C57BL/6J: 68 / 162 (42.0%)
◦ C57BL/6NRj: 9 / 27 (33.3%)
◦ 129S5/SvEvBrd: 1 / 5 (20.0%)
• Solution 3: 129S5/SvEvBrd and C57BL/6JRj and C57BL/6NTac
◦ C57BL/6JRj: 68 / 162 (42.0%)
◦ C57BL/6NTac: 9 / 27 (33.3%)
◦ 129S5/SvEvBrd: 1 / 5 (20.0%)
• Solution 4: 129S5/SvEvBrd and C57BL/6JRj and C57BL/6NRj
◦ C57BL/6JRj: 68 / 162 (42.0%)
◦ C57BL/6NRj: 9 / 27 (33.3%)
◦ 129S5/SvEvBrd: 1 / 5 (20.0%)

NOTE: There is a discrepancy between the diagnostic backgrounds detected and the primary and secondary background analysis (CBA/J, C57BL/6J, C57BL/6NTac). This is uncommon and should be investigated further.

No genetic constructs were detected in this sample.

WARNING:
• There is a discrepancy between the diagnostic backgrounds detected ((129S5/SvEvBrd and C57BL/6J and C57BL/6NTac) or (129S5/SvEvBrd and C57BL/6J and C57BL/6NRj) or (129S5/SvEvBrd and C57BL/6JRj and C57BL/6NTac) or (129S5/SvEvBrd and C57BL/6JRj and C57BL/6NRj)) and the primary background (C57BL/6J and C57BL/6NTac) and secondary background (CBA/J). This is uncommon and should be investigated further.
• The presence of a single diagnostic heterozygous call for a single inbred strain should be treated with caution.
• This sample likely has more than 2 genetic backgrounds (unexplained regions and/or fractured ideogram). The strain selected for secondary background may be incorrect. The estimation of the contribution of primary and secondary background are likely incorrect. This can potentially be addressed with input from the user.</td></tr>
<tr><td>Genotyping Quality</td><td>Excellent (74 N calls)
All reported results are dependent on genotyping quality.</td></tr>
<tr><td>Chromosomal Sex</td><td>XO</td></tr>
<tr><td>Inbreeding Estimate</td><td>54.7% Inbred
(Percentage of the genome (autosomal and X chromosomes) that is homozygous or hemizygous for primary, secondary, and unknown backgrounds. See Genome Analysis)</td></tr>
<tr><td>Constructs Detected</td><td>

BlastR	bpA	Cas9	chlor	cHS4	Cre	DTA	Flp	g_FP	hCMV_a	hCMV_b	hTK_pr	iCre	IRES	Luc	r_FP	rtTA	SV40	tTA
-	-	-	-	-	-	-	-	-	-	-	-	-	-	-	-	-	-	-

* This sample has abnormal raw intensity values. Therefore, positive or questionable returns for constructs should be treated with caution.</td></tr>
</table>

MiniMUGA Background Analysis v2.3.1

Refined Ideogram

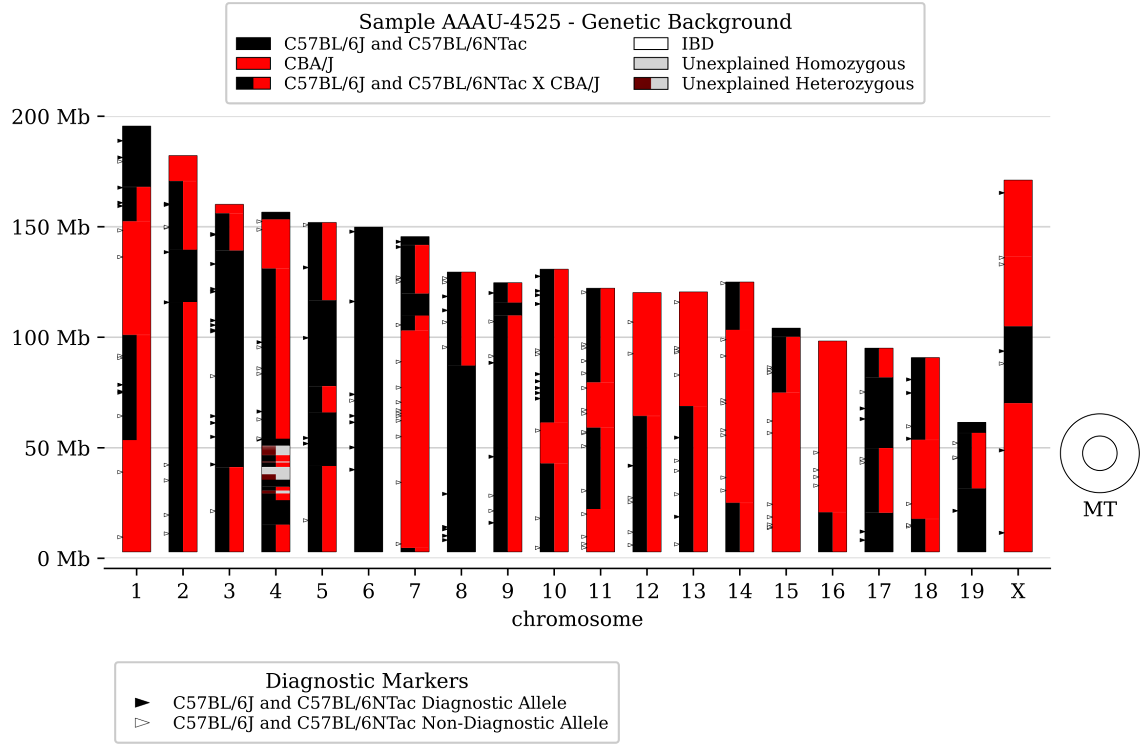

Genome Analysis

Background	Zygosity	Informative Markers	Informative Markers %	Genome %
C57BL/6J and C57BL/6NTac	N/A	743	28.5%	23.5%
CBA/J	Homozygous	680	26.1%	27.1%
C57BL/6J and C57BL/6NTac X CBA/J	Heterozygous	1168	44.8%	48.9%
Unexplained	Homozygous	5	0.2%	0.1%
Unexplained	Heterozygous	10	0.4%	0.3%
	**Total**	2606	100.0%	99.9%

**Y Chromosome**	Not Applicable
**MT Genome**	MT Haplogroup 6 - 100.0% Consistent<br>Includes C57BL/6J, C57BL/6NTac, CBA/J and 165 other strains

MiniMUGA Background Analysis v2.3.1

Backgrounds Detected (Diagnostic Alleles)

Diagnostic Class	Diagnostic Alleles Observed: Homozygous	Heterozygous	Potential	% Observed
C57BL/6J, C57BL/6JJicTac, C57BL/6JRj	12	30	102	41.2%
C57BL/6J, C57BL/6JRj	4	7	31	35.5%
C57BL/6J, C57BL/6JEiJ, C57BL/6JJicTac, C57BL/6JRj	1	9	21	47.6%
C57BL/6NJ, C57BL/6NRj, C57BL/6NTac	0	4	10	40.0%
C57BL/6NRj, C57BL/6NTac	0	4	15	26.7%
B6N-Tyr<c-Brd>/BrdCrCrl, C57BL/6J, C57BL/6JJicTac, C57BL/6JRj	0	3	5	60.0%
129S5/SvEvBrd	0	1	5	20.0%
B6N-Tyr<c-Brd>/BrdCrCrl, C57BL/6J, C57BL/6JBomTac, C57BL/6JEiJ, C57BL/6JJicTac, C57BL/6JOlaHsd, C57BL/6JRj	0	1	2	50.0%
B6N-Tyr<c-Brd>/BrdCrCrl, C57BL/6J, C57BL/6JEiJ, C57BL/6JJicTac, C57BL/6JRj	0	1	1	100.0%
B6N-Tyr<c-Brd>/BrdCrCrl, C57BL/6NCrl, C57BL/6NHsd, C57BL/6NJ, C57BL/6NRj, C57BL/6NTac	0	1	2	50.0%

Minimal Strain Sets Explaining All Diagnostic Classes (Number of Markers Explained):

- Solution 1: 129S5/SvEvBrd and C57BL/6J and C57BL/6NTac
  - C57BL/6J: 68 / 162 (42.0%)
  - C57BL/6NTac: 9 / 27 (33.3%)
  - 129S5/SvEvBrd: 1 / 5 (20.0%)
- Solution 2: 129S5/SvEvBrd and C57BL/6J and C57BL/6NRj
  - C57BL/6J: 68 / 162 (42.0%)
  - C57BL/6NRj: 9 / 27 (33.3%)
  - 129S5/SvEvBrd: 1 / 5 (20.0%)
- Solution 3: 129S5/SvEvBrd and C57BL/6JRj and C57BL/6NTac
  - C57BL/6JRj: 68 / 162 (42.0%)
  - C57BL/6NTac: 9 / 27 (33.3%)
  - 129S5/SvEvBrd: 1 / 5 (20.0%)
- Solution 4: 129S5/SvEvBrd and C57BL/6JRj and C57BL/6NRj
  - C57BL/6JRj: 68 / 162 (42.0%)
  - C57BL/6NRj: 9 / 27 (33.3%)
  - 129S5/SvEvBrd: 1 / 5 (20.0%)

Chromosome	Start (Mb)	Stop (Mb)	Background	Zygosity
1	3000000	53457225	CBA/J	Homozygous
1	53457225	101065154	C57BL/6J and C57BL/6NTac and CBA/J	Heterozygous
1	101065154	152511643	CBA/J	Homozygous
1	152511643	168019536	C57BL/6J and C57BL/6NTac and CBA/J	Heterozygous
1	168019536	195471971	C57BL/6J and C57BL/6NTac	N/A
2	3000000	115970567	C57BL/6J and C57BL/6NTac and CBA/J	Heterozygous
2	115970567	139631657	C57BL/6J and C57BL/6NTac	N/A
2	139631657	170694096	C57BL/6J and C57BL/6NTac and CBA/J	Heterozygous
2	170694096	182113224	CBA/J	Homozygous

MiniMUGA Background Analysis v2.3.1

**Diplotype Intervals**	3	3000000	41230643	C57BL/6J and C57BL/6NTac and CBA/J	Heterozygous
	3	41230643	139297311	C57BL/6J and C57BL/6NTac	N/A
	3	139297311	156090101	C57BL/6J and C57BL/6NTac and CBA/J	Heterozygous
	3	156090101	160039680	CBA/J	Homozygous
	4	3000000	15188739	C57BL/6J and C57BL/6NTac and CBA/J	Heterozygous
	4	15188739	26280383	C57BL/6J and C57BL/6NTac	N/A
	4	26280383	29346519	C57BL/6J and C57BL/6NTac and CBA/J	Heterozygous
	4	29346519	30650814	Unexplained	Heterozygous
	4	30650814	32327128	C57BL/6J and C57BL/6NTac and CBA/J	Heterozygous
	4	32327128	35563307	C57BL/6J and C57BL/6NTac	N/A
	4	35563307	37995481	Unexplained	Heterozygous
	4	37995481	41348396	Unexplained	Homozygous
	4	41348396	43372387	C57BL/6J and C57BL/6NTac and CBA/J	Heterozygous
	4	43372387	43819249	Unexplained	Heterozygous
	4	43819249	46665692	C57BL/6J and C57BL/6NTac and CBA/J	Heterozygous
	4	46665692	50929602	Unexplained	Heterozygous
	4	50929602	54114833	C57BL/6J and C57BL/6NTac	N/A
	4	54114833	131104093	C57BL/6J and C57BL/6NTac and CBA/J	Heterozygous
	4	131104093	153356388	CBA/J	Homozygous
	4	153356388	156508116	C57BL/6J and C57BL/6NTac	N/A
	5	3000000	41755530	C57BL/6J and C57BL/6NTac and CBA/J	Heterozygous
	5	41755530	66015308	C57BL/6J and C57BL/6NTac	N/A
	5	66015308	77895581	C57BL/6J and C57BL/6NTac and CBA/J	Heterozygous
	5	77895581	116795433	C57BL/6J and C57BL/6NTac	N/A
	5	116795433	151834684	C57BL/6J and C57BL/6NTac and CBA/J	Heterozygous
	6	3000000	149736546	C57BL/6J and C57BL/6NTac	N/A
	7	3000000	4674486	C57BL/6J and C57BL/6NTac and CBA/J	Heterozygous
	7	4674486	103084424	CBA/J	Homozygous
	7	103084424	109805667	C57BL/6J and C57BL/6NTac and CBA/J	Heterozygous
	7	109805667	119823617	C57BL/6J and C57BL/6NTac	N/A
	7	119823617	141750158	C57BL/6J and C57BL/6NTac and CBA/J	Heterozygous
	7	141750158	145441459	C57BL/6J and C57BL/6NTac	N/A

MiniMUGA Background Analysis v2.3.1

	8	3000000	87222027	C57BL/6J and C57BL/6NTac	N/A
	8	87222027	129401213	C57BL/6J and C57BL/6NTac and CBA/J	Heterozygous
	9	3000000	109855467	C57BL/6J and C57BL/6NTac and CBA/J	Heterozygous
	9	109855467	115715944	C57BL/6J and C57BL/6NTac	N/A
	9	115715944	124595110	C57BL/6J and C57BL/6NTac and CBA/J	Heterozygous
	10	3000000	42917049	C57BL/6J and C57BL/6NTac and CBA/J	Heterozygous
	10	42917049	61450853	CBA/J	Homozygous
	10	61450853	130694993	C57BL/6J and C57BL/6NTac and CBA/J	Heterozygous
	11	3000000	22302070	CBA/J	Homozygous
	11	22302070	59127711	C57BL/6J and C57BL/6NTac and CBA/J	Heterozygous
	11	59127711	79617327	CBA/J	Homozygous
	11	79617327	122082543	C57BL/6J and C57BL/6NTac and CBA/J	Heterozygous
	12	3000000	64411355	C57BL/6J and C57BL/6NTac and CBA/J	Heterozygous
	12	64411355	120129022	CBA/J	Homozygous
	13	3000000	68886272	C57BL/6J and C57BL/6NTac and CBA/J	Heterozygous
	13	68886272	120421639	CBA/J	Homozygous
	14	3000000	25112834	C57BL/6J and C57BL/6NTac and CBA/J	Heterozygous
	14	25112834	103377147	CBA/J	Homozygous
	14	103377147	124902244	C57BL/6J and C57BL/6NTac and CBA/J	Heterozygous
	15	3000000	74996398	CBA/J	Homozygous
	15	74996398	100173036	C57BL/6J and C57BL/6NTac and CBA/J	Heterozygous
	15	100173036	104043685	C57BL/6J and C57BL/6NTac	N/A
	16	3000000	20813513	C57BL/6J and C57BL/6NTac and CBA/J	Heterozygous
	16	20813513	98207768	CBA/J	Homozygous
	17	3000000	20616647	C57BL/6J and C57BL/6NTac	N/A
	17	20616647	49885651	C57BL/6J and C57BL/6NTac and CBA/J	Heterozygous
	17	49885651	81881415	C57BL/6J and C57BL/6NTac	N/A
	17	81881415	94987271	C57BL/6J and C57BL/6NTac and CBA/J	Heterozygous
	18	3000000	17841108	C57BL/6J and C57BL/6NTac and CBA/J	Heterozygous
	18	17841108	53636096	CBA/J	Homozygous
	18	53636096	90702639	C57BL/6J and C57BL/6NTac and CBA/J	Heterozygous
	19	3000000	31636352	C57BL/6J and C57BL/6NTac	N/A

MiniMUGA Background Analysis v2.3.1

	19	31636352	56751518	C57BL/6J and C57BL/6NTac and CBA/J	Heterozygous
	19	56751518	61431566	C57BL/6J and C57BL/6NTac	N/A
	X	3000000	70193631	C57BL/6J and C57BL/6NTac and CBA/J	Hemizygous
	X	70193631	105020820	C57BL/6J and C57BL/6NTac	Hemizygous
	X	105020820	136441962	CBA/J	Hemizygous
	X	136441962	171031299	C57BL/6J and C57BL/6NTac and CBA/J	Hemizygous
	MT	0	0	IBD	Hemizygous
