# Supplementary material for: Deletion of 9p drives B-ALL through heterozygous inactivation of Pax5 and Cd72 in preleukemic cells
Source: JCI Insight. 2026 Feb 17;11(7):e199464. doi: 10.1172/jci.insight.199464 (PMC13134721; doi:10.1172/jci.insight.199464)
Supplement: Supplemental data set 1 [file jciinsight-11-199464-s204.zip › Strain_Genotyping/Q305-results-report.pdf]

MiniMUGA Background Analysis v2.3.1

**Sample ID**	Q305
**Neogen ID**	AAAU-4524
**Summary**	The genotype of this sample is of **excellent** quality. It is **female** and **outbred**, and likely a mix of **C57BL/6J and C57BL/6NTac** and **CBA/J**. Clustering of unexplained markers is evidence of an additional background strain.<br><br>Diagnostic SNPs are likely explained by the presence of the background strains<br>• Solution 1: 129S5/SvEvBrd and C57BL/6J and C57BL/6NRj<br>◦ C57BL/6J: 90 / 164 (54.9%)<br>◦ C57BL/6NRj: 20 / 40 (50.0%)<br>◦ 129S5/SvEvBrd: 1 / 5 (20.0%)<br>• Solution 2: 129S5/SvEvBrd and C57BL/6JRj and C57BL/6NRj<br>◦ C57BL/6JRj: 90 / 164 (54.9%)<br>◦ C57BL/6NRj: 20 / 40 (50.0%)<br>◦ 129S5/SvEvBrd: 1 / 5 (20.0%)<br><br>NOTE: There is a discrepancy between the diagnostic backgrounds detected and the primary and secondary background analysis (CBA/J, C57BL/6J, C57BL/6NTac). This is uncommon and should be investigated further.<br><br>No genetic constructs were detected in this sample.<br><br>WARNING:<br>• There is a discrepancy between the diagnostic backgrounds detected ((129S5/SvEvBrd and C57BL/6J and C57BL/6NRj) or (129S5/SvEvBrd and C57BL/6JRj and C57BL/6NRj)) and the primary background (C57BL/6J and C57BL/6NTac) and secondary background (CBA/J). This is uncommon and should be investigated further.<br>• The presence of a single diagnostic heterozygous call for a single inbred strain should be treated with caution.<br>• This sample likely has more than 2 genetic backgrounds (unexplained regions and/or fractured ideogram). The strain selected for secondary background may be incorrect. The estimation of the contribution of primary and secondary background are likely incorrect. This can potentially be addressed with input from the user.
**Genotyping Quality**	**Excellent (9 N calls)**<br>All reported results are dependent on genotyping quality.
**Chromosomal Sex**	XX
**Inbreeding Estimate**	54.2% Inbred<br>(Percentage of the genome (autosomal and X chromosomes) that is homozygous or hemizygous for primary, secondary, and unknown backgrounds. See Genome Analysis)

Constructs Detected

BlastR	bpA	Cas9	chlor	cHS4	Cre	DTA	Flp	g_FP	hCMV_a	hCMV_b	hTK_pr	iCre	IRES	Luc	r_FP	rtTA	SV40	tTA
-	-	-	-	-	-	-	-	-	-	-	-	-	-	-	-	-	-	-

MiniMUGA Background Analysis v2.3.1

Refined Ideogram

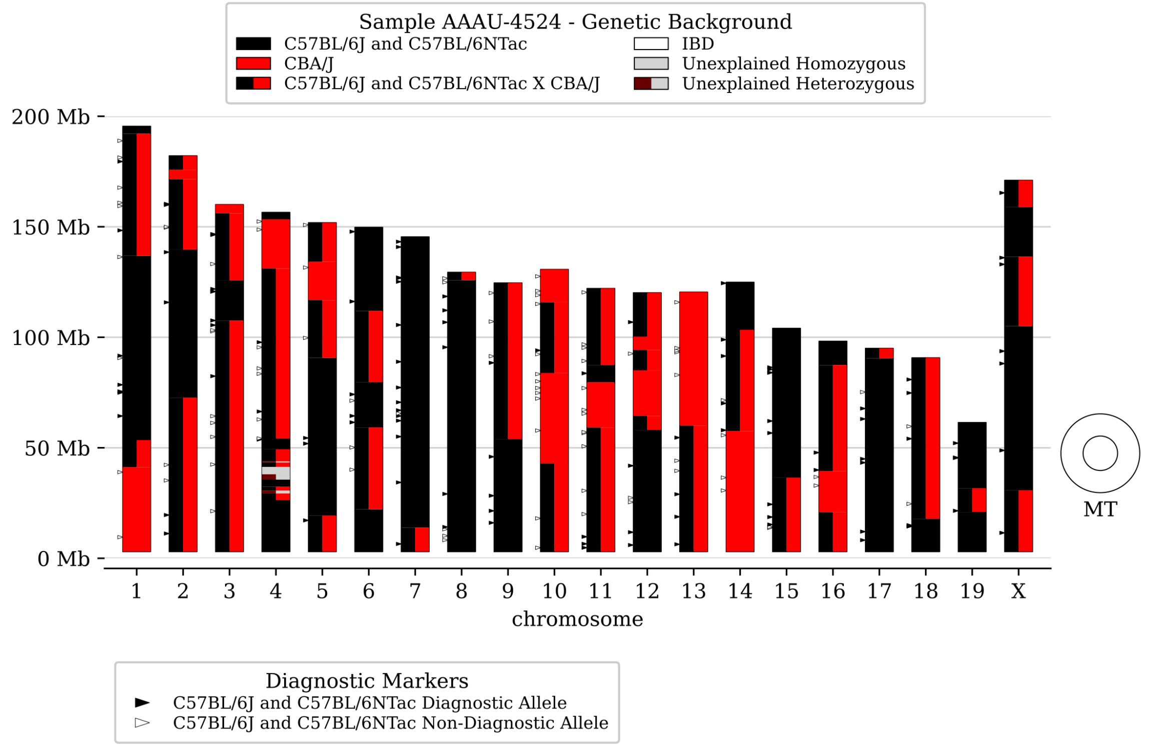

Genome Analysis

Background	Zygosity	Informative Markers	Informative Markers %	Genome %
C57BL/6J and C57BL/6NTac	N/A	1210	45.6%	41.6%
CBA/J	Homozygous	345	13.0%	12.5%
C57BL/6J and C57BL/6NTac X CBA/J	Heterozygous	1081	40.8%	45.6%
Unexplained	Homozygous	5	0.2%	0.1%
Unexplained	Heterozygous	10	0.4%	0.2%
	**Total**	2651	100.0%	100.0%

Y Chromosome

Not Applicable

MT Genome

MT Haplogroup 6 - 100.0% Consistent
Includes C57BL/6J, C57BL/6NTac, CBA/J and 165 other strains

MiniMUGA Background Analysis v2.3.1

Backgrounds Detected (Diagnostic Alleles)

Diagnostic Class	Diagnostic Alleles Observed: Homozygous	Heterozygous	Potential	% Observed
C57BL/6J, C57BL/6JJicTac, C57BL/6JRj	7	53	102	58.8%
C57BL/6J, C57BL/6JRj	2	11	31	41.9%
C57BL/6J, C57BL/6JEiJ, C57BL/6JJicTac, C57BL/6JRj	1	12	21	61.9%
C57BL/6NRj, C57BL/6NTac	0	9	15	60.0%
C57BL/6NJ, C57BL/6NRj, C57BL/6NTac	0	6	10	60.0%
C57BL/6NCrl, C57BL/6NHsd, C57BL/6NJ, C57BL/6NRj, C57BL/6NTac	0	2	2	100.0%
129S5/SvEvBrd	0	1	5	20.0%
B6N-Tyr<c-Brd>/BrdCrCrl, C57BL/6J, C57BL/6JBomTac, C57BL/6JEiJ, C57BL/6JJicTac, C57BL/6JOlaHsd, C57BL/6JRj	0	1	2	50.0%
B6N-Tyr<c-Brd>/BrdCrCrl, C57BL/6J, C57BL/6JEiJ, C57BL/6JJicTac, C57BL/6JRj	0	1	1	100.0%
B6N-Tyr<c-Brd>/BrdCrCrl, C57BL/6J, C57BL/6JJicTac, C57BL/6JRj	0	1	5	20.0%
B6N-Tyr<c-Brd>/BrdCrCrl, C57BL/6NCrl, C57BL/6NHsd, C57BL/6NJ, C57BL/6NRj, C57BL/6NTac	0	1	2	50.0%
C57BL/6J, C57BL/6JBomTac, C57BL/6JEiJ, C57BL/6JJicTac, C57BL/6JOlaHsd, C57BL/6JRj	0	1	2	50.0%
C57BL/6NHsd, C57BL/6NJ, C57BL/6NRj, C57BL/6NTac	0	1	1	100.0%
C57BL/6NRj	0	1	10	10.0%

Minimal Strain Sets Explaining All Diagnostic Classes (Number of Markers Explained):

- Solution 1: 129S5/SvEvBrd and C57BL/6J and C57BL/6NRj
    - C57BL/6J: 90 / 164 (54.9%)
    - C57BL/6NRj: 20 / 40 (50.0%)
    - 129S5/SvEvBrd: 1 / 5 (20.0%)
- Solution 2: 129S5/SvEvBrd and C57BL/6JRj and C57BL/6NRj
    - C57BL/6JRj: 90 / 164 (54.9%)
    - C57BL/6NRj: 20 / 40 (50.0%)
    - 129S5/SvEvBrd: 1 / 5 (20.0%)

Chromosome	Start (Mb)	Stop (Mb)	Background	Zygosity
1	3000000	41199760	CBA/J	Homozygous
1	41199760	53457225	C57BL/6J and C57BL/6NTac and CBA/J	Heterozygous
1	53457225	136798402	C57BL/6J and C57BL/6NTac	N/A
1	136798402	192078159	C57BL/6J and C57BL/6NTac and CBA/J	Heterozygous
1	192078159	195471971	C57BL/6J and C57BL/6NTac	N/A
2	3000000	72629186	C57BL/6J and C57BL/6NTac and CBA/J	Heterozygous
2	72629186	139631657	C57BL/6J and C57BL/6NTac	N/A
2	139631657	171484731	C57BL/6J and C57BL/6NTac and CBA/J	Heterozygous
2	171484731	175780822	CBA/J	Homozygous

MiniMUGA Background Analysis v2.3.1

Diplotype Intervals	2	175780822	182113224	C57BL/6J and C57BL/6NTac and CBA/J	Heterozygous
	3	3000000	107536398	C57BL/6J and C57BL/6NTac and CBA/J	Heterozygous
	3	107536398	125708355	C57BL/6J and C57BL/6NTac	N/A
	3	125708355	156090101	C57BL/6J and C57BL/6NTac and CBA/J	Heterozygous
	3	156090101	160039680	CBA/J	Homozygous
	4	3000000	26280383	C57BL/6J and C57BL/6NTac	N/A
	4	26280383	29346519	C57BL/6J and C57BL/6NTac and CBA/J	Heterozygous
	4	29346519	30650814	Unexplained	Heterozygous
	4	30650814	32327128	C57BL/6J and C57BL/6NTac and CBA/J	Heterozygous
	4	32327128	35563307	C57BL/6J and C57BL/6NTac	N/A
	4	35563307	37995481	Unexplained	Heterozygous
	4	37995481	41348396	Unexplained	Homozygous
	4	41348396	43372387	C57BL/6J and C57BL/6NTac and CBA/J	Heterozygous
	4	43372387	43819249	Unexplained	Heterozygous
	4	43819249	49280860	C57BL/6J and C57BL/6NTac and CBA/J	Heterozygous
	4	49280860	54114833	C57BL/6J and C57BL/6NTac	N/A
	4	54114833	131104093	C57BL/6J and C57BL/6NTac and CBA/J	Heterozygous
	4	131104093	153356388	CBA/J	Homozygous
	4	153356388	156508116	C57BL/6J and C57BL/6NTac	N/A
	5	3000000	19267794	C57BL/6J and C57BL/6NTac and CBA/J	Heterozygous
	5	19267794	90681879	C57BL/6J and C57BL/6NTac	N/A
	5	90681879	116795433	C57BL/6J and C57BL/6NTac and CBA/J	Heterozygous
	5	116795433	134172373	CBA/J	Homozygous
	5	134172373	151834684	C57BL/6J and C57BL/6NTac and CBA/J	Heterozygous
	6	3000000	22152593	C57BL/6J and C57BL/6NTac	N/A
	6	22152593	59156970	C57BL/6J and C57BL/6NTac and CBA/J	Heterozygous
	6	59156970	79701235	C57BL/6J and C57BL/6NTac	N/A
	6	79701235	111891908	C57BL/6J and C57BL/6NTac and CBA/J	Heterozygous
	6	111891908	149736546	C57BL/6J and C57BL/6NTac	N/A
	7	3000000	13838979	C57BL/6J and C57BL/6NTac and CBA/J	Heterozygous
	7	13838979	145441459	C57BL/6J and C57BL/6NTac	N/A

MiniMUGA Background Analysis v2.3.1

	8	3000000	125832225	C57BL/6J and C57BL/6NTac	N/A
	8	125832225	129401213	C57BL/6J and C57BL/6NTac and CBA/J	Heterozygous
	9	3000000	53875687	C57BL/6J and C57BL/6NTac	N/A
	9	53875687	124595110	C57BL/6J and C57BL/6NTac and CBA/J	Heterozygous
	10	3000000	42858234	C57BL/6J and C57BL/6NTac and CBA/J	Heterozygous
	10	42858234	83779430	CBA/J	Homozygous
	10	83779430	115781736	C57BL/6J and C57BL/6NTac and CBA/J	Heterozygous
	10	115781736	130694993	CBA/J	Homozygous
	11	3000000	59127711	C57BL/6J and C57BL/6NTac and CBA/J	Heterozygous
	11	59127711	79617327	CBA/J	Homozygous
	11	79617327	87432699	C57BL/6J and C57BL/6NTac	N/A
	11	87432699	122082543	C57BL/6J and C57BL/6NTac and CBA/J	Heterozygous
	12	3000000	58069123	C57BL/6J and C57BL/6NTac	N/A
	12	58069123	64411355	C57BL/6J and C57BL/6NTac and CBA/J	Heterozygous
	12	64411355	85015902	CBA/J	Homozygous
	12	85015902	94246475	C57BL/6J and C57BL/6NTac and CBA/J	Heterozygous
	12	94246475	100284662	CBA/J	Homozygous
	12	100284662	120129022	C57BL/6J and C57BL/6NTac and CBA/J	Heterozygous
	13	3000000	60016573	C57BL/6J and C57BL/6NTac and CBA/J	Heterozygous
	13	60016573	120421639	CBA/J	Homozygous
	14	3000000	57544602	CBA/J	Homozygous
	14	57544602	103377147	C57BL/6J and C57BL/6NTac and CBA/J	Heterozygous
	14	103377147	124902244	C57BL/6J and C57BL/6NTac	N/A
	15	3000000	36473640	C57BL/6J and C57BL/6NTac and CBA/J	Heterozygous
	15	36473640	104043685	C57BL/6J and C57BL/6NTac	N/A
	16	3000000	20813513	C57BL/6J and C57BL/6NTac and CBA/J	Heterozygous
	16	20813513	39422954	CBA/J	Homozygous
	16	39422954	87403166	C57BL/6J and C57BL/6NTac and CBA/J	Heterozygous
	16	87403166	98207768	C57BL/6J and C57BL/6NTac	N/A
	17	3000000	90487634	C57BL/6J and C57BL/6NTac	N/A
	17	90487634	94987271	C57BL/6J and C57BL/6NTac and CBA/J	Heterozygous
	18	3000000	17841108	C57BL/6J and C57BL/6NTac	N/A

MiniMUGA Background Analysis v2.3.1

	18	17841108	90702639	C57BL/6J and C57BL/6NTac and CBA/J	Heterozygous
	19	3000000	20955280	C57BL/6J and C57BL/6NTac	N/A
	19	20955280	31636352	C57BL/6J and C57BL/6NTac and CBA/J	Heterozygous
	19	31636352	61431566	C57BL/6J and C57BL/6NTac	N/A
	X	3000000	30738124	C57BL/6J and C57BL/6NTac and CBA/J	Heterozygous
	X	30738124	105020820	C57BL/6J and C57BL/6NTac	N/A
	X	105020820	136441962	C57BL/6J and C57BL/6NTac and CBA/J	Heterozygous
	X	136441962	158871958	C57BL/6J and C57BL/6NTac	N/A
	X	158871958	171031299	C57BL/6J and C57BL/6NTac and CBA/J	Heterozygous
	MT	0	0	IBD	Hemizygous
