# Supplementary material for: Deletion of 9p drives B-ALL through heterozygous inactivation of Pax5 and Cd72 in preleukemic cells
Source: JCI Insight. 2026 Feb 17;11(7):e199464. doi: 10.1172/jci.insight.199464 (PMC13134721; doi:10.1172/jci.insight.199464)
Supplement: Supplemental data set 1 [file jciinsight-11-199464-s204.zip › Strain_Genotyping/Q306-results-report.pdf]

MiniMUGA Background Analysis v2.3.1

**Sample ID**	Q306
**Neogen ID**	AAAU-4526
**Summary**	The genotype of this sample is of **excellent** quality. It is **female** and **outbred**, and likely a mix of **C57BL/6J and C57BL/6NTac** and **CBA/J**. Clustering of unexplained markers is evidence of an additional background strain.<br><br>Diagnostic SNPs are likely explained by the presence of the background strains<br>• Solution 1: 129S5/SvEvBrd and C57BL/6J and C57BL/6NRj<br>◦ C57BL/6J: 97 / 163 (59.5%)<br>◦ C57BL/6NRj: 14 / 39 (35.9%)<br>◦ 129S5/SvEvBrd: 1 / 5 (20.0%)<br>• Solution 2: 129S5/SvEvBrd and C57BL/6JRj and C57BL/6NRj<br>◦ C57BL/6JRj: 97 / 163 (59.5%)<br>◦ C57BL/6NRj: 14 / 39 (35.9%)<br>◦ 129S5/SvEvBrd: 1 / 5 (20.0%)<br><br>NOTE: There is a discrepancy between the diagnostic backgrounds detected and the primary and secondary background analysis (CBA/J, C57BL/6J, C57BL/6NTac). This is uncommon and should be investigated further.<br><br>No genetic constructs were detected in this sample.<br><br>WARNING:<br>• There is a discrepancy between the diagnostic backgrounds detected ((129S5/SvEvBrd and C57BL/6J and C57BL/6NRj) or (129S5/SvEvBrd and C57BL/6JRj and C57BL/6NRj)) and the primary background (C57BL/6J and C57BL/6NTac) and secondary background (CBA/J). This is uncommon and should be investigated further.<br>• The presence of a single diagnostic heterozygous call for a single inbred strain should be treated with caution.<br>• This sample likely has more than 2 genetic backgrounds (unexplained regions and/or fractured ideogram). The strain selected for secondary background may be incorrect. The estimation of the contribution of primary and secondary background are likely incorrect. This can potentially be addressed with input from the user.
**Genotyping Quality**	**Excellent (9 N calls)**<br>All reported results are dependent on genotyping quality.
**Chromosomal Sex**	XX
**Inbreeding Estimate**	49.1% Inbred<br>(Percentage of the genome (autosomal and X chromosomes) that is homozygous or hemizygous for primary, secondary, and unknown backgrounds. See Genome Analysis)

Constructs Detected

BlastR	bpA	Cas9	chlor	cHS4	Cre	DTA	Flp	g_FP	hCMV_a	hCMV_b	hTK_pr	iCre	IRES	Luc	r_FP	rtTA	SV40	tTA
-	-	-	-	-	-	-	-	-	-	-	-	-	-	-	-	-	-	-

MiniMUGA Background Analysis v2.3.1

Refined Ideogram

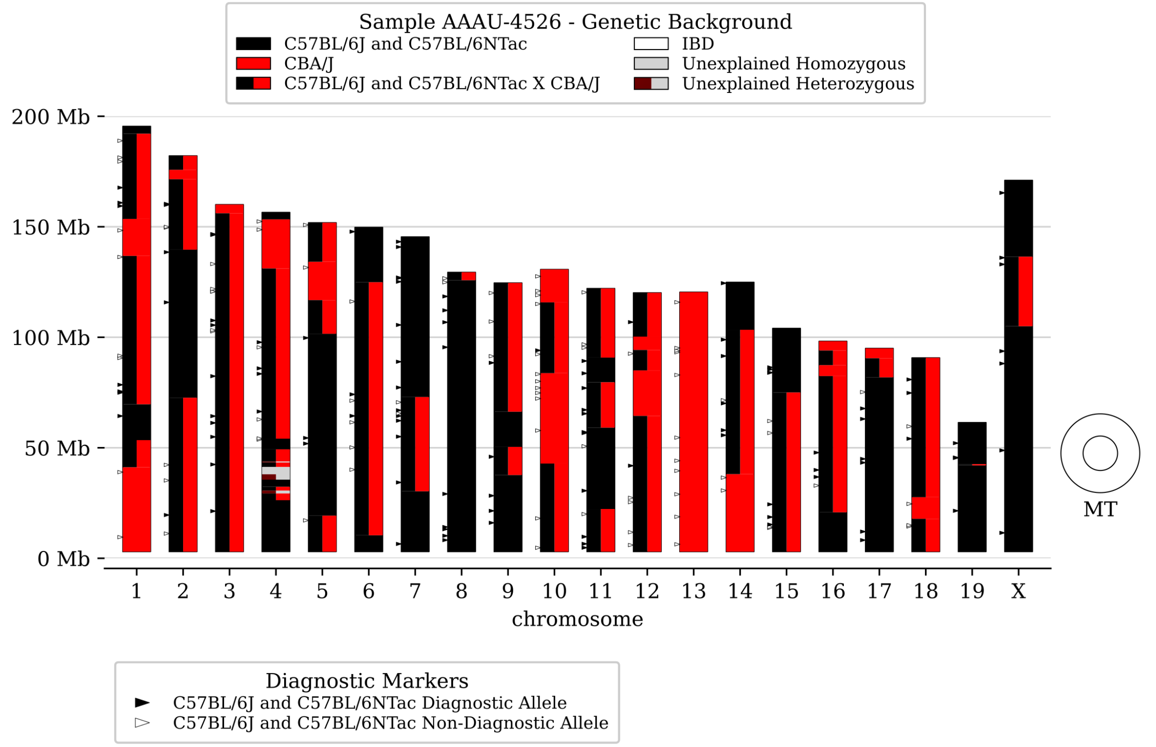

Genome Analysis

Background	Zygosity	Informative Markers	Informative Markers %	Genome %
C57BL/6J and C57BL/6NTac	N/A	1028	38.7%	34.9%
CBA/J	Homozygous	343	12.9%	14.0%
C57BL/6J and C57BL/6NTac X CBA/J	Heterozygous	1269	47.8%	50.7%
Unexplained	Homozygous	5	0.2%	0.1%
Unexplained	Heterozygous	10	0.4%	0.2%
	**Total**	2655	100.0%	99.9%

Y Chromosome

Not Applicable

MT Genome

MT Haplogroup 6 - 100.0% Consistent
Includes C57BL/6J, C57BL/6NTac, CBA/J and 165 other strains

MiniMUGA Background Analysis v2.3.1

Backgrounds Detected (Diagnostic Alleles)

Diagnostic Class	Diagnostic Alleles Observed: Homozygous	Heterozygous	Potential	% Observed
C57BL/6J, C57BL/6JJicTac, C57BL/6JRj	8	59	102	65.7%
C57BL/6J, C57BL/6JRj	4	11	31	48.4%
C57BL/6J, C57BL/6JEiJ, C57BL/6JJicTac, C57BL/6JRj	2	8	21	47.6%
C57BL/6NRj, C57BL/6NTac	0	6	15	40.0%
C57BL/6NJ, C57BL/6NRj, C57BL/6NTac	0	5	10	50.0%
B6N-Tyr<c-Brd>/BrdCrCrl, C57BL/6J, C57BL/6JJicTac, C57BL/6JRj	0	2	5	40.0%
129S5/SvEvBrd	0	1	5	20.0%
B6N-Tyr<c-Brd>/BrdCrCrl, C57BL/6J, C57BL/6JBomTac, C57BL/6JEiJ, C57BL/6JJicTac, C57BL/6JOlaHsd, C57BL/6JRj	0	1	2	50.0%
B6N-Tyr<c-Brd>/BrdCrCrl, C57BL/6J, C57BL/6JEiJ, C57BL/6JJicTac, C57BL/6JRj	0	1	1	100.0%
B6N-Tyr<c-Brd>/BrdCrCrl, C57BL/6NCrl, C57BL/6NHsd, C57BL/6NJ, C57BL/6NRj, C57BL/6NTac	0	1	2	50.0%
C57BL/6J, C57BL/6JEiJ, C57BL/6JJicTac, C57BL/6JOlaHsd, C57BL/6JRj	0	1	1	100.0%
C57BL/6NCrl, C57BL/6NHsd, C57BL/6NJ, C57BL/6NRj, C57BL/6NTac	0	1	2	50.0%
C57BL/6NRj	0	1	10	10.0%

Minimal Strain Sets Explaining All Diagnostic Classes (Number of Markers Explained):

- Solution 1: 129S5/SvEvBrd and C57BL/6J and C57BL/6NRj
  - C57BL/6J: 97 / 163 (59.5%)
  - C57BL/6NRj: 14 / 39 (35.9%)
  - 129S5/SvEvBrd: 1 / 5 (20.0%)
- Solution 2: 129S5/SvEvBrd and C57BL/6JRj and C57BL/6NRj
  - C57BL/6JRj: 97 / 163 (59.5%)
  - C57BL/6NRj: 14 / 39 (35.9%)
  - 129S5/SvEvBrd: 1 / 5 (20.0%)

Chromosome	Start (Mb)	Stop (Mb)	Background	Zygosity
1	3000000	41199760	CBA/J	Homozygous
1	41199760	53457225	C57BL/6J and C57BL/6NTac and CBA/J	Heterozygous
1	53457225	69700765	C57BL/6J and C57BL/6NTac	N/A
1	69700765	136798402	C57BL/6J and C57BL/6NTac and CBA/J	Heterozygous
1	136798402	153548642	CBA/J	Homozygous
1	153548642	192078159	C57BL/6J and C57BL/6NTac and CBA/J	Heterozygous
1	192078159	195471971	C57BL/6J and C57BL/6NTac	N/A
2	3000000	72629186	C57BL/6J and C57BL/6NTac and CBA/J	Heterozygous
2	72629186	139631657	C57BL/6J and C57BL/6NTac	N/A
2	139631657	171484731	C57BL/6J and C57BL/6NTac and CBA/J	Heterozygous

MiniMUGA Background Analysis v2.3.1

**Diplotype Intervals**	2	171484731	175780822	CBA/J	Homozygous
	2	175780822	182113224	C57BL/6J and C57BL/6NTac and CBA/J	Heterozygous
	3	3000000	156090101	C57BL/6J and C57BL/6NTac and CBA/J	Heterozygous
	3	156090101	160039680	CBA/J	Homozygous
	4	3000000	26280383	C57BL/6J and C57BL/6NTac	N/A
	4	26280383	29346519	C57BL/6J and C57BL/6NTac and CBA/J	Heterozygous
	4	29346519	30650814	Unexplained	Heterozygous
	4	30650814	32327128	C57BL/6J and C57BL/6NTac and CBA/J	Heterozygous
	4	32327128	35563307	C57BL/6J and C57BL/6NTac	N/A
	4	35563307	37995481	Unexplained	Heterozygous
	4	37995481	41348396	Unexplained	Homozygous
	4	41348396	43372387	C57BL/6J and C57BL/6NTac and CBA/J	Heterozygous
	4	43372387	43819249	Unexplained	Heterozygous
	4	43819249	49280860	C57BL/6J and C57BL/6NTac and CBA/J	Heterozygous
	4	49280860	54114833	C57BL/6J and C57BL/6NTac	N/A
	4	54114833	131104093	C57BL/6J and C57BL/6NTac and CBA/J	Heterozygous
	4	131104093	153356388	CBA/J	Homozygous
	4	153356388	156508116	C57BL/6J and C57BL/6NTac	N/A
	5	3000000	19267794	C57BL/6J and C57BL/6NTac and CBA/J	Heterozygous
	5	19267794	101581477	C57BL/6J and C57BL/6NTac	N/A
	5	101581477	116795433	C57BL/6J and C57BL/6NTac and CBA/J	Heterozygous
	5	116795433	134172373	CBA/J	Homozygous
	5	134172373	151834684	C57BL/6J and C57BL/6NTac and CBA/J	Heterozygous
	6	3000000	10424941	C57BL/6J and C57BL/6NTac	N/A
	6	10424941	124881471	C57BL/6J and C57BL/6NTac and CBA/J	Heterozygous
	6	124881471	149736546	C57BL/6J and C57BL/6NTac	N/A
	7	3000000	30335112	C57BL/6J and C57BL/6NTac	N/A
	7	30335112	72944748	C57BL/6J and C57BL/6NTac and CBA/J	Heterozygous
	7	72944748	145441459	C57BL/6J and C57BL/6NTac	N/A
	8	3000000	125832225	C57BL/6J and C57BL/6NTac	N/A
	8	125832225	129401213	C57BL/6J and C57BL/6NTac and CBA/J	Heterozygous
	9	3000000	37691490	C57BL/6J and C57BL/6NTac	N/A

9	37691490	50381732	C57BL/6J and C57BL/6NTac and CBA/J	Heterozygous
9	50381732	66341356	C57BL/6J and C57BL/6NTac	N/A
9	66341356	124595110	C57BL/6J and C57BL/6NTac and CBA/J	Heterozygous
10	3000000	42858234	C57BL/6J and C57BL/6NTac and CBA/J	Heterozygous
10	42858234	83779430	CBA/J	Homozygous
10	83779430	115781736	C57BL/6J and C57BL/6NTac and CBA/J	Heterozygous
10	115781736	130694993	CBA/J	Homozygous
11	3000000	22302070	C57BL/6J and C57BL/6NTac and CBA/J	Heterozygous
11	22302070	59127711	C57BL/6J and C57BL/6NTac	N/A
11	59127711	79617327	C57BL/6J and C57BL/6NTac and CBA/J	Heterozygous
11	79617327	90803561	C57BL/6J and C57BL/6NTac	N/A
11	90803561	122082543	C57BL/6J and C57BL/6NTac and CBA/J	Heterozygous
12	3000000	64411355	C57BL/6J and C57BL/6NTac and CBA/J	Heterozygous
12	64411355	85015902	CBA/J	Homozygous
12	85015902	94246475	C57BL/6J and C57BL/6NTac and CBA/J	Heterozygous
12	94246475	100284662	CBA/J	Homozygous
12	100284662	120129022	C57BL/6J and C57BL/6NTac and CBA/J	Heterozygous
13	3000000	120421639	CBA/J	Homozygous
14	3000000	38092288	CBA/J	Homozygous
14	38092288	103377147	C57BL/6J and C57BL/6NTac and CBA/J	Heterozygous
14	103377147	124902244	C57BL/6J and C57BL/6NTac	N/A
15	3000000	74996398	C57BL/6J and C57BL/6NTac and CBA/J	Heterozygous
15	74996398	104043685	C57BL/6J and C57BL/6NTac	N/A
16	3000000	20813513	C57BL/6J and C57BL/6NTac	N/A
16	20813513	82429429	C57BL/6J and C57BL/6NTac and CBA/J	Heterozygous
16	82429429	87403166	CBA/J	Homozygous
16	87403166	93997286	C57BL/6J and C57BL/6NTac and CBA/J	Heterozygous
16	93997286	98207768	CBA/J	Homozygous
17	3000000	81881415	C57BL/6J and C57BL/6NTac	N/A
17	81881415	90487634	C57BL/6J and C57BL/6NTac and CBA/J	Heterozygous
17	90487634	94987271	CBA/J	Homozygous
18	3000000	17841108	C57BL/6J and C57BL/6NTac and CBA/J	Heterozygous

MiniMUGA Background Analysis v2.3.1

	18	17841108	27650151	CBA/J	Homozygous
	18	27650151	90702639	C57BL/6J and C57BL/6NTac and CBA/J	Heterozygous
	19	3000000	42043276	C57BL/6J and C57BL/6NTac	N/A
	19	42043276	42582533	C57BL/6J and C57BL/6NTac and CBA/J	Heterozygous
	19	42582533	61431566	C57BL/6J and C57BL/6NTac	N/A
	X	3000000	105020820	C57BL/6J and C57BL/6NTac	N/A
	X	105020820	136441962	C57BL/6J and C57BL/6NTac and CBA/J	Heterozygous
	X	136441962	171031299	C57BL/6J and C57BL/6NTac	N/A
	MT	0	0	IBD	Hemizygous
