# Supplementary material for: Deletion of 9p drives B-ALL through heterozygous inactivation of Pax5 and Cd72 in preleukemic cells
Source: JCI Insight. 2026 Feb 17;11(7):e199464. doi: 10.1172/jci.insight.199464 (PMC13134721; doi:10.1172/jci.insight.199464)
Supplement: Supplemental data set 1 [file jciinsight-11-199464-s204.zip › Strain_Genotyping/Q421-results-report.pdf]

MiniMUGA Background Analysis v2.3.1

**Sample ID**	Q421
**Neogen ID**	AAAU-4528
**Summary**	The genotype of this sample is of **excellent** quality. It is **female** and **outbred**, and likely a mix of **C57BL/6J and C57BL/6NTac** and **CBA/J**. Clustering of unexplained markers is evidence of an additional background strain.<br><br>Diagnostic SNPs are likely explained by the presence of the background strains<br>• Solution 1: 129S5/SvEvBrd and C57BL/6J and C57BL/6NRj<br>◦ C57BL/6J: 78 / 162 (48.1%)<br>◦ C57BL/6NRj: 14 / 40 (35.0%)<br>◦ 129S5/SvEvBrd: 1 / 5 (20.0%)<br>• Solution 2: 129S5/SvEvBrd and C57BL/6JRj and C57BL/6NRj<br>◦ C57BL/6JRj: 78 / 162 (48.1%)<br>◦ C57BL/6NRj: 14 / 40 (35.0%)<br>◦ 129S5/SvEvBrd: 1 / 5 (20.0%)<br><br>NOTE: There is a discrepancy between the diagnostic backgrounds detected and the primary and secondary background analysis (CBA/J, C57BL/6J, C57BL/6NTac). This is uncommon and should be investigated further.<br><br>No genetic constructs were detected in this sample.<br><br>WARNING:<br>• There is a discrepancy between the diagnostic backgrounds detected ((129S5/SvEvBrd and C57BL/6J and C57BL/6NRj) or (129S5/SvEvBrd and C57BL/6JRj and C57BL/6NRj)) and the primary background (C57BL/6J and C57BL/6NTac) and secondary background (CBA/J). This is uncommon and should be investigated further.<br>• The presence of a single diagnostic heterozygous call for a single inbred strain should be treated with caution.<br>• This sample likely has more than 2 genetic backgrounds (unexplained regions and/or fractured ideogram). The strain selected for secondary background may be incorrect. The estimation of the contribution of primary and secondary background are likely incorrect. This can potentially be addressed with input from the user.
**Genotyping Quality**	**Excellent (14 N calls)**<br>All reported results are dependent on genotyping quality.
**Chromosomal Sex**	XX
**Inbreeding Estimate**	49.5% Inbred<br>(Percentage of the genome (autosomal and X chromosomes) that is homozygous or hemizygous for primary, secondary, and unknown backgrounds. See Genome Analysis)

Constructs Detected

BlastR	bpA	Cas9	chlor	cHS4	Cre	DTA	Flp	g_FP	hCMV_a	hCMV_b	hTK_pr	iCre	IRES	Luc	r_FP	rtTA	SV40	tTA
-	-	-	-	-	-	-	-	-	-	-	-	-	-	-	-	-	-	-

MiniMUGA Background Analysis v2.3.1

Refined Ideogram

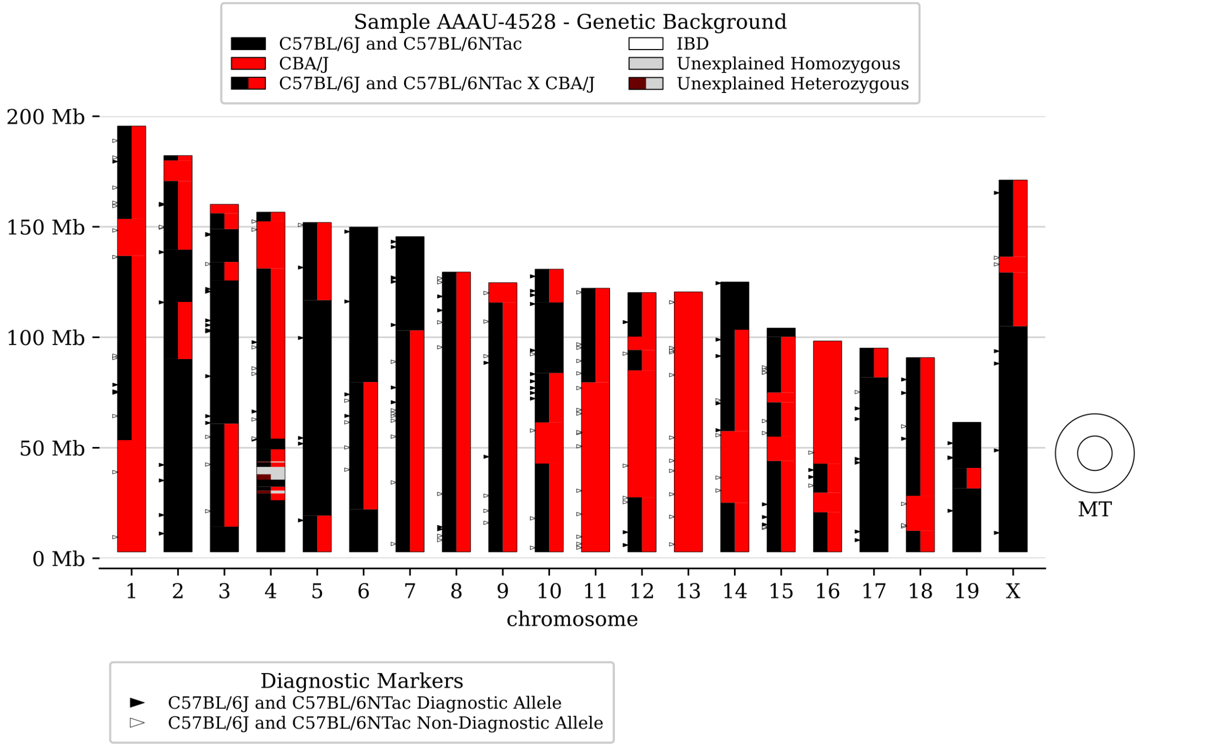

Genome Analysis

Background	Zygosity	Informative Markers	Informative Markers %	Genome %
C57BL/6J and C57BL/6NTac	N/A	845	31.9%	29.1%
CBA/J	Homozygous	455	17.2%	20.3%
C57BL/6J and C57BL/6NTac X CBA/J	Heterozygous	1334	50.4%	50.3%
Unexplained	Homozygous	5	0.2%	0.1%
Unexplained	Heterozygous	10	0.4%	0.2%
	**Total**	2649	100.1%	100.0%

Y Chromosome

Not Applicable

MT Genome

MT Haplogroup 6 - 100.0% Consistent
Includes C57BL/6J, C57BL/6NTac, CBA/J and 165 other strains

MiniMUGA Background Analysis v2.3.1

Backgrounds Detected (Diagnostic Alleles)

Diagnostic Class	Diagnostic Alleles Observed: Homozygous	Heterozygous	Potential	% Observed
C57BL/6J, C57BL/6JJicTac, C57BL/6JRj	7	45	102	51.0%
C57BL/6J, C57BL/6JRj	1	10	31	35.5%
C57BL/6J, C57BL/6JEiJ, C57BL/6JJicTac, C57BL/6JRj	1	8	21	42.9%
C57BL/6NRj, C57BL/6NTac	0	6	15	40.0%
C57BL/6NJ, C57BL/6NRj, C57BL/6NTac	0	4	10	40.0%
B6N-Tyr<c-Brd>/BrdCrCrl, C57BL/6J, C57BL/6JJicTac, C57BL/6JRj	0	3	5	60.0%
B6N-Tyr<c-Brd>/BrdCrCrl, C57BL/6J, C57BL/6JBomTac, C57BL/6JEiJ, C57BL/6JJicTac, C57BL/6JOlaHsd, C57BL/6JRj	0	2	2	100.0%
129S5/SvEvBrd	0	1	5	20.0%
B6N-Tyr<c-Brd>/BrdCrCrl, C57BL/6J, C57BL/6JEiJ, C57BL/6JJicTac, C57BL/6JRj	0	1	1	100.0%
B6N-Tyr<c-Brd>/BrdCrCrl, C57BL/6NCrl, C57BL/6NHsd, C57BL/6NJ, C57BL/6NRj, C57BL/6NTac	0	1	2	50.0%
C57BL/6NCrl, C57BL/6NHsd, C57BL/6NJ, C57BL/6NRj, C57BL/6NTac	0	1	2	50.0%
C57BL/6NHsd, C57BL/6NJ, C57BL/6NRj, C57BL/6NTac	0	1	1	100.0%
C57BL/6NRj	0	1	10	10.0%

Minimal Strain Sets Explaining All Diagnostic Classes (Number of Markers Explained):

- Solution 1: 129S5/SvEvBrd and C57BL/6J and C57BL/6NRj
    - C57BL/6J: 78 / 162 (48.1%)
    - C57BL/6NRj: 14 / 40 (35.0%)
    - 129S5/SvEvBrd: 1 / 5 (20.0%)
- Solution 2: 129S5/SvEvBrd and C57BL/6JRj and C57BL/6NRj
    - C57BL/6JRj: 78 / 162 (48.1%)
    - C57BL/6NRj: 14 / 40 (35.0%)
    - 129S5/SvEvBrd: 1 / 5 (20.0%)

Chromosome	Start (Mb)	Stop (Mb)	Background	Zygosity
1	3000000	53457225	CBA/J	Homozygous
1	53457225	136798402	C57BL/6J and C57BL/6NTac and CBA/J	Heterozygous
1	136798402	153548642	CBA/J	Homozygous
1	153548642	195471971	C57BL/6J and C57BL/6NTac and CBA/J	Heterozygous
2	3000000	90144439	C57BL/6J and C57BL/6NTac	N/A
2	90144439	115970567	C57BL/6J and C57BL/6NTac and CBA/J	Heterozygous
2	115970567	139631657	C57BL/6J and C57BL/6NTac	N/A
2	139631657	170694096	C57BL/6J and C57BL/6NTac and CBA/J	Heterozygous
2	170694096	179996376	CBA/J	Homozygous
2	179996376	182113224	C57BL/6J and C57BL/6NTac and CBA/J	Heterozygous

MiniMUGA Background Analysis v2.3.1

Diplotype Intervals	3	3000000	14328941	C57BL/6J and C57BL/6NTac	N/A
	3	14328941	60850190	C57BL/6J and C57BL/6NTac and CBA/J	Heterozygous
	3	60850190	125708355	C57BL/6J and C57BL/6NTac	N/A
	3	125708355	134049530	C57BL/6J and C57BL/6NTac and CBA/J	Heterozygous
	3	134049530	148967944	C57BL/6J and C57BL/6NTac	N/A
	3	148967944	156090101	C57BL/6J and C57BL/6NTac and CBA/J	Heterozygous
	3	156090101	160039680	CBA/J	Homozygous
	4	3000000	26280383	C57BL/6J and C57BL/6NTac	N/A
	4	26280383	29346519	C57BL/6J and C57BL/6NTac and CBA/J	Heterozygous
	4	29346519	30650814	Unexplained	Heterozygous
	4	30650814	32327128	C57BL/6J and C57BL/6NTac and CBA/J	Heterozygous
	4	32327128	35563307	C57BL/6J and C57BL/6NTac	N/A
	4	35563307	37995481	Unexplained	Heterozygous
	4	37995481	41348396	Unexplained	Homozygous
	4	41348396	43372387	C57BL/6J and C57BL/6NTac and CBA/J	Heterozygous
	4	43372387	43819249	Unexplained	Heterozygous
	4	43819249	49280860	C57BL/6J and C57BL/6NTac and CBA/J	Heterozygous
	4	49280860	54114833	C57BL/6J and C57BL/6NTac	N/A
	4	54114833	131104093	C57BL/6J and C57BL/6NTac and CBA/J	Heterozygous
	4	131104093	152440879	CBA/J	Homozygous
	4	152440879	156508116	C57BL/6J and C57BL/6NTac and CBA/J	Heterozygous
	5	3000000	19267794	C57BL/6J and C57BL/6NTac and CBA/J	Heterozygous
	5	19267794	116795433	C57BL/6J and C57BL/6NTac	N/A
	5	116795433	151834684	C57BL/6J and C57BL/6NTac and CBA/J	Heterozygous
	6	3000000	22152593	C57BL/6J and C57BL/6NTac	N/A
	6	22152593	79701235	C57BL/6J and C57BL/6NTac and CBA/J	Heterozygous
	6	79701235	149736546	C57BL/6J and C57BL/6NTac	N/A
	7	3000000	103084424	C57BL/6J and C57BL/6NTac and CBA/J	Heterozygous
	7	103084424	145441459	C57BL/6J and C57BL/6NTac	N/A
	8	3000000	129401213	C57BL/6J and C57BL/6NTac and CBA/J	Heterozygous
	9	3000000	115715944	C57BL/6J and C57BL/6NTac and CBA/J	Heterozygous

MiniMUGA Background Analysis v2.3.1

9	115715944	124595110	CBA/J	Homozygous
10	3000000	42858234	C57BL/6J and C57BL/6NTac and CBA/J	Heterozygous
10	42858234	61450853	CBA/J	Homozygous
10	61450853	83779430	C57BL/6J and C57BL/6NTac and CBA/J	Heterozygous
10	83779430	115781736	C57BL/6J and C57BL/6NTac	N/A
10	115781736	130694993	C57BL/6J and C57BL/6NTac and CBA/J	Heterozygous
11	3000000	79617327	CBA/J	Homozygous
11	79617327	122082543	C57BL/6J and C57BL/6NTac and CBA/J	Heterozygous
12	3000000	27585493	C57BL/6J and C57BL/6NTac and CBA/J	Heterozygous
12	27585493	85015902	CBA/J	Homozygous
12	85015902	94246475	C57BL/6J and C57BL/6NTac and CBA/J	Heterozygous
12	94246475	100284662	CBA/J	Homozygous
12	100284662	120129022	C57BL/6J and C57BL/6NTac and CBA/J	Heterozygous
13	3000000	120421639	CBA/J	Homozygous
14	3000000	25112834	C57BL/6J and C57BL/6NTac and CBA/J	Heterozygous
14	25112834	57544602	CBA/J	Homozygous
14	57544602	103377147	C57BL/6J and C57BL/6NTac and CBA/J	Heterozygous
14	103377147	124902244	C57BL/6J and C57BL/6NTac	N/A
15	3000000	44010563	C57BL/6J and C57BL/6NTac and CBA/J	Heterozygous
15	44010563	55016741	CBA/J	Homozygous
15	55016741	70554147	C57BL/6J and C57BL/6NTac and CBA/J	Heterozygous
15	70554147	74996398	CBA/J	Homozygous
15	74996398	100173036	C57BL/6J and C57BL/6NTac and CBA/J	Heterozygous
15	100173036	104043685	C57BL/6J and C57BL/6NTac	N/A
16	3000000	20813513	C57BL/6J and C57BL/6NTac and CBA/J	Heterozygous
16	20813513	29701002	CBA/J	Homozygous
16	29701002	42816746	C57BL/6J and C57BL/6NTac and CBA/J	Heterozygous
16	42816746	98207768	CBA/J	Homozygous
17	3000000	81881415	C57BL/6J and C57BL/6NTac	N/A
17	81881415	94987271	C57BL/6J and C57BL/6NTac and CBA/J	Heterozygous
18	3000000	12406382	C57BL/6J and C57BL/6NTac and CBA/J	Heterozygous
18	12406382	28258011	CBA/J	Homozygous
18	28258011	90702639	C57BL/6J and C57BL/6NTac and CBA/J	Heterozygous

	19	3000000	31636352	C57BL/6J and C57BL/6NTac	N/A
	19	31636352	40841805	C57BL/6J and C57BL/6NTac and CBA/J	Heterozygous
	19	40841805	61431566	C57BL/6J and C57BL/6NTac	N/A
	X	3000000	105020820	C57BL/6J and C57BL/6NTac	N/A
	X	105020820	129295190	C57BL/6J and C57BL/6NTac and CBA/J	Heterozygous
	X	129295190	136441962	CBA/J	Homozygous
	X	136441962	171031299	C57BL/6J and C57BL/6NTac and CBA/J	Heterozygous
	MT	0	0	IBD	Hemizygous
