# Supplementary material for: Deletion of 9p drives B-ALL through heterozygous inactivation of Pax5 and Cd72 in preleukemic cells
Source: JCI Insight. 2026 Feb 17;11(7):e199464. doi: 10.1172/jci.insight.199464 (PMC13134721; doi:10.1172/jci.insight.199464)
Supplement: Supplemental data set 1 [file jciinsight-11-199464-s204.zip › Strain_Genotyping/Q492-results-report.pdf]

MiniMUGA Background Analysis v2.3.1

<table>
<tr><th>Sample ID</th><td>Q492</td></tr>
<tr><th>Neogen ID</th><td>AAAU-4539</td></tr>
<tr><th>Summary</th><td>The genotype of this sample is of excellent quality. It is female and outbred, and likely a mix of C57BL/6J and C57BL/6NTac and CBA/J. Clustering of unexplained markers is evidence of an additional background strain.

Diagnostic SNPs are likely explained by the presence of the background strains

Solution 1: 129S5/SvEvBrd and C57BL/6J and C57BL/6NRj
C57BL/6J: 80 / 162 (49.4%)
C57BL/6NRj: 16 / 40 (40.0%)
129S5/SvEvBrd: 1 / 5 (20.0%)
Solution 2: 129S5/SvEvBrd and C57BL/6JRj and C57BL/6NRj
C57BL/6JRj: 80 / 162 (49.4%)
C57BL/6NRj: 16 / 40 (40.0%)
129S5/SvEvBrd: 1 / 5 (20.0%)

NOTE: There is a discrepancy between the diagnostic backgrounds detected and the primary and secondary background analysis (CBA/J, C57BL/6J, C57BL/6NTac). This is uncommon and should be investigated further.

No genetic constructs were detected in this sample.

WARNING:

There is a discrepancy between the diagnostic backgrounds detected ((129S5/SvEvBrd and C57BL/6J and C57BL/6NRj) or (129S5/SvEvBrd and C57BL/6JRj and C57BL/6NRj)) and the primary background (C57BL/6J and C57BL/6NTac) and secondary background (CBA/J). This is uncommon and should be investigated further.
The presence of a single diagnostic heterozygous call for a single inbred strain should be treated with caution.
This sample likely has more than 2 genetic backgrounds (unexplained regions and/or fractured ideogram). The strain selected for secondary background may be incorrect. The estimation of the contribution of primary and secondary background are likely incorrect. This can potentially be addressed with input from the user.
</td></tr>
<tr><th>Genotyping Quality</th><td>Excellent (5 N calls)
All reported results are dependent on genotyping quality.</td></tr>
<tr><th>Chromosomal Sex</th><td>XX</td></tr>
<tr><th>Inbreeding Estimate</th><td>52.6% Inbred
(Percentage of the genome (autosomal and X chromosomes) that is homozygous or hemizygous for primary, secondary, and unknown backgrounds. See Genome Analysis)</td></tr>
<tr><th>Constructs Detected</th><td>

BlastR	bpA	Cas9	chlor	cHS4	Cre	DTA	Flp	g_FP	hCMV_a	hCMV_b	hTK_pr	iCre	IRES	Luc	r_FP	rtTA	SV40	tTA
-	-	-	-	-	-	-	-	-	-	-	-	-	-	-	-	-	-	-

</td></tr>
</table>

MiniMUGA Background Analysis v2.3.1

Refined Ideogram

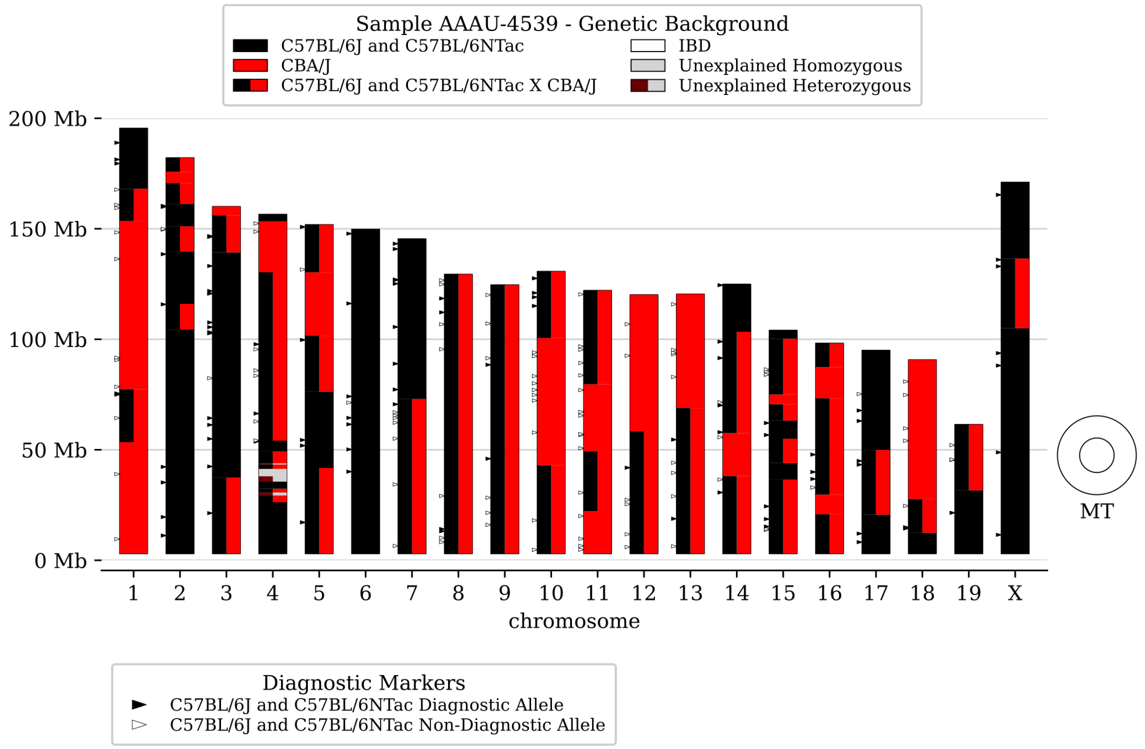

Genome Analysis

Background	Zygosity	Informative Markers	Informative Markers %	Genome %
C57BL/6J and C57BL/6NTac	N/A	888	33.4%	32.3%
CBA/J	Homozygous	488	18.3%	20.1%
C57BL/6J and C57BL/6NTac X CBA/J	Heterozygous	1269	47.7%	47.3%
Unexplained	Homozygous	5	0.2%	0.1%
Unexplained	Heterozygous	10	0.4%	0.2%
	**Total**	2660	100.0%	100.0%

**Y Chromosome**	Not Applicable
**MT Genome**	MT Haplogroup 6 - 100.0% Consistent<br>Includes C57BL/6J, C57BL/6NTac, CBA/J and 165 other strains

MiniMUGA Background Analysis v2.3.1

Backgrounds Detected (Diagnostic Alleles)

Diagnostic Class	Diagnostic Alleles Observed			
	Homozygous	Heterozygous	Potential	% Observed
C57BL/6J, C57BL/6JJicTac, C57BL/6JRj	13	39	102	51.0%
C57BL/6J, C57BL/6JRj	4	8	31	38.7%
C57BL/6J, C57BL/6JEiJ, C57BL/6JJicTac, C57BL/6JRj	2	11	21	61.9%
C57BL/6NRj, C57BL/6NTac	0	7	15	46.7%
C57BL/6NJ, C57BL/6NRj, C57BL/6NTac	0	4	10	40.0%
C57BL/6NCrl, C57BL/6NHsd, C57BL/6NJ, C57BL/6NRj, C57BL/6NTac	0	2	2	100.0%
129S5/SvEvBrd	0	1	5	20.0%
B6N-Tyr<c-Brd>/BrdCrCrl, C57BL/6J, C57BL/6JBomTac, C57BL/6JEiJ, C57BL/6JJicTac, C57BL/6JOlaHsd, C57BL/6JRj	0	1	2	50.0%
B6N-Tyr<c-Brd>/BrdCrCrl, C57BL/6J, C57BL/6JEiJ, C57BL/6JJicTac, C57BL/6JRj	0	1	1	100.0%
B6N-Tyr<c-Brd>/BrdCrCrl, C57BL/6J, C57BL/6JJicTac, C57BL/6JRj	0	1	5	20.0%
B6N-Tyr<c-Brd>/BrdCrCrl, C57BL/6NCrl, C57BL/6NHsd, C57BL/6NJ, C57BL/6NRj, C57BL/6NTac	0	1	2	50.0%
C57BL/6NHsd, C57BL/6NJ, C57BL/6NRj, C57BL/6NTac	0	1	1	100.0%
C57BL/6NRj	0	1	10	10.0%

Minimal Strain Sets Explaining All Diagnostic Classes (Number of Markers Explained):

- Solution 1: 129S5/SvEvBrd and C57BL/6J and C57BL/6NRj
    - C57BL/6J: 80 / 162 (49.4%)
    - C57BL/6NRj: 16 / 40 (40.0%)
    - 129S5/SvEvBrd: 1 / 5 (20.0%)
- Solution 2: 129S5/SvEvBrd and C57BL/6JRj and C57BL/6NRj
    - C57BL/6JRj: 80 / 162 (49.4%)
    - C57BL/6NRj: 16 / 40 (40.0%)
    - 129S5/SvEvBrd: 1 / 5 (20.0%)

Chromosome	Start (Mb)	Stop (Mb)	Background	Zygosity
1	3000000	53457225	CBA/J	Homozygous
1	53457225	77401276	C57BL/6J and C57BL/6NTac and CBA/J	Heterozygous
1	77401276	153548642	CBA/J	Homozygous
1	153548642	168019536	C57BL/6J and C57BL/6NTac and CBA/J	Heterozygous
1	168019536	195471971	C57BL/6J and C57BL/6NTac	N/A
2	3000000	104448986	C57BL/6J and C57BL/6NTac	N/A
2	104448986	115970567	C57BL/6J and C57BL/6NTac and CBA/J	Heterozygous
2	115970567	139631657	C57BL/6J and C57BL/6NTac	N/A
2	139631657	151062687	C57BL/6J and C57BL/6NTac and CBA/J	Heterozygous
2	151062687	161221795	C57BL/6J and C57BL/6NTac	N/A

MiniMUGA Background Analysis v2.3.1

**Diplotype Intervals**	2	161221795	170694096	C57BL/6J and C57BL/6NTac and CBA/J	Heterozygous
	2	170694096	175780822	CBA/J	Homozygous
	2	175780822	182113224	C57BL/6J and C57BL/6NTac and CBA/J	Heterozygous
	3	3000000	37371933	C57BL/6J and C57BL/6NTac and CBA/J	Heterozygous
	3	37371933	139297311	C57BL/6J and C57BL/6NTac	N/A
	3	139297311	156090101	C57BL/6J and C57BL/6NTac and CBA/J	Heterozygous
	3	156090101	160039680	CBA/J	Homozygous
	4	3000000	26280383	C57BL/6J and C57BL/6NTac	N/A
	4	26280383	29346519	C57BL/6J and C57BL/6NTac and CBA/J	Heterozygous
	4	29346519	30650814	Unexplained	Heterozygous
	4	30650814	32327128	C57BL/6J and C57BL/6NTac and CBA/J	Heterozygous
	4	32327128	35563307	C57BL/6J and C57BL/6NTac	N/A
	4	35563307	37995481	Unexplained	Heterozygous
	4	37995481	41348396	Unexplained	Homozygous
	4	41348396	43372387	C57BL/6J and C57BL/6NTac and CBA/J	Heterozygous
	4	43372387	43819249	Unexplained	Heterozygous
	4	43819249	49280860	C57BL/6J and C57BL/6NTac and CBA/J	Heterozygous
	4	49280860	54114833	C57BL/6J and C57BL/6NTac	N/A
	4	54114833	130336992	C57BL/6J and C57BL/6NTac and CBA/J	Heterozygous
	4	130336992	153356388	CBA/J	Homozygous
	4	153356388	156508116	C57BL/6J and C57BL/6NTac	N/A
	5	3000000	41755530	C57BL/6J and C57BL/6NTac and CBA/J	Heterozygous
	5	41755530	76250760	C57BL/6J and C57BL/6NTac	N/A
	5	76250760	101581477	C57BL/6J and C57BL/6NTac and CBA/J	Heterozygous
	5	101581477	130280923	CBA/J	Homozygous
	5	130280923	151834684	C57BL/6J and C57BL/6NTac and CBA/J	Heterozygous
	6	3000000	149736546	C57BL/6J and C57BL/6NTac	N/A
	7	3000000	72944748	C57BL/6J and C57BL/6NTac and CBA/J	Heterozygous
	7	72944748	145441459	C57BL/6J and C57BL/6NTac	N/A
	8	3000000	129401213	C57BL/6J and C57BL/6NTac and CBA/J	Heterozygous
	9	3000000	124595110	C57BL/6J and C57BL/6NTac and CBA/J	Heterozygous
	10	3000000	42917049	C57BL/6J and C57BL/6NTac and CBA/J	Heterozygous

10	42917049	100561092	CBA/J	Homozygous
10	100561092	130694993	C57BL/6J and C57BL/6NTac and CBA/J	Heterozygous
11	3000000	22302070	CBA/J	Homozygous
11	22302070	49269299	C57BL/6J and C57BL/6NTac and CBA/J	Heterozygous
11	49269299	79617327	CBA/J	Homozygous
11	79617327	122082543	C57BL/6J and C57BL/6NTac and CBA/J	Heterozygous
12	3000000	58307190	C57BL/6J and C57BL/6NTac and CBA/J	Heterozygous
12	58307190	120129022	CBA/J	Homozygous
13	3000000	68886272	C57BL/6J and C57BL/6NTac and CBA/J	Heterozygous
13	68886272	120421639	CBA/J	Homozygous
14	3000000	38092288	C57BL/6J and C57BL/6NTac and CBA/J	Heterozygous
14	38092288	57544602	CBA/J	Homozygous
14	57544602	103377147	C57BL/6J and C57BL/6NTac and CBA/J	Heterozygous
14	103377147	124902244	C57BL/6J and C57BL/6NTac	N/A
15	3000000	36473640	C57BL/6J and C57BL/6NTac and CBA/J	Heterozygous
15	36473640	44010563	C57BL/6J and C57BL/6NTac	N/A
15	44010563	55016741	C57BL/6J and C57BL/6NTac and CBA/J	Heterozygous
15	55016741	63227188	C57BL/6J and C57BL/6NTac	N/A
15	63227188	70554147	C57BL/6J and C57BL/6NTac and CBA/J	Heterozygous
15	70554147	74996398	CBA/J	Homozygous
15	74996398	100173036	C57BL/6J and C57BL/6NTac and CBA/J	Heterozygous
15	100173036	104043685	C57BL/6J and C57BL/6NTac	N/A
16	3000000	20813513	C57BL/6J and C57BL/6NTac and CBA/J	Heterozygous
16	20813513	29701002	CBA/J	Homozygous
16	29701002	73280590	C57BL/6J and C57BL/6NTac and CBA/J	Heterozygous
16	73280590	87403166	CBA/J	Homozygous
16	87403166	98207768	C57BL/6J and C57BL/6NTac and CBA/J	Heterozygous
17	3000000	20616647	C57BL/6J and C57BL/6NTac	N/A
17	20616647	49885651	C57BL/6J and C57BL/6NTac and CBA/J	Heterozygous
17	49885651	94987271	C57BL/6J and C57BL/6NTac	N/A
18	3000000	12406382	C57BL/6J and C57BL/6NTac	N/A
18	12406382	27650151	C57BL/6J and C57BL/6NTac and CBA/J	Heterozygous

MiniMUGA Background Analysis v2.3.1

	18	27650151	90702639	CBA/J	Homozygous
	19	3000000	31636352	C57BL/6J and C57BL/6NTac	N/A
	19	31636352	61431566	C57BL/6J and C57BL/6NTac and CBA/J	Heterozygous
	X	3000000	105020820	C57BL/6J and C57BL/6NTac	N/A
	X	105020820	136441962	C57BL/6J and C57BL/6NTac and CBA/J	Heterozygous
	X	136441962	171031299	C57BL/6J and C57BL/6NTac	N/A
	MT	0	0	IBD	Hemizygous
