# Supplementary material for: Deletion of 9p drives B-ALL through heterozygous inactivation of Pax5 and Cd72 in preleukemic cells
Source: JCI Insight. 2026 Feb 17;11(7):e199464. doi: 10.1172/jci.insight.199464 (PMC13134721; doi:10.1172/jci.insight.199464)
Supplement: Supplemental data set 1 [file jciinsight-11-199464-s204.zip › Strain_Genotyping/Q531-results-report.pdf]

MiniMUGA Background Analysis v2.3.1

<table>
<tr><th>Sample ID</th><td>Q531</td></tr>
<tr><th>Neogen ID</th><td>AAAU-4490</td></tr>
<tr><th>Summary</th><td>
The genotype of this sample is of excellent quality. It is female and outbred, and likely a mix of C57BL/6J and C57BL/6NTac and CBA/J. Clustering of unexplained markers is evidence of an additional background strain.

Diagnostic SNPs are likely explained by the presence of the background strains

Solution 1: C57BL/6J and C57BL/6NRj
C57BL/6J: 61 / 162 (37.7%)
C57BL/6NRj: 16 / 39 (41.0%)
Solution 2: C57BL/6JRj and C57BL/6NRj
C57BL/6JRj: 61 / 162 (37.7%)
C57BL/6NRj: 16 / 39 (41.0%)

NOTE: There is a discrepancy between the diagnostic backgrounds detected and the primary and secondary background analysis (CBA/J, C57BL/6J, C57BL/6NTac). This is uncommon and should be investigated further.

No genetic constructs were detected in this sample.

WARNING:
There is a discrepancy between the diagnostic backgrounds detected ((C57BL/6J and C57BL/6NRj) or (C57BL/6JRj and C57BL/6NRj)) and the primary background (C57BL/6J and C57BL/6NTac) and secondary background (CBA/J). This is uncommon and should be investigated further.
</td></tr>
<tr><th>Genotyping Quality</th><td>Excellent (11 N calls)
All reported results are dependent on genotyping quality.</td></tr>
<tr><th>Chromosomal Sex</th><td>XX</td></tr>
<tr><th>Inbreeding Estimate</th><td>69.2% Inbred
(Percentage of the genome (autosomal and X chromosomes) that is homozygous or hemizygous for primary, secondary, and unknown backgrounds. See Genome Analysis)</td></tr>
<tr><th>Constructs Detected</th><td>
<table>
<tr><th>BlastR</th><th>bpA</th><th>Cas9</th><th>chlor</th><th>cHS4</th><th>Cre</th><th>DTA</th><th>Flp</th><th>g_FP</th><th>hCMV_a</th><th>hCMV_b</th><th>hTK_pr</th><th>iCre</th><th>IRES</th><th>Luc</th><th>r_FP</th><th>rtTA</th><th>SV40</th><th>tTA</th></tr>
<tr><td>-</td><td>-</td><td>-</td><td>-</td><td>-</td><td>-</td><td>-</td><td>-</td><td>-</td><td>-</td><td>-</td><td>-</td><td>-</td><td>-</td><td>-</td><td>-</td><td>-</td><td>-</td><td>-</td></tr>
</table>
</td></tr>
</table>

MiniMUGA Background Analysis v2.3.1

Refined Ideogram

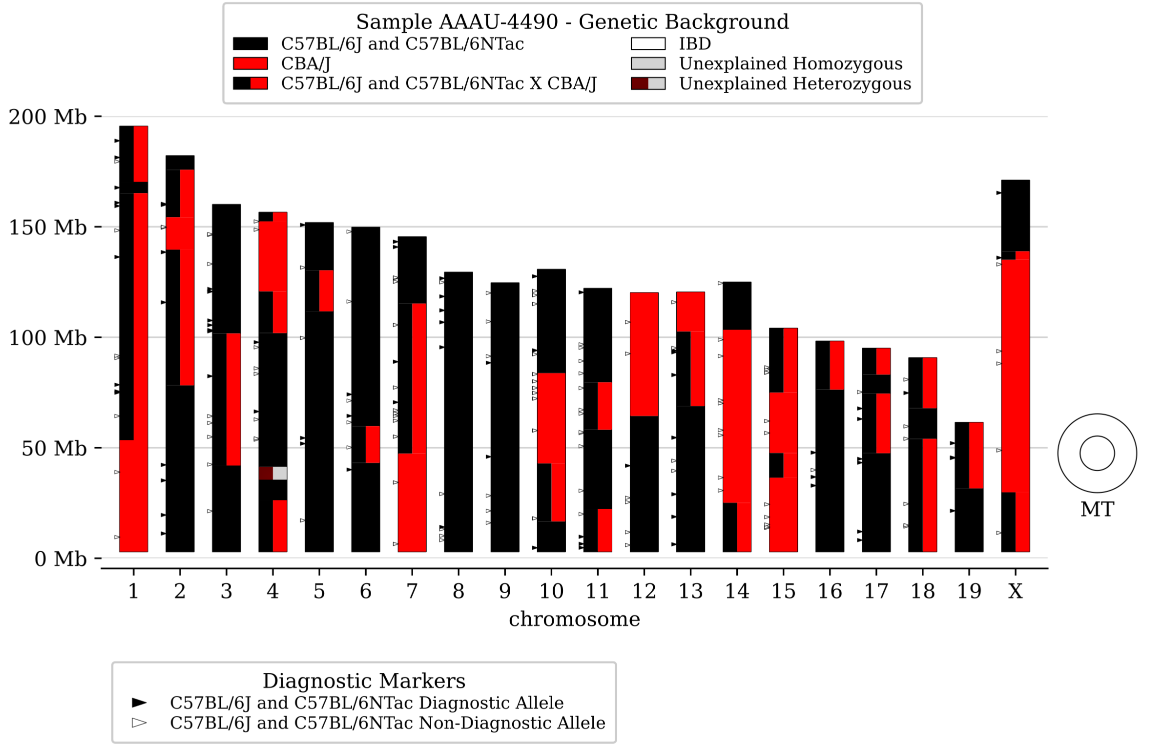

Genome Analysis

Background	Zygosity	Informative Markers	Informative Markers %	Genome %
C57BL/6J and C57BL/6NTac	N/A	1222	46.0%	49.8%
CBA/J	Homozygous	538	20.3%	19.4%
C57BL/6J and C57BL/6NTac X CBA/J	Heterozygous	884	33.3%	30.6%
Unexplained	Heterozygous	11	0.4%	0.2%
	**Total**	2655	100.0%	100.0%

Y Chromosome

Not Applicable

MT Genome

MT Haplogroup 6 - 100.0% Consistent
Includes C57BL/6J, C57BL/6NTac, CBA/J and 165 other strains

MiniMUGA Background Analysis v2.3.1

Backgrounds Detected (Diagnostic Alleles)

Diagnostic Class	Diagnostic Alleles Observed: Homozygous	Diagnostic Alleles Observed: Heterozygous	Potential	% Observed
C57BL/6J, C57BL/6JJicTac, C57BL/6JRj	5	35	102	39.2%
C57BL/6J, C57BL/6JEiJ, C57BL/6JJicTac, C57BL/6JRj	6	4	21	47.6%
C57BL/6NRj, C57BL/6NTac	3	5	15	53.3%
C57BL/6J, C57BL/6JRj	0	7	31	22.6%
C57BL/6NJ, C57BL/6NRj, C57BL/6NTac	3	1	10	40.0%
B6N-Tyr<c-Brd>/BrdCrCrl, C57BL/6J, C57BL/6JEiJ, C57BL/6JJicTac, C57BL/6JRj	1	0	1	100.0%
B6N-Tyr<c-Brd>/BrdCrCrl, C57BL/6J, C57BL/6JJicTac, C57BL/6JRj	0	2	5	40.0%
B6N-Tyr<c-Brd>/BrdCrCrl, C57BL/6NCrl, C57BL/6NHsd, C57BL/6NJ, C57BL/6NRj, C57BL/6NTac	1	0	2	50.0%
C57BL/6NCrl, C57BL/6NHsd, C57BL/6NJ, C57BL/6NRj, C57BL/6NTac	0	2	2	100.0%
C57BL/6J, C57BL/6JBomTac, C57BL/6JEiJ, C57BL/6JJicTac, C57BL/6JOlaHsd, C57BL/6JRj	0	1	2	50.0%
C57BL/6NRj	0	1	10	10.0%

Minimal Strain Sets Explaining All Diagnostic Classes (Number of Markers Explained):

- Solution 1: C57BL/6J and C57BL/6NRj
  - C57BL/6J: 61 / 162 (37.7%)
  - C57BL/6NRj: 16 / 39 (41.0%)
- Solution 2: C57BL/6JRj and C57BL/6NRj
  - C57BL/6JRj: 61 / 162 (37.7%)
  - C57BL/6NRj: 16 / 39 (41.0%)

Chromosome	Start (Mb)	Stop (Mb)	Background	Zygosity
1	3000000	53457225	CBA/J	Homozygous
1	53457225	165183608	C57BL/6J and C57BL/6NTac and CBA/J	Heterozygous
1	165183608	170316822	C57BL/6J and C57BL/6NTac	N/A
1	170316822	195471971	C57BL/6J and C57BL/6NTac and CBA/J	Heterozygous
2	3000000	78267191	C57BL/6J and C57BL/6NTac	N/A
2	78267191	139631657	C57BL/6J and C57BL/6NTac and CBA/J	Heterozygous
2	139631657	154349372	CBA/J	Homozygous
2	154349372	175780822	C57BL/6J and C57BL/6NTac and CBA/J	Heterozygous
2	175780822	182113224	C57BL/6J and C57BL/6NTac	N/A
3	3000000	41975127	C57BL/6J and C57BL/6NTac	N/A
3	41975127	101716043	C57BL/6J and C57BL/6NTac and CBA/J	Heterozygous
3	101716043	160039680	C57BL/6J and C57BL/6NTac	N/A
4	3000000	26280383	C57BL/6J and C57BL/6NTac and CBA/J	Heterozygous

MiniMUGA Background Analysis v2.3.1

**Diplotype Intervals**	4	26280383	35563307	C57BL/6J and C57BL/6NTac	N/A
	4	35563307	41348396	Unexplained	Heterozygous
	4	41348396	101914190	C57BL/6J and C57BL/6NTac	N/A
	4	101914190	120738488	C57BL/6J and C57BL/6NTac and CBA/J	Heterozygous
	4	120738488	152440879	CBA/J	Homozygous
	4	152440879	156508116	C57BL/6J and C57BL/6NTac and CBA/J	Heterozygous
	5	3000000	111745102	C57BL/6J and C57BL/6NTac	N/A
	5	111745102	130280923	C57BL/6J and C57BL/6NTac and CBA/J	Heterozygous
	5	130280923	151834684	C57BL/6J and C57BL/6NTac	N/A
	6	3000000	43184432	C57BL/6J and C57BL/6NTac	N/A
	6	43184432	59791688	C57BL/6J and C57BL/6NTac and CBA/J	Heterozygous
	6	59791688	149736546	C57BL/6J and C57BL/6NTac	N/A
	7	3000000	47395440	CBA/J	Homozygous
	7	47395440	115227247	C57BL/6J and C57BL/6NTac and CBA/J	Heterozygous
	7	115227247	145441459	C57BL/6J and C57BL/6NTac	N/A
	8	3000000	129401213	C57BL/6J and C57BL/6NTac	N/A
	9	3000000	124595110	C57BL/6J and C57BL/6NTac	N/A
	10	3000000	16704298	C57BL/6J and C57BL/6NTac	N/A
	10	16704298	42917049	C57BL/6J and C57BL/6NTac and CBA/J	Heterozygous
	10	42917049	83779430	CBA/J	Homozygous
	10	83779430	130694993	C57BL/6J and C57BL/6NTac	N/A
	11	3000000	22302070	C57BL/6J and C57BL/6NTac and CBA/J	Heterozygous
	11	22302070	58168384	C57BL/6J and C57BL/6NTac	N/A
	11	58168384	79617327	C57BL/6J and C57BL/6NTac and CBA/J	Heterozygous
	11	79617327	122082543	C57BL/6J and C57BL/6NTac	N/A
	12	3000000	64411355	C57BL/6J and C57BL/6NTac	N/A
	12	64411355	120129022	CBA/J	Homozygous
	13	3000000	68886272	C57BL/6J and C57BL/6NTac	N/A
	13	68886272	102595519	C57BL/6J and C57BL/6NTac and CBA/J	Heterozygous
	13	102595519	120421639	CBA/J	Homozygous
	14	3000000	25112834	C57BL/6J and C57BL/6NTac and CBA/J	Heterozygous

MiniMUGA Background Analysis v2.3.1

	14	25112834	103377147	CBA/J	Homozygous
	14	103377147	124902244	C57BL/6J and C57BL/6NTac	N/A
	15	3000000	36473640	CBA/J	Homozygous
	15	36473640	47626553	C57BL/6J and C57BL/6NTac and CBA/J	Heterozygous
	15	47626553	74996398	CBA/J	Homozygous
	15	74996398	104043685	C57BL/6J and C57BL/6NTac and CBA/J	Heterozygous
	16	3000000	76315797	C57BL/6J and C57BL/6NTac	N/A
	16	76315797	98207768	C57BL/6J and C57BL/6NTac and CBA/J	Heterozygous
	17	3000000	47545390	C57BL/6J and C57BL/6NTac	N/A
	17	47545390	74502727	C57BL/6J and C57BL/6NTac and CBA/J	Heterozygous
	17	74502727	83146268	C57BL/6J and C57BL/6NTac	N/A
	17	83146268	94987271	C57BL/6J and C57BL/6NTac and CBA/J	Heterozygous
	18	3000000	54023745	C57BL/6J and C57BL/6NTac and CBA/J	Heterozygous
	18	54023745	67937187	C57BL/6J and C57BL/6NTac	N/A
	18	67937187	90702639	C57BL/6J and C57BL/6NTac and CBA/J	Heterozygous
	19	3000000	31636352	C57BL/6J and C57BL/6NTac	N/A
	19	31636352	61431566	C57BL/6J and C57BL/6NTac and CBA/J	Heterozygous
	X	3000000	29836043	C57BL/6J and C57BL/6NTac and CBA/J	Heterozygous
	X	29836043	135099309	CBA/J	Homozygous
	X	135099309	138881041	C57BL/6J and C57BL/6NTac and CBA/J	Heterozygous
	X	138881041	171031299	C57BL/6J and C57BL/6NTac	N/A
	MT	0	0	IBD	Hemizygous
