# Supplementary material for: Deletion of 9p drives B-ALL through heterozygous inactivation of Pax5 and Cd72 in preleukemic cells
Source: JCI Insight. 2026 Feb 17;11(7):e199464. doi: 10.1172/jci.insight.199464 (PMC13134721; doi:10.1172/jci.insight.199464)
Supplement: Supplemental data set 1 [file jciinsight-11-199464-s204.zip › Strain_Genotyping/Q532-results-report.pdf]

MiniMUGA Background Analysis v2.3.1

**Sample ID**	Q532
**Neogen ID**	AAAU-4530
**Summary**	The genotype of this sample is of **excellent** quality. It is **female** and **outbred**, and likely a mix of **C57BL/6J and C57BL/6NRj** and **CBA/J**. Clustering of unexplained markers is evidence of an additional background strain.<br><br>Diagnostic SNPs are likely explained by the presence of the background strains<br>• Solution 1: 129S5/SvEvBrd and C57BL/6J and C57BL/6NRj<br>◦ C57BL/6J: 49 / 157 (31.2%)<br>◦ C57BL/6NRj: 20 / 40 (50.0%)<br>◦ 129S5/SvEvBrd: 1 / 5 (20.0%)<br>• Solution 2: 129S5/SvEvBrd and C57BL/6JRj and C57BL/6NRj<br>◦ C57BL/6JRj: 49 / 157 (31.2%)<br>◦ C57BL/6NRj: 20 / 40 (50.0%)<br>◦ 129S5/SvEvBrd: 1 / 5 (20.0%)<br><br>NOTE: There is a discrepancy between the diagnostic backgrounds detected and the primary and secondary background analysis (C57BL/6NRj, CBA/J, C57BL/6J). This is uncommon and should be investigated further.<br><br>No genetic constructs were detected in this sample.<br><br>WARNING:<br>• There is a discrepancy between the diagnostic backgrounds detected ((129S5/SvEvBrd and C57BL/6J and C57BL/6NRj) or (129S5/SvEvBrd and C57BL/6JRj and C57BL/6NRj)) and the primary background (C57BL/6J and C57BL/6NRj) and secondary background (CBA/J). This is uncommon and should be investigated further.<br>• The presence of a single diagnostic heterozygous call for a single inbred strain should be treated with caution.<br>• This sample likely has more than 2 genetic backgrounds (unexplained regions and/or fractured ideogram). The strain selected for secondary background may be incorrect. The estimation of the contribution of primary and secondary background are likely incorrect. This can potentially be addressed with input from the user.
**Genotyping Quality**	**Excellent (12 N calls)**<br>All reported results are dependent on genotyping quality.
**Chromosomal Sex**	XX
**Inbreeding Estimate**	66.4% Inbred<br>(Percentage of the genome (autosomal and X chromosomes) that is homozygous or hemizygous for primary, secondary, and unknown backgrounds. See Genome Analysis)

Constructs Detected

BlastR	bpA	Cas9	chlor	cHS4	Cre	DTA	Flp	g_FP	hCMV_a	hCMV_b	hTK_pr	iCre	IRES	Luc	r_FP	rtTA	SV40	tTA
-	-	-	-	-	-	-	-	-	-	-	-	-	-	-	-	-	-	-

MiniMUGA Background Analysis v2.3.1

Refined Ideogram

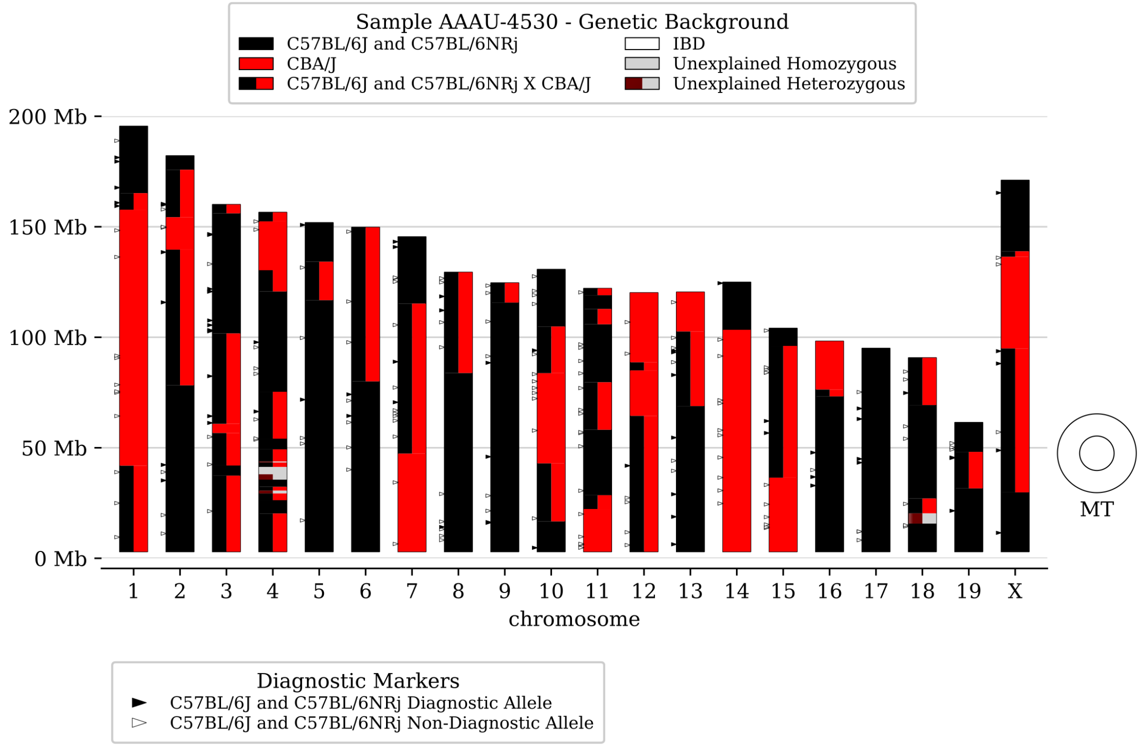

Genome Analysis

Background	Zygosity	Informative Markers	Informative Markers %	Genome %
C57BL/6J and C57BL/6NRj	N/A	1193	44.8%	45.8%
CBA/J	Homozygous	556	20.9%	20.5%
C57BL/6J and C57BL/6NRj X CBA/J	Heterozygous	893	33.5%	33.2%
Unexplained	Homozygous	5	0.2%	0.1%
Unexplained	Heterozygous	15	0.6%	0.3%
	**Total**	2662	100.0%	99.9%

Y Chromosome

Not Applicable

MT Genome

MT Haplogroup 6 - 100.0% Consistent
Includes C57BL/6J, C57BL/6NRj, CBA/J and 165 other strains

MiniMUGA Background Analysis v2.3.1

Backgrounds Detected (Diagnostic Alleles)

Diagnostic Class	Diagnostic Alleles Observed: Homozygous	Heterozygous	Potential	% Observed
C57BL/6J, C57BL/6JJicTac, C57BL/6JRj	3	30	102	32.4%
C57BL/6J, C57BL/6JEiJ, C57BL/6JJicTac, C57BL/6JRj	5	3	21	38.1%
C57BL/6NRj, C57BL/6NTac	2	8	15	66.7%
C57BL/6NJ, C57BL/6NRj, C57BL/6NTac	3	1	10	40.0%
C57BL/6J, C57BL/6JRj	0	6	31	19.4%
B6N-Tyr<c-Brd>/BrdCrCrl, C57BL/6NCrl, C57BL/6NHsd, C57BL/6NJ, C57BL/6NRj, C57BL/6NTac	1	0	2	50.0%
C57BL/6NCrl, C57BL/6NHsd, C57BL/6NJ, C57BL/6NRj, C57BL/6NTac	0	2	2	100.0%
C57BL/6NRj	0	2	10	20.0%
129S5/SvEvBrd	0	1	5	20.0%
B6N-Tyr<c-Brd>/BrdCrCrl, C57BL/6J, C57BL/6JEiJ, C57BL/6JJicTac, C57BL/6JRj	0	1	1	100.0%
C57BL/6J, C57BL/6JBomTac, C57BL/6JEiJ, C57BL/6JJicTac, C57BL/6JOlaHsd, C57BL/6JRj	0	1	2	50.0%
C57BL/6NHsd, C57BL/6NJ, C57BL/6NRj, C57BL/6NTac	0	1	1	100.0%

Minimal Strain Sets Explaining All Diagnostic Classes (Number of Markers Explained):

- Solution 1: 129S5/SvEvBrd and C57BL/6J and C57BL/6NRj
  - C57BL/6J: 49 / 157 (31.2%)
  - C57BL/6NRj: 20 / 40 (50.0%)
  - 129S5/SvEvBrd: 1 / 5 (20.0%)
- Solution 2: 129S5/SvEvBrd and C57BL/6JRj and C57BL/6NRj
  - C57BL/6JRj: 49 / 157 (31.2%)
  - C57BL/6NRj: 20 / 40 (50.0%)
  - 129S5/SvEvBrd: 1 / 5 (20.0%)

Chromosome	Start (Mb)	Stop (Mb)	Background	Zygosity
1	3000000	41869819	C57BL/6J and C57BL/6NRj and CBA/J	Heterozygous
1	41869819	157713559	CBA/J	Homozygous
1	157713559	165183608	C57BL/6J and C57BL/6NRj and CBA/J	Heterozygous
1	165183608	195471971	C57BL/6J and C57BL/6NRj	N/A
2	3000000	78267191	C57BL/6J and C57BL/6NRj	N/A
2	78267191	139631657	C57BL/6J and C57BL/6NRj and CBA/J	Heterozygous
2	139631657	154349372	CBA/J	Homozygous
2	154349372	175780822	C57BL/6J and C57BL/6NRj and CBA/J	Heterozygous
2	175780822	182113224	C57BL/6J and C57BL/6NRj	N/A
3	3000000	37371933	C57BL/6J and C57BL/6NRj and CBA/J	Heterozygous
3	37371933	41975127	C57BL/6J and C57BL/6NRj	N/A

**Diplotype Intervals**	3	41975127	56655047	C57BL/6J and C57BL/6NRj and CBA/J	Heterozygous
	3	56655047	60850190	CBA/J	Homozygous
	3	60850190	101716043	C57BL/6J and C57BL/6NRj and CBA/J	Heterozygous
	3	101716043	156090101	C57BL/6J and C57BL/6NRj	N/A
	3	156090101	160039680	C57BL/6J and C57BL/6NRj and CBA/J	Heterozygous
	4	3000000	20258658	C57BL/6J and C57BL/6NRj and CBA/J	Heterozygous
	4	20258658	26280383	C57BL/6J and C57BL/6NRj	N/A
	4	26280383	29346519	C57BL/6J and C57BL/6NRj and CBA/J	Heterozygous
	4	29346519	30650814	Unexplained	Heterozygous
	4	30650814	32327128	C57BL/6J and C57BL/6NRj and CBA/J	Heterozygous
	4	32327128	35563307	C57BL/6J and C57BL/6NRj	N/A
	4	35563307	37995481	Unexplained	Heterozygous
	4	37995481	41348396	Unexplained	Homozygous
	4	41348396	43372387	C57BL/6J and C57BL/6NRj and CBA/J	Heterozygous
	4	43372387	43819249	Unexplained	Heterozygous
	4	43819249	49280860	C57BL/6J and C57BL/6NRj and CBA/J	Heterozygous
	4	49280860	54114833	C57BL/6J and C57BL/6NRj	N/A
	4	54114833	75318594	C57BL/6J and C57BL/6NRj and CBA/J	Heterozygous
	4	75318594	120738488	C57BL/6J and C57BL/6NRj	N/A
	4	120738488	130336992	C57BL/6J and C57BL/6NRj and CBA/J	Heterozygous
	4	130336992	152440879	CBA/J	Homozygous
	4	152440879	156508116	C57BL/6J and C57BL/6NRj and CBA/J	Heterozygous
	5	3000000	116795433	C57BL/6J and C57BL/6NRj	N/A
	5	116795433	134172373	C57BL/6J and C57BL/6NRj and CBA/J	Heterozygous
	5	134172373	151834684	C57BL/6J and C57BL/6NRj	N/A
	6	3000000	80057017	C57BL/6J and C57BL/6NRj	N/A
	6	80057017	149736546	C57BL/6J and C57BL/6NRj and CBA/J	Heterozygous
	7	3000000	47395440	CBA/J	Homozygous
	7	47395440	115227247	C57BL/6J and C57BL/6NRj and CBA/J	Heterozygous
	7	115227247	145441459	C57BL/6J and C57BL/6NRj	N/A
	8	3000000	83842204	C57BL/6J and C57BL/6NRj	N/A

MiniMUGA Background Analysis v2.3.1

	8	83842204	129401213	C57BL/6J and C57BL/6NRj and CBA/J	Heterozygous
	9	3000000	115715944	C57BL/6J and C57BL/6NRj	N/A
	9	115715944	124595110	C57BL/6J and C57BL/6NRj and CBA/J	Heterozygous
	10	3000000	16704298	C57BL/6J and C57BL/6NRj	N/A
	10	16704298	42917049	C57BL/6J and C57BL/6NRj and CBA/J	Heterozygous
	10	42917049	83779430	CBA/J	Homozygous
	10	83779430	104861956	C57BL/6J and C57BL/6NRj and CBA/J	Heterozygous
	10	104861956	130694993	C57BL/6J and C57BL/6NRj	N/A
	11	3000000	22302070	CBA/J	Homozygous
	11	22302070	28525615	C57BL/6J and C57BL/6NRj and CBA/J	Heterozygous
	11	28525615	58168384	C57BL/6J and C57BL/6NRj	N/A
	11	58168384	79617327	C57BL/6J and C57BL/6NRj and CBA/J	Heterozygous
	11	79617327	105886229	C57BL/6J and C57BL/6NRj	N/A
	11	105886229	112771442	C57BL/6J and C57BL/6NRj and CBA/J	Heterozygous
	11	112771442	119038285	C57BL/6J and C57BL/6NRj	N/A
	11	119038285	122082543	C57BL/6J and C57BL/6NRj and CBA/J	Heterozygous
	12	3000000	64411355	C57BL/6J and C57BL/6NRj and CBA/J	Heterozygous
	12	64411355	85015902	CBA/J	Homozygous
	12	85015902	88650858	C57BL/6J and C57BL/6NRj and CBA/J	Heterozygous
	12	88650858	120129022	CBA/J	Homozygous
	13	3000000	68886272	C57BL/6J and C57BL/6NRj	N/A
	13	68886272	102595519	C57BL/6J and C57BL/6NRj and CBA/J	Heterozygous
	13	102595519	120421639	CBA/J	Homozygous
	14	3000000	103377147	CBA/J	Homozygous
	14	103377147	124902244	C57BL/6J and C57BL/6NRj	N/A
	15	3000000	36473640	CBA/J	Homozygous
	15	36473640	96089091	C57BL/6J and C57BL/6NRj and CBA/J	Heterozygous
	15	96089091	104043685	C57BL/6J and C57BL/6NRj	N/A
	16	3000000	73280590	C57BL/6J and C57BL/6NRj	N/A
	16	73280590	76315797	C57BL/6J and C57BL/6NRj and CBA/J	Heterozygous
	16	76315797	98207768	CBA/J	Homozygous
	17	3000000	94987271	C57BL/6J and C57BL/6NRj	N/A

MiniMUGA Background Analysis v2.3.1

	18	3000000	15685654	C57BL/6J and C57BL/6NRj	N/A
	18	15685654	20363699	Unexplained	Heterozygous
	18	20363699	27036500	C57BL/6J and C57BL/6NRj and CBA/J	Heterozygous
	18	27036500	69337106	C57BL/6J and C57BL/6NRj	N/A
	18	69337106	90702639	C57BL/6J and C57BL/6NRj and CBA/J	Heterozygous
	19	3000000	31636352	C57BL/6J and C57BL/6NRj	N/A
	19	31636352	48128956	C57BL/6J and C57BL/6NRj and CBA/J	Heterozygous
	19	48128956	61431566	C57BL/6J and C57BL/6NRj	N/A
	X	3000000	29836043	C57BL/6J and C57BL/6NRj	N/A
	X	29836043	94918419	C57BL/6J and C57BL/6NRj and CBA/J	Heterozygous
	X	94918419	136441962	CBA/J	Homozygous
	X	136441962	138881041	C57BL/6J and C57BL/6NRj and CBA/J	Heterozygous
	X	138881041	171031299	C57BL/6J and C57BL/6NRj	N/A
	MT	0	0	IBD	Hemizygous
