# Supplementary material for: Deletion of 9p drives B-ALL through heterozygous inactivation of Pax5 and Cd72 in preleukemic cells
Source: JCI Insight. 2026 Feb 17;11(7):e199464. doi: 10.1172/jci.insight.199464 (PMC13134721; doi:10.1172/jci.insight.199464)
Supplement: Supplemental data set 1 [file jciinsight-11-199464-s204.zip › Strain_Genotyping/Q533-results-report.pdf]

MiniMUGA Background Analysis v2.3.1

**Sample ID**	Q533
**Neogen ID**	AAAU-4531
**Summary**	The genotype of this sample is of **excellent** quality. It is **female** and **outbred**, and likely a mix of **C57BL/6J and C57BL/6NTac** and **CBA/J**. Clustering of unexplained markers is evidence of an additional background strain.<br><br>Diagnostic SNPs are likely explained by the presence of the background strains<br>• Solution 1: 129S5/SvEvBrd and C57BL/6J and C57BL/6NRj<br>◦ C57BL/6J: 49 / 157 (31.2%)<br>◦ C57BL/6NRj: 19 / 40 (47.5%)<br>◦ 129S5/SvEvBrd: 1 / 5 (20.0%)<br>• Solution 2: 129S5/SvEvBrd and C57BL/6JRj and C57BL/6NRj<br>◦ C57BL/6JRj: 49 / 157 (31.2%)<br>◦ C57BL/6NRj: 19 / 40 (47.5%)<br>◦ 129S5/SvEvBrd: 1 / 5 (20.0%)<br><br>NOTE: There is a discrepancy between the diagnostic backgrounds detected and the primary and secondary background analysis (CBA/J, C57BL/6J, C57BL/6NTac). This is uncommon and should be investigated further.<br><br>No genetic constructs were detected in this sample.<br><br>WARNING:<br>• There is a discrepancy between the diagnostic backgrounds detected ((129S5/SvEvBrd and C57BL/6J and C57BL/6NRj) or (129S5/SvEvBrd and C57BL/6JRj and C57BL/6NRj)) and the primary background (C57BL/6J and C57BL/6NTac) and secondary background (CBA/J). This is uncommon and should be investigated further.<br>• The presence of a single diagnostic heterozygous call for a single inbred strain should be treated with caution.<br>• This sample likely has more than 2 genetic backgrounds (unexplained regions and/or fractured ideogram). The strain selected for secondary background may be incorrect. The estimation of the contribution of primary and secondary background are likely incorrect. This can potentially be addressed with input from the user.
**Genotyping Quality**	**Excellent (8 N calls)**<br>All reported results are dependent on genotyping quality.
**Chromosomal Sex**	XX
**Inbreeding Estimate**	60.3% Inbred<br>(Percentage of the genome (autosomal and X chromosomes) that is homozygous or hemizygous for primary, secondary, and unknown backgrounds. See Genome Analysis)
**Constructs Detected**	(see below)

Constructs Detected

BlastR	bpA	Cas9	chlor	cHS4	Cre	DTA	Flp	g_FP	hCMV_a	hCMV_b	hTK_pr	iCre	IRES	Luc	r_FP	rtTA	SV40	tTA
-	-	-	-	-	-	-	-	-	-	-	-	-	-	-	-	-	-	-

MiniMUGA Background Analysis v2.3.1

Refined Ideogram

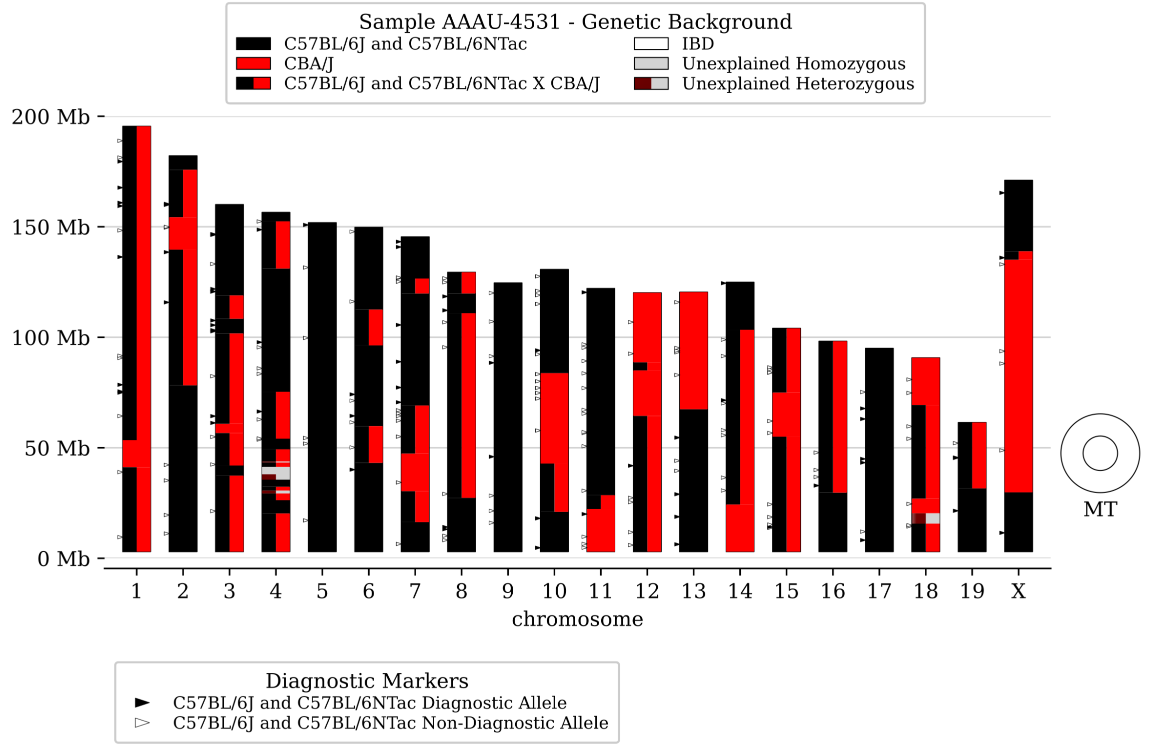

Genome Analysis

Background	Zygosity	Informative Markers	Informative Markers %	Genome %
C57BL/6J and C57BL/6NTac	N/A	1116	41.9%	45.0%
CBA/J	Homozygous	408	15.3%	15.1%
C57BL/6J and C57BL/6NTac X CBA/J	Heterozygous	1119	42.0%	39.4%
Unexplained	Homozygous	5	0.2%	0.1%
Unexplained	Heterozygous	16	0.6%	0.3%
	**Total**	2664	100.0%	99.9%

Y Chromosome

Not Applicable

MT Genome

MT Haplogroup 6 - 100.0% Consistent
Includes C57BL/6J, C57BL/6NTac, CBA/J and 165 other strains

MiniMUGA Background Analysis v2.3.1

Backgrounds Detected (Diagnostic Alleles)

	Diagnostic Alleles Observed			
**Diagnostic Class**	**Homozygous**	**Heterozygous**	**Potential**	**% Observed**
C57BL/6J, C57BL/6JJicTac, C57BL/6JRj	5	28	102	32.4%
C57BL/6J, C57BL/6JEiJ, C57BL/6JJicTac, C57BL/6JRj	5	3	21	38.1%
C57BL/6NRj, C57BL/6NTac	3	5	15	53.3%
C57BL/6NJ, C57BL/6NRj, C57BL/6NTac	2	4	10	60.0%
C57BL/6J, C57BL/6JRj	0	6	31	19.4%
B6N-Tyr<c-Brd>/BrdCrCrl, C57BL/6NCrl, C57BL/6NHsd, C57BL/6NJ, C57BL/6NRj, C57BL/6NTac	1	0	2	50.0%
C57BL/6NCrl, C57BL/6NHsd, C57BL/6NJ, C57BL/6NRj, C57BL/6NTac	0	2	2	100.0%
129S5/SvEvBrd	0	1	5	20.0%
B6N-Tyr<c-Brd>/BrdCrCrl, C57BL/6J, C57BL/6JEiJ, C57BL/6JJicTac, C57BL/6JRj	0	1	1	100.0%
C57BL/6J, C57BL/6JBomTac, C57BL/6JEiJ, C57BL/6JJicTac, C57BL/6JOlaHsd, C57BL/6JRj	0	1	2	50.0%
C57BL/6NHsd, C57BL/6NJ, C57BL/6NRj, C57BL/6NTac	0	1	1	100.0%
C57BL/6NRj	0	1	10	10.0%

Minimal Strain Sets Explaining All Diagnostic Classes (Number of Markers Explained):

- Solution 1: 129S5/SvEvBrd and C57BL/6J and C57BL/6NRj
    - C57BL/6J: 49 / 157 (31.2%)
    - C57BL/6NRj: 19 / 40 (47.5%)
    - 129S5/SvEvBrd: 1 / 5 (20.0%)
- Solution 2: 129S5/SvEvBrd and C57BL/6JRj and C57BL/6NRj
    - C57BL/6JRj: 49 / 157 (31.2%)
    - C57BL/6NRj: 19 / 40 (47.5%)
    - 129S5/SvEvBrd: 1 / 5 (20.0%)

Chromosome	Start (Mb)	Stop (Mb)	Background	Zygosity
1	3000000	41199760	C57BL/6J and C57BL/6NTac and CBA/J	Heterozygous
1	41199760	53457225	CBA/J	Homozygous
1	53457225	195471971	C57BL/6J and C57BL/6NTac and CBA/J	Heterozygous
2	3000000	78267191	C57BL/6J and C57BL/6NTac	N/A
2	78267191	139631657	C57BL/6J and C57BL/6NTac and CBA/J	Heterozygous
2	139631657	154349372	CBA/J	Homozygous
2	154349372	175780822	C57BL/6J and C57BL/6NTac and CBA/J	Heterozygous
2	175780822	182113224	C57BL/6J and C57BL/6NTac	N/A
3	3000000	37371933	C57BL/6J and C57BL/6NTac and CBA/J	Heterozygous
3	37371933	41975127	C57BL/6J and C57BL/6NTac	N/A
3	41975127	56655047	C57BL/6J and C57BL/6NTac and CBA/J	Heterozygous
3	56655047	60850190	CBA/J	Homozygous

MiniMUGA Background Analysis v2.3.1

**Diplotype Intervals**	3	60850190	101716043	C57BL/6J and C57BL/6NTac and CBA/J	Heterozygous
	3	101716043	108381941	C57BL/6J and C57BL/6NTac	N/A
	3	108381941	118919242	C57BL/6J and C57BL/6NTac and CBA/J	Heterozygous
	3	118919242	160039680	C57BL/6J and C57BL/6NTac	N/A
	4	3000000	20258658	C57BL/6J and C57BL/6NTac and CBA/J	Heterozygous
	4	20258658	26280383	C57BL/6J and C57BL/6NTac	N/A
	4	26280383	29346519	C57BL/6J and C57BL/6NTac and CBA/J	Heterozygous
	4	29346519	30650814	Unexplained	Heterozygous
	4	30650814	32327128	C57BL/6J and C57BL/6NTac and CBA/J	Heterozygous
	4	32327128	35563307	C57BL/6J and C57BL/6NTac	N/A
	4	35563307	37995481	Unexplained	Heterozygous
	4	37995481	41348396	Unexplained	Homozygous
	4	41348396	43372387	C57BL/6J and C57BL/6NTac and CBA/J	Heterozygous
	4	43372387	43819249	Unexplained	Heterozygous
	4	43819249	49280860	C57BL/6J and C57BL/6NTac and CBA/J	Heterozygous
	4	49280860	54114833	C57BL/6J and C57BL/6NTac	N/A
	4	54114833	75318594	C57BL/6J and C57BL/6NTac and CBA/J	Heterozygous
	4	75318594	131104093	C57BL/6J and C57BL/6NTac	N/A
	4	131104093	152440879	C57BL/6J and C57BL/6NTac and CBA/J	Heterozygous
	4	152440879	156508116	C57BL/6J and C57BL/6NTac	N/A
	5	3000000	151834684	C57BL/6J and C57BL/6NTac	N/A
	6	3000000	43184432	C57BL/6J and C57BL/6NTac	N/A
	6	43184432	59791688	C57BL/6J and C57BL/6NTac and CBA/J	Heterozygous
	6	59791688	96327282	C57BL/6J and C57BL/6NTac	N/A
	6	96327282	112515862	C57BL/6J and C57BL/6NTac and CBA/J	Heterozygous
	6	112515862	149736546	C57BL/6J and C57BL/6NTac	N/A
	7	3000000	16360273	C57BL/6J and C57BL/6NTac	N/A
	7	16360273	30335112	C57BL/6J and C57BL/6NTac and CBA/J	Heterozygous
	7	30335112	47395440	CBA/J	Homozygous
	7	47395440	69096424	C57BL/6J and C57BL/6NTac and CBA/J	Heterozygous
	7	69096424	119823617	C57BL/6J and C57BL/6NTac	N/A

MiniMUGA Background Analysis v2.3.1

7	119823617	126580094	C57BL/6J and C57BL/6NTac and CBA/J	Heterozygous
7	126580094	145441459	C57BL/6J and C57BL/6NTac	N/A
8	3000000	27348459	C57BL/6J and C57BL/6NTac	N/A
8	27348459	110881875	C57BL/6J and C57BL/6NTac and CBA/J	Heterozygous
8	110881875	119835722	C57BL/6J and C57BL/6NTac	N/A
8	119835722	129401213	C57BL/6J and C57BL/6NTac and CBA/J	Heterozygous
9	3000000	124595110	C57BL/6J and C57BL/6NTac	N/A
10	3000000	21018821	C57BL/6J and C57BL/6NTac	N/A
10	21018821	42858234	C57BL/6J and C57BL/6NTac and CBA/J	Heterozygous
10	42858234	83779430	CBA/J	Homozygous
10	83779430	130694993	C57BL/6J and C57BL/6NTac	N/A
11	3000000	22302070	CBA/J	Homozygous
11	22302070	28525615	C57BL/6J and C57BL/6NTac and CBA/J	Heterozygous
11	28525615	122082543	C57BL/6J and C57BL/6NTac	N/A
12	3000000	64411355	C57BL/6J and C57BL/6NTac and CBA/J	Heterozygous
12	64411355	85015902	CBA/J	Homozygous
12	85015902	88650858	C57BL/6J and C57BL/6NTac and CBA/J	Heterozygous
12	88650858	120129022	CBA/J	Homozygous
13	3000000	67442927	C57BL/6J and C57BL/6NTac	N/A
13	67442927	120421639	CBA/J	Homozygous
14	3000000	24355636	CBA/J	Homozygous
14	24355636	103377147	C57BL/6J and C57BL/6NTac and CBA/J	Heterozygous
14	103377147	124902244	C57BL/6J and C57BL/6NTac	N/A
15	3000000	55016741	C57BL/6J and C57BL/6NTac and CBA/J	Heterozygous
15	55016741	74996398	CBA/J	Homozygous
15	74996398	104043685	C57BL/6J and C57BL/6NTac and CBA/J	Heterozygous
16	3000000	29701002	C57BL/6J and C57BL/6NTac	N/A
16	29701002	98207768	C57BL/6J and C57BL/6NTac and CBA/J	Heterozygous
17	3000000	94987271	C57BL/6J and C57BL/6NTac	N/A
18	3000000	15685654	C57BL/6J and C57BL/6NTac and CBA/J	Heterozygous
18	15685654	20363699	Unexplained	Heterozygous
18	20363699	27036500	CBA/J	Homozygous

MiniMUGA Background Analysis v2.3.1

	18	27036500	69337106	C57BL/6J and C57BL/6NTac and CBA/J	Heterozygous
	18	69337106	90702639	CBA/J	Homozygous
	19	3000000	31636352	C57BL/6J and C57BL/6NTac	N/A
	19	31636352	61431566	C57BL/6J and C57BL/6NTac and CBA/J	Heterozygous
	X	3000000	29836043	C57BL/6J and C57BL/6NTac	N/A
	X	29836043	135099309	CBA/J	Homozygous
	X	135099309	138881041	C57BL/6J and C57BL/6NTac and CBA/J	Heterozygous
	X	138881041	171031299	C57BL/6J and C57BL/6NTac	N/A
	MT	0	0	IBD	Hemizygous
