# Supplementary material for: Deletion of 9p drives B-ALL through heterozygous inactivation of Pax5 and Cd72 in preleukemic cells
Source: JCI Insight. 2026 Feb 17;11(7):e199464. doi: 10.1172/jci.insight.199464 (PMC13134721; doi:10.1172/jci.insight.199464)
Supplement: Supplemental data set 1 [file jciinsight-11-199464-s204.zip › Strain_Genotyping/Q640-results-report.pdf]

MiniMUGA Background Analysis v2.3.1

**Sample ID**	Q640
**Neogen ID**	AAAU-4546
**Summary**	The genotype of this sample is of **excellent** quality. It is **female** and **outbred**, and likely a mix of **C57BL/6J and C57BL/6NRj** and **CBA/J**. Clustering of unexplained markers is evidence of an additional background strain.<br><br>Diagnostic SNPs are likely explained by the presence of the background strains<br>• Solution 1: C57BL/6J and C57BL/6NRj<br>◦ C57BL/6J: 58 / 160 (36.2%)<br>◦ C57BL/6NRj: 18 / 39 (46.2%)<br>• Solution 2: C57BL/6JRj and C57BL/6NRj<br>◦ C57BL/6JRj: 58 / 160 (36.2%)<br>◦ C57BL/6NRj: 18 / 39 (46.2%)<br><br>No genetic constructs were detected in this sample.
**Genotyping Quality**	**Excellent (2 N calls)**<br>All reported results are dependent on genotyping quality.
**Chromosomal Sex**	XX
**Inbreeding Estimate**	63.9% Inbred<br>(Percentage of the genome (autosomal and X chromosomes) that is homozygous or hemizygous for primary, secondary, and unknown backgrounds. See Genome Analysis)

Constructs Detected

BlastR	bpA	Cas9	chlor	cHS4	Cre	DTA	Flp	g_FP	hCMV_a	hCMV_b	hTK_pr	iCre	IRES	Luc	r_FP	rtTA	SV40	tTA
-	-	-	-	-	-	-	-	-	-	-	-	-	-	-	-	-	-	-

Refined Ideogram

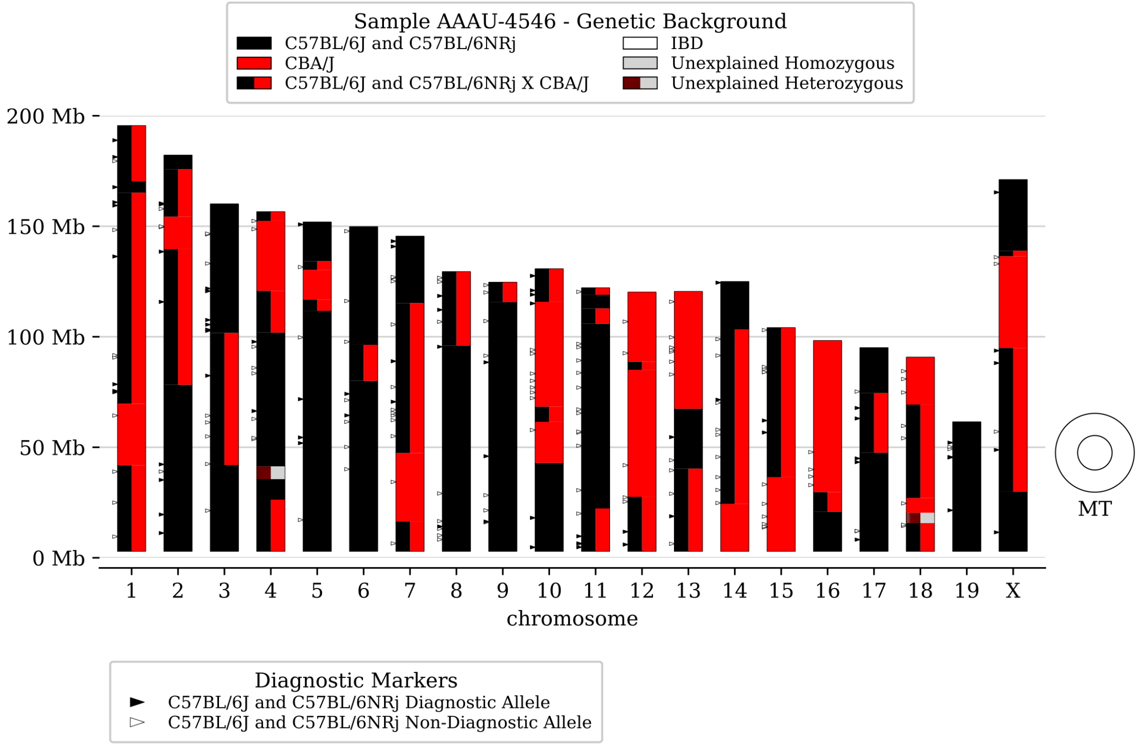

MiniMUGA Background Analysis v2.3.1

Genome Analysis

Background	Zygosity	Informative Markers	Informative Markers %	Genome %
C57BL/6J and C57BL/6NRj	N/A	1130	42.4%	43.7%
CBA/J	Homozygous	579	21.7%	20.2%
C57BL/6J and C57BL/6NRj X CBA/J	Heterozygous	940	35.3%	35.7%
Unexplained	Heterozygous	15	0.6%	0.4%
	**Total**	2664	100.0%	100.0%

Y Chromosome

Not Applicable

MT Genome

MT Haplogroup 6 - 100.0% Consistent
Includes C57BL/6J, C57BL/6NRj, CBA/J and 165 other strains

Backgrounds Detected (Diagnostic Alleles)

	Diagnostic Alleles Observed			
Diagnostic Class	Homozygous	Heterozygous	Potential	% Observed
C57BL/6J, C57BL/6JJicTac, C57BL/6JRj	4	35	102	38.2%
C57BL/6J, C57BL/6JEiJ, C57BL/6JJicTac, C57BL/6JRj	5	5	21	47.6%
C57BL/6NRj, C57BL/6NTac	4	4	15	53.3%
C57BL/6NJ, C57BL/6NRj, C57BL/6NTac	3	2	10	50.0%
C57BL/6J, C57BL/6JRj	0	7	31	22.6%
B6N-Tyr<c-Brd>/BrdCrCrl, C57BL/6J, C57BL/6JEiJ, C57BL/6JJicTac, C57BL/6JRj	1	0	1	100.0%
B6N-Tyr<c-Brd>/BrdCrCrl, C57BL/6NCrl, C57BL/6NHsd, C57BL/6NJ, C57BL/6NRj, C57BL/6NTac	1	0	2	50.0%
C57BL/6NCrl, C57BL/6NHsd, C57BL/6NJ, C57BL/6NRj, C57BL/6NTac	0	2	2	100.0%
C57BL/6NRj	0	2	10	20.0%
B6N-Tyr<c-Brd>/BrdCrCrl, C57BL/6J, C57BL/6JJicTac, C57BL/6JRj	0	1	5	20.0%

Minimal Strain Sets Explaining All Diagnostic Classes (Number of Markers Explained):

- Solution 1: C57BL/6J and C57BL/6NRj
  - C57BL/6J: 58 / 160 (36.2%)
  - C57BL/6NRj: 18 / 39 (46.2%)
- Solution 2: C57BL/6JRj and C57BL/6NRj
  - C57BL/6JRj: 58 / 160 (36.2%)
  - C57BL/6NRj: 18 / 39 (46.2%)

Chromosome	Start (Mb)	Stop (Mb)	Background	Zygosity
1	3000000	41869819	C57BL/6J and C57BL/6NRj and CBA/J	Heterozygous
1	41869819	69700765	CBA/J	Homozygous
1	69700765	165183608	C57BL/6J and C57BL/6NRj and CBA/J	Heterozygous
1	165183608	170316822	C57BL/6J and C57BL/6NRj	N/A
1	170316822	195471971	C57BL/6J and C57BL/6NRj and CBA/J	Heterozygous
2	3000000	78267191	C57BL/6J and C57BL/6NRj	N/A

MiniMUGA Background Analysis v2.3.1

**Diplotype Intervals**	2	78267191	139631657	C57BL/6J and C57BL/6NRj and CBA/J	Heterozygous
	2	139631657	154349372	CBA/J	Homozygous
	2	154349372	175780822	C57BL/6J and C57BL/6NRj and CBA/J	Heterozygous
	2	175780822	182113224	C57BL/6J and C57BL/6NRj	N/A
	3	3000000	41975127	C57BL/6J and C57BL/6NRj	N/A
	3	41975127	101716043	C57BL/6J and C57BL/6NRj and CBA/J	Heterozygous
	3	101716043	160039680	C57BL/6J and C57BL/6NRj	N/A
	4	3000000	26280383	C57BL/6J and C57BL/6NRj and CBA/J	Heterozygous
	4	26280383	35563307	C57BL/6J and C57BL/6NRj	N/A
	4	35563307	41348396	Unexplained	Heterozygous
	4	41348396	101914190	C57BL/6J and C57BL/6NRj	N/A
	4	101914190	120738488	C57BL/6J and C57BL/6NRj and CBA/J	Heterozygous
	4	120738488	152440879	CBA/J	Homozygous
	4	152440879	156508116	C57BL/6J and C57BL/6NRj and CBA/J	Heterozygous
	5	3000000	111745102	C57BL/6J and C57BL/6NRj	N/A
	5	111745102	116795433	C57BL/6J and C57BL/6NRj and CBA/J	Heterozygous
	5	116795433	130280923	CBA/J	Homozygous
	5	130280923	134172373	C57BL/6J and C57BL/6NRj and CBA/J	Heterozygous
	5	134172373	151834684	C57BL/6J and C57BL/6NRj	N/A
	6	3000000	80057017	C57BL/6J and C57BL/6NRj	N/A
	6	80057017	96327282	C57BL/6J and C57BL/6NRj and CBA/J	Heterozygous
	6	96327282	149736546	C57BL/6J and C57BL/6NRj	N/A
	7	3000000	16360273	C57BL/6J and C57BL/6NRj and CBA/J	Heterozygous
	7	16360273	47395440	CBA/J	Homozygous
	7	47395440	115227247	C57BL/6J and C57BL/6NRj and CBA/J	Heterozygous
	7	115227247	145441459	C57BL/6J and C57BL/6NRj	N/A
	8	3000000	96009840	C57BL/6J and C57BL/6NRj	N/A
	8	96009840	129401213	C57BL/6J and C57BL/6NRj and CBA/J	Heterozygous
	9	3000000	115715944	C57BL/6J and C57BL/6NRj	N/A
	9	115715944	124595110	C57BL/6J and C57BL/6NRj and CBA/J	Heterozygous
	10	3000000	42917049	C57BL/6J and C57BL/6NRj	N/A

MiniMUGA Background Analysis v2.3.1

	10	42917049	61450853	CBA/J	Homozygous
	10	61450853	68332199	C57BL/6J and C57BL/6NRj and CBA/J	Heterozygous
	10	68332199	115781736	CBA/J	Homozygous
	10	115781736	130694993	C57BL/6J and C57BL/6NRj and CBA/J	Heterozygous
	11	3000000	22302070	C57BL/6J and C57BL/6NRj and CBA/J	Heterozygous
	11	22302070	105886229	C57BL/6J and C57BL/6NRj	N/A
	11	105886229	112771442	C57BL/6J and C57BL/6NRj and CBA/J	Heterozygous
	11	112771442	119038285	C57BL/6J and C57BL/6NRj	N/A
	11	119038285	122082543	C57BL/6J and C57BL/6NRj and CBA/J	Heterozygous
	12	3000000	27585493	C57BL/6J and C57BL/6NRj and CBA/J	Heterozygous
	12	27585493	85015902	CBA/J	Homozygous
	12	85015902	88650858	C57BL/6J and C57BL/6NRj and CBA/J	Heterozygous
	12	88650858	120129022	CBA/J	Homozygous
	13	3000000	40278277	C57BL/6J and C57BL/6NRj and CBA/J	Heterozygous
	13	40278277	67442927	C57BL/6J and C57BL/6NRj	N/A
	13	67442927	120421639	CBA/J	Homozygous
	14	3000000	24355636	CBA/J	Homozygous
	14	24355636	103377147	C57BL/6J and C57BL/6NRj and CBA/J	Heterozygous
	14	103377147	124902244	C57BL/6J and C57BL/6NRj	N/A
	15	3000000	36473640	CBA/J	Homozygous
	15	36473640	104043685	C57BL/6J and C57BL/6NRj and CBA/J	Heterozygous
	16	3000000	20813513	C57BL/6J and C57BL/6NRj	N/A
	16	20813513	29701002	C57BL/6J and C57BL/6NRj and CBA/J	Heterozygous
	16	29701002	98207768	CBA/J	Homozygous
	17	3000000	47545390	C57BL/6J and C57BL/6NRj	N/A
	17	47545390	74502727	C57BL/6J and C57BL/6NRj and CBA/J	Heterozygous
	17	74502727	94987271	C57BL/6J and C57BL/6NRj	N/A
	18	3000000	15685654	C57BL/6J and C57BL/6NRj and CBA/J	Heterozygous
	18	15685654	20363699	Unexplained	Heterozygous
	18	20363699	27036500	CBA/J	Homozygous
	18	27036500	69337106	C57BL/6J and C57BL/6NRj and CBA/J	Heterozygous
	18	69337106	90702639	CBA/J	Homozygous

MiniMUGA Background Analysis v2.3.1

	19	3000000	61431566	C57BL/6J and C57BL/6NRj	N/A
	X	3000000	29836043	C57BL/6J and C57BL/6NRj	N/A
	X	29836043	94918419	C57BL/6J and C57BL/6NRj and CBA/J	Heterozygous
	X	94918419	136441962	CBA/J	Homozygous
	X	136441962	138881041	C57BL/6J and C57BL/6NRj and CBA/J	Heterozygous
	X	138881041	171031299	C57BL/6J and C57BL/6NRj	N/A
	MT	0	0	IBD	Hemizygous
