# Supplementary material for: Deletion of 9p drives B-ALL through heterozygous inactivation of Pax5 and Cd72 in preleukemic cells
Source: JCI Insight. 2026 Feb 17;11(7):e199464. doi: 10.1172/jci.insight.199464 (PMC13134721; doi:10.1172/jci.insight.199464)
Supplement: Supplemental data set 1 [file jciinsight-11-199464-s204.zip › Strain_Genotyping/Q723-results-report.pdf]

MiniMUGA Background Analysis v2.3.1

**Sample ID**	Q723
**Neogen ID**	AAAU-4495
**Summary**	The genotype of this sample is of **excellent** quality. It is **female** and **outbred**, and likely a mix of **C57BL/6J and C57BL/6NTac** and **CBA/J**. Clustering of unexplained markers is evidence of an additional background strain.<br><br>Diagnostic SNPs are likely explained by the presence of the background strains<br>• Solution 1: C57BL/6J and C57BL/6NRj<br>◦ C57BL/6J: 64 / 155 (41.3%)<br>◦ C57BL/6NRj: 16 / 39 (41.0%)<br>• Solution 2: C57BL/6JRj and C57BL/6NRj<br>◦ C57BL/6JRj: 64 / 155 (41.3%)<br>◦ C57BL/6NRj: 16 / 39 (41.0%)<br><br>NOTE: There is a discrepancy between the diagnostic backgrounds detected and the primary and secondary background analysis (CBA/J, C57BL/6J, C57BL/6NTac). This is uncommon and should be investigated further.<br><br>No genetic constructs were detected in this sample.<br><br>WARNING:<br>• There is a discrepancy between the diagnostic backgrounds detected ((C57BL/6J and C57BL/6NRj) or (C57BL/6JRj and C57BL/6NRj)) and the primary background (C57BL/6J and C57BL/6NTac) and secondary background (CBA/J). This is uncommon and should be investigated further.<br>• This sample likely has more than 2 genetic backgrounds (unexplained regions and/or fractured ideogram). The strain selected for secondary background may be incorrect. The estimation of the contribution of primary and secondary background are likely incorrect. This can potentially be addressed with input from the user.
**Genotyping Quality**	**Excellent (2 N calls)**<br>All reported results are dependent on genotyping quality.
**Chromosomal Sex**	XX
**Inbreeding Estimate**	68.1% Inbred<br>(Percentage of the genome (autosomal and X chromosomes) that is homozygous or hemizygous for primary, secondary, and unknown backgrounds. See Genome Analysis)

Constructs Detected

BlastR	bpA	Cas9	chlor	cHS4	Cre	DTA	Flp	g_FP	hCMV_a	hCMV_b	hTK_pr	iCre	IRES	Luc	r_FP	rtTA	SV40	tTA
-	-	-	-	-	-	-	-	-	-	-	-	-	-	-	-	-	-	-

MiniMUGA Background Analysis v2.3.1

Refined Ideogram

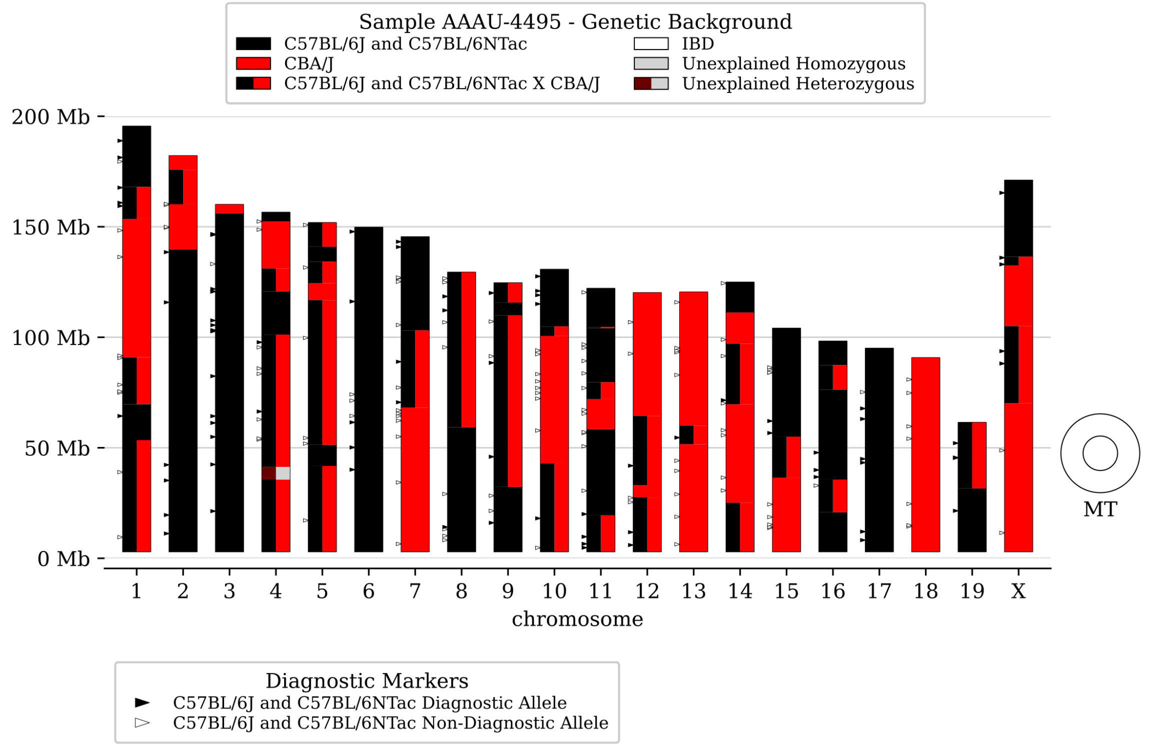

Genome Analysis

Background	Zygosity	Informative Markers	Informative Markers %	Genome %
C57BL/6J and C57BL/6NTac	N/A	1054	39.6%	40.7%
CBA/J	Homozygous	715	26.8%	27.3%
C57BL/6J and C57BL/6NTac X CBA/J	Heterozygous	885	33.2%	31.7%
Unexplained	Heterozygous	10	0.4%	0.2%
	**Total**	2664	100.0%	99.9%

**Y Chromosome**	Not Applicable
**MT Genome**	MT Haplogroup 6 - 100.0% Consistent<br>Includes C57BL/6J, C57BL/6NTac, CBA/J and 165 other strains

MiniMUGA Background Analysis v2.3.1

Backgrounds Detected (Diagnostic Alleles)

	Diagnostic Alleles Observed			
**Diagnostic Class**	**Homozygous**	**Heterozygous**	**Potential**	**% Observed**
C57BL/6J, C57BL/6JJicTac, C57BL/6JRj	14	29	102	42.2%
C57BL/6J, C57BL/6JRj	4	6	31	32.3%
C57BL/6J, C57BL/6JEiJ, C57BL/6JJicTac, C57BL/6JRj	2	8	21	47.6%
C57BL/6NRj, C57BL/6NTac	2	5	15	46.7%
C57BL/6NJ, C57BL/6NRj, C57BL/6NTac	1	4	10	50.0%
B6N-Tyr<c-Brd>/BrdCrCrl, C57BL/6NCrl, C57BL/6NHsd, C57BL/6NJ, C57BL/6NRj, C57BL/6NTac	1	0	2	50.0%
C57BL/6NCrl, C57BL/6NHsd, C57BL/6NJ, C57BL/6NRj, C57BL/6NTac	0	2	2	100.0%
B6N-Tyr<c-Brd>/BrdCrCrl, C57BL/6J, C57BL/6JEiJ, C57BL/6JJicTac, C57BL/6JRj	0	1	1	100.0%
C57BL/6NRj	0	1	10	10.0%

Minimal Strain Sets Explaining All Diagnostic Classes (Number of Markers Explained):

- Solution 1: C57BL/6J and C57BL/6NRj
  - C57BL/6J: 64 / 155 (41.3%)
  - C57BL/6NRj: 16 / 39 (41.0%)
- Solution 2: C57BL/6JRj and C57BL/6NRj
  - C57BL/6JRj: 64 / 155 (41.3%)
  - C57BL/6NRj: 16 / 39 (41.0%)

Diplotype Intervals

Chromosome	Start (Mb)	Stop (Mb)	Background	Zygosity
1	3000000	53457225	C57BL/6J and C57BL/6NTac and CBA/J	Heterozygous
1	53457225	69700765	C57BL/6J and C57BL/6NTac	N/A
1	69700765	90903197	C57BL/6J and C57BL/6NTac and CBA/J	Heterozygous
1	90903197	153548642	CBA/J	Homozygous
1	153548642	168019536	C57BL/6J and C57BL/6NTac and CBA/J	Heterozygous
1	168019536	195471971	C57BL/6J and C57BL/6NTac	N/A
2	3000000	139631657	C57BL/6J and C57BL/6NTac	N/A
2	139631657	160174252	CBA/J	Homozygous
2	160174252	175780822	C57BL/6J and C57BL/6NTac and CBA/J	Heterozygous
2	175780822	182113224	CBA/J	Homozygous
3	3000000	156090101	C57BL/6J and C57BL/6NTac	N/A
3	156090101	160039680	CBA/J	Homozygous
4	3000000	35563307	C57BL/6J and C57BL/6NTac and CBA/J	Heterozygous
4	35563307	41348396	Unexplained	Heterozygous
4	41348396	101144501	C57BL/6J and C57BL/6NTac and CBA/J	Heterozygous
4	101144501	120738488	C57BL/6J and C57BL/6NTac	N/A

MiniMUGA Background Analysis v2.3.1

4	120738488	131104093	C57BL/6J and C57BL/6NTac and CBA/J	Heterozygous
4	131104093	152440879	CBA/J	Homozygous
4	152440879	156508116	C57BL/6J and C57BL/6NTac	N/A
5	3000000	41755530	C57BL/6J and C57BL/6NTac and CBA/J	Heterozygous
5	41755530	51299144	C57BL/6J and C57BL/6NTac	N/A
5	51299144	116795433	C57BL/6J and C57BL/6NTac and CBA/J	Heterozygous
5	116795433	124446826	CBA/J	Homozygous
5	124446826	134172373	C57BL/6J and C57BL/6NTac and CBA/J	Heterozygous
5	134172373	140985717	C57BL/6J and C57BL/6NTac	N/A
5	140985717	151834684	C57BL/6J and C57BL/6NTac and CBA/J	Heterozygous
6	3000000	149736546	C57BL/6J and C57BL/6NTac	N/A
7	3000000	68153750	CBA/J	Homozygous
7	68153750	103084424	C57BL/6J and C57BL/6NTac and CBA/J	Heterozygous
7	103084424	145441459	C57BL/6J and C57BL/6NTac	N/A
8	3000000	59285811	C57BL/6J and C57BL/6NTac	N/A
8	59285811	129401213	C57BL/6J and C57BL/6NTac and CBA/J	Heterozygous
9	3000000	32287190	C57BL/6J and C57BL/6NTac	N/A
9	32287190	109855467	C57BL/6J and C57BL/6NTac and CBA/J	Heterozygous
9	109855467	115715944	C57BL/6J and C57BL/6NTac	N/A
9	115715944	124595110	C57BL/6J and C57BL/6NTac and CBA/J	Heterozygous
10	3000000	42858234	C57BL/6J and C57BL/6NTac and CBA/J	Heterozygous
10	42858234	100561092	CBA/J	Homozygous
10	100561092	104861956	C57BL/6J and C57BL/6NTac and CBA/J	Heterozygous
10	104861956	130694993	C57BL/6J and C57BL/6NTac	N/A
11	3000000	19463075	C57BL/6J and C57BL/6NTac and CBA/J	Heterozygous
11	19463075	58406228	C57BL/6J and C57BL/6NTac	N/A
11	58406228	72044583	CBA/J	Homozygous
11	72044583	79617327	C57BL/6J and C57BL/6NTac and CBA/J	Heterozygous
11	79617327	104154012	C57BL/6J and C57BL/6NTac	N/A
11	104154012	104675339	C57BL/6J and C57BL/6NTac and CBA/J	Heterozygous
11	104675339	122082543	C57BL/6J and C57BL/6NTac	N/A

MiniMUGA Background Analysis v2.3.1

	12	3000000	27585493	C57BL/6J and C57BL/6NTac and CBA/J	Heterozygous
	12	27585493	33130555	CBA/J	Homozygous
	12	33130555	64411355	C57BL/6J and C57BL/6NTac and CBA/J	Heterozygous
	12	64411355	120129022	CBA/J	Homozygous
	13	3000000	51605798	CBA/J	Homozygous
	13	51605798	60016573	C57BL/6J and C57BL/6NTac and CBA/J	Heterozygous
	13	60016573	120421639	CBA/J	Homozygous
	14	3000000	25112834	C57BL/6J and C57BL/6NTac and CBA/J	Heterozygous
	14	25112834	69660428	CBA/J	Homozygous
	14	69660428	97106405	C57BL/6J and C57BL/6NTac and CBA/J	Heterozygous
	14	97106405	111185375	CBA/J	Homozygous
	14	111185375	124902244	C57BL/6J and C57BL/6NTac	N/A
	15	3000000	36473640	CBA/J	Homozygous
	15	36473640	55016741	C57BL/6J and C57BL/6NTac and CBA/J	Heterozygous
	15	55016741	104043685	C57BL/6J and C57BL/6NTac	N/A
	16	3000000	20813513	C57BL/6J and C57BL/6NTac	N/A
	16	20813513	35579120	C57BL/6J and C57BL/6NTac and CBA/J	Heterozygous
	16	35579120	76315797	C57BL/6J and C57BL/6NTac	N/A
	16	76315797	87403166	C57BL/6J and C57BL/6NTac and CBA/J	Heterozygous
	16	87403166	98207768	C57BL/6J and C57BL/6NTac	N/A
	17	3000000	94987271	C57BL/6J and C57BL/6NTac	N/A
	18	3000000	90702639	CBA/J	Homozygous
	19	3000000	31636352	C57BL/6J and C57BL/6NTac	N/A
	19	31636352	61431566	C57BL/6J and C57BL/6NTac and CBA/J	Heterozygous
	X	3000000	70193631	CBA/J	Homozygous
	X	70193631	105020820	C57BL/6J and C57BL/6NTac and CBA/J	Heterozygous
	X	105020820	132528229	CBA/J	Homozygous
	X	132528229	136441962	C57BL/6J and C57BL/6NTac and CBA/J	Heterozygous
	X	136441962	171031299	C57BL/6J and C57BL/6NTac	N/A
	MT	0	0	IBD	Hemizygous
