# Supplementary material for: Deletion of 9p drives B-ALL through heterozygous inactivation of Pax5 and Cd72 in preleukemic cells
Source: JCI Insight. 2026 Feb 17;11(7):e199464. doi: 10.1172/jci.insight.199464 (PMC13134721; doi:10.1172/jci.insight.199464)
Supplement: Supplemental data set 1 [file jciinsight-11-199464-s204.zip › Strain_Genotyping/Q724-results-report.pdf]

MiniMUGA Background Analysis v2.3.1

<table>
<tr><th>Sample ID</th><td>Q724</td></tr>
<tr><th>Neogen ID</th><td>AAAU-4549</td></tr>
<tr><th>Summary</th><td>The genotype of this sample is of excellent quality. It is female and outbred, and likely a mix of C57BL/6J and C57BL/6NTac and CBA/J. Clustering of unexplained markers is evidence of an additional background strain.

Diagnostic SNPs are likely explained by the presence of the background strains
Solution 1: C57BL/6J and C57BL/6NRj
C57BL/6J: 55 / 162 (34.0%)
C57BL/6NRj: 22 / 40 (55.0%)
Solution 2: C57BL/6JRj and C57BL/6NRj
C57BL/6JRj: 55 / 162 (34.0%)
C57BL/6NRj: 22 / 40 (55.0%)
NOTE: There is a discrepancy between the diagnostic backgrounds detected and the primary and secondary background analysis (CBA/J, C57BL/6J, C57BL/6NTac). This is uncommon and should be investigated further.

No genetic constructs were detected in this sample.

WARNING:
There is a discrepancy between the diagnostic backgrounds detected ((C57BL/6J and C57BL/6NRj) or (C57BL/6JRj and C57BL/6NRj)) and the primary background (C57BL/6J and C57BL/6NTac) and secondary background (CBA/J). This is uncommon and should be investigated further.</td></tr>
<tr><th>Genotyping Quality</th><td>Excellent (14 N calls)
All reported results are dependent on genotyping quality.</td></tr>
<tr><th>Chromosomal Sex</th><td>XX</td></tr>
<tr><th>Inbreeding Estimate</th><td>65.4% Inbred
(Percentage of the genome (autosomal and X chromosomes) that is homozygous or hemizygous for primary, secondary, and unknown backgrounds. See Genome Analysis)</td></tr>
<tr><th>Constructs Detected</th><td>

BlastR	bpA	Cas9	chlor	cHS4	Cre	DTA	Flp	g_FP	hCMV_a	hCMV_b	hTK_pr	iCre	IRES	Luc	r_FP	rtTA	SV40	tTA
-	-	-	-	-	-	-	-	-	-	-	-	-	-	-	-	-	-	-

</td></tr>
</table>

MiniMUGA Background Analysis v2.3.1

Refined Ideogram

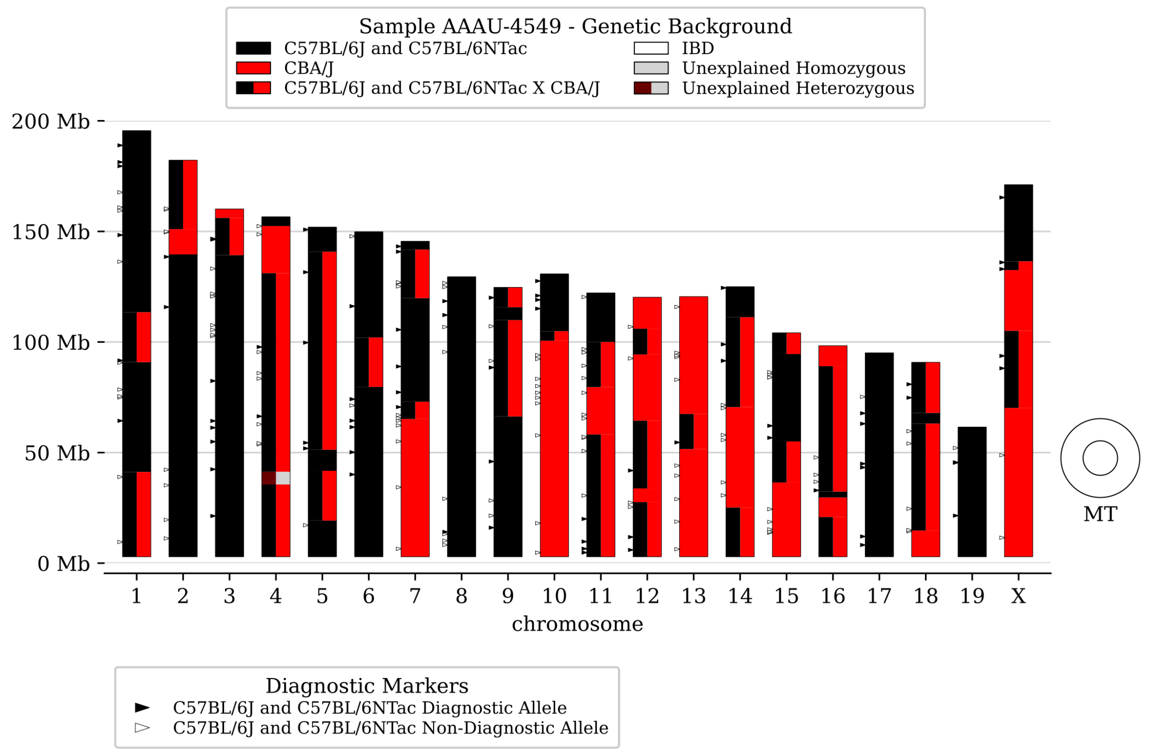

Genome Analysis

Background	Zygosity	Informative Markers	Informative Markers %	Genome %
C57BL/6J and C57BL/6NTac	N/A	1193	45.0%	43.1%
CBA/J	Homozygous	610	23.0%	22.3%
C57BL/6J and C57BL/6NTac X CBA/J	Heterozygous	836	31.5%	34.4%
Unexplained	Heterozygous	11	0.4%	0.2%
	**Total**	2650	99.9%	100.0%

Y Chromosome

Not Applicable

MT Genome

MT Haplogroup 6 - 100.0% Consistent
Includes C57BL/6J, C57BL/6NTac, CBA/J and 165 other strains

MiniMUGA Background Analysis v2.3.1

Backgrounds Detected (Diagnostic Alleles)

	Diagnostic Alleles Observed			
**Diagnostic Class**	**Homozygous**	**Heterozygous**	**Potential**	**% Observed**
C57BL/6J, C57BL/6JJicTac, C57BL/6JRj	5	31	102	35.3%
C57BL/6NRj, C57BL/6NTac	3	8	15	73.3%
C57BL/6J, C57BL/6JRj	4	4	31	25.8%
C57BL/6NJ, C57BL/6NRj, C57BL/6NTac	4	2	10	60.0%
C57BL/6J, C57BL/6JEiJ, C57BL/6JJicTac, C57BL/6JRj	1	7	21	38.1%
C57BL/6NCrl, C57BL/6NHsd, C57BL/6NJ, C57BL/6NRj, C57BL/6NTac	0	2	2	100.0%
B6N-Tyr<c-Brd>/BrdCrCrl, C57BL/6J, C57BL/6JBomTac, C57BL/6JEiJ, C57BL/6JJicTac, C57BL/6JOlaHsd, C57BL/6JRj	0	1	2	50.0%
B6N-Tyr<c-Brd>/BrdCrCrl, C57BL/6J, C57BL/6JEiJ, C57BL/6JJicTac, C57BL/6JRj	0	1	1	100.0%
B6N-Tyr<c-Brd>/BrdCrCrl, C57BL/6J, C57BL/6JJicTac, C57BL/6JRj	0	1	5	20.0%
B6N-Tyr<c-Brd>/BrdCrCrl, C57BL/6NCrl, C57BL/6NHsd, C57BL/6NJ, C57BL/6NRj, C57BL/6NTac	0	1	2	50.0%
C57BL/6NHsd, C57BL/6NJ, C57BL/6NRj, C57BL/6NTac	0	1	1	100.0%
C57BL/6NRj	0	1	10	10.0%

Minimal Strain Sets Explaining All Diagnostic Classes (Number of Markers Explained):

- Solution 1: C57BL/6J and C57BL/6NRj
  - C57BL/6J: 55 / 162 (34.0%)
  - C57BL/6NRj: 22 / 40 (55.0%)
- Solution 2: C57BL/6JRj and C57BL/6NRj
  - C57BL/6JRj: 55 / 162 (34.0%)
  - C57BL/6NRj: 22 / 40 (55.0%)

Chromosome	Start (Mb)	Stop (Mb)	Background	Zygosity
1	3000000	41199760	C57BL/6J and C57BL/6NTac and CBA/J	Heterozygous
1	41199760	90903197	C57BL/6J and C57BL/6NTac	N/A
1	90903197	113437009	C57BL/6J and C57BL/6NTac and CBA/J	Heterozygous
1	113437009	195471971	C57BL/6J and C57BL/6NTac	N/A
2	3000000	139631657	C57BL/6J and C57BL/6NTac	N/A
2	139631657	151062687	CBA/J	Homozygous
2	151062687	182113224	C57BL/6J and C57BL/6NTac and CBA/J	Heterozygous
3	3000000	139297311	C57BL/6J and C57BL/6NTac	N/A
3	139297311	156090101	C57BL/6J and C57BL/6NTac and CBA/J	Heterozygous
3	156090101	160039680	CBA/J	Homozygous
4	3000000	35563307	C57BL/6J and C57BL/6NTac and CBA/J	Heterozygous
4	35563307	41348396	Unexplained	Heterozygous

MiniMUGA Background Analysis v2.3.1

**Diplotype Intervals**	4	41348396	131104093	C57BL/6J and C57BL/6NTac and CBA/J	Heterozygous
	4	131104093	152440879	CBA/J	Homozygous
	4	152440879	156508116	C57BL/6J and C57BL/6NTac	N/A
	5	3000000	19267794	C57BL/6J and C57BL/6NTac	N/A
	5	19267794	41755530	C57BL/6J and C57BL/6NTac and CBA/J	Heterozygous
	5	41755530	51299144	C57BL/6J and C57BL/6NTac	N/A
	5	51299144	140780463	C57BL/6J and C57BL/6NTac and CBA/J	Heterozygous
	5	140780463	151834684	C57BL/6J and C57BL/6NTac	N/A
	6	3000000	79701235	C57BL/6J and C57BL/6NTac	N/A
	6	79701235	101966063	C57BL/6J and C57BL/6NTac and CBA/J	Heterozygous
	6	101966063	149736546	C57BL/6J and C57BL/6NTac	N/A
	7	3000000	65214761	CBA/J	Homozygous
	7	65214761	72944748	C57BL/6J and C57BL/6NTac and CBA/J	Heterozygous
	7	72944748	119823617	C57BL/6J and C57BL/6NTac	N/A
	7	119823617	141750158	C57BL/6J and C57BL/6NTac and CBA/J	Heterozygous
	7	141750158	145441459	C57BL/6J and C57BL/6NTac	N/A
	8	3000000	129401213	C57BL/6J and C57BL/6NTac	N/A
	9	3000000	66341356	C57BL/6J and C57BL/6NTac	N/A
	9	66341356	109855467	C57BL/6J and C57BL/6NTac and CBA/J	Heterozygous
	9	109855467	115715944	C57BL/6J and C57BL/6NTac	N/A
	9	115715944	124595110	C57BL/6J and C57BL/6NTac and CBA/J	Heterozygous
	10	3000000	100561092	CBA/J	Homozygous
	10	100561092	104861956	C57BL/6J and C57BL/6NTac and CBA/J	Heterozygous
	10	104861956	130694993	C57BL/6J and C57BL/6NTac	N/A
	11	3000000	58168384	C57BL/6J and C57BL/6NTac and CBA/J	Heterozygous
	11	58168384	79617327	CBA/J	Homozygous
	11	79617327	99943553	C57BL/6J and C57BL/6NTac and CBA/J	Heterozygous
	11	99943553	122082543	C57BL/6J and C57BL/6NTac	N/A
	12	3000000	27585493	C57BL/6J and C57BL/6NTac and CBA/J	Heterozygous
	12	27585493	33782811	CBA/J	Homozygous
	12	33782811	64411355	C57BL/6J and C57BL/6NTac and CBA/J	Heterozygous

MiniMUGA Background Analysis v2.3.1

	12	64411355	94371165	CBA/J	Homozygous
	12	94371165	105994851	C57BL/6J and C57BL/6NTac and CBA/J	Heterozygous
	12	105994851	120129022	CBA/J	Homozygous
	13	3000000	51605798	CBA/J	Homozygous
	13	51605798	67442927	C57BL/6J and C57BL/6NTac and CBA/J	Heterozygous
	13	67442927	120421639	CBA/J	Homozygous
	14	3000000	25112834	C57BL/6J and C57BL/6NTac and CBA/J	Heterozygous
	14	25112834	70580779	CBA/J	Homozygous
	14	70580779	111185375	C57BL/6J and C57BL/6NTac and CBA/J	Heterozygous
	14	111185375	124902244	C57BL/6J and C57BL/6NTac	N/A
	15	3000000	36473640	CBA/J	Homozygous
	15	36473640	55016741	C57BL/6J and C57BL/6NTac and CBA/J	Heterozygous
	15	55016741	94556557	C57BL/6J and C57BL/6NTac	N/A
	15	94556557	104043685	C57BL/6J and C57BL/6NTac and CBA/J	Heterozygous
	16	3000000	20813513	C57BL/6J and C57BL/6NTac and CBA/J	Heterozygous
	16	20813513	29701002	CBA/J	Homozygous
	16	29701002	32364421	C57BL/6J and C57BL/6NTac	N/A
	16	32364421	89037512	C57BL/6J and C57BL/6NTac and CBA/J	Heterozygous
	16	89037512	98207768	CBA/J	Homozygous
	17	3000000	94987271	C57BL/6J and C57BL/6NTac	N/A
	18	3000000	14753212	CBA/J	Homozygous
	18	14753212	63069205	C57BL/6J and C57BL/6NTac and CBA/J	Heterozygous
	18	63069205	67937187	C57BL/6J and C57BL/6NTac	N/A
	18	67937187	90702639	C57BL/6J and C57BL/6NTac and CBA/J	Heterozygous
	19	3000000	61431566	C57BL/6J and C57BL/6NTac	N/A
	X	3000000	70193631	CBA/J	Homozygous
	X	70193631	105020820	C57BL/6J and C57BL/6NTac and CBA/J	Heterozygous
	X	105020820	132528229	CBA/J	Homozygous
	X	132528229	136441962	C57BL/6J and C57BL/6NTac and CBA/J	Heterozygous
	X	136441962	171031299	C57BL/6J and C57BL/6NTac	N/A
	MT	0	0	IBD	Hemizygous
