# Supplementary material for: Deletion of 9p drives B-ALL through heterozygous inactivation of Pax5 and Cd72 in preleukemic cells
Source: JCI Insight. 2026 Feb 17;11(7):e199464. doi: 10.1172/jci.insight.199464 (PMC13134721; doi:10.1172/jci.insight.199464)
Supplement: Supplemental data set 1 [file jciinsight-11-199464-s204.zip › Strain_Genotyping/Q725-results-report.pdf]

MiniMUGA Background Analysis v2.3.1

<table>
<tr><td>Sample ID</td><td>Q725</td></tr>
<tr><td>Neogen ID</td><td>AAAU-4496</td></tr>
<tr><td>Summary</td><td>The genotype of this sample is of excellent quality. It is female and outbred, and likely a mix of C57BL/6J and C57BL/6NTac and CBA/J. Clustering of unexplained markers is evidence of an additional background strain.

Diagnostic SNPs are likely explained by the presence of the background strains
Solution 1: C57BL/6J and C57BL/6NRj
C57BL/6J: 73 / 155 (47.1%)
C57BL/6NRj: 16 / 37 (43.2%)
Solution 2: C57BL/6JRj and C57BL/6NRj
C57BL/6JRj: 73 / 155 (47.1%)
C57BL/6NRj: 16 / 37 (43.2%)
NOTE: There is a discrepancy between the diagnostic backgrounds detected and the primary and secondary background analysis (CBA/J, C57BL/6J, C57BL/6NTac). This is uncommon and should be investigated further.

No genetic constructs were detected in this sample.

WARNING:
There is a discrepancy between the diagnostic backgrounds detected ((C57BL/6J and C57BL/6NRj) or (C57BL/6JRj and C57BL/6NRj)) and the primary background (C57BL/6J and C57BL/6NTac) and secondary background (CBA/J). This is uncommon and should be investigated further.
This sample likely has more than 2 genetic backgrounds (unexplained regions and/or fractured ideogram). The strain selected for secondary background may be incorrect. The estimation of the contribution of primary and secondary background are likely incorrect. This can potentially be addressed with input from the user.</td></tr>
<tr><td>Genotyping Quality</td><td>Excellent (17 N calls)
All reported results are dependent on genotyping quality.</td></tr>
<tr><td>Chromosomal Sex</td><td>XX</td></tr>
<tr><td>Inbreeding Estimate</td><td>64.6% Inbred
(Percentage of the genome (autosomal and X chromosomes) that is homozygous or hemizygous for primary, secondary, and unknown backgrounds. See Genome Analysis)</td></tr>
<tr><td>Constructs Detected</td><td>

BlastR	bpA	Cas9	chlor	cHS4	Cre	DTA	Flp	g_FP	hCMV_a	hCMV_b	hTK_pr	iCre	IRES	Luc	r_FP	rtTA	SV40	tTA
-	-	-	-	-	-	-	-	-	-	-	-	-	-	-	-	-	-	-

</td></tr>
</table>

MiniMUGA Background Analysis v2.3.1

Refined Ideogram

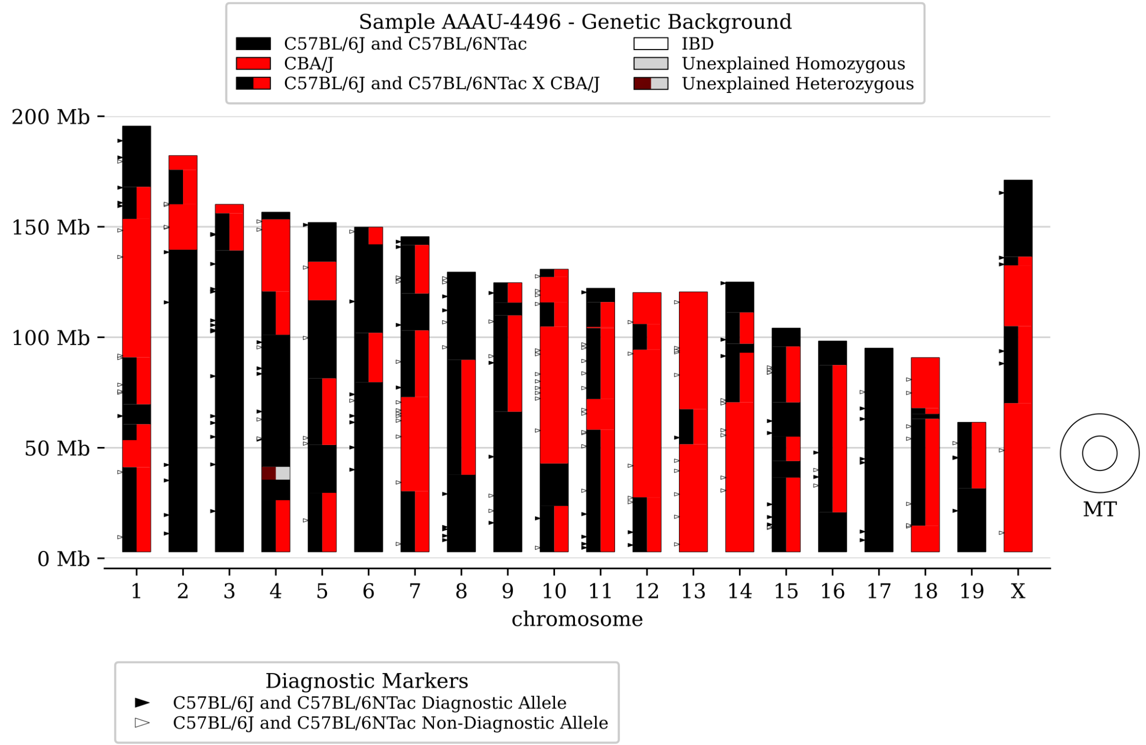

Genome Analysis

Background	Zygosity	Informative Markers	Informative Markers %	Genome %
C57BL/6J and C57BL/6NTac	N/A	962	36.2%	38.8%
CBA/J	Homozygous	666	25.1%	25.9%
C57BL/6J and C57BL/6NTac X CBA/J	Heterozygous	1016	38.3%	35.1%
Unexplained	Heterozygous	11	0.4%	0.2%
	**Total**	2655	100.0%	100.0%

Y Chromosome: Not Applicable

MT Genome: MT Haplogroup 6 - 100.0% Consistent
Includes C57BL/6J, C57BL/6NTac, CBA/J and 165 other strains

MiniMUGA Background Analysis v2.3.1

Backgrounds Detected (Diagnostic Alleles)

Diagnostic Class	Diagnostic Alleles Observed: Homozygous	Heterozygous	Potential	% Observed
C57BL/6J, C57BL/6JJicTac, C57BL/6JRj	6	42	102	47.1%
C57BL/6J, C57BL/6JRj	5	6	31	35.5%
C57BL/6J, C57BL/6JEiJ, C57BL/6JJicTac, C57BL/6JRj	2	11	21	61.9%
C57BL/6NRj, C57BL/6NTac	2	7	15	60.0%
C57BL/6NJ, C57BL/6NRj, C57BL/6NTac	1	4	10	50.0%
B6N-Tyr<c-Brd>/BrdCrCrl, C57BL/6J, C57BL/6JEiJ, C57BL/6JJicTac, C57BL/6JRj	1	0	1	100.0%
B6N-Tyr<c-Brd>/BrdCrCrl, C57BL/6NCrl, C57BL/6NHsd, C57BL/6NJ, C57BL/6NRj, C57BL/6NTac	0	1	2	50.0%
C57BL/6NRj	0	1	10	10.0%

Minimal Strain Sets Explaining All Diagnostic Classes (Number of Markers Explained):

- Solution 1: C57BL/6J and C57BL/6NRj
  - C57BL/6J: 73 / 155 (47.1%)
  - C57BL/6NRj: 16 / 37 (43.2%)
- Solution 2: C57BL/6JRj and C57BL/6NRj
  - C57BL/6JRj: 73 / 155 (47.1%)
  - C57BL/6NRj: 16 / 37 (43.2%)

Diplotype Intervals

Chromosome	Start (Mb)	Stop (Mb)	Background	Zygosity
1	3000000	41199760	C57BL/6J and C57BL/6NTac and CBA/J	Heterozygous
1	41199760	53457225	CBA/J	Homozygous
1	53457225	60621237	C57BL/6J and C57BL/6NTac and CBA/J	Heterozygous
1	60621237	69700765	C57BL/6J and C57BL/6NTac	N/A
1	69700765	90903197	C57BL/6J and C57BL/6NTac and CBA/J	Heterozygous
1	90903197	153548642	CBA/J	Homozygous
1	153548642	168019536	C57BL/6J and C57BL/6NTac and CBA/J	Heterozygous
1	168019536	195471971	C57BL/6J and C57BL/6NTac	N/A
2	3000000	139631657	C57BL/6J and C57BL/6NTac	N/A
2	139631657	160174252	CBA/J	Homozygous
2	160174252	175780822	C57BL/6J and C57BL/6NTac and CBA/J	Heterozygous
2	175780822	182113224	CBA/J	Homozygous
3	3000000	139297311	C57BL/6J and C57BL/6NTac	N/A
3	139297311	156090101	C57BL/6J and C57BL/6NTac and CBA/J	Heterozygous
3	156090101	160039680	CBA/J	Homozygous
4	3000000	26280383	C57BL/6J and C57BL/6NTac and CBA/J	Heterozygous
4	26280383	35563307	C57BL/6J and C57BL/6NTac	N/A

4	35563307	41348396	Unexplained	Heterozygous
4	41348396	101144501	C57BL/6J and C57BL/6NTac	N/A
4	101144501	120738488	C57BL/6J and C57BL/6NTac and CBA/J	Heterozygous
4	120738488	153356388	CBA/J	Homozygous
4	153356388	156508116	C57BL/6J and C57BL/6NTac	N/A
5	3000000	29588943	C57BL/6J and C57BL/6NTac and CBA/J	Heterozygous
5	29588943	51299144	C57BL/6J and C57BL/6NTac	N/A
5	51299144	81363817	C57BL/6J and C57BL/6NTac and CBA/J	Heterozygous
5	81363817	116795433	C57BL/6J and C57BL/6NTac	N/A
5	116795433	134172373	CBA/J	Homozygous
5	134172373	151834684	C57BL/6J and C57BL/6NTac	N/A
6	3000000	79701235	C57BL/6J and C57BL/6NTac	N/A
6	79701235	101966063	C57BL/6J and C57BL/6NTac and CBA/J	Heterozygous
6	101966063	142043514	C57BL/6J and C57BL/6NTac	N/A
6	142043514	149736546	C57BL/6J and C57BL/6NTac and CBA/J	Heterozygous
7	3000000	30335112	C57BL/6J and C57BL/6NTac and CBA/J	Heterozygous
7	30335112	72944748	CBA/J	Homozygous
7	72944748	103084424	C57BL/6J and C57BL/6NTac and CBA/J	Heterozygous
7	103084424	119823617	C57BL/6J and C57BL/6NTac	N/A
7	119823617	141750158	C57BL/6J and C57BL/6NTac and CBA/J	Heterozygous
7	141750158	145441459	C57BL/6J and C57BL/6NTac	N/A
8	3000000	37790271	C57BL/6J and C57BL/6NTac	N/A
8	37790271	89745720	C57BL/6J and C57BL/6NTac and CBA/J	Heterozygous
8	89745720	129401213	C57BL/6J and C57BL/6NTac	N/A
9	3000000	66341356	C57BL/6J and C57BL/6NTac	N/A
9	66341356	109855467	C57BL/6J and C57BL/6NTac and CBA/J	Heterozygous
9	109855467	115715944	C57BL/6J and C57BL/6NTac	N/A
9	115715944	124595110	C57BL/6J and C57BL/6NTac and CBA/J	Heterozygous
10	3000000	23654421	C57BL/6J and C57BL/6NTac and CBA/J	Heterozygous
10	23654421	42917049	C57BL/6J and C57BL/6NTac	N/A
10	42917049	104861956	CBA/J	Homozygous

MiniMUGA Background Analysis v2.3.1

	10	104861956	115781736	C57BL/6J and C57BL/6NTac and CBA/J	Heterozygous
	10	115781736	127271560	CBA/J	Homozygous
	10	127271560	130694993	C57BL/6J and C57BL/6NTac and CBA/J	Heterozygous
	11	3000000	58168384	C57BL/6J and C57BL/6NTac and CBA/J	Heterozygous
	11	58168384	72044583	CBA/J	Homozygous
	11	72044583	104154012	C57BL/6J and C57BL/6NTac and CBA/J	Heterozygous
	11	104154012	104675339	CBA/J	Homozygous
	11	104675339	115896459	C57BL/6J and C57BL/6NTac and CBA/J	Heterozygous
	11	115896459	122082543	C57BL/6J and C57BL/6NTac	N/A
	12	3000000	27585493	C57BL/6J and C57BL/6NTac and CBA/J	Heterozygous
	12	27585493	94371165	CBA/J	Homozygous
	12	94371165	105994851	C57BL/6J and C57BL/6NTac and CBA/J	Heterozygous
	12	105994851	120129022	CBA/J	Homozygous
	13	3000000	51605798	CBA/J	Homozygous
	13	51605798	67442927	C57BL/6J and C57BL/6NTac and CBA/J	Heterozygous
	13	67442927	120421639	CBA/J	Homozygous
	14	3000000	70580779	CBA/J	Homozygous
	14	70580779	93002544	C57BL/6J and C57BL/6NTac and CBA/J	Heterozygous
	14	93002544	97106405	C57BL/6J and C57BL/6NTac	N/A
	14	97106405	111185375	C57BL/6J and C57BL/6NTac and CBA/J	Heterozygous
	14	111185375	124902244	C57BL/6J and C57BL/6NTac	N/A
	15	3000000	36473640	C57BL/6J and C57BL/6NTac and CBA/J	Heterozygous
	15	36473640	44010563	C57BL/6J and C57BL/6NTac	N/A
	15	44010563	55016741	C57BL/6J and C57BL/6NTac and CBA/J	Heterozygous
	15	55016741	70554147	C57BL/6J and C57BL/6NTac	N/A
	15	70554147	95814574	C57BL/6J and C57BL/6NTac and CBA/J	Heterozygous
	15	95814574	104043685	C57BL/6J and C57BL/6NTac	N/A
	16	3000000	20813513	C57BL/6J and C57BL/6NTac	N/A
	16	20813513	87403166	C57BL/6J and C57BL/6NTac and CBA/J	Heterozygous
	16	87403166	98207768	C57BL/6J and C57BL/6NTac	N/A
	17	3000000	94987271	C57BL/6J and C57BL/6NTac	N/A
	18	3000000	14753212	CBA/J	Homozygous

	18	14753212	63069205	C57BL/6J and C57BL/6NTac and CBA/J	Heterozygous
	18	63069205	65345130	C57BL/6J and C57BL/6NTac	N/A
	18	65345130	67937187	C57BL/6J and C57BL/6NTac and CBA/J	Heterozygous
	18	67937187	90702639	CBA/J	Homozygous
	19	3000000	31636352	C57BL/6J and C57BL/6NTac	N/A
	19	31636352	61431566	C57BL/6J and C57BL/6NTac and CBA/J	Heterozygous
	X	3000000	70193631	CBA/J	Homozygous
	X	70193631	105020820	C57BL/6J and C57BL/6NTac and CBA/J	Heterozygous
	X	105020820	132528229	CBA/J	Homozygous
	X	132528229	136441962	C57BL/6J and C57BL/6NTac and CBA/J	Heterozygous
	X	136441962	171031299	C57BL/6J and C57BL/6NTac	N/A
	MT	0	0	IBD	Hemizygous
