# Supplementary material for: Deletion of 9p drives B-ALL through heterozygous inactivation of Pax5 and Cd72 in preleukemic cells
Source: JCI Insight. 2026 Feb 17;11(7):e199464. doi: 10.1172/jci.insight.199464 (PMC13134721; doi:10.1172/jci.insight.199464)
Supplement: Supplemental data set 1 [file jciinsight-11-199464-s204.zip › Strain_Genotyping/Q726-results-report.pdf]

MiniMUGA Background Analysis v2.3.1

**Sample ID**	Q726
**Neogen ID**	AAAU-4497
**Summary**	The genotype of this sample is of **excellent** quality. It is **female** and **outbred**, and likely a mix of **C57BL/6J and C57BL/6NTac** and **CBA/J**. Clustering of unexplained markers is evidence of an additional background strain.<br><br>Diagnostic SNPs are likely explained by the presence of the background strains<br>• Solution 1: C57BL/6J and C57BL/6NRj<br>◦ C57BL/6J: 68 / 160 (42.5%)<br>◦ C57BL/6NRj: 19 / 40 (47.5%)<br>• Solution 2: C57BL/6JRj and C57BL/6NRj<br>◦ C57BL/6JRj: 68 / 160 (42.5%)<br>◦ C57BL/6NRj: 19 / 40 (47.5%)<br><br>NOTE: There is a discrepancy between the diagnostic backgrounds detected and the primary and secondary background analysis (CBA/J, C57BL/6J, C57BL/6NTac). This is uncommon and should be investigated further.<br><br>No genetic constructs were detected in this sample.<br><br>WARNING:<br>• There is a discrepancy between the diagnostic backgrounds detected ((C57BL/6J and C57BL/6NRj) or (C57BL/6JRj and C57BL/6NRj)) and the primary background (C57BL/6J and C57BL/6NTac) and secondary background (CBA/J). This is uncommon and should be investigated further.<br>• This sample likely has more than 2 genetic backgrounds (unexplained regions and/or fractured ideogram). The strain selected for secondary background may be incorrect. The estimation of the contribution of primary and secondary background are likely incorrect. This can potentially be addressed with input from the user.
**Genotyping Quality**	**Excellent (10 N calls)**<br>All reported results are dependent on genotyping quality.
**Chromosomal Sex**	XX
**Inbreeding Estimate**	67.1% Inbred<br>(Percentage of the genome (autosomal and X chromosomes) that is homozygous or hemizygous for primary, secondary, and unknown backgrounds. See Genome Analysis)

Constructs Detected

BlastR	bpA	Cas9	chlor	cHS4	Cre	DTA	Flp	g_FP	hCMV_a	hCMV_b	hTK_pr	iCre	IRES	Luc	r_FP	rtTA	SV40	tTA
-	-	-	-	-	-	-	-	-	-	-	-	-	-	-	-	-	-	-

MiniMUGA Background Analysis v2.3.1

Refined Ideogram

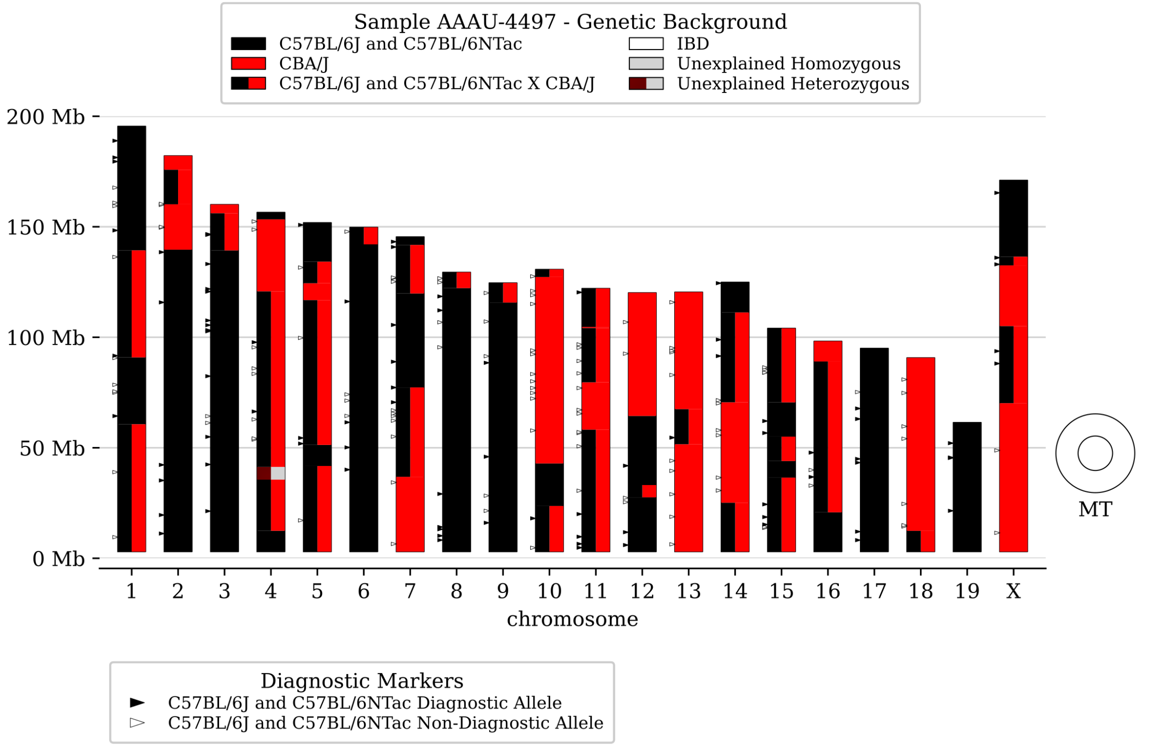

Genome Analysis

Background	Zygosity	Informative Markers	Informative Markers %	Genome %
C57BL/6J and C57BL/6NTac	N/A	1174	44.1%	44.0%
CBA/J	Homozygous	614	23.0%	23.2%
C57BL/6J and C57BL/6NTac X CBA/J	Heterozygous	864	32.4%	32.7%
Unexplained	Heterozygous	12	0.5%	0.2%
	**Total**	2664	100.0%	100.1%

Y Chromosome

Not Applicable

MT Genome

MT Haplogroup 6 - 100.0% Consistent
Includes C57BL/6J, C57BL/6NTac, CBA/J and 165 other strains

MiniMUGA Background Analysis v2.3.1

Backgrounds Detected (Diagnostic Alleles)

Diagnostic Class	Diagnostic Alleles Observed: Homozygous	Heterozygous	Potential	% Observed
C57BL/6J, C57BL/6JJicTac, C57BL/6JRj	8	37	102	44.1%
C57BL/6J, C57BL/6JEiJ, C57BL/6JJicTac, C57BL/6JRj	2	11	21	61.9%
C57BL/6NRj, C57BL/6NTac	4	5	15	60.0%
C57BL/6J, C57BL/6JRj	4	4	31	25.8%
C57BL/6NJ, C57BL/6NRj, C57BL/6NTac	4	1	10	50.0%
C57BL/6NCrl, C57BL/6NHsd, C57BL/6NJ, C57BL/6NRj, C57BL/6NTac	0	2	2	100.0%
B6N-Tyr<c-Brd>/BrdCrCrl, C57BL/6J, C57BL/6JEiJ, C57BL/6JJicTac, C57BL/6JRj	0	1	1	100.0%
B6N-Tyr<c-Brd>/BrdCrCrl, C57BL/6J, C57BL/6JJicTac, C57BL/6JRj	0	1	5	20.0%
B6N-Tyr<c-Brd>/BrdCrCrl, C57BL/6NCrl, C57BL/6NHsd, C57BL/6NJ, C57BL/6NRj, C57BL/6NTac	0	1	2	50.0%
C57BL/6NHsd, C57BL/6NJ, C57BL/6NRj, C57BL/6NTac	0	1	1	100.0%
C57BL/6NRj	0	1	10	10.0%

Minimal Strain Sets Explaining All Diagnostic Classes (Number of Markers Explained):

- Solution 1: C57BL/6J and C57BL/6NRj
  - C57BL/6J: 68 / 160 (42.5%)
  - C57BL/6NRj: 19 / 40 (47.5%)
- Solution 2: C57BL/6JRj and C57BL/6NRj
  - C57BL/6JRj: 68 / 160 (42.5%)
  - C57BL/6NRj: 19 / 40 (47.5%)

Chromosome	Start (Mb)	Stop (Mb)	Background	Zygosity
1	3000000	60621237	C57BL/6J and C57BL/6NTac and CBA/J	Heterozygous
1	60621237	90903197	C57BL/6J and C57BL/6NTac	N/A
1	90903197	139287790	C57BL/6J and C57BL/6NTac and CBA/J	Heterozygous
1	139287790	195471971	C57BL/6J and C57BL/6NTac	N/A
2	3000000	139631657	C57BL/6J and C57BL/6NTac	N/A
2	139631657	160174252	CBA/J	Homozygous
2	160174252	175780822	C57BL/6J and C57BL/6NTac and CBA/J	Heterozygous
2	175780822	182113224	CBA/J	Homozygous
3	3000000	139297311	C57BL/6J and C57BL/6NTac	N/A
3	139297311	156090101	C57BL/6J and C57BL/6NTac and CBA/J	Heterozygous
3	156090101	160039680	CBA/J	Homozygous
4	3000000	12446904	C57BL/6J and C57BL/6NTac	N/A
4	12446904	35563307	C57BL/6J and C57BL/6NTac and CBA/J	Heterozygous
4	35563307	41348396	Unexplained	Heterozygous

Diplotype Intervals

4	41348396	120738488	C57BL/6J and C57BL/6NTac and CBA/J	Heterozygous
4	120738488	153356388	CBA/J	Homozygous
4	153356388	156508116	C57BL/6J and C57BL/6NTac	N/A
5	3000000	41755530	C57BL/6J and C57BL/6NTac and CBA/J	Heterozygous
5	41755530	51299144	C57BL/6J and C57BL/6NTac	N/A
5	51299144	116795433	C57BL/6J and C57BL/6NTac and CBA/J	Heterozygous
5	116795433	124446826	CBA/J	Homozygous
5	124446826	134172373	C57BL/6J and C57BL/6NTac and CBA/J	Heterozygous
5	134172373	151834684	C57BL/6J and C57BL/6NTac	N/A
6	3000000	142043514	C57BL/6J and C57BL/6NTac	N/A
6	142043514	149736546	C57BL/6J and C57BL/6NTac and CBA/J	Heterozygous
7	3000000	36856023	CBA/J	Homozygous
7	36856023	77291515	C57BL/6J and C57BL/6NTac and CBA/J	Heterozygous
7	77291515	119823617	C57BL/6J and C57BL/6NTac	N/A
7	119823617	141750158	C57BL/6J and C57BL/6NTac and CBA/J	Heterozygous
7	141750158	145441459	C57BL/6J and C57BL/6NTac	N/A
8	3000000	122263600	C57BL/6J and C57BL/6NTac	N/A
8	122263600	129401213	C57BL/6J and C57BL/6NTac and CBA/J	Heterozygous
9	3000000	115715944	C57BL/6J and C57BL/6NTac	N/A
9	115715944	124595110	C57BL/6J and C57BL/6NTac and CBA/J	Heterozygous
10	3000000	23654421	C57BL/6J and C57BL/6NTac and CBA/J	Heterozygous
10	23654421	42917049	C57BL/6J and C57BL/6NTac	N/A
10	42917049	127271560	CBA/J	Homozygous
10	127271560	130694993	C57BL/6J and C57BL/6NTac and CBA/J	Heterozygous
11	3000000	58168384	C57BL/6J and C57BL/6NTac and CBA/J	Heterozygous
11	58168384	79617327	CBA/J	Homozygous
11	79617327	104154012	C57BL/6J and C57BL/6NTac and CBA/J	Heterozygous
11	104154012	104675339	CBA/J	Homozygous
11	104675339	122082543	C57BL/6J and C57BL/6NTac and CBA/J	Heterozygous
12	3000000	27585493	C57BL/6J and C57BL/6NTac	N/A
12	27585493	33130555	C57BL/6J and C57BL/6NTac and CBA/J	Heterozygous

MiniMUGA Background Analysis v2.3.1

	12	33130555	64411355	C57BL/6J and C57BL/6NTac	N/A
	12	64411355	120129022	CBA/J	Homozygous
	13	3000000	51605798	CBA/J	Homozygous
	13	51605798	67442927	C57BL/6J and C57BL/6NTac and CBA/J	Heterozygous
	13	67442927	120421639	CBA/J	Homozygous
	14	3000000	25112834	C57BL/6J and C57BL/6NTac and CBA/J	Heterozygous
	14	25112834	70580779	CBA/J	Homozygous
	14	70580779	111185375	C57BL/6J and C57BL/6NTac and CBA/J	Heterozygous
	14	111185375	124902244	C57BL/6J and C57BL/6NTac	N/A
	15	3000000	36473640	C57BL/6J and C57BL/6NTac and CBA/J	Heterozygous
	15	36473640	44010563	C57BL/6J and C57BL/6NTac	N/A
	15	44010563	55016741	C57BL/6J and C57BL/6NTac and CBA/J	Heterozygous
	15	55016741	70554147	C57BL/6J and C57BL/6NTac	N/A
	15	70554147	104043685	C57BL/6J and C57BL/6NTac and CBA/J	Heterozygous
	16	3000000	20813513	C57BL/6J and C57BL/6NTac	N/A
	16	20813513	89037512	C57BL/6J and C57BL/6NTac and CBA/J	Heterozygous
	16	89037512	98207768	CBA/J	Homozygous
	17	3000000	94987271	C57BL/6J and C57BL/6NTac	N/A
	18	3000000	12406382	C57BL/6J and C57BL/6NTac and CBA/J	Heterozygous
	18	12406382	90702639	CBA/J	Homozygous
	19	3000000	61431566	C57BL/6J and C57BL/6NTac	N/A
	X	3000000	70193631	CBA/J	Homozygous
	X	70193631	105020820	C57BL/6J and C57BL/6NTac and CBA/J	Heterozygous
	X	105020820	132528229	CBA/J	Homozygous
	X	132528229	136441962	C57BL/6J and C57BL/6NTac and CBA/J	Heterozygous
	X	136441962	171031299	C57BL/6J and C57BL/6NTac	N/A
	MT	0	0	IBD	Hemizygous
