# Supplementary material for: Deletion of 9p drives B-ALL through heterozygous inactivation of Pax5 and Cd72 in preleukemic cells
Source: JCI Insight. 2026 Feb 17;11(7):e199464. doi: 10.1172/jci.insight.199464 (PMC13134721; doi:10.1172/jci.insight.199464)
Supplement: Supplemental data set 1 [file jciinsight-11-199464-s204.zip › Strain_Genotyping/Q855-results-report.pdf]

MiniMUGA Background Analysis v2.3.1

**Sample ID**	Q855
**Neogen ID**	AAAU-5390
**Summary**	The genotype of this sample is of **excellent** quality. It is **female** and **outbred**, and likely a mix of **C57BL/6J and C57BL/6NRj** and **CBA/J**. Clustering of unexplained markers is evidence of an additional background strain.<br><br>Diagnostic SNPs are likely explained by the presence of the background strains<br>• Solution 1: 129S5/SvEvBrd and C57BL/6J and C57BL/6NRj<br>◦ C57BL/6J: 56 / 155 (36.1%)<br>◦ C57BL/6NRj: 18 / 40 (45.0%)<br>◦ 129S5/SvEvBrd: 1 / 5 (20.0%)<br>• Solution 2: 129S5/SvEvBrd and C57BL/6JRj and C57BL/6NRj<br>◦ C57BL/6JRj: 56 / 155 (36.1%)<br>◦ C57BL/6NRj: 18 / 40 (45.0%)<br>◦ 129S5/SvEvBrd: 1 / 5 (20.0%)<br><br>NOTE: There is a discrepancy between the diagnostic backgrounds detected and the primary and secondary background analysis (C57BL/6J, CBA/J, C57BL/6NRj). This is uncommon and should be investigated further.<br><br>No genetic constructs were detected in this sample.<br><br>WARNING:<br>• There is a discrepancy between the diagnostic backgrounds detected ((129S5/SvEvBrd and C57BL/6J and C57BL/6NRj) or (129S5/SvEvBrd and C57BL/6JRj and C57BL/6NRj)) and the primary background (C57BL/6J and C57BL/6NRj) and secondary background (CBA/J). This is uncommon and should be investigated further.<br>• The presence of a single diagnostic heterozygous call for a single inbred strain should be treated with caution.<br>• This sample likely has more than 2 genetic backgrounds (unexplained regions and/or fractured ideogram). The strain selected for secondary background may be incorrect. The estimation of the contribution of primary and secondary background are likely incorrect. This can potentially be addressed with input from the user.
**Genotyping Quality**	**Excellent (1 N calls)**<br>All reported results are dependent on genotyping quality.
**Chromosomal Sex**	XX
**Inbreeding Estimate**	69.2% Inbred<br>(Percentage of the genome (autosomal and X chromosomes) that is homozygous or hemizygous for primary, secondary, and unknown backgrounds. See Genome Analysis)

Constructs Detected

BlastR	bpA	Cas9	chlor	cHS4	Cre	DTA	Flp	g_FP	hCMV_a	hCMV_b	hTK_pr	iCre	IRES	Luc	r_FP	rtTA	SV40	tTA
-	-	-	-	-	-	-	-	-	-	-	-	-	-	-	-	-	-	-

MiniMUGA Background Analysis v2.3.1

Refined Ideogram

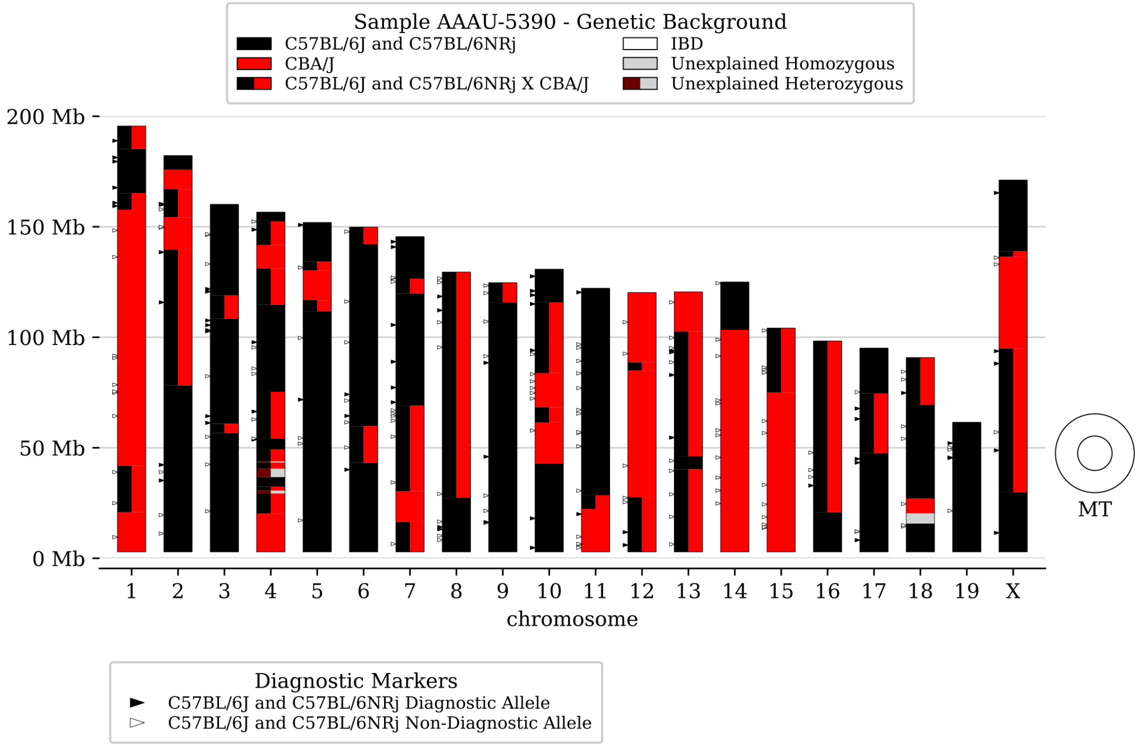

Genome Analysis

Background	Zygosity	Informative Markers	Informative Markers %	Genome %
C57BL/6J and C57BL/6NRj	N/A	1252	47.0%	46.0%
CBA/J	Homozygous	570	21.4%	23.0%
C57BL/6J and C57BL/6NRj X CBA/J	Heterozygous	828	31.1%	30.6%
Unexplained	Homozygous	6	0.2%	0.2%
Unexplained	Heterozygous	10	0.4%	0.2%
	**Total**	2666	100.1%	100.0%

Y Chromosome

Not Applicable

MT Genome

MT Haplogroup 6 - 100.0% Consistent
Includes C57BL/6J, C57BL/6NRj, CBA/J and 165 other strains

MiniMUGA Background Analysis v2.3.1

Backgrounds Detected (Diagnostic Alleles)

Diagnostic Class	Diagnostic Alleles Observed: Homozygous	Heterozygous	Potential	% Observed
C57BL/6J, C57BL/6JJicTac, C57BL/6JRj	8	32	102	39.2%
C57BL/6J, C57BL/6JEiJ, C57BL/6JJicTac, C57BL/6JRj	4	4	21	38.1%
C57BL/6J, C57BL/6JRj	2	5	31	22.6%
C57BL/6NRj, C57BL/6NTac	1	7	15	53.3%
C57BL/6NJ, C57BL/6NRj, C57BL/6NTac	2	2	10	40.0%
B6N-Tyr<c-Brd>/BrdCrCrl, C57BL/6NCrl, C57BL/6NHsd, C57BL/6NJ, C57BL/6NRj, C57BL/6NTac	1	0	2	50.0%
C57BL/6NCrl, C57BL/6NHsd, C57BL/6NJ, C57BL/6NRj, C57BL/6NTac	0	2	2	100.0%
C57BL/6NRj	0	2	10	20.0%
129S5/SvEvBrd	0	1	5	20.0%
B6N-Tyr<c-Brd>/BrdCrCrl, C57BL/6J, C57BL/6JEiJ, C57BL/6JJicTac, C57BL/6JRj	0	1	1	100.0%
C57BL/6NHsd, C57BL/6NJ, C57BL/6NRj, C57BL/6NTac	0	1	1	100.0%

Minimal Strain Sets Explaining All Diagnostic Classes (Number of Markers Explained):

- Solution 1: 129S5/SvEvBrd and C57BL/6J and C57BL/6NRj
  - C57BL/6J: 56 / 155 (36.1%)
  - C57BL/6NRj: 18 / 40 (45.0%)
  - 129S5/SvEvBrd: 1 / 5 (20.0%)
- Solution 2: 129S5/SvEvBrd and C57BL/6JRj and C57BL/6NRj
  - C57BL/6JRj: 56 / 155 (36.1%)
  - C57BL/6NRj: 18 / 40 (45.0%)
  - 129S5/SvEvBrd: 1 / 5 (20.0%)

Chromosome	Start (Mb)	Stop (Mb)	Background	Zygosity
1	3000000	20833151	CBA/J	Homozygous
1	20833151	41869819	C57BL/6J and C57BL/6NRj and CBA/J	Heterozygous
1	41869819	157713559	CBA/J	Homozygous
1	157713559	165183608	C57BL/6J and C57BL/6NRj and CBA/J	Heterozygous
1	165183608	185190026	C57BL/6J and C57BL/6NRj	N/A
1	185190026	195471971	C57BL/6J and C57BL/6NRj and CBA/J	Heterozygous
2	3000000	78267191	C57BL/6J and C57BL/6NRj	N/A
2	78267191	139631657	C57BL/6J and C57BL/6NRj and CBA/J	Heterozygous
2	139631657	154349372	CBA/J	Homozygous
2	154349372	166963888	C57BL/6J and C57BL/6NRj and CBA/J	Heterozygous
2	166963888	175780822	CBA/J	Homozygous
2	175780822	182113224	C57BL/6J and C57BL/6NRj	N/A
3	3000000	56655047	C57BL/6J and C57BL/6NRj	N/A

MiniMUGA Background Analysis v2.3.1

**Diplotype Intervals**	3	56655047	60850190	C57BL/6J and C57BL/6NRj and CBA/J	Heterozygous
	3	60850190	108381941	C57BL/6J and C57BL/6NRj	N/A
	3	108381941	118919242	C57BL/6J and C57BL/6NRj and CBA/J	Heterozygous
	3	118919242	160039680	C57BL/6J and C57BL/6NRj	N/A
	4	3000000	20258658	CBA/J	Homozygous
	4	20258658	29346519	C57BL/6J and C57BL/6NRj and CBA/J	Heterozygous
	4	29346519	30650814	Unexplained	Heterozygous
	4	30650814	32327128	C57BL/6J and C57BL/6NRj and CBA/J	Heterozygous
	4	32327128	36784495	C57BL/6J and C57BL/6NRj	N/A
	4	36784495	40531709	Unexplained	Heterozygous
	4	40531709	43372387	C57BL/6J and C57BL/6NRj and CBA/J	Heterozygous
	4	43372387	43819249	Unexplained	Heterozygous
	4	43819249	49280860	C57BL/6J and C57BL/6NRj and CBA/J	Heterozygous
	4	49280860	54114833	C57BL/6J and C57BL/6NRj	N/A
	4	54114833	75318594	C57BL/6J and C57BL/6NRj and CBA/J	Heterozygous
	4	75318594	114710010	C57BL/6J and C57BL/6NRj	N/A
	4	114710010	131104093	C57BL/6J and C57BL/6NRj and CBA/J	Heterozygous
	4	131104093	141726218	CBA/J	Homozygous
	4	141726218	152440879	C57BL/6J and C57BL/6NRj and CBA/J	Heterozygous
	4	152440879	156508116	C57BL/6J and C57BL/6NRj	N/A
	5	3000000	111745102	C57BL/6J and C57BL/6NRj	N/A
	5	111745102	116795433	C57BL/6J and C57BL/6NRj and CBA/J	Heterozygous
	5	116795433	130280923	CBA/J	Homozygous
	5	130280923	134172373	C57BL/6J and C57BL/6NRj and CBA/J	Heterozygous
	5	134172373	151834684	C57BL/6J and C57BL/6NRj	N/A
	6	3000000	43184432	C57BL/6J and C57BL/6NRj	N/A
	6	43184432	59791688	C57BL/6J and C57BL/6NRj and CBA/J	Heterozygous
	6	59791688	142043514	C57BL/6J and C57BL/6NRj	N/A
	6	142043514	149736546	C57BL/6J and C57BL/6NRj and CBA/J	Heterozygous
	7	3000000	16360273	C57BL/6J and C57BL/6NRj and CBA/J	Heterozygous
	7	16360273	30335112	CBA/J	Homozygous

MiniMUGA Background Analysis v2.3.1

	7	30335112	69096424	C57BL/6J and C57BL/6NRj and CBA/J	Heterozygous
	7	69096424	119823617	C57BL/6J and C57BL/6NRj	N/A
	7	119823617	126580094	C57BL/6J and C57BL/6NRj and CBA/J	Heterozygous
	7	126580094	145441459	C57BL/6J and C57BL/6NRj	N/A
	8	3000000	27348459	C57BL/6J and C57BL/6NRj	N/A
	8	27348459	129401213	C57BL/6J and C57BL/6NRj and CBA/J	Heterozygous
	9	3000000	115715944	C57BL/6J and C57BL/6NRj	N/A
	9	115715944	124595110	C57BL/6J and C57BL/6NRj and CBA/J	Heterozygous
	10	3000000	42858234	C57BL/6J and C57BL/6NRj	N/A
	10	42858234	61450853	CBA/J	Homozygous
	10	61450853	68332199	C57BL/6J and C57BL/6NRj and CBA/J	Heterozygous
	10	68332199	83779430	CBA/J	Homozygous
	10	83779430	115781736	C57BL/6J and C57BL/6NRj and CBA/J	Heterozygous
	10	115781736	130694993	C57BL/6J and C57BL/6NRj	N/A
	11	3000000	22302070	CBA/J	Homozygous
	11	22302070	28525615	C57BL/6J and C57BL/6NRj and CBA/J	Heterozygous
	11	28525615	122082543	C57BL/6J and C57BL/6NRj	N/A
	12	3000000	27585493	C57BL/6J and C57BL/6NRj and CBA/J	Heterozygous
	12	27585493	85015902	CBA/J	Homozygous
	12	85015902	88650858	C57BL/6J and C57BL/6NRj and CBA/J	Heterozygous
	12	88650858	120129022	CBA/J	Homozygous
	13	3000000	40278277	C57BL/6J and C57BL/6NRj and CBA/J	Heterozygous
	13	40278277	46136691	C57BL/6J and C57BL/6NRj	N/A
	13	46136691	102595519	C57BL/6J and C57BL/6NRj and CBA/J	Heterozygous
	13	102595519	120421639	CBA/J	Homozygous
	14	3000000	103377147	CBA/J	Homozygous
	14	103377147	124902244	C57BL/6J and C57BL/6NRj	N/A
	15	3000000	74996398	CBA/J	Homozygous
	15	74996398	104043685	C57BL/6J and C57BL/6NRj and CBA/J	Heterozygous
	16	3000000	20813513	C57BL/6J and C57BL/6NRj	N/A
	16	20813513	98207768	C57BL/6J and C57BL/6NRj and CBA/J	Heterozygous
	17	3000000	47545390	C57BL/6J and C57BL/6NRj	N/A

MiniMUGA Background Analysis v2.3.1

	17	47545390	74502727	C57BL/6J and C57BL/6NRj and CBA/J	Heterozygous
	17	74502727	94987271	C57BL/6J and C57BL/6NRj	N/A
	18	3000000	15685654	C57BL/6J and C57BL/6NRj	N/A
	18	15685654	20363699	Unexplained	Homozygous
	18	20363699	27036500	CBA/J	Homozygous
	18	27036500	69337106	C57BL/6J and C57BL/6NRj	N/A
	18	69337106	90702639	C57BL/6J and C57BL/6NRj and CBA/J	Heterozygous
	19	3000000	61431566	C57BL/6J and C57BL/6NRj	N/A
	X	3000000	29836043	C57BL/6J and C57BL/6NRj	N/A
	X	29836043	94918419	C57BL/6J and C57BL/6NRj and CBA/J	Heterozygous
	X	94918419	136441962	CBA/J	Homozygous
	X	136441962	138881041	C57BL/6J and C57BL/6NRj and CBA/J	Heterozygous
	X	138881041	171031299	C57BL/6J and C57BL/6NRj	N/A
	MT	0	0	IBD	Hemizygous
