# Supplementary material for: Deletion of 9p drives B-ALL through heterozygous inactivation of Pax5 and Cd72 in preleukemic cells
Source: JCI Insight. 2026 Feb 17;11(7):e199464. doi: 10.1172/jci.insight.199464 (PMC13134721; doi:10.1172/jci.insight.199464)
Supplement: Supplemental data set 1 [file jciinsight-11-199464-s204.zip › Strain_Genotyping/Q930-results-report.pdf]

MiniMUGA Background Analysis v2.3.1

<table>
<tr><td>Sample ID</td><td>Q930</td></tr>
<tr><td>Neogen ID</td><td>AAAU-4532</td></tr>
<tr><td>Summary</td><td>The genotype of this sample is of good quality. Errors in sample background determination are more frequent with this level of genotyping quality, particularly in samples from standard commercial inbred strains. Please consult the Report Guide and Neogen if reported results are unexpected.

It is female and outbred, and likely a mix of C57BL/6J and C57BL/6NRj and CBA/J. Clustering of unexplained markers is evidence of an additional background strain.

Diagnostic SNPs are likely explained by the presence of the background strains
• Solution 1: 129S5/SvEvBrd and C57BL/6J and C57BL/6NRj
◦ C57BL/6J: 60 / 162 (37.0%)
◦ C57BL/6NRj: 22 / 40 (55.0%)
◦ 129S5/SvEvBrd: 1 / 5 (20.0%)
• Solution 2: 129S5/SvEvBrd and C57BL/6JRj and C57BL/6NRj
◦ C57BL/6JRj: 60 / 162 (37.0%)
◦ C57BL/6NRj: 22 / 40 (55.0%)
◦ 129S5/SvEvBrd: 1 / 5 (20.0%)

NOTE: There is a discrepancy between the diagnostic backgrounds detected and the primary and secondary background analysis (C57BL/6NRj, CBA/J, C57BL/6J). This is uncommon and should be investigated further.

No genetic constructs were detected in this sample.

WARNING:
• There is a discrepancy between the diagnostic backgrounds detected ((129S5/SvEvBrd and C57BL/6J and C57BL/6NRj) or (129S5/SvEvBrd and C57BL/6JRj and C57BL/6NRj)) and the primary background (C57BL/6J and C57BL/6NRj) and secondary background (CBA/J). This is uncommon and should be investigated further.
• The presence of a single diagnostic heterozygous call for a single inbred strain should be treated with caution.
• This sample likely has more than 2 genetic backgrounds (unexplained regions and/or fractured ideogram). The strain selected for secondary background may be incorrect. The estimation of the contribution of primary and secondary background are likely incorrect. This can potentially be addressed with input from the user.</td></tr>
<tr><td>Genotyping Quality</td><td>Good (107 N calls)
All reported results are dependent on genotyping quality.</td></tr>
<tr><td>Chromosomal Sex</td><td>XX</td></tr>
<tr><td>Inbreeding Estimate</td><td>66.2% Inbred
(Percentage of the genome (autosomal and X chromosomes) that is homozygous or hemizygous for primary, secondary, and unknown backgrounds. See Genome Analysis)</td></tr>
<tr><td>Constructs Detected</td><td>

BlastR	bpA	Cas9	chlor	cHS4	Cre	DTA	Flp	g_FP	hCMV_a	hCMV_b	hTK_pr	iCre	IRES	Luc	r_FP	rtTA	SV40	tTA
-	-	-	-	-	-	-	-	-	-	-	-	-	-	-	-	-	-	-

</td></tr>
</table>

MiniMUGA Background Analysis v2.3.1

Refined Ideogram

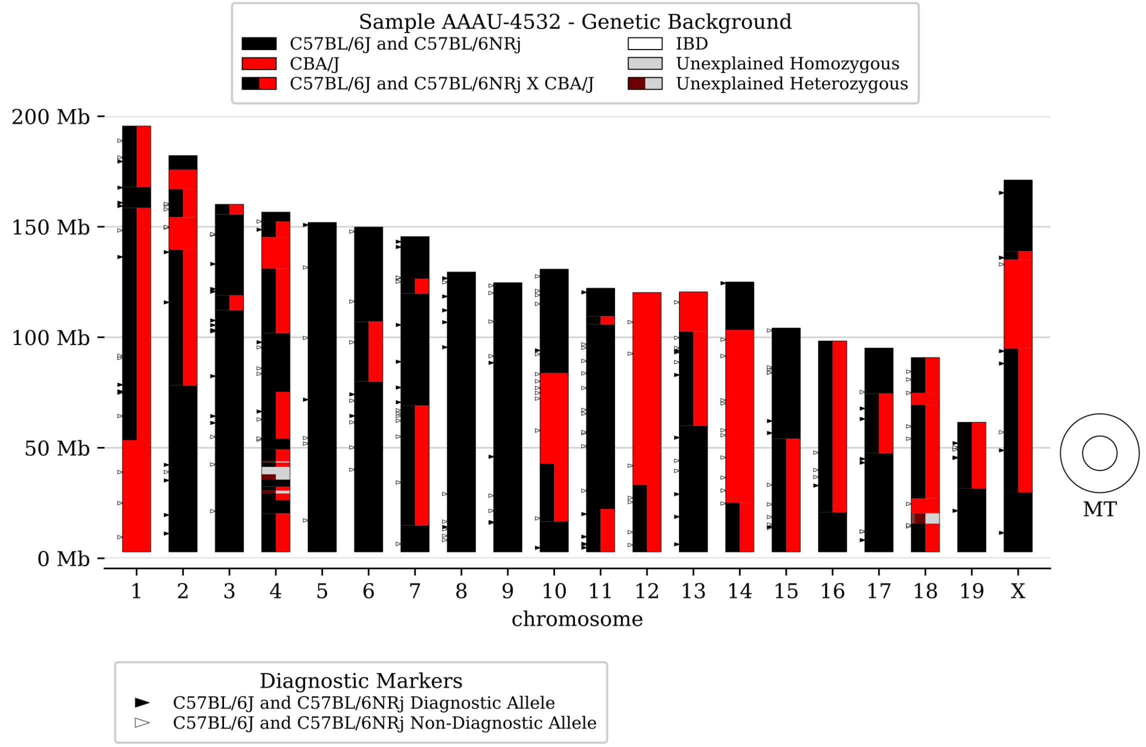

Genome Analysis

Background	Zygosity	Informative Markers	Informative Markers %	Genome %
C57BL/6J and C57BL/6NRj	N/A	1292	50.1%	51.9%
CBA/J	Homozygous	358	13.9%	14.2%
C57BL/6J and C57BL/6NRj X CBA/J	Heterozygous	906	35.2%	33.5%
Unexplained	Homozygous	5	0.2%	0.1%
Unexplained	Heterozygous	16	0.6%	0.3%
	**Total**	2577	100.0%	100.0%

Y Chromosome

Not Applicable

MT Genome

MT Haplogroup 6 - 100.0% Consistent
Includes C57BL/6J, C57BL/6NRj, CBA/J and 165 other strains

MiniMUGA Background Analysis v2.3.1

Backgrounds Detected (Diagnostic Alleles)

Diagnostic Class	Diagnostic Alleles Observed Homozygous	Heterozygous	Potential	% Observed
C57BL/6J, C57BL/6JJicTac, C57BL/6JRj	3	36	102	38.2%
C57BL/6J, C57BL/6JEiJ, C57BL/6JJicTac, C57BL/6JRj	6	5	21	52.4%
C57BL/6NRj, C57BL/6NTac	6	5	15	73.3%
C57BL/6NJ, C57BL/6NRj, C57BL/6NTac	3	2	10	50.0%
C57BL/6J, C57BL/6JRj	0	7	31	22.6%
B6N-Tyr<c-Brd>/BrdCrCrl, C57BL/6NCrl, C57BL/6NHsd, C57BL/6NJ, C57BL/6NRj, C57BL/6NTac	1	0	2	50.0%
C57BL/6NCrl, C57BL/6NHsd, C57BL/6NJ, C57BL/6NRj, C57BL/6NTac	0	2	2	100.0%
C57BL/6NRj	0	2	10	20.0%
129S5/SvEvBrd	0	1	5	20.0%
B6N-Tyr<c-Brd>/BrdCrCrl, C57BL/6J, C57BL/6JEiJ, C57BL/6JJicTac, C57BL/6JRj	0	1	1	100.0%
B6N-Tyr<c-Brd>/BrdCrCrl, C57BL/6J, C57BL/6JJicTac, C57BL/6JRj	0	1	5	20.0%
C57BL/6J, C57BL/6JBomTac, C57BL/6JEiJ, C57BL/6JJicTac, C57BL/6JOlaHsd, C57BL/6JRj	0	1	2	50.0%
C57BL/6NHsd, C57BL/6NJ, C57BL/6NRj, C57BL/6NTac	0	1	1	100.0%

Minimal Strain Sets Explaining All Diagnostic Classes (Number of Markers Explained):

- Solution 1: 129S5/SvEvBrd and C57BL/6J and C57BL/6NRj
    - C57BL/6J: 60 / 162 (37.0%)
    - C57BL/6NRj: 22 / 40 (55.0%)
    - 129S5/SvEvBrd: 1 / 5 (20.0%)
- Solution 2: 129S5/SvEvBrd and C57BL/6JRj and C57BL/6NRj
    - C57BL/6JRj: 60 / 162 (37.0%)
    - C57BL/6NRj: 22 / 40 (55.0%)
    - 129S5/SvEvBrd: 1 / 5 (20.0%)

Chromosome	Start (Mb)	Stop (Mb)	Background	Zygosity
1	3000000	53457225	CBA/J	Homozygous
1	53457225	158479371	C57BL/6J and C57BL/6NRj and CBA/J	Heterozygous
1	158479371	168019536	C57BL/6J and C57BL/6NRj	N/A
1	168019536	195471971	C57BL/6J and C57BL/6NRj and CBA/J	Heterozygous
2	3000000	78267191	C57BL/6J and C57BL/6NRj	N/A
2	78267191	139631657	C57BL/6J and C57BL/6NRj and CBA/J	Heterozygous
2	139631657	154349372	CBA/J	Homozygous
2	154349372	166963888	C57BL/6J and C57BL/6NRj and CBA/J	Heterozygous
2	166963888	175780822	CBA/J	Homozygous
2	175780822	182113224	C57BL/6J and C57BL/6NRj	N/A
3	3000000	112280452	C57BL/6J and C57BL/6NRj	N/A

MiniMUGA Background Analysis v2.3.1

**Diplotype Intervals**	3	112280452	118919242	C57BL/6J and C57BL/6NRj and CBA/J	Heterozygous
	3	118919242	155643965	C57BL/6J and C57BL/6NRj	N/A
	3	155643965	160039680	C57BL/6J and C57BL/6NRj and CBA/J	Heterozygous
	4	3000000	20258658	C57BL/6J and C57BL/6NRj and CBA/J	Heterozygous
	4	20258658	26280383	C57BL/6J and C57BL/6NRj	N/A
	4	26280383	29346519	C57BL/6J and C57BL/6NRj and CBA/J	Heterozygous
	4	29346519	30650814	Unexplained	Heterozygous
	4	30650814	32327128	C57BL/6J and C57BL/6NRj and CBA/J	Heterozygous
	4	32327128	35563307	C57BL/6J and C57BL/6NRj	N/A
	4	35563307	37995481	Unexplained	Heterozygous
	4	37995481	41348396	Unexplained	Homozygous
	4	41348396	43372387	C57BL/6J and C57BL/6NRj and CBA/J	Heterozygous
	4	43372387	43819249	Unexplained	Heterozygous
	4	43819249	49280860	C57BL/6J and C57BL/6NRj and CBA/J	Heterozygous
	4	49280860	54114833	C57BL/6J and C57BL/6NRj	N/A
	4	54114833	75318594	C57BL/6J and C57BL/6NRj and CBA/J	Heterozygous
	4	75318594	101914190	C57BL/6J and C57BL/6NRj	N/A
	4	101914190	131104093	C57BL/6J and C57BL/6NRj and CBA/J	Heterozygous
	4	131104093	145315418	CBA/J	Homozygous
	4	145315418	152440879	C57BL/6J and C57BL/6NRj and CBA/J	Heterozygous
	4	152440879	156508116	C57BL/6J and C57BL/6NRj	N/A
	5	3000000	151834684	C57BL/6J and C57BL/6NRj	N/A
	6	3000000	80057017	C57BL/6J and C57BL/6NRj	N/A
	6	80057017	107054750	C57BL/6J and C57BL/6NRj and CBA/J	Heterozygous
	6	107054750	149736546	C57BL/6J and C57BL/6NRj	N/A
	7	3000000	14936147	C57BL/6J and C57BL/6NRj	N/A
	7	14936147	69096424	C57BL/6J and C57BL/6NRj and CBA/J	Heterozygous
	7	69096424	119823617	C57BL/6J and C57BL/6NRj	N/A
	7	119823617	126580094	C57BL/6J and C57BL/6NRj and CBA/J	Heterozygous
	7	126580094	145441459	C57BL/6J and C57BL/6NRj	N/A
	8	3000000	129401213	C57BL/6J and C57BL/6NRj	N/A

MiniMUGA Background Analysis v2.3.1

	9	3000000	124595110	C57BL/6J and C57BL/6NRj	N/A
	10	3000000	16704298	C57BL/6J and C57BL/6NRj	N/A
	10	16704298	42858234	C57BL/6J and C57BL/6NRj and CBA/J	Heterozygous
	10	42858234	83779430	CBA/J	Homozygous
	10	83779430	130694993	C57BL/6J and C57BL/6NRj	N/A
	11	3000000	22302070	C57BL/6J and C57BL/6NRj and CBA/J	Heterozygous
	11	22302070	105886229	C57BL/6J and C57BL/6NRj	N/A
	11	105886229	109459854	C57BL/6J and C57BL/6NRj and CBA/J	Heterozygous
	11	109459854	122082543	C57BL/6J and C57BL/6NRj	N/A
	12	3000000	33130555	C57BL/6J and C57BL/6NRj and CBA/J	Heterozygous
	12	33130555	120129022	CBA/J	Homozygous
	13	3000000	60016573	C57BL/6J and C57BL/6NRj	N/A
	13	60016573	102595519	C57BL/6J and C57BL/6NRj and CBA/J	Heterozygous
	13	102595519	120421639	CBA/J	Homozygous
	14	3000000	25112834	C57BL/6J and C57BL/6NRj and CBA/J	Heterozygous
	14	25112834	103377147	CBA/J	Homozygous
	14	103377147	124902244	C57BL/6J and C57BL/6NRj	N/A
	15	3000000	54017167	C57BL/6J and C57BL/6NRj and CBA/J	Heterozygous
	15	54017167	104043685	C57BL/6J and C57BL/6NRj	N/A
	16	3000000	20813513	C57BL/6J and C57BL/6NRj	N/A
	16	20813513	98207768	C57BL/6J and C57BL/6NRj and CBA/J	Heterozygous
	17	3000000	47545390	C57BL/6J and C57BL/6NRj	N/A
	17	47545390	74502727	C57BL/6J and C57BL/6NRj and CBA/J	Heterozygous
	17	74502727	94987271	C57BL/6J and C57BL/6NRj	N/A
	18	3000000	15685654	C57BL/6J and C57BL/6NRj and CBA/J	Heterozygous
	18	15685654	20363699	Unexplained	Heterozygous
	18	20363699	27036500	CBA/J	Homozygous
	18	27036500	69337106	C57BL/6J and C57BL/6NRj and CBA/J	Heterozygous
	18	69337106	74900603	CBA/J	Homozygous
	18	74900603	90702639	C57BL/6J and C57BL/6NRj and CBA/J	Heterozygous
	19	3000000	31636352	C57BL/6J and C57BL/6NRj	N/A

MiniMUGA Background Analysis v2.3.1

	19	31636352	61431566	C57BL/6J and C57BL/6NRj and CBA/J	Heterozygous
	X	3000000	29836043	C57BL/6J and C57BL/6NRj	N/A
	X	29836043	94918419	C57BL/6J and C57BL/6NRj and CBA/J	Heterozygous
	X	94918419	135099309	CBA/J	Homozygous
	X	135099309	138881041	C57BL/6J and C57BL/6NRj and CBA/J	Heterozygous
	X	138881041	171031299	C57BL/6J and C57BL/6NRj	N/A
	MT	0	0	IBD	Hemizygous
