# Supplementary material for: Deletion of 9p drives B-ALL through heterozygous inactivation of Pax5 and Cd72 in preleukemic cells
Source: JCI Insight. 2026 Feb 17;11(7):e199464. doi: 10.1172/jci.insight.199464 (PMC13134721; doi:10.1172/jci.insight.199464)
Supplement: Supplemental data set 1 [file jciinsight-11-199464-s204.zip › Strain_Genotyping/Q931-results-report.pdf]

MiniMUGA Background Analysis v2.3.1

<table>
<tr><th>Sample ID</th><td>Q931</td></tr>
<tr><th>Neogen ID</th><td>AAAU-4533</td></tr>
<tr><th>Summary</th><td>The genotype of this sample is of excellent quality. It is female and outbred, and likely a mix of C57BL/6J and C57BL/6NRj and CBA/J. Clustering of unexplained markers is evidence of an additional background strain.

Diagnostic SNPs are likely explained by the presence of the background strains

Solution 1: 129S5/SvEvBrd and C57BL/6J and C57BL/6NRj
C57BL/6J: 49 / 160 (30.6%)
C57BL/6NRj: 20 / 39 (51.3%)
129S5/SvEvBrd: 1 / 5 (20.0%)
Solution 2: 129S5/SvEvBrd and C57BL/6JRj and C57BL/6NRj
C57BL/6JRj: 49 / 160 (30.6%)
C57BL/6NRj: 20 / 39 (51.3%)
129S5/SvEvBrd: 1 / 5 (20.0%)

NOTE: There is a discrepancy between the diagnostic backgrounds detected and the primary and secondary background analysis (C57BL/6NRj, CBA/J, C57BL/6J). This is uncommon and should be investigated further.

No genetic constructs were detected in this sample.

WARNING:

There is a discrepancy between the diagnostic backgrounds detected ((129S5/SvEvBrd and C57BL/6J and C57BL/6NRj) or (129S5/SvEvBrd and C57BL/6JRj and C57BL/6NRj)) and the primary background (C57BL/6J and C57BL/6NRj) and secondary background (CBA/J). This is uncommon and should be investigated further.
The presence of a single diagnostic heterozygous call for a single inbred strain should be treated with caution.
This sample likely has more than 2 genetic backgrounds (unexplained regions and/or fractured ideogram). The strain selected for secondary background may be incorrect. The estimation of the contribution of primary and secondary background are likely incorrect. This can potentially be addressed with input from the user.
</td></tr>
<tr><th>Genotyping Quality</th><td>Excellent (6 N calls)
All reported results are dependent on genotyping quality.</td></tr>
<tr><th>Chromosomal Sex</th><td>XX</td></tr>
<tr><th>Inbreeding Estimate</th><td>63.9% Inbred
(Percentage of the genome (autosomal and X chromosomes) that is homozygous or hemizygous for primary, secondary, and unknown backgrounds. See Genome Analysis)</td></tr>
<tr><th>Constructs Detected</th><td>
<table>
<tr><th>BlastR</th><th>bpA</th><th>Cas9</th><th>chlor</th><th>cHS4</th><th>Cre</th><th>DTA</th><th>Flp</th><th>g_FP</th><th>hCMV_a</th><th>hCMV_b</th><th>hTK_pr</th><th>iCre</th><th>IRES</th><th>Luc</th><th>r_FP</th><th>rtTA</th><th>SV40</th><th>tTA</th></tr>
<tr><td>-</td><td>-</td><td>-</td><td>-</td><td>-</td><td>-</td><td>-</td><td>-</td><td>-</td><td>-</td><td>-</td><td>-</td><td>-</td><td>-</td><td>-</td><td>-</td><td>-</td><td>-</td><td>-</td></tr>
</table>
</td></tr>
</table>

MiniMUGA Background Analysis v2.3.1

**Refined Ideogram**	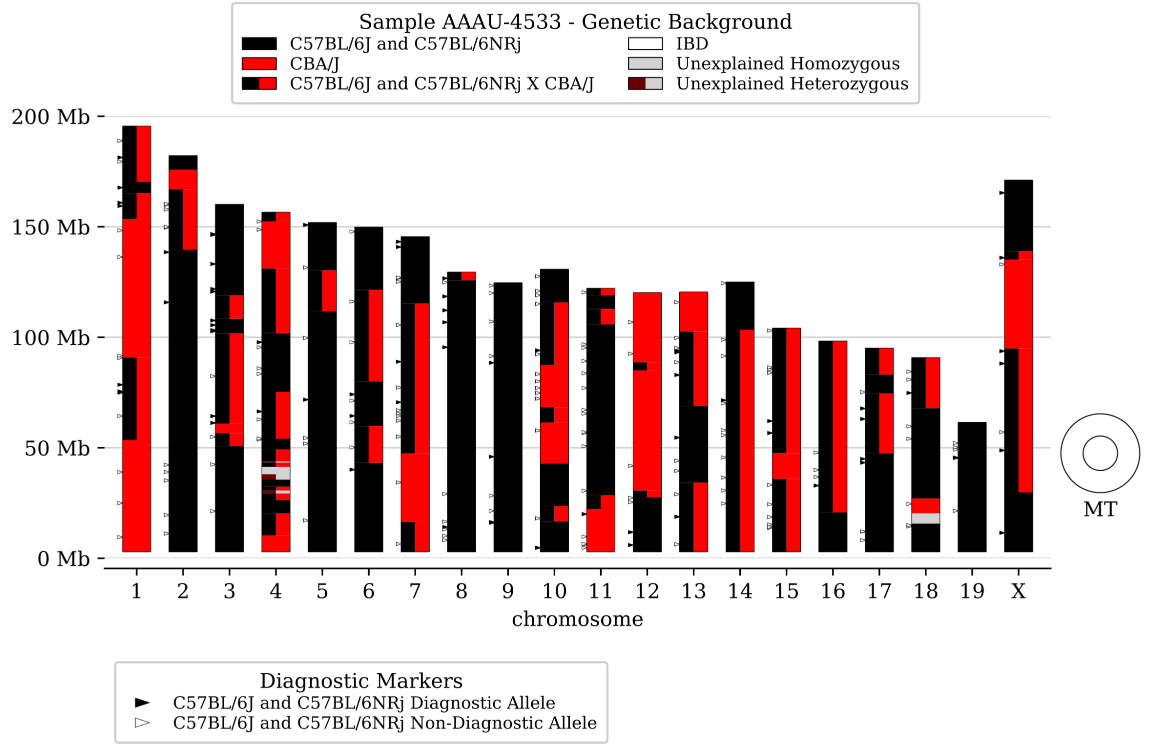

Genome Analysis

Background	Zygosity	Informative Markers	Informative Markers %	Genome %
C57BL/6J and C57BL/6NRj	N/A	1285	48.2%	47.9%
CBA/J	Homozygous	426	16.0%	15.7%
C57BL/6J and C57BL/6NRj X CBA/J	Heterozygous	936	35.1%	35.9%
Unexplained	Homozygous	11	0.4%	0.3%
Unexplained	Heterozygous	9	0.3%	0.2%
	**Total**	2667	100.0%	100.0%

**Y Chromosome**	Not Applicable
**MT Genome**	MT Haplogroup 6 - 100.0% Consistent<br>Includes C57BL/6J, C57BL/6NRj, CBA/J and 165 other strains

MiniMUGA Background Analysis v2.3.1

Backgrounds Detected (Diagnostic Alleles)

Diagnostic Class	Diagnostic Alleles Observed: Homozygous	Heterozygous	Potential	% Observed
C57BL/6J, C57BL/6JJicTac, C57BL/6JRj	8	27	102	34.3%
C57BL/6J, C57BL/6JEiJ, C57BL/6JJicTac, C57BL/6JRj	5	2	21	33.3%
C57BL/6NRj, C57BL/6NTac	1	9	15	66.7%
C57BL/6NJ, C57BL/6NRj, C57BL/6NTac	3	2	10	50.0%
C57BL/6J, C57BL/6JRj	1	4	31	16.1%
B6N-Tyr<c-Brd>/BrdCrCrl, C57BL/6NCrl, C57BL/6NHsd, C57BL/6NJ, C57BL/6NRj, C57BL/6NTac	1	0	2	50.0%
C57BL/6NCrl, C57BL/6NHsd, C57BL/6NJ, C57BL/6NRj, C57BL/6NTac	0	2	2	100.0%
C57BL/6NRj	0	2	10	20.0%
129S5/SvEvBrd	0	1	5	20.0%
B6N-Tyr<c-Brd>/BrdCrCrl, C57BL/6J, C57BL/6JEiJ, C57BL/6JJicTac, C57BL/6JRj	0	1	1	100.0%
B6N-Tyr<c-Brd>/BrdCrCrl, C57BL/6J, C57BL/6JJicTac, C57BL/6JRj	0	1	5	20.0%

Minimal Strain Sets Explaining All Diagnostic Classes (Number of Markers Explained):

- Solution 1: 129S5/SvEvBrd and C57BL/6J and C57BL/6NRj
  - C57BL/6J: 49 / 160 (30.6%)
  - C57BL/6NRj: 20 / 39 (51.3%)
  - 129S5/SvEvBrd: 1 / 5 (20.0%)
- Solution 2: 129S5/SvEvBrd and C57BL/6JRj and C57BL/6NRj
  - C57BL/6JRj: 49 / 160 (30.6%)
  - C57BL/6NRj: 20 / 39 (51.3%)
  - 129S5/SvEvBrd: 1 / 5 (20.0%)

Chromosome	Start (Mb)	Stop (Mb)	Background	Zygosity
1	3000000	53457225	CBA/J	Homozygous
1	53457225	90903197	C57BL/6J and C57BL/6NRj and CBA/J	Heterozygous
1	90903197	153548642	CBA/J	Homozygous
1	153548642	165183608	C57BL/6J and C57BL/6NRj and CBA/J	Heterozygous
1	165183608	170316822	C57BL/6J and C57BL/6NRj	N/A
1	170316822	195471971	C57BL/6J and C57BL/6NRj and CBA/J	Heterozygous
2	3000000	139631657	C57BL/6J and C57BL/6NRj	N/A
2	139631657	166963888	C57BL/6J and C57BL/6NRj and CBA/J	Heterozygous
2	166963888	175780822	CBA/J	Homozygous
2	175780822	182113224	C57BL/6J and C57BL/6NRj	N/A
3	3000000	50769488	C57BL/6J and C57BL/6NRj	N/A
3	50769488	56655047	C57BL/6J and C57BL/6NRj and CBA/J	Heterozygous
3	56655047	60850190	CBA/J	Homozygous

MiniMUGA Background Analysis v2.3.1

**Diplotype Intervals**	3	60850190	101716043	C57BL/6J and C57BL/6NRj and CBA/J	Heterozygous
	3	101716043	108381941	C57BL/6J and C57BL/6NRj	N/A
	3	108381941	118919242	C57BL/6J and C57BL/6NRj and CBA/J	Heterozygous
	3	118919242	160039680	C57BL/6J and C57BL/6NRj	N/A
	4	3000000	10320109	CBA/J	Homozygous
	4	10320109	20258658	C57BL/6J and C57BL/6NRj and CBA/J	Heterozygous
	4	20258658	26280383	C57BL/6J and C57BL/6NRj	N/A
	4	26280383	29346519	C57BL/6J and C57BL/6NRj and CBA/J	Heterozygous
	4	29346519	30650814	Unexplained	Heterozygous
	4	30650814	32327128	C57BL/6J and C57BL/6NRj and CBA/J	Heterozygous
	4	32327128	35563307	C57BL/6J and C57BL/6NRj	N/A
	4	35563307	37995481	Unexplained	Heterozygous
	4	37995481	41348396	Unexplained	Homozygous
	4	41348396	43372387	C57BL/6J and C57BL/6NRj and CBA/J	Heterozygous
	4	43372387	43819249	Unexplained	Heterozygous
	4	43819249	49280860	C57BL/6J and C57BL/6NRj and CBA/J	Heterozygous
	4	49280860	54114833	C57BL/6J and C57BL/6NRj	N/A
	4	54114833	75318594	C57BL/6J and C57BL/6NRj and CBA/J	Heterozygous
	4	75318594	101914190	C57BL/6J and C57BL/6NRj	N/A
	4	101914190	131104093	C57BL/6J and C57BL/6NRj and CBA/J	Heterozygous
	4	131104093	152440879	CBA/J	Homozygous
	4	152440879	156508116	C57BL/6J and C57BL/6NRj and CBA/J	Heterozygous
	5	3000000	111745102	C57BL/6J and C57BL/6NRj	N/A
	5	111745102	130280923	C57BL/6J and C57BL/6NRj and CBA/J	Heterozygous
	5	130280923	151834684	C57BL/6J and C57BL/6NRj	N/A
	6	3000000	43184432	C57BL/6J and C57BL/6NRj	N/A
	6	43184432	59791688	C57BL/6J and C57BL/6NRj and CBA/J	Heterozygous
	6	59791688	80057017	C57BL/6J and C57BL/6NRj	N/A
	6	80057017	121394377	C57BL/6J and C57BL/6NRj and CBA/J	Heterozygous
	6	121394377	149736546	C57BL/6J and C57BL/6NRj	N/A
	7	3000000	16360273	C57BL/6J and C57BL/6NRj and CBA/J	Heterozygous

MiniMUGA Background Analysis v2.3.1

7	16360273	47395440	CBA/J	Homozygous
7	47395440	115227247	C57BL/6J and C57BL/6NRj and CBA/J	Heterozygous
7	115227247	145441459	C57BL/6J and C57BL/6NRj	N/A
8	3000000	125832225	C57BL/6J and C57BL/6NRj	N/A
8	125832225	129401213	C57BL/6J and C57BL/6NRj and CBA/J	Heterozygous
9	3000000	124595110	C57BL/6J and C57BL/6NRj	N/A
10	3000000	16704298	C57BL/6J and C57BL/6NRj	N/A
10	16704298	23654421	C57BL/6J and C57BL/6NRj and CBA/J	Heterozygous
10	23654421	42917049	C57BL/6J and C57BL/6NRj	N/A
10	42917049	61450853	CBA/J	Homozygous
10	61450853	68332199	C57BL/6J and C57BL/6NRj and CBA/J	Heterozygous
10	68332199	87502245	CBA/J	Homozygous
10	87502245	115781736	C57BL/6J and C57BL/6NRj and CBA/J	Heterozygous
10	115781736	130694993	C57BL/6J and C57BL/6NRj	N/A
11	3000000	22302070	CBA/J	Homozygous
11	22302070	28525615	C57BL/6J and C57BL/6NRj and CBA/J	Heterozygous
11	28525615	105886229	C57BL/6J and C57BL/6NRj	N/A
11	105886229	112771442	C57BL/6J and C57BL/6NRj and CBA/J	Heterozygous
11	112771442	119038285	C57BL/6J and C57BL/6NRj	N/A
11	119038285	122082543	C57BL/6J and C57BL/6NRj and CBA/J	Heterozygous
12	3000000	27585493	C57BL/6J and C57BL/6NRj	N/A
12	27585493	30614550	C57BL/6J and C57BL/6NRj and CBA/J	Heterozygous
12	30614550	85015902	CBA/J	Homozygous
12	85015902	88650858	C57BL/6J and C57BL/6NRj and CBA/J	Heterozygous
12	88650858	120129022	CBA/J	Homozygous
13	3000000	34155213	C57BL/6J and C57BL/6NRj and CBA/J	Heterozygous
13	34155213	68886272	C57BL/6J and C57BL/6NRj	N/A
13	68886272	102595519	C57BL/6J and C57BL/6NRj and CBA/J	Heterozygous
13	102595519	120421639	CBA/J	Homozygous
14	3000000	103377147	C57BL/6J and C57BL/6NRj and CBA/J	Heterozygous
14	103377147	124902244	C57BL/6J and C57BL/6NRj	N/A

MiniMUGA Background Analysis v2.3.1

	15	3000000	35896650	C57BL/6J and C57BL/6NRj and CBA/J	Heterozygous
	15	35896650	47626553	CBA/J	Homozygous
	15	47626553	104043685	C57BL/6J and C57BL/6NRj and CBA/J	Heterozygous
	16	3000000	20813513	C57BL/6J and C57BL/6NRj	N/A
	16	20813513	98207768	C57BL/6J and C57BL/6NRj and CBA/J	Heterozygous
	17	3000000	47545390	C57BL/6J and C57BL/6NRj	N/A
	17	47545390	74502727	C57BL/6J and C57BL/6NRj and CBA/J	Heterozygous
	17	74502727	83146268	C57BL/6J and C57BL/6NRj	N/A
	17	83146268	94987271	C57BL/6J and C57BL/6NRj and CBA/J	Heterozygous
	18	3000000	15685654	C57BL/6J and C57BL/6NRj	N/A
	18	15685654	20363699	Unexplained	Homozygous
	18	20363699	27036500	CBA/J	Homozygous
	18	27036500	67937187	C57BL/6J and C57BL/6NRj	N/A
	18	67937187	90702639	C57BL/6J and C57BL/6NRj and CBA/J	Heterozygous
	19	3000000	61431566	C57BL/6J and C57BL/6NRj	N/A
	X	3000000	29836043	C57BL/6J and C57BL/6NRj	N/A
	X	29836043	94918419	C57BL/6J and C57BL/6NRj and CBA/J	Heterozygous
	X	94918419	135099309	CBA/J	Homozygous
	X	135099309	138881041	C57BL/6J and C57BL/6NRj and CBA/J	Heterozygous
	X	138881041	171031299	C57BL/6J and C57BL/6NRj	N/A
	MT	0	0	IBD	Hemizygous
