# Supplementary material for: Deletion of 9p drives B-ALL through heterozygous inactivation of Pax5 and Cd72 in preleukemic cells
Source: JCI Insight. 2026 Feb 17;11(7):e199464. doi: 10.1172/jci.insight.199464 (PMC13134721; doi:10.1172/jci.insight.199464)
Supplement: Supplemental data set 1 [file jciinsight-11-199464-s204.zip › Strain_Genotyping/W529-results-report.pdf]

MiniMUGA Background Analysis v2.3.1

**Sample ID**	W529
**Neogen ID**	AAAU-4511
**Summary**	The genotype of this sample is of **excellent** quality. It is **female** and **outbred**, and likely a mix of **C57BL/6J and C57BL/6NTac** and **CBA/J**. Clustering of unexplained markers is evidence of an additional background strain.<br><br>Diagnostic SNPs are likely explained by the presence of the background strains<br>• Solution 1: 129S5/SvEvBrd and C57BL/6J and C57BL/6NRj<br>◦ C57BL/6J: 49 / 162 (30.2%)<br>◦ C57BL/6NRj: 24 / 40 (60.0%)<br>◦ 129S5/SvEvBrd: 1 / 5 (20.0%)<br>• Solution 2: 129S5/SvEvBrd and C57BL/6JRj and C57BL/6NRj<br>◦ C57BL/6JRj: 49 / 162 (30.2%)<br>◦ C57BL/6NRj: 24 / 40 (60.0%)<br>◦ 129S5/SvEvBrd: 1 / 5 (20.0%)<br><br>NOTE: There is a discrepancy between the diagnostic backgrounds detected and the primary and secondary background analysis (CBA/J, C57BL/6J, C57BL/6NTac). This is uncommon and should be investigated further.<br><br>No genetic constructs were detected in this sample.<br><br>WARNING:<br>• There is a discrepancy between the diagnostic backgrounds detected ((129S5/SvEvBrd and C57BL/6J and C57BL/6NRj) or (129S5/SvEvBrd and C57BL/6JRj and C57BL/6NRj)) and the primary background (C57BL/6J and C57BL/6NTac) and secondary background (CBA/J). This is uncommon and should be investigated further.<br>• The presence of a single diagnostic heterozygous call for a single inbred strain should be treated with caution.<br>• This sample likely has more than 2 genetic backgrounds (unexplained regions and/or fractured ideogram). The strain selected for secondary background may be incorrect. The estimation of the contribution of primary and secondary background are likely incorrect. This can potentially be addressed with input from the user.
**Genotyping Quality**	**Excellent (5 N calls)**<br>All reported results are dependent on genotyping quality.
**Chromosomal Sex**	XX
**Inbreeding Estimate**	61.8% Inbred<br>(Percentage of the genome (autosomal and X chromosomes) that is homozygous or hemizygous for primary, secondary, and unknown backgrounds. See Genome Analysis)

Constructs Detected

BlastR	bpA	Cas9	chlor	cHS4	Cre	DTA	Flp	g_FP	hCMV_a	hCMV_b	hTK_pr	iCre	IRES	Luc	r_FP	rtTA	SV40	tTA
-	-	-	-	-	-	-	-	-	-	-	-	-	-	-	-	-	-	-

MiniMUGA Background Analysis v2.3.1

Refined Ideogram

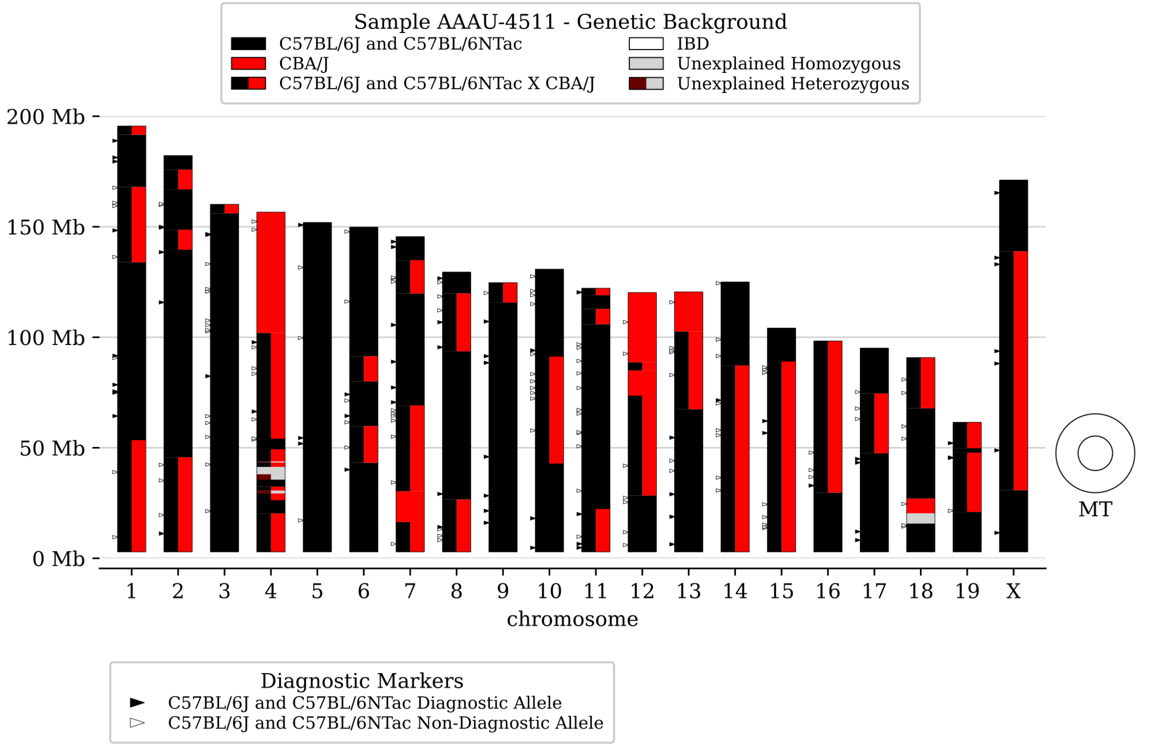

Genome Analysis

Background	Zygosity	Informative Markers	Informative Markers %	Genome %
C57BL/6J and C57BL/6NTac	N/A	1482	55.8%	56.2%
CBA/J	Homozygous	151	5.7%	5.3%
C57BL/6J and C57BL/6NTac X CBA/J	Heterozygous	1004	37.8%	38.1%
Unexplained	Homozygous	11	0.4%	0.3%
Unexplained	Heterozygous	10	0.4%	0.2%
	**Total**	2658	100.1%	100.1%

Y Chromosome

Not Applicable

MT Genome

MT Haplogroup 6 - 100.0% Consistent
Includes C57BL/6J, C57BL/6NTac, CBA/J and 165 other strains

MiniMUGA Background Analysis v2.3.1

Backgrounds Detected (Diagnostic Alleles)

	Diagnostic Alleles Observed			
**Diagnostic Class**	**Homozygous**	**Heterozygous**	**Potential**	**% Observed**
C57BL/6J, C57BL/6JJicTac, C57BL/6JRj	3	26	102	28.4%
C57BL/6NRj, C57BL/6NTac	3	8	15	73.3%
C57BL/6J, C57BL/6JEiJ, C57BL/6JJicTac, C57BL/6JRj	1	8	21	42.9%
C57BL/6NJ, C57BL/6NRj, C57BL/6NTac	2	5	10	70.0%
C57BL/6J, C57BL/6JRj	0	7	31	22.6%
B6N-Tyr<c-Brd>/BrdCrCrl, C57BL/6NCrl, C57BL/6NHsd, C57BL/6NJ, C57BL/6NRj, C57BL/6NTac	1	1	2	100.0%
B6N-Tyr<c-Brd>/BrdCrCrl, C57BL/6J, C57BL/6JJicTac, C57BL/6JRj	0	2	5	40.0%
C57BL/6NCrl, C57BL/6NHsd, C57BL/6NJ, C57BL/6NRj, C57BL/6NTac	0	2	2	100.0%
129S5/SvEvBrd	0	1	5	20.0%
B6N-Tyr<c-Brd>/BrdCrCrl, C57BL/6J, C57BL/6JEiJ, C57BL/6JJicTac, C57BL/6JRj	0	1	1	100.0%
C57BL/6J, C57BL/6JBomTac, C57BL/6JEiJ, C57BL/6JJicTac, C57BL/6JOlaHsd, C57BL/6JRj	0	1	2	50.0%
C57BL/6NHsd, C57BL/6NJ, C57BL/6NRj, C57BL/6NTac	0	1	1	100.0%
C57BL/6NRj	0	1	10	10.0%

Minimal Strain Sets Explaining All Diagnostic Classes (Number of Markers Explained):

- Solution 1: 129S5/SvEvBrd and C57BL/6J and C57BL/6NRj
  - C57BL/6J: 49 / 162 (30.2%)
  - C57BL/6NRj: 24 / 40 (60.0%)
  - 129S5/SvEvBrd: 1 / 5 (20.0%)
- Solution 2: 129S5/SvEvBrd and C57BL/6JRj and C57BL/6NRj
  - C57BL/6JRj: 49 / 162 (30.2%)
  - C57BL/6NRj: 24 / 40 (60.0%)
  - 129S5/SvEvBrd: 1 / 5 (20.0%)

Chromosome	Start (Mb)	Stop (Mb)	Background	Zygosity
1	3000000	53457225	C57BL/6J and C57BL/6NTac and CBA/J	Heterozygous
1	53457225	133964494	C57BL/6J and C57BL/6NTac	N/A
1	133964494	168019536	C57BL/6J and C57BL/6NTac and CBA/J	Heterozygous
1	168019536	191629867	C57BL/6J and C57BL/6NTac	N/A
1	191629867	195471971	C57BL/6J and C57BL/6NTac and CBA/J	Heterozygous
2	3000000	45666278	C57BL/6J and C57BL/6NTac and CBA/J	Heterozygous
2	45666278	139631657	C57BL/6J and C57BL/6NTac	N/A
2	139631657	148625771	C57BL/6J and C57BL/6NTac and CBA/J	Heterozygous
2	148625771	166963888	C57BL/6J and C57BL/6NTac	N/A
2	166963888	175780822	C57BL/6J and C57BL/6NTac and CBA/J	Heterozygous

MiniMUGA Background Analysis v2.3.1

**Diplotype Intervals**	2	175780822	182113224	C57BL/6J and C57BL/6NTac	N/A
	3	3000000	156090101	C57BL/6J and C57BL/6NTac	N/A
	3	156090101	160039680	C57BL/6J and C57BL/6NTac and CBA/J	Heterozygous
	4	3000000	20258658	C57BL/6J and C57BL/6NTac and CBA/J	Heterozygous
	4	20258658	26280383	C57BL/6J and C57BL/6NTac	N/A
	4	26280383	29346519	C57BL/6J and C57BL/6NTac and CBA/J	Heterozygous
	4	29346519	30650814	Unexplained	Heterozygous
	4	30650814	32327128	C57BL/6J and C57BL/6NTac and CBA/J	Heterozygous
	4	32327128	35563307	C57BL/6J and C57BL/6NTac	N/A
	4	35563307	37995481	Unexplained	Heterozygous
	4	37995481	41348396	Unexplained	Homozygous
	4	41348396	43372387	C57BL/6J and C57BL/6NTac and CBA/J	Heterozygous
	4	43372387	43819249	Unexplained	Heterozygous
	4	43819249	49280860	C57BL/6J and C57BL/6NTac and CBA/J	Heterozygous
	4	49280860	54114833	C57BL/6J and C57BL/6NTac	N/A
	4	54114833	101914190	C57BL/6J and C57BL/6NTac and CBA/J	Heterozygous
	4	101914190	156508116	CBA/J	Homozygous
	5	3000000	151834684	C57BL/6J and C57BL/6NTac	N/A
	6	3000000	43184432	C57BL/6J and C57BL/6NTac	N/A
	6	43184432	59791688	C57BL/6J and C57BL/6NTac and CBA/J	Heterozygous
	6	59791688	80057017	C57BL/6J and C57BL/6NTac	N/A
	6	80057017	91366295	C57BL/6J and C57BL/6NTac and CBA/J	Heterozygous
	6	91366295	149736546	C57BL/6J and C57BL/6NTac	N/A
	7	3000000	16360273	C57BL/6J and C57BL/6NTac and CBA/J	Heterozygous
	7	16360273	30335112	CBA/J	Homozygous
	7	30335112	69096424	C57BL/6J and C57BL/6NTac and CBA/J	Heterozygous
	7	69096424	119823617	C57BL/6J and C57BL/6NTac	N/A
	7	119823617	134805535	C57BL/6J and C57BL/6NTac and CBA/J	Heterozygous
	7	134805535	145441459	C57BL/6J and C57BL/6NTac	N/A
	8	3000000	26598325	C57BL/6J and C57BL/6NTac and CBA/J	Heterozygous
	8	26598325	93626178	C57BL/6J and C57BL/6NTac	N/A

8	93626178	119835722	C57BL/6J and C57BL/6NTac and CBA/J	Heterozygous
8	119835722	129401213	C57BL/6J and C57BL/6NTac	N/A
9	3000000	115715944	C57BL/6J and C57BL/6NTac	N/A
9	115715944	124595110	C57BL/6J and C57BL/6NTac and CBA/J	Heterozygous
10	3000000	42858234	C57BL/6J and C57BL/6NTac	N/A
10	42858234	91235291	C57BL/6J and C57BL/6NTac and CBA/J	Heterozygous
10	91235291	130694993	C57BL/6J and C57BL/6NTac	N/A
11	3000000	22302070	C57BL/6J and C57BL/6NTac and CBA/J	Heterozygous
11	22302070	105886229	C57BL/6J and C57BL/6NTac	N/A
11	105886229	112771442	C57BL/6J and C57BL/6NTac and CBA/J	Heterozygous
11	112771442	119038285	C57BL/6J and C57BL/6NTac	N/A
11	119038285	122082543	C57BL/6J and C57BL/6NTac and CBA/J	Heterozygous
12	3000000	28407677	C57BL/6J and C57BL/6NTac	N/A
12	28407677	73558201	C57BL/6J and C57BL/6NTac and CBA/J	Heterozygous
12	73558201	85015902	CBA/J	Homozygous
12	85015902	88650858	C57BL/6J and C57BL/6NTac and CBA/J	Heterozygous
12	88650858	120129022	CBA/J	Homozygous
13	3000000	67442927	C57BL/6J and C57BL/6NTac	N/A
13	67442927	102595519	C57BL/6J and C57BL/6NTac and CBA/J	Heterozygous
13	102595519	120421639	CBA/J	Homozygous
14	3000000	87213383	C57BL/6J and C57BL/6NTac and CBA/J	Heterozygous
14	87213383	124902244	C57BL/6J and C57BL/6NTac	N/A
15	3000000	89025824	C57BL/6J and C57BL/6NTac and CBA/J	Heterozygous
15	89025824	104043685	C57BL/6J and C57BL/6NTac	N/A
16	3000000	29701002	C57BL/6J and C57BL/6NTac	N/A
16	29701002	98207768	C57BL/6J and C57BL/6NTac and CBA/J	Heterozygous
17	3000000	47545390	C57BL/6J and C57BL/6NTac	N/A
17	47545390	74502727	C57BL/6J and C57BL/6NTac and CBA/J	Heterozygous
17	74502727	94987271	C57BL/6J and C57BL/6NTac	N/A
18	3000000	15685654	C57BL/6J and C57BL/6NTac	N/A

MiniMUGA Background Analysis v2.3.1

	18	15685654	20363699	Unexplained	Homozygous
	18	20363699	27036500	CBA/J	Homozygous
	18	27036500	67937187	C57BL/6J and C57BL/6NTac	N/A
	18	67937187	90702639	C57BL/6J and C57BL/6NTac and CBA/J	Heterozygous
	19	3000000	20955280	C57BL/6J and C57BL/6NTac	N/A
	19	20955280	47746251	C57BL/6J and C57BL/6NTac and CBA/J	Heterozygous
	19	47746251	49870985	C57BL/6J and C57BL/6NTac	N/A
	19	49870985	61431566	C57BL/6J and C57BL/6NTac and CBA/J	Heterozygous
	X	3000000	30815921	C57BL/6J and C57BL/6NTac	N/A
	X	30815921	138881041	C57BL/6J and C57BL/6NTac and CBA/J	Heterozygous
	X	138881041	171031299	C57BL/6J and C57BL/6NTac	N/A
	MT	0	0	IBD	Hemizygous
