# Supplementary material for: Deletion of 9p drives B-ALL through heterozygous inactivation of Pax5 and Cd72 in preleukemic cells
Source: JCI Insight. 2026 Feb 17;11(7):e199464. doi: 10.1172/jci.insight.199464 (PMC13134721; doi:10.1172/jci.insight.199464)
Supplement: Supplemental data set 1 [file jciinsight-11-199464-s204.zip › Strain_Genotyping/W531-results-report.pdf]

MiniMUGA Background Analysis v2.3.1

**Sample ID**	W531
**Neogen ID**	AAAU-4534
**Summary**	The genotype of this sample is of **excellent** quality. It is **female** and **outbred**, and likely a mix of **C57BL/6J and C57BL/6NRj** and **CBA/J**. Clustering of unexplained markers is evidence of an additional background strain.<br><br>Diagnostic SNPs are likely explained by the presence of the background strains<br>• Solution 1: 129S5/SvEvBrd and C57BL/6J and C57BL/6NRj<br>◦ C57BL/6J: 64 / 157 (40.8%)<br>◦ C57BL/6NRj: 19 / 39 (48.7%)<br>◦ 129S5/SvEvBrd: 1 / 5 (20.0%)<br>• Solution 2: 129S5/SvEvBrd and C57BL/6JRj and C57BL/6NRj<br>◦ C57BL/6JRj: 64 / 157 (40.8%)<br>◦ C57BL/6NRj: 19 / 39 (48.7%)<br>◦ 129S5/SvEvBrd: 1 / 5 (20.0%)<br><br>NOTE: There is a discrepancy between the diagnostic backgrounds detected and the primary and secondary background analysis (C57BL/6NRj, CBA/J, C57BL/6J). This is uncommon and should be investigated further.<br><br>No genetic constructs were detected in this sample.<br><br>WARNING:<br>• There is a discrepancy between the diagnostic backgrounds detected ((129S5/SvEvBrd and C57BL/6J and C57BL/6NRj) or (129S5/SvEvBrd and C57BL/6JRj and C57BL/6NRj)) and the primary background (C57BL/6J and C57BL/6NRj) and secondary background (CBA/J). This is uncommon and should be investigated further.<br>• The presence of a single diagnostic heterozygous call for a single inbred strain should be treated with caution.<br>• This sample likely has more than 2 genetic backgrounds (unexplained regions and/or fractured ideogram). The strain selected for secondary background may be incorrect. The estimation of the contribution of primary and secondary background are likely incorrect. This can potentially be addressed with input from the user.
**Genotyping Quality**	**Excellent (8 N calls)**<br>All reported results are dependent on genotyping quality.
**Chromosomal Sex**	XX
**Inbreeding Estimate**	62.9% Inbred<br>(Percentage of the genome (autosomal and X chromosomes) that is homozygous or hemizygous for primary, secondary, and unknown backgrounds. See Genome Analysis)

Constructs Detected

BlastR	bpA	Cas9	chlor	cHS4	Cre	DTA	Flp	g_FP	hCMV_a	hCMV_b	hTK_pr	iCre	IRES	Luc	r_FP	rtTA	SV40	tTA
-	-	-	-	-	-	-	-	-	-	-	-	-	-	-	-	-	-	-

MiniMUGA Background Analysis v2.3.1

Refined Ideogram

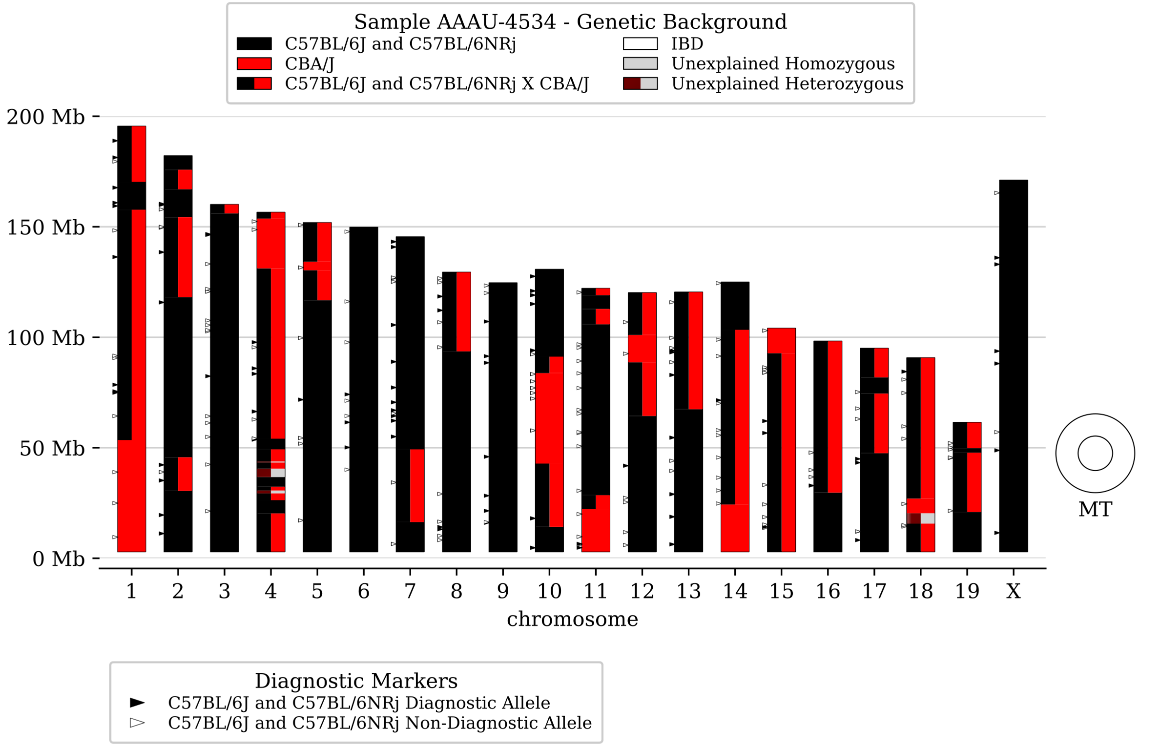

Genome Analysis

Background	Zygosity	Informative Markers	Informative Markers %	Genome %
C57BL/6J and C57BL/6NRj	N/A	1419	53.3%	55.6%
CBA/J	Homozygous	200	7.5%	7.3%
C57BL/6J and C57BL/6NRj X CBA/J	Heterozygous	1027	38.6%	36.7%
Unexplained	Heterozygous	16	0.6%	0.4%
	**Total**	2662	100.0%	100.0%

Y Chromosome

Not Applicable

MT Genome

MT Haplogroup 6 - 100.0% Consistent
Includes C57BL/6J, C57BL/6NRj, CBA/J and 165 other strains

MiniMUGA Background Analysis v2.3.1

Backgrounds Detected (Diagnostic Alleles)

	Diagnostic Alleles Observed			
**Diagnostic Class**	**Homozygous**	**Heterozygous**	**Potential**	**% Observed**
C57BL/6J, C57BL/6JJicTac, C57BL/6JRj	8	35	102	42.2%
C57BL/6J, C57BL/6JEiJ, C57BL/6JJicTac, C57BL/6JRj	4	6	21	47.6%
C57BL/6J, C57BL/6JRj	4	5	31	29.0%
C57BL/6NRj, C57BL/6NTac	3	5	15	53.3%
C57BL/6NJ, C57BL/6NRj, C57BL/6NTac	0	6	10	60.0%
B6N-Tyr<c-Brd>/BrdCrCrl, C57BL/6NCrl, C57BL/6NHsd, C57BL/6NJ, C57BL/6NRj, C57BL/6NTac	1	0	2	50.0%
C57BL/6NCrl, C57BL/6NHsd, C57BL/6NJ, C57BL/6NRj, C57BL/6NTac	0	2	2	100.0%
C57BL/6NRj	0	2	10	20.0%
129S5/SvEvBrd	0	1	5	20.0%
B6N-Tyr<c-Brd>/BrdCrCrl, C57BL/6J, C57BL/6JEiJ, C57BL/6JJicTac, C57BL/6JRj	0	1	1	100.0%
C57BL/6J, C57BL/6JBomTac, C57BL/6JEiJ, C57BL/6JJicTac, C57BL/6JOlaHsd, C57BL/6JRj	0	1	2	50.0%

Minimal Strain Sets Explaining All Diagnostic Classes (Number of Markers Explained):

- Solution 1: 129S5/SvEvBrd and C57BL/6J and C57BL/6NRj
  - C57BL/6J: 64 / 157 (40.8%)
  - C57BL/6NRj: 19 / 39 (48.7%)
  - 129S5/SvEvBrd: 1 / 5 (20.0%)
- Solution 2: 129S5/SvEvBrd and C57BL/6JRj and C57BL/6NRj
  - C57BL/6JRj: 64 / 157 (40.8%)
  - C57BL/6NRj: 19 / 39 (48.7%)
  - 129S5/SvEvBrd: 1 / 5 (20.0%)

Chromosome	Start (Mb)	Stop (Mb)	Background	Zygosity
1	3000000	53457225	CBA/J	Homozygous
1	53457225	157713559	C57BL/6J and C57BL/6NRj and CBA/J	Heterozygous
1	157713559	170316822	C57BL/6J and C57BL/6NRj	N/A
1	170316822	195471971	C57BL/6J and C57BL/6NRj and CBA/J	Heterozygous
2	3000000	30514669	C57BL/6J and C57BL/6NRj	N/A
2	30514669	45666278	C57BL/6J and C57BL/6NRj and CBA/J	Heterozygous
2	45666278	118156944	C57BL/6J and C57BL/6NRj	N/A
2	118156944	154349372	C57BL/6J and C57BL/6NRj and CBA/J	Heterozygous
2	154349372	166963888	C57BL/6J and C57BL/6NRj	N/A
2	166963888	175780822	C57BL/6J and C57BL/6NRj and CBA/J	Heterozygous
2	175780822	182113224	C57BL/6J and C57BL/6NRj	N/A
3	3000000	156090101	C57BL/6J and C57BL/6NRj	N/A

MiniMUGA Background Analysis v2.3.1

**Diplotype Intervals**	3	156090101	160039680	C57BL/6J and C57BL/6NRj and CBA/J	Heterozygous
	4	3000000	20258658	C57BL/6J and C57BL/6NRj and CBA/J	Heterozygous
	4	20258658	26280383	C57BL/6J and C57BL/6NRj	N/A
	4	26280383	29346519	C57BL/6J and C57BL/6NRj and CBA/J	Heterozygous
	4	29346519	30650814	Unexplained	Heterozygous
	4	30650814	32327128	C57BL/6J and C57BL/6NRj and CBA/J	Heterozygous
	4	32327128	36784495	C57BL/6J and C57BL/6NRj	N/A
	4	36784495	40531709	Unexplained	Heterozygous
	4	40531709	43372387	C57BL/6J and C57BL/6NRj and CBA/J	Heterozygous
	4	43372387	43819249	Unexplained	Heterozygous
	4	43819249	49280860	C57BL/6J and C57BL/6NRj and CBA/J	Heterozygous
	4	49280860	54114833	C57BL/6J and C57BL/6NRj	N/A
	4	54114833	131104093	C57BL/6J and C57BL/6NRj and CBA/J	Heterozygous
	4	131104093	153688585	CBA/J	Homozygous
	4	153688585	156508116	C57BL/6J and C57BL/6NRj and CBA/J	Heterozygous
	5	3000000	116795433	C57BL/6J and C57BL/6NRj	N/A
	5	116795433	130280923	C57BL/6J and C57BL/6NRj and CBA/J	Heterozygous
	5	130280923	134172373	CBA/J	Homozygous
	5	134172373	151834684	C57BL/6J and C57BL/6NRj and CBA/J	Heterozygous
	6	3000000	149736546	C57BL/6J and C57BL/6NRj	N/A
	7	3000000	16360273	C57BL/6J and C57BL/6NRj	N/A
	7	16360273	49270765	C57BL/6J and C57BL/6NRj and CBA/J	Heterozygous
	7	49270765	145441459	C57BL/6J and C57BL/6NRj	N/A
	8	3000000	93626178	C57BL/6J and C57BL/6NRj	N/A
	8	93626178	129401213	C57BL/6J and C57BL/6NRj and CBA/J	Heterozygous
	9	3000000	124595110	C57BL/6J and C57BL/6NRj	N/A
	10	3000000	14185354	C57BL/6J and C57BL/6NRj	N/A
	10	14185354	42858234	C57BL/6J and C57BL/6NRj and CBA/J	Heterozygous
	10	42858234	83779430	CBA/J	Homozygous
	10	83779430	91235291	C57BL/6J and C57BL/6NRj and CBA/J	Heterozygous
	10	91235291	130694993	C57BL/6J and C57BL/6NRj	N/A

MiniMUGA Background Analysis v2.3.1

11	3000000	22302070	CBA/J	Homozygous
11	22302070	28525615	C57BL/6J and C57BL/6NRj and CBA/J	Heterozygous
11	28525615	105886229	C57BL/6J and C57BL/6NRj	N/A
11	105886229	112771442	C57BL/6J and C57BL/6NRj and CBA/J	Heterozygous
11	112771442	119038285	C57BL/6J and C57BL/6NRj	N/A
11	119038285	122082543	C57BL/6J and C57BL/6NRj and CBA/J	Heterozygous
12	3000000	64411355	C57BL/6J and C57BL/6NRj	N/A
12	64411355	88650858	C57BL/6J and C57BL/6NRj and CBA/J	Heterozygous
12	88650858	101027932	CBA/J	Homozygous
12	101027932	120129022	C57BL/6J and C57BL/6NRj and CBA/J	Heterozygous
13	3000000	67442927	C57BL/6J and C57BL/6NRj	N/A
13	67442927	120421639	C57BL/6J and C57BL/6NRj and CBA/J	Heterozygous
14	3000000	24355636	CBA/J	Homozygous
14	24355636	103377147	C57BL/6J and C57BL/6NRj and CBA/J	Heterozygous
14	103377147	124902244	C57BL/6J and C57BL/6NRj	N/A
15	3000000	92737752	C57BL/6J and C57BL/6NRj and CBA/J	Heterozygous
15	92737752	104043685	CBA/J	Homozygous
16	3000000	29701002	C57BL/6J and C57BL/6NRj	N/A
16	29701002	98207768	C57BL/6J and C57BL/6NRj and CBA/J	Heterozygous
17	3000000	47545390	C57BL/6J and C57BL/6NRj	N/A
17	47545390	74502727	C57BL/6J and C57BL/6NRj and CBA/J	Heterozygous
17	74502727	81881415	C57BL/6J and C57BL/6NRj	N/A
17	81881415	94987271	C57BL/6J and C57BL/6NRj and CBA/J	Heterozygous
18	3000000	15685654	C57BL/6J and C57BL/6NRj and CBA/J	Heterozygous
18	15685654	20363699	Unexplained	Heterozygous
18	20363699	27036500	CBA/J	Homozygous
18	27036500	90702639	C57BL/6J and C57BL/6NRj and CBA/J	Heterozygous
19	3000000	20955280	C57BL/6J and C57BL/6NRj	N/A
19	20955280	47746251	C57BL/6J and C57BL/6NRj and CBA/J	Heterozygous
19	47746251	49870985	C57BL/6J and C57BL/6NRj	N/A
19	49870985	61431566	C57BL/6J and C57BL/6NRj and CBA/J	Heterozygous

MiniMUGA Background Analysis v2.3.1

	X	3000000	171031299	C57BL/6J and C57BL/6NRj	N/A
	MT	0	0	IBD	Hemizygous
