# Supplementary material for: Deletion of 9p drives B-ALL through heterozygous inactivation of Pax5 and Cd72 in preleukemic cells
Source: JCI Insight. 2026 Feb 17;11(7):e199464. doi: 10.1172/jci.insight.199464 (PMC13134721; doi:10.1172/jci.insight.199464)
Supplement: Supplemental data set 1 [file jciinsight-11-199464-s204.zip › Strain_Genotyping/W715-results-report.pdf]

MiniMUGA Background Analysis v2.3.1

**Sample ID**	W715
**Neogen ID**	AAAU-4535
**Summary**	The genotype of this sample is of **excellent** quality. It is **female** and **outbred**, and likely a mix of **C57BL/6J and C57BL/6NTac** and **CBA/J**. Clustering of unexplained markers is evidence of an additional background strain.<br><br>Diagnostic SNPs are likely explained by the presence of the background strains<br>• Solution 1: C57BL/6J and C57BL/6NRj<br>◦ C57BL/6J: 58 / 158 (36.7%)<br>◦ C57BL/6NRj: 25 / 38 (65.8%)<br>• Solution 2: C57BL/6JRj and C57BL/6NRj<br>◦ C57BL/6JRj: 58 / 158 (36.7%)<br>◦ C57BL/6NRj: 25 / 38 (65.8%)<br><br>NOTE: There is a discrepancy between the diagnostic backgrounds detected and the primary and secondary background analysis (CBA/J, C57BL/6J, C57BL/6NTac). This is uncommon and should be investigated further.<br><br>No genetic constructs were detected in this sample.<br><br>WARNING:<br>• There is a discrepancy between the diagnostic backgrounds detected ((C57BL/6J and C57BL/6NRj) or (C57BL/6JRj and C57BL/6NRj)) and the primary background (C57BL/6J and C57BL/6NTac) and secondary background (CBA/J). This is uncommon and should be investigated further.
**Genotyping Quality**	**Excellent (10 N calls)**<br>All reported results are dependent on genotyping quality.
**Chromosomal Sex**	XX
**Inbreeding Estimate**	54.8% Inbred<br>(Percentage of the genome (autosomal and X chromosomes) that is homozygous or hemizygous for primary, secondary, and unknown backgrounds. See Genome Analysis)

Constructs Detected

BlastR	bpA	Cas9	chlor	cHS4	Cre	DTA	Flp	g_FP	hCMV_a	hCMV_b	hTK_pr	iCre	IRES	Luc	r_FP	rtTA	SV40	tTA
-	-	-	-	-	-	-	-	-	-	-	-	-	-	-	-	-	-	-

MiniMUGA Background Analysis v2.3.1

Refined Ideogram

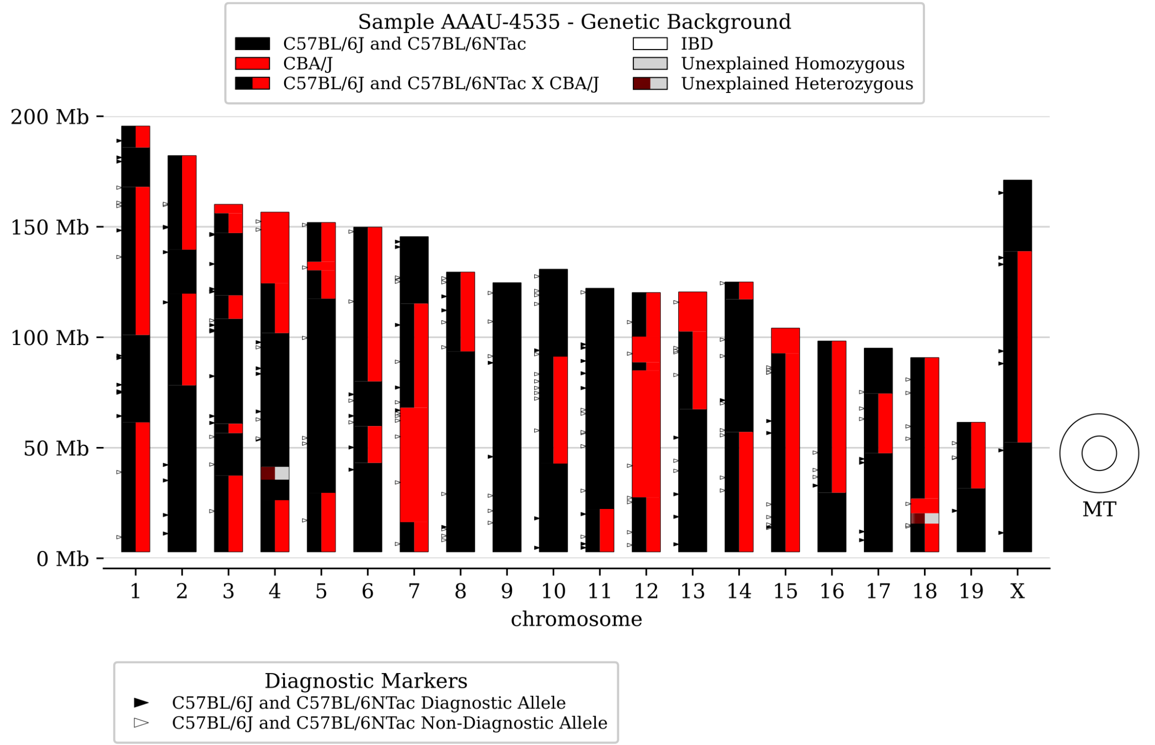

Genome Analysis

Background	Zygosity	Informative Markers	Informative Markers %	Genome %
C57BL/6J and C57BL/6NTac	N/A	1131	42.4%	47.2%
CBA/J	Homozygous	263	9.9%	7.6%
C57BL/6J and C57BL/6NTac X CBA/J	Heterozygous	1254	47.1%	44.8%
Unexplained	Heterozygous	17	0.6%	0.4%
	**Total**	2665	100.0%	100.0%

**Y Chromosome**	Not Applicable
**MT Genome**	MT Haplogroup 6 - 100.0% Consistent<br>Includes C57BL/6J, C57BL/6NTac, CBA/J and 165 other strains

MiniMUGA Background Analysis v2.3.1

Backgrounds Detected (Diagnostic Alleles)

	Diagnostic Alleles Observed			
Diagnostic Class	Homozygous	Heterozygous	Potential	% Observed
C57BL/6J, C57BL/6JJicTac, C57BL/6JRj	6	28	102	33.3%
C57BL/6J, C57BL/6JEiJ, C57BL/6JJicTac, C57BL/6JRj	5	6	21	52.4%
C57BL/6NJ, C57BL/6NRj, C57BL/6NTac	4	5	10	90.0%
C57BL/6NRj, C57BL/6NTac	2	9	15	73.3%
C57BL/6J, C57BL/6JRj	0	10	31	32.3%
B6N-Tyr<c-Brd>/BrdCrCrl, C57BL/6NCrl, C57BL/6NHsd, C57BL/6NJ, C57BL/6NRj, C57BL/6NTac	1	1	2	100.0%
B6N-Tyr<c-Brd>/BrdCrCrl, C57BL/6J, C57BL/6JEiJ, C57BL/6JJicTac, C57BL/6JRj	1	0	1	100.0%
C57BL/6NRj	0	2	10	20.0%
C57BL/6J, C57BL/6JBomTac, C57BL/6JEiJ, C57BL/6JJicTac, C57BL/6JOlaHsd, C57BL/6JRj	0	1	2	50.0%
C57BL/6J, C57BL/6JEiJ, C57BL/6JJicTac, C57BL/6JOlaHsd, C57BL/6JRj	0	1	1	100.0%
C57BL/6NHsd, C57BL/6NJ, C57BL/6NRj, C57BL/6NTac	0	1	1	100.0%

Minimal Strain Sets Explaining All Diagnostic Classes (Number of Markers Explained):

- Solution 1: C57BL/6J and C57BL/6NRj
  - C57BL/6J: 58 / 158 (36.7%)
  - C57BL/6NRj: 25 / 38 (65.8%)
- Solution 2: C57BL/6JRj and C57BL/6NRj
  - C57BL/6JRj: 58 / 158 (36.7%)
  - C57BL/6NRj: 25 / 38 (65.8%)

Chromosome	Start (Mb)	Stop (Mb)	Background	Zygosity
1	3000000	61451021	C57BL/6J and C57BL/6NTac and CBA/J	Heterozygous
1	61451021	101065154	C57BL/6J and C57BL/6NTac	N/A
1	101065154	168019536	C57BL/6J and C57BL/6NTac and CBA/J	Heterozygous
1	168019536	185877635	C57BL/6J and C57BL/6NTac	N/A
1	185877635	195471971	C57BL/6J and C57BL/6NTac and CBA/J	Heterozygous
2	3000000	78267191	C57BL/6J and C57BL/6NTac	N/A
2	78267191	119722376	C57BL/6J and C57BL/6NTac and CBA/J	Heterozygous
2	119722376	139631657	C57BL/6J and C57BL/6NTac	N/A
2	139631657	182113224	C57BL/6J and C57BL/6NTac and CBA/J	Heterozygous
3	3000000	37371933	C57BL/6J and C57BL/6NTac and CBA/J	Heterozygous
3	37371933	56655047	C57BL/6J and C57BL/6NTac	N/A
3	56655047	60850190	C57BL/6J and C57BL/6NTac and CBA/J	Heterozygous
3	60850190	108381941	C57BL/6J and C57BL/6NTac	N/A

MiniMUGA Background Analysis v2.3.1

**Diplotype Intervals**	3	108381941	118919242	C57BL/6J and C57BL/6NTac and CBA/J	Heterozygous
	3	118919242	147169673	C57BL/6J and C57BL/6NTac	N/A
	3	147169673	156090101	C57BL/6J and C57BL/6NTac and CBA/J	Heterozygous
	3	156090101	160039680	CBA/J	Homozygous
	4	3000000	26280383	C57BL/6J and C57BL/6NTac and CBA/J	Heterozygous
	4	26280383	35563307	C57BL/6J and C57BL/6NTac	N/A
	4	35563307	41348396	Unexplained	Heterozygous
	4	41348396	101914190	C57BL/6J and C57BL/6NTac	N/A
	4	101914190	124400069	C57BL/6J and C57BL/6NTac and CBA/J	Heterozygous
	4	124400069	156508116	CBA/J	Homozygous
	5	3000000	29588943	C57BL/6J and C57BL/6NTac and CBA/J	Heterozygous
	5	29588943	117486064	C57BL/6J and C57BL/6NTac	N/A
	5	117486064	130280923	C57BL/6J and C57BL/6NTac and CBA/J	Heterozygous
	5	130280923	134172373	CBA/J	Homozygous
	5	134172373	151834684	C57BL/6J and C57BL/6NTac and CBA/J	Heterozygous
	6	3000000	43184432	C57BL/6J and C57BL/6NTac	N/A
	6	43184432	59791688	C57BL/6J and C57BL/6NTac and CBA/J	Heterozygous
	6	59791688	80057017	C57BL/6J and C57BL/6NTac	N/A
	6	80057017	149736546	C57BL/6J and C57BL/6NTac and CBA/J	Heterozygous
	7	3000000	16360273	C57BL/6J and C57BL/6NTac and CBA/J	Heterozygous
	7	16360273	68153750	CBA/J	Homozygous
	7	68153750	115227247	C57BL/6J and C57BL/6NTac and CBA/J	Heterozygous
	7	115227247	145441459	C57BL/6J and C57BL/6NTac	N/A
	8	3000000	93626178	C57BL/6J and C57BL/6NTac	N/A
	8	93626178	129401213	C57BL/6J and C57BL/6NTac and CBA/J	Heterozygous
	9	3000000	124595110	C57BL/6J and C57BL/6NTac	N/A
	10	3000000	42858234	C57BL/6J and C57BL/6NTac	N/A
	10	42858234	91235291	C57BL/6J and C57BL/6NTac and CBA/J	Heterozygous
	10	91235291	130694993	C57BL/6J and C57BL/6NTac	N/A
	11	3000000	22302070	C57BL/6J and C57BL/6NTac and CBA/J	Heterozygous
	11	22302070	122082543	C57BL/6J and C57BL/6NTac	N/A

MiniMUGA Background Analysis v2.3.1

	12	3000000	27585493	C57BL/6J and C57BL/6NTac and CBA/J	Heterozygous
	12	27585493	85015902	CBA/J	Homozygous
	12	85015902	88650858	C57BL/6J and C57BL/6NTac and CBA/J	Heterozygous
	12	88650858	100284662	CBA/J	Homozygous
	12	100284662	120129022	C57BL/6J and C57BL/6NTac and CBA/J	Heterozygous
	13	3000000	67442927	C57BL/6J and C57BL/6NTac	N/A
	13	67442927	102595519	C57BL/6J and C57BL/6NTac and CBA/J	Heterozygous
	13	102595519	120421639	CBA/J	Homozygous
	14	3000000	57122837	C57BL/6J and C57BL/6NTac and CBA/J	Heterozygous
	14	57122837	117206934	C57BL/6J and C57BL/6NTac	N/A
	14	117206934	124902244	C57BL/6J and C57BL/6NTac and CBA/J	Heterozygous
	15	3000000	92737752	C57BL/6J and C57BL/6NTac and CBA/J	Heterozygous
	15	92737752	104043685	CBA/J	Homozygous
	16	3000000	29701002	C57BL/6J and C57BL/6NTac	N/A
	16	29701002	98207768	C57BL/6J and C57BL/6NTac and CBA/J	Heterozygous
	17	3000000	47545390	C57BL/6J and C57BL/6NTac	N/A
	17	47545390	74502727	C57BL/6J and C57BL/6NTac and CBA/J	Heterozygous
	17	74502727	94987271	C57BL/6J and C57BL/6NTac	N/A
	18	3000000	15685654	C57BL/6J and C57BL/6NTac and CBA/J	Heterozygous
	18	15685654	20363699	Unexplained	Heterozygous
	18	20363699	27036500	CBA/J	Homozygous
	18	27036500	90702639	C57BL/6J and C57BL/6NTac and CBA/J	Heterozygous
	19	3000000	31636352	C57BL/6J and C57BL/6NTac	N/A
	19	31636352	61431566	C57BL/6J and C57BL/6NTac and CBA/J	Heterozygous
	X	3000000	52416633	C57BL/6J and C57BL/6NTac	N/A
	X	52416633	138881041	C57BL/6J and C57BL/6NTac and CBA/J	Heterozygous
	X	138881041	171031299	C57BL/6J and C57BL/6NTac	N/A
	MT	0	0	IBD	Hemizygous
