# Supplementary material for: Deletion of 9p drives B-ALL through heterozygous inactivation of Pax5 and Cd72 in preleukemic cells
Source: JCI Insight. 2026 Feb 17;11(7):e199464. doi: 10.1172/jci.insight.199464 (PMC13134721; doi:10.1172/jci.insight.199464)
Supplement: Supplemental data set 1 [file jciinsight-11-199464-s204.zip › Strain_Genotyping/W718-results-report.pdf]

MiniMUGA Background Analysis v2.3.1

<table>
<tr><th>Sample ID</th><td>W718</td></tr>
<tr><th>Neogen ID</th><td>AAAU-4514</td></tr>
<tr><th>Summary</th><td>The genotype of this sample is of excellent quality. It is female and outbred, and likely a mix of C57BL/6J and C57BL/6NTac and CBA/J. Clustering of unexplained markers is evidence of an additional background strain.

Diagnostic SNPs are likely explained by the presence of the background strains

Solution 1: 129S5/SvEvBrd and C57BL/6J and C57BL/6NTac
C57BL/6J: 64 / 162 (39.5%)
C57BL/6NTac: 18 / 30 (60.0%)
129S5/SvEvBrd: 1 / 5 (20.0%)
Solution 2: 129S5/SvEvBrd and C57BL/6J and C57BL/6NRj
C57BL/6J: 64 / 162 (39.5%)
C57BL/6NRj: 18 / 30 (60.0%)
129S5/SvEvBrd: 1 / 5 (20.0%)
Solution 3: 129S5/SvEvBrd and C57BL/6JRj and C57BL/6NTac
C57BL/6JRj: 64 / 162 (39.5%)
C57BL/6NTac: 18 / 30 (60.0%)
129S5/SvEvBrd: 1 / 5 (20.0%)
Solution 4: 129S5/SvEvBrd and C57BL/6JRj and C57BL/6NRj
C57BL/6JRj: 64 / 162 (39.5%)
C57BL/6NRj: 18 / 30 (60.0%)
129S5/SvEvBrd: 1 / 5 (20.0%)

NOTE: There is a discrepancy between the diagnostic backgrounds detected and the primary and secondary background analysis (CBA/J, C57BL/6J, C57BL/6NTac). This is uncommon and should be investigated further.

No genetic constructs were detected in this sample.

WARNING:

There is a discrepancy between the diagnostic backgrounds detected ((129S5/SvEvBrd and C57BL/6J and C57BL/6NTac) or (129S5/SvEvBrd and C57BL/6J and C57BL/6NRj) or (129S5/SvEvBrd and C57BL/6JRj and C57BL/6NTac) or (129S5/SvEvBrd and C57BL/6JRj and C57BL/6NRj)) and the primary background (C57BL/6J and C57BL/6NTac) and secondary background (CBA/J). This is uncommon and should be investigated further.
The presence of a single diagnostic heterozygous call for a single inbred strain should be treated with caution.
This sample likely has more than 2 genetic backgrounds (unexplained regions and/or fractured ideogram). The strain selected for secondary background may be incorrect. The estimation of the contribution of primary and secondary background are likely incorrect. This can potentially be addressed with input from the user.
</td></tr>
<tr><th>Genotyping Quality</th><td>Excellent (3 N calls)
All reported results are dependent on genotyping quality.</td></tr>
<tr><th>Chromosomal Sex</th><td>XX</td></tr>
<tr><th>Inbreeding Estimate</th><td>56.9% Inbred
(Percentage of the genome (autosomal and X chromosomes) that is homozygous or hemizygous for primary, secondary, and unknown backgrounds. See Genome Analysis)</td></tr>
<tr><th>Constructs Detected</th><td>

BlastR	bpA	Cas9	chlor	cHS4	Cre	DTA	Flp	g_FP	hCMV_a	hCMV_b	hTK_pr	iCre	IRES	Luc	r_FP	rtTA	SV40	tTA
-	-	-	-	-	-	-	-	-	-	-	-	-	-	-	-	-	-	-

</td></tr>
</table>

MiniMUGA Background Analysis v2.3.1

Refined Ideogram

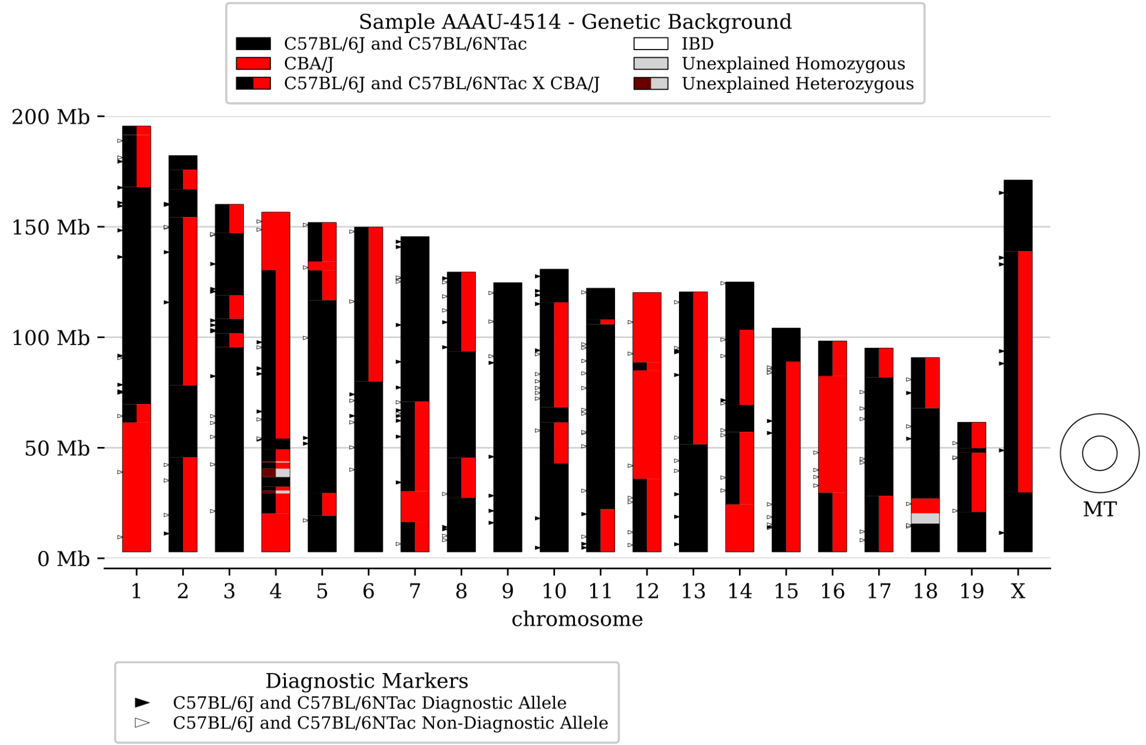

Genome Analysis

Background	Zygosity	Informative Markers	Informative Markers %	Genome %
C57BL/6J and C57BL/6NTac	N/A	1209	45.5%	45.8%
CBA/J	Homozygous	300	11.3%	10.9%
C57BL/6J and C57BL/6NTac X CBA/J	Heterozygous	1135	42.7%	42.9%
Unexplained	Homozygous	6	0.2%	0.2%
Unexplained	Heterozygous	10	0.4%	0.2%
	**Total**	2660	100.1%	100.0%

Y Chromosome

Not Applicable

MT Genome

MT Haplogroup 6 - 100.0% Consistent
Includes C57BL/6J, C57BL/6NTac, CBA/J and 165 other strains

MiniMUGA Background Analysis v2.3.1

Backgrounds Detected (Diagnostic Alleles)

Diagnostic Class	Diagnostic Alleles Observed: Homozygous	Heterozygous	Potential	% Observed
C57BL/6J, C57BL/6JJicTac, C57BL/6JRj	6	38	102	43.1%
C57BL/6J, C57BL/6JEiJ, C57BL/6JJicTac, C57BL/6JRj	3	5	21	38.1%
C57BL/6NRj, C57BL/6NTac	2	7	15	60.0%
C57BL/6J, C57BL/6JRj	0	8	31	25.8%
C57BL/6NJ, C57BL/6NRj, C57BL/6NTac	2	4	10	60.0%
B6N-Tyr<c-Brd>/BrdCrCrl, C57BL/6J, C57BL/6JJicTac, C57BL/6JRj	0	2	5	40.0%
B6N-Tyr<c-Brd>/BrdCrCrl, C57BL/6NCrl, C57BL/6NHsd, C57BL/6NJ, C57BL/6NRj, C57BL/6NTac	1	0	2	50.0%
129S5/SvEvBrd	0	1	5	20.0%
B6N-Tyr<c-Brd>/BrdCrCrl, C57BL/6J, C57BL/6JEiJ, C57BL/6JJicTac, C57BL/6JRj	0	1	1	100.0%
C57BL/6J, C57BL/6JBomTac, C57BL/6JEiJ, C57BL/6JJicTac, C57BL/6JOlaHsd, C57BL/6JRj	0	1	2	50.0%
C57BL/6NCrl, C57BL/6NHsd, C57BL/6NJ, C57BL/6NRj, C57BL/6NTac	0	1	2	50.0%
C57BL/6NHsd, C57BL/6NJ, C57BL/6NRj, C57BL/6NTac	0	1	1	100.0%

Minimal Strain Sets Explaining All Diagnostic Classes (Number of Markers Explained):

- Solution 1: 129S5/SvEvBrd and C57BL/6J and C57BL/6NTac
  - C57BL/6J: 64 / 162 (39.5%)
  - C57BL/6NTac: 18 / 30 (60.0%)
  - 129S5/SvEvBrd: 1 / 5 (20.0%)
- Solution 2: 129S5/SvEvBrd and C57BL/6J and C57BL/6NRj
  - C57BL/6J: 64 / 162 (39.5%)
  - C57BL/6NRj: 18 / 30 (60.0%)
  - 129S5/SvEvBrd: 1 / 5 (20.0%)
- Solution 3: 129S5/SvEvBrd and C57BL/6JRj and C57BL/6NTac
  - C57BL/6JRj: 64 / 162 (39.5%)
  - C57BL/6NTac: 18 / 30 (60.0%)
  - 129S5/SvEvBrd: 1 / 5 (20.0%)
- Solution 4: 129S5/SvEvBrd and C57BL/6JRj and C57BL/6NRj
  - C57BL/6JRj: 64 / 162 (39.5%)
  - C57BL/6NRj: 18 / 30 (60.0%)
  - 129S5/SvEvBrd: 1 / 5 (20.0%)

Chromosome	Start (Mb)	Stop (Mb)	Background	Zygosity
1	3000000	61451021	CBA/J	Homozygous
1	61451021	69700765	C57BL/6J and C57BL/6NTac and CBA/J	Heterozygous
1	69700765	168019536	C57BL/6J and C57BL/6NTac	N/A
1	168019536	191479666	C57BL/6J and C57BL/6NTac and CBA/J	Heterozygous
1	191479666	191629867	C57BL/6J and C57BL/6NTac	N/A
1	191629867	195471971	C57BL/6J and C57BL/6NTac and CBA/J	Heterozygous
2	3000000	45666278	C57BL/6J and C57BL/6NTac and CBA/J	Heterozygous

Diplotype Intervals	2	45666278	78267191	C57BL/6J and C57BL/6NTac	N/A
	2	78267191	154349372	C57BL/6J and C57BL/6NTac and CBA/J	Heterozygous
	2	154349372	166963888	C57BL/6J and C57BL/6NTac	N/A
	2	166963888	175780822	C57BL/6J and C57BL/6NTac and CBA/J	Heterozygous
	2	175780822	182113224	C57BL/6J and C57BL/6NTac	N/A
	3	3000000	95548182	C57BL/6J and C57BL/6NTac	N/A
	3	95548182	101716043	C57BL/6J and C57BL/6NTac and CBA/J	Heterozygous
	3	101716043	108381941	C57BL/6J and C57BL/6NTac	N/A
	3	108381941	118919242	C57BL/6J and C57BL/6NTac and CBA/J	Heterozygous
	3	118919242	147169673	C57BL/6J and C57BL/6NTac	N/A
	3	147169673	160039680	C57BL/6J and C57BL/6NTac and CBA/J	Heterozygous
	4	3000000	20258658	CBA/J	Homozygous
	4	20258658	29346519	C57BL/6J and C57BL/6NTac and CBA/J	Heterozygous
	4	29346519	30650814	Unexplained	Heterozygous
	4	30650814	32327128	C57BL/6J and C57BL/6NTac and CBA/J	Heterozygous
	4	32327128	36784495	C57BL/6J and C57BL/6NTac	N/A
	4	36784495	40531709	Unexplained	Heterozygous
	4	40531709	43372387	C57BL/6J and C57BL/6NTac and CBA/J	Heterozygous
	4	43372387	43819249	Unexplained	Heterozygous
	4	43819249	49280860	C57BL/6J and C57BL/6NTac and CBA/J	Heterozygous
	4	49280860	54114833	C57BL/6J and C57BL/6NTac	N/A
	4	54114833	130336992	C57BL/6J and C57BL/6NTac and CBA/J	Heterozygous
	4	130336992	156508116	CBA/J	Homozygous
	5	3000000	19267794	C57BL/6J and C57BL/6NTac	N/A
	5	19267794	29588943	C57BL/6J and C57BL/6NTac and CBA/J	Heterozygous
	5	29588943	116795433	C57BL/6J and C57BL/6NTac	N/A
	5	116795433	130280923	C57BL/6J and C57BL/6NTac and CBA/J	Heterozygous
	5	130280923	134172373	CBA/J	Homozygous
	5	134172373	151834684	C57BL/6J and C57BL/6NTac and CBA/J	Heterozygous
	6	3000000	80057017	C57BL/6J and C57BL/6NTac	N/A
	6	80057017	149736546	C57BL/6J and C57BL/6NTac and CBA/J	Heterozygous

MiniMUGA Background Analysis v2.3.1

7	3000000	16360273	C57BL/6J and C57BL/6NTac and CBA/J	Heterozygous
7	16360273	30335112	CBA/J	Homozygous
7	30335112	70828686	C57BL/6J and C57BL/6NTac and CBA/J	Heterozygous
7	70828686	145441459	C57BL/6J and C57BL/6NTac	N/A
8	3000000	27348459	C57BL/6J and C57BL/6NTac	N/A
8	27348459	45403996	C57BL/6J and C57BL/6NTac and CBA/J	Heterozygous
8	45403996	93626178	C57BL/6J and C57BL/6NTac	N/A
8	93626178	129401213	C57BL/6J and C57BL/6NTac and CBA/J	Heterozygous
9	3000000	124595110	C57BL/6J and C57BL/6NTac	N/A
10	3000000	42858234	C57BL/6J and C57BL/6NTac	N/A
10	42858234	61450853	C57BL/6J and C57BL/6NTac and CBA/J	Heterozygous
10	61450853	68332199	C57BL/6J and C57BL/6NTac	N/A
10	68332199	115781736	C57BL/6J and C57BL/6NTac and CBA/J	Heterozygous
10	115781736	130694993	C57BL/6J and C57BL/6NTac	N/A
11	3000000	22302070	C57BL/6J and C57BL/6NTac and CBA/J	Heterozygous
11	22302070	105886229	C57BL/6J and C57BL/6NTac	N/A
11	105886229	108040605	C57BL/6J and C57BL/6NTac and CBA/J	Heterozygous
11	108040605	122082543	C57BL/6J and C57BL/6NTac	N/A
12	3000000	35924045	C57BL/6J and C57BL/6NTac and CBA/J	Heterozygous
12	35924045	85015902	CBA/J	Homozygous
12	85015902	88650858	C57BL/6J and C57BL/6NTac and CBA/J	Heterozygous
12	88650858	120129022	CBA/J	Homozygous
13	3000000	51605798	C57BL/6J and C57BL/6NTac	N/A
13	51605798	120421639	C57BL/6J and C57BL/6NTac and CBA/J	Heterozygous
14	3000000	24355636	CBA/J	Homozygous
14	24355636	57122837	C57BL/6J and C57BL/6NTac and CBA/J	Heterozygous
14	57122837	69368817	C57BL/6J and C57BL/6NTac	N/A
14	69368817	103377147	C57BL/6J and C57BL/6NTac and CBA/J	Heterozygous
14	103377147	124902244	C57BL/6J and C57BL/6NTac	N/A
15	3000000	89025824	C57BL/6J and C57BL/6NTac and CBA/J	Heterozygous

MiniMUGA Background Analysis v2.3.1

	15	89025824	104043685	C57BL/6J and C57BL/6NTac	N/A
	16	3000000	29701002	C57BL/6J and C57BL/6NTac and CBA/J	Heterozygous
	16	29701002	82429429	CBA/J	Homozygous
	16	82429429	98207768	C57BL/6J and C57BL/6NTac and CBA/J	Heterozygous
	17	3000000	28225412	C57BL/6J and C57BL/6NTac and CBA/J	Heterozygous
	17	28225412	81881415	C57BL/6J and C57BL/6NTac	N/A
	17	81881415	94987271	C57BL/6J and C57BL/6NTac and CBA/J	Heterozygous
	18	3000000	15685654	C57BL/6J and C57BL/6NTac	N/A
	18	15685654	20363699	Unexplained	Homozygous
	18	20363699	27036500	CBA/J	Homozygous
	18	27036500	67937187	C57BL/6J and C57BL/6NTac	N/A
	18	67937187	90702639	C57BL/6J and C57BL/6NTac and CBA/J	Heterozygous
	19	3000000	20955280	C57BL/6J and C57BL/6NTac	N/A
	19	20955280	47746251	C57BL/6J and C57BL/6NTac and CBA/J	Heterozygous
	19	47746251	49870985	C57BL/6J and C57BL/6NTac	N/A
	19	49870985	61431566	C57BL/6J and C57BL/6NTac and CBA/J	Heterozygous
	X	3000000	29836043	C57BL/6J and C57BL/6NTac	N/A
	X	29836043	138881041	C57BL/6J and C57BL/6NTac and CBA/J	Heterozygous
	X	138881041	171031299	C57BL/6J and C57BL/6NTac	N/A
	MT	0	0	IBD	Hemizygous
