# Supplementary material for: Deletion of 9p drives B-ALL through heterozygous inactivation of Pax5 and Cd72 in preleukemic cells
Source: JCI Insight. 2026 Feb 17;11(7):e199464. doi: 10.1172/jci.insight.199464 (PMC13134721; doi:10.1172/jci.insight.199464)
Supplement: Supplemental data set 1 [file jciinsight-11-199464-s204.zip › Strain_Genotyping/W985-results-report.pdf]

MiniMUGA Background Analysis v2.3.1

**Sample ID**	W985
**Neogen ID**	AAAU-4515
**Summary**	The genotype of this sample is of **excellent** quality. It is **female** and **outbred**, and likely a mix of **C57BL/6J and C57BL/6NTac** and **CBA/J**. Clustering of unexplained markers is evidence of an additional background strain.<br><br>Diagnostic SNPs are likely explained by the presence of the background strains<br>• Solution 1: 129S5/SvEvBrd and C57BL/6J and C57BL/6NRj<br>◦ C57BL/6J: 62 / 158 (39.2%)<br>◦ C57BL/6NRj: 16 / 37 (43.2%)<br>◦ 129S5/SvEvBrd: 1 / 5 (20.0%)<br>• Solution 2: 129S5/SvEvBrd and C57BL/6JRj and C57BL/6NRj<br>◦ C57BL/6JRj: 62 / 158 (39.2%)<br>◦ C57BL/6NRj: 16 / 37 (43.2%)<br>◦ 129S5/SvEvBrd: 1 / 5 (20.0%)<br><br>NOTE: There is a discrepancy between the diagnostic backgrounds detected and the primary and secondary background analysis (CBA/J, C57BL/6J, C57BL/6NTac). This is uncommon and should be investigated further.<br><br>No genetic constructs were detected in this sample.<br><br>WARNING:<br>• There is a discrepancy between the diagnostic backgrounds detected ((129S5/SvEvBrd and C57BL/6J and C57BL/6NRj) or (129S5/SvEvBrd and C57BL/6JRj and C57BL/6NRj)) and the primary background (C57BL/6J and C57BL/6NTac) and secondary background (CBA/J). This is uncommon and should be investigated further.<br>• The presence of a single diagnostic heterozygous call for a single inbred strain should be treated with caution.<br>• This sample likely has more than 2 genetic backgrounds (unexplained regions and/or fractured ideogram). The strain selected for secondary background may be incorrect. The estimation of the contribution of primary and secondary background are likely incorrect. This can potentially be addressed with input from the user.
**Genotyping Quality**	**Excellent (16 N calls)**<br>All reported results are dependent on genotyping quality.
**Chromosomal Sex**	XX
**Inbreeding Estimate**	52.5% Inbred<br>(Percentage of the genome (autosomal and X chromosomes) that is homozygous or hemizygous for primary, secondary, and unknown backgrounds. See Genome Analysis)

Constructs Detected

BlastR	bpA	Cas9	chlor	cHS4	Cre	DTA	Flp	g_FP	hCMV_a	hCMV_b	hTK_pr	iCre	IRES	Luc	r_FP	rtTA	SV40	tTA
-	-	-	-	-	-	-	-	-	-	-	-	-	-	-	-	-	-	-

MiniMUGA Background Analysis v2.3.1

Refined Ideogram

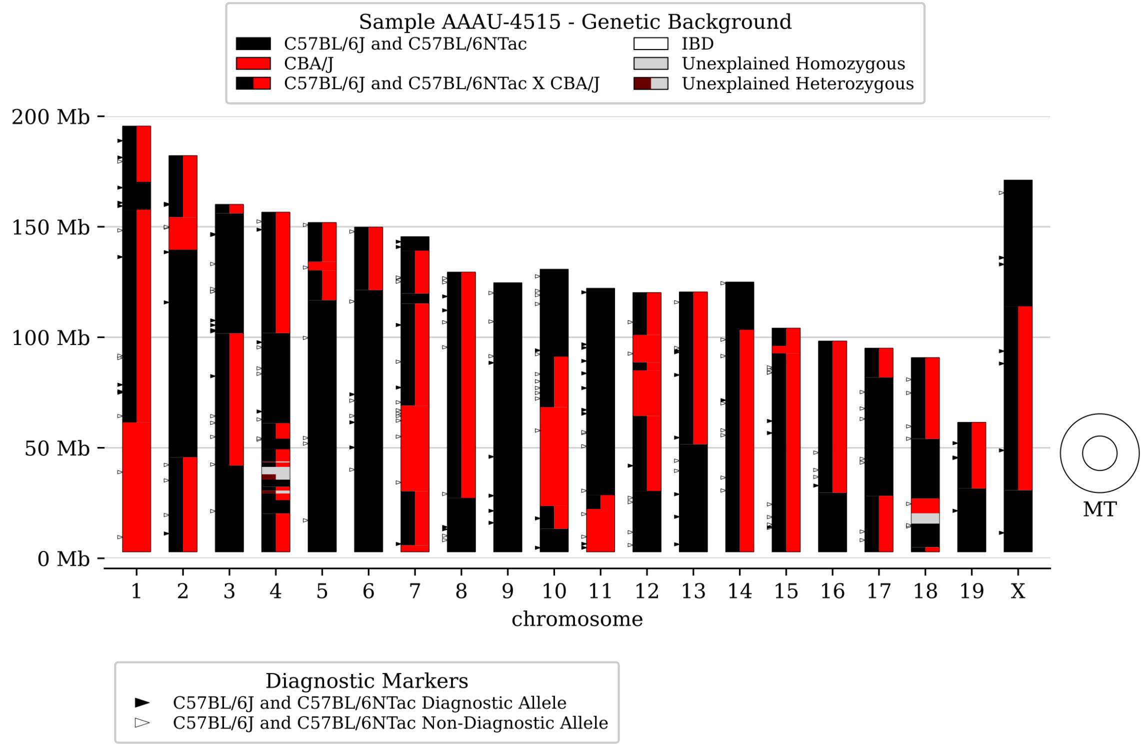

Genome Analysis

Background	Zygosity	Informative Markers	Informative Markers %	Genome %
C57BL/6J and C57BL/6NTac	N/A	1096	41.2%	43.4%
CBA/J	Homozygous	251	9.4%	8.8%
C57BL/6J and C57BL/6NTac X CBA/J	Heterozygous	1288	48.5%	47.4%
Unexplained	Homozygous	11	0.4%	0.3%
Unexplained	Heterozygous	11	0.4%	0.2%
	**Total**	2657	99.9%	100.1%

**Y Chromosome**	Not Applicable
**MT Genome**	MT Haplogroup 6 - 100.0% Consistent<br>Includes C57BL/6J, C57BL/6NTac, CBA/J and 165 other strains

MiniMUGA Background Analysis v2.3.1

Backgrounds Detected (Diagnostic Alleles)

Diagnostic Class	Diagnostic Alleles Observed: Homozygous	Heterozygous	Potential	% Observed
C57BL/6J, C57BL/6JJicTac, C57BL/6JRj	3	38	102	40.2%
C57BL/6J, C57BL/6JEiJ, C57BL/6JJicTac, C57BL/6JRj	2	8	21	47.6%
C57BL/6J, C57BL/6JRj	3	5	31	25.8%
C57BL/6NRj, C57BL/6NTac	2	6	15	53.3%
C57BL/6NJ, C57BL/6NRj, C57BL/6NTac	1	5	10	60.0%
B6N-Tyr<c-Brd>/BrdCrCrl, C57BL/6J, C57BL/6JEiJ, C57BL/6JJicTac, C57BL/6JRj	1	0	1	100.0%
B6N-Tyr<c-Brd>/BrdCrCrl, C57BL/6NCrl, C57BL/6NHsd, C57BL/6NJ, C57BL/6NRj, C57BL/6NTac	1	0	2	50.0%
129S5/SvEvBrd	0	1	5	20.0%
C57BL/6J, C57BL/6JBomTac, C57BL/6JEiJ, C57BL/6JJicTac, C57BL/6JOlaHsd, C57BL/6JRj	0	1	2	50.0%
C57BL/6J, C57BL/6JEiJ, C57BL/6JJicTac, C57BL/6JOlaHsd, C57BL/6JRj	0	1	1	100.0%
C57BL/6NRj	0	1	10	10.0%

Minimal Strain Sets Explaining All Diagnostic Classes (Number of Markers Explained):

- Solution 1: 129S5/SvEvBrd and C57BL/6J and C57BL/6NRj
    - C57BL/6J: 62 / 158 (39.2%)
    - C57BL/6NRj: 16 / 37 (43.2%)
    - 129S5/SvEvBrd: 1 / 5 (20.0%)
- Solution 2: 129S5/SvEvBrd and C57BL/6JRj and C57BL/6NRj
    - C57BL/6JRj: 62 / 158 (39.2%)
    - C57BL/6NRj: 16 / 37 (43.2%)
    - 129S5/SvEvBrd: 1 / 5 (20.0%)

Chromosome	Start (Mb)	Stop (Mb)	Background	Zygosity
1	3000000	61451021	CBA/J	Homozygous
1	61451021	157713559	C57BL/6J and C57BL/6NTac and CBA/J	Heterozygous
1	157713559	170316822	C57BL/6J and C57BL/6NTac	N/A
1	170316822	195471971	C57BL/6J and C57BL/6NTac and CBA/J	Heterozygous
2	3000000	45666278	C57BL/6J and C57BL/6NTac and CBA/J	Heterozygous
2	45666278	139631657	C57BL/6J and C57BL/6NTac	N/A
2	139631657	154349372	CBA/J	Homozygous
2	154349372	182113224	C57BL/6J and C57BL/6NTac and CBA/J	Heterozygous
3	3000000	41975127	C57BL/6J and C57BL/6NTac	N/A
3	41975127	101716043	C57BL/6J and C57BL/6NTac and CBA/J	Heterozygous
3	101716043	156090101	C57BL/6J and C57BL/6NTac	N/A
3	156090101	160039680	C57BL/6J and C57BL/6NTac and CBA/J	Heterozygous

MiniMUGA Background Analysis v2.3.1

**Diplotype Intervals**	4	3000000	20258658	C57BL/6J and C57BL/6NTac and CBA/J	Heterozygous
	4	20258658	26280383	C57BL/6J and C57BL/6NTac	N/A
	4	26280383	29346519	C57BL/6J and C57BL/6NTac and CBA/J	Heterozygous
	4	29346519	30650814	Unexplained	Heterozygous
	4	30650814	32327128	C57BL/6J and C57BL/6NTac and CBA/J	Heterozygous
	4	32327128	35563307	C57BL/6J and C57BL/6NTac	N/A
	4	35563307	37995481	Unexplained	Heterozygous
	4	37995481	41348396	Unexplained	Homozygous
	4	41348396	43372387	C57BL/6J and C57BL/6NTac and CBA/J	Heterozygous
	4	43372387	43819249	Unexplained	Heterozygous
	4	43819249	49280860	C57BL/6J and C57BL/6NTac and CBA/J	Heterozygous
	4	49280860	54114833	C57BL/6J and C57BL/6NTac	N/A
	4	54114833	61028988	C57BL/6J and C57BL/6NTac and CBA/J	Heterozygous
	4	61028988	101914190	C57BL/6J and C57BL/6NTac	N/A
	4	101914190	156508116	C57BL/6J and C57BL/6NTac and CBA/J	Heterozygous
	5	3000000	116795433	C57BL/6J and C57BL/6NTac	N/A
	5	116795433	130280923	C57BL/6J and C57BL/6NTac and CBA/J	Heterozygous
	5	130280923	134172373	CBA/J	Homozygous
	5	134172373	151834684	C57BL/6J and C57BL/6NTac and CBA/J	Heterozygous
	6	3000000	121394377	C57BL/6J and C57BL/6NTac	N/A
	6	121394377	149736546	C57BL/6J and C57BL/6NTac and CBA/J	Heterozygous
	7	3000000	5883284	CBA/J	Homozygous
	7	5883284	30335112	C57BL/6J and C57BL/6NTac and CBA/J	Heterozygous
	7	30335112	69096424	CBA/J	Homozygous
	7	69096424	115227247	C57BL/6J and C57BL/6NTac and CBA/J	Heterozygous
	7	115227247	119823617	C57BL/6J and C57BL/6NTac	N/A
	7	119823617	139153295	C57BL/6J and C57BL/6NTac and CBA/J	Heterozygous
	7	139153295	145441459	C57BL/6J and C57BL/6NTac	N/A
	8	3000000	27348459	C57BL/6J and C57BL/6NTac	N/A
	8	27348459	129401213	C57BL/6J and C57BL/6NTac and CBA/J	Heterozygous
	9	3000000	124595110	C57BL/6J and C57BL/6NTac	N/A

MiniMUGA Background Analysis v2.3.1

10	3000000	13392478	C57BL/6J and C57BL/6NTac	N/A
10	13392478	23654421	C57BL/6J and C57BL/6NTac and CBA/J	Heterozygous
10	23654421	68332199	CBA/J	Homozygous
10	68332199	91235291	C57BL/6J and C57BL/6NTac and CBA/J	Heterozygous
10	91235291	130694993	C57BL/6J and C57BL/6NTac	N/A
11	3000000	22302070	CBA/J	Homozygous
11	22302070	28525615	C57BL/6J and C57BL/6NTac and CBA/J	Heterozygous
11	28525615	122082543	C57BL/6J and C57BL/6NTac	N/A
12	3000000	30464480	C57BL/6J and C57BL/6NTac	N/A
12	30464480	64411355	C57BL/6J and C57BL/6NTac and CBA/J	Heterozygous
12	64411355	85015902	CBA/J	Homozygous
12	85015902	88650858	C57BL/6J and C57BL/6NTac and CBA/J	Heterozygous
12	88650858	101027932	CBA/J	Homozygous
12	101027932	120129022	C57BL/6J and C57BL/6NTac and CBA/J	Heterozygous
13	3000000	51605798	C57BL/6J and C57BL/6NTac	N/A
13	51605798	120421639	C57BL/6J and C57BL/6NTac and CBA/J	Heterozygous
14	3000000	103377147	C57BL/6J and C57BL/6NTac and CBA/J	Heterozygous
14	103377147	124902244	C57BL/6J and C57BL/6NTac	N/A
15	3000000	92737752	C57BL/6J and C57BL/6NTac and CBA/J	Heterozygous
15	92737752	96089091	CBA/J	Homozygous
15	96089091	104043685	C57BL/6J and C57BL/6NTac and CBA/J	Heterozygous
16	3000000	29701002	C57BL/6J and C57BL/6NTac	N/A
16	29701002	98207768	C57BL/6J and C57BL/6NTac and CBA/J	Heterozygous
17	3000000	28225412	C57BL/6J and C57BL/6NTac and CBA/J	Heterozygous
17	28225412	81881415	C57BL/6J and C57BL/6NTac	N/A
17	81881415	94987271	C57BL/6J and C57BL/6NTac and CBA/J	Heterozygous
18	3000000	4990076	C57BL/6J and C57BL/6NTac and CBA/J	Heterozygous
18	4990076	15685654	C57BL/6J and C57BL/6NTac	N/A
18	15685654	20363699	Unexplained	Homozygous
18	20363699	27036500	CBA/J	Homozygous
18	27036500	54023745	C57BL/6J and C57BL/6NTac	N/A

MiniMUGA Background Analysis v2.3.1

	18	54023745	90702639	C57BL/6J and C57BL/6NTac and CBA/J	Heterozygous
	19	3000000	31636352	C57BL/6J and C57BL/6NTac	N/A
	19	31636352	61431566	C57BL/6J and C57BL/6NTac and CBA/J	Heterozygous
	X	3000000	30815921	C57BL/6J and C57BL/6NTac	N/A
	X	30815921	113964840	C57BL/6J and C57BL/6NTac and CBA/J	Heterozygous
	X	113964840	171031299	C57BL/6J and C57BL/6NTac	N/A
	MT	0	0	IBD	Hemizygous
