# Supplementary material for: Deletion of 9p drives B-ALL through heterozygous inactivation of Pax5 and Cd72 in preleukemic cells
Source: JCI Insight. 2026 Feb 17;11(7):e199464. doi: 10.1172/jci.insight.199464 (PMC13134721; doi:10.1172/jci.insight.199464)
Supplement: Supplemental data set 1 [file jciinsight-11-199464-s204.zip › Strain_Genotyping/W986-results-report.pdf]

MiniMUGA Background Analysis v2.3.1

**Sample ID**	W986
**Neogen ID**	AAAU-4517

Summary

The genotype of this sample is of **excellent** quality. It is **female** and **outbred**, and likely a mix of **C57BL/6J and C57BL/6NRj** and **CBA/J**. Clustering of unexplained markers is evidence of an additional background strain.

Diagnostic SNPs are likely explained by the presence of the background strains

- Solution 1: 129S5/SvEvBrd and C57BL/6J and C57BL/6NRj
    - C57BL/6J: 65 / 163 (39.9%)
    - C57BL/6NRj: 21 / 39 (53.8%)
    - 129S5/SvEvBrd: 1 / 5 (20.0%)
- Solution 2: 129S5/SvEvBrd and C57BL/6JRj and C57BL/6NRj
    - C57BL/6JRj: 65 / 163 (39.9%)
    - C57BL/6NRj: 21 / 39 (53.8%)
    - 129S5/SvEvBrd: 1 / 5 (20.0%)

NOTE: There is a discrepancy between the diagnostic backgrounds detected and the primary and secondary background analysis (C57BL/6NRj, CBA/J, C57BL/6J). This is uncommon and should be investigated further.

The sample may contain the following genetic constructs: **bpA**

WARNING:

- There is a discrepancy between the diagnostic backgrounds detected ((129S5/SvEvBrd and C57BL/6J and C57BL/6NRj) or (129S5/SvEvBrd and C57BL/6JRj and C57BL/6NRj)) and the primary background (C57BL/6J and C57BL/6NRj) and secondary background (CBA/J). This is uncommon and should be investigated further.
- The presence of a single diagnostic heterozygous call for a single inbred strain should be treated with caution.
- This sample likely has more than 2 genetic backgrounds (unexplained regions and/or fractured ideogram). The strain selected for secondary background may be incorrect. The estimation of the contribution of primary and secondary background are likely incorrect. This can potentially be addressed with input from the user.

**Genotyping Quality**	**Excellent (10 N calls)**<br>All reported results are dependent on genotyping quality.
**Chromosomal Sex**	XX
**Inbreeding Estimate**	61.7% Inbred<br>(Percentage of the genome (autosomal and X chromosomes) that is homozygous or hemizygous for primary, secondary, and unknown backgrounds. See Genome Analysis)

Constructs Detected

BlastR	bpA	Cas9	chlor	cHS4	Cre	DTA	Flp	g_FP	hCMV_a	hCMV_b	hTK_pr	iCre	IRES	Luc	r_FP	rtTA	SV40	tTA
-	?	-	-	-	-	-	-	-	-	-	-	-	-	-	-	-	-	-

MiniMUGA Background Analysis v2.3.1

Refined Ideogram

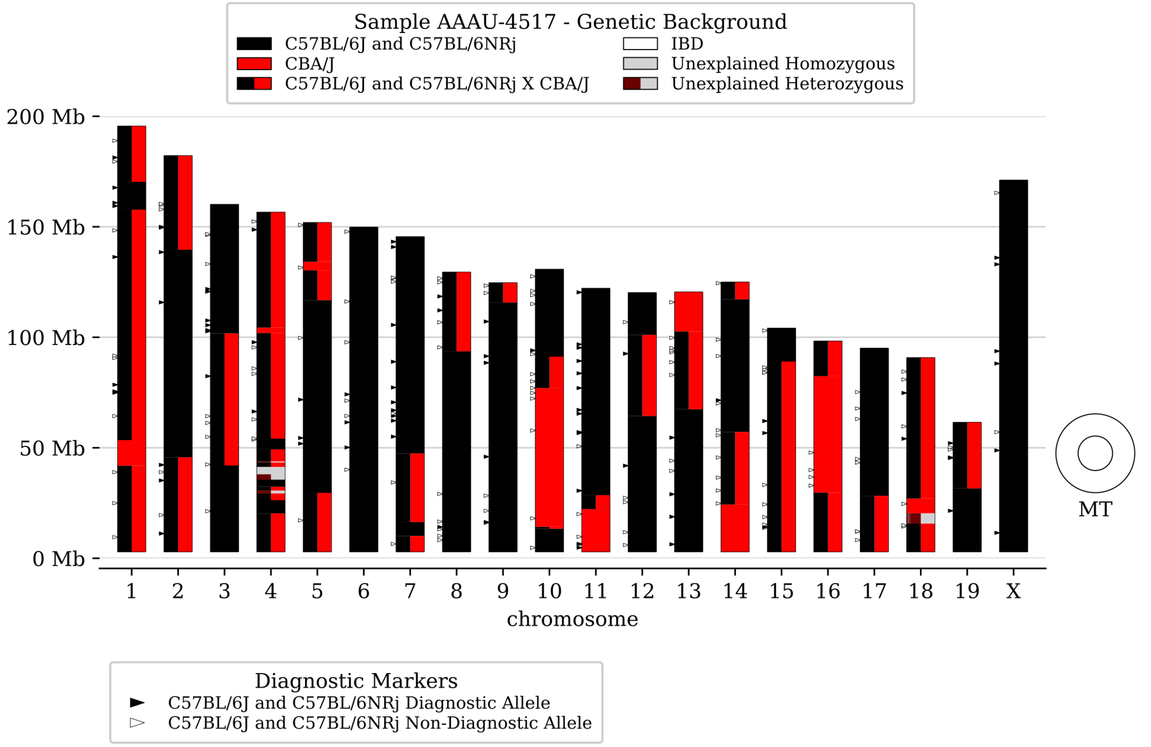

Genome Analysis

Background	Zygosity	Informative Markers	Informative Markers %	Genome %
C57BL/6J and C57BL/6NRj	N/A	1459	54.8%	53.9%
CBA/J	Homozygous	207	7.8%	7.7%
C57BL/6J and C57BL/6NRj X CBA/J	Heterozygous	977	36.7%	37.9%
Unexplained	Homozygous	5	0.2%	0.1%
Unexplained	Heterozygous	15	0.6%	0.3%
	**Total**	2663	100.1%	99.9%

**Y Chromosome**	Not Applicable
**MT Genome**	MT Haplogroup 6 - 100.0% Consistent<br>Includes C57BL/6J, C57BL/6NRj, CBA/J and 165 other strains

MiniMUGA Background Analysis v2.3.1

Backgrounds Detected (Diagnostic Alleles)

	Diagnostic Alleles Observed			
Diagnostic Class	Homozygous	Heterozygous	Potential	% Observed
C57BL/6J, C57BL/6JJicTac, C57BL/6JRj	5	34	102	38.2%
C57BL/6J, C57BL/6JEiJ, C57BL/6JJicTac, C57BL/6JRj	2	10	21	57.1%
C57BL/6J, C57BL/6JRj	3	6	31	29.0%
C57BL/6NRj, C57BL/6NTac	2	6	15	53.3%
C57BL/6NJ, C57BL/6NRj, C57BL/6NTac	2	5	10	70.0%
B6N-Tyr<c-Brd>/BrdCrCrl, C57BL/6NCrl, C57BL/6NHsd, C57BL/6NJ, C57BL/6NRj, C57BL/6NTac	1	1	2	100.0%
B6N-Tyr<c-Brd>/BrdCrCrl, C57BL/6J, C57BL/6JJicTac, C57BL/6JRj	0	2	5	40.0%
C57BL/6NCrl, C57BL/6NHsd, C57BL/6NJ, C57BL/6NRj, C57BL/6NTac	0	2	2	100.0%
C57BL/6NRj	0	2	10	20.0%
129S5/SvEvBrd	0	1	5	20.0%
B6N-Tyr<c-Brd>/BrdCrCrl, C57BL/6J, C57BL/6JEiJ, C57BL/6JJicTac, C57BL/6JRj	0	1	1	100.0%
C57BL/6J, C57BL/6JBomTac, C57BL/6JEiJ, C57BL/6JJicTac, C57BL/6JOlaHsd, C57BL/6JRj	0	1	2	50.0%
C57BL/6J, C57BL/6JEiJ, C57BL/6JJicTac, C57BL/6JOlaHsd, C57BL/6JRj	0	1	1	100.0%

Minimal Strain Sets Explaining All Diagnostic Classes (Number of Markers Explained):

- Solution 1: 129S5/SvEvBrd and C57BL/6J and C57BL/6NRj
  - C57BL/6J: 65 / 163 (39.9%)
  - C57BL/6NRj: 21 / 39 (53.8%)
  - 129S5/SvEvBrd: 1 / 5 (20.0%)
- Solution 2: 129S5/SvEvBrd and C57BL/6JRj and C57BL/6NRj
  - C57BL/6JRj: 65 / 163 (39.9%)
  - C57BL/6NRj: 21 / 39 (53.8%)
  - 129S5/SvEvBrd: 1 / 5 (20.0%)

Chromosome	Start (Mb)	Stop (Mb)	Background	Zygosity
1	3000000	41869819	C57BL/6J and C57BL/6NRj and CBA/J	Heterozygous
1	41869819	53457225	CBA/J	Homozygous
1	53457225	157713559	C57BL/6J and C57BL/6NRj and CBA/J	Heterozygous
1	157713559	170316822	C57BL/6J and C57BL/6NRj	N/A
1	170316822	195471971	C57BL/6J and C57BL/6NRj and CBA/J	Heterozygous
2	3000000	45666278	C57BL/6J and C57BL/6NRj and CBA/J	Heterozygous
2	45666278	139631657	C57BL/6J and C57BL/6NRj	N/A
2	139631657	182113224	C57BL/6J and C57BL/6NRj and CBA/J	Heterozygous
3	3000000	41975127	C57BL/6J and C57BL/6NRj	N/A
3	41975127	101716043	C57BL/6J and C57BL/6NRj and CBA/J	Heterozygous

MiniMUGA Background Analysis v2.3.1

Diplotype Intervals	3	101716043	160039680	C57BL/6J and C57BL/6NRj	N/A
	4	3000000	20258658	C57BL/6J and C57BL/6NRj and CBA/J	Heterozygous
	4	20258658	26280383	C57BL/6J and C57BL/6NRj	N/A
	4	26280383	29346519	C57BL/6J and C57BL/6NRj and CBA/J	Heterozygous
	4	29346519	30650814	Unexplained	Heterozygous
	4	30650814	32327128	C57BL/6J and C57BL/6NRj and CBA/J	Heterozygous
	4	32327128	35563307	C57BL/6J and C57BL/6NRj	N/A
	4	35563307	37995481	Unexplained	Heterozygous
	4	37995481	41348396	Unexplained	Homozygous
	4	41348396	43372387	C57BL/6J and C57BL/6NRj and CBA/J	Heterozygous
	4	43372387	43819249	Unexplained	Heterozygous
	4	43819249	49280860	C57BL/6J and C57BL/6NRj and CBA/J	Heterozygous
	4	49280860	54114833	C57BL/6J and C57BL/6NRj	N/A
	4	54114833	101914190	C57BL/6J and C57BL/6NRj and CBA/J	Heterozygous
	4	101914190	104362509	CBA/J	Homozygous
	4	104362509	156508116	C57BL/6J and C57BL/6NRj and CBA/J	Heterozygous
	5	3000000	29588943	C57BL/6J and C57BL/6NRj and CBA/J	Heterozygous
	5	29588943	116795433	C57BL/6J and C57BL/6NRj	N/A
	5	116795433	130280923	C57BL/6J and C57BL/6NRj and CBA/J	Heterozygous
	5	130280923	134172373	CBA/J	Homozygous
	5	134172373	151834684	C57BL/6J and C57BL/6NRj and CBA/J	Heterozygous
	6	3000000	149736546	C57BL/6J and C57BL/6NRj	N/A
	7	3000000	10069735	C57BL/6J and C57BL/6NRj and CBA/J	Heterozygous
	7	10069735	16360273	C57BL/6J and C57BL/6NRj	N/A
	7	16360273	47395440	C57BL/6J and C57BL/6NRj and CBA/J	Heterozygous
	7	47395440	145441459	C57BL/6J and C57BL/6NRj	N/A
	8	3000000	93626178	C57BL/6J and C57BL/6NRj	N/A
	8	93626178	129401213	C57BL/6J and C57BL/6NRj and CBA/J	Heterozygous
	9	3000000	115715944	C57BL/6J and C57BL/6NRj	N/A
	9	115715944	124595110	C57BL/6J and C57BL/6NRj and CBA/J	Heterozygous
	10	3000000	13392478	C57BL/6J and C57BL/6NRj	N/A

MiniMUGA Background Analysis v2.3.1

10	13392478	14185354	C57BL/6J and C57BL/6NRj and CBA/J	Heterozygous
10	14185354	77055656	CBA/J	Homozygous
10	77055656	91235291	C57BL/6J and C57BL/6NRj and CBA/J	Heterozygous
10	91235291	130694993	C57BL/6J and C57BL/6NRj	N/A
11	3000000	22302070	CBA/J	Homozygous
11	22302070	28525615	C57BL/6J and C57BL/6NRj and CBA/J	Heterozygous
11	28525615	122082543	C57BL/6J and C57BL/6NRj	N/A
12	3000000	64411355	C57BL/6J and C57BL/6NRj	N/A
12	64411355	101027932	C57BL/6J and C57BL/6NRj and CBA/J	Heterozygous
12	101027932	120129022	C57BL/6J and C57BL/6NRj	N/A
13	3000000	67442927	C57BL/6J and C57BL/6NRj	N/A
13	67442927	102595519	C57BL/6J and C57BL/6NRj and CBA/J	Heterozygous
13	102595519	120421639	CBA/J	Homozygous
14	3000000	24355636	CBA/J	Homozygous
14	24355636	57122837	C57BL/6J and C57BL/6NRj and CBA/J	Heterozygous
14	57122837	117206934	C57BL/6J and C57BL/6NRj	N/A
14	117206934	124902244	C57BL/6J and C57BL/6NRj and CBA/J	Heterozygous
15	3000000	89025824	C57BL/6J and C57BL/6NRj and CBA/J	Heterozygous
15	89025824	104043685	C57BL/6J and C57BL/6NRj	N/A
16	3000000	29701002	C57BL/6J and C57BL/6NRj and CBA/J	Heterozygous
16	29701002	82429429	CBA/J	Homozygous
16	82429429	98207768	C57BL/6J and C57BL/6NRj and CBA/J	Heterozygous
17	3000000	28225412	C57BL/6J and C57BL/6NRj and CBA/J	Heterozygous
17	28225412	94987271	C57BL/6J and C57BL/6NRj	N/A
18	3000000	15685654	C57BL/6J and C57BL/6NRj and CBA/J	Heterozygous
18	15685654	20363699	Unexplained	Heterozygous
18	20363699	27036500	CBA/J	Homozygous
18	27036500	90702639	C57BL/6J and C57BL/6NRj and CBA/J	Heterozygous
19	3000000	31636352	C57BL/6J and C57BL/6NRj	N/A
19	31636352	61431566	C57BL/6J and C57BL/6NRj and CBA/J	Heterozygous
X	3000000	171031299	C57BL/6J and C57BL/6NRj	N/A
MT	0	0	IBD	Hemizygous
