# Supplementary material for: Deletion of 9p drives B-ALL through heterozygous inactivation of Pax5 and Cd72 in preleukemic cells
Source: JCI Insight. 2026 Feb 17;11(7):e199464. doi: 10.1172/jci.insight.199464 (PMC13134721; doi:10.1172/jci.insight.199464)
Supplement: Supplemental data set 1 [file jciinsight-11-199464-s204.zip › Strain_Genotyping/W987-results-report.pdf]

MiniMUGA Background Analysis v2.3.1

Sample ID

W987

Neogen ID

AAAU-4518

Summary

The genotype of this sample is of **excellent** quality. It is **female** and **outbred**, and likely a mix of **C57BL/6J and C57BL/6NTac** and **CBA/J**. Clustering of unexplained markers is evidence of an additional background strain.

Diagnostic SNPs are likely explained by the presence of the background strains

- Solution 1: 129S5/SvEvBrd and C57BL/6J and C57BL/6NRj
  - C57BL/6J: 85 / 163 (52.1%)
  - C57BL/6NRj: 21 / 40 (52.5%)
  - 129S5/SvEvBrd: 1 / 5 (20.0%)
- Solution 2: 129S5/SvEvBrd and C57BL/6JRj and C57BL/6NRj
  - C57BL/6JRj: 85 / 163 (52.1%)
  - C57BL/6NRj: 21 / 40 (52.5%)
  - 129S5/SvEvBrd: 1 / 5 (20.0%)

NOTE: There is a discrepancy between the diagnostic backgrounds detected and the primary and secondary background analysis (CBA/J, C57BL/6J, C57BL/6NTac). This is uncommon and should be investigated further.

No genetic constructs were detected in this sample.

WARNING:

- There is a discrepancy between the diagnostic backgrounds detected ((129S5/SvEvBrd and C57BL/6J and C57BL/6NRj) or (129S5/SvEvBrd and C57BL/6JRj and C57BL/6NRj)) and the primary background (C57BL/6J and C57BL/6NTac) and secondary background (CBA/J). This is uncommon and should be investigated further.
- The presence of a single diagnostic heterozygous call for a single inbred strain should be treated with caution.
- This sample likely has more than 2 genetic backgrounds (unexplained regions and/or fractured ideogram). The strain selected for secondary background may be incorrect. The estimation of the contribution of primary and secondary background are likely incorrect. This can potentially be addressed with input from the user.

Genotyping Quality

Excellent (13 N calls)
All reported results are dependent on genotyping quality.

Chromosomal Sex

XX

Inbreeding Estimate

57.0% Inbred
(Percentage of the genome (autosomal and X chromosomes) that is homozygous or hemizygous for primary, secondary, and unknown backgrounds. See Genome Analysis)

Constructs Detected

BlastR	bpA	Cas9	chlor	cHS4	Cre	DTA	Flp	g_FP	hCMV_a	hCMV_b	hTK_pr	iCre	IRES	Luc	r_FP	rtTA	SV40	tTA
-	-	-	-	-	-	-	-	-	-	-	-	-	-	-	-	-	-	-

MiniMUGA Background Analysis v2.3.1

**Refined Ideogram**	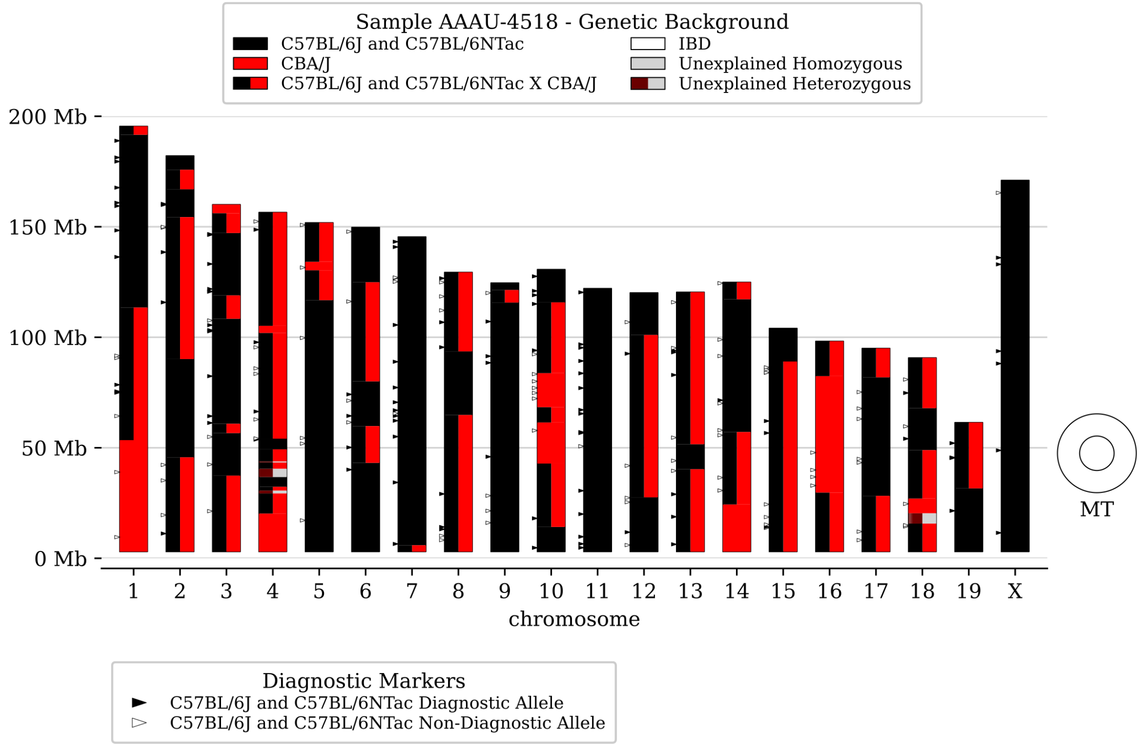

Genome Analysis

Background	Zygosity	Informative Markers	Informative Markers %	Genome %
C57BL/6J and C57BL/6NTac	N/A	1367	51.5%	49.5%
CBA/J	Homozygous	185	7.0%	7.5%
C57BL/6J and C57BL/6NTac X CBA/J	Heterozygous	1082	40.8%	42.6%
Unexplained	Heterozygous	18	0.7%	0.4%
	**Total**	2652	100.0%	100.0%

**Y Chromosome**	Not Applicable
**MT Genome**	MT Haplogroup 6 - 100.0% Consistent<br>Includes C57BL/6J, C57BL/6NTac, CBA/J and 165 other strains

MiniMUGA Background Analysis v2.3.1

Backgrounds Detected (Diagnostic Alleles)

Diagnostic Class	Diagnostic Alleles Observed: Homozygous	Diagnostic Alleles Observed: Heterozygous	Potential	% Observed
C57BL/6J, C57BL/6JJicTac, C57BL/6JRj	4	54	102	56.9%
C57BL/6J, C57BL/6JEiJ, C57BL/6JJicTac, C57BL/6JRj	1	10	21	52.4%
C57BL/6J, C57BL/6JRj	1	10	31	35.5%
C57BL/6NRj, C57BL/6NTac	1	8	15	60.0%
C57BL/6NJ, C57BL/6NRj, C57BL/6NTac	2	4	10	60.0%
B6N-Tyr<c-Brd>/BrdCrCrl, C57BL/6J, C57BL/6JJicTac, C57BL/6JRj	0	2	5	40.0%
B6N-Tyr<c-Brd>/BrdCrCrl, C57BL/6NCrl, C57BL/6NHsd, C57BL/6NJ, C57BL/6NRj, C57BL/6NTac	1	0	2	50.0%
C57BL/6NCrl, C57BL/6NHsd, C57BL/6NJ, C57BL/6NRj, C57BL/6NTac	0	2	2	100.0%
C57BL/6NRj	0	2	10	20.0%
129S5/SvEvBrd	0	1	5	20.0%
B6N-Tyr<c-Brd>/BrdCrCrl, C57BL/6J, C57BL/6JEiJ, C57BL/6JJicTac, C57BL/6JRj	0	1	1	100.0%
C57BL/6J, C57BL/6JBomTac, C57BL/6JEiJ, C57BL/6JJicTac, C57BL/6JOlaHsd, C57BL/6JRj	0	1	2	50.0%
C57BL/6J, C57BL/6JEiJ, C57BL/6JJicTac, C57BL/6JOlaHsd, C57BL/6JRj	0	1	1	100.0%
C57BL/6NHsd, C57BL/6NJ, C57BL/6NRj, C57BL/6NTac	0	1	1	100.0%

Minimal Strain Sets Explaining All Diagnostic Classes (Number of Markers Explained):

- Solution 1: 129S5/SvEvBrd and C57BL/6J and C57BL/6NRj
    - C57BL/6J: 85 / 163 (52.1%)
    - C57BL/6NRj: 21 / 40 (52.5%)
    - 129S5/SvEvBrd: 1 / 5 (20.0%)
- Solution 2: 129S5/SvEvBrd and C57BL/6JRj and C57BL/6NRj
    - C57BL/6JRj: 85 / 163 (52.1%)
    - C57BL/6NRj: 21 / 40 (52.5%)
    - 129S5/SvEvBrd: 1 / 5 (20.0%)

Chromosome	Start (Mb)	Stop (Mb)	Background	Zygosity
1	3000000	53457225	CBA/J	Homozygous
1	53457225	113437009	C57BL/6J and C57BL/6NTac and CBA/J	Heterozygous
1	113437009	191629867	C57BL/6J and C57BL/6NTac	N/A
1	191629867	195471971	C57BL/6J and C57BL/6NTac and CBA/J	Heterozygous
2	3000000	45666278	C57BL/6J and C57BL/6NTac and CBA/J	Heterozygous
2	45666278	90144439	C57BL/6J and C57BL/6NTac	N/A
2	90144439	154349372	C57BL/6J and C57BL/6NTac and CBA/J	Heterozygous
2	154349372	166963888	C57BL/6J and C57BL/6NTac	N/A
2	166963888	175780822	C57BL/6J and C57BL/6NTac and CBA/J	Heterozygous

MiniMUGA Background Analysis v2.3.1

**Diplotype Intervals**	2	175780822	182113224	C57BL/6J and C57BL/6NTac	N/A
	3	3000000	37371933	C57BL/6J and C57BL/6NTac and CBA/J	Heterozygous
	3	37371933	56655047	C57BL/6J and C57BL/6NTac	N/A
	3	56655047	60850190	C57BL/6J and C57BL/6NTac and CBA/J	Heterozygous
	3	60850190	108381941	C57BL/6J and C57BL/6NTac	N/A
	3	108381941	118919242	C57BL/6J and C57BL/6NTac and CBA/J	Heterozygous
	3	118919242	147169673	C57BL/6J and C57BL/6NTac	N/A
	3	147169673	156090101	C57BL/6J and C57BL/6NTac and CBA/J	Heterozygous
	3	156090101	160039680	CBA/J	Homozygous
	4	3000000	20258658	CBA/J	Homozygous
	4	20258658	29346519	C57BL/6J and C57BL/6NTac and CBA/J	Heterozygous
	4	29346519	30650814	Unexplained	Heterozygous
	4	30650814	32327128	C57BL/6J and C57BL/6NTac and CBA/J	Heterozygous
	4	32327128	36784495	C57BL/6J and C57BL/6NTac	N/A
	4	36784495	40531709	Unexplained	Heterozygous
	4	40531709	43372387	C57BL/6J and C57BL/6NTac and CBA/J	Heterozygous
	4	43372387	43819249	Unexplained	Heterozygous
	4	43819249	49280860	C57BL/6J and C57BL/6NTac and CBA/J	Heterozygous
	4	49280860	54114833	C57BL/6J and C57BL/6NTac	N/A
	4	54114833	101914190	C57BL/6J and C57BL/6NTac and CBA/J	Heterozygous
	4	101914190	105177765	CBA/J	Homozygous
	4	105177765	156508116	C57BL/6J and C57BL/6NTac and CBA/J	Heterozygous
	5	3000000	116795433	C57BL/6J and C57BL/6NTac	N/A
	5	116795433	130280923	C57BL/6J and C57BL/6NTac and CBA/J	Heterozygous
	5	130280923	134172373	CBA/J	Homozygous
	5	134172373	151834684	C57BL/6J and C57BL/6NTac and CBA/J	Heterozygous
	6	3000000	43184432	C57BL/6J and C57BL/6NTac	N/A
	6	43184432	59791688	C57BL/6J and C57BL/6NTac and CBA/J	Heterozygous
	6	59791688	80057017	C57BL/6J and C57BL/6NTac	N/A
	6	80057017	124881471	C57BL/6J and C57BL/6NTac and CBA/J	Heterozygous
	6	124881471	149736546	C57BL/6J and C57BL/6NTac	N/A

MiniMUGA Background Analysis v2.3.1

	7	3000000	5883380	C57BL/6J and C57BL/6NTac and CBA/J	Heterozygous
	7	5883380	145441459	C57BL/6J and C57BL/6NTac	N/A
	8	3000000	64818329	C57BL/6J and C57BL/6NTac and CBA/J	Heterozygous
	8	64818329	93626178	C57BL/6J and C57BL/6NTac	N/A
	8	93626178	129401213	C57BL/6J and C57BL/6NTac and CBA/J	Heterozygous
	9	3000000	115715944	C57BL/6J and C57BL/6NTac	N/A
	9	115715944	121366889	C57BL/6J and C57BL/6NTac and CBA/J	Heterozygous
	9	121366889	124595110	C57BL/6J and C57BL/6NTac	N/A
	10	3000000	14185354	C57BL/6J and C57BL/6NTac	N/A
	10	14185354	42858234	C57BL/6J and C57BL/6NTac and CBA/J	Heterozygous
	10	42858234	61450853	CBA/J	Homozygous
	10	61450853	68332199	C57BL/6J and C57BL/6NTac and CBA/J	Heterozygous
	10	68332199	83779430	CBA/J	Homozygous
	10	83779430	115781736	C57BL/6J and C57BL/6NTac and CBA/J	Heterozygous
	10	115781736	130694993	C57BL/6J and C57BL/6NTac	N/A
	11	3000000	122082543	C57BL/6J and C57BL/6NTac	N/A
	12	3000000	27585493	C57BL/6J and C57BL/6NTac	N/A
	12	27585493	101027932	C57BL/6J and C57BL/6NTac and CBA/J	Heterozygous
	12	101027932	120129022	C57BL/6J and C57BL/6NTac	N/A
	13	3000000	40278277	C57BL/6J and C57BL/6NTac and CBA/J	Heterozygous
	13	40278277	51605798	C57BL/6J and C57BL/6NTac	N/A
	13	51605798	120421639	C57BL/6J and C57BL/6NTac and CBA/J	Heterozygous
	14	3000000	24355636	CBA/J	Homozygous
	14	24355636	57122837	C57BL/6J and C57BL/6NTac and CBA/J	Heterozygous
	14	57122837	117206934	C57BL/6J and C57BL/6NTac	N/A
	14	117206934	124902244	C57BL/6J and C57BL/6NTac and CBA/J	Heterozygous
	15	3000000	89025824	C57BL/6J and C57BL/6NTac and CBA/J	Heterozygous
	15	89025824	104043685	C57BL/6J and C57BL/6NTac	N/A
	16	3000000	29701002	C57BL/6J and C57BL/6NTac and CBA/J	Heterozygous
	16	29701002	82429429	CBA/J	Homozygous

MiniMUGA Background Analysis v2.3.1

	16	82429429	98207768	C57BL/6J and C57BL/6NTac and CBA/J	Heterozygous
	17	3000000	28225412	C57BL/6J and C57BL/6NTac and CBA/J	Heterozygous
	17	28225412	81881415	C57BL/6J and C57BL/6NTac	N/A
	17	81881415	94987271	C57BL/6J and C57BL/6NTac and CBA/J	Heterozygous
	18	3000000	15685654	C57BL/6J and C57BL/6NTac and CBA/J	Heterozygous
	18	15685654	20363699	Unexplained	Heterozygous
	18	20363699	27036500	CBA/J	Homozygous
	18	27036500	48930428	C57BL/6J and C57BL/6NTac and CBA/J	Heterozygous
	18	48930428	67937187	C57BL/6J and C57BL/6NTac	N/A
	18	67937187	90702639	C57BL/6J and C57BL/6NTac and CBA/J	Heterozygous
	19	3000000	31636352	C57BL/6J and C57BL/6NTac	N/A
	19	31636352	61431566	C57BL/6J and C57BL/6NTac and CBA/J	Heterozygous
	X	3000000	171031299	C57BL/6J and C57BL/6NTac	N/A
	MT	0	0	IBD	Hemizygous
