# Supplementary material for: Deletion of 9p drives B-ALL through heterozygous inactivation of Pax5 and Cd72 in preleukemic cells
Source: JCI Insight. 2026 Feb 17;11(7):e199464. doi: 10.1172/jci.insight.199464 (PMC13134721; doi:10.1172/jci.insight.199464)
Supplement: Supplemental data set 1 [file jciinsight-11-199464-s204.zip › Strain_Genotyping/X540-results-report.pdf]

MiniMUGA Background Analysis v2.3.1

<table>
<tr><td>Sample ID</td><td>X540</td></tr>
<tr><td>Neogen ID</td><td>AAAU-4516</td></tr>
<tr><td>Summary</td><td>The genotype of this sample is of excellent quality. It is female and outbred, and likely a mix of C57BL/6J and C57BL/6NTac and CBA/J. Clustering of unexplained markers is evidence of an additional background strain.

Diagnostic SNPs are likely explained by the presence of the background strains
Solution 1: 129S5/SvEvBrd and C57BL/6J and C57BL/6NRj
C57BL/6J: 66 / 162 (40.7%)
C57BL/6NRj: 28 / 40 (70.0%)
129S5/SvEvBrd: 1 / 5 (20.0%)
Solution 2: 129S5/SvEvBrd and C57BL/6JRj and C57BL/6NRj
C57BL/6JRj: 66 / 162 (40.7%)
C57BL/6NRj: 28 / 40 (70.0%)
129S5/SvEvBrd: 1 / 5 (20.0%)
NOTE: There is a discrepancy between the diagnostic backgrounds detected and the primary and secondary background analysis (CBA/J, C57BL/6J, C57BL/6NTac). This is uncommon and should be investigated further.

No genetic constructs were detected in this sample.

WARNING:
There is a discrepancy between the diagnostic backgrounds detected ((129S5/SvEvBrd and C57BL/6J and C57BL/6NRj) or (129S5/SvEvBrd and C57BL/6JRj and C57BL/6NRj)) and the primary background (C57BL/6J and C57BL/6NTac) and secondary background (CBA/J). This is uncommon and should be investigated further.
The presence of a single diagnostic heterozygous call for a single inbred strain should be treated with caution.
This sample likely has more than 2 genetic backgrounds (unexplained regions and/or fractured ideogram). The strain selected for secondary background may be incorrect. The estimation of the contribution of primary and secondary background are likely incorrect. This can potentially be addressed with input from the user.</td></tr>
<tr><td>Genotyping Quality</td><td>Excellent (17 N calls)
All reported results are dependent on genotyping quality.</td></tr>
<tr><td>Chromosomal Sex</td><td>XX</td></tr>
<tr><td>Inbreeding Estimate</td><td>40.4% Inbred
(Percentage of the genome (autosomal and X chromosomes) that is homozygous or hemizygous for primary, secondary, and unknown backgrounds. See Genome Analysis)</td></tr>
<tr><td>Constructs Detected</td><td>

BlastR	bpA	Cas9	chlor	cHS4	Cre	DTA	Flp	g_FP	hCMV_a	hCMV_b	hTK_pr	iCre	IRES	Luc	r_FP	rtTA	SV40	tTA
-	-	-	-	-	-	-	-	-	-	-	-	-	-	-	-	-	-	-

</td></tr>
</table>

MiniMUGA Background Analysis v2.3.1

Refined Ideogram

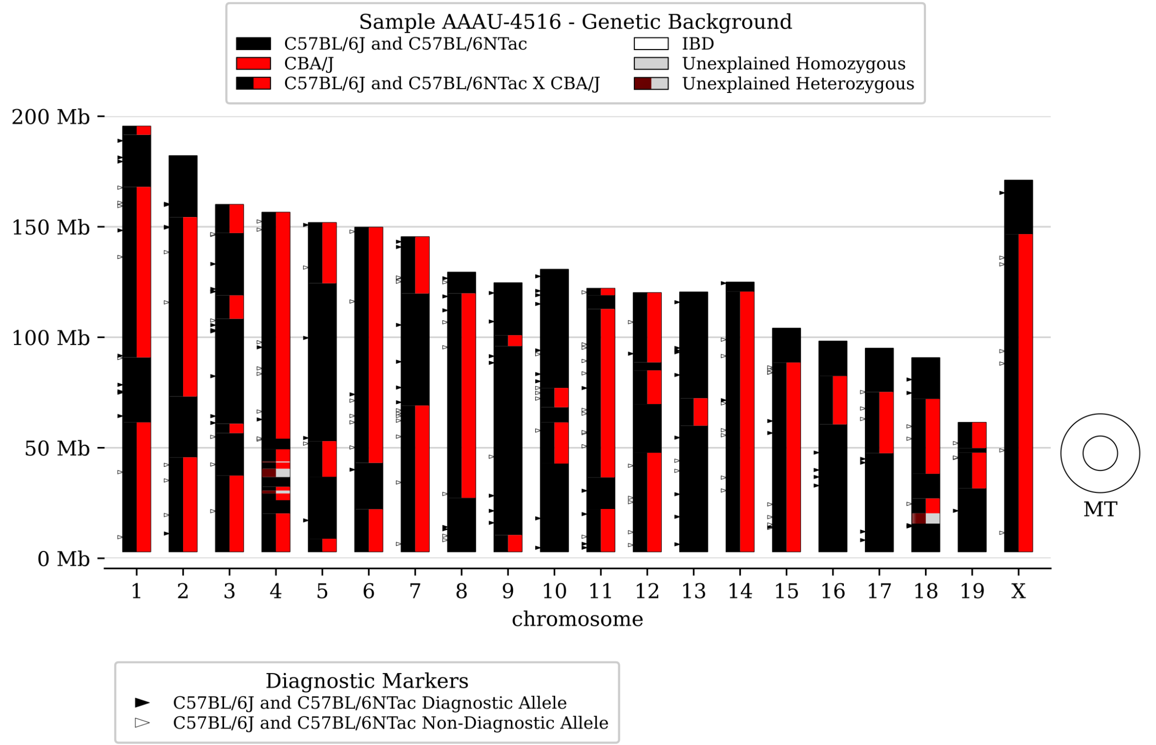

Genome Analysis

Background	Zygosity	Informative Markers	Informative Markers %	Genome %
C57BL/6J and C57BL/6NTac	N/A	1152	43.6%	40.4%
C57BL/6J and C57BL/6NTac X CBA/J	Heterozygous	1473	55.7%	59.2%
Unexplained	Heterozygous	19	0.7%	0.4%
	**Total**	2644	100.0%	100.0%

Y Chromosome

Not Applicable

MT Genome

MT Haplogroup 6 - 100.0% Consistent
Includes C57BL/6J, C57BL/6NTac, CBA/J and 165 other strains

MiniMUGA Background Analysis v2.3.1

Backgrounds Detected (Diagnostic Alleles)

Diagnostic Class	Diagnostic Alleles Observed: Homozygous	Heterozygous	Potential	% Observed
C57BL/6J, C57BL/6JJicTac, C57BL/6JRj	0	44	102	43.1%
C57BL/6NRj, C57BL/6NTac	0	12	15	80.0%
C57BL/6NJ, C57BL/6NRj, C57BL/6NTac	0	10	10	100.0%
C57BL/6J, C57BL/6JRj	0	9	31	29.0%
C57BL/6J, C57BL/6JEiJ, C57BL/6JJicTac, C57BL/6JRj	0	7	21	33.3%
B6N-Tyr<c-Brd>/BrdCrCrl, C57BL/6J, C57BL/6JJicTac, C57BL/6JRj	0	4	5	80.0%
B6N-Tyr<c-Brd>/BrdCrCrl, C57BL/6NCrl, C57BL/6NHsd, C57BL/6NJ, C57BL/6NRj, C57BL/6NTac	0	2	2	100.0%
C57BL/6NCrl, C57BL/6NHsd, C57BL/6NJ, C57BL/6NRj, C57BL/6NTac	0	2	2	100.0%
129S5/SvEvBrd	0	1	5	20.0%
C57BL/6J, C57BL/6JBomTac, C57BL/6JEiJ, C57BL/6JJicTac, C57BL/6JOlaHsd, C57BL/6JRj	0	1	2	50.0%
C57BL/6J, C57BL/6JEiJ, C57BL/6JJicTac, C57BL/6JOlaHsd, C57BL/6JRj	0	1	1	100.0%
C57BL/6NHsd, C57BL/6NJ, C57BL/6NRj, C57BL/6NTac	0	1	1	100.0%
C57BL/6NRj	0	1	10	10.0%

Minimal Strain Sets Explaining All Diagnostic Classes (Number of Markers Explained):

- Solution 1: 129S5/SvEvBrd and C57BL/6J and C57BL/6NRj
    - C57BL/6J: 66 / 162 (40.7%)
    - C57BL/6NRj: 28 / 40 (70.0%)
    - 129S5/SvEvBrd: 1 / 5 (20.0%)
- Solution 2: 129S5/SvEvBrd and C57BL/6JRj and C57BL/6NRj
    - C57BL/6JRj: 66 / 162 (40.7%)
    - C57BL/6NRj: 28 / 40 (70.0%)
    - 129S5/SvEvBrd: 1 / 5 (20.0%)

Chromosome	Start (Mb)	Stop (Mb)	Background	Zygosity
1	3000000	61451021	C57BL/6J and C57BL/6NTac and CBA/J	Heterozygous
1	61451021	90903197	C57BL/6J and C57BL/6NTac	N/A
1	90903197	168019536	C57BL/6J and C57BL/6NTac and CBA/J	Heterozygous
1	168019536	191629867	C57BL/6J and C57BL/6NTac	N/A
1	191629867	195471971	C57BL/6J and C57BL/6NTac and CBA/J	Heterozygous
2	3000000	45666278	C57BL/6J and C57BL/6NTac and CBA/J	Heterozygous
2	45666278	73223831	C57BL/6J and C57BL/6NTac	N/A
2	73223831	154349372	C57BL/6J and C57BL/6NTac and CBA/J	Heterozygous
2	154349372	182113224	C57BL/6J and C57BL/6NTac	N/A
3	3000000	37371933	C57BL/6J and C57BL/6NTac and CBA/J	Heterozygous

MiniMUGA Background Analysis v2.3.1

**Diplotype Intervals**	3	37371933	56655047	C57BL/6J and C57BL/6NTac	N/A
	3	56655047	60850190	C57BL/6J and C57BL/6NTac and CBA/J	Heterozygous
	3	60850190	108381941	C57BL/6J and C57BL/6NTac	N/A
	3	108381941	118919242	C57BL/6J and C57BL/6NTac and CBA/J	Heterozygous
	3	118919242	147169673	C57BL/6J and C57BL/6NTac	N/A
	3	147169673	160039680	C57BL/6J and C57BL/6NTac and CBA/J	Heterozygous
	4	3000000	20258658	C57BL/6J and C57BL/6NTac and CBA/J	Heterozygous
	4	20258658	26280383	C57BL/6J and C57BL/6NTac	N/A
	4	26280383	29346519	C57BL/6J and C57BL/6NTac and CBA/J	Heterozygous
	4	29346519	30650814	Unexplained	Heterozygous
	4	30650814	32327128	C57BL/6J and C57BL/6NTac and CBA/J	Heterozygous
	4	32327128	36784495	C57BL/6J and C57BL/6NTac	N/A
	4	36784495	40531709	Unexplained	Heterozygous
	4	40531709	43372387	C57BL/6J and C57BL/6NTac and CBA/J	Heterozygous
	4	43372387	43819249	Unexplained	Heterozygous
	4	43819249	49280860	C57BL/6J and C57BL/6NTac and CBA/J	Heterozygous
	4	49280860	54114833	C57BL/6J and C57BL/6NTac	N/A
	4	54114833	156508116	C57BL/6J and C57BL/6NTac and CBA/J	Heterozygous
	5	3000000	8810106	C57BL/6J and C57BL/6NTac and CBA/J	Heterozygous
	5	8810106	36875036	C57BL/6J and C57BL/6NTac	N/A
	5	36875036	52975754	C57BL/6J and C57BL/6NTac and CBA/J	Heterozygous
	5	52975754	124446826	C57BL/6J and C57BL/6NTac	N/A
	5	124446826	151834684	C57BL/6J and C57BL/6NTac and CBA/J	Heterozygous
	6	3000000	22152593	C57BL/6J and C57BL/6NTac and CBA/J	Heterozygous
	6	22152593	43184432	C57BL/6J and C57BL/6NTac	N/A
	6	43184432	149736546	C57BL/6J and C57BL/6NTac and CBA/J	Heterozygous
	7	3000000	69096424	C57BL/6J and C57BL/6NTac and CBA/J	Heterozygous
	7	69096424	119823617	C57BL/6J and C57BL/6NTac	N/A
	7	119823617	145441459	C57BL/6J and C57BL/6NTac and CBA/J	Heterozygous
	8	3000000	27348459	C57BL/6J and C57BL/6NTac	N/A

8	27348459	119835722	C57BL/6J and C57BL/6NTac and CBA/J	Heterozygous
8	119835722	129401213	C57BL/6J and C57BL/6NTac	N/A
9	3000000	10507235	C57BL/6J and C57BL/6NTac and CBA/J	Heterozygous
9	10507235	96036921	C57BL/6J and C57BL/6NTac	N/A
9	96036921	100907696	C57BL/6J and C57BL/6NTac and CBA/J	Heterozygous
9	100907696	124595110	C57BL/6J and C57BL/6NTac	N/A
10	3000000	42858234	C57BL/6J and C57BL/6NTac	N/A
10	42858234	61450853	C57BL/6J and C57BL/6NTac and CBA/J	Heterozygous
10	61450853	68332199	C57BL/6J and C57BL/6NTac	N/A
10	68332199	77055656	C57BL/6J and C57BL/6NTac and CBA/J	Heterozygous
10	77055656	130694993	C57BL/6J and C57BL/6NTac	N/A
11	3000000	22302070	C57BL/6J and C57BL/6NTac and CBA/J	Heterozygous
11	22302070	36618681	C57BL/6J and C57BL/6NTac	N/A
11	36618681	112771442	C57BL/6J and C57BL/6NTac and CBA/J	Heterozygous
11	112771442	119038285	C57BL/6J and C57BL/6NTac	N/A
11	119038285	122082543	C57BL/6J and C57BL/6NTac and CBA/J	Heterozygous
12	3000000	47723179	C57BL/6J and C57BL/6NTac and CBA/J	Heterozygous
12	47723179	69789714	C57BL/6J and C57BL/6NTac	N/A
12	69789714	85015902	C57BL/6J and C57BL/6NTac and CBA/J	Heterozygous
12	85015902	88650858	C57BL/6J and C57BL/6NTac	N/A
12	88650858	120129022	C57BL/6J and C57BL/6NTac and CBA/J	Heterozygous
13	3000000	60016573	C57BL/6J and C57BL/6NTac	N/A
13	60016573	72382747	C57BL/6J and C57BL/6NTac and CBA/J	Heterozygous
13	72382747	120421639	C57BL/6J and C57BL/6NTac	N/A
14	3000000	120643228	C57BL/6J and C57BL/6NTac and CBA/J	Heterozygous
14	120643228	124902244	C57BL/6J and C57BL/6NTac	N/A
15	3000000	88538882	C57BL/6J and C57BL/6NTac and CBA/J	Heterozygous
15	88538882	104043685	C57BL/6J and C57BL/6NTac	N/A
16	3000000	60597221	C57BL/6J and C57BL/6NTac	N/A

MiniMUGA Background Analysis v2.3.1

	16	60597221	82429429	C57BL/6J and C57BL/6NTac and CBA/J	Heterozygous
	16	82429429	98207768	C57BL/6J and C57BL/6NTac	N/A
	17	3000000	47545390	C57BL/6J and C57BL/6NTac	N/A
	17	47545390	75218527	C57BL/6J and C57BL/6NTac and CBA/J	Heterozygous
	17	75218527	94987271	C57BL/6J and C57BL/6NTac	N/A
	18	3000000	15685654	C57BL/6J and C57BL/6NTac	N/A
	18	15685654	20363699	Unexplained	Heterozygous
	18	20363699	27036500	C57BL/6J and C57BL/6NTac and CBA/J	Heterozygous
	18	27036500	38237964	C57BL/6J and C57BL/6NTac	N/A
	18	38237964	72051338	C57BL/6J and C57BL/6NTac and CBA/J	Heterozygous
	18	72051338	90702639	C57BL/6J and C57BL/6NTac	N/A
	19	3000000	31636352	C57BL/6J and C57BL/6NTac	N/A
	19	31636352	47746251	C57BL/6J and C57BL/6NTac and CBA/J	Heterozygous
	19	47746251	49870985	C57BL/6J and C57BL/6NTac	N/A
	19	49870985	61431566	C57BL/6J and C57BL/6NTac and CBA/J	Heterozygous
	X	3000000	146651558	C57BL/6J and C57BL/6NTac and CBA/J	Heterozygous
	X	146651558	171031299	C57BL/6J and C57BL/6NTac	N/A
	MT	0	0	IBD	Hemizygous
