# Supplementary material for: Deletion of 9p drives B-ALL through heterozygous inactivation of Pax5 and Cd72 in preleukemic cells
Source: JCI Insight. 2026 Feb 17;11(7):e199464. doi: 10.1172/jci.insight.199464 (PMC13134721; doi:10.1172/jci.insight.199464)
Supplement: Supplemental data set 1 [file jciinsight-11-199464-s204.zip › Strain_Genotyping/X701-results-report.pdf]

MiniMUGA Background Analysis v2.3.1

**Sample ID**	X701
**Neogen ID**	AAAU-4504
**Summary**	The genotype of this sample is of **excellent** quality. It is **XO** and **outbred**, and likely a mix of **C57BL/6J and C57BL/6NTac** and **CBA/J**. Clustering of unexplained markers is evidence of an additional background strain.<br><br>Diagnostic SNPs are likely explained by the presence of the background strains<br>• Solution 1: 129S5/SvEvBrd and C57BL/10ScCr and C57BL/6J and C57BL/6JBomTac and C57BL/6NTac and DBA/2J and DBA/2NCrl<br>◦ C57BL/6J: 72 / 159 (45.3%)<br>◦ C57BL/6NTac: 25 / 29 (86.2%)<br>◦ 129S5/SvEvBrd: 1 / 5 (20.0%)<br>◦ C57BL/10ScCr: 1 / 180 (0.6%)<br>◦ C57BL/6J or C57BL/6JBomTac: 1 / 2 (50.0%)<br>◦ C57BL/6JBomTac: 1 / 40 (2.5%)<br>◦ DBA/2J: 1 / 117 (0.9%)<br>◦ DBA/2NCrl: 1 / 104 (1.0%)<br>• Solution 2: 129S5/SvEvBrd and C57BL/10ScNHsd and C57BL/6J and C57BL/6JBomTac and C57BL/6NTac and DBA/2J and DBA/2NCrl<br>◦ C57BL/6J: 72 / 159 (45.3%)<br>◦ C57BL/6NTac: 25 / 29 (86.2%)<br>◦ 129S5/SvEvBrd: 1 / 5 (20.0%)<br>◦ C57BL/10ScNHsd: 1 / 180 (0.6%)<br>◦ C57BL/6J or C57BL/6JBomTac: 1 / 2 (50.0%)<br>◦ C57BL/6JBomTac: 1 / 40 (2.5%)<br>◦ DBA/2J: 1 / 117 (0.9%)<br>◦ DBA/2NCrl: 1 / 104 (1.0%)<br>• Solution 3: 129S5/SvEvBrd and C57BL/10ScCr and C57BL/6J and C57BL/6JBomTac and C57BL/6NRj and DBA/2J and DBA/2NCrl<br>◦ C57BL/6J: 72 / 159 (45.3%)<br>◦ C57BL/6NRj: 25 / 29 (86.2%)<br>◦ 129S5/SvEvBrd: 1 / 5 (20.0%)<br>◦ C57BL/10ScCr: 1 / 180 (0.6%)<br>◦ C57BL/6J or C57BL/6JBomTac: 1 / 2 (50.0%)<br>◦ C57BL/6JBomTac: 1 / 40 (2.5%)<br>◦ DBA/2J: 1 / 117 (0.9%)<br>◦ DBA/2NCrl: 1 / 104 (1.0%)<br>• Solution 4: 129S5/SvEvBrd and C57BL/10ScCr and C57BL/6JBomTac and C57BL/6JRj and C57BL/6NTac and DBA/2J and DBA/2NCrl<br>◦ C57BL/6JRj: 72 / 159 (45.3%)<br>◦ C57BL/6NTac: 25 / 29 (86.2%)<br>◦ 129S5/SvEvBrd: 1 / 5 (20.0%)<br>◦ C57BL/10ScCr: 1 / 180 (0.6%)<br>◦ C57BL/6JBomTac or C57BL/6JRj: 1 / 2 (50.0%)<br>◦ C57BL/6JBomTac: 1 / 40 (2.5%)<br>◦ DBA/2J: 1 / 117 (0.9%)<br>◦ DBA/2NCrl: 1 / 104 (1.0%)<br>• Solution 5: 129S5/SvEvBrd and C57BL/10ScNHsd and C57BL/6J and C57BL/6JBomTac and C57BL/6NRj and DBA/2J and DBA/2NCrl<br>◦ C57BL/6J: 72 / 159 (45.3%)<br>◦ C57BL/6NRj: 25 / 29 (86.2%)<br>◦ 129S5/SvEvBrd: 1 / 5 (20.0%)<br>◦ C57BL/10ScNHsd: 1 / 180 (0.6%)<br>◦ C57BL/6J or C57BL/6JBomTac: 1 / 2 (50.0%)<br>◦ C57BL/6JBomTac: 1 / 40 (2.5%)<br>◦ DBA/2J: 1 / 117 (0.9%)<br>◦ DBA/2NCrl: 1 / 104 (1.0%)<br>• Solution 6: 129S5/SvEvBrd and C57BL/10ScNHsd and C57BL/6JBomTac and C57BL/6JRj and C57BL/6NTac and DBA/2J and DBA/2NCrl

MiniMUGA Background Analysis v2.3.1

	◦ C57BL/6JRj: 72 / 159 (45.3%)<br>◦ C57BL/6NTac: 25 / 29 (86.2%)<br>◦ 129S5/SvEvBrd: 1 / 5 (20.0%)<br>◦ C57BL/10ScNHsd: 1 / 180 (0.6%)<br>◦ C57BL/6JBomTac or C57BL/6JRj: 1 / 2 (50.0%)<br>◦ C57BL/6JBomTac: 1 / 40 (2.5%)<br>◦ DBA/2J: 1 / 117 (0.9%)<br>◦ DBA/2NCrl: 1 / 104 (1.0%)<br>• Solution 7: 129S5/SvEvBrd and C57BL/10ScCr and C57BL/6JBomTac and C57BL/6JRj and C57BL/6NRj and DBA/2J and DBA/2NCrl<br>◦ C57BL/6JRj: 72 / 159 (45.3%)<br>◦ C57BL/6NRj: 25 / 29 (86.2%)<br>◦ 129S5/SvEvBrd: 1 / 5 (20.0%)<br>◦ C57BL/10ScCr: 1 / 180 (0.6%)<br>◦ C57BL/6JBomTac or C57BL/6JRj: 1 / 2 (50.0%)<br>◦ C57BL/6JBomTac: 1 / 40 (2.5%)<br>◦ DBA/2J: 1 / 117 (0.9%)<br>◦ DBA/2NCrl: 1 / 104 (1.0%)<br>• Solution 8: 129S5/SvEvBrd and C57BL/10ScNHsd and C57BL/6JBomTac and C57BL/6JRj and C57BL/6NRj and DBA/2J and DBA/2NCrl<br>◦ C57BL/6JRj: 72 / 159 (45.3%)<br>◦ C57BL/6NRj: 25 / 29 (86.2%)<br>◦ 129S5/SvEvBrd: 1 / 5 (20.0%)<br>◦ C57BL/10ScNHsd: 1 / 180 (0.6%)<br>◦ C57BL/6JBomTac or C57BL/6JRj: 1 / 2 (50.0%)<br>◦ C57BL/6JBomTac: 1 / 40 (2.5%)<br>◦ DBA/2J: 1 / 117 (0.9%)<br>◦ DBA/2NCrl: 1 / 104 (1.0%)<br><br>NOTE: There is a discrepancy between the diagnostic backgrounds detected and the primary and secondary background analysis (CBA/J, C57BL/6J, C57BL/6NTac). This is uncommon and should be investigated further.<br><br>No genetic constructs were detected in this sample.<br><br>WARNING:<br>• There is a discrepancy between the diagnostic backgrounds detected ((129S5/SvEvBrd and C57BL/10ScCr and C57BL/6J and C57BL/6JBomTac and C57BL/6NTac and DBA/2J and DBA/2NCrl) or (129S5/SvEvBrd and C57BL/10ScNHsd and C57BL/6J and C57BL/6JBomTac and C57BL/6NTac and DBA/2J and DBA/2NCrl) or (129S5/SvEvBrd and C57BL/10ScCr and C57BL/6J and C57BL/6JBomTac and C57BL/6NRj and DBA/2J and DBA/2NCrl) or (129S5/SvEvBrd and C57BL/10ScCr and C57BL/6JBomTac and C57BL/6JRj and C57BL/6NTac and DBA/2J and DBA/2NCrl) or (129S5/SvEvBrd and C57BL/10ScNHsd and C57BL/6J and C57BL/6JBomTac and C57BL/6NRj and DBA/2J and DBA/2NCrl) or (129S5/SvEvBrd and C57BL/10ScNHsd and C57BL/6JBomTac and C57BL/6JRj and C57BL/6NTac and DBA/2J and DBA/2NCrl) or (129S5/SvEvBrd and C57BL/10ScCr and C57BL/6JBomTac and C57BL/6JRj and C57BL/6NRj and DBA/2J and DBA/2NCrl) or (129S5/SvEvBrd and C57BL/10ScNHsd and C57BL/6JBomTac and C57BL/6JRj and C57BL/6NRj and DBA/2J and DBA/2NCrl)) and the primary background (C57BL/6J and C57BL/6NTac) and secondary background (CBA/J). This is uncommon and should be investigated further.<br>• The presence of a single diagnostic heterozygous call for a single inbred strain should be treated with caution.<br>• This sample likely has more than 2 genetic backgrounds (unexplained regions and/or fractured ideogram). The strain selected for secondary background may be incorrect. The estimation of the contribution of primary and secondary background are likely incorrect. This can potentially be addressed with input from the user.
**Genotyping Quality**	**Excellent (88 N calls)**<br>All reported results are dependent on genotyping quality.
**Chromosomal Sex**	XO

MiniMUGA Background Analysis v2.3.1

Inbreeding Estimate

46.7% Inbred
(Percentage of the genome (autosomal and X chromosomes) that is homozygous or hemizygous for primary, secondary, and unknown backgrounds. See Genome Analysis)

Constructs Detected

BlastR	bpA	Cas9	chlor	cHS4	Cre	DTA	Flp	g_FP	hCMV_a	hCMV_b	hTK_pr	iCre	IRES	Luc	r_FP	rtTA	SV40	tTA
-	-	-	-	-	-	-	-	-	-	-	-	-	-	-	-	-	-	-

* This sample has abnormal raw intensity values. Therefore, positive or questionable returns for constructs should be treated with caution.

Refined Ideogram

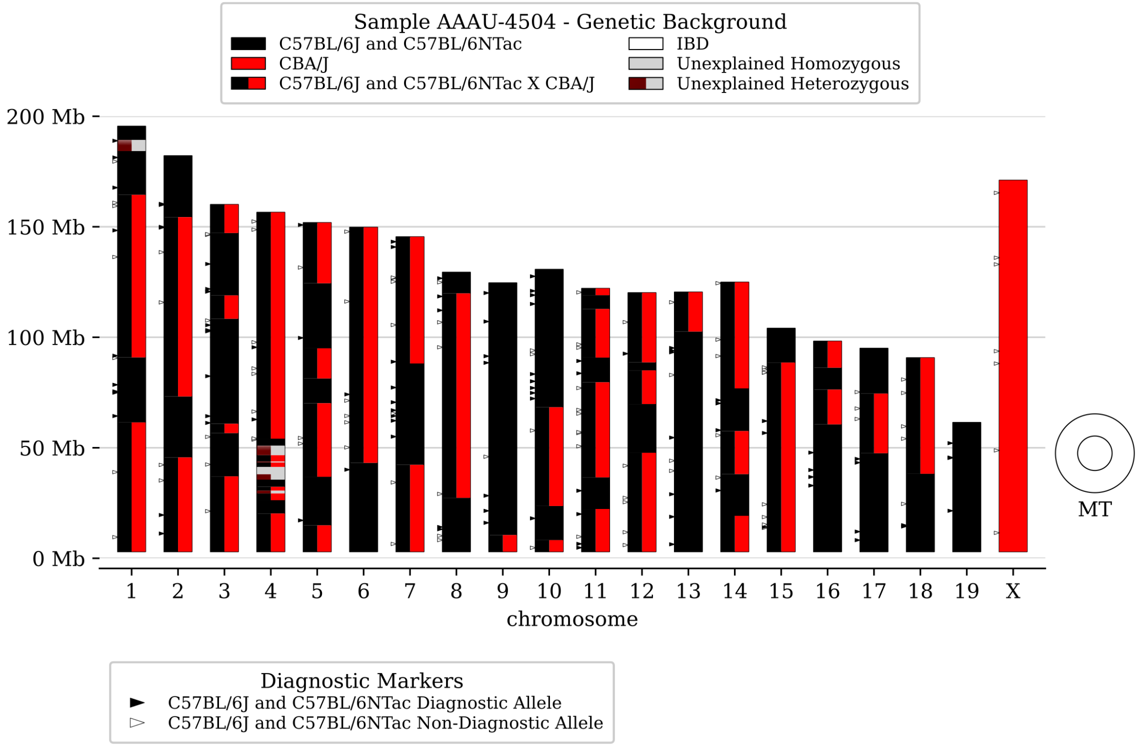

Genome Analysis

Background	Zygosity	Informative Markers	Informative Markers %	Genome %
C57BL/6J and C57BL/6NTac	N/A	1150	43.7%	40.0%
CBA/J	Homozygous	1	0.0%	0.0%
C57BL/6J and C57BL/6NTac X CBA/J	Heterozygous	1444	54.9%	59.3%
Unexplained	Homozygous	5	0.2%	0.1%
Unexplained	Heterozygous	31	1.2%	0.5%
	**Total**	2631	100.0%	99.9%

Y Chromosome

Not Applicable

MT Genome

MT Haplogroup 6 - 100.0% Consistent
Includes C57BL/6J, C57BL/6NTac, CBA/J and 165 other strains

MiniMUGA Background Analysis v2.3.1

Backgrounds Detected (Diagnostic Alleles)

	Diagnostic Alleles Observed			
**Diagnostic Class**	**Homozygous**	**Heterozygous**	**Potential**	**% Observed**
C57BL/6J, C57BL/6JJicTac, C57BL/6JRj	1	49	102	49.0%
C57BL/6J, C57BL/6JRj	0	11	31	35.5%
C57BL/6NJ, C57BL/6NRj, C57BL/6NTac	1	9	10	100.0%
C57BL/6NRj, C57BL/6NTac	0	11	15	73.3%
C57BL/6J, C57BL/6JEiJ, C57BL/6JJicTac, C57BL/6JRj	0	8	21	38.1%
B6N-Tyr<c-Brd>/BrdCrCrl, C57BL/6J, C57BL/6JJicTac, C57BL/6JRj	0	3	5	60.0%
B6N-Tyr<c-Brd>/BrdCrCrl, C57BL/6NCrl, C57BL/6NHsd, C57BL/6NJ, C57BL/6NRj, C57BL/6NTac	0	2	2	100.0%
C57BL/6NCrl, C57BL/6NHsd, C57BL/6NJ, C57BL/6NRj, C57BL/6NTac	0	2	2	100.0%
129S5/SvEvBrd	0	1	5	20.0%
C57BL/10ScCr, C57BL/10ScNHsd	0	1	180	0.6%
C57BL/6J, C57BL/6JBomTac, C57BL/6JEiJ, C57BL/6JJicTac, C57BL/6JOlaHsd, C57BL/6JRj	0	1	2	50.0%
C57BL/6JBomTac	0	1	40	2.5%
DBA/2J	0	1	117	0.9%
DBA/2NCrl	0	1	104	1.0%

Minimal Strain Sets Explaining All Diagnostic Classes (Number of Markers Explained):

- Solution 1: 129S5/SvEvBrd and C57BL/10ScCr and C57BL/6J and C57BL/6JBomTac and C57BL/6NTac and DBA/2J and DBA/2NCrl
  - C57BL/6J: 72 / 159 (45.3%)
  - C57BL/6NTac: 25 / 29 (86.2%)
  - 129S5/SvEvBrd: 1 / 5 (20.0%)
  - C57BL/10ScCr: 1 / 180 (0.6%)
  - C57BL/6J or C57BL/6JBomTac: 1 / 2 (50.0%)
  - C57BL/6JBomTac: 1 / 40 (2.5%)
  - DBA/2J: 1 / 117 (0.9%)
  - DBA/2NCrl: 1 / 104 (1.0%)
- Solution 2: 129S5/SvEvBrd and C57BL/10ScNHsd and C57BL/6J and C57BL/6JBomTac and C57BL/6NTac and DBA/2J and DBA/2NCrl
  - C57BL/6J: 72 / 159 (45.3%)
  - C57BL/6NTac: 25 / 29 (86.2%)
  - 129S5/SvEvBrd: 1 / 5 (20.0%)
  - C57BL/10ScNHsd: 1 / 180 (0.6%)
  - C57BL/6J or C57BL/6JBomTac: 1 / 2 (50.0%)
  - C57BL/6JBomTac: 1 / 40 (2.5%)
  - DBA/2J: 1 / 117 (0.9%)
  - DBA/2NCrl: 1 / 104 (1.0%)
- Solution 3: 129S5/SvEvBrd and C57BL/10ScCr and C57BL/6J and C57BL/6JBomTac and C57BL/6NRj and DBA/2J and DBA/2NCrl
  - C57BL/6J: 72 / 159 (45.3%)
  - C57BL/6NRj: 25 / 29 (86.2%)
  - 129S5/SvEvBrd: 1 / 5 (20.0%)
  - C57BL/10ScCr: 1 / 180 (0.6%)
  - C57BL/6J or C57BL/6JBomTac: 1 / 2 (50.0%)
  - C57BL/6JBomTac: 1 / 40 (2.5%)
  - DBA/2J: 1 / 117 (0.9%)
  - DBA/2NCrl: 1 / 104 (1.0%)

- Solution 4: 129S5/SvEvBrd and C57BL/10ScCr and C57BL/6JBomTac and C57BL/6JRj and C57BL/6NTac and DBA/2J and DBA/2NCrl
    - C57BL/6JRj: 72 / 159 (45.3%)
    - C57BL/6NTac: 25 / 29 (86.2%)
    - 129S5/SvEvBrd: 1 / 5 (20.0%)
    - C57BL/10ScCr: 1 / 180 (0.6%)
    - C57BL/6JBomTac or C57BL/6JRj: 1 / 2 (50.0%)
    - C57BL/6JBomTac: 1 / 40 (2.5%)
    - DBA/2J: 1 / 117 (0.9%)
    - DBA/2NCrl: 1 / 104 (1.0%)
- Solution 5: 129S5/SvEvBrd and C57BL/10ScNHsd and C57BL/6J and C57BL/6JBomTac and C57BL/6NRj and DBA/2J and DBA/2NCrl
    - C57BL/6J: 72 / 159 (45.3%)
    - C57BL/6NRj: 25 / 29 (86.2%)
    - 129S5/SvEvBrd: 1 / 5 (20.0%)
    - C57BL/10ScNHsd: 1 / 180 (0.6%)
    - C57BL/6J or C57BL/6JBomTac: 1 / 2 (50.0%)
    - C57BL/6JBomTac: 1 / 40 (2.5%)
    - DBA/2J: 1 / 117 (0.9%)
    - DBA/2NCrl: 1 / 104 (1.0%)
- Solution 6: 129S5/SvEvBrd and C57BL/10ScNHsd and C57BL/6JBomTac and C57BL/6JRj and C57BL/6NTac and DBA/2J and DBA/2NCrl
    - C57BL/6JRj: 72 / 159 (45.3%)
    - C57BL/6NTac: 25 / 29 (86.2%)
    - 129S5/SvEvBrd: 1 / 5 (20.0%)
    - C57BL/10ScNHsd: 1 / 180 (0.6%)
    - C57BL/6JBomTac or C57BL/6JRj: 1 / 2 (50.0%)
    - C57BL/6JBomTac: 1 / 40 (2.5%)
    - DBA/2J: 1 / 117 (0.9%)
    - DBA/2NCrl: 1 / 104 (1.0%)
- Solution 7: 129S5/SvEvBrd and C57BL/10ScCr and C57BL/6JBomTac and C57BL/6JRj and C57BL/6NRj and DBA/2J and DBA/2NCrl
    - C57BL/6JRj: 72 / 159 (45.3%)
    - C57BL/6NRj: 25 / 29 (86.2%)
    - 129S5/SvEvBrd: 1 / 5 (20.0%)
    - C57BL/10ScCr: 1 / 180 (0.6%)
    - C57BL/6JBomTac or C57BL/6JRj: 1 / 2 (50.0%)
    - C57BL/6JBomTac: 1 / 40 (2.5%)
    - DBA/2J: 1 / 117 (0.9%)
    - DBA/2NCrl: 1 / 104 (1.0%)
- Solution 8: 129S5/SvEvBrd and C57BL/10ScNHsd and C57BL/6JBomTac and C57BL/6JRj and C57BL/6NRj and DBA/2J and DBA/2NCrl
    - C57BL/6JRj: 72 / 159 (45.3%)
    - C57BL/6NRj: 25 / 29 (86.2%)
    - 129S5/SvEvBrd: 1 / 5 (20.0%)
    - C57BL/10ScNHsd: 1 / 180 (0.6%)
    - C57BL/6JBomTac or C57BL/6JRj: 1 / 2 (50.0%)
    - C57BL/6JBomTac: 1 / 40 (2.5%)
    - DBA/2J: 1 / 117 (0.9%)
    - DBA/2NCrl: 1 / 104 (1.0%)

Chromosome	Start (Mb)	Stop (Mb)	Background	Zygosity
1	3000000	61451021	C57BL/6J and C57BL/6NTac and CBA/J	Heterozygous
1	61451021	90903197	C57BL/6J and C57BL/6NTac	N/A
1	90903197	164435178	C57BL/6J and C57BL/6NTac and CBA/J	Heterozygous

MiniMUGA Background Analysis v2.3.1

Diplotype Intervals	1	164435178	184243385	C57BL/6J and C57BL/6NTac	N/A
	1	184243385	189310430	Unexplained	Heterozygous
	1	189310430	195471971	C57BL/6J and C57BL/6NTac	N/A
	2	3000000	45620442	C57BL/6J and C57BL/6NTac and CBA/J	Heterozygous
	2	45620442	73223831	C57BL/6J and C57BL/6NTac	N/A
	2	73223831	154349372	C57BL/6J and C57BL/6NTac and CBA/J	Heterozygous
	2	154349372	182113224	C57BL/6J and C57BL/6NTac	N/A
	3	3000000	37065410	C57BL/6J and C57BL/6NTac and CBA/J	Heterozygous
	3	37065410	56655047	C57BL/6J and C57BL/6NTac	N/A
	3	56655047	60850190	C57BL/6J and C57BL/6NTac and CBA/J	Heterozygous
	3	60850190	108381941	C57BL/6J and C57BL/6NTac	N/A
	3	108381941	118919242	C57BL/6J and C57BL/6NTac and CBA/J	Heterozygous
	3	118919242	147169673	C57BL/6J and C57BL/6NTac	N/A
	3	147169673	160039680	C57BL/6J and C57BL/6NTac and CBA/J	Heterozygous
	4	3000000	20258658	C57BL/6J and C57BL/6NTac and CBA/J	Heterozygous
	4	20258658	26280383	C57BL/6J and C57BL/6NTac	N/A
	4	26280383	29346519	C57BL/6J and C57BL/6NTac and CBA/J	Heterozygous
	4	29346519	30650814	Unexplained	Heterozygous
	4	30650814	32327128	C57BL/6J and C57BL/6NTac and CBA/J	Heterozygous
	4	32327128	35563307	C57BL/6J and C57BL/6NTac	N/A
	4	35563307	37995481	Unexplained	Heterozygous
	4	37995481	41348396	Unexplained	Homozygous
	4	41348396	43372387	C57BL/6J and C57BL/6NTac and CBA/J	Heterozygous
	4	43372387	43819249	Unexplained	Heterozygous
	4	43819249	46665692	C57BL/6J and C57BL/6NTac and CBA/J	Heterozygous
	4	46665692	50929602	Unexplained	Heterozygous
	4	50929602	54114833	C57BL/6J and C57BL/6NTac	N/A
	4	54114833	156508116	C57BL/6J and C57BL/6NTac and CBA/J	Heterozygous
	5	3000000	14885741	C57BL/6J and C57BL/6NTac and CBA/J	Heterozygous
	5	14885741	36875036	C57BL/6J and C57BL/6NTac	N/A
	5	36875036	70142638	C57BL/6J and C57BL/6NTac and CBA/J	Heterozygous

5	70142638	81363817	C57BL/6J and C57BL/6NTac	N/A
5	81363817	94997713	C57BL/6J and C57BL/6NTac and CBA/J	Heterozygous
5	94997713	124446826	C57BL/6J and C57BL/6NTac	N/A
5	124446826	151834684	C57BL/6J and C57BL/6NTac and CBA/J	Heterozygous
6	3000000	43184432	C57BL/6J and C57BL/6NTac	N/A
6	43184432	149736546	C57BL/6J and C57BL/6NTac and CBA/J	Heterozygous
7	3000000	42273938	C57BL/6J and C57BL/6NTac and CBA/J	Heterozygous
7	42273938	88139775	C57BL/6J and C57BL/6NTac	N/A
7	88139775	145441459	C57BL/6J and C57BL/6NTac and CBA/J	Heterozygous
8	3000000	27348459	C57BL/6J and C57BL/6NTac	N/A
8	27348459	119835722	C57BL/6J and C57BL/6NTac and CBA/J	Heterozygous
8	119835722	129401213	C57BL/6J and C57BL/6NTac	N/A
9	3000000	10507235	C57BL/6J and C57BL/6NTac and CBA/J	Heterozygous
9	10507235	124595110	C57BL/6J and C57BL/6NTac	N/A
10	3000000	8205640	C57BL/6J and C57BL/6NTac and CBA/J	Heterozygous
10	8205640	23654421	C57BL/6J and C57BL/6NTac	N/A
10	23654421	68332199	C57BL/6J and C57BL/6NTac and CBA/J	Heterozygous
10	68332199	130694993	C57BL/6J and C57BL/6NTac	N/A
11	3000000	22302070	C57BL/6J and C57BL/6NTac and CBA/J	Heterozygous
11	22302070	36618681	C57BL/6J and C57BL/6NTac	N/A
11	36618681	79617327	C57BL/6J and C57BL/6NTac and CBA/J	Heterozygous
11	79617327	90803561	C57BL/6J and C57BL/6NTac	N/A
11	90803561	112771442	C57BL/6J and C57BL/6NTac and CBA/J	Heterozygous
11	112771442	119038285	C57BL/6J and C57BL/6NTac	N/A
11	119038285	122082543	C57BL/6J and C57BL/6NTac and CBA/J	Heterozygous
12	3000000	47723179	C57BL/6J and C57BL/6NTac and CBA/J	Heterozygous
12	47723179	69789714	C57BL/6J and C57BL/6NTac	N/A
12	69789714	85015902	C57BL/6J and C57BL/6NTac and CBA/J	Heterozygous
12	85015902	88650858	C57BL/6J and C57BL/6NTac	N/A

MiniMUGA Background Analysis v2.3.1

	12	88650858	120129022	C57BL/6J and C57BL/6NTac and CBA/J	Heterozygous
	13	3000000	102595519	C57BL/6J and C57BL/6NTac	N/A
	13	102595519	120421639	C57BL/6J and C57BL/6NTac and CBA/J	Heterozygous
	14	3000000	19213621	C57BL/6J and C57BL/6NTac and CBA/J	Heterozygous
	14	19213621	38092288	C57BL/6J and C57BL/6NTac	N/A
	14	38092288	57544602	C57BL/6J and C57BL/6NTac and CBA/J	Heterozygous
	14	57544602	76871639	C57BL/6J and C57BL/6NTac	N/A
	14	76871639	124902244	C57BL/6J and C57BL/6NTac and CBA/J	Heterozygous
	15	3000000	88538882	C57BL/6J and C57BL/6NTac and CBA/J	Heterozygous
	15	88538882	104043685	C57BL/6J and C57BL/6NTac	N/A
	16	3000000	60597221	C57BL/6J and C57BL/6NTac	N/A
	16	60597221	76315797	C57BL/6J and C57BL/6NTac and CBA/J	Heterozygous
	16	76315797	86241389	C57BL/6J and C57BL/6NTac	N/A
	16	86241389	98207768	C57BL/6J and C57BL/6NTac and CBA/J	Heterozygous
	17	3000000	47545390	C57BL/6J and C57BL/6NTac	N/A
	17	47545390	74502727	C57BL/6J and C57BL/6NTac and CBA/J	Heterozygous
	17	74502727	94987271	C57BL/6J and C57BL/6NTac	N/A
	18	3000000	38237964	C57BL/6J and C57BL/6NTac	N/A
	18	38237964	90702639	C57BL/6J and C57BL/6NTac and CBA/J	Heterozygous
	19	3000000	61431566	C57BL/6J and C57BL/6NTac	N/A
	X	3000000	171031299	C57BL/6J and C57BL/6NTac and CBA/J	Hemizygous
	MT	0	0	IBD	Hemizygous
