# Supplementary material for: Deletion of 9p drives B-ALL through heterozygous inactivation of Pax5 and Cd72 in preleukemic cells
Source: JCI Insight. 2026 Feb 17;11(7):e199464. doi: 10.1172/jci.insight.199464 (PMC13134721; doi:10.1172/jci.insight.199464)

Figure 1E

Primers
VHJ558-Forward
JH3-Reverse

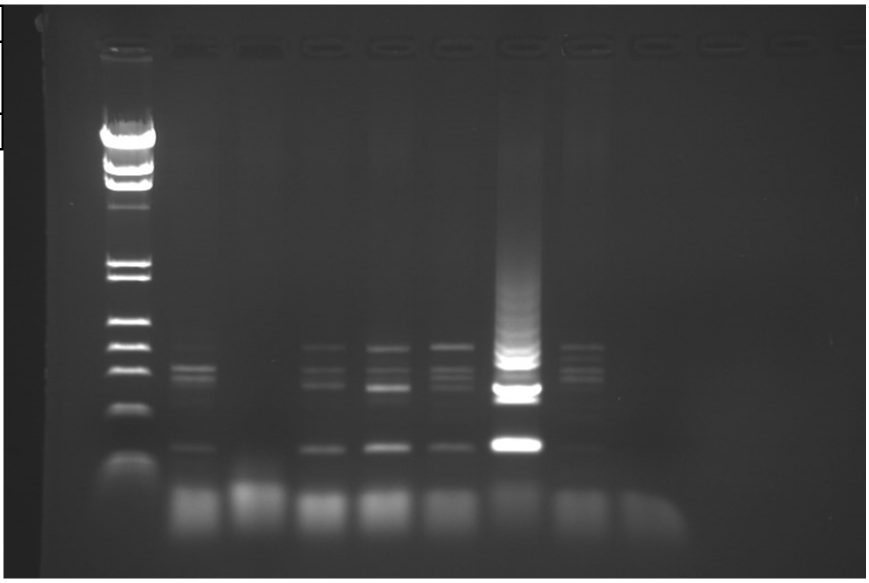

Primers
VH7183-Forward
JH3-Reverse

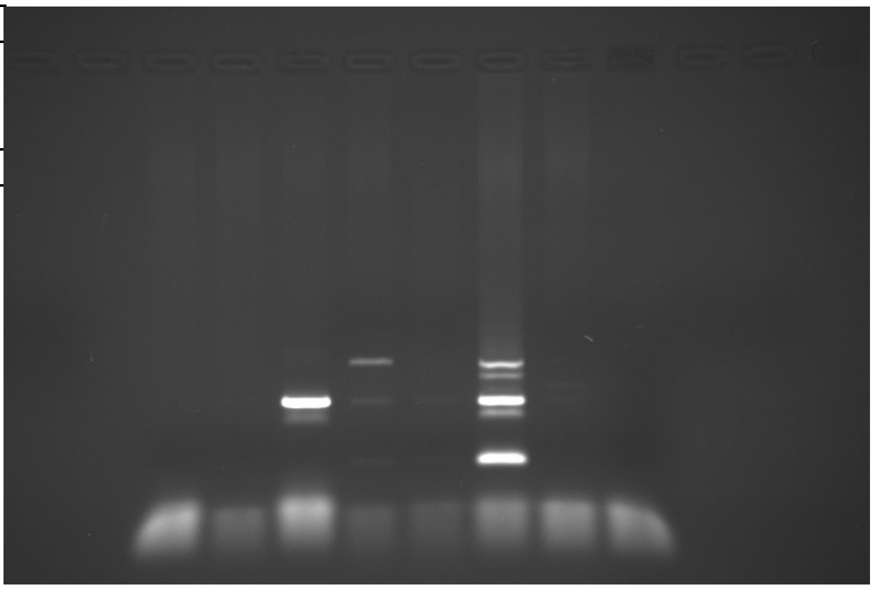

Primers
VHQ52-Forward
JH3-Reverse

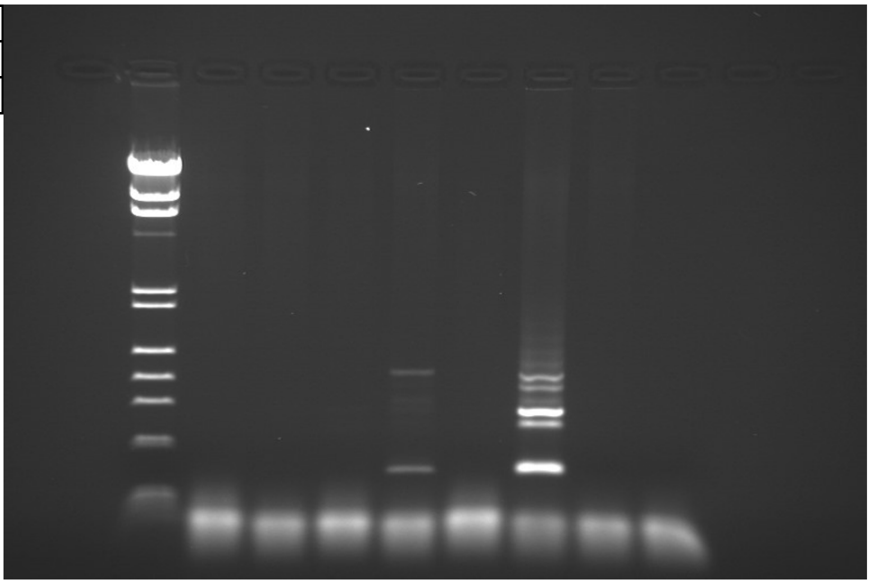

Primers
VHGam3.8-Forward
JH3-Reverse

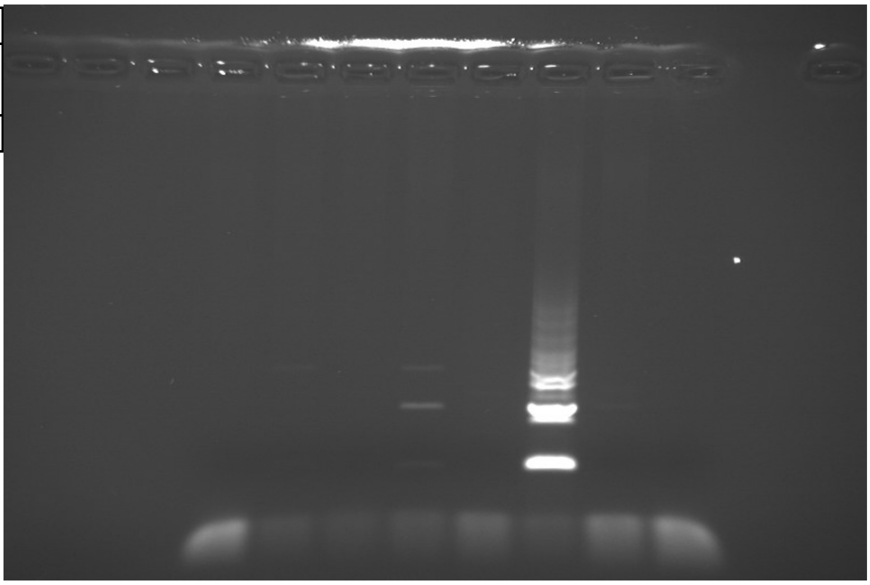

Primers
V_H3609-Forward
JH3-Reverse

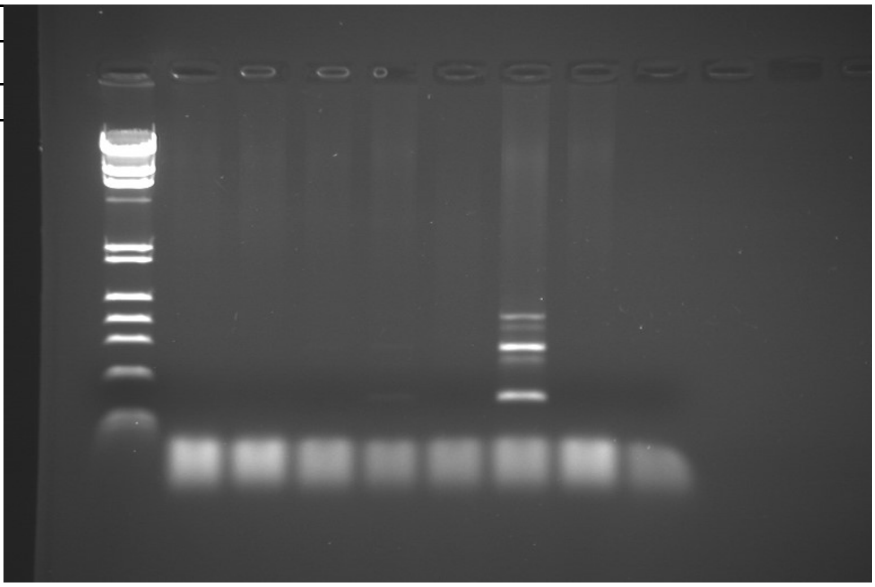

Primers
JH3-Reverse

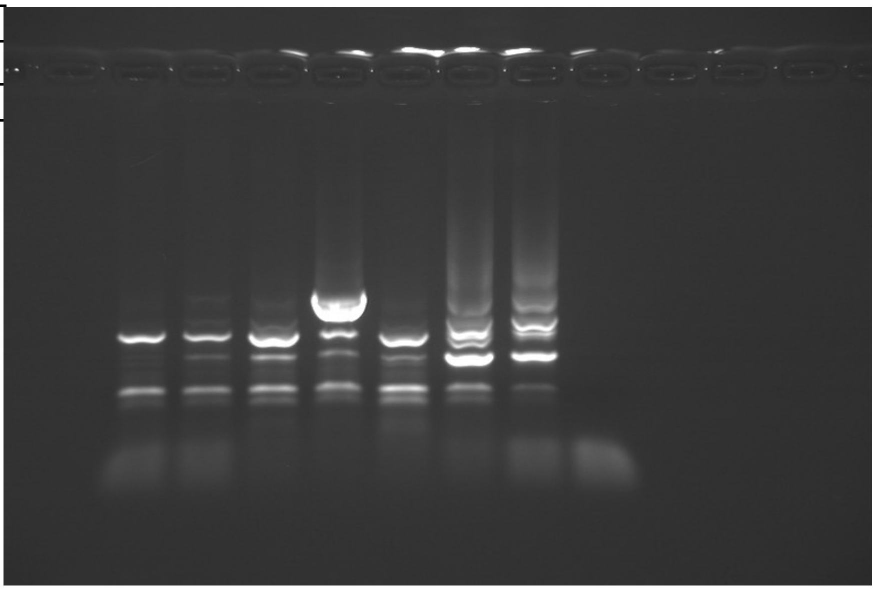

Primers
Cmu-Forward
Cmu-Reverse

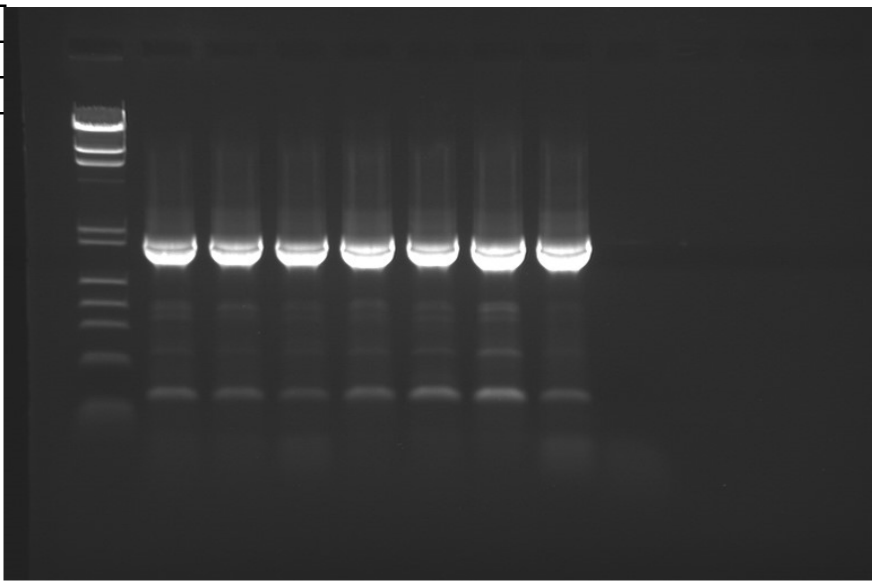

Supplement: Unedited blot and gel images [file jciinsight-11-199464-s205.pdf]
